# Catalytic Asymmetric Cyclizative Rearrangement of Anilines and Vicinal Diketones to Access 2,2‐Disubstituted Indolin‐3‐ones

**DOI:** 10.1002/advs.202402532

**Published:** 2024-04-24

**Authors:** Rui Quan, Xing‐Zi Li, Zi‐Qi Wang, Yu‐Ping He, Hua Wu

**Affiliations:** ^1^ Shanghai Frontiers Science Center for Drug Target Identification and Delivery National Key Laboratory of Innovative Immunotherapy, and Shanghai Key Laboratory for Molecular Engineering of Chiral Drugs School of Pharmaceutical Sciences Shanghai Jiao Tong University 800 Dongchuan Road, Minhang Shanghai 200240 China; ^2^ Department of Chemistry College of Sciences Shanghai University Shanghai 200444 China

**Keywords:** 1,2‐rearrangement, anilines, indolin‐3‐ones, organocatalysis, quaternary stereocenter

## Abstract

The efficient synthesis of chiral 2,2‐disubstituted indolin‐3‐ones is of great importance due to its significant synthetic and biological applications. However, catalytic enantioselective methods for de novo synthesis of such heterocycles remain scarce. Herein, a novel cyclizative rearrangement of readily available anilines and vicinal diketones for the one‐step construction of enantioenriched 2,2‐disubstituted indolin‐3‐ones is presented. The reaction proceeds through a self‐sorted [3+2] heteroannulation/regioselective dehydration/1,2‐ester shift process. Only chiral phosphoric acid is employed to promote the entire sequence and simplify the manipulation of this protocol. Various common aniline derivatives are successfully applied to asymmetric synthesis as 1,3‐binuclephiles for the first time. Remarkably, the observed stereoselectivity is proposed to originate from an amine‐directed regio‐ and enantioselective *ortho*‐Csp^2^‐H addition of the anilines to the ketones. A range of synthetic transformations of the resulting products are demonstrated as well.

## Graphical abstract

Anilines are successfully applied to a catalytic asymmetric cyclizative rearrangement (CACR) sequence, which enables straightforward access to 2,2‐disubstituted indolin‐3‐ones in high yield and enantiopurity with adequate structural diversity. A high regio‐ and stereoselective *ortho*‐carbon addition of anilines to ketones is the key to the success of this overall sequence.

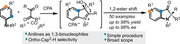

## 1 Introduction

**Scheme 1:**
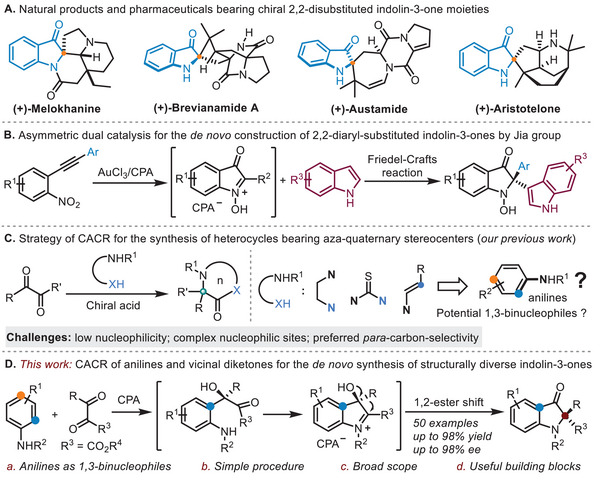
Occurrence of 2,2‐disubstituted indolin‐3‐ones and asymmetric catalytic de novo synthesis.

Chiral 2,2‐disubstituted indolin‐3‐one represents an important class of the heterocyclic skeleton that occurs frequently in natural alkaloids, clinical drugs, and commercial dyes, such as (+)‐Melokhanine,^[^ ^1^ ^]^ (+)‐Brevianamide A,^[^ ^2^ ^]^ (+)‐Austamide^[^ ^3^ ^]^ and (+)‐Aristotelone^[^ ^4^ ^]^ (**Scheme** **1**A). Moreover, it is also a versatile synthetic intermediate usually employed in the synthesis of pharmaceuticals, pesticides, fluorescence probes, and solar‐cell materials.^[^ ^5^, ^6^, ^7^ ^]^ Therefore, considerable effort has been dedicated to its catalytic asymmetric synthesis over the years. In this context, a strategy based on the derivation of the preconstructed indolin‐3‐one scaffolds, mainly centering on 2‐aryl‐3*H*‐indol‐3‐ones and 2‐mono‐substituted indolin‐3‐ones,^[^ ^8^ ^]^ has been widely adopted to access this important family of chiral *N*‐heterocycles, but it relies heavily on the availability of the raw material 2‐substituted indoles and the products are highly restricted to 2‐aryl‐ or 2,2‐diaryl‐substituted indolin‐3‐ones. On the other hand, however, there are very few reports dealing with the de novo synthesis of chiral 2,2‐disubstituted indolin‐3‐ones from two achiral linear compounds. In this regard, representatively, Jia et al. reported an elegant synthesis of *N*‐hydroxylated 2,2‐diaryl‐substituted indolin‐3‐ones via tandem formal heteroannulation/asymmetric Friedel‐Crafts reaction under gold/chiral phosphoric acid dual catalysis (Scheme 1B).^[^ ^9^ ^]^ Alternatively, asymmetric enzyme‐catalyzed oxidative cross‐coupling of indoles with different reaction partners, including ketones and indoles, was also documented.^[^ ^10^ ^]^ Notably, all these examples featured either arylation or alkylation of the in situ formed 2‐substituted indol‐3‐ones as the enantiodetermining step, and the resulting products were basically limited to 2‐aryl‐substituted indolin‐3‐ones as well. Undoubtedly, investigating the useful and general methods for the de novo construction of structurally diverse indolin‐3‐ones bearing quaternary stereocenters from readily available substrates is still highly interesting and desirable.

The asymmetric 1,2‐rearrangement is a robust method for stereoselectively building carbon─carbon bonds.^[^ ^11^ ^]^ Recently, on the basis of it, a range of important stereocenters and building blocks have been efficiently constructed. Despite these significant advances, however, catalytic asymmetric 1,2‐rearrangements have rarely been successfully applied to the synthesis of enantioenriched heterocycles. Recently, we disclosed a novel strategy of catalytic asymmetric cyclizative rearrangement (CACR) which enabled the efficient construction of a series of synthetically challenging heterocycles bearing aza‐quaternary stereocenters (Scheme 1C).^[^ ^12^, ^13^, ^14^ ^]^ Aimed toward a general and flexible strategy for the synthesis of enantiopure 2,2‐disubstituted indolin‐3‐ones, we decided to pursue an alternative approach, that is, the development of a CACR of aniline derivatives and vicinal diketones. This strategy can in principle operate on diverse substituted substrates, and the resulting products are synthetically versatile due to the presence of functionalizable ester derivatives. It is worth noting that aniline is one of the most commonly used substrates in organic synthesis, but it has rarely been employed as a potential 1,3‐binucleophile in the area of asymmetric catalysis due to various intrinsic difficulties, such as low nucleophilicity, multiple nucleophilic sites, preferred *para*‐carbon‐selectivity as well as compatibility issues of amine.^[^ ^15^ ^]^ Among them, overriding aniline‐inherent regioselectivities by means of catalyst control is undoubtedly the most challenging.^[^ ^16^ ^]^ It is foreseeable that overcoming this formidable challenge would not only furnish direct access to more diverse building blocks but also significantly expand the synthetic utility of the anilines. On the other hand, as specific aromatic amines, the more nucleophilic α‐ and β‐naphthylamines with fixed *ortho*‐nucleophilic‐site have been used as 1,3‐binucleophiles for few enantioselective cyclization reactions.^[^ ^17^, ^18^ ^]^ We report herein the successful realization of this endeavor via chiral phosphoric acid catalyzed CACR of diverse anilines and vicinal diketones using a simple procedure, which provides a modular and general synthesis of 2,2‐disubstituted indolin‐3‐ones in a high level of yield and enantiopurity (Scheme 1D). Mechanistic studies as well as further synthetic transformations of the resulting products were also performed.

## 2 Results and Discussion

After a series of initial trials (Tables S1–S6, Supporting Information), we set out to optimize the model cyclizative rearrangement between *meta*‐benzyloxy‐substituted aniline **1a** (1.0 equiv)^[^ ^19^ ^]^ and 2,3‐diketoester **2a** (1.0 equiv)^[^ ^20^ ^]^ under the catalysis of chiral phosphoric acid (CPA)^[^ ^21^ ^]^ in methyl *tert*‐butyl ether (TBME, *c* 0.1 m) at 60 °C with 4 Å molecular sieves (300 mg mmol^−1^) as dehydrating additive (**Table** **1**). In the beginning, with the extensively used chiral acid **4a**, the desired 2,2‐disubstituted indolin‐3‐one **3a** was successfully obtained in good yield and enantioselectivity (entry 1). Subsequently, the structure of the CPA was fine‐tuned (entries 2–6), and chiral phosphoric acid **4f** stood out as the best catalyst in terms of both yield and enantioselectivity (entry 6). With **4f** (0.1 equiv) as a catalyst, other conditions were surveyed varying the additives, temperature, and solvent (entries 7–14), which gave no further improvement on the reaction outcome. Finally, the optimum conditions found consisted of performing the rearrangement of **1a** and **2a** in TBME (*c* 0.1 m) at 60 °C in the presence of **4f** (0.1 equiv) and 4 Å molecular sieves. Under these conditions, the desired rearranged product **3a** was isolated in 93% yield with 91% *ee*. Surprisingly, the product resulting from the competing *para*‐carbon‐alkylation of aniline **1a** with 2,3‐diketoester **2a** was not observed under these conditions.

**Table 1:** Survey of reaction conditions.

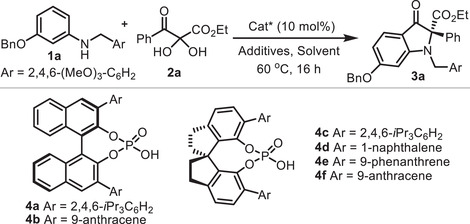					
Entry^a)^	Cat*	Solvent	Additives	Yield (%)^b)^	Ee (%)^c)^
1	**4a**	TBME	4 Å M.S.	79	76
2	**4b**	TBME	4 Å M.S.	41	71
3	**4c**	TBME	4 Å M.S.	80	72
4	**4d**	TBME	4 Å M.S.	83	80
5	**4e**	TBME	4 Å M.S.	87	86
6	**4f**	TBME	4 Å M.S.	93	91
7	**4f**	TBME	3 Å M.S.	87	90
8	**4f**	TBME	5 Å M.S.	77	90
9^d)^	**4f**	TBME	4 Å M.S.	75	90
10^e)^	**4f**	TBME	4 Å M.S.	89	90
11^f)^	**4f**	TBME	4 Å M.S.	93	89
12	**4f**	Toluene	4 Å M.S.	94	90
13	**4f**	CyH	4 Å M.S.	69	85
14	**4f**	DCE	4 Å M.S.	69	70

^a)^ Standard conditions: **1a** (0.05 mmol), **2a** (0.05 mmol), Cat* (0.1 equiv), M.S. (15 mg), solvent (*c* 0.1 M), 60 °C, sealed tube, 16 h;

^b)^ Isolated yields;

^c)^ Determined by HPLC analysis;

^d)^ 50 °C;

^e)^ 70 °C;

^f)^ 80 °C;

Abbreviations: M.S. = molecular sieves; TBME = methyl *tert*‐butyl ether, CyH = cyclohexane, DCE = 1,2‐dichloroethane.

With the optimized conditions in hand, the scope of this asymmetric cyclizative rearrangement was next examined (**Table** **2**). Regarding the aryl group of 3‐aryl substituted 2,3‐diketoesters, the presence of electron‐neutral (H, Ph), electron‐donating (MeO), and electron‐withdrawing (F, Br, CN, NO_2_, CF_3_, CO_2_Et) groups at the *para*‐position of the phenyl ring were all well tolerated (**3a**–**3i**). The absolute configuration of **3a** was determined to be (*R*) by X‐ray crystallography, and the configurations of the other indolin‐3‐ones were assigned accordingly.^[^ ^22^ ^]^ Performing the gram scale reactions of **1a** (2.0 mmol) and **2a** (2.0 mmol) in the presence of **4f** (0.1 equiv) gave the desired product **4a** in retentive yield and enantioselectivity (90% yield, 91% *ee*). Substituents at the *meta*‐position of the phenyl ring underwent the asymmetric rearrangement reaction smoothly (**3j**–**3k**). Decreased stereoselectivities were observed with aryl groups bearing *ortho‐*substituents (**3l–3m**) while the yields were also in high level. Moreover, 3,5‐dimethylphenyl, 3,4‐dichlorophenyl, benzo[*d*][1,3]dioxole, 1‐naphthyl, 2‐naphthyl, and even heterocycles (2‐thiophene and 3‐thiophene) were transferred without event (**3n**–**3t**). Aspirin and (*S*)‐(+)‐Ibuprofen derived tricarbonyl compounds furnished the corresponding 2,2‐disubstituted indolin‐3‐ones in excellent stereoselectivities (**3u**–**3v**). 2,3‐Diketoester bearing functionalizable alkenyl group was also tested and delivered the desired product **3w** in excellent enantiomeric excess. Remarkably, alkyl groups, such as cyclohexyl, propyl, and chloromethyl, gave the desired products **3x–3z** in excellent enantiopurities as well. Furthermore, 2,3‐diketoesters derived from various alcohols, such as methyl, isopropyl, *tert*‐butyl and 2‐(trimethylsilyl)ethyl, were all well‐accepted substrates (**3aa**–**3ad**). A scale‐up reaction of **1a** (3.0 mmol) and **2ac** (3.0 mmol) was also conducted and gave the desired product **3ac** (1.68 g) in 94% yield with 95% *ee*. Both benzil and 1‐phenylpropane‐1,2‐dione gave no reaction, which indicated the importance of ester moiety on substrate **2**.

**Table 2:** Substrate the scope of the 2,3‐diketoesters and anilines^a)^.

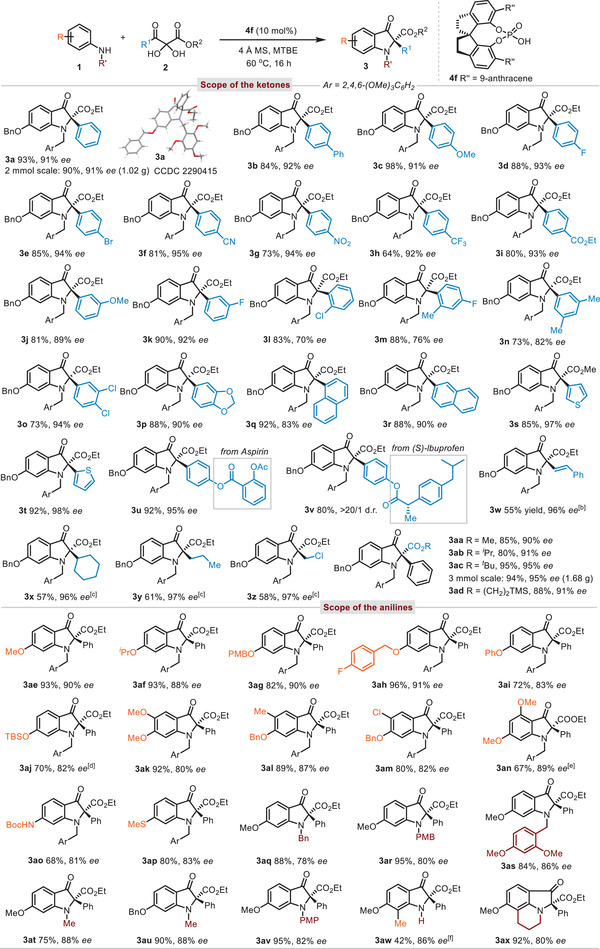

^a)^ **1** (0.05 mmol), **2** (0.05 mmol), **4f** (0.1 equiv), 4 Å M.S. (15 mg), TBME (*c* 0.1 m), 60 °C, sealed tube, 16 h;

^b)^ **1a** (0.05 mmol), **2** (0.0675 mmol), toluene (*c* 0.1 m), 40 °C;

^c)^ **1** (0.05 mmol), **2a** (0.06 mmol), 40 °C;

^d)^ 80 °C;

^e)^ 50 °C, 60 h;

^f)^ **1** (0.07 mmol), **2a** (0.05 mmol), 40 °C, 36 h.

The scope of the aniline derivatives **1** was then examined and representative examples were displayed in Table 2. The cyclizative rearrangement of anilines with different *meta*‐alkoxy residues, such as methyl, isopropyl, *p*‐methoxybenzyl (PMB), and *p*‐fluorobenzyl, proceeded smoothly to provide the rearranged products **3ae**–**3ah** in excellent yields and enantioselectivities. Moreover, aryloxyl as well as (*tert*‐butyldimethylsilyl)oxy groups were also tolerated albeit with slightly diminished enantioselectivities (**3ai–3aj**). Remarkably, additional substituents, such as methoxyl, methyl, and chloro groups, at different positions of the benzene ring delivered the desired products in high enantiomeric excesses (**3ak–3an**).

In principle, the *meta*‐electron donating substituents on anilines were capable of enhancing the nucleophilicity of the Csp^2^ at 6‐position. Indeed, other heteroatoms, such as nitrogen and sulfur, were able to play the same role in this reaction, providing the indolin‐3‐ones **3ao–3ap** with high enantiopurities. Alternatively, a series of *N*‐substituents, such as benzyl, *para*‐methoxybenzyl, 2,4‐dimethoxybenzyl, methyl, and *para*‐methoxyphenyl groups, were successfully engaged thus leading to structurally diverse products **3aq–3av** in high yields with remarkable enantiocontrol. Notably, primary amine was also an applicable candidate to give the desired product **3aw** in moderate yield with high enantiopurity. The lower yield of **3aw** was due to the relatively messy reaction. Furthermore, a tricyclic compound **3ax** could be also obtained in excellent yield with high enantiomeric excess when 5‐methoxy‐1,2,3,4‐tetrahydroquinoline was employed as a reaction partner. Notably, in all cases, no products originating from *para*‐Friedel‐Crafts addition of anilines **1** onto vicinal diketones **2** were observed.

**Scheme 2:**
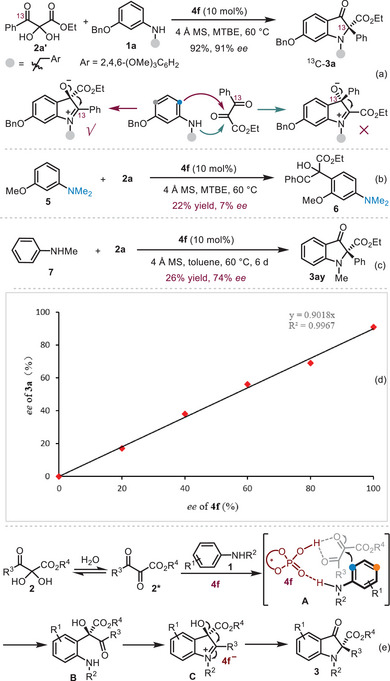
Control experiments.

To understand the possible reaction mechanism, a series of control experiments were performed (**Scheme** **2**). Initially, ^13^C‐labeled 2,3‐diketoester **2a’** was synthesized to undergo this asymmetric cyclizative 1,2‐rearrangement. Significantly, the isotopically labeled carbon exists as the aza‐quaternary stereocenter rather than the carbonyl moiety in the product ^13^C‐**3a** (see Supporting Information for details), which suggests that the reaction proceeds via a 1,2‐ester shift instead of a 1,2‐phenyl migration (Scheme 2a). Importantly, it also implies that this CACR might be initiated by the nucleophilic 1,2‐addition of C(sp^2^)‐H to the central carbonyl group of 2,3‐diketoester **2**. Interestingly, *N,N*‐dimethyl‐substituted aniline **5** only delivered the *para*‐carbon attack product **6** with a low level of yield and enantioselectivity (Scheme 2b), which indicated that the hydrogen bond between aniline derivative **1a** and the chiral catalyst **4f** is not only important for the stereoselectivity but also crucial for the regiocontrol. Interestingly, the simple *N*‐methylaniline **7** featured lower nucleophilicity could also undergo this cyclizative rearrangement under the standard reaction conditions, affording the desired product **3ay** with good enantioselectivity (74% *ee*, Scheme 2c). The low yield (26%) of **3ay** due to the low conversion of the substrates **7** and **2a**, and the product resulting from the *para*‐Friedel‐Crafts addition of aniline **7** to substrate **2a** was not observed as well. Other achiral strong Brønsted acids such as trifluoroacetic acid and *p*‐toluenesulfonic acid, gave no reaction. These results suggest again that the hydrogen bond between the amine moiety and the bifunctional catalyst **4f** is the key to the high regioselectivity rather than the orientation effect of the electron‐donating groups at the *meta*‐position of the anilines **1**. Finally, we performed a set of experiments with *ee*‐varied chiral phosphoric acid **4f**, aniline **1a**, and 2,3‐diketoester **2a** under the standard conditions (Scheme 2d). The results revealed the linear relationship between the *ee* value of **4f** and the *ee* value of the indolin‐3‐one **3a**, which suggested that a monomeric complex may work as a catalyst to promote the reaction.^[^ ^23^ ^]^ On the basis of the mechanistic experiments, a possible reaction pathway is proposed in Scheme 2e. Initially, under our conditions, substrate **2** could readily dehydrate to the vicinal tricarbonyl compound 2* where the central carbonyl group is highly nucleophilic. Subsequently, the CPA‐catalyzed regioselective and stereoselective catalytic *ortho*‐carbon‐addition of anilines **1** to 2,3‐diketoesters **2** occurs through a key intermediate **A**, where the *para*‐Csp^2^ is too far to reach the central carbonyl group of **2** while *ortho*‐Csp^2^ attack is favored, leading to the formation of the enantioenriched tertiary alcohol **B**. A crucial α‐hydroxyl iminium intermediate **C** was then generated from the intramolecular cyclizative condensation of **B** in the presence of **4f**. Finally, a stereospecific 1,2‐ester shift^[^ ^13^, ^14^ ^]^ afforded the desired product **3** with the observed stereocontrol.

**Scheme 3:**
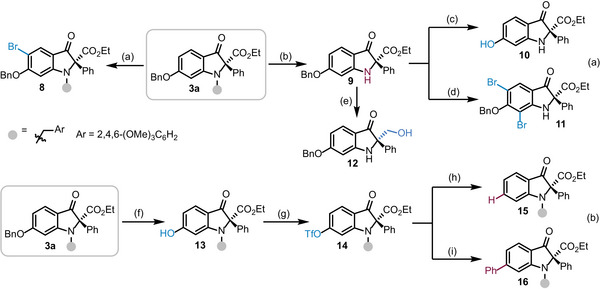
Representative product transformations. Conditions: a) NBS (1.2 equiv), DME, RT, 88% yield; b) DDQ (2.0 equiv), DCM/H_2_O, RT, 82% yield; c) Pd/C, H_2_, EtOAc, RT, 89% yield; d) NBS (2.8 equiv), DME, RT, 87% yield; e) BH_3_∙DMS (3.0 equiv), THF, 60 ^o^C, 10 h, 62% yield; f) Pd/C, H_2_, EtOAc, RT, 95% yield; g) Tf_2_O (2.0 equiv), DMAP (0.1 equiv), Et_3_N (4.0 equiv), DCM, 0 ^o^C, 3 h, 84% yield; h) Pd(OAc)_2_ (5 mol%), PPh_3_ (10 mol%), Et_3_N (3.0 equiv), HCOOH (2.0 equiv), DMF, 70 ^o^C, 12 h, 94% yield; i) Pd(PPh_3_)_2_Cl_2_ (10 mol%), Cs_2_CO_3_ (2.0 equiv), PhB(OH)_2_ (2.0 equiv), dioxane/H_2_O, 110 ^o^C, 3 h, 96% yield. Abbreviations: NBS = *N*‐bromosuccinimide; DME = Dimethoxyethane; DDQ = 2,3‐Dichloro‐5,6‐dicyano‐1,4‐benzoquinone; DCM = CH_2_Cl_2_; DMAP = 4‐Dimethylaminopyridine; DMF = Dimethylformamide.

Further transformations of the enantioenriched indolin‐3‐one **3a** were performed to illustrate the synthetic potential of our reaction (**Scheme** **3**). Mono‐bromination of **3a** in the presence of *N*‐bromosuccinimide (NBS, 1.2 equiv) delivered compound **8** with a high yield. Treatment of **3a** with excessive 2,3‐dichloro‐5,6‐dicyano‐1,4‐benzoquinone (DDQ) at room temperature afforded the *N*‐deprotection product **9** which could be further transformed to the *O*‐deprotection product **10** in high yield. On the other hand, treatment of **9** with 2.8 equiv of NBS afforded the dibrominated product **11** that was armed for further functionalization (Scheme 3a). In the presence of the excessive BH_3_∙DMS, the ester group of compound **9** could be easily reduced to the primary alcohol (**12**). Moreover, deprotection of **3a** gave the product **13** bearing a hydroxyl group which provided a versatile handle for the functionalization of the rearranged products. As depicted in Scheme 3b, triflation of compound **13** delivered an important building block **14** in 84% yield. Reduction of **14** under the catalysis of palladium complex combining with HCOOH as a reductant led to the formation of compound **15**. Finally, Pd(II)‐catalyzed Suzuki‐Miyaura cross coupling of **14** with phenyl boronic acid gave the desired product **16** in excellent yield.

## 3 Conclusion

In conclusion, we have developed a CACR of aniline derivatives and vicinal diketones for the efficient synthesis of indolin‐3‐ones bearing quaternary stereocenters. An unusual amine‐directed regio‐ and stereoselective *ortho*‐Csp^2^‐H addition of anilines to ketones is the key to the success of this CACR. Notably, aniline derivatives are employed as highly effective 1,3‐binuclephiles in the field of asymmetric catalysis for the first time. This asymmetric tandem transformation, catalyzed by a single chiral phosphoric acid, exhibits excellent efficiency, simple procedure, good functional group compatibility, and broad substrate scope. The synthetic potential was also illustrated by the postfunctionalization of the resulting products. The development of cyclizative rearrangements is a fascinating research field, and further research on this topic is underway in our laboratory.

## Conflict of Interest

The authors declare no conflict of interest.

## Supporting information

Supporting Information

Supporting Information

## Data Availability

The data that support the findings of this study are available in the supplementary material of this article.
