# Supplementary material for: Catalytic Asymmetric Cyclizative Rearrangement of Anilines and Vicinal Diketones to Access 2,2‐Disubstituted Indolin‐3‐ones
Source: Adv Sci (Weinh). 2024 Apr 24;11(25):2402532. doi: 10.1002/advs.202402532 (PMC11220653; doi:10.1002/advs.202402532)
Supplement: Supplementary file 1 — Supporting Information [file ADVS-11-2402532-s001.pdf]

ADVANCED SCIENCE
Open Access

Supporting Information

for *Adv. Sci.*, DOI 10.1002/advs.202402532

Catalytic Asymmetric Cyclizative Rearrangement of Anilines and Vicinal Diketones to Access 2,2-Disubstituted Indolin-3-ones

Rui Quan, Xing-Zi Li, Zi-Qi Wang, Yu-Ping He and Hua Wu**

Table of Contents

1. General information S2

2. Synthesis and characterization data of **1** S3

3. Survey of reaction conditions S9

4. General procedure for the synthesis of **3** S14

5. Characterization data of **3** S15

6. Control experiments S48

7. Gram scale reaction and synthetic transformations S54

8. References S64

9. Crystallographic data of **3a** S65

10. Copies of NMR spectra S79

11. Copies of HPLC chromatograms S159

1. General information

NMR spectra were recorded on Brucker AV 400 MHz spectrometer. Chemical shifts are given in ppm. The spectra are calibrated to the residual ^1H and ^{13}C signals of the solvents. Data for ^1H NMR are recorded as follows: chemical shift (δ, ppm), multiplicity (s = singlet, d = doublet, t = triplet, q = quartet, sept = septet, m = multiplet or unresolved, brs = broad singlet, dd = doublet-doublet, dt = doublet of triplets, dq = doublet of quartet, ddd = doublet of doublet of doublets, tt = triplet of triplet, qd = quartet of doublets), coupling constant (s) in Hz, integration. data for ^{13}C NMR and ^{19}F NMR are reported in terms of chemical shift (δ, ppm). The *ee* values were determined by HPLC (SHIMADZU LC-20AT) using a Daicel chiral column. Mass spectrometry analysis was carried out using an electrospray spectrometer Waters Micromass Q-TOF Premier Mass Spectrometer. Melting points were measured with SGW X-4 micro melting point apparatus. Optical rotations were measured on a Rudolph Research Analytical Autopol VI automatic polarimeter using a 50 mm path-length cell at 589 nm.

Materials and Methods: Unless otherwise stated, starting materials were purchased from commercial sources (Adamas-Beta®, Shanghai Haohong Scientific Co., Ltd., Shanghai Bide pharmatech Co., Ltd., J&K®, Meryer, Macklin, Aladdin® and Energy chemical), and used without further purification. The chiral phosphoric acids were purchased from Daicel Chiral Technologies (China) Co., Ltd. Sensitive compounds were stored in adesiccator or in a glove box if required. Solvents were purchased in HPLC quality, degassed by purging thoroughly with argon and dried over activated molecular sieves of appropriate size. Alternatively, they were purged with argon and passed through alumina columns in a solvent purification system (Innovative Technology). The $CDCl_3$ was supplied by Shanghai Haohong Scientific Co., Ltd. Reactions were monitored by thin layer chromatography (TLC) using Xinnuo TLC silica gel 60 F254. Compounds were visualized by UV-light at 254 nm and by dipping the plates in an ethanolic vanillin/sulfuric acid solution or an aqueous potassium permanganate solution followed by heating. Flash column chromatography was performed over silica gel (300-400 mesh). The $CDCl_3$ used in the NMR experiments was stored over anhydrous K_2CO_3 before used.

2. Synthesis and characterization data of 1

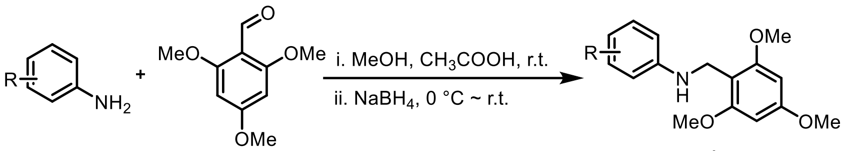

To a stirred solution of 2,4,6-trimethoxybenzaldehyde (3.0 mmol, 1.0 equiv) in MeOH (20 mL) was added aniline (3.0 mmol, 1.0 equiv), then a few drops of acetic acid was added. After stirred at room temperature for 2 hours, the solution was cooled to 0 °C and $NaBH_4$ (7.5 mmol, 2.5 equiv) was added slowly. Then the reaction mixture was stirred at room temperature for 12 h. After completion of reaction (monitored by TLC), the reaction was quenched by H_2O (20 mL) and extracted with ethyl acetate (20 mL × 3). The combined organic layers were washed with brine, dried over anhydrous Na_2SO_4, filtered and concentrated under reduced pressure. The crude residue was purified by flash chromatography on silica gel eluting with PE/EA = 10/1 ~ 5/1 to afford the compounds **1**.

3-(benzyloxy)-*N*-(2,4,6-trimethoxybenzyl)aniline (1a)

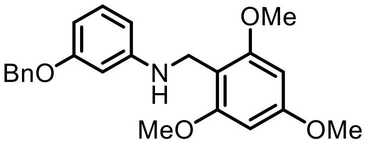

1.05 g, white solid, 92% yield, m.p. = 123 – 125 °C.

^{1}H NMR (400 MHz, Chloroform-*d*) δ 7.47 – 7.41 (m, 2H), 7.41 – 7.35 (m, 2H), 7.35 – 7.29 (m, 1H), 7.05 (t, *J* = 8.1 Hz, 1H), 6.45 (t, *J* = 2.3 Hz, 1H), 6.34 (ddd, *J* = 17.1, 8.1, 1.9 Hz, 2H), 6.14 (s, 2H), 5.03 (s, 2H), 4.28 (s, 2H), 4.11 (brs, 1H), 3.83 (s, 6H), 3.81 (s, 3H).

^{13}C NMR (101 MHz, Chloroform-*d*) δ 160.8, 160.2, 159.5, 150.5, 137.7, 129.8, 128.6, 127.9, 127.6, 108.2, 107.1, 103.4, 100.2, 90.7, 69.9, 55.9, 55.5, 36.7.

HRMS (ESI) m/z: $[M + H]^+$ Calcd for $C_{23}H_{26}NO_4^+$ 380.1856; Found 380.1849.

3-methoxy-*N*-(2,4,6-trimethoxybenzyl)aniline (1ae)

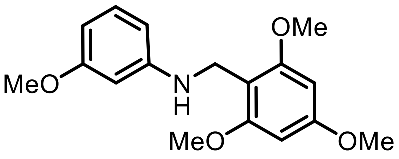

801 mg, white solid, 88% yield, m.p. = 121 – 123 °C.

^{1}H NMR (400 MHz, Chloroform-*d*) ^{1}H NMR (400 MHz, Chloroform-*d*) δ 7.08 – 7.01 (m, 1H), 6.36 – 6.32 (m, 2H), 6.26 – 6.22 (m, 1H), 6.13 (s, 2H), 4.28 (s, 2H), 4.10 (brs, 1H), 3.83 (s, 6H), 3.81 (s, 3H), 3.77 (s, 3H).

^{13}C NMR (101 MHz, Chloroform-*d*) δ 160.9, 160.8, 159.5, 150.4, 129.8, 108.2, 106.8, 102.6, 99.2, 90.7, 55.9, 55.5, 55.2, 36.6.

HRMS (ESI) m/z: $[M + H]^+$ Calcd for $C_{17}H_{22}NO_4^+$ 304.1543; Found 304.1544.

3-isopropoxy-*N*-(2,4,6-trimethoxybenzyl)aniline (1af)

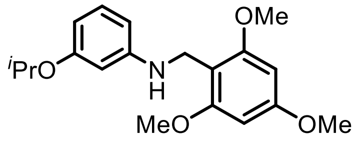

815 mg, white solid, 82% yield, m.p. = 87 – 88 °C.

^{1}H NMR (400 MHz, Chloroform-*d*) δ 7.02 (t, *J* = 8.0 Hz, 1H), 6.35 – 6.30 (m, 2H), 6.23 (ddd, *J* = 8.1, 2.4, 0.9 Hz, 1H), 6.13 (s, 2H), 4.51 (p, *J* = 6.1 Hz, 1H), 4.26 (s, 2H), 4.06 (s, 1H), 3.82 (s, 6H), 3.81 (s, 3H), 1.32 (d, *J* = 6.0 Hz, 6H).

^{13}C NMR (101 MHz, Chloroform-*d*) δ 160.8, 159.5, 159.2, 150.5, 129.7, 108.3, 106.7, 104.6, 101.3, 90.8, 69.6, 55.9, 55.5, 36.7, 22.4.

HRMS (ESI) m/z: $[M + H]^+$ Calcd for $C_{19}H_{26}NO_4^+$ 332.1856; Found 332.1860.

3-((4-methoxybenzyl)oxy)-*N*-(2,4,6-trimethoxybenzyl)aniline (1ag)

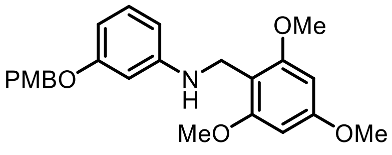

1.18 g, white solid, 96% yield, m.p. = 137 – 139 °C.

^{1}H NMR (400 MHz, Chloroform-*d*) δ 7.39 – 7.33 (m, 2H), 7.05 (t, *J* = 8.1 Hz, 1H), 6.94 – 6.89 (m, 2H), 6.44 (t, *J* = 2.3 Hz, 1H), 6.38 – 6.29 (m, 2H), 6.14 (s, 2H), 4.96 (s, 2H), 4.28 (s, 2H), 4.11 (brs, 1H), 3.83 (s, 6H), 3.82 (s, 3H), 3.81 (s, 3H).

^{13}C NMR (101 MHz, Chloroform-*d*) δ 160.8, 160.2, 159.49, 159.47, 150.5, 129.74,

129.71, 129.3, 114.1, 108.2, 107.0, 103.5, 100.2, 90.7, 69.6, 55.9, 55.5, 55.4, 36.7.

HRMS (ESI) m/z: $[M + Na]^+$ Calcd for $C_{24}H_{27}NNaO_5^+$ 432.1781; Found 432.1788.

3-((4-fluorobenzyl)oxy)-*N*-(2,4,6-trimethoxybenzyl)aniline (1ah)

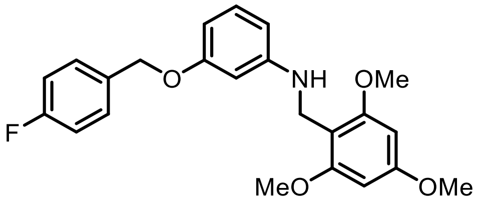

1.07 g, white solid, 90% yield, m.p. = 130 – 132 °C.

^{1}H NMR (400 MHz, Chloroform-*d*) δ 7.43 – 7.37 (m, 2H), 7.10 – 7.02 (m, 3H), 6.42 (t, J = 2.3 Hz, 1H), 6.36 (dd, J = 8.1, 2.2 Hz, 1H), 6.29 (dd, J = 8.1, 2.4 Hz, 1H), 6.13 (s, 2H), 4.98 (s, 2H), 4.27 (s, 2H), 4.11 (s, 1H), 3.82 (s, 6H), 3.81 (s, 3H).

^{13}C NMR (101 MHz, Chloroform-*d*) δ 162.56 (d, J = 245.6 Hz), 160.41 (d, J = 84.4 Hz), 150.5, 133.44, 133.40, 129.8, 129.43 (d, J = 8.1 Hz), 115.51 (d, J = 21.2 Hz), 108.1, 107.2, 103.4, 100.1, 90.7, 69.2, 55.9, 55.5, 36.7.

^{19}F NMR (376 MHz, Chloroform-*d*) δ -114.7.

HRMS (ESI) m/z: $[M + Na]^+$ Calcd for $C_{23}H_{24}FNNaO_4^+$ 420.1582; Found 420.1583.

3-phenoxy-*N*-(2,4,6-trimethoxybenzyl)aniline (1ai)

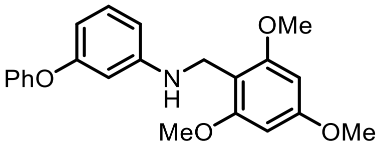

899 mg, white solid, 82% yield, m.p. = 105 – 107 °C.

^{1}H NMR (400 MHz, Chloroform-*d*) δ 7.34 – 7.27 (m, 2H), 7.11 – 7.03 (m, 2H), 7.02 – 6.98 (m, 2H), 6.50 – 6.45 (m, 2H), 6.33 – 6.28 (m, 1H), 6.11 (s, 2H), 4.25 (s, 2H), 4.18 (brs, 1H), 3.81 (s, 3H), 3.77 (s, 6H).

^{13}C NMR (101 MHz, Chloroform-*d*) δ 160.8, 159.4, 158.1, 157.9, 150.6, 130.0, 129.6, 122.7, 118.7, 109.2, 108.0, 107.8, 104.2, 90.7, 55.8, 55.5, 36.6.

HRMS (ESI) m/z: $[M + Na]^+$ Calcd for $C_{22}H_{23}NNaO_4^+$ 388.1519; Found 388.1524.

3-((*tert*-butyldimethylsilyl)oxy)-*N*-(2,4,6-trimethoxybenzyl)aniline (1aj)

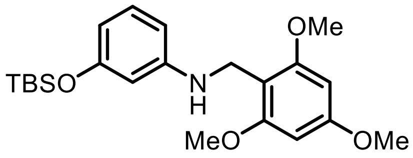

944 mg, colorless oil, 78% yield.

^{1}H NMR (400 MHz, Chloroform-*d*) δ 6.98 (t, *J* = 8.0 Hz, 1H), 6.34 (ddd, *J* = 8.1, 2.3, 0.9 Hz, 1H), 6.29 (t, *J* = 2.3 Hz, 1H), 6.17 (ddd, *J* = 8.0, 2.3, 0.9 Hz, 1H), 6.13 (s, 2H), 4.26 (s, 2H), 3.83 (s, 6H), 3.81 (s, 3H), 0.99 (s, 9H), 0.20 (s, 6H).

^{13}C NMR (101 MHz, Chloroform-*d*) δ 160.7, 159.5, 156.7, 150.5, 129.5, 109.0, 108.2, 107.4, 105.4, 90.7, 55.9, 55.5, 36.7, 25.9, 18.3, -4.3.

HRMS (ESI) m/z: $[M + H]^+$ Calcd for $C_{22}H_{34}NO_4Si^+$ 404.2252; Found 404.2261.

3,4-dimethoxy-*N*-(2,4,6-trimethoxybenzyl)aniline (1ak)

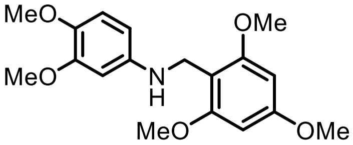

940 mg, white solid, 94% yield, m.p. = 135 – 137 °C.

^{1}H NMR (400 MHz, Chloroform-*d*) δ 6.72 (d, *J* = 8.6 Hz, 1H), 6.41 (d, *J* = 2.6 Hz, 1H), 6.28 (dd, *J* = 8.5, 2.6 Hz, 1H), 6.13 (s, 2H), 4.25 (s, 2H), 3.93 (brs, 1H), 3.84 (s, 3H), 3.83 (s, 6H), 3.80 (s, 3H), 3.79 (s, 3H).

^{13}C NMR (101 MHz, Chloroform-*d*) δ 160.7, 159.5, 145.0, 144.0, 141.5, 113.3, 108.3, 104.7, 99.5, 90.7, 56.9, 55.9, 55.8, 55.5, 37.5.

HRMS (ESI) m/z: $[M + Na]^+$ Calcd for $C_{18}H_{23}NNaO_5^+$ 356.1468; Found 356.1473.

3-(benzyloxy)-4-methyl-*N*-(2,4,6-trimethoxybenzyl)aniline (1al)

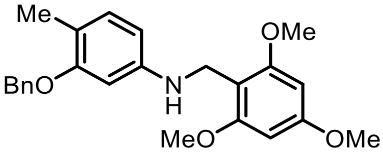

1.03 g, white solid, 87% yield, m.p. = 94 – 96 °C.

^{1}H NMR (400 MHz, Chloroform-*d*) δ 7.50 – 7.45 (m, 2H), 7.43 – 7.37 (m, 2H), 7.36 – 7.30 (m, 1H), 6.95 (d, *J* = 8.0 Hz, 1H), 6.44 (d, *J* = 2.2 Hz, 1H), 6.32 (dd, *J* = 7.9, 2.2 Hz, 1H), 6.14 (s, 2H), 5.06 (s, 2H), 4.30 (s, 2H), 4.06 (s, 1H), 3.84 (s, 6H), 3.81 (s, 3H), 2.18 (s, 3H).

^{13}C NMR (101 MHz, Chloroform-*d*) δ 160.7, 159.5, 157.7, 148.5, 138.0, 130.9, 128.6, 127.7, 127.3, 115.6, 108.4, 105.9, 98.5, 90.7, 69.8, 55.9, 55.4, 37.0, 15.6.

HRMS (ESI) m/z: $[M + Na]^+$ Calcd for $C_{24}H_{27}NNaO_4^+$ 416.1832; Found 416.1843.

3-(benzyloxy)-4-chloro-*N*-(2,4,6-trimethoxybenzyl)aniline (1am)

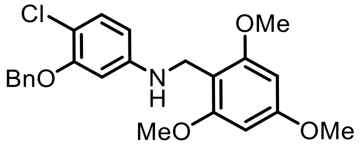

1.13 g, white solid, 91% yield, m.p. = 98 – 100 °C.

^{1}H NMR (400 MHz, Chloroform-*d*) δ 7.48 (d, *J* = 7.3 Hz, 2H), 7.38 (t, *J* = 7.5 Hz, 2H), 7.34 – 7.29 (m, 1H), 7.09 (d, *J* = 8.6 Hz, 1H), 6.45 (d, *J* = 2.5 Hz, 1H), 6.28 (dd, *J* = 8.6, 2.5 Hz, 1H), 6.13 (s, 2H), 5.10 (s, 2H), 4.25 (s, 2H), 4.16 (brs, 1H), 3.83 (s, 6H), 3.80 (s, 3H).

^{13}C NMR (101 MHz, Chloroform-*d*) δ 160.9, 159.4, 154.9, 148.9, 137.1, 130.3, 128.7, 128.0, 127.3, 110.8, 107.8, 107.1, 100.0, 90.8, 70.8, 55.9, 55.5, 36.8.

HRMS (ESI) m/z: $[M + Na]^+$ Calcd for $C_{23}H_{24}ClNNaO_4^+$ 436.1286; Found 436.1295.

3,5-dimethoxy-*N*-(2,4,6-trimethoxybenzyl)aniline (1an)

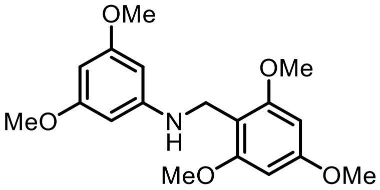

810 mg, yellow solid, 81% yield, m.p. = 95 – 97 °C.

^{1}H NMR (400 MHz, Chloroform-*d*) δ 6.13 (s, 2H), 5.96 (d, *J* = 2.2 Hz, 2H), 5.85 (t, *J* = 2.2 Hz, 1H), 4.27 (s, 2H), 4.12 (brs, 1H), 3.83 (s, 6H), 3.80 (s, 3H), 3.75 (s, 6H).

^{13}C NMR (101 MHz, Chloroform-*d*) δ 161.7, 160.8, 159.4, 150.9, 108.1, 92.1, 90.7, 89.9, 55.8, 55.4, 55.2, 36.6.

HRMS (ESI) m/z: $[M + H]^+$ Calcd for $C_{18}H_{24}NO_5^+$ 334.1649; Found 334.1655.

Tert-butyl (3-((2,4,6-trimethoxybenzyl)amino)phenyl)carbamate (1ao)

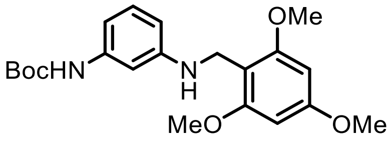

991 mg, pale yellow solid, 85% yield, m.p. = 104 – 106 °C.

^{1}H NMR (400 MHz, Chloroform-*d*) δ 7.04 (t, *J* = 8.0 Hz, 1H), 6.89 (s, 1H), 6.53 (dd, *J* = 7.9, 2.0 Hz, 1H), 6.44 (dd, *J* = 8.1, 2.2 Hz, 1H), 6.38 (s, 1H), 6.12 (s, 2H), 4.26 (s, 2H), 4.09 (brs, 1H), 3.82 (s, 6H), 3.80 (s, 3H), 1.51 (s, 9H).

^{13}C NMR (101 MHz, Chloroform-*d*) δ 160.8, 159.5, 152.8, 149.9, 139.2, 129.5, 108.3, 108.1, 107.5, 103.7, 90.6, 80.2, 55.8, 55.4, 36.6, 28.5.

HRMS (ESI) m/z: $[M + Na]^+$ Calcd for $C_{21}H_{28}N_2NaO_5^+$ 411.1890; Found 411.1897.

3-(methylthio)-N-(2,4,6-trimethoxybenzyl)aniline (1ap)

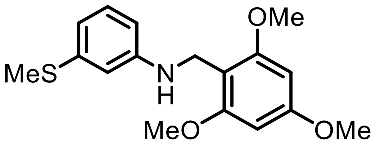

738 mg, yellow foam, 77% yield.

^{1}H NMR (400 MHz, Chloroform-*d*) δ 7.06 (t, *J* = 7.9 Hz, 1H), 6.69 (t, *J* = 2.0 Hz, 1H), 6.57 (m, 1H), 6.51 (dd, *J* = 8.1, 2.3 Hz, 1H), 6.13 (s, 2H), 4.28 (s, 2H), 4.13 (brs, 1H), 3.84 (s, 6H), 3.80 (s, 3H), 2.45 (s, 3H).

^{13}C NMR (101 MHz, Chloroform-*d*) δ 160.8, 159.4, 149.3, 138.9, 129.4, 115.4, 111.4, 111.0, 108.0, 90.7, 55.9, 55.5, 36.5, 15.9.

HRMS (ESI) m/z: $[M + Na]^+$ Calcd for $C_{21}H_{28}N_2NaO_5^+$ 342.1134; Found 342.1141.

3. Survey of reaction conditions

Table S1. Effect of Chiral phosphoric acid[a]

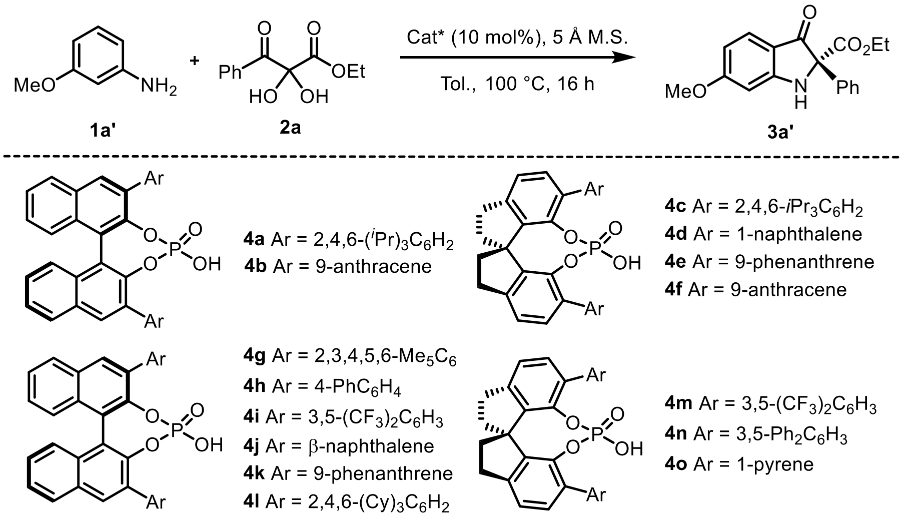

Entry	Cat.*	Yield [%][b]	*Ee* [%][c]
1	**4a**	72	0
2	**4b**	23	26
3	**4c**	10	40
4	**4d**	13	34
5	**4e**	10	43
6	**4f**	27	40
7	**4g**	27	21
8	**4h**	8	6
9	**4i**	11	4
10	**4j**	20	10
11	**4k**	14	34
12	**4l**	41	7
13	**4m**	14	20
14	**4n**	21	12
15	**4o**	17	37

[a] Standard conditions: **1a'** (0.05 mmol), **2a** (0.06 mmol), Cat* (0.005 mmol), 5 Å M.S. (15 mg), Tol. (*c* 0.1 M), sealed tube, 100 °C, 16 h. Abbreviations: Cy = cyclohexyl, M.S. = molecular sieves. [b] Isolated yields. [c] Determined by HPLC analysis.

Table S2. Effect of molecular sieves[a]

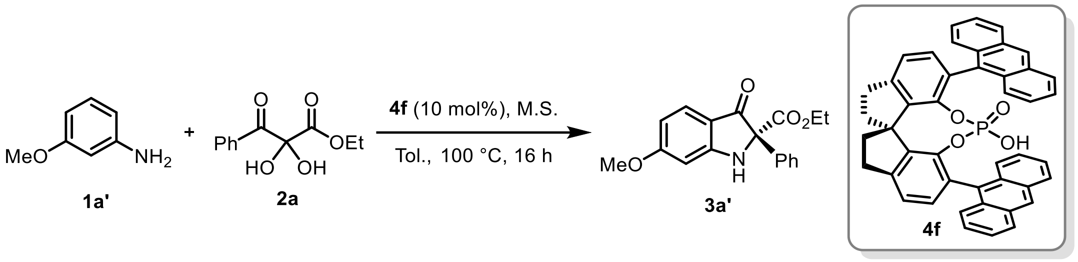

Entry	M.S.	Yield [%][b]	*Ee* [%][c]
1	3 Å	14	61
2	4 Å	11	69
3	5 Å	27	40

[a] Standard conditions: **1a'** (0.05 mmol), **2a** (0.06 mmol), **4f** (0.005 mmol), M.S. (15 mg), Tol. (*c* 0.1 M), sealed tube, 100 °C, 16 h. Abbreviations: M.S. = molecular sieves. [b] Isolated yields. [c] Determined by HPLC analysis.

Table S3. Effect of temperature[a]

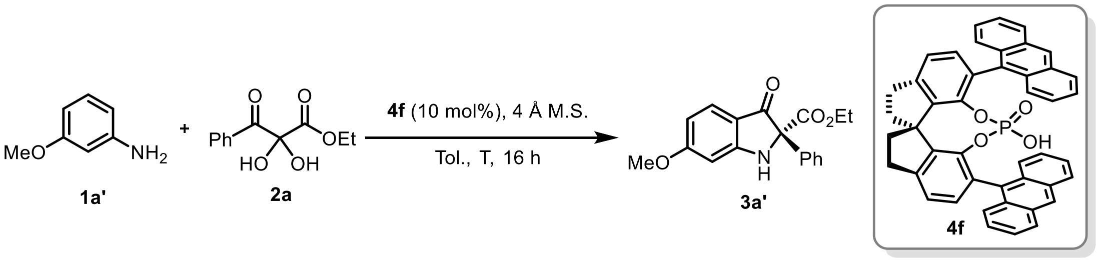

Entry	T [°C]	Yield [%][b]	*Ee* [%][c]
1	80	23	80
2	70	13	91
3	60	trace	-
4	50	trace	-

[a] Standard conditions: **1a'** (0.05 mmol), **2a** (0.06 mmol), **4f** (0.005 mmol), 4 Å M.S. (15 mg), Tol. (*c* 0.1 M), sealed tube, 16 h. Abbreviations: M.S. = molecular sieves. [b] Isolated yields. [c] Determined by HPLC analysis.

Table S4. Effect of solvent[a]

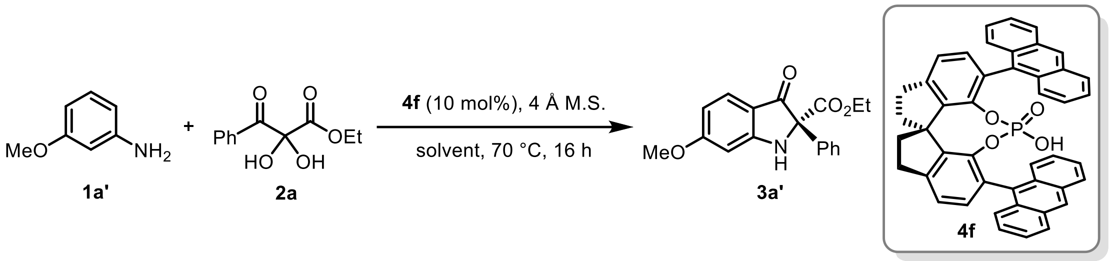

Entry	Solvent	Yield [%][b]	*Ee* [%][c]
1	CyH	80	44
2	DCE	13	45
3	PhF	22	70
4	CCl_4	30	53
5	TBME	18	91
6	DME	14	38

[a] Standard conditions: **1a'** (0.05 mmol), **2a** (0.06 mmol), **4f** (0.005 mmol), 4 Å M.S. (15 mg), solvent (*c* 0.1 M), sealed tube, 70 °C, 16 h. Abbreviations: M.S. = molecular sieves. [b] Isolated yields. [c] Determined by HPLC analysis.

Table S5. Effect of additives[a]

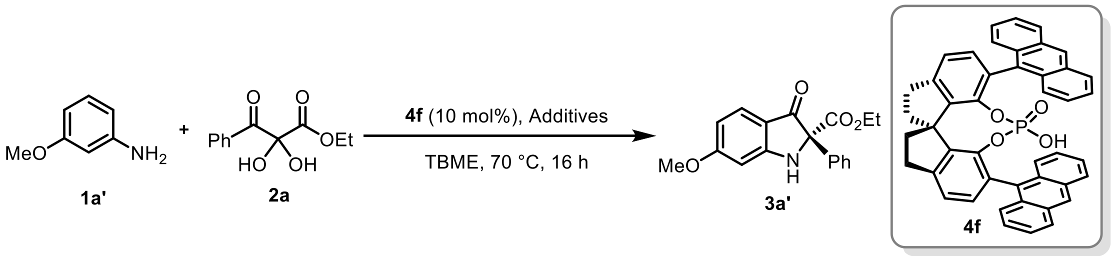

Entry	Additives	Yield [%][b]	*Ee* [%][c]
1	K_2SO_4 (50 mg)	37	45
2	Na_2SO_4 (50 mg)	18	67
3	4 Å M.S. (15 mg) + $Zn(OTf)_2$ (10 mol%)	12	22
4	4 Å M.S. (15 mg) + $Mg(OTf)_2$ (10 mol%)	25	24
5	4 Å M.S. (15 mg) + $Sc(OTf)_3$ (10 mol%)	17	34

[a] Standard conditions: **1a'** (0.05 mmol), **2a** (0.06 mmol), **4f** (0.005 mmol), TBME (*c* 0.1 M), sealed tube, 70 °C, 16 h. Abbreviations: M.S. = molecular sieves; [b] Isolated yields. [c] Determined by HPLC analysis.

Table S6. Effect of aniline derivatives substrates and other conditions[a]

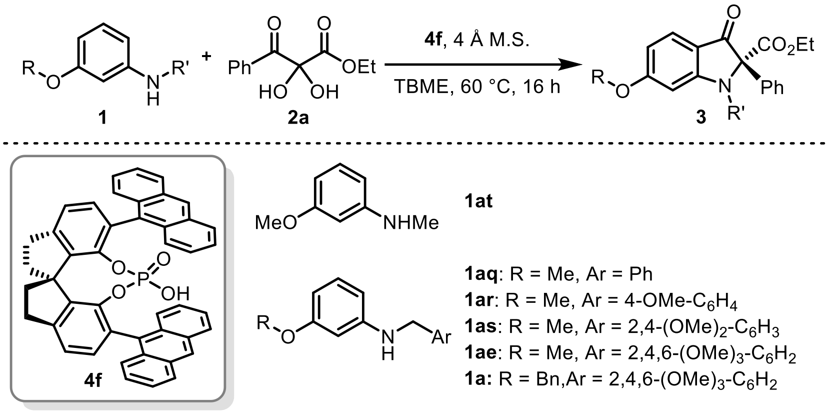

Entry	Substrate **1**	Yield [%][b]	*Ee* [%][c]
1[d]	**1at**	59	88
2	**1at**	75	89
3[e]	**1at**	27	89
4[f]	**1at**	59	88
5[g]	**1at**	75	89
6[h]	**1at**	46	84
7[d]	**1aq**	88	78
8	**1aq**	95	78
9	**1ar**	95	80
10	**1as**	84	85
11	**1ae**	88	90
12[g]	**1ae**	93	90
13[e][g]	**1ae**	81	91
14[e][g][i]	**1ae**	77	90
15[e][g][j]	**1ae**	74	90
16[e][g][k]	**1ae**	57	88
17[l]	**1ae**	70	88
18[e]	**1ae**	71	90
19[m]	**1ae**	70	90
20[g]	**1a**	93	91

[a] Standard conditions: **1** (0.05 mmol), **2a** (0.06 mmol), **4f** (0.005 mmol), 4 Å M.S. (15 mg), TBME (*c* 0.1 M), sealed tube, 60 °C. Abbreviations: M.S. = molecular sieves. [b] Isolated yields. [c] Determined by HPLC analysis. [d] 70 °C. [e] 50 °C. [f] TBME (*c* 0.05 M). [g] **2** (0.05 mmol). [h] **2** (0.075 mmol). [i] Na_2SO_4 replace 4 Å M.S. [j] $MgSO_4$ replace 4 Å M.S. [k] K_2SO_4 replace 4 Å M.S. [l] 4 Å M.S. (25 mg). [m] **4f** (0.0025 mmol).

4. General procedure for the synthesis of 3

General procedure for the synthesis of racemic 3

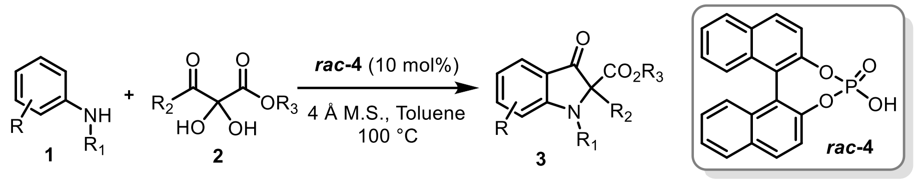

A suspension of **1** (0.1 mmol, 1.0 equiv), **2** (0.1 mmol, 1.0 equiv), ***rac*-4** (0.01 mmol, 0.1 equiv), 4 Å molecular sieves (30 mg) in toluene (1.0 mL) was stirred in a dry sealed tube. The reaction mixture was stirred at 100 °C for 16 h. After completion of the reaction (monitored by TLC), the solvent was removed under vacuum. The residue was purified by column chromatography on silica gel eluting with petroleum ether/ethyl acetate to afford the racemic products **3** in good to high yields.

Note: The tri-carbonyl compounds **2** were easily prepared, and the characterization data for these compounds have been shown according to the literature.[1-7] The ratio between hydrate form and tricarbonyl form was detected by **^{1}H NMR**, and the molecular weight was calculated accordingly.

General procedure for the enantioselective synthesis of 3

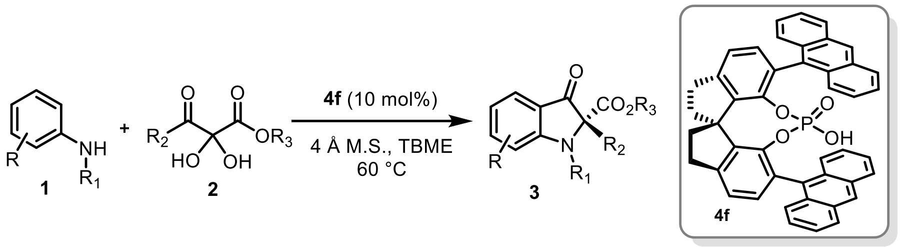

A suspension of **1** (0.05 mmol, 1.0 equiv), **2** (0.05 mmol, 1.0 equiv), **4f** (0.005 mmol, 0.1 equiv), 4 Å molecular sieves (15 mg) in TBME (0.5 mL) was stirred in a dry sealed tube. The reaction mixture was stirred at 60 °C for 16 h or for the indicated time. After completion of the reaction (monitored by TLC), the solvent was removed under vacuum. The residue was purified by column chromatography on silica gel eluting with petroleum ether/ethyl acetate to afford the products **3** in good to high yields.

5. Synthesis and characterization data of 3

Ethyl (*R*)-6-(benzyloxy)-3-oxo-2-phenyl-1-(2,4,6-trimethoxybenzyl)indoline-2-carboxylate (3a)

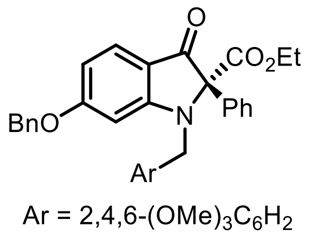

60 °C, 16 h. 19.6 mg, yellow solid, 93% yield, 91% *ee*, m.p. = 111 – 113 °C.

^{1}H NMR (400 MHz, Chloroform-*d*) δ 7.51 – 7.36 (m, 8H), 7.22 – 7.16 (m, 3H), 6.91 (d, *J* = 2.0 Hz, 1H), 6.42 (dd, *J* = 8.6, 2.0 Hz, 1H), 5.95 (s, 2H), 5.18 (s, 2H), 4.67 (d, *J* = 14.9 Hz, 1H), 4.61 (d, *J* = 14.9 Hz, 1H), 4.08 (dq, *J* = 10.7, 7.1 Hz, 1H), 3.89 (dq, *J* = 10.7, 7.1 Hz, 1H), 3.75 (s, 3H), 3.57 (s, 6H), 1.11 (t, *J* = 7.1 Hz, 3H).

^{13}C NMR (101 MHz, Chloroform-*d*) δ 193.3, 167.3, 167.1, 165.3, 161.3, 159.7, 136.3, 134.4, 128.8, 128.4, 127.8, 127.70, 127.68, 127.6, 127.4, 111.3, 107.8, 105.6, 94.0, 90.1, 80.2, 70.3, 61.6, 55.4, 55.3, 37.0, 14.0.

HRMS (ESI) m/z: $[M + H]^+$ Calcd for $C_{34}H_{34}NO_7^+$ 568.2330; Found 568.2329.

$[\alpha]_D^{23}$ = -157.9 (*c* 2.0, $CHCl_3$).

Chiral HPLC: Chiralpak AD-H, hexane:*i*PrOH = 70:30, 1.0 mL/min, 254 nm; tR = 7.6 min (minor), 9.4 min (major).

Ethyl (*R*)-2-([1,1'-biphenyl]-4-yl)-6-(benzyloxy)-3-oxo-1-(2,4,6-trimethoxybenzyl)indoline-2-carboxylate (3b)

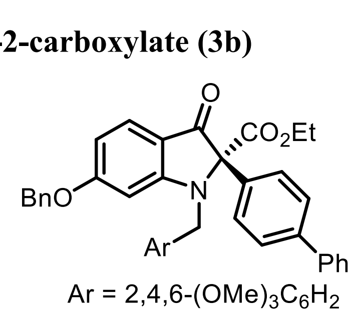

60 °C, 16 h. 27.1 mg, yellow oil, 84% yield, 92% *ee*.

^{1}H NMR (400 MHz, Chloroform-*d*) δ 7.55 – 7.28 (m, 15H), 6.91 (d, *J* = 2.0 Hz, 1H), 6.43 (dd, *J* = 8.6, 2.0 Hz, 1H), 5.92 (s, 2H), 5.19 (s, 2H), 4.70 (d, *J* = 14.9 Hz, 1H), 4.64 (d, *J* = 14.9 Hz, 1H), 4.13 (dq, *J* = 10.7, 7.1 Hz, 1H), 3.96 (dq, *J* = 10.8, 7.1 Hz, 1H),

3.70 (s, 3H), 3.58 (s, 6H), 1.15 (t, *J* = 7.1 Hz, 3H).

^{13}C NMR (101 MHz, Chloroform-*d*) δ 193.3, 167.4, 167.0, 165.3, 161.4, 159.6, 141.1, 140.4, 136.3, 133.7, 128.9, 128.8, 128.5, 128.1, 127.7, 127.5, 127.3, 127.2, 126.4, 111.2, 107.9, 105.6, 94.0, 90.0, 70.4, 61.7, 55.4, 55.3, 37.0, 14.1.

HRMS (ESI) m/z: $[M + H]^+$ Calcd for $C_{40}H_{38}NO_7^+$ 644.2643; Found 644.2644.

$[\alpha]_D^{23}$ = -224.8 (*c* 1.0, $CHCl_3$).

Chiral HPLC: Chiralpak AD-H, hexane:*i*PrOH = 70:30, 1.0 mL/min, 254 nm; tR = 11.8 min (minor), 13.8 min (major).

Ethyl (*R*)-6-(benzyloxy)-2-(4-methoxyphenyl)-3-oxo-1-(2,4,6-trimethoxybenzyl)indoline-2-carboxylate (3c)

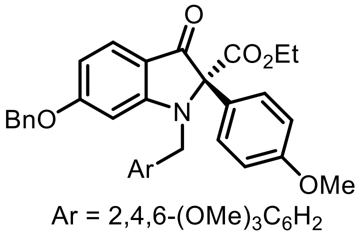

60 °C, 16 h. 29.3 mg, yellow oil, 98% yield, 91% *ee.*

^{1}H NMR (400 MHz, Chloroform-*d*) δ 7.51 – 7.34 (m, 8H), 6.87 (d, *J* = 2.1 Hz, 1H), 6.75 – 6.69 (m, 2H), 6.40 (dd, *J* = 8.6, 2.0 Hz, 1H), 5.95 (s, 2H), 5.17 (s, 2H), 4.63 (d, *J* = 14.9 Hz, 1H), 4.58 (d, *J* = 14.9 Hz, 1H), 4.06 (dq, *J* = 10.7, 7.1 Hz, 1H), 3.86 (dq, *J* = 10.8, 7.2 Hz, 1H), 3.75 (s, 3H), 3.75 (s, 3H), 3.59 (s, 6H), 1.09 (t, *J* = 7.1 Hz, 3H).

^{13}C NMR (101 MHz, Chloroform-*d*) δ 193.7, 167.34, 167.33, 165.2, 161.3, 159.7, 159.1, 136.4, 129.1, 128.9, 128.4, 127.7, 127.4, 126.6, 113.3, 111.3, 107.7, 105.7, 93.9, 90.1, 70.4, 61.6, 55.5, 55.4, 55.3, 36.9, 14.0.

HRMS (ESI) m/z: $[M + H]^+$ Calcd for $C_{35}H_{36}NO_8^+$ 598.2435; Found 598.2435.

$[\alpha]_D^{23}$ = -188.9 (*c* 1.0, $CHCl_3$).

Chiral HPLC: Chiralpak AD-H, hexane:*i*PrOH = 70:30, 1.0 mL/min, 254 nm; tR = 11.0 min (minor), 14.7 min (major).

Ethyl (*R*)-6-(benzyloxy)-2-(4-fluorophenyl)-3-oxo-1-(2,4,6-trimethoxybenzyl)indoline-2-carboxylate (3d)

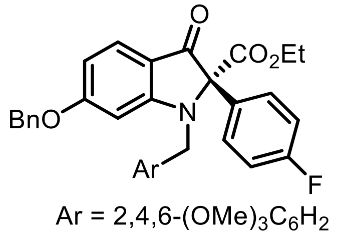

60 °C, 16 h. 25.7 mg, yellow oil, 88% yield, 93% *ee.*

^{1}H NMR (400 MHz, Chloroform-*d*) δ 7.52 – 7.36 (m, 8H), 6.91 (d, *J* = 2.0 Hz, 1H), 6.87 – 6.79 (m, 2H), 6.43 (dd, *J* = 8.6, 2.0 Hz, 1H), 5.93 (s, 2H), 5.19 (s, 2H), 4.65 (d, *J* = 14.9 Hz, 1H), 4.58 (d, *J* = 14.9 Hz, 1H), 4.09 (dq, *J* = 10.7, 7.1 Hz, 1H), 3.93 (dq, *J* = 10.8, 7.1 Hz, 1H), 3.75 (s, 3H), 3.57 (s, 6H), 1.12 (t, *J* = 7.1 Hz, 3H).

^{13}C NMR (101 MHz, Chloroform-*d*) δ 193.2, 167.5, 167.0, 165.4, 162.4 (d, *J* = 245.9 Hz), 161.5, 159.6, 136.3, 130.3 (d, *J* = 3.5 Hz), 129.5 (d, *J* = 8.1 Hz), 128.9, 128.5, 127.7, 127.5, 114.4 (d, *J* = 21.2 Hz), 111.1, 108.0, 105.4, 94.1, 90.1, 70.4, 61.8, 55.5, 55.3, 37.1, 14.1.

^{19}F NMR (377 MHz, Chloroform-*d*) δ -115.42.

HRMS (ESI) m/z: $[M + Na]^+$ Calcd for $C_{34}H_{32}FNNaO_7^+$ 608.2055; Found 608.2055.

$[\alpha]_D^{23}$ = -177.7 (*c* 1.0, $CHCl_3$).

Chiral HPLC: Chiralpak AD-H, hexane:*i*PrOH = 85:15, 1.0 mL/min, 254 nm; tR = 15.7 min (minor), 17.7 min (major).

Ethyl (*R*)-6-(benzyloxy)-2-(4-bromophenyl)-3-oxo-1-(2,4,6-trimethoxybenzyl)indoline-2-carboxylate (3e)

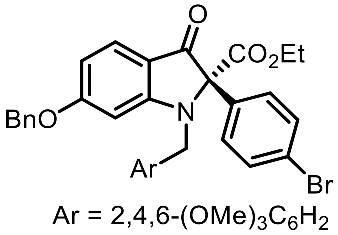

60 °C, 16 h. 27.3 mg, yellow oil, 85% yield, 94% *ee.*

^{1}H NMR (400 MHz, Chloroform-*d*) δ 7.43 – 7.35 (m, 5H), 7.34 – 7.29 (m, 1H), 7.28 – 7.24 (m, 2H), 7.20 – 7.16 (m, 2H), 6.83 (d, *J* = 2.1 Hz, 1H), 6.36 (dd, *J* = 8.6, 2.0 Hz, 1H), 5.85 (s, 2H), 5.12 (s, 2H), 4.58 (d, *J* = 14.9 Hz, 1H), 4.51 (d, *J* = 14.9 Hz, 1H),

4.02 (dq, J = 10.7, 7.1 Hz, 1H), 3.88 (dq, J = 10.8, 7.1 Hz, 1H), 3.69 (s, 3H), 3.49 (s, 6H), 1.06 (t, J = 7.1 Hz, 3H).

^{13}C NMR (101 MHz, Chloroform-*d*) δ 192.7, 167.5, 166.7, 165.4, 161.5, 159.5, 136.3, 133.7, 130.7, 129.4, 128.9, 128.5, 127.7, 127.5, 121.8, 110.9, 108.1, 105.2, 94.1, 90.0, 70.4, 61.8, 55.5, 55.3, 37.1, 14.1.

HRMS (ESI) m/z: $[M + H]^+$ Calcd for $C_{35}H_{36}O_8^+$ 598.2435; Found 598.2435.

$[\alpha]_D^{23}$ = -196.5 (*c* 1.0, $CHCl_3$).

Chiral HPLC: Chiralpak AD-H, hexane:*i*PrOH = 70:30, 1.0 mL/min, 254 nm; tR = 8.5 min (minor), 11.1 min (major).

Ethyl (*R*)-6-(benzyloxy)-2-(4-cyanophenyl)-3-oxo-1-(2,4,6-trimethoxybenzyl)indoline-2-carboxylate (3f)

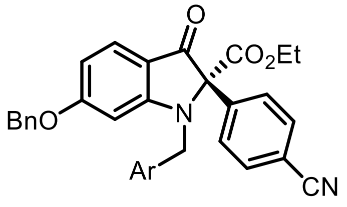

60 °C, 16 h. 24.0 mg, yellow solid, 81% yield, 95% *ee*, m.p. = 60 – 62 °C.

^{1}H NMR (400 MHz, Chloroform-*d*) δ 7.62 – 7.56 (m, 2H), 7.51 – 7.36 (m, 8H), 6.95 (d, J = 2.0 Hz, 1H), 6.46 (dd, J = 8.6, 2.0 Hz, 1H), 5.89 (s, 2H), 5.21 (s, 2H), 4.68 (d, J = 14.9 Hz, 1H), 4.58 (d, J = 14.9 Hz, 1H), 4.19 – 3.99 (m, 2H), 3.75 (s, 3H), 3.54 (s, 6H), 1.17 (t, J = 7.1 Hz, 3H).

^{13}C NMR (101 MHz, Chloroform-*d*) δ 191.8, 167.8, 166.2, 165.7, 161.7, 159.5, 140.1, 136.1, 131.2, 128.9, 128.6, 128.3, 127.73, 127.71, 119.1, 111.1, 110.7, 108.5, 105.0, 94.3, 90.0, 80.0, 70.5, 62.1, 55.5, 55.2, 37.4, 14.1.

HRMS (ESI) m/z: $[M + Na]^+$ Calcd for $C_{35}H_{32}N_2NaO_7^+$ 615.2102; Found 615.2104.

$[\alpha]_D^{23}$ = -224.8 (*c* 1.0, $CHCl_3$).

Chiral HPLC: Chiralpak AD-H, hexane:*i*PrOH = 70:30, 1.0 mL/min, 254 nm; tR = 10.0 min (minor), 12.0 min (major).

Ethyl (*R*)-6-(benzyloxy)-2-(4-nitrophenyl)-3-oxo-1-(2,4,6-trimethoxybenzyl)indoline-2-carboxylate (3g)

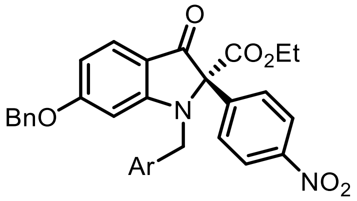

Ar = 2,4,6-$(OMe)_3C_6H_2$

60 °C, 16 h. 22.3 mg, yellow solid, 73 % yield, 94% *ee*, m.p. = 58 – 60 °C.

^{1}H NMR (400 MHz, Chloroform-*d*) δ 7.97 – 7.91 (m, 2H), 7.68 – 7.63 (m, 2H), 7.53 – 7.37 (m, 6H), 6.95 (d, *J* = 2.1 Hz, 1H), 6.47 (dd, *J* = 8.6, 2.0 Hz, 1H), 5.88 (s, 2H), 5.22 (s, 2H), 4.70 (d, *J* = 14.9 Hz, 1H), 4.60 (d, *J* = 14.9 Hz, 1H), 4.20 – 4.02 (m, 2H), 3.72 (s, 3H), 3.55 (s, 6H), 1.18 (t, *J* = 7.1 Hz, 3H).

^{13}C NMR (101 MHz, Chloroform-*d*) δ 191.6, 167.8, 166.2, 165.7, 161.8, 159.5, 147.2, 142.2, 136.2, 128.9, 128.59, 128.56, 127.8, 127.7, 122.5, 110.6, 108.6, 104.9, 94.3, 90.0, 80.0, 70.6, 62.2, 55.5, 55.3, 37.4, 14.1.

HRMS (ESI) m/z: $[M + Na]^+$ Calcd for $C_{34}H_{32}N_2NaO_9^+$ 635.2000; Found 635.2006.

$[\alpha]_D^{23}$ = -346.8 (*c* 0.5, $CHCl_3$).

Chiral HPLC: Chiralpak AD-H, hexane:*i*PrOH = 70:30, 1.0 mL/min, 254 nm; tR = 9.7 min (minor), 13.8 min (major).

Ethyl (*R*)-6-(benzyloxy)-3-oxo-2-(4-(trifluoromethyl)phenyl)-1-(2,4,6-trimethoxybenzyl)indoline-2-carboxylate (3h)

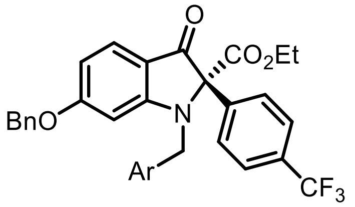

Ar = 2,4,6-$(OMe)_3C_6H_2$

60 °C, 16 h. 20.4 mg, yellow solid, 64 % yield, 92% *ee*, m.p. = 72 – 74 °C.

^{1}H NMR (400 MHz, Chloroform-*d*) δ 7.61 - 7.56 (m, 2H), 7.52 – 7.35 (m, 8H), 6.93 (d, *J* = 2.0 Hz, 1H), 6.46 (dd, *J* = 8.6, 2.0 Hz, 1H), 5.89 (s, 2H), 5.21 (s, 2H), 4.69 (d, *J* = 14.8 Hz, 1H), 4.62 (d, *J* = 14.9 Hz, 1H), 4.14 (dq, *J* = 10.8, 7.1 Hz, 1H), 4.03 (dq, *J*

= 10.8, 7.1 Hz, 1H), 3.73 (s, 3H), 3.55 (s, 6H), 1.17 (t, *J* = 7.1 Hz, 3H).

^{13}C NMR (101 MHz, Chloroform-*d*) δ 192.3, 167.7, 166.5, 165.6, 161.6, 159.5, 138.7, 136.2, 129.5 (q, *J* = 32.1 Hz), 128.9, 128.5, 128.0, 127.7, 127.6, 124.32 (q, *J* = 3.8 Hz), 124.31 (q, *J* = 272.1 Hz), 110.8, 108.3, 105.1, 94.1, 90.0, 79.9, 77.4, 70.5, 61.9, 55.4, 55.2, 37.1, 14.1.

^{19}F NMR (377 MHz, Chloroform-*d*) δ -62.9.

HRMS (ESI) m/z: $[M + Na]^+$ Calcd for $C_{35}H_{32}F_3NNaO_7^+$ 658.2023; Found 658.2030.

$[\alpha]_D^{23}$ = -158.7 (*c* 2.0, $CHCl_3$).

Chiral HPLC: Chiralpak AD-H, hexane:*i*PrOH = 70:30, 1.0 mL/min, 254 nm; tR = 5.6 min (minor), 6.8 min (major).

Ethyl (*R*)-6-(benzyloxy)-2-(4-(ethoxycarbonyl)phenyl)-3-oxo-1-(2,4,6-trimethoxybenzyl)indoline-2-carboxylate (3i)

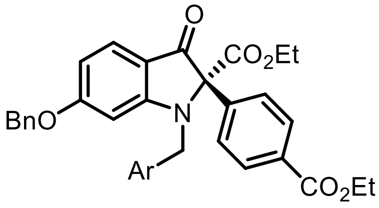

60 °C, 16 h. 25.6 mg, yellow oil, 80% yield, 93% *ee*.

^{1}H NMR (400 MHz, Chloroform-*d*) δ 7.85 – 7.80 (m, 2H), 7.57 – 7.53 (m, 2H), 7.50 – 7.36 (m, 6H), 6.92 (d, *J* = 2.0 Hz, 1H), 6.44 (dd, *J* = 8.6, 2.1 Hz, 1H), 5.90 (s, 2H), 5.20 (s, 2H), 4.66 (d, *J* = 14.9 Hz, 1H), 4.60 (d, *J* = 14.9 Hz, 1H), 4.34 (q, *J* = 7.1 Hz, 2H), 4.09 (dq, *J* = 10.7, 7.1 Hz, 1H), 3.94 (dq, *J* = 10.8, 7.1 Hz, 1H), 3.73 (s, 3H), 3.55 (s, 6H), 1.37 (t, *J* = 7.1 Hz, 3H), 1.13 (t, *J* = 7.1 Hz, 3H).

^{13}C NMR (101 MHz, Chloroform-*d*) δ 192.4, 167.6, 166.7, 165.5, 161.5, 159.6, 139.6, 136.3, 129.6, 128.91, 128.87, 128.5, 127.7, 127.63, 127.58, 111.0, 108.1, 105.2, 94.1, 90.1, 70.5, 61.8, 60.9, 55.5, 55.3, 37.2, 14.4, 14.1.

HRMS (ESI) m/z: $[M + Na]^+$ Calcd for $C_{37}H_{37}NNaO_9^+$ 662.2361; Found 662.2366.

$[\alpha]_D^{23}$ = -219.1 (*c* 1.0, $CHCl_3$).

Chiral HPLC: Chiralpak AD-H, hexane:*i*PrOH = 70:30, 1.0 mL/min, 254 nm; tR = 7.9 min (minor), 22.6 min (major).

Ethyl (*R*)-6-(benzyloxy)-2-(3-methoxyphenyl)-3-oxo-1-(2,4,6-trimethoxybenzyl)indoline-2-carboxylate (3j)

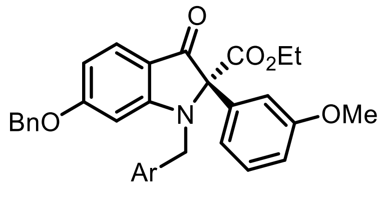

Ar = 2,4,6-$(OMe)_3C_6H_2$

60 °C, 16 h. 24.1 mg, yellow oil, 81% yield, 89% *ee*.

^{1}H NMR (400 MHz, Chloroform-*d*) δ 7.50 – 7.35 (m, 6H), 7.14 – 7.02 (m, 3H), 6.88 (d, *J* = 2.1 Hz, 1H), 6.73 (ddd, *J* = 8.0, 2.6, 1.2 Hz, 1H), 6.41 (dd, *J* = 8.6, 2.0 Hz, 1H), 5.94 (s, 2H), 5.17 (s, 2H), 4.65 (d, *J* = 14.9 Hz, 1H), 4.59 (d, *J* = 14.9 Hz, 1H), 4.08 (dq, *J* = 10.6, 7.0 Hz, 1H), 3.89 (dq, *J* = 10.8, 7.2 Hz, 1H), 3.75 (s, 3H), 3.70 (s, 3H), 3.58 (s, 6H), 1.10 (t, *J* = 7.1 Hz, 3H).

^{13}C NMR (101 MHz, Chloroform-*d*) δ 193.1, 167.4, 167.0, 165.3, 161.3, 159.7, 159.2, 136.4, 135.9, 128.9, 128.7, 128.5, 127.7, 127.5, 120.2, 113.7, 113.2, 111.2, 107.8, 105.6, 93.9, 90.1, 80.1, 70.4, 61.6, 55.5, 55.3, 55.2, 37.0, 29.8, 14.1.

HRMS (ESI) m/z: $[M + Na]^+$ Calcd for $C_{35}H_{35}NNaO_8^+$ 620.2255; Found 620.2255.

$[\alpha]_D^{23}$ = -162.7 (*c* 2.0, $CHCl_3$).

Chiral HPLC: Chiralpak AD-H, hexane:*i*PrOH = 70:30, 1.0 mL/min, 254 nm; tR = 7.5 min (minor), 10.0 min (major).

Ethyl (*R*)-6-(benzyloxy)-2-(3-fluorophenyl)-3-oxo-1-(2,4,6-trimethoxybenzyl)indoline-2-carboxylate (3k)

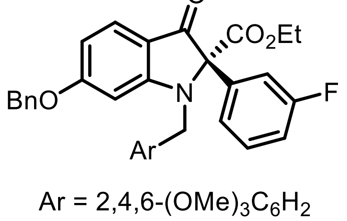

Ar = 2,4,6-$(OMe)_3C_6H_2$

60 °C, 16 h. 26.4 mg, yellow oil, 90% yield, 92% *ee*.

^{1}H NMR (400 MHz, Chloroform-*d*) δ 7.50 – 7.36 (m, 6H), 7.28 – 7.26 (m, 1H), 7.24 (ddd, *J* = 5.7, 2.7, 1.4 Hz, 2H), 7.11 (td, *J* = 8.2, 6.1 Hz, 1H), 6.90 – 6.82 (m, 2H), 6.43

(dd, J = 8.6, 2.0 Hz, 1H), 5.93 (s, 2H), 5.19 (s, 2H), 4.65 (d, J = 14.8 Hz, 1H), 4.60 (d, J = 14.8 Hz, 1H), 4.10 (dq, J = 10.8, 7.1 Hz, 1H), 3.96 (dq, J = 10.7, 7.1 Hz, 1H), 3.75 (s, 3H), 3.59 (s, 6H), 1.13 (t, J = 7.1 Hz, 3H).

^{13}C NMR (101 MHz, Chloroform-*d*) δ 192.6, 167.6, 166.6, 165.4, 162.4 (d, J = 243.5 Hz), 161.5, 159.6, 137.1 (d, J = 7.9 Hz), 136.3, 129.0 (d, J = 8.2 Hz), 128.9, 128.5, 127.7, 127.6, 123.3 (d, J = 2.9 Hz), 115.0 (d, J = 24.0 Hz), 114.4 (d, J = 21.1 Hz), 111.0, 108.1, 105.2, 94.1, 90.1, 79.7, 70.5, 61.8, 55.5, 55.3, 37.0, 14.1.

^{19}F NMR (376 MHz, Chloroform-*d*) δ -113.8.

HRMS (ESI) m/z: $[M + Na]^+$ Calcd for $C_{34}H_{32}FNNaO_7^+$ 608.2055; Found 608.2056.

$[\alpha]_D^{23}$ = -156.7 (*c* 1.0, $CHCl_3$).

Chiral HPLC: Chiralpak AD-H, hexane:*i*PrOH = 70:30, 1.0 mL/min, 254 nm; tR = 6.5 min (minor), 7.5 min (major).

Ethyl (*R*)-6-(benzyloxy)-2-(2-chlorophenyl)-3-oxo-1-(2,4,6-trimethoxybenzyl)indoline-2-carboxylate (3l)

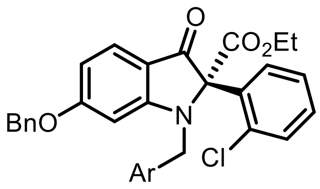

60 °C, 16 h. 25.0 mg, yellow oil, 83% yield, 70% *ee.*

^{1}H NMR (400 MHz, Chloroform-*d*) δ 7.54 (d, J = 8.5 Hz, 1H), 7.47 – 7.32 (m, 6H), 7.17 – 7.00 (m, 3H), 6.71 (s, 1H), 6.41 (dd, J = 8.6, 2.1 Hz, 1H), 5.79 (s, 2H), 5.12 (d, J = 11.5 Hz, 1H), 5.06 (d, J = 11.7 Hz, 1H), 4.69 (s, 2H), 4.30 (q, J = 7.1 Hz, 2H), 3.70 (s, 3H), 3.52 (s, 6H), 1.28 (t, J = 7.1 Hz, 3H).

^{13}C NMR (101 MHz, Chloroform-*d*) δ 191.6, 167.2, 167.0, 164.0, 160.8, 159.3, 136.5, 135.0, 133.0, 130.2, 129.3, 128.82, 128.4, 127.7, 126.3, 125.5, 113.3, 107.8, 105.5, 94.2, 89.8, 82.6, 70.3, 62.7, 55.4, 55.0, 37.3, 14.1.

Chiral HPLC: Chiralpak AD-H, hexane:*i*PrOH = 70:30, 1.0 mL/min, 254 nm; tR = 7.8 min (minor), 10.0 min (major).

HRMS (ESI) m/z: $[M + Na]^+$ Calcd for $C_{34}H_{32}ClNNaO_7^+$ 624.1760; Found 624.1763.

$[\alpha]_D^{23}$ = -26.8 (*c* 2.0, $CHCl_3$).

Ethyl (*R*)-6-(benzyloxy)-2-(4-fluoro-2-methylphenyl)-3-oxo-1-(2,4,6-trimethoxybenzyl)indoline-2-carboxylate (3m)

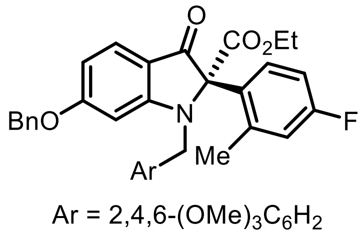

60 °C, 16 h. 26.4 mg, yellow oil, 88% yield, 76% *ee*.

^{1}H NMR (400 MHz, Chloroform-*d*) δ 7.53 (d, *J* = 8.6 Hz, 1H), 7.47 – 7.33 (m, 5H), 7.22 (dd, *J* = 8.7, 5.8 Hz, 1H), 6.75 – 6.67 (m, 2H), 6.49 (dd, *J* = 9.8, 2.8 Hz, 1H), 6.42 (dd, *J* = 8.6, 2.0 Hz, 1H), 5.80 (s, 2H), 5.14 (d, *J* = 11.7 Hz, 1H), 5.08 (d, *J* = 11.7 Hz, 1H), 4.69 (d, *J* = 15.0 Hz, 2H), 4.65 (d, *J* = 14.9 Hz, 2H), 4.31 (q, *J* = 7.1 Hz, 2H), 3.71 (s, 3H), 3.53 (s, 6H), 1.29 (t, *J* = 7.1 Hz, 3H).

^{13}C NMR (101 MHz, Chloroform-*d*) δ 192.2, 167.8, 167.2, 163.8, 162.2 (d, *J* = 246.5 Hz), 159.1, 141.9 (d, *J* = 7.9 Hz), 136.4, 132.9, 129.1 (d, J = 3.1 Hz), 128.9, 128.4, 127.7, 126.5, 117.5 (d, *J* = 21.1 Hz), 112.6, 111.0 (d, *J* = 21.0 Hz), 108.1, 105.7, 94.2, 89.8, 83.2, 70.3, 62.7, 55.5, 55.0, 37.1, 20.2, 14.2.

^{19}F NMR (376 MHz, Chloroform-*d*) δ -115.8.

HRMS (ESI) m/z: $[M + Na]^+$ Calcd for $C_{35}H_{34}FNNaO_7^+$ 622.2212; Found 622.2217.

$[\alpha]_D^{23}$ = -99.7 (*c* 1.0, $CHCl_3$).

Chiral HPLC: Chiralpak AD-H, hexane:*i*PrOH = 85:15, 1.0 mL/min, 254 nm; tR = 16.9 min (minor), 18.4 min (major).

Ethyl (*R*)-6-(benzyloxy)-2-(3,5-dimethylphenyl)-3-oxo-1-(2,4,6-trimethoxybenzyl)indoline-2-carboxylate (3n)

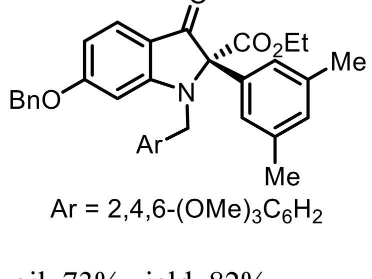

60 °C, 60 h. 21.8 mg, yellow oil, 73% yield, 82% *ee*.

^{1}H NMR (400 MHz, Chloroform-*d*) δ 7.52 – 7.35 (m, 6H), 7.00 (d, *J* = 1.6 Hz, 2H), 6.88 (d, *J* = 2.2 Hz, 1H), 6.78 (s, 1H), 6.41 (dd, *J* = 8.6, 2.0 Hz, 1H), 5.93 (s, 2H), 5.18 (s, 2H), 4.64 (d, *J* = 14.9 Hz, 1H), 4.58 (d, *J* = 15.0 Hz, 1H), 4.12 (dq, *J* = 10.8, 7.1 Hz, 1H), 3.98 (dq, *J* = 10.7, 7.1 Hz, 1H), 3.75 (s, 3H), 3.57 (s, 6H), 2.17 (s, 6H), 1.13 (t, *J* = 7.1 Hz, 3H).

^{13}C NMR (101 MHz, Chloroform-*d*) δ 193.5, 167.3, 167.1, 165.2, 161.2, 159.7, 136.9, 136.4, 134.3, 129.3, 128.9, 128.4, 127.8, 127.4, 125.5, 111.5, 107.6, 105.8, 94.0, 90.1, 70.3, 61.6, 55.4, 55.3, 37.1, 21.6, 14.1.

HRMS (ESI) m/z: $[M + Na]^+$ Calcd for $C_{36}H_{37}NNaO_7^+$ 618.2462; Found 618.2461.

$[\alpha]_D^{23}$ = -139.1 (*c* 1.0, $CHCl_3$).

Chiral HPLC: Chiralpak IC, hexane:*i*PrOH = 70:30, 1.0 mL/min, 254 nm; tR = 15.8 min (major), 21.2 min (minor).

Ethyl (*R*)-6-(benzyloxy)-2-(3,4-dichlorophenyl)-3-oxo-1-(2,4,6-trimethoxybenzyl)indoline-2-carboxylate (3o)

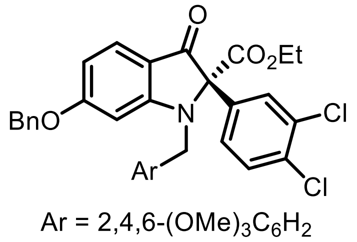

60 °C, 16 h. 23.3 mg, yellow oil, 73% yield, 94% *ee.*

^{1}H NMR (400 MHz, Chloroform-*d*) δ 7.51 – 7.36 (m, 8H), 7.17 (d, *J* = 8.5 Hz, 1H), 6.89 (d, *J* = 2.0 Hz, 1H), 6.46 (dd, *J* = 8.6, 2.0 Hz, 1H), 5.90 (s, 2H), 5.21 (s, 2H), 4.67 (d, *J* = 14.8 Hz, 1H), 4.58 (d, *J* = 14.8 Hz, 1H), 4.21 – 4.05 (m, 2H), 3.75 (s, 3H), 3.59 (s, 6H), 1.19 (t, *J* = 7.1 Hz, 3H).

^{13}C NMR (101 MHz, Chloroform-*d*) δ 192.1, 167.7, 166.2, 165.6, 161.6, 159.4, 136.2, 135.1, 131.5, 131.3, 129.5, 129.3, 128.9, 128.5, 127.73, 127.66, 127.4, 110.7, 108.4, 104.9, 94.2, 90.1, 70.5, 62.1, 55.5, 55.3, 37.1, 14.2.

HRMS (ESI) m/z: $[M + Na]^+$ Calcd for $C_{34}H_{31}Cl_2NNaO_7^+$ 658.1370; Found 658.1374.

$[\alpha]_D^{23}$ = -206.2 (*c* 1.0, $CHCl_3$).

Chiral HPLC: Chiralpak AD-H, hexane:*i*PrOH = 70:30, 1.0 mL/min, 254 nm; tR = 6.7

min (minor), 7.5 min (major).

Ethyl (*R*)-2-(benzo[d][1,3]dioxol-5-yl)-6-(benzyloxy)-3-oxo-1-(2,4,6-trimethoxybenzyl)indoline-2-carboxylate (3p)

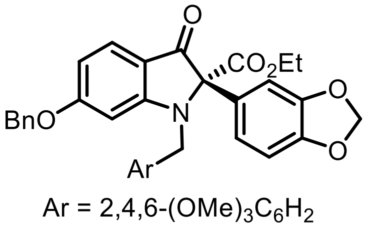

60 °C, 16 h. 26.9 mg, yellow oil, 88% yield, 90% *ee.*

^{1}H NMR (400 MHz, Chloroform-*d*) δ 7.51 – 7.34 (m, 6H), 6.99 – 6.91 (m, 2H), 6.82 (d, *J* = 2.1 Hz, 1H), 6.60 (d, *J* = 8.3 Hz, 1H), 6.41 (dd, *J* = 8.6, 2.0 Hz, 1H), 5.94 (s, 2H), 5.88 – 5.84 (m, 2H), 5.17 (s, 2H), 4.62 (d, *J* = 14.9 Hz, 1H), 4.58 (d, *J* = 14.7 Hz, 1H), 4.08 (dq, *J* = 10.8, 7.1 Hz, 1H), 3.90 (dq, *J* = 10.7, 7.1 Hz, 1H), 3.75 (s, 3H), 3.61 (s, 6H), 1.10 (t, *J* = 7.1 Hz, 3H).

^{13}C NMR (101 MHz, Chloroform-*d*) δ 193.4, 167.4, 167.0, 165.2, 161.3, 159.7, 147.2, 147.1, 136.4, 128.9, 128.5, 128.3, 127.7, 127.5, 121.3, 111.2, 108.8, 107.8, 107.6, 105.5, 101.0, 93.9, 90.1, 70.4, 61.7, 55.5, 55.4, 36.8, 14.1.

HRMS (ESI) m/z: $[M + Na]^+$ Calcd for $C_{35}H_{33}NNaO_9^+$ 634.2048; Found 634.2051.

$[\alpha]_D^{23}$ = -176.2 (*c* 0.5, $CHCl_3$).

Chiral HPLC: Chiralpak AD-H, hexane:*i*PrOH = 70:30, 1.0 mL/min, 254 nm; tR = 10.5 min (minor), 14.9 min (major).

Methyl (*R*)-6-(benzyloxy)-2-(naphthalen-1-yl)-3-oxo-1-(2,4,6-trimethoxybenzyl)indoline-2-carboxylate (3q)

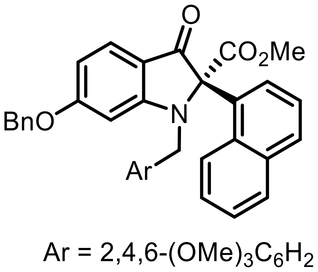

60 °C, 16 h. 27.8 mg, yellow oil, 92% yield, 83% *ee.*

^{1}H NMR (400 MHz, Chloroform-*d*) δ 7.69 – 7.56 (m, 3H), 7.50 – 7.45 (m, 2H), 7.45 – 7.39 (m, 3H), 7.39 – 7.34 (m, 1H), 7.33 – 7.28 (m, 1H), 7.25 – 7.21 (m, 1H), 7.09 (brs,

2H), 6.76 (s, 1H), 6.49 (dd, J = 8.6, 2.0 Hz, 1H), 5.35 (brs, 2H), 5.18 (d, J = 11.7 Hz, 1H), 5.11 (d, J = 11.5 Hz, 1H), 4.82 (d, J = 15.0 Hz, 1H), 4.69 (d, J = 15.1 Hz, 1H), 3.90 (s, 3H), 3.54 (s, 3H), 3.30 (s, 6H).

^{13}C NMR (101 MHz, Chloroform-*d*) δ 192.7, 168.9, 167.4, 163.5, 160.1, 158.5, 136.4, 134.0, 131.9, 130.9, 129.8, 128.9, 128.5, 128.4, 127.8, 126.8, 125.7, 125.2, 125.0, 124.0, 112.2, 108.4, 105.0, 94.3, 89.2, 70.4, 55.2, 54.6, 53.5, 37.1.

HRMS (ESI) m/z: $[M + Na]^+$ Calcd for $C_{37}H_{33}NNaO_7^+$ 626.2149; Found 626.2148.

$[\alpha]_D^{23}$ = -192.3 (*c* 1.0, $CHCl_3$).

Chiral HPLC: Chiralpak IG, hexane:*i*PrOH = 70:30, 1.0 mL/min, 254 nm; tR = 26.3 min (minor), 33.1 min (major).

Ethyl (*R*)-6-(benzyloxy)-2-(naphthalen-2-yl)-3-oxo-1-(2,4,6-trimethoxybenzyl)indoline-2-carboxylate (3r)

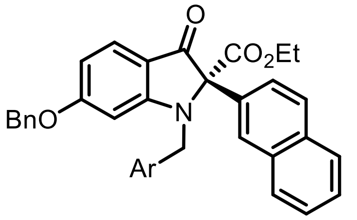

Ar = 2,4,6-$(OMe)_3C_6H_2$

60 °C, 36 h. 27.2 mg, yellow oil, 88% yield, 90% *ee.*

^{1}H NMR (400 MHz, Chloroform-*d*) δ 8.02 (d, J = 1.9 Hz, 1H), 7.73 – 7.69 (m, 1H), 7.67 – 7.64 (m, 1H), 7.60 (d, J = 8.8 Hz, 1H), 7.53 – 7.34 (m, 9H), 6.95 (d, J = 2.0 Hz, 1H), 6.45 (dd, J = 8.6, 2.0 Hz, 1H), 5.79 (s, 2H), 5.21 (s, 2H), 4.70 (d, J = 14.9 Hz, 2H), 4.65 (d, J = 14.9 Hz, 2H), 4.17 (dq J = 10.8 7.1 Hz, 1H), 4.03 (dq J = 10.8, 7.1 Hz, 1H), 3.59 (s, 3H), 3.52 (s, 6H), 1.17 (t, J = 7.1 Hz, 3H).

^{13}C NMR (101 MHz, Chloroform-*d*) δ 193.2, 167.5, 167.1, 165.4, 161.2, 159.6, 136.3, 133.0, 132.9, 132.1, 128.9, 128.51, 128.48, 127.8, 127.5, 127.3, 127.2, 127.1, 126.0, 125.53, 125.50, 111.3, 107.9, 105.5, 94.1, 90.0, 80.5, 70.4, 61.8, 55.34, 55.29, 37.1, 14.1.

HRMS (ESI) m/z: $[M + Na]^+$ Calcd for $C_{37}H_{33}NNaO_7^+$ 640.2306; Found 640.2316.

$[\alpha]_D^{23}$ = -227.6 (*c* 1.0, $CHCl_3$).

Chiral HPLC: Chiralpak AD-H, hexane:*i*PrOH = 70:30, 1.0 mL/min, 254 nm; tR = 9.6

min (minor), 14.0 min (major).

Methyl (*R*)-6-(benzyloxy)-3-oxo-2-(thiophen-3-yl)-1-(2,4,6-trimethoxybenzyl)indoline-2-carboxylate (3s)

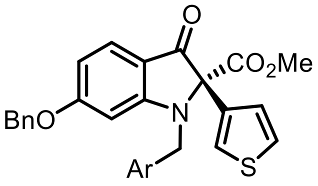

Ar = 2,4,6-$(OMe)_3C_6H_2$

60 °C, 16 h. 23.8 mg, yellow solid, 85% yield, 97% *ee*, m.p. = 120 – 122 °C.

^{1}H NMR (400 MHz, Chloroform-*d*) δ 7.71 (dd, *J* = 3.1, 1.3 Hz, 1H), 7.51 – 7.41 (m, 5H), 7.41 – 7.36 (m, 1H), 7.08 (dd, *J* = 5.1, 3.0 Hz, 1H), 6.90 (d, *J* = 2.1 Hz, 1H), 6.86 (dd, *J* = 5.0, 1.3 Hz, 1H), 6.42 (dd, *J* = 8.7, 2.0 Hz, 1H), 5.97 (s, 2H), 5.20 (s, 2H), 4.56 (s, 2H), 3.76 (s, 3H), 3.63 (s, 6H), 3.46 (s, 3H).

^{13}C NMR (101 MHz, Chloroform-*d*) δ 192.1, 167.6, 167.4, 165.0, 161.3, 159.6, 136.3, 134.4, 128.9, 128.5, 127.7, 127.6, 126.8, 124.33, 124.29, 110.8, 107.9, 105.4, 93.8, 90.1, 70.4, 55.5, 55.4, 52.5, 36.6, 13.9.

HRMS (ESI) m/z: $[M + Na]^+$ Calcd for $C_{31}H_{29}NNaO_7S^+$ 582.1557; Found 582.1560.

$[\alpha]_D^{23}$ = -123.5 (*c* 1.0, $CHCl_3$).

Chiral HPLC: Chiralpak AD-H, hexane:*i*PrOH = 70:30, 1.0 mL/min, 254 nm; tR = 9.2 min (minor), 12.0 min (major).

Ethyl (*R*)-6-(benzyloxy)-3-oxo-2-(thiophen-2-yl)-1-(2,4,6-trimethoxybenzyl)indoline-2-carboxylate (3t)

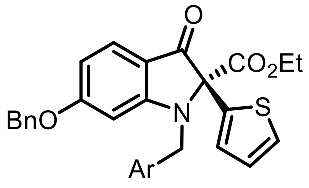

Ar = 2,4,6-$(OMe)_3C_6H_2$

60 °C, 16 h. 26.3 mg, yellow solid, 92% yield, 98% *ee*, m.p. = 126 – 128 °C.

^{1}H NMR (400 MHz, Chloroform-*d*) δ 7.53 – 7.35 (m, 6H), 7.19 (dd, *J* = 5.1, 1.3 Hz, 1H), 6.97 – 6.93 (m, 1H), 6.92 – 6.88 (m, 1H), 6.85 – 6.81 (m, 1H), 6.43 (dd, *J* = 8.6, 2.0 Hz, 1H), 5.98 (s, 2H), 5.21 (s, 2H), 4.64 (d, *J* = 15.0 Hz, 2H), 4.60 (d, *J* = 15.3 Hz, 2H), 4.08 – 3.99 (m, 1H), 3.80 – 3.71 (m, 4H), 3.65 (d, *J* = 1.3 Hz, 6H), 1.02 (t, *J* = 7.1

Hz, 3H).

^{13}C NMR (101 MHz, Chloroform-*d*) δ 191.6, 167.6, 166.8, 165.0, 161.3, 159.7, 136.3, 136.1, 128.9, 128.5, 127.9, 127.8, 126.2, 125.8, 125.6, 110.0, 107.9, 105.7, 94.0, 90.0, 70.4, 62.0, 55.5, 55.4, 36.4, 13.9.

HRMS (ESI) m/z: $[M + Na]^+$ Calcd for $C_{32}H_{31}NNaO_7S^+$ 596.1713; Found 596.1711.

$[\alpha]_D^{23}$ = -174.5 (*c* 1.0, $CHCl_3$).

Chiral HPLC: Chiralpak AD-H, hexane:*i*PrOH = 70:30, 1.0 mL/min, 254 nm; tR = 7.7 min (minor), 9.8 min (major).

Ethyl (*R*)-2-(4-((2-acetoxybenzoyl)oxy)phenyl)-6-(benzyloxy)-3-oxo-1-(2,4,6-trimethoxybenzyl)indoline-2-carboxylate (3u)

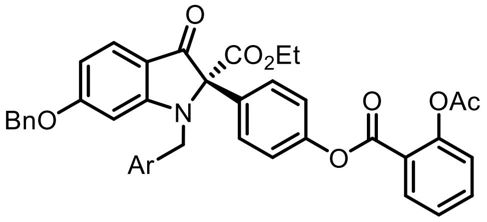

Ar = 2,4,6-$(OMe)_3C_6H_2$

60 °C, 16 h. 34.3 mg, yellow oil, 92% yield, 95% *ee.*

^{1}H NMR (400 MHz, Chloroform-*d*) δ 8.17 (dd, *J* = 7.9, 1.7 Hz, 1H), 7.62 (ddd, *J* = 8.1, 7.4, 1.7 Hz, 1H), 7.54 – 7.35 (m, 9H), 7.16 (dd, *J* = 8.1, 1.1 Hz, 1H), 7.00 – 6.94 (m, 2H), 6.92 (d, *J* = 2.1 Hz, 1H), 6.43 (dd, *J* = 8.6, 2.0 Hz, 1H), 5.96 (s, 2H), 5.19 (s, 2H), 4.69 (d, *J* = 14.8 Hz, 1H), 4.60 (d, *J* = 14.9 Hz, 1H), 4.11 (dq, *J* = 10.8, 7.1 Hz, 1H), 3.95 (dq, *J* = 10.7, 7.1 Hz, 1H), 3.75 (s, 3H), 3.58 (s, 6H), 2.28 (s, 3H), 1.13 (t, *J* = 7.1 Hz, 3H).

^{13}C NMR (101 MHz, Chloroform-*d*) δ 193.1, 169.9, 167.5, 167.0, 165.4, 163.0, 161.5, 159.6, 151.2, 150.1, 136.3, 134.6, 132.4, 132.3, 129.0, 128.9, 128.5, 127.7, 127.5, 126.3, 124.1, 122.9, 120.7, 111.2, 108.0, 105.3, 94.1, 90.1, 80.0, 70.4, 61.8, 55.5, 55.3, 37.2, 21.1, 14.1.

HRMS (ESI) m/z: $[M + Na]^+$ Calcd for $C_{43}H_{39}NNaO_{11}^+$ 768.2415; Found 768.2416.

$[\alpha]_D^{23}$ = -143.3 (*c* 1.0, $CHCl_3$).

Chiral HPLC: Chiralpak AD-H, hexane:*i*PrOH = 70:30, 1.0 mL/min, 254 nm; tR = 14.7 min (minor), 23.8 min (major).

Ethyl (R)-6-(benzyloxy)-2-(4-(((S)-2-(4-isobutylphenyl)propanoyl)oxy)phenyl)-3-oxo-1-(2,4,6-trimethoxybenzyl)indoline-2-carboxylate (3v)

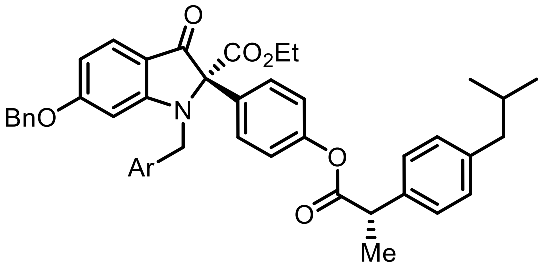

Ar = 2,4,6-(OMe)$_3$C$_6$H$_2$

60 °C, 16 h. 30.7 mg, yellow oil, 80% yield, >20/1 *dr*.

^{1}**H NMR** (major) (400 MHz, Chloroform-*d*) δ 7.43 – 7.26 (m, 8H), 7.22 – 7.16 (m, 2H), 7.05 (dd, *J* = 8.2, 2.2 Hz, 2H), 6.82 (d, *J* = 2.1 Hz, 1H), 6.74 – 6.68 (m, 2H), 6.33 (dd, *J* = 8.6, 2.0 Hz, 1H), 5.84 (s, 2H), 5.10 (s, 2H), 4.57 (d, *J* = 14.8 Hz, 1H), 4.48 (d, *J* = 14.8 Hz, 1H), 3.99 (dq, *J* = 10.8, 7.1 Hz, 1H), 3.89 – 3.77 (m, 2H), 3.63 (s, 3H), 3.47 (s, 6H), 2.39 (d, *J* = 7.2 Hz, 2H), 1.79 (sept, *J* = 6.8 Hz, 1H), 1.50 (dd, *J* = 7.2, 1.2 Hz, 3H), 1.03 (t, *J* = 7.1 Hz, 3H), 0.83 (dd, *J* = 6.6, 1.8 Hz, 6H).

^{13}**C NMR** (major) (101 MHz, Chloroform-*d*) δ 193.0, 173.2, 167.4, 167.0, 165.4, 161.4, 159.6, 150.4, 140.9, 137.4, 136.3, 131.9, 129.6, 128.9, 128.7, 128.5, 127.7, 127.5, 127.3, 120.5, 111.2, 107.9, 105.3, 94.1, 90.1, 79.9, 70.4, 61.7, 55.4, 55.3, 45.3, 45.2, 37.1, 30.3, 22.5, 18.6, 14.1.

HRMS (ESI) m/z: [M + Na]$^+$ Calcd for $C_{47}H_{49}NNaO_9^+$ 794.3300; Found 794.3302.

$[\alpha]_D^{23}$ = -132.8 (*c* 1.0, CHCl$_3$).

Ethyl (*R,E*)-6-(benzyloxy)-3-oxo-2-styryl-1-(2,4,6-trimethoxybenzyl)indoline-2-carboxylate (3w)

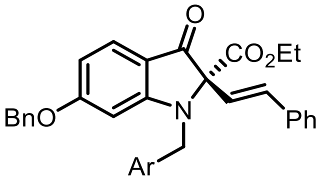

Ar = 2,4,6-(OMe)$_3$C$_6$H$_2$

Toluene, 40 °C, 36 h, 16.3 mg, yellow oil, 55% yield, 96% *ee*.

^{1}**H NMR** (400 MHz, Chloroform-*d*) δ 7.51 – 7.42 (m, 5H), 7.40 – 7.36 (m, 1H), 7.24 – 7.12 (m, 5H), 6.90 (d, *J* = 2.1 Hz, 1H), 6.67 (d, *J* = 16.4 Hz, 1H), 6.42 (dd, *J* = 8.6, 2.0

Hz, 1H), 6.13 (d, J = 16.3 Hz, 1H), 5.99 (s, 2H), 5.21 (s, 2H), 4.71 (d, J = 14.8 Hz, 1H), 4.60 (d, J = 14.8 Hz, 1H), 4.09 (dq, J = 10.7, 7.1 Hz, 1H), 3.99 (dq, J = 10.7, 7.1 Hz, 1H), 3.72 (s, 6H), 3.68 (s, 3H), 1.14 (t, J = 7.1 Hz, 3H).

^{13}C NMR (101 MHz, Chloroform-*d*) δ 193.1, 167.5, 167.3, 165.1, 161.4, 159.7, 136.9, 136.4, 130.7, 128.9, 128.5, 128.3, 127.7, 127.6, 127.5, 126.8, 123.2, 111.1, 107.7, 106.1, 93.9, 90.3, 70.4, 62.0, 55.6, 55.4, 36.5, 14.2.

HRMS (ESI) m/z: $[M + Na]^+$ Calcd for $C_{36}H_{35}NNaO_7^+$ 616.2306; Found 616.2309.

$[\alpha]_D^{23}$ = -113.8 (*c* 0.5, $CHCl_3$).

Chiral HPLC: Chiralpak AD-H, hexane:*i*PrOH = 70:30, 1.0 mL/min, 254 nm; tR = 9.1 min (minor), 14.3 min (major).

Ethyl (*R*)-6-(benzyloxy)-2-cyclohexyl-3-oxo-1-(2,4,6-trimethoxybenzyl)indoline-2-carboxylate (3x)

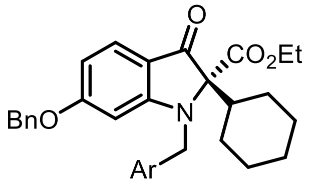

40 °C, 48 h. 16.3 mg, yellow oil, 57% yield, 96% *ee.*

^{1}H NMR (400 MHz, Chloroform-*d*) δ 7.47 – 7.33 (m, 6H), 6.62 (d, J = 2.0 Hz, 1H), 6.35 (dd, J = 8.6, 2.0 Hz, 1H), 6.08 (s, 2H), 5.11 (d, J = 12.2 Hz, 1H), 5.08 (d, J = 12.4 Hz, 1H), 4.54 (d, J = 15.1 Hz, 1H), 4.50 (d, J = 15.0 Hz, 1H), 3.74 (s, 6H), 3.61 (dq, J = 10.8, 7.1 Hz, 1H), 2.43 (tt, J = 12.0, 2.7 Hz, 1H), 2.08 – 2.01 (m, 1H), 1.84 – 1.71 (m, 2H), 1.65 – 1.53 (m, 3H), 1.43 – 1.38 (m, 1H), 1.25 – 1.16 (m, 1H), 1.08 – 0.95 (m, 4H), 0.80 – 0.68 (m, 1H).

^{13}C NMR (101 MHz, Chloroform-*d*) δ 195.5, 168.0, 166.8, 164.3, 161.3, 160.0, 136.5, 128.8, 128.3, 127.7, 125.8, 114.5, 106.8, 105.5, 93.7, 90.3, 78.4, 70.2, 61.0, 55.6, 55.5, 40.7, 35.9, 27.1, 26.93, 26.85, 26.6, 25.8, 14.0.

HRMS (ESI) m/z: $[M + Na]^+$ Calcd for $C_{34}H_{39}NNaO_7^+$ 596.2619; Found 596.2624.

$[\alpha]_D^{23}$ = +104.2 (*c* 1.0, $CHCl_3$).

Chiral HPLC: Chiralpak AD-H, hexane:*i*PrOH = 70:30, 1.0 mL/min, 254 nm; tR = 4.8 min (minor), 6.6 min (major).

Ethyl (*R*)-6-(benzyloxy)-3-oxo-2-propyl-1-(2,4,6-trimethoxybenzyl)indoline-2-carboxylate (3y)

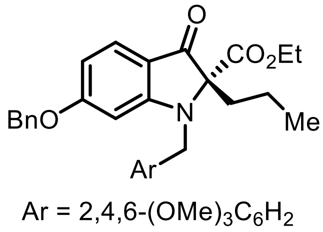

40 ºC, 48 h. 16.9 mg, yellow oil, 61% yield, 97% *ee*.

^{1}H NMR (400 MHz, Chloroform-*d*) δ 7.50 – 7.34 (m, 6H), 6.78 (d, *J* = 2.1 Hz, 1H), 6.37 (dd, *J* = 8.6, 2.0 Hz, 1H), 6.09 (s, 2H), 5.17 (s, 2H), 4.57 (d, *J* = 14.9 Hz, 1H), 4.52 (d, *J* = 14.9 Hz, 1H), 3.99 – 3.90 (m, 1H), 3.83 – 3.75 (m, 4H), 3.75 (s, 6H), 2.23 (ddd, *J* = 14.1, 11.9, 5.1 Hz, 1H), 1.91 (ddd, *J* = 14.1, 12.2, 3.9 Hz, 1H), 1.04 (t, *J* = 7.1 Hz, 3H), 0.90 – 0.70 (m, 2H), 0.63 (t, *J* = 7.2 Hz, 3H).

^{13}C NMR (101 MHz, Chloroform-*d*) δ 195.0, 168.2, 167.3, 165.1, 161.4, 159.8, 136.5, 128.9, 128.4, 127.7, 126.4, 113.2, 107.1, 105.7, 93.6, 90.3, 78.2, 70.4, 61.4, 55.6, 55.5, 35.8, 34.1, 15.8, 14.11, 14.06.

HRMS (ESI) m/z: $[M + Na]^+$ Calcd for $C_{31}H_{35}NNaO_7^+$ 556.2306; Found 556.2315.

$[\alpha]_D^{23}$ = +126.7 (*c* 1.0, $CHCl_3$).

Chiral HPLC: Chiralpak AD-H, hexane:*i*PrOH = 90:10, 1.0 mL/min, 254 nm; tR = 18.8 min (minor), 20.7 min (major).

Ethyl (*R*)-6-(benzyloxy)-2-(chloromethyl)-3-oxo-1-(2,4,6-trimethoxybenzyl)indoline-2 - carboxylate (3z)

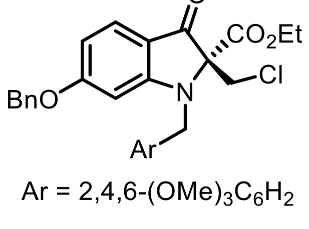

40 ºC, 36 h. 15.2 mg, yellow oil, 58% yield, 97% *ee*.

^{1}H NMR (400 MHz, Chloroform-*d*) δ 7.50 – 7.46 (m, 3H), 7.45 – 7.41 (m, 2H), 7.39 – 7.37 (m, 1H), 6.83 (d, *J* = 2.0 Hz, 1H), 6.41 (dd, *J* = 8.6, 2.0 Hz, 1H), 6.08 (s, 2H), 5.19 (s, 2H), 4.64 (d, *J* = 15.1 Hz, 1H), 4.54 (d, *J* = 15.1 Hz, 1H), 4.26 (d, *J* = 11.7 Hz, 1H), 4.20 (d, *J* = 11.7 Hz, 1H). 3.89 – 3.82 (m, 1H), 3.80 (s, 3H), 3.76 (s, 6H), 3.60 (dq, *J* =

10.7, 7.1 Hz, 1H), 0.95 (t, *J* = 7.1 Hz, 3H).

^{13}C NMR (101 MHz, Chloroform-*d*) δ 192.1, 167.7, 166.7, 165.7, 161.5, 159.8, 136.3, 128.9, 128.5, 127.7, 126.7, 112.9, 107.6, 105.1, 93.7, 90.3, 76.2, 70.4, 61.9, 55.7, 55.5, 43.2, 35.7, 13.8.

HRMS (ESI) m/z: $[M + Na]^+$ Calcd for $C_{29}H_{30}ClNNaO_7^+$ 562.1603; Found 562.1603.

$[\alpha]_D^{23}$ = +143.6 (*c* 0.5, $CHCl_3$).

Chiral HPLC: Chiralpak IG, hexane:*i*PrOH = 70:30, 1.0 mL/min, 254 nm; tR = 12.8 min (minor), 15.9 min (major).

Methyl (*R*)-6-(benzyloxy)-3-oxo-2-phenyl-1-(2,4,6-trimethoxybenzyl)indoline-2-carboxylate (3aa)

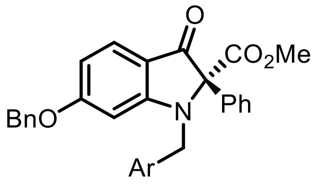

60 °C, 16 h. 23.5 mg, yellow solid, 85% yield, 90% *ee*, m.p. = 151 – 153 °C.

^{1}H NMR (400 MHz, Chloroform-*d*) δ 7.50 – 7.36 (m, 8H), 7.24 – 7.19 (m, 3H), 6.89 (d, *J* = 2.1 Hz, 1H), 6.42 (dd, *J* = 8.6, 2.0 Hz, 1H), 5.95 (s, 2H), 5.18 (s, 2H), 4.64 (d, *J* = 14.9 Hz, 1H), 4.59 (d, *J* = 14.8 Hz, 1H), 3.75 (s, 3H), 3.56 (s, 6H), 3.46 (s, 3H).

^{13}C NMR (101 MHz, Chloroform-*d*) δ 193.2, 167.7, 167.5, 165.2, 161.4, 159.8, 136.4, 134.2, 128.9, 128.5, 127.9, 127.8, 127.74, 127.72, 127.5, 111.2, 107.9, 105.5, 94.0, 90.2, 79.9, 70.4, 55.5, 55.4, 52.4, 36.9.

HRMS (ESI) m/z: $[M + Na]^+$ Calcd for $C_{33}H_{31}NNaO_7^+$576.1993; Found 576.1998.

$[\alpha]_D^{23}$ = -160.0 (*c* 0.5, $CHCl_3$).

Chiral HPLC: Chiralpak AD-H, hexane:*i*PrOH = 70:30, 1.0 mL/min, 254 nm; tR = 8.3 min (minor), 11.9 min (major).

Isopropyl (*R*)-6-(benzyloxy)-3-oxo-2-phenyl-1-(2,4,6-trimethoxybenzyl)indoline-2-carboxylate (3ab)

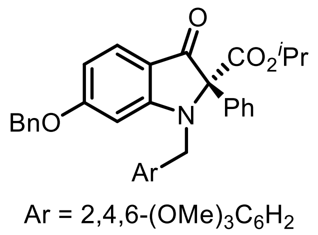

60 °C, 16 h. 23.3 mg, yellow oil, 80% yield, 91% *ee.*

^{1}H NMR (400 MHz, Chloroform-*d*) δ 7.50 – 7.35 (m, 8H), 7.16 – 7.11 (m, 3H), 6.90 (d, *J* = 2.1 Hz, 1H), 6.41 (dd, *J* = 8.6, 2.1 Hz, 1H), 5.92 (s, 2H), 5.17 (s, 2H), 4.97 (sept, *J* = 6.2 Hz, 1H), 4.66 (d, *J* = 14.9 Hz, 1H), 4.58 (d, *J* = 14.9 Hz, 1H), 3.75 (s, 3H), 3.56 (s, 6H), 1.18 (d, *J* = 6.2 Hz, 3H), 1.12 (d, *J* = 6.3 Hz, 3H).

^{13}C NMR (101 MHz, Chloroform-*d*) δ 193.5, 167.3, 166.5, 165.5, 161.3, 159.6, 136.4, 134.8, 128.9, 128.4, 127.8, 127.7, 127.6, 127.5, 127.4, 111.5, 107.8, 105.8, 94.1, 90.1, 70.3, 69.5, 55.5, 55.2, 37.4, 21.62, 21.56.

HRMS (ESI) m/z: $[M + Na]^+$ Calcd for $C_{35}H_{35}NNaO_7^+$604.2306; Found 604.2306.

$[\alpha]_D^{23}$ = -169.8 (*c* 1.0, $CHCl_3$).

Chiral HPLC: Chiralpak IG, hexane:*i*PrOH = 70:30, 1.0 mL/min, 254 nm; tR = 13.2 min (minor), 14.7 min (major).

***Tert*-butyl (*R*)-6-(benzyloxy)-3-oxo-2-phenyl-1-(2,4,6-trimethoxybenzyl)indoline-2-carboxylate (3ac)**

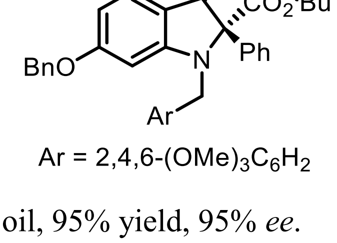

60 °C, 16 h. 28.4 mg, yellow oil, 95% yield, 95% *ee.*

^{1}H NMR (400 MHz, Chloroform-*d*) δ 7.51 – 7.35 (m, 8H), 7.15 – 7.07 (m, 3H), 6.89 (d, *J* = 2.1 Hz, 1H), 6.40 (dd, *J* = 8.6, 2.0 Hz, 1H), 5.92 (s, 2H), 5.18 (d, *J* = 11.6 Hz, 1H), 5.15 (d, *J* = 11.6 Hz, 1H), 4.68 (d, *J* = 14.9 Hz, 1H), 4.60 (d, *J* = 14.9 Hz, 1H), 3.75 (s, 3H), 3.55 (s, 6H), 1.38 (s, 9H).

^{13}C NMR (101 MHz, Chloroform-*d*) δ 193.7, 167.2, 165.7, 165.5, 161.3, 159.7, 136.4,

134.8, 128.8, 128.4, 127.8, 127.7, 127.42, 127.37, 127.3, 111.5, 107.6, 106.0, 94.1, 90.2, 82.2, 81.4, 70.3, 55.5, 55.2, 37.6, 27.9.

HRMS (ESI) m/z: $[M + Na]^+$ Calcd for $C_{36}H_{37}NNaO_7^+$ 618.2462; Found 618.2466.

$[\alpha]_D^{23}$ = -214.1 (*c* 1.0, $CHCl_3$).

Chiral HPLC: Chiralpak IC, hexane:*i*PrOH = 70:30, 1.0 mL/min, 254 nm; tR = 10.1 min (major), 26.0 min (minor).

2-(trimethylsilyl)ethyl (*R*)-6-(benzyloxy)-3-oxo-2-phenyl-1-(2,4,6-trimethoxybenzyl)indoline-2-carboxylate (3ad)

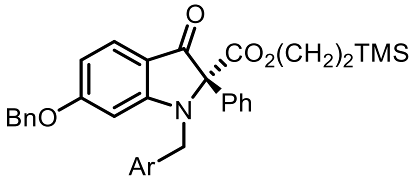

60 °C, 16 h. 28.1 mg, yellow oil, 88% yield, 91% *ee*.

^{1}H NMR (400 MHz, Chloroform-*d*) δ 7.52 – 7.35 (m, 8H), 7.22 – 7.16 (m, 3H), 6.91 (d, *J* = 2.1 Hz, 1H), 6.41 (dd, *J* = 8.6, 2.1 Hz, 1H), 5.94 (s, 2H), 5.18 (s, 2H), 4.68 (d, *J* = 14.9 Hz, 1H), 4.60 (d, *J* = 14.9 Hz, 1H)., 4.10 (ddd, *J* = 11.8, 10.8, 6.0 Hz, 1H), 3.86 (ddd, *J* = 11.9, 10.8, 5.4 Hz, 1H), 3.75 (s, 3H), 3.56 (s, 6H), 0.86 (ddd, *J* = 13.6, 11.9, 6.0 Hz, 1H), 0.76 (ddd, *J* = 13.6, 11.7, 5.4 Hz, 1H), -0.05 (s, 9H).

^{13}C NMR (101 MHz, Chloroform-*d*) δ 193.3, 167.34, 167.26, 165.3, 161.3, 159.7, 136.4, 134.5, 128.9, 128.4, 127.79, 127.77, 127.7, 127.6, 127.4, 111.4, 107.7, 105.7, 94.0, 90.1, 70.3, 64.0, 55.4, 55.3, 37.0, 17.1, -1.5.

HRMS (ESI) m/z: $[M + Na]^+$ Calcd for $C_{37}H_{41}NNaO_7Si^+$ 662.2545; Found 662.2545.

$[\alpha]_D^{23}$ = -133.7 (*c* 1.0, $CHCl_3$).

Chiral HPLC: Chiralpak AD-H, hexane:*i*PrOH = 70:30, 1.0 mL/min, 254 nm; tR = 5.6 min (minor), 8.2 min (major).

Ethyl (*R*)-6-methoxy-3-oxo-2-phenyl-1-(2,4,6-trimethoxybenzyl)indoline-2-carboxylate (3ae)

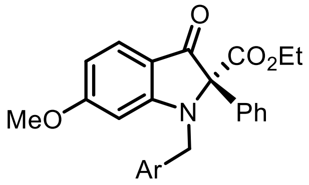

Ar = 2,4,6-$(OMe)_3C_6H_2$

60 °C, 16 h. 22.8 mg, yellow oil, 93% yield, 90% *ee*.

^{1}H NMR (400 MHz, Chloroform-*d*) δ 7.49 – 7.42 (m, 3H), 7.20 – 7.16 (m, 3H), 6.80 (d, *J* = 2.1 Hz, 1H), 6.33 (dd, *J* = 8.6, 2.1 Hz, 1H), 5.95 (s, 2H), 4.65 (d, *J* = 14.9 Hz, 1H), 4.59 (d, *J* = 14.9 Hz, 1H), 4.08 (dq, *J* = 10.7, 7.1 Hz, 1H), 3.93 – 3.84 (m, 4H), 3.75 (s, 3H), 3.59 (s, 6H), 1.09 (t, *J* = 7.1 Hz, 3H).

^{13}C NMR (101 MHz, Chloroform-*d*) δ 193.3, 168.2, 167.2, 165.4, 161.3, 159.7, 134.5, 127.8, 127.7, 127.6, 127.3, 111.1, 107.4, 105.7, 93.0, 90.1, 80.3, 61.6, 55.6, 55.5, 55.3, 37.1, 14.0.

HRMS (ESI) m/z: $[M + Na]^+$ Calcd for $C_{28}H_{29}NNaO_7^+$ 514.1836; Found 514.1835.

$[\alpha]_D^{23}$ = -173.1 (*c* 1.0, $CHCl_3$).

Chiral HPLC: Chiralpak AD-H, hexane:*i*PrOH = 70:30, 1.0 mL/min, 254 nm; tR = 6.5 min (minor), 8.6 min (major).

Ethyl (*R*)-6-isopropoxy-3-oxo-2-phenyl-1-(2,4,6-trimethoxybenzyl)indoline-2-carboxylate (3af)

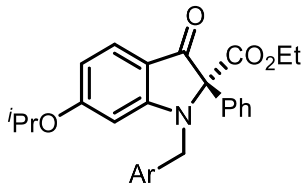

Ar = 2,4,6-$(OMe)_3C_6H_2$

60 °C, 16 h. 24.3 mg, yellow solid, 93% yield, 88% *ee*, m.p. = 117 – 119 °C.

^{1}H NMR (400 MHz, Chloroform-*d*) δ 7.48 – 7.43 (m, 2H), 7.42 (d, *J* = 8.5 Hz, 1H), 7.20 – 7.16 (m, 3H), 6.72 (d, *J* = 2.1 Hz, 1H), 6.29 (dd, *J* = 8.6, 2.0 Hz, 1H), 5.94 (s, 2H), 4.74 – 4.53 (m, 3H), 4.08 (dq, *J* = 10.6, 7.1 Hz, 1H), 3.90 (dq, *J* = 10.6, 7.2 Hz, 1H), 3.75 (s, 3H), 3.58 (s, 6H), 1.43 (d, *J* = 2.5 Hz, 1H), 1.41 (d, *J* = 2.4 Hz, 1H), 1.11

(t, J = 7.1 Hz, 3H).

^{13}C NMR (101 MHz, Chloroform-*d*) δ 193.2, 167.2, 166.7, 165.3, 161.3, 159.7, 134.6, 127.8, 127.6, 127.4, 110.8, 108.3, 105.6, 94.4, 90.1, 80.3, 70.3, 61.6, 55.5, 55.3, 37.0, 22.3, 22.3, 14.1.

HRMS (ESI) m/z: $[M + Na]^+$ Calcd for $C_{30}H_{33}NNaO_7^+$ 542.2149; Found 542.2150.

$[\alpha]_D^{23}$ = -162.9 (*c* 1.0, $CHCl_3$).

Chiral HPLC: Chiralpak AD-H, hexane:*i*PrOH = 70:30, 1.0 mL/min, 254 nm; tR = 4.8 min (minor), 6.2 min (major).

Ethyl (*R*)-6-((4-methoxybenzyl)oxy)-3-oxo-2-phenyl-1-(2,4,6-trimethoxybenzyl)indoline-2-carboxylate (3ag)

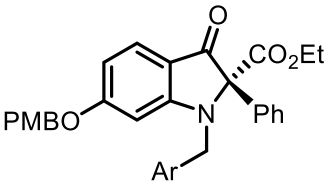

60 °C, 16 h. 24.4 mg, yellow solid, 82% yield, 90% *ee*, m.p. = 139 – 141 °C.

^{1}H NMR (400 MHz, Chloroform-*d*) δ 7.49 – 7.37 (m, 5H), 7.22 – 7.15 (m, 3H), 6.98 – 6.93 (m, 1H), 6.39 (dd, J = 8.6, 2.0 Hz, 1H), 5.94 (s, 2H), 5.10 (s, 2H), 4.65 (d, J = 14.9 Hz, 1H), 4.59 (d, J = 14.9 Hz, 1H), 4.07 (dq, J = 10.7, 7.1 Hz, 1H), 3.93 – 3.83 (m, 4H), 3.75 (s, 3H), 3.57 (s, 6H), 1.10 (t, J = 7.1 Hz, 3H).

^{13}C NMR (101 MHz, Chloroform-*d*) δ 193.4, 167.5, 167.2, 165.4, 161.3, 159.9, 159.7, 134.5, 129.6, 128.4, 127.80, 127.76, 127.6, 127.4, 114.3, 111.2, 107.9, 105.7, 93.9, 90.1, 80.2, 70.2, 61.6, 55.5, 55.3, 37.0, 14.1.,

HRMS (ESI) m/z: $[M + Na]^+$ Calcd for $C_{35}H_{35}NNaO_8^+$ 620.2255; Found 620.2254.

$[\alpha]_D^{23}$ = -177.6 (*c* 1.0, $CHCl_3$).

Chiral HPLC: Chiralpak AD-H, hexane:*i*PrOH = 70:30, 1.0 mL/min, 254 nm; tR = 8.6 min (minor), 12.4 min (major).

Ethyl (*R*)-6-((4-fluorobenzyl)oxy)-3-oxo-2-phenyl-1-(2,4,6-trimethoxybenzyl)indoline-2-carboxylate (3ah)

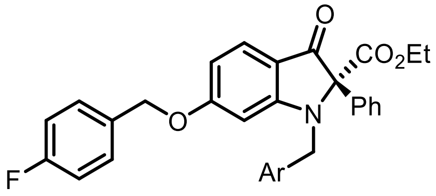

Ar = 2,4,6-$(OMe)_3C_6H_2$

60 °C, 16 h. 28.1 mg, yellow oil, 96% yield, 91% *ee*, m.p. = 139 – 141 °C.

^{1}H NMR (400 MHz, Chloroform-*d*) δ 7.49 – 7.40 (m, 5H), 7.20 – 7.09 (m, 5H), 6.87 (d, *J* = 2.1 Hz, 1H), 6.39 (dd, *J* = 8.6, 2.1 Hz, 1H), 5.94 (s, 2H), 5.13 (s, 2H), 4.65 (d, *J* = 14.9 Hz, 1H), 4.59 (d, *J* = 14.9 Hz, 1H), 4.07 (dq, *J* = 10.7, 7.1 Hz, 1H), 3.88 (dq, *J* = 10.7, 7.1 Hz, 1H), 3.75 (s, 3H), 3.57 (s, 6H), 1.10 (t, *J* = 7.1 Hz, 3H).

^{13}C NMR (101 MHz, Chloroform-*d*) δ 193.4, 167.2, 167.1, 165.3, 162.9 (d, *J* = 246.7 Hz), 161.4, 159.7, 134.4, 132.2 (d, *J* = 3.3 Hz), 129.6 (d, *J* = 8.1 Hz), 127.8, 127.7, 127.7, 127.5, 115.8 (d, *J* = 21.3 Hz), 111.5, 107.6, 105.6, 94.0, 90.2, 80.3, 69.7, 61.6, 55.5, 55.3, 37.1, 14.1.

^{19}F NMR (377 MHz, Chloroform-*d*) δ -113.6.

HRMS (ESI) m/z: $[M + Na]^+$ Calcd for $C_{34}H_{32}FNNaO_7^+$ 608.2055; Found 608.2055.

$[\alpha]_D^{23}$ = -133.5 (*c* 1.0, $CHCl_3$).

Chiral HPLC: Chiralpak AD-H, hexane:*i*PrOH = 70:30, 1.0 mL/min, 254 nm; tR = 7.8 min (minor), 9.9 min (major).

Ethyl (R)-3-oxo-6-phenoxy-2-phenyl-1-(2,4,6-trimethoxybenzyl)indoline-2-carboxylate (3ai)

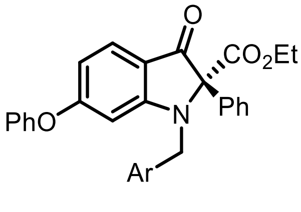

Ar = 2,4,6-$(OMe)_3C_6H_2$

60 °C, 48 h. 22.7 mg, yellow oil, 72% yield, 83% *ee*.

^{1}H NMR (400 MHz, Chloroform-*d*) δ 7.47 – 7.40 (m, 5H), 7.25 – 7.21 (m, 1H), 7.20 – 7.16 (m, 5H), 6.81 (d, *J* = 2.0 Hz, 1H), 6.37 (dd, *J* = 8.5, 2.0 Hz, 1H), 5.87 (s, 2H), 4.55 (d, *J* = 14.9 Hz, 1H), 4.49 (d, *J* = 14.9 Hz, 1H), 4.08 (dq, *J* = 10.7, 7.1 Hz, 1H), 3.90 (dq, *J* = 10.8, 7.1 Hz, 1H), 3.73 (s, 3H), 3.44 (s, 6H), 1.10 (t, *J* = 7.1 Hz, 3H).

^{13}C NMR (101 MHz, Chloroform-*d*) δ 193.6, 167.0, 166.7, 165.0, 161.3, 159.6, 155.4, 134.3, 130.2, 127.8, 127.74, 127.70, 127.6, 124.9, 120.9, 112.5, 108.9, 105.5, 97.5, 90.0, 80.4, 61.7, 55.4, 55.1, 37.2, 14.0.

HRMS (ESI) m/z: $[M + Na]^+$ Calcd for $C_{30}H_{33}NNaO_7^+$ 576.1993; Found 576.1995.

$[\alpha]_D^{23}$ = -133.9 (*c* 1.0, $CHCl_3$).

Chiral HPLC: Chiralpak AD-H, hexane:*i*PrOH = 70:30, 1.0 mL/min, 254 nm; tR = 5.7 min (minor), 7.2 min (major).

Ethyl (*R*)-6-((tert-butyldimethylsilyl)oxy)-3-oxo-2-phenyl-1-(2,4,6-trimethoxybenzyl)indoline-2-carboxylate (3aj)

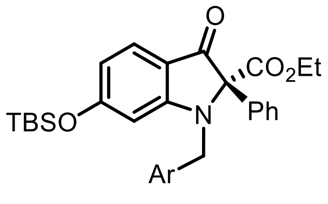

80 °C, 16 h. 20.8 mg, yellow oil, 70% yield, 82% *ee*.

^{1}H NMR (400 MHz, Chloroform-*d*) δ 7.47 – 7.43 (m, 2H), 7.40 (d, *J* = 8.4 Hz, 1H), 7.19 – 7.15 (m, 3H), 6.69 (d, *J* = 1.9 Hz, 1H), 6.23 (dd, *J* = 8.4, 2.0 Hz, 1H), 5.93 (s, 2H), 4.62 (d, *J* = 14.8 Hz, 1H), 4.56 (d, *J* = 14.8 Hz, 1H), 4.07 (dq, *J* = 10.8, 7.1 Hz, 1H), 3.90 (dq, *J* = 10.8, 7.1 Hz, 1H), 3.75 (s, 3H), 3.58 (s, 6H), 1.10 (t, *J* = 7.1 Hz, 3H), 1.03 (s, 9H), 0.30 (s, 6H).

^{13}C NMR (101 MHz, Chloroform-*d*) δ 193.6, 167.2, 165.3, 164.8, 161.3, 159.7, 134.5, 127.77, 127.75, 127.6, 127.3, 111.8, 111.5, 105.6, 100.4, 90.1, 80.2, 61.6, 55.5, 55.3, 37.0, 25.7, 18.3, 14.0, -4.1.

HRMS (ESI) m/z: $[M + Na]^+$ Calcd for $C_{33}H_{41}NNaO_7Si^+$ 614.2545; Found 614.2547.

$[\alpha]_D^{23}$ = -159.7 (*c* 1.0, $CHCl_3$).

Chiral HPLC: Chiralpak AD-H, hexane:*i*PrOH = 90:10, 1.0 mL/min, 254 nm; tR = 7.2 min (minor), 9.0 min (major).

Ethyl (*R*)-5,6-dimethoxy-3-oxo-2-phenyl-1-(2,4,6-trimethoxybenzyl)indoline-2-carboxylate (3ak)

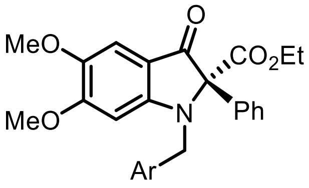

Ar = 2,4,6-$(OMe)_3C_6H_2$

60 °C, 16 h. 24.1 mg, yellow oil, 92% yield, 80% *ee*.

^{1}H NMR (400 MHz, Chloroform-*d*) δ 7.48 – 7.42 (m, 2H), 7.22 – 7.15 (m, 3H), 6.92 (s, 1H), 6.85 (s, 1H), 5.96 (s, 2H), 4.64 (d, *J* = 14.9 Hz, 1H), 4.56 (d, *J* = 14.9 Hz, 1H), 4.11 (dq, *J* = 10.8, 7.1 Hz, 1H), 4.01 (s, 3H), 3.94 (dq, *J* = 10.7, 7.1 Hz, 1H), 3.80 (s, 3H), 3.75 (s, 3H), 3.59 (s, 6H), 1.12 (t, *J* = 7.1 Hz, 3H).

^{13}C NMR (101 MHz, Chloroform-*d*) δ 193.6, 167.3, 161.3, 159.6, 158.7, 143.2, 134.7, 127.83, 127.79, 127.6, 108.6, 105.9, 105.3, 92.8, 90.2, 80.3, 61.6, 56.3, 56.2, 55.5, 55.4, 37.3, 14.1.

HRMS (ESI) m/z: $[M + Na]^+$ Calcd for $C_{29}H_{31}NNaO_8^+$ 544.1942; Found 544.1954.

$[\alpha]_D^{23}$ = -136.8 (*c* 1.0, $CHCl_3$).

Chiral HPLC: Chiralpak IC, hexane:*i*PrOH = 70:30, 1.0 mL/min, 254 nm; tR = 33.9 min (minor), 39.6 min (major).

Ethyl (*R*)-6-(benzyloxy)-5-methyl-3-oxo-2-phenyl-1-(2,4,6-trimethoxybenzyl)indoline-2-carboxylate (3al)

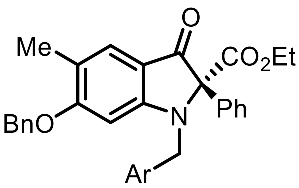

Ar = 2,4,6-$(OMe)_3C_6H_2$

60 °C, 16 h. 25.9 mg, yellow solid, 89% yield, 87% *ee*, m.p. = 120 – 122 °C.

^{1}H NMR (400 MHz, Chloroform-*d*) δ 7.53 – 7.43 (m, 6H), 7.41 – 7.36 (m, 1H), 7.30 (d, *J* = 1.1 Hz, 1H), 7.22 – 7.15 (m, 3H), 6.85 (s, 1H), 5.95 (s, 2H), 5.20 (s, 2H), 4.66 (d, *J* = 14.8 Hz, 1H), 4.59 (d, *J* = 14.9 Hz, 1H), 4.10 (dq, *J* = 10.7, 7.1 Hz, 1H), 3.93 (dq, *J* = 10.7, 7.1 Hz, 1H), 3.76 (s, 3H), 3.56 (s, 6H), 2.19 – 2.14 (m, 3H), 1.12 (t, *J* =

7.1 Hz, 3H).

^{13}C NMR (101 MHz, Chloroform-*d*) δ 193.3, 167.3, 165.7, 164.6, 161.3, 159.7, 136.5, 134.7, 128.8, 128.3, 127.8, 127.5, 127.3, 126.6, 118.5, 110.1, 105.8, 92.0, 90.1, 80.2, 70.1, 61.6, 55.5, 55.4, 37.2, 16.1, 14.1.

HRMS (ESI) m/z: $[M + Na]^+$ Calcd for $C_{35}H_{35}NNaO_7^+$ 604.2306; Found 604.2309.

$[\alpha]_D^{23}$ = -143.2 (*c* 1.0, $CHCl_3$).

Chiral HPLC: Chiralpak AD-H, hexane:*i*PrOH = 70:30, 1.0 mL/min, 254 nm; tR = 6.8 min (major), 8.6 min (minor).

Ethyl (*R*)-6-(benzyloxy)-5-chloro-3-oxo-2-phenyl-1-(2,4,6-trimethoxybenzyl)indoline-2-carboxylate (3am)

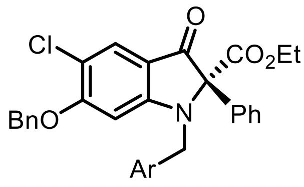

Ar = 2,4,6-$(OMe)_3C_6H_2$

60 °C, 16 h. 24.1 mg, yellow solid, 80% yield, 82% *ee*, m.p. = 57 – 59 °C.

^{1}H NMR (400 MHz, Chloroform-*d*) δ 7.56 – 7.49 (m, 3H), 7.49 – 7.34 (m, 5H), 7.21 (dt, *J* = 4.6, 1.9 Hz, 3H), 6.90 (s, 1H), 5.95 (s, 2H), 5.25 (s, 2H), 4.64 (d, *J* = 14.9 Hz, 1H), 4.57 (d, *J* = 14.9 Hz, 1H), 4.08 (dq, *J* = 10.8, 7.1 Hz, 1H), 3.88 (dq, *J* = 10.8, 7.1 Hz, 1H), 3.76 (s, 3H), 3.55 (s, 6H), 1.11 (t, *J* = 7.1 Hz, 3H).

^{13}C NMR (101 MHz, Chloroform-*d*) δ 192.5, 166.7, 163.4, 161.7, 161.4, 159.6, 135.7, 133.9, 128.8, 128.4, 127.82, 127.75, 127.6, 127.1, 126.5, 114.1, 110.9, 105.1, 93.7, 90.1, 80.0, 70.8, 61.7, 55.4, 55.3, 37.1, 13.9.

HRMS (ESI) m/z: $[M + Na]^+$ Calcd for $C_{34}H_{32}ClNNaO_7^+$ 624.1760; Found 624.1762.

$[\alpha]_D^{23}$ = -103.8 (*c* 0.5, $CHCl_3$).

Chiral HPLC: Chiralpak AD-H, hexane:*i*PrOH = 85:15, 1.0 mL/min, 254 nm; tR = 13.5 min (major), 14.7 min (major).

Ethyl (*R*)-4,6-dimethoxy-3-oxo-2-phenyl-1-(2,4,6-trimethoxybenzyl)indoline-2-carboxylate (3an)

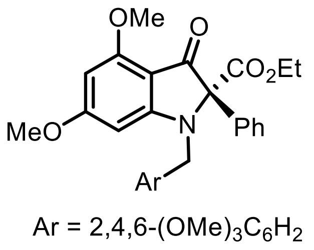

60 °C, 16 h. 17.5 mg, yellow solid, 67% yield, 89% *ee*, m.p. = 172 – 174 °C.

^{1}H NMR (400 MHz, Chloroform-*d*) δ 7.51 – 7.43 (m, 2H), 7.19 – 7.12 (m, 3H), 6.46 (d, *J* = 1.8 Hz, 1H), 5.93 (s, 2H), 5.74 (d, *J* = 1.8 Hz, 1H), 4.64 (d, *J* = 14.9 Hz, 1H), 4.57 (d, *J* = 14.9 Hz, 1H), 4.04 (dq, *J* = 10.7, 7.1 Hz, 1H), 3.93 (s, 3H), 3.87 (dq, *J* = 10.7, 7.1 Hz, 4H), 3.80 (s, 3H), 3.74 (s, 3H), 3.57 (s, 6H), 1.10 (t, *J* = 7.1 Hz, 3H).

^{13}C NMR (101 MHz, Chloroform-*d*) δ 190.6, 169.5, 167.1, 166.0, 161.2, 160.9, 159.6, 134.6, 127.7, 127.5, 127.3, 105.5, 101.0, 90.0, 89.0, 85.8, 61.5, 55.7, 55.6, 55.4, 55.2, 37.1, 14.0.

HRMS (ESI) m/z: $[M + Na]^+$ Calcd for $C_{29}H_{31}NNaO_8^+$ 544.1942; Found 544.1945.

$[\alpha]_D^{23}$ = -93.2 (*c* 1.0, $CHCl_3$).

Chiral HPLC: Chiralpak AD-H, hexane:*i*PrOH = 70:30, 1.0 mL/min, 254 nm; tR = 7.4 min (major), 10.4 min (minor).

Ethyl (*R*)-6-((*tert*-butoxycarbonyl)amino)-3-oxo-2-phenyl-1-(2,4,6-trimethoxybenzyl)indoline-2-carboxylate (3ao)

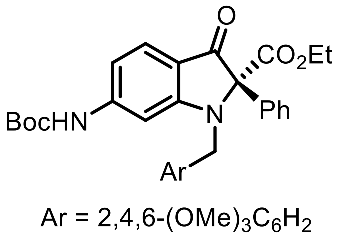

60 °C, 36 h. 19.6 mg, yellow solid, 68% yield, 81% *ee*, m.p. = 177 – 179 °C.

^{1}H NMR (400 MHz, Chloroform-*d*) δ 7.99 – 7.98 (m, 1H), 7.49 – 7.46 (m, 2H), 7.38 (d, *J* = 8.3 Hz, 1H), 7.17 – 7.14 (m, 3H), 6.74 (s, 1H), 6.30 (dd, *J* = 8.4, 1.7 Hz, 1H), 5.93 (s, 2H), 4.69 (d, *J* = 15.0 Hz, 1H), 4.64 (d, *J* = 15.0 Hz, 1H), 4.04 (dq, *J* = 10.8, 7.1 Hz, 1H), 3.84 (dq, *J* = 10.7, 7.1 Hz, 1H), 3.75 (s, 3H), 3.59 (s, 6H), 1.56 (s, 9H),

1.06 (t, J = 7.1 Hz, 3H).

^{13}C NMR (101 MHz, Chloroform-*d*) δ 193.7, 167.0, 164.9, 161.3, 159.8, 152.1, 147.2, 134.3, 127.7, 127.62, 127.57, 126.9, 112.2, 109.0, 105.9, 97.9, 90.0, 81.4, 80.2, 61.6, 55.5, 55.1, 37.1, 28.4, 14.0.

HRMS (ESI) m/z: $[M + Na]^+$ Calcd for $C_{32}H_{36}N_2NaO_8^+$ 599.2364; Found 599.2366.

$[\alpha]_D^{23}$ = -174.2 (*c* 0.5, $CHCl_3$).

Chiral HPLC: Chiralpak IC, hexane:*i*PrOH = 70:30, 1.0 mL/min, 254 nm; tR = 8.0 min (minor), 13.2 min (major).

Ethyl (*R*)-6-(methylthio)-3-oxo-2-phenyl-1-(2,4,6-trimethoxybenzyl)indoline-2-carboxylate (3ap)

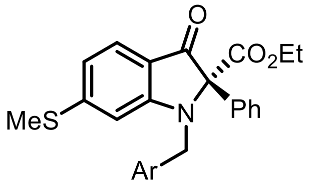

60 °C, 24 h. 20.2 mg, yellow solid, 80% yield, 83% *ee*, m.p. = 155 – 157 °C.

^{1}H NMR (400 MHz, Chloroform-*d*) δ 7.48 – 7.43 (m, 2H), 7.39 (d, J = 8.2 Hz, 1H), 7.21 – 7.17 (m, 3H), 7.13 (d, J = 1.5 Hz, 1H), 6.57 (dd, J = 8.2, 1.5 Hz, 1H), 5.95 (s, 2H), 4.68 – 4.58 (m, 2H), 4.07 (dq, J = 10.8, 7.1 Hz, 1H), 3.86 (dq, J = 10.8, 7.2 Hz, 1H), 3.75 (s, 3H), 3.59 (s, 6H), 2.56 (s, 3H), 1.09 (t, J = 7.1 Hz, 3H).

^{13}C NMR (101 MHz, Chloroform-*d*) δ 194.1, 167.0, 163.0, 161.4, 159.7, 151.4, 134.2, 127.9, 127.7, 125.7, 115.2, 114.4, 105.6, 105.3, 90.1, 80.0, 61.7, 55.5, 55.3, 37.0, 14.8, 14.0.

HRMS (ESI) m/z: $[M + Na]^+$ Calcd for $C_{30}H_{33}NNaO_7^+$ 576.1993; Found 576.1995.

$[\alpha]_D^{23}$ = -191.5 (*c* 0.5, $CHCl_3$).

Chiral HPLC: Chiralpak AD-H, hexane:*i*PrOH = 70:30, 1.0 mL/min, 254 nm; tR = 6.5 min (minor), 9.8 min (major).

Ethyl (R)-1-benzyl-6-methoxy-3-oxo-2-phenylindoline-2-carboxylate (3aq)

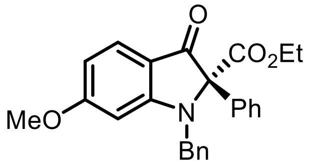

60 °C, 16 h. 17.7 mg, yellow oil, 88% yield, 78% *ee*.

^{1}H NMR (400 MHz, Chloroform-*d*) δ 7.57 (d, *J* = 8.6 Hz, 1H), 7.38 – 7.32 (m, 3H), 7.31 – 7.28 (m, 2H), 7.25 – 7.17 (m, 3H), 7.10 – 7.08 (m, 2H), 6.39 (dd, *J* = 8.6, 2.1 Hz, 1H), 6.04 (d, *J* = 2.1 Hz, 1H), 4.71 (d, *J* = 17.0 Hz, 1H), 4.64 (d, *J* = 16.9 Hz, 1H), 4.14 – 3.99 (m, 2H), 3.71 (s, 3H), 1.14 (t, *J* = 7.1 Hz, 3H).

^{13}C NMR (101 MHz, Chloroform-*d*) δ 192.3, 168.4, 167.9, 163.2, 137.2, 134.9, 129.0, 128.70, 128.66, 127.8, 127.5, 127.3, 126.7, 112.5, 107.9, 93.0, 81.1, 62.5, 55.7, 48.6, 14.0.

HRMS (ESI) m/z: $[M + Na]^+$ Calcd for $C_{25}H_{23}NNaO_4^+$ 424.1519; Found 424.1522.

$[\alpha]_D^{23}$ = -149.2 (*c* 1.0, $CHCl_3$).

Chiral HPLC: Chiralpak AD-H, hexane:*i*PrOH = 70:30, 1.0 mL/min, 254 nm; tR = 6.7 min (minor), 8.9 min (major).

Ethyl (*R*)-6-methoxy-1-(4-methoxybenzyl)-3-oxo-2-phenylindoline-2-carboxylate (3ar)

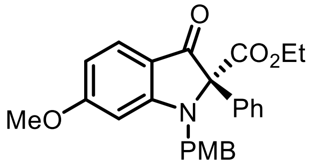

60 °C, 16 h. 20.4 mg, yellow oil, 95% yield, 80% *ee*.

^{1}H NMR (400 MHz, Chloroform-*d*) δ 7.56 (d, *J* = 8.6 Hz, 1H), 7.39 – 7.33 (m, 3H), 7.32 – 7.28 (m, 2H), 7.00 (d, *J* = 8.3 Hz, 2H), 6.77 (d, *J* = 8.7 Hz, 2H), 6.38 (dd, *J* = 8.6, 2.1 Hz, 1H), 6.07 (d, *J* = 2.1 Hz, 1H), 4.66 – 4.55 (m, 2H), 4.17 – 4.02 (m, 2H), 3.75 (s, 3H), 3.73 (s, 3H), 1.17 (t, *J* = 7.1 Hz, 3H).

^{13}C NMR (101 MHz, Chloroform-*d*) δ 192.3, 168.3, 167.9, 163.2, 158.9, 135.0, 129.2, 129.0, 128.7, 128.0, 127.9, 127.5, 114.0, 112.6, 107.8, 93.0, 81.2, 62.5, 55.7, 55.4, 48.1,

14.0.

HRMS (ESI) m/z: $[M + Na]^+$ Calcd for $C_{26}H_{26}NO_5^+$432.1805; Found 432.1804.

$[\alpha]_D^{23}$ = -118.8 (*c* 1.0, $CHCl_3$).

Chiral HPLC: Chiralpak AD-H, hexane:*i*PrOH = 70:30, 1.0 mL/min, 254 nm; tR = 7.8 min (minor), 11.0 min (major).

Ethyl (*R*)-1-(2,4-dimethoxybenzyl)-6-methoxy-3-oxo-2-phenylindoline-2-carboxylate (3as)

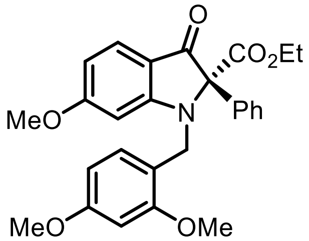

60 °C, 16 h. 19.4 mg, yellow oil, 84% yield, 86% *ee*.

^{1}H NMR (400 MHz, Chloroform-*d*) δ 7.55 (d, *J* = 8.6 Hz, 1H), 7.36 – 7.28 (m, 5H), 6.78 (d, *J* = 8.4 Hz, 1H), 6.41 (d, *J* = 2.4 Hz, 1H), 6.38 (dd, *J* = 8.6, 2.1 Hz, 1H), 6.29 (dd, *J* = 8.4, 2.4 Hz, 1H), 6.13 (d, *J* = 2.1 Hz, 1H), 4.63 (d, *J* = 17.4 Hz, 1H), 4.56 (d, *J* = 17.5 Hz, 1H). 4.12 – 3.98 (m, 2H), 3.78 (s, 3H), 3.77 (s, 3H), 3.75 (s, 3H), 1.12 (t, J = 7.1 Hz, 3H).

^{13}C NMR (101 MHz, Chloroform-*d*) δ 192.5, 168.4, 167.9, 163.6, 160.1, 157.7, 134.8, 128.8, 128.5, 128.1, 127.7, 127.5, 117.1, 112.3, 107.7, 103.8, 98.4, 92.9, 80.9, 62.3, 55.8, 55.5, 55.3, 43.1, 14.0.

HRMS (ESI) m/z: $[M + Na]^+$ Calcd for $C_{27}H_{27}NNaO_6^+$ 484.1731; Found 484.1731.

$[\alpha]_D^{23}$ = -216.3 (*c* 1.0, $CHCl_3$).

Chiral HPLC: Chiralpak AD-H, hexane:*i*PrOH = 70:30, 1.0 mL/min, 254 nm; tR = 8.5 min (minor), 9.8 min (major).

Ethyl (*R*)-6-methoxy-1-methyl-3-oxo-2-phenylindoline-2-carboxylate (3at)

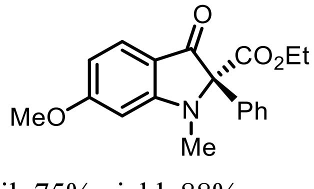

60 °C, 16 h. 12.2 mg, yellow oil, 75% yield, 88% *ee*.

^{1}H NMR (400 MHz, Chloroform-*d*) δ 7.51 (d, *J* = 8.6 Hz, 1H), 7.40 – 7.30 (m, 5H),

6.37 (dd, J = 8.6, 2.1 Hz, 1H), 6.25 (d, J = 2.1 Hz, 1H), 4.36 – 4.21 (m, 2H), 3.91 (s, 3H), 3.02 (s, 3H), 1.27 (t, J = 7.1 Hz, 3H).

^{13}C NMR (101 MHz, Chloroform-*d*) δ 191.9, 168.5, 167.3, 163.4, 134.3, 128.6, 128.4, 127.5, 127.4, 111.9, 107.4, 91.5, 80.6, 62.3, 55.7, 30.1, 14.2.

HRMS (ESI) m/z: $[M + Na]^+$ Calcd for $C_{19}H_{19}NNaO_4^+$ 348.1206; Found 348.1211.

$[\alpha]_D^{23}$ = -193.0 (*c* 0.2, $CHCl_3$).

Chiral HPLC: Chiralpak = OD-H, hexane:*i*PrOH = 70:30, 1.0 mL/min, 254 nm; tR = 5.1 min (minor), 5.7 min (major).

Ethyl (*R*)-6-(benzyloxy)-1-methyl-3-oxo-2-phenylindoline-2-carboxylate (3au)

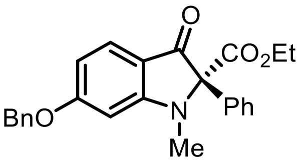

60 °C, 16 h. 18.0 mg, yellow oil, 90% yield, 88% *ee*.

^{1}H NMR (400 MHz, Chloroform-*d*) δ 7.53 (d, J = 8.6 Hz, 1H), 7.47 – 7.32 (m, 10H), 6.45 (dd, J = 8.6, 2.0 Hz, 1H), 6.35 (d, J = 2.1 Hz, 1H), 5.16 (s, 2H), 4.35 – 4.23 (m, 2H), 3.01 (s, 3H), 1.28 (t, J = 7.0 Hz, 3H).

^{13}C NMR (101 MHz, Chloroform-*d*) δ 192.1, 167.8, 167.4, 163.5, 136.1, 134.4, 128.9, 128.8, 128.6, 128.5, 127.8, 127.7, 127.6, 112.3, 107.8, 92.7, 80.7, 70.6, 62.5, 30.2, 14.3.

HRMS (ESI) m/z: $[M + Na]^+$ Calcd for $C_{25}H_{23}NNaO_4^+$ 424.1519; Found 424.1526.

$[\alpha]_D^{23}$ = -93.3 (*c* 1.0, $CHCl_3$).

Chiral HPLC: Chiralpak AD-H, hexane:*i*PrOH = 70:30, 1.0 mL/min, 254 nm; tR = 8.2 min (minor), 16.9 min (major).

Ethyl (*R*)-6-methoxy-1-(4-methoxyphenyl)-3-oxo-2-phenylindoline-2-carboxylate (3av)

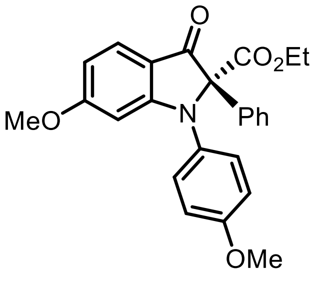

60 °C, 36 h. 19.9 mg, yellow oil, 95% yield, 82% *ee*.

^{1}H NMR (400 MHz, Chloroform-*d*) δ 7.58 (d, *J* = 8.7 Hz, 1H), 7.45 – 7.40 (m, 2H), 7.33 – 7.30 (m, 3H), 7.12 – 7.08 (m, 2H), 6.81 – 6.77 (m, 2H), 6.46 (dd, *J* = 8.7, 2.1 Hz, 1H), 6.34 (d, *J* = 2.1 Hz, 1H), 4.12 (qd, *J* = 7.1, 3.1 Hz, 2H), 3.79 (s, 3H), 3.77 (s, 3H), 1.09 (t, *J* = 7.1 Hz, 3H).

^{13}C NMR (101 MHz, Chloroform-*d*) δ 193.1, 168.2, 167.2, 163.0, 158.1, 135.4, 132.1, 128.6, 128.5, 128.4, 127.44, 127.37, 114.6, 113.1, 109.3, 94.4, 82.8, 62.3, 55.8, 55.5, 14.1.

HRMS (ESI) m/z: $[M + Na]^+$ Calcd for $C_{25}H_{23}NNaO_5^+$ 440.1468; Found 440.1474.

$[\alpha]_D^{23}$ = -10.1 (*c* 1.0, $CHCl_3$).

Chiral HPLC: Chiralpak AD-H, hexane:*i*PrOH = 70:30, 1.0 mL/min, 254 nm; tR = 6.9 min (minor), 7.7 min (major).

Ethyl (*R*)-6-methoxy-7-methyl-3-oxo-2-phenylindoline-2-carboxylate (3aw)

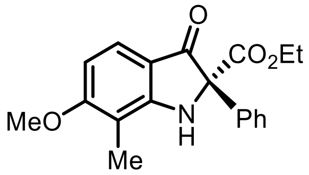

(**1a:2** = 1:1.4), 40 °C, 36 h. 6.8 mg, yellow solid, 42% yield, 86% *ee*, m.p. = 101 – 103 °C.

^{1}H NMR (400 MHz, Chloroform-*d*) δ 8.06 (dq, *J* = 6.5, 2.3 Hz, 2H), 7.50 – 7.38 (m, 3H), 7.11 (d, *J* = 8.0 Hz, 1H), 6.65 (d, *J* = 8.1 Hz, 1H), 4.18 (dq, *J* = 10.7, 7.1 Hz, 2H), 4.03 (dq, *J* = 10.6, 7.1 Hz, 1H), 3.86 (s, 3H), 2.47 (s, 3H), 0.96 (t, *J* = 7.1 Hz, 3H).

^{13}C NMR (101 MHz, Chloroform-*d*) δ 174.6, 172.7, 160.3, 154.7, 131.8, 131.5, 130.0, 128.8, 128.3, 121.3, 119.1, 107.5, 85.5, 63.1, 56.0, 13.9, 10.6.

HRMS (ESI) m/z: $[M + Na]^+$ Calcd for $C_{19}H_{19}NNaO_4^+$ 348.1206; Found 348.1212.

$[\alpha]_D^{23}$ = -5.5 (*c* 0.2, $CHCl_3$).

Chiral HPLC: Chiralpak IG, hexane:*i*PrOH = 80:20, 1.0 mL/min, 254 nm; tR = 8.8 min (major), 9.6 min (minor).

Ethyl (*R*)-7-methoxy-1-oxo-2-phenyl-1,2,5,6-tetrahydro-4*H*-pyrrolo[3,2,1-*ij*]quinoline-2-carboxylate (3ax)

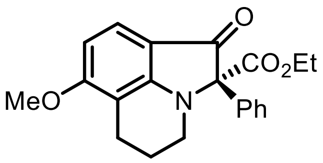

60 °C, 16 h. 16.3 mg, yellow solid, 92% yield, 80% *ee*, m.p. = 93 – 95 °C.

^{1}H NMR (400 MHz, Chloroform-*d*) δ 7.42 (d, *J* = 8.7 Hz, 1H), 7.39 – 7.30 (m, 5H), 6.36 (d, *J* = 8.7 Hz, 1H), 4.34 – 4.22 (m, 2H), 3.89 (s, 3H), 3.37 (ddd, *J* = 11.5, 8.5, 4.1 Hz, 1H), 3.27 (dt, *J* = 11.6, 4.8 Hz, 1H), 2.76 (dt, *J* = 16.8, 5.4 Hz, 1H), 2.65 (ddd, *J* = 16.8, 8.9, 5.6 Hz, 1H), 2.16 – 2.01 (m, 2H), 1.27 (t, *J* = 7.1 Hz, 3H).

^{13}C NMR (101 MHz, Chloroform-*d*) δ 192.6, 167.6, 163.5, 159.8, 134.6, 128.7, 128.5, 127.4, 125.1, 110.6, 106.4, 102.9, 80.1, 62.3, 56.1, 40.9, 21.5, 19.0, 14.3.

HRMS (ESI) m/z: $[M + Na]^+$ Calcd for $C_{21}H_{21}NNaO_4^+$ 374.1363; Found 374.1374.

$[\alpha]_D^{23}$ = -203.5 (*c* 1.0, $CHCl_3$).

Chiral HPLC: Chiralpak OD-H, hexane:*i*PrOH = 70:30, 1.0 mL/min, 254 nm; tR = 5.2 min (minor), 6.1 min (major).

6. Control experiments

6.1 ^{13}C experiment

Procedure for the synthesis of ^{13}C-labelled compound **2a’**

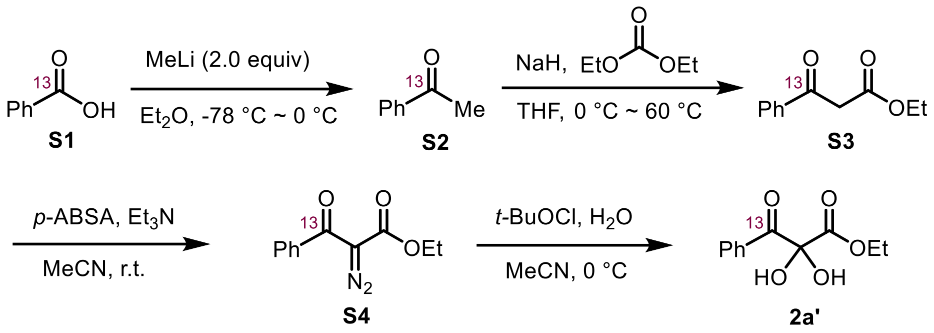

To a dried round-bottom flask under Ar was added the ^{13}C-labelled carboxylic acid **S1** (1.0 g, 8.13 mmol, 1.0 equiv) in Et_2O (10 mL). The round-bottom flask was cooled to 0 °C and 1.6 M solution of MeLi in Et_2O (10.1 mL, 16.2 mmol, 2.0 equiv) was added dropwise via syringe. The reaction was allowed to warm to RT and stirred for 14 hours. At this time the reaction was deemed complete by TLC analysis, and was quenched with saturated NH_4Cl solution at 0 °C. The biphasic mixture was extracted with Et_2O (20mL × 3). The combined organics were then dried over anhydrous $MgSO_4$, filtered, and concentrated in vacuo, the obtained ^{13}C-labelled benzophenone **S2** was directly used for the next step without further purification.

To a dried 100 mL three-necked flask at 0 °C was added NaH (488 mg, 60% w/w, 12.2 mmol, 1.5 equiv), diethyl carbonate (2.93 g, 24.4 mmol, 3.0 equiv), and dry THF (10 mL) under Ar, then the solution of **S2** (from the previous step, 8.13 mmol, 1.0 equiv) in dry THF (10 mL) was added dropwise over 20 minutes and raise the temperature to 60 °C overnight. The reaction was cooled down to room temperature and ice-cold water was slowly added until the solid was dissolved completely. Then, the reaction system was diluted with ethyl acetate. The organic layer was separated, washed with water and then saturated brine and finally dried over anhydrous Na_2SO_4. After the solvent was evaporated with the aid of rotary evaporator, the residue was purified by flash chromatography on silica gel with ethyl acetate/hexanes (1/10) to afford the product **S3** (1.37 g, 87% yield for 2 steps).

A solution of **S3** (1.37 g, 7.1 mmol, 1.0 equiv) and *p*-acetamidobenzenesulfonyl azide (*p*-ABSA) (2.04 g, 8.5 mmol, 1.2 equiv) in 50 mL of MeCN was added triethylamine (1.95 mL, 15.6 mmol, 2.0 equiv) at 0 °C, and the resulting solution was stirred at room temperature for 2 hours and the resulting solution was concentrated under reduced pressure. The residue was purified by flash column chromatography on silica gel with ethyl acetate/hexanes (1/10) to afford the product **S4** (1.44 g, 93% yield).

A solution of **S4** (6.6 mmol, 1.44 g, 1.0 equiv) in 20 mL of solvent (CH_3CN/H_2O = 9/1) at 0 °C was added *tert*-butyl hypochlorite (0.61 ml, 5.5 mmol) dropwise over 30 min via syringe pump. The reaction was stirred for an additional 30 min and then concentrated under reduced pressure. The residue was purified by flash column chromatography on silica gel with ethyl acetate/hexanes (1/5) to afford the ^{13}C-labelled compound **2a'** (0.93 g, 65% yield). The characterization data for **2a'** has been shown in the literature.[7]

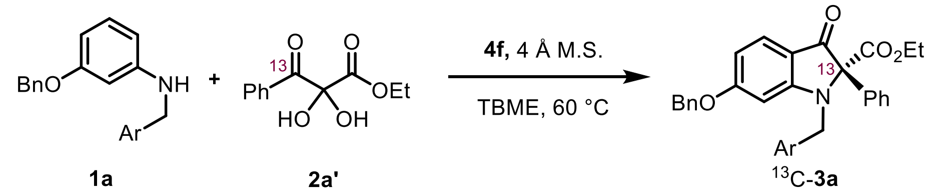

1a Ar = 2,4,6-(OMe)$_3$-C$_6$H$_2$ **2a'** ^{13}C-**3a** 92%, 91% *ee*

A suspension of **1a** (0.05 mmol, 1.0 equiv), **2a'** (0.05 mmol, 1.0 equiv), **4f** (0.005 mmol, 0.1 equiv), 4 Å molecular sieves (15 mg) in TBME (0.5 mL) was stirred in a dry sealed tube under the air. The reaction mixture was stirred at 60 °C for 16 h. After completion of the reaction (monitored by TLC), the solvent was removed under vacuum. The residue was purified by column chromatography on silica gel eluting with petroleum ether/ethyl acetate to afford the product ^{13}C-**3a** as yellow oil with 92% yield and 91% *ee*.

Ethyl (*R*)-6-(benzyloxy)-3-oxo-2-phenyl-1-(2,4,6-trimethoxybenzyl)indoline-2-carboxylate-2-^{13}C (^{13}C-3a)

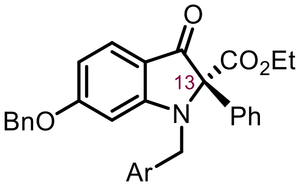

Ar = 2,4,6-$(OMe)_3C_6H_2$

60 °C, 16 h. 26.2 mg, yellow viscous oil, 92% yield, 91% *ee*.

^{1}H NMR (400 MHz, Chloroform-*d*) δ 7.50 – 7.36 (m, 8H), 7.20 – 7.17 (m, 3H), 6.90 (d, *J* = 2.1 Hz, 1H), 6.41 (dd, *J* = 8.6, 2.0 Hz, 1H), 5.94 (s, 2H), 5.18 (s, 2H), 4.68 – 4.57 (m, 2H), 4.08 (dq, *J* = 10.7, 7.1 Hz, 1H), 3.88 (dq, *J* = 10.8, 7.1 Hz, 1H), 3.75 (s, 3H), 3.56 (s, 6H), 1.10 (t, *J* = 7.1 Hz, 3H).

^{13}C NMR (101 MHz, Chloroform-*d*) δ 195.1 – 191.1 (m), 176.4, 167.7 – 165.2 (m), 161.3, 159.7, 136.4, 134.4 (d, *J* = 49.9 Hz), 128.9, 128.5, 127.8, 127.8, 127.7, 127.6, 127.4 (d, *J* = 2.2 Hz), 111.3 (d, *J* = 14.5 Hz), 107.8, 105.6, 94.0, 90.1, 80.2, 70.4, 61.6, 55.5, 55.3, 37.0, 14.0.

HRMS (ESI) m/z: $[M + Na]^+$ Calcd for $C_{33}{}^{13}CH_{33}NNaO_7^+$ 591.2183; Found 591.2196.

$[\alpha]_D^{23}$ = -168.8 (*c* 1.0, $CHCl_3$).

Chiral HPLC: Chiralpak AD-H, hexane:iPrOH = 70:30, 1.0 mL/min, 254 nm; tR = 7.6 min (minor), 9.4 min (major).

6.2 Experiment for survey of nucleophilic site

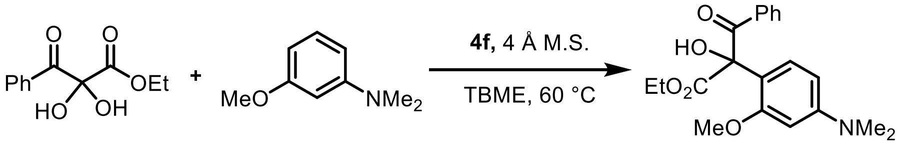

2a **5** **6**

A suspension of **2a** (0.05 mmol, 1.0 equiv), **5** (0.05 mmol, 1.0 equiv), **4f** (0.005 mmol, 0.1 equiv), 4Å molecular sieves (15 mg) in TBME (0.5 mL) was stirred in a dry sealed tube. The reaction mixture was stirred at 60 °C. The product **6** was formed with majority of starting materials remained (monitored by TLC) after 16 h. The solvent was removed under vacuum and residue was purified by column chromatography on silica gel eluting with petroleum ether/ethyl acetate to afford the products **6** as yellow oil.

Ethyl 2-(4-(dimethylamino)-2-methoxyphenyl)-2-hydroxy-3-oxo-3-phenylpropanoate (6)

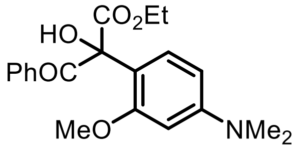

60 °C, 16 h. 3.9 mg, yellow oil, 22% yield, 7% *ee.*

^{1}H NMR (400 MHz, Chloroform-*d*) δ 7.97 – 7.94 (m, 2H), 7.46 – 7.41 (m, 1H), 7.31 – 7.26 (m, 3H), 6.31 (dd, *J* = 8.7, 2.5 Hz, 1H), 6.10 (d, *J* = 2.4 Hz, 1H), 4.96 (s, 1H), 4.35 – 4.19 (m, 2H), 3.45 (s, 3H), 2.95 (s, 6H), 1.22 (t, *J* = 7.1 Hz, 3H).

^{13}C NMR (101 MHz, Chloroform-*d*) δ 196.1, 172.1, 158.1, 152.3, 134.4, 132.7, 130.0, 128.3, 127.9, 116.0, 104.8, 96.7, 83.0, 62.7, 55.3, 40.5, 14.1.

HRMS (ESI) m/z: $[M + Na]^+$ Calcd for $C_{20}H_{23}NNaO_5^+$ 380.1468; Found 380.1477.

Chiral HPLC: Chiralpak IG, hexane:*i*PrOH = 70:30, 1.0 mL/min, 254 nm; tR = 35.9 min (major), 41.0 min (minor).

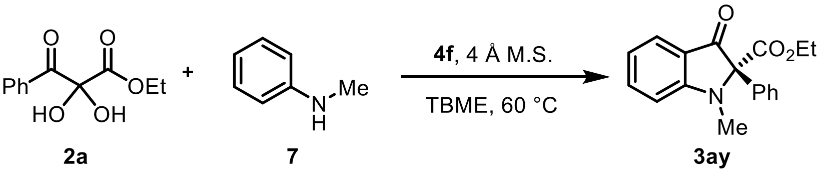

A suspension of **2a** (0.05 mmol, 1.0 equiv), **7** (0.05 mmol, 1.0 equiv), **4f** (0.005 mmol, 0.1 equiv), 4Å molecular sieves (15 mg) in TBME (0.5 mL) was stirred in a dry sealed tube . The reaction mixture was stirred at 60 °C. The product was formed with majority of starting materials remained (monitored by TLC) after 24 h. The solvent was removed under vacuum and residue was purified by column chromatography on silica gel eluting with petroleum ether/ethyl acetate to afford the products **3ay** as yellow oil.

Ethyl (*R*)-1-methyl-3-oxo-2-phenylindoline-2-carboxylate (3ay)

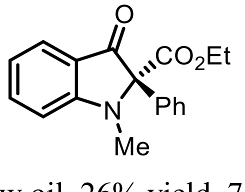

Toluene, 60 °C, 6 d. 3.8 mg, yellow oil, 26% yield, 74% *ee.*

^{1}H NMR (400 MHz, Chloroform-*d*) δ 7.61 – 7.49 (m, 2H), 7.41 – 7.31 (m, 5H), 6.88 (d, *J* = 8.4 Hz, 1H), 6.79 (t, *J* = 7.5 Hz, 1H), 4.35 – 4.21 (m, 2H), 3.05 (s, 3H), 1.26 (t,

J = 7.1 Hz, 4H).

^{13}C NMR (101 MHz, Chloroform-*d*) δ 194.4, 167.3, 161.4, 138.1, 134.0, 128.8, 128.7, 127.5, 126.0, 118.5, 118.3, 108.6, 62.5, 30.2, 14.3.

$[\alpha]_D^{23}$ = -113.2 (*c* 0.5, $CHCl_3$).

HRMS (ESI) m/z: $[M + Na]^+$ Calcd for $C_{18}H_{17}NNaO_3^+$ 318.1101; Found 318.1106.

Chiral HPLC: Chiralpak -, hexane:*i*PrOH = 70:30, 1.0 mL/min, 254 nm; tR = 5.3 min (minor), 6.9 min (major).

6.3 Experiment for non-linear effect

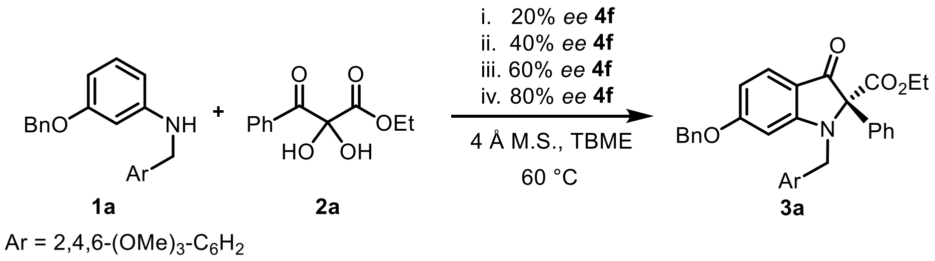

A suspension of **1a** (0.05 mmol, 1.0 equiv), **2a** (0.05 mmol, 1.0 equiv), **4f** (0.005 mmol, 0.1 equiv), 4 Å molecular sieves (15 mg) in TBME (0.5 mL) was stirred in a dry sealed tube. The reaction mixture was stirred at 60 °C for 16 h. After completion of the reaction (monitored by TLC), the solvent was removed under vacuum. The residue was purified by column chromatography on silica gel eluting with petroleum ether/ethyl acetate to afford the product **3a** as yellow oil.

**Entry**	***ee* of 4f [%]**	***ee* of 3a [%]**
1	0	0
2	20	17
3	40	38
4	60	56
5	80	69
6	> 99	91

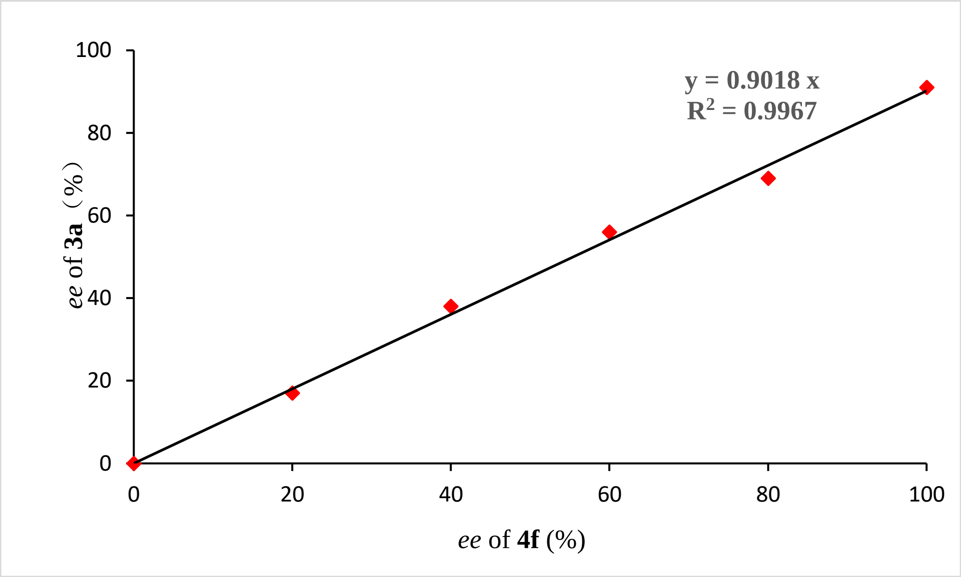

Figure S1. Non-linear effect

7. Scale-up reaction and product transformations

7.1 Scale-up reaction

2 mmol scale reaction of 1a and 2a

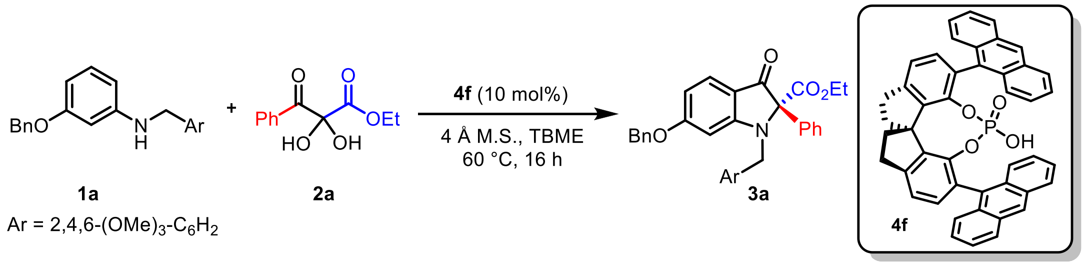

A suspension of **1a** (758 mg, 2.0 mmol, 1.0 equiv), **2a** (448 mg, 2.0 mmol, 1.0 equiv), **4f** (0.2 mmol, 0.1 equiv), 4Å molecular sieves (600 mg) in TBME (20 mL) was stirred in a dry round-bottom flask. The reaction mixture was stirred at 60 °C for 16 h. After completion of the reaction (monitored by TLC), the solvent was removed under vacuum. The residue was purified by column chromatography on silica gel eluting with petroleum ether/ethyl acetate = 8/1 to afford the product **3a** in 90% yield (1.02 g) with 91% *ee* as a yellow solid.

3 mmol scale reaction of 1a and 2ac

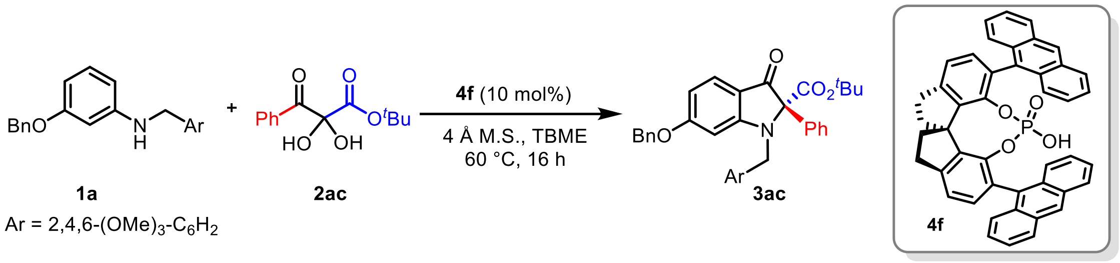

A suspension of **1a** (1.137 g, 3.0 mmol, 1.0 equiv), **2ac** (756 mg, 3.0 mmol, 1.0 equiv), **4f** (200 mg, 0.3 mmol, 0.1 equiv), 4Å molecular sieves (900 mg) in TBME (30 mL) was stirred in a dry round-bottom flask. The reaction mixture was stirred at 60 °C for 16 h. After completion of the reaction (monitored by TLC), the solvent was removed under vacuum. The residue was purified by column chromatography on silica gel eluting with petroleum ether/ethyl acetate = 10/1 to afford the product **3ac** in 94% yield (1.68 g) with 95% *ee* as yellow oil.

7.2 Synthetic transformations

Synthesis of 8

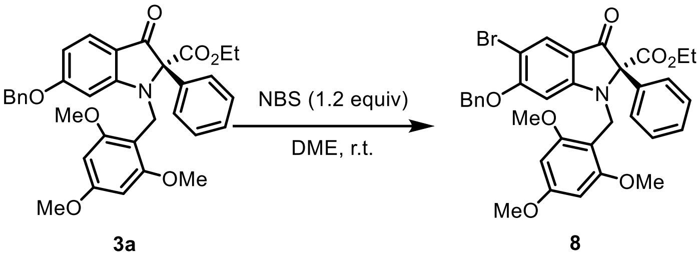

3a (56.7 mg, 0.1 mmol, 1.0 equiv) was dissolved in DME (1.0 mL). The solution was cooled to 0 °C with an ice bath and NBS (0.12 mmol, 21.4 mg, 1.2 equiv) was added. The ice bath was removed and the reaction mixture was stirred at room temperature overnight. After completion of the reaction (monitored by TLC), the mixture was filtered and the filtrate was evaporated under vacuum. The residue was purified by column chromatography on silica gel, eluting with ethyl acetate/petroleum ether to give compound **8** with 88% yield (56.8 mg) as a yellow solid.

Ethyl (*R*)-6-(benzyloxy)-5-bromo-3-oxo-2-phenyl-1-(2,4,6-trimethoxybenzyl)indoline-2-carboxylate (8)

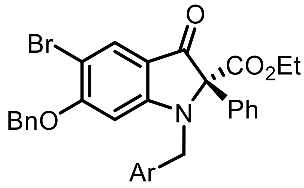

Ar = 2,4,6-$(OMe)_3$-C_6H_2

56.8 mg, yellow solid, 88% yield, m.p. = 135 – 137 °C.

^{1}H NMR (400 MHz, Chloroform-*d*) δ 7.70 (s, 1H), 7.55 – 7.53 (m, 2H), 7.47 – 7.42 (m, 4H), 7.40 – 7.36 (m, 1H), 7.23 – 7.19 (m, 3H), 6.88 (s, 1H), 5.95 (s, 2H), 5.25 (s, 2H), 4.64 (d, *J* = 14.9 Hz, 1H), 4.58 (d, *J* = 14.9 Hz, 1H), 4.08 (dq, *J* = 10.7, 7.1 Hz, 1H), 3.87 (dq, *J* = 10.7, 7.1 Hz, 1H), 3.76 (s, 3H), 3.55 (s, 6H), 1.10 (t, *J* = 7.1 Hz, 3H).

^{13}C NMR (101 MHz, Chloroform-*d*) δ 192.3, 166.8, 164.0, 162.3, 161.5, 159.7, 135.8, 134.0, 129.9, 128.9, 128.4, 128.0, 127.9, 127.7, 127.1, 112.0, 105.1, 102.2, 93.7, 90.2, 80.2, 70.9, 61.8, 55.5, 55.4, 37.2, 14.0.

$[\alpha]_D^{23}$ = -127.0 (*c* 1.0, $CHCl_3$).

HRMS (ESI) m/z: $[M + Na]^+$ Calcd for $C_{34}H_{32}BrNNaO_7^+$ 668.1254; Found 668.1259.

Synthesis of 9

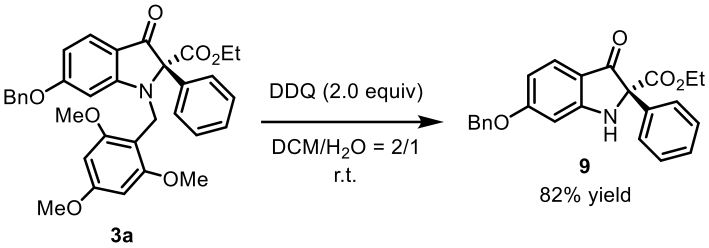

To a stirred solution of **3a** (56.7 mg, 0.1 mmol, 1.0 equiv) in DCM/H_2O = 2/1 (1.5 mL) was added DDQ (45.4 mg, 0.2 mmol, 2.0 equiv), the reaction mixture was stirred at room temperature overnight. After completion of the reaction (monitored by TLC), a saturated solution of NH_4Cl (3 mL) was added to reaction and the mixture was extracted with DCM (5 mL × 3). The organic layers were washed with brine, dried over Na_2SO_4, filtered, and evaporated under vacuum. The residue was purified by column chromatography on silica gel, eluting with ethyl acetate/petroleum ether to give the compound **9** in 82% yield (31.8 mg) as a yellow solid.

Ethyl (*R*)-6-(benzyloxy)-3-oxo-2-phenylindoline-2-carboxylate (9)

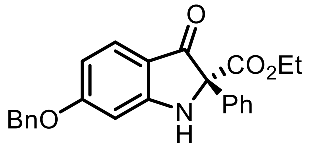

31.8 mg, yellow solid, 82% yield, m.p. = 113 – 115 °C.

^{1}H NMR (400 MHz, Chloroform-*d*) δ 7.77 – 7.74 (m, 2H), 7.52 (d, *J* = 8.5 Hz, 1H), 7.45 – 7.29 (m, 8H), 6.56 – 6.51 (m, 2H), 5.70 (s, 1H), 5.12 (s, 2H), 4.33 – 4.21 (m, 2H), 1.29 (t, *J* = 7.1 Hz, 3H).

^{13}C NMR (101 MHz, Chloroform-*d*) δ 191.3, 168.1, 167.3, 163.1, 136.4, 136.0, 128.9, 128.6, 128.52, 128.50, 127.6, 127.2, 126.4, 113.3, 110.7, 96.9, 70.6, 63.2, 14.1.

$[\alpha]_D^{23}$ = -15.6 (*c* 1.0, $CHCl_3$).

HRMS (ESI) m/z: $[M + Na]^+$ Calcd for $C_{24}H_{21}NNaO_4^+$ 410.1363; Found 410.1376.

Synthesis of 10

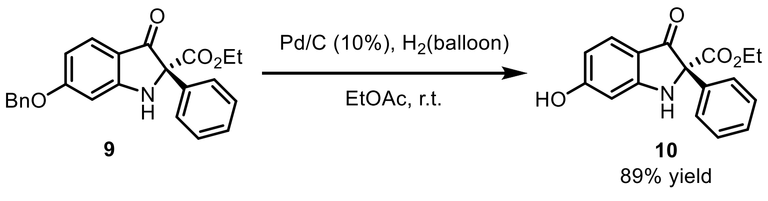

To a stirred solution of **9** (0.1 mmol, 38.7 mg) in ethyl acetate (1.0 mL) was added Pd/C (10 wt%, 4.4 mg). The reaction mixture was stirred and hydrogenated (with H_2 balloon) at room temperature overnight. After completion of the reaction (monitored by TLC), The mixture was filtered with celite and the filtrate was evaporated under vacuum. The residue was purified by column chromatography on silica gel, eluting with ethyl acetate/petroleum ether to give the compound **10** with 89% yield (26.4 mg) as a yellow solid.

Ethyl (*R*)-6-hydroxy-3-oxo-2-phenylindoline-2-carboxylate (10)

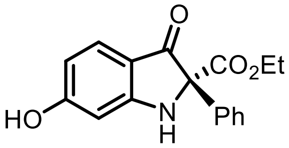

26.4 mg, yellow solid, 89% yield, m.p. = 191 – 193 °C.

^{1}H NMR (400 MHz, Chloroform-*d*) δ 7.73 – 7.70 (m, 2H), 7.45 (d, *J* = 8.9 Hz, 1H), 7.39 – 7.30 (m, 3H), 6.38 – 6.35 (m, 2H), 5.66 (s, 1H), 4.33 – 4.21 (m, 2H), 1.28 (t, *J* = 7.1 Hz, 4H).

^{13}C NMR (101 MHz, Chloroform-*d*) δ 192.1, 168.2, 165.9, 163.4, 136.3, 128.8, 128.6, 127.8, 126.4, 112.6, 110.9, 98.3, 75.7, 63.3, 14.1.

$[\alpha]_D^{23}$ = -1.6 (*c* 0.5, $CHCl_3$).

HRMS (ESI) m/z: $[M + Na]^+$ Calcd for $C_{17}H_{15}NNaO_4^+$ 320.0893; Found 320.0904.

Synthesis of 11

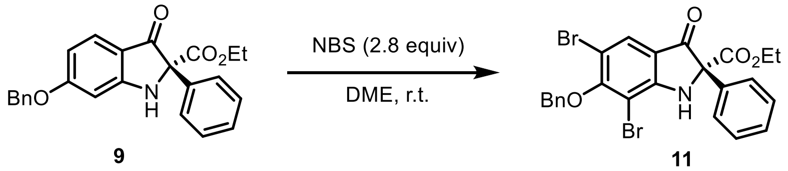

9 (38.7 mg, 0.1 mmol, 1.0 equiv) was dissolved in DME (1.0 mL). The solution was cooled to 0 °C with an ice bath and NBS (49.8 mg, 0.28 mmol, 2.8 equiv) was added. The ice bath was removed and the reaction mixture was stirred at room temperature overnight. After completion of the reaction (monitored by TLC), the mixture was filtered and the filtrate was evaporated under vacuum. The residue was purified by column chromatography on silica gel, eluting with ethyl acetate/petroleum ether to give

the compound **11** in 87% yield (47.4 mg) as a yellow solid.

Ethyl (*R*)-6-(benzyloxy)-5,7-dibromo-3-oxo-2-phenylindoline-2-carboxylate (11)

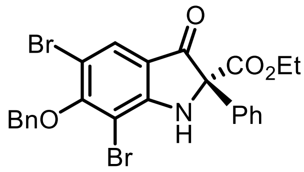

47.4 mg, yellow solid, 87% yield, m.p. = 98 – 100 °C.

^{1}H NMR (400 MHz, Chloroform-*d*) δ 7.84 – 7.74 (m, 3H), 7.66 – 7.59 (m, 2H), 7.48 – 7.34 (m, 6H), 5.93 (s, 1H), 5.12 (s, 2H), 4.30 (m, 2H), 1.31 (t, J = 7.1 Hz, 3H).

^{13}C NMR (101 MHz, Chloroform-*d*) δ 190.9, 166.9, 159.9, 159.1, 135.7, 135.0, 128.97, 128.96, 128.84, 128.83, 128.81, 128.7, 126.2, 117.7, 109.6, 102.7, 76.0, 75.3, 63.5, 14.1.

$[\alpha]_D^{23}$ = -62.6 (*c* 2.0, $CHCl_3$).

HRMS (ESI) m/z: $[M + Na]^+$ Calcd for $C_{24}H_{19}Br_2NNaO_4^+$ 565.9573; Found 565.9581.

Synthesis of 12

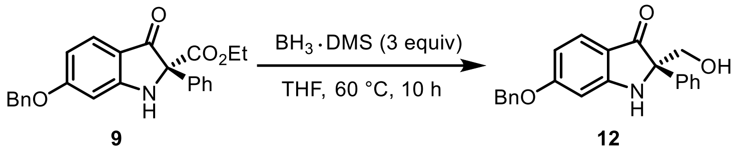

A solution of **9** (19.4 mg, 0.05 mmol) in dry THF (0.5 mL) was treated with borane-dimethyl sulfide complex (67.5 μL, 2 M in THF, 3 equiv) at 0 °C under Ar. The resulting mixture was heated at 60 °C for 10 h and then cooled to 0 °C. The reaction was then quenched with MeOH (1 mL) at 0 °C. The solvent was removed under reduced pressure, and the crude product was purified by silica gel column chromatography (PE/EA = 1/1) to afford **12** in 62% yield (10.7 mg) as yellow oil.

(*S*)-6-(benzyloxy)-2-(hydroxymethyl)-2-phenylindolin-3-one (12)

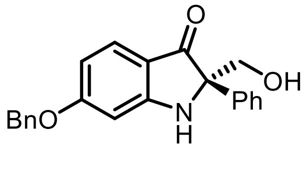

10.7 mg, yellow oil, 62% yield.

^{1}H NMR (400 MHz, Chloroform-*d*) δ 7.64 – 7.59 (m, 2H), 7.50 (d, J = 8.6 Hz, 1H), 7.45 – 7.34 (m, 7H), 7.33 – 7.28 (m, 1H), 6.51 (dd, J = 8.6, 2.1 Hz, 1H), 6.47 (d, J = 2.0 Hz, 1H), 5.35 (brs, 1H), 5.12 (s, 2H), 4.10 (d, J = 11.3 Hz, 1H), 4.02 (d, J = 11.3 Hz, 1H), 2.07 (brs, 1H).

^{13}C NMR (101 MHz, Chloroform-*d*) δ 197.9, 167.3, 163.2, 136.9, 136.1, 129.0, 128.9, 128.5, 128.3, 127.6, 126.9, 126.1, 114.2, 110.1, 96.4, 73.7, 70.5, 68.0.

$[\alpha]_D^{23}$ = -10.4 (*c* 0.5, $CHCl_3$).

HRMS (ESI) m/z: $[M + H]^+$ Calcd for $C_{22}H_{20}NO_3^+$ 346.1438; Found 346.1443.

Synthesis of 13

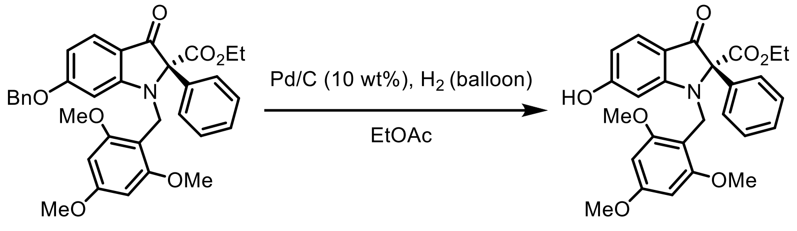

3a **13**
95% yield

To a stirred solution of **3a** (56.7 mg, 0.1 mmol) in ethyl acetate (1.0 mL) was added Pd/C (4.4 mg, 10 wt%). The reaction mixture was stirred and hydrogenated with hydrogen balloon at room temperature overnight. After completion of the reaction (monitored by TLC), The mixture was filtered with diatomite and the filtrate was evaporated under vacuum. The residue was purified by column chromatography on silica gel, eluting with ethyl acetate/petroleum ether to give the compound **13** in 95% yield (45.4 mg) as a yellow solid.

Ethyl (*R*)-6-hydroxy-3-oxo-2-phenyl-1-(2,4,6-trimethoxybenzyl)indoline-2-carboxylate (13)

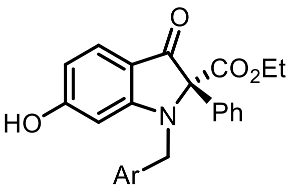

Ar = 2,4,6-$(OMe)_3$-C_6H_2

45.4 mg, yellow solid, 95% yield, m.p. = 182 – 184 °C.

^{1}H NMR (400 MHz, Chloroform-*d*) δ 7.46 – 7.41 (m, 2H), 7.34 (d, *J* = 8.5 Hz, 1H), 7.16 – 7.12 (m, 3H), 6.73 (d, *J* = 2.0 Hz, 1H), 6.17 (dd, *J* = 8.5, 2.0 Hz, 1H), 5.90 (s, 2H), 4.58 (d, *J* = 15.0 Hz, 1H), 4.54 (d, *J* = 14.8 Hz, 1H), 4.02 (dq, *J* = 10.7, 7.1 Hz, 1H), 3.81 (dq, *J* = 10.8, 7.1 Hz, 1H), 3.73 (s, 3H), 3.55 (s, 6H), 1.06 (t, *J* = 7.1 Hz, 3H).

^{13}C NMR (101 MHz, Chloroform-*d*) δ 194.1, 167.2, 166.2, 165.9, 161.3, 159.7, 134.2,

127.82, 127.79, 127.7, 110.5, 108.5, 105.6, 95.5, 90.0, 80.0, 61.7, 55.5, 55.2, 36.8, 14.0.

$[\alpha]_D^{23}$ = -7.8 (*c* 1.0, $CHCl_3$).

HRMS (ESI) m/z: $[M + Na]^+$ Calcd for $C_{27}H_{27}NNaO_7^+$ 500.1680; Found 500.1691.

Synthesis of 14

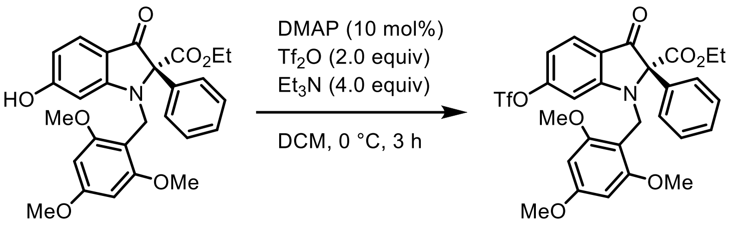

13 **14** 84% yield

To a solution of **13** (47.8 mg, 0.1 mmol, 1.0 equiv), Et_3N (40.4 mg, 0.4 mmol, 4.0 equiv.) and DMAP (1.2 mg, 0.01 mmol, 10 mol%) in anhydrous DCM (2.0 mL) was added Tf_2O (54.2 mg, 0.2 mmol, 2.0 equiv) at 0 °C. The reaction mixture was stirred at room temperature for 3 h. After completion of the reaction (monitored by TLC), a saturated solution of $NaHCO_3$ was added to quench the reaction and the mixture was extracted with DCM (5 mL x 3). The organic layers were washed with water and brine, dried over Na_2SO_4, filtered, and evaporated. The residue was purified by column chromatography on silica gel, eluting with ethyl acetate/petroleum ether to give the compound **14** in 84% yield (51.2 mg) as a yellow solid.

Ethyl (*R*)-3-oxo-2-phenyl-6-(((trifluoromethyl)sulfonyl)oxy)-1-(2,4,6-trimethoxybenzyl)indoline-2-carboxylate (14)

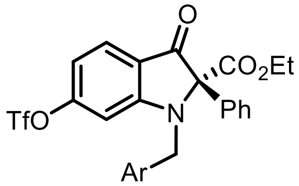

Ar = 2,4,6-$(OMe)_3$-C_6H_2

51.2 mg, yellow solid, 84% yield, m.p. = 85 – 87 °C.

^{1}H NMR (400 MHz, Chloroform-*d*) δ 7.55 (d, *J* = 8.4 Hz, 1H), 7.45 – 7.42 (m, 2H), 7.26 (d, *J* = 2.2 Hz, 1H), 7.24 – 7.22 (m, 3H), 6.58 (dd, *J* = 8.4, 2.1 Hz, 1H), 5.96 (s, 2H), 4.66 (d, *J* = 15.0 Hz, 1H), 4.59 (d, *J* = 15.0 Hz, 1H), 4.07 (dq, *J* = 10.8, 7.1 Hz, 1H), 3.86 (dq, *J* = 10.7, 7.1 Hz, 1H), 3.76 (s, 3H), 3.60 (s, 6H), 1.09 (t, *J* = 7.1 Hz, 3H).

^{13}C NMR (101 MHz, Chloroform-*d*) δ 194.1, 166.3, 163.6, 161.6, 159.7, 156.3, 133.1, 128.2, 128.1, 127.8, 127.6, 118.8 (q, J = 320.6 Hz), 117.1, 110.4, 104.6, 103.5, 90.1, 80.3, 62.0, 55.5, 55.2, 37.5, 14.0.

^{19}F NMR (377 MHz, Chloroform-*d*) δ -73.1.

$[\alpha]_D^{23}$ = -171.1 (*c* 1.0, $CHCl_3$).

HRMS (ESI) m/z: $[M + Na]^+$ Calcd for $C_{28}H_{26}F_3NNaO_9^+$ 632.1173; Found 632.1175.

Synthesis of 15

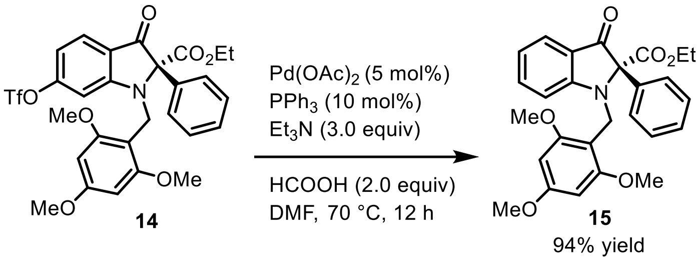

Under N_2, to an oven-dried Schlenk flask were sequentially added $Pd(OAc)_2$ (1.2 mg, 0.005 mmol, 5 mol%), PPh_3 (2.6 mg, 0.01 mmol, 10 mol%), anhydrous DMF (2.0 mL), anhydrous Et_3N (30.3 mg, 0.3 mmol, 3.0 equiv), and HCO_2H (9.2 mg, 0.2 mmol, 2.0 equiv). Next, a solution of **14** (61.0 mg, 0.1 mmol, 1.0 equiv) in anhydrous DMF (2.0 mL) was added. The reaction mixture was stirred at 70 °C for 12 h. After completion of the reaction (monitored by TLC), the reaction was cooled to rt. A saturated aqueous solution of NH_4Cl (5 mL) was added to quench the reaction. The layers were separated, and the aqueous layer was extracted with ethyl acetate (5 mL × 3). The combined organic layers were washed with brine (20 mL), dried over Na_2SO_4, and concentrated. The residue was purified by silica gel flash column chromatography to afford **15** (43.4 mg, 94% yield) as a yellow solid.

Ethyl (*R*)-3-oxo-2-phenyl-1-(2,4,6-trimethoxybenzyl)indoline-2-carboxylate (15)

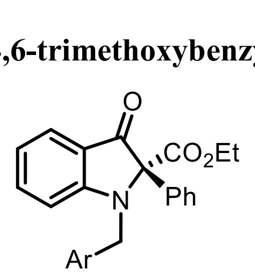

Ar = 2,4,6-$(OMe)_3$-C_6H_2

43.4 mg, yellow solid, 94% yield, m.p. = 85 – 87 °C.

^{1}H NMR (400 MHz, Chloroform-*d*) δ 7.55 – 7.49 (m, 2H), 7.48 – 7.43 (m, 2H), 7.34

(d, J = 8.5 Hz, 1H), 7.20 – 7.16 (m, 3H), 6.72 (ddd, J = 7.8, 7.0, 0.8 Hz, 1H), 5.93 (s, 2H), 4.72 – 4.63 (m, 2H), 4.04 (dq, J = 10.7, 7.1 Hz, 1H), 3.82 (dq, J = 10.7, 7.1 Hz, 1H), 3.75 (s, 3H), 3.57 (s, 6H), 1.07 (t, J = 7.1 Hz, 3H).

^{13}C NMR (101 MHz, Chloroform-*d*) δ 195.7, 167.0, 163.3, 161.3, 159.8, 137.5, 133.9, 127.8, 127.72, 127.69, 125.9, 117.5, 117.2, 110.2, 105.7, 90.1, 79.4, 61.6, 55.5, 55.2, 36.8, 14.0.

$[\alpha]_D^{23}$ = -209.2 (*c* 2.0, $CHCl_3$).

HRMS (ESI) m/z: $[M + Na]^+$ Calcd for $C_{27}H_{27}NNaO_6^+$ 484.1731; Found 484.1732.

Synthesis of 16

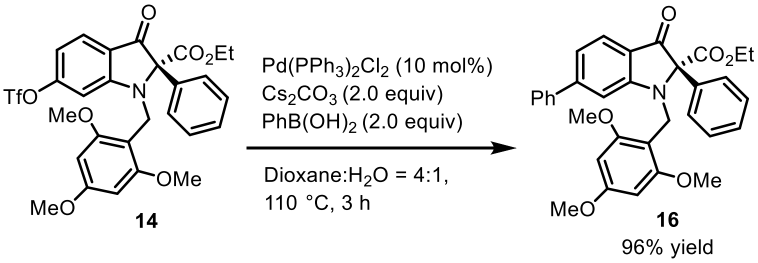

$Pd(PPh_3)_2Cl_2$ (7.0 mg, 0.01 mmol, 10 mol%), Cs_2CO_3 (65.2 mg, 0.2 mmol, 2.0 equiv) and $PhB(OH)_2$ (24.4 mg, 0.2 mmol, 2.0 equiv) were added to a solution of **14** (61.0 mg, 0.1 mmol, 1.0 equiv) in 1,4-dioxane/H_2O (V/V = 4/1, 2.0 mL). The reaction mixture was stirred under reflux for 3 h. After completion of the reaction (monitored by TLC), the reaction was quenched with saturated aqueous NH_4Cl (5 mL), the resulting mixture was extracted with EtOAc (5 mL x 3). The combined organic layers were washed with water and brine, dried over Na_2SO_4, filtered, and evaporated. The residue was purified by column chromatography on silica gel, eluting with ethyl acetate/petroleum ether to give the compound **16** in 96% yield (51.6 mg) as yellow oil.

Ethyl (*R*)-3-oxo-2,6-diphenyl-1-(2,4,6-trimethoxybenzyl)indoline-2-carboxylate (16)

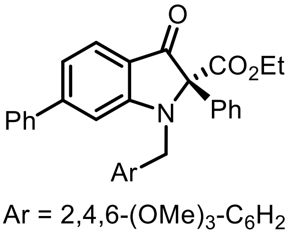

Ar = 2,4,6-(OMe)$_3$-C_6H_2

51.6 mg, yellow oil, 96% yield.

^{1}H NMR (400 MHz, Chloroform-*d*) δ 7.73 – 7.70 (m, 2H), 7.60 – 7.56 (m, 2H), 7.52 – 7.48 (m, 4H), 7.45 – 7.41 (m, 1H), 7.22 – 7.19 (m, 3H), 6.99 (dd, *J* = 8.0, 1.4 Hz, 1H), 5.96 (s, 2H), 4.77 – 4.67 (m, 2H), 4.09 (dq, *J* = 10.7, 7.1 Hz, 1H), 3.89 (dq, *J* = 10.7, 7.2 Hz, 1H), 3.76 (s, 3H), 3.57 (s, 6H), 1.11 (t, *J* = 7.1 Hz, 3H).

^{13}C NMR (101 MHz, Chloroform-*d*) δ 195.3, 167.1, 163.6, 161.3, 159.7, 150.5, 141.2, 134.2, 129.0, 128.5, 127.9, 127.8, 127.5, 126.1, 117.5, 116.4, 108.8, 105.7, 90.1, 80.2, 61.7, 55.5, 55.3, 37.1, 14.0.

$[\alpha]_D^{23}$ = -220.8 (*c* 2.0, $CHCl_3$).

HRMS (ESI) m/z: $[M + Na]^+$ Calcd for $C_{33}H_{32}NO_6^+$ 538.2224; Found 538.2223.

8. References

[1] P. A. Peixoto, A. Boulangé, S. Leleu, X. Franck, *Eur. J. Org. Chem.* **2013**, 3316.

[2] M. G. Ferlin, G. Chiarelotto, V. Gasparotto, L. Dalla Via, V. Pezzi, L. Barzon, G. Palù, I. Castagliuolo, *J. Med. Chem.* **2005**, 48, 3417.

[3] P. M. Truong, P. Y. Zavalij, M. P. Doyle, *Angew. Chem. Int. Ed*. **2014**, 53, 6468.

[4] I. W. Lin, C. N. Lok, K. Yan, C. M. Che, *Chem. Commun.* **2013**, 49, 3297.

[5] F. A. Cotton, C. Y. Liu, C. A. Murillo, D. Villagrán, X. Wang, *J. Am. Chem. Soc.* **2003**, 125, 13564.

[6] Y.-P. He, R. Quan, X.-Z. Li, J. Zhu, H. Wu, *Angew. Chem. Int. Ed.* **2023**, 62, e202217954.

[7] D. Tian, Z.-C. Li, Z.-H. Sun, Y.-P. He, L.-P. Xu, H. Wu, *Angew. Chem. Int. Ed.* **2023**, e202313797.

9. Crystallographic data for 3a (CCDC 2290415)

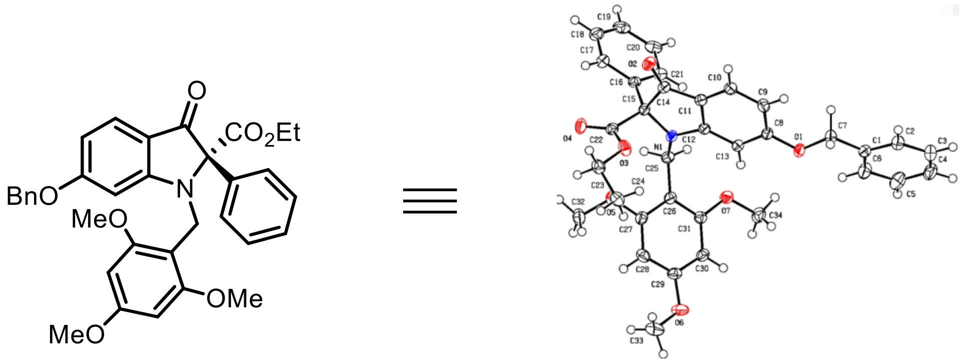

Bond precision: C-C = 0.0027 A Wavelength=1.54178

Cell:	a=12.1135(4)	b=8.4828(3)	c=14.4305(5)
	alpha=90	beta=107.090(1)	gamma=90
Temperature:	173 K		

	Calculated	Reported
Volume	1417.35(8)	1417.35(8)
Space group	P 21	P 1 21 1
Hall group	P 2yb	P 2yb
Moiety formula	C34 H33 N O7	C34 H33 N O7
Sum formula	C34 H33 N O7	C34 H33 N O7
Mr	567.61	567.61
Dx,g cm-3	1.330	1.330
Z	2	2
Mu (mm-1)	0.759	0.759
F000	600.0	600.0
F000’	601.91	
h,k,lmax	14,10,17	14,9,17
Nref	5008[2690]	4840
Tmin,Tmax	0.853,0.886	0.669,0.754
Tmin’	0.853	

Correction method= # Reported T Limits: Tmin=0.669 Tmax=0.754
AbsCorr = MULTI-SCAN

Data completeness= 1.80/0.97 Theta(max)= 66.558

R(reflections)= 0.0274(4782) wR2(reflections)= 0.0728(4840)

S = 1.055 Npar= 384

Table 1 Crystal data and structure refinement for b_a.

Identification code	b_a
Empirical formula	$C_{34}H_{33}NO_7$
Formula weight	567.61
Temperature/K	173.00
Crystal system	monoclinic
Space group	$P2_1$
a/Å	12.1135(4)
b/Å	8.4828(3)
c/Å	14.4305(5)
α/°	90
β/°	107.0900(10)
γ/°	90
Volume/Å^3	1417.35(8)
Z	2
ρ_{calc}g/cm^3	1.330
μ/mm^{-1}	0.759
F(000)	600.0
Crystal size/mm^3	0.21 × 0.2 × 0.16
Radiation	CuKα (λ = 1.54178)
2Θ range for data collection/°	6.408 to 133.116
Index ranges	$-14 \leq h \leq 14$, $-9 \leq k \leq 9$, $-17 \leq l \leq 17$
Reflections collected	53980
Independent reflections	4840 [R_{int} = 0.0329, R_{sigma} = 0.0182]
Data/restraints/parameters	4840/1/384
Goodness-of-fit on F^2	1.055
Final R indexes [I>=2σ (I)]	R_1 = 0.0274, wR_2 = 0.0726
Final R indexes [all data]	R_1 = 0.0281, wR_2 = 0.0728

Largest diff. peak/hole / e Å^{-3} 0.17/-0.15

Flack parameter -0.03(9)

Table 2 Fractional Atomic Coordinates (×10^4) and Equivalent Isotropic Displacement Parameters (Å^2×10^3) for b_a. U_{eq} is defined as 1/3 of the trace of the orthogonalised U_{IJ} tensor.

Atom	x	y	z	U(eq)
O1	8024.9(11)	7499.2(17)	6586.8(9)	24.9(3)
N1	6613.2(12)	5271.1(18)	3384.7(10)	18.1(3)
C1	8801.3(16)	8041(2)	8294.0(13)	24.7(4)
O2	8940.0(11)	6135.3(16)	2435.6(9)	25.0(3)
C2	9597.6(17)	8783(3)	9065.8(14)	32.0(5)
O3	6308.6(12)	7374.7(16)	1851.4(9)	27.4(3)
C3	9546.9(19)	8589(3)	10007.2(15)	38.4(5)
O4	6009.8(13)	5248(2)	883.2(9)	33.5(3)
C4	8699(2)	7659(3)	10188.6(14)	38.2(5)
O5	4124.0(11)	5686.0(18)	1877.6(9)	30.2(3)
C5	7889(2)	6944(3)	9424.6(16)	38.8(5)
O6	1738.8(11)	7708.9(19)	3778.9(10)	32.9(3)
C6	7932.6(19)	7138(3)	8474.9(15)	32.0(4)
O7	4991.9(11)	4475.5(17)	5165.9(9)	26.7(3)
C7	8966.6(16)	8216(2)	7304.8(13)	25.3(4)
C8	8167.3(15)	7228(2)	5700.5(13)	21.3(4)
C9	9160.8(16)	7719(2)	5455.5(13)	23.6(4)
C10	9275.1(15)	7320(2)	4563.7(13)	22.7(4)
C11	8390.9(15)	6497(2)	3905.4(12)	19.6(4)
C12	7382.6(14)	6046(2)	4142.5(12)	18.2(3)
C13	7272.7(15)	6403(2)	5055.3(12)	20.3(4)
C14	8293.2(14)	5969(2)	2938.9(12)	19.0(3)

C15	7131.7(14)	5032(2)	2593.7(12)	19.0(4)
C16	7472.8(14)	3333(2)	2484.4(13)	19.8(4)
C17	7679.7(16)	2781(2)	1639.5(13)	25.5(4)
C18	8091.6(16)	1263(2)	1600.5(15)	30.7(5)
C19	8299.7(16)	277(2)	2400.5(16)	30.7(4)
C20	8129.5(16)	826(2)	3247.4(16)	29.2(4)
C21	7733.5(15)	2352(2)	3294.5(14)	23.7(4)
C22	6398.3(15)	5855(2)	1662.2(13)	21.1(4)
C23	5698.5(17)	8385(2)	1048.0(13)	27.2(4)
C24	5204.3(18)	9740(3)	1463.1(15)	32.3(5)
C25	5640.2(14)	4303(2)	3475.5(13)	20.9(4)
C26	4588.0(14)	5201(2)	3535.3(13)	20.4(4)
C27	3833.5(15)	5891(2)	2715.5(13)	22.5(4)
C28	2860.0(16)	6741(2)	2752.5(14)	25.7(4)
C29	2634.0(15)	6858(2)	3639.4(14)	24.2(4)
C30	3311.3(15)	6091(2)	4458.2(13)	22.9(4)
C31	4281.3(15)	5271(2)	4400.6(12)	20.9(4)
C32	3275.8(17)	6046(3)	988.0(13)	33.4(5)
C33	1122.8(18)	8703(3)	3005.5(17)	34.5(5)
C34	4762.2(19)	4562(3)	6077.2(14)	31.1(4)

Table 3 Anisotropic Displacement Parameters (Å^2×10^3) for b_a. The Anisotropic displacement factor exponent takes the form: -2π^2[h^{2}a*^{2}U$_{11}$+2hka*b*U$_{12}$+…].

Atom	U11	U22	U33	U23	U13	U12
O1	23.5(6)	34.6(8)	17.4(6)	-4.7(5)	7.4(5)	-2.2(5)
N1	18.9(7)	20.5(7)	16.4(7)	-0.4(6)	7.7(5)	-0.4(6)
C1	25.5(9)	27.6(11)	21.5(9)	-1.6(7)	7.6(7)	5.7(7)
O2	26.8(6)	29.0(7)	23.2(6)	-0.6(5)	13.8(5)	-2.6(6)
C2	27.9(10)	42.9(13)	24.4(9)	-6.8(8)	6.7(8)	0.1(9)

O3	33.3(7)	21.3(7)	22.2(6)	4.2(5)	-0.3(5)	1.5(5)
C3	35.7(11)	54.4(14)	21.8(9)	-7.3(10)	3.2(8)	5.5(10)
O4	42.1(8)	38.0(8)	17.2(6)	-4.3(6)	3.7(6)	11.8(6)
C4	49.5(13)	47.3(13)	21.0(9)	2.4(9)	15.0(9)	11.9(11)
O5	25.2(6)	46.8(10)	18.9(6)	-1.5(6)	7.1(5)	0.3(6)
C5	49.4(13)	40.9(13)	32.7(11)	0.0(10)	22.1(10)	-2.2(10)
O6	24.4(7)	36.5(8)	41.3(8)	4.9(7)	14.9(6)	10.6(6)
C6	36.9(11)	35.1(12)	25.3(10)	-6.5(8)	11.2(8)	-4.9(9)
O7	26.8(6)	34.2(8)	21.7(6)	5.2(6)	10.9(5)	6.4(6)
C7	24.3(9)	31.1(11)	19.3(9)	-4.3(7)	4.5(7)	-0.4(7)
C8	24.3(9)	22.8(9)	17.3(8)	1.0(7)	6.7(7)	5.0(7)
C9	21.7(9)	26.7(10)	21.1(8)	-0.3(7)	4.0(7)	-2.6(7)
C10	21.1(8)	26.9(10)	21.7(9)	2.9(7)	8.5(7)	-0.2(7)
C11	20.6(8)	19.1(9)	20.1(8)	3.4(7)	7.7(7)	2.5(6)
C12	19.7(8)	17.4(8)	17.8(8)	2.2(7)	5.9(7)	2.8(7)
C13	19.8(8)	24.1(9)	18.4(8)	0.0(7)	7.6(7)	1.3(7)
C14	22.5(8)	16.0(8)	19.9(8)	2.4(7)	8.3(7)	2.2(7)
C15	20.6(8)	21.0(9)	16.7(8)	0.1(7)	7.5(7)	1.1(7)
C16	16.1(8)	20.8(9)	23.5(8)	-2.3(7)	7.4(6)	-1.1(6)
C17	26.4(9)	27.1(10)	23.6(9)	-3.7(8)	8.3(7)	1.7(8)
C18	25.7(9)	32.1(12)	34.0(10)	-11.6(9)	8.3(8)	1.7(8)
C19	21.0(9)	21.1(10)	47.7(12)	-5.9(9)	6.6(8)	2.3(8)
C20	23.4(9)	24.2(10)	39.9(11)	5.8(8)	9.0(8)	4.1(8)
C21	21.5(9)	23.5(10)	27.3(9)	1.3(7)	9.0(7)	2.4(7)
C22	21.1(8)	23.5(9)	20.8(9)	0.9(7)	9.5(7)	0.9(7)
C23	29.5(10)	27.9(10)	22.3(9)	9.1(8)	4.7(7)	4.7(8)
C24	31.1(10)	35.2(12)	27.7(10)	5.1(8)	4.3(8)	8.4(8)
C25	20.9(9)	19.7(9)	23.7(9)	-1.3(7)	9.0(7)	-1.3(7)
C26	19.6(8)	19.9(9)	22.6(8)	-4.1(7)	7.5(7)	-2.9(7)

C27	21.8(8)	25.9(9)	21.2(9)	-3.8(7)	8.3(7)	-4.8(7)
C28	19.3(9)	30.0(10)	26.5(9)	2.7(8)	4.4(7)	-1.1(7)
C29	17.7(8)	24.6(10)	31.4(10)	-2.7(8)	9.1(7)	-0.5(7)
C30	21.7(8)	25.8(10)	23.6(9)	-3.9(7)	10.4(7)	-3.0(7)
C31	20.0(8)	20.6(9)	22.1(8)	-0.5(7)	6.0(7)	-1.0(7)
C32	30.8(10)	47.8(13)	19.9(9)	0.0(9)	4.8(8)	-5.5(10)
C33	26.1(10)	30.2(11)	47.9(12)	8.2(9)	12.0(9)	5.9(8)
C34	39.5(11)	33.8(11)	22.5(9)	4.5(8)	12.8(8)	5.8(9)

Table 4 Bond Lengths for b_a.

Atom	Atom	Length/Å
O1	C7	1.432(2)
O1	C8	1.359(2)
N1	C12	1.378(2)
N1	C15	1.470(2)
N1	C25	1.473(2)
C1	C2	1.391(3)
C1	C6	1.387(3)
C1	C7	1.506(2)
O2	C14	1.223(2)
C2	C3	1.388(3)
O3	C22	1.329(2)
O3	C23	1.457(2)
C3	C4	1.380(4)
O4	C22	1.200(2)
C4	C5	1.382(3)
O5	C27	1.366(2)
O5	C32	1.422(2)
C5	C6	1.397(3)

O6	C29	1.366(2)
O6	C33	1.423(3)
O7	C31	1.363(2)
O7	C34	1.424(2)
C8	C9	1.413(3)
C8	C13	1.392(3)
C9	C10	1.376(2)
C10	C11	1.393(3)
C11	C12	1.414(2)
C11	C14	1.436(2)
C12	C13	1.395(2)
C14	C15	1.564(2)
C15	C16	1.520(2)
C15	C22	1.543(2)
C16	C17	1.396(3)
C16	C21	1.393(3)
C17	C18	1.388(3)
C18	C19	1.387(3)
C19	C20	1.379(3)
C20	C21	1.390(3)
C23	C24	1.500(3)
C25	C26	1.509(2)
C26	C27	1.394(3)
C26	C31	1.405(2)
C27	C28	1.396(3)
C28	C29	1.389(3)
C29	C30	1.386(3)
C30	C31	1.389(3)

Table 5 Bond Angles for b_a.

Atom	Atom	Atom	Angle/°
C8	O1	C7	117.28(14)
C12	N1	C15	109.78(13)
C12	N1	C25	124.59(14)
C15	N1	C25	121.86(14)
C2	C1	C7	117.39(17)
C6	C1	C2	119.03(17)
C6	C1	C7	123.54(17)
C3	C2	C1	120.7(2)
C22	O3	C23	117.41(15)
C4	C3	C2	120.2(2)
C3	C4	C5	119.53(19)
C27	O5	C32	117.78(15)
C4	C5	C6	120.6(2)
C29	O6	C33	116.99(15)
C1	C6	C5	119.95(19)
C31	O7	C34	118.34(14)
O1	C7	C1	110.15(15)
O1	C8	C9	122.58(16)
O1	C8	C13	115.16(15)
C13	C8	C9	122.25(16)
C10	C9	C8	119.25(17)
C9	C10	C11	119.50(16)
C10	C11	C12	121.09(16)
C10	C11	C14	130.25(16)
C12	C11	C14	108.66(15)
N1	C12	C11	111.59(14)
N1	C12	C13	128.52(15)

C13	C12	C11	119.90(16)
C8	C13	C12	117.96(16)
O2	C14	C11	131.02(17)
O2	C14	C15	122.87(16)
C11	C14	C15	106.10(14)
N1	C15	C14	103.44(13)
N1	C15	C16	113.74(14)
N1	C15	C22	110.25(13)
C16	C15	C14	105.55(13)
C16	C15	C22	116.28(14)
C22	C15	C14	106.34(14)
C17	C16	C15	122.20(17)
C21	C16	C15	118.72(16)
C21	C16	C17	118.54(17)
C18	C17	C16	120.38(19)
C19	C18	C17	120.34(19)
C20	C19	C18	119.74(19)
C19	C20	C21	120.12(19)
C20	C21	C16	120.78(18)
O3	C22	C15	108.70(15)
O4	C22	O3	125.15(18)
O4	C22	C15	126.08(18)
O3	C23	C24	107.56(15)
N1	C25	C26	115.79(15)
C27	C26	C25	121.19(15)
C27	C26	C31	117.25(16)
C31	C26	C25	121.38(16)
O5	C27	C26	114.99(16)
O5	C27	C28	122.66(17)

C26	C27	C28	122.34(16)
C29	C28	C27	118.00(17)
O6	C29	C28	123.61(17)
O6	C29	C30	114.76(16)
C30	C29	C28	121.63(16)
C29	C30	C31	118.88(16)
O7	C31	C26	115.48(15)
O7	C31	C30	122.93(15)
C30	C31	C26	121.59(16)

Table 6 Torsion Angles for b_a.

A	B	C	D	Angle/°
O1	C8	C9	C10	176.15(17)
O1	C8	C13	C12	-178.15(16)
N1	C12	C13	C8	-178.90(17)
N1	C15	C16	C17	162.21(15)
N1	C15	C16	C21	-26.3(2)
N1	C15	C22	O3	57.02(18)
N1	C15	C22	O4	-125.80(19)
N1	C25	C26	C27	75.5(2)
N1	C25	C26	C31	-109.47(18)
C1	C2	C3	C4	-0.3(3)
O2	C14	C15	N1	-175.00(16)
O2	C14	C15	C16	65.3(2)
O2	C14	C15	C22	-58.8(2)
C2	C1	C6	C5	-2.0(3)
C2	C1	C7	O1	-175.11(17)
C2	C3	C4	C5	-1.1(4)
C3	C4	C5	C6	0.9(4)

C4	C5	C6	C1	0.7(3)
O5	C27	C28	C29	179.03(17)
O6	C29	C30	C31	-176.31(16)
C6	C1	C2	C3	1.9(3)
C6	C1	C7	O1	7.5(3)
C7	O1	C8	C9	-4.3(3)
C7	O1	C8	C13	174.46(16)
C7	C1	C2	C3	-175.68(19)
C7	C1	C6	C5	175.4(2)
C8	O1	C7	C1	-165.07(15)
C8	C9	C10	C11	2.6(3)
C9	C8	C13	C12	0.6(3)
C9	C10	C11	C12	-0.8(3)
C9	C10	C11	C14	179.04(19)
C10	C11	C12	N1	178.94(16)
C10	C11	C12	C13	-1.1(3)
C10	C11	C14	O2	-2.1(3)
C10	C11	C14	C15	176.93(18)
C11	C12	C13	C8	1.2(2)
C11	C14	C15	N1	5.86(18)
C11	C14	C15	C16	-113.89(15)
C11	C14	C15	C22	122.02(15)
C12	N1	C15	C14	-6.57(18)
C12	N1	C15	C16	107.40(16)
C12	N1	C15	C22	-119.93(16)
C12	N1	C25	C26	78.5(2)
C12	C11	C14	O2	177.74(19)
C12	C11	C14	C15	-3.21(19)
C13	C8	C9	C10	-2.5(3)

C14	C11	C12	N1	-0.9(2)
C14	C11	C12	C13	179.00(16)
C14	C15	C16	C17	-85.09(19)
C14	C15	C16	C21	86.36(18)
C14	C15	C22	O3	-54.47(17)
C14	C15	C22	O4	122.7(2)
C15	N1	C12	C11	5.0(2)
C15	N1	C12	C13	-174.91(17)
C15	N1	C25	C26	-125.62(17)
C15	C16	C17	C18	174.06(17)
C15	C16	C21	C20	-175.33(16)
C16	C15	C22	O3	-171.63(14)
C16	C15	C22	O4	5.6(3)
C16	C17	C18	C19	0.2(3)
C17	C16	C21	C20	-3.6(3)
C17	C18	C19	C20	-2.0(3)
C18	C19	C20	C21	1.0(3)
C19	C20	C21	C16	1.8(3)
C21	C16	C17	C18	2.6(3)
C22	O3	C23	C24	152.83(17)
C22	C15	C16	C17	32.5(2)
C22	C15	C16	C21	-156.04(16)
C23	O3	C22	O4	-0.5(3)
C23	O3	C22	C15	176.69(14)
C25	N1	C12	C11	163.35(16)
C25	N1	C12	C13	-16.6(3)
C25	N1	C15	C14	-165.60(14)
C25	N1	C15	C16	-51.6(2)
C25	N1	C15	C22	81.04(19)

C25	C26	C27	O5	0.1(3)
C25	C26	C27	C28	-179.38(17)
C25	C26	C31	O7	-0.3(2)
C25	C26	C31	C30	-179.77(18)
C26	C27	C28	C29	-1.6(3)
C27	C26	C31	O7	174.96(17)
C27	C26	C31	C30	-4.6(3)
C27	C28	C29	O6	177.15(18)
C27	C28	C29	C30	-3.4(3)
C28	C29	C30	C31	4.2(3)
C29	C30	C31	O7	-179.54(17)
C29	C30	C31	C26	-0.1(3)
C31	C26	C27	O5	-175.16(16)
C31	C26	C27	C28	5.4(3)
C32	O5	C27	C26	166.36(18)
C32	O5	C27	C28	-14.2(3)
C33	O6	C29	C28	-9.6(3)
C33	O6	C29	C30	170.90(18)
C34	O7	C31	C26	177.02(16)
C34	O7	C31	C30	-3.5(3)

Table 7 Hydrogen Atom Coordinates (Å×104) and Isotropic Displacement Parameters (Å2×103) for b_a.

Atom	x	y	z	U(eq)
H2	10182.03	9429.45	8947.04	38
H3	10097.86	9097.22	10528.52	46
H4	8671.75	7511.18	10834.17	46
H5	7297.46	6316.05	9546.46	47
H6	7367.68	6651.65	7953.16	38
H7A	9011.16	9348.41	7153.44	30

H7B	9700.76	7709.91	7298.5	30
H9	9743.23	8317.16	5901.27	28
H10	9952.17	7604.2	4398.48	27
H13	6607.84	6092.04	5231.02	24
H17	7537.65	3447.28	1087.96	31
H18	8231.76	897.15	1023.08	37
H19	8558.45	-772.41	2364.88	37
H20	8283.31	159.14	3799.7	35
H21	7639.35	2731.87	3886.02	28
H23A	5072.69	7788.28	584.71	33
H23B	6236.97	8776.72	700.13	33
H24A	4861.31	10502.35	948.79	48
H24B	5819.63	10252.59	1970.73	48
H24C	4608.82	9350.4	1741.07	48
H25A	5916.02	3642.49	4063.94	25
H25B	5405.16	3583.8	2911.29	25
H28	2367.05	7224.22	2187.95	31
H30	3115.28	6126.77	5048.72	27
H32A	3555.43	5719.96	445.27	50
H32B	3127.91	7183.06	950.12	50
H32C	2559.33	5481.46	955.47	50
H33A	699.71	8054.66	2453.33	52
H33B	1668.22	9385.55	2810.15	52
H33C	576.3	9354.62	3220.45	52
H34A	3983.1	4162.68	6009.01	47
H34B	4817.45	5660.33	6296.21	47
H34C	5327.99	3921.53	6553.66	47

10. NMR spectra

2a

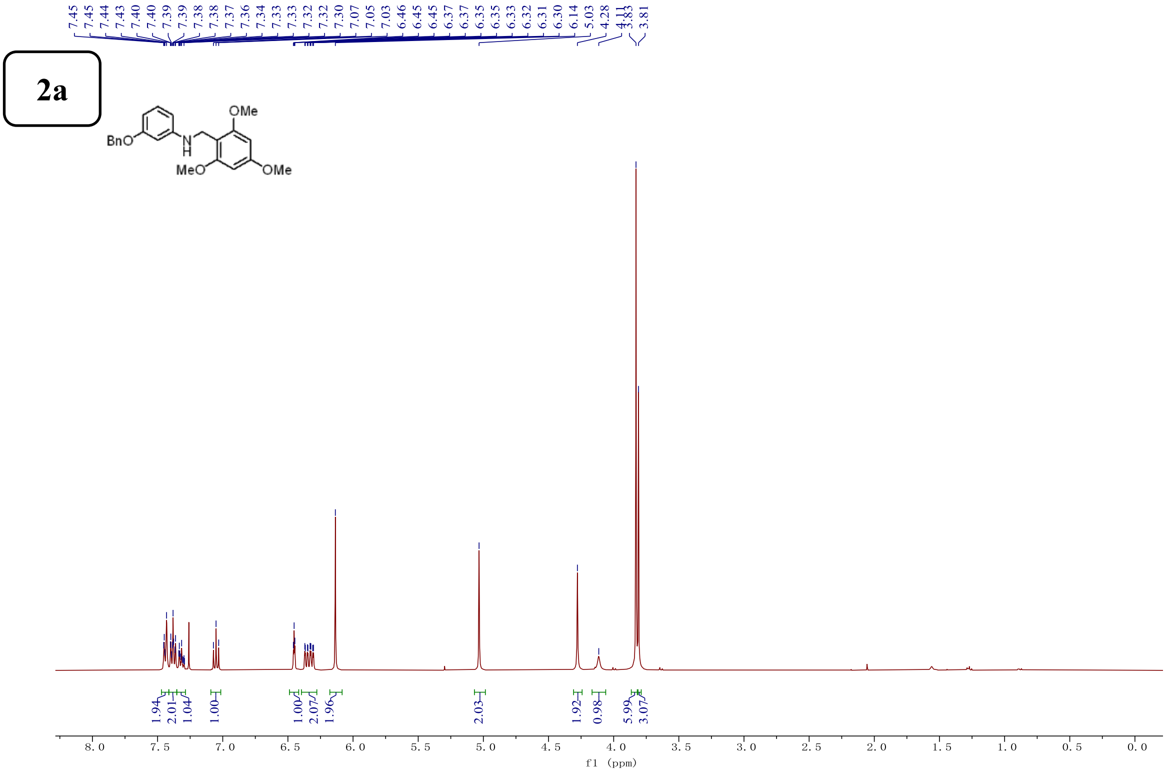

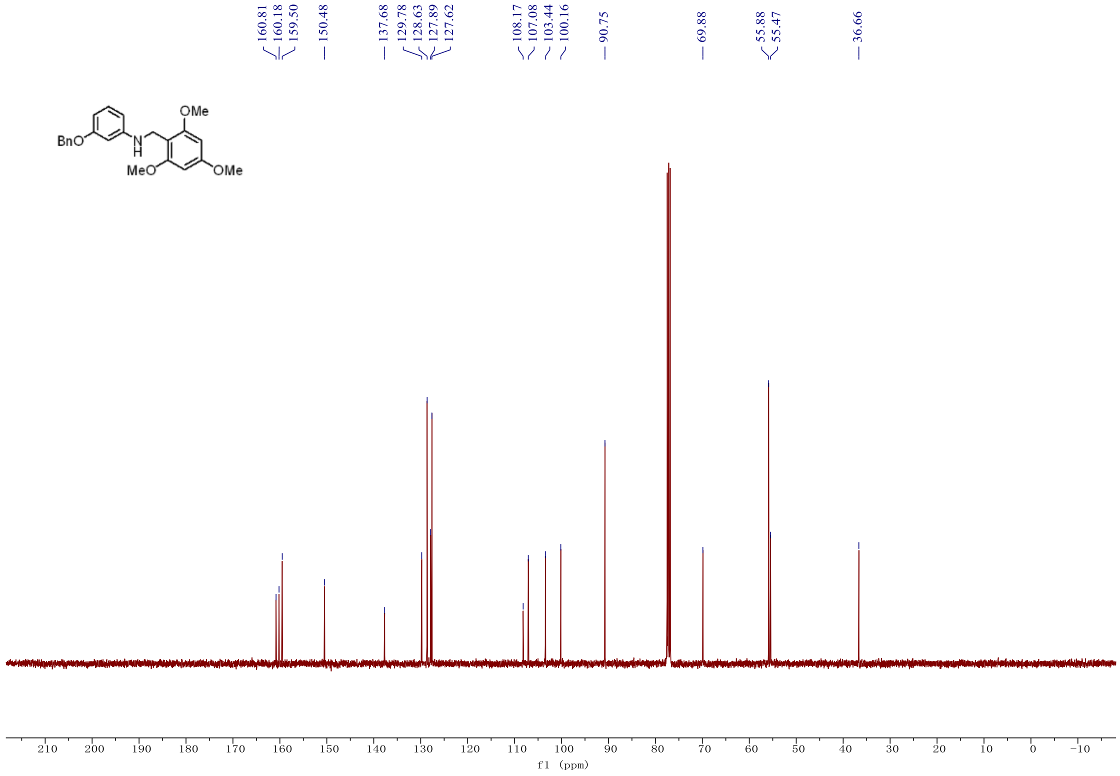

2ae

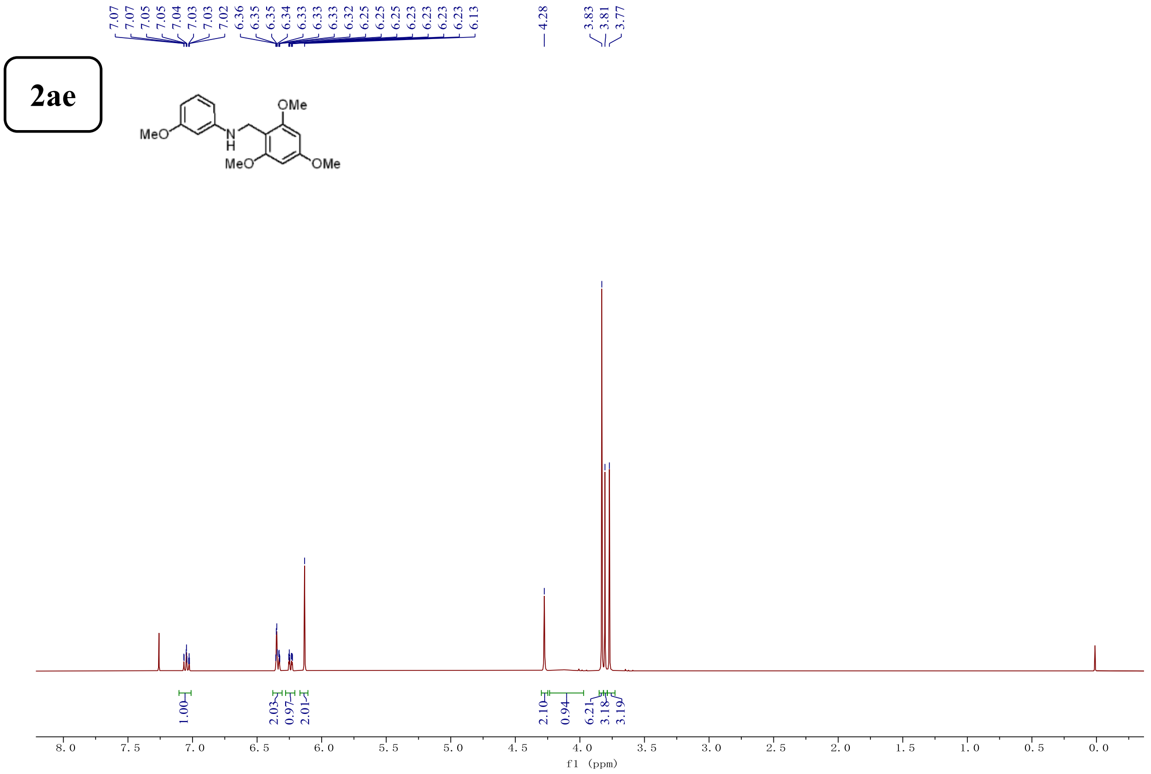
7.07
7.07
7.05
7.05
7.04
7.03
7.03
7.02
6.36
6.35
6.35
6.34
6.33
6.33
6.33
6.32
6.25
6.25
6.23
6.23
6.23
6.23
6.13
4.28
3.83
3.81
3.77
MeO
N
H
OMe
MeO
OMe
1.00
2.03
0.97
2.01
2.10
0.94
6.21
3.18
3.19
f1 (ppm)

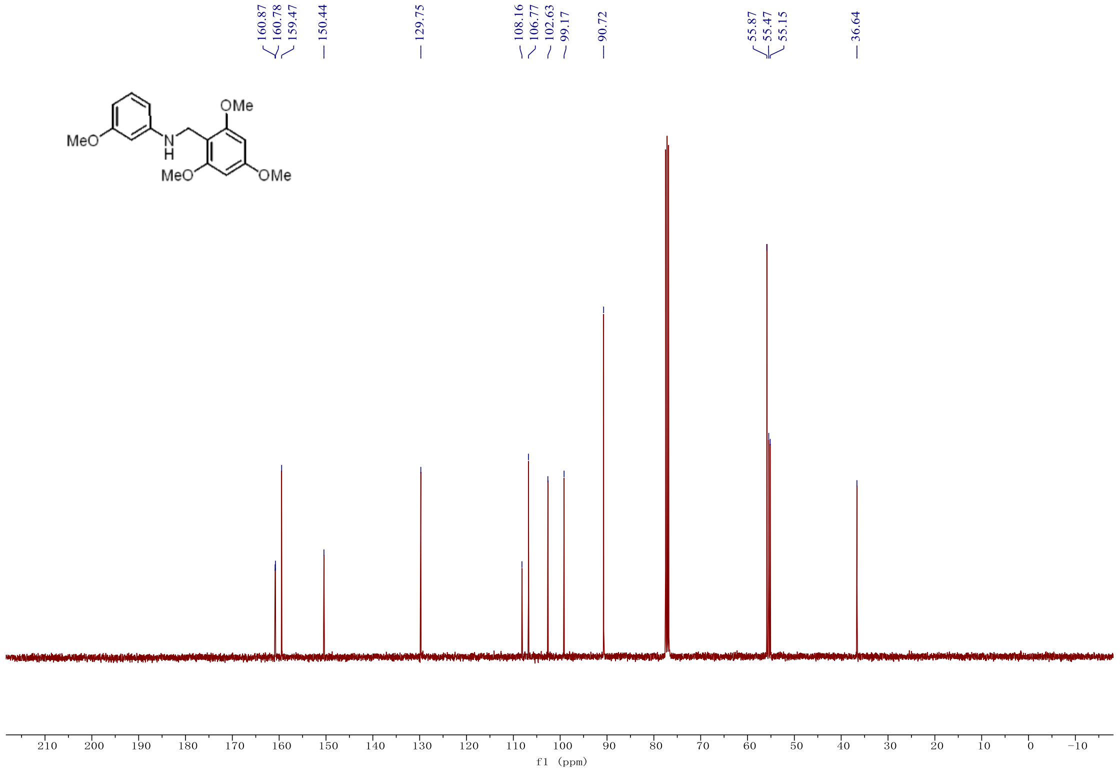
160.87
160.78
159.47
150.44
129.75
108.16
106.77
102.63
99.17
90.72
55.87
55.47
55.15
36.64
MeO
N
H
OMe
MeO
OMe
f1 (ppm)

2af

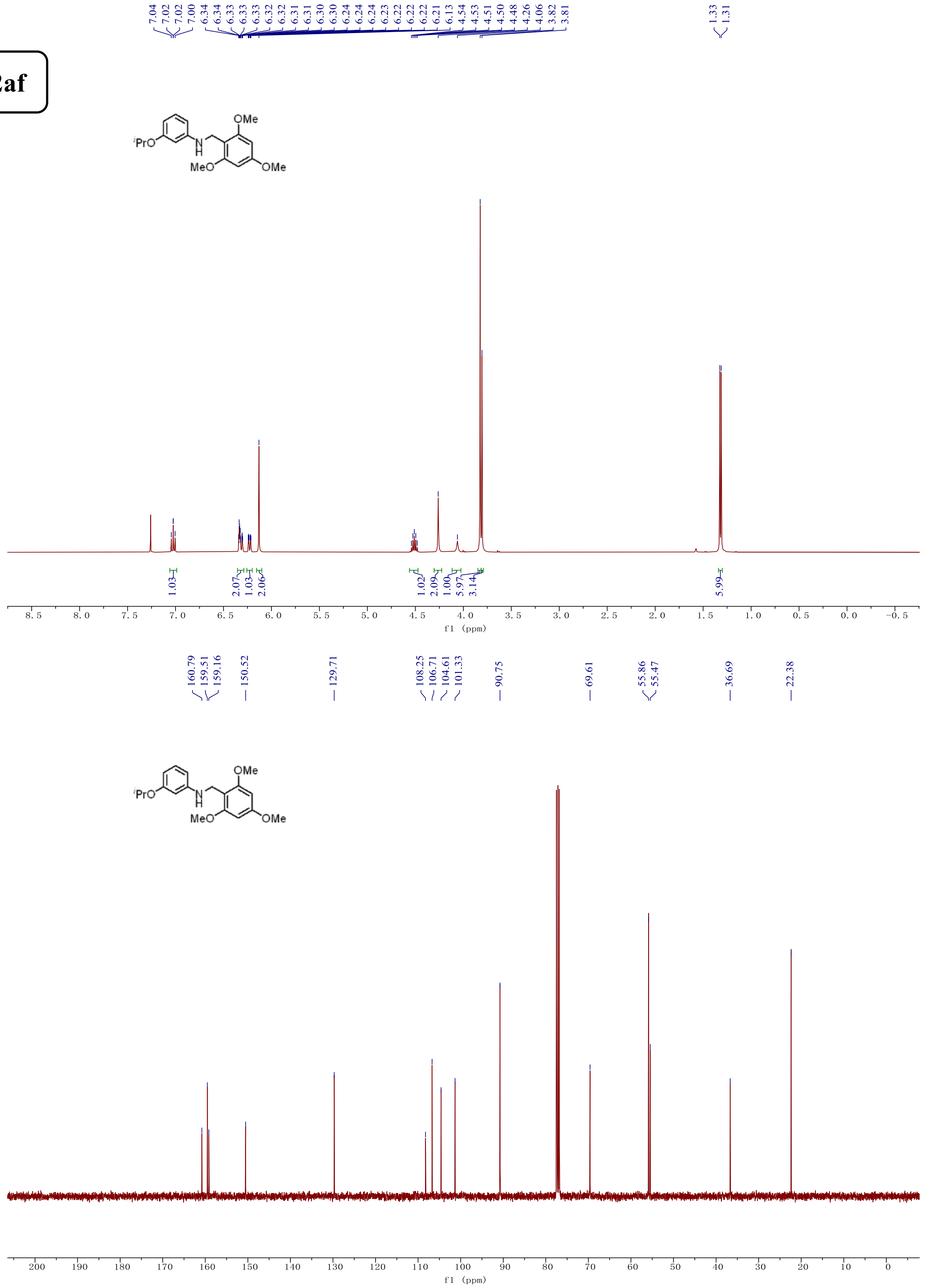

7.04
7.02
7.02
7.00
6.34
6.34
6.33
6.33
6.33
6.32
6.32
6.31
6.31
6.30
6.30
6.24
6.24
6.23
6.22
6.22
6.22
6.21
6.13
4.54
4.53
4.51
4.50
4.48
4.26
4.06
3.82
3.81
1.33
1.31
^{i}PrO
N
H
OMe
MeO
OMe
1.03
2.07
1.03
2.06
1.02
2.09
1.00
5.97
3.14
5.99
f1 (ppm)
160.79
159.51
159.16
150.52
129.71
108.25
106.71
104.61
101.33
90.75
69.61
55.86
55.47
36.69
22.38
f1 (ppm)

2ag

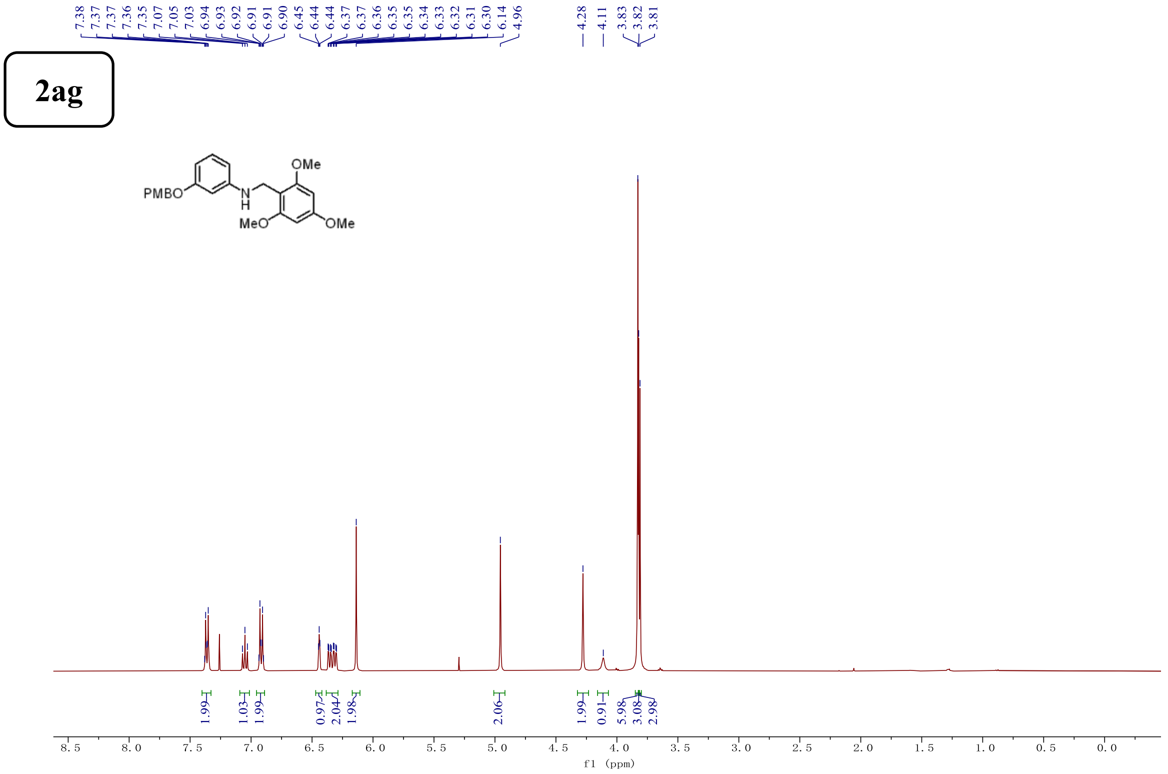

7.38
7.37
7.37
7.36
7.35
7.07
7.05
7.03
6.94
6.93
6.92
6.91
6.91
6.90
6.45
6.44
6.44
6.37
6.37
6.36
6.35
6.35
6.34
6.33
6.32
6.31
6.30
6.14
4.96
4.28
4.11
3.83
3.82
3.81
PMBO
N
H
OMe
MeO
OMe
1.99
1.03
1.99
0.97
2.04
1.98
2.06
1.99
0.91
5.98
3.08
2.98
f1 (ppm)

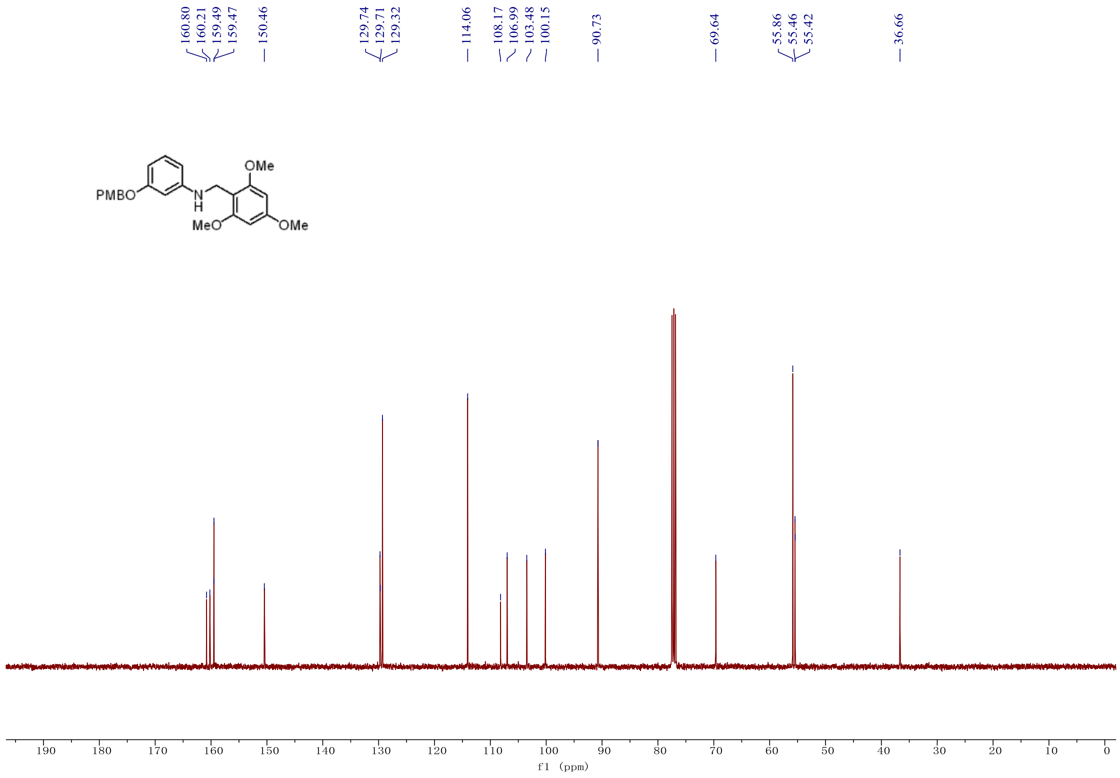

160.80
160.21
159.49
159.47
150.46
129.74
129.71
129.32
114.06
108.17
106.99
103.48
100.15
90.73
69.64
55.86
55.46
55.42
36.66
PMBO
N
H
OMe
MeO
OMe
f1 (ppm)

2ah

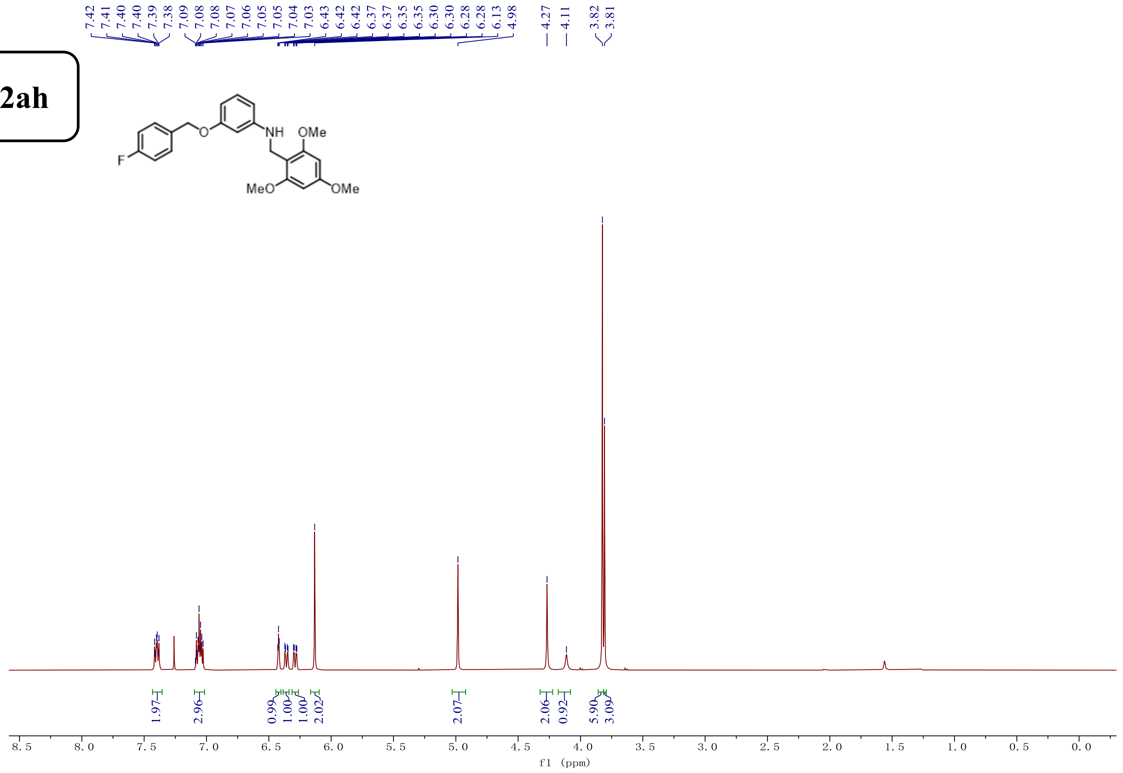

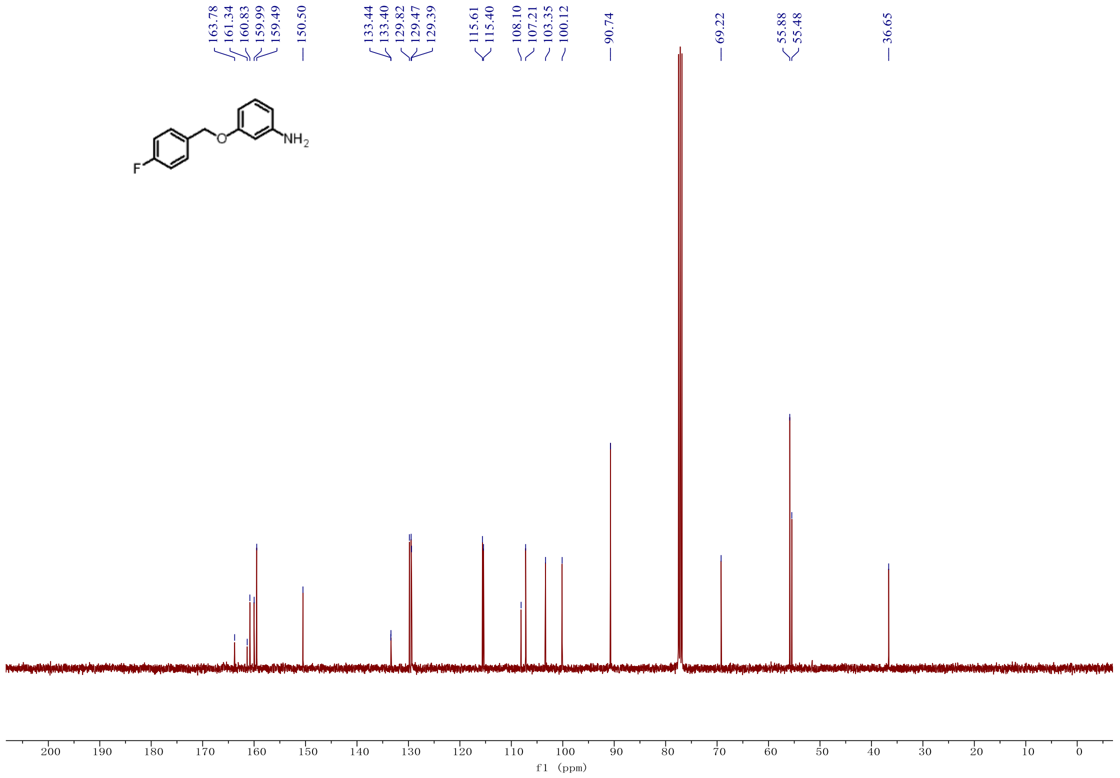

2ai

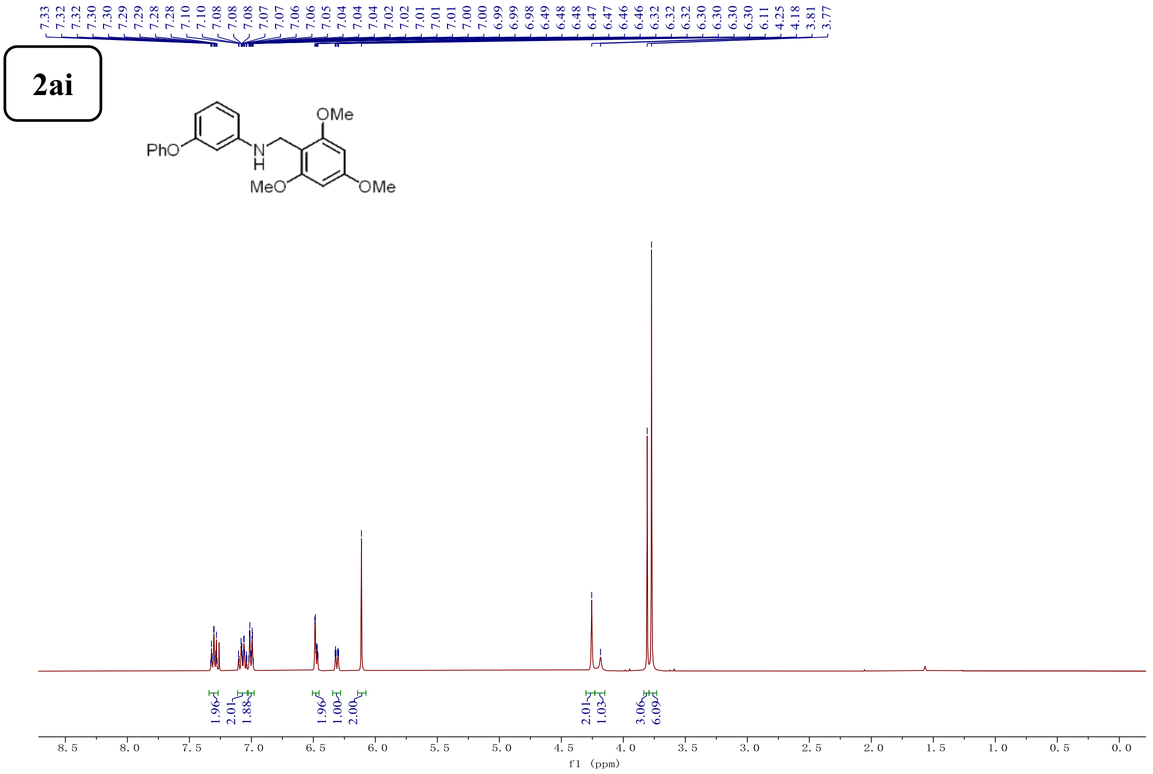
7.33
7.32
7.32
7.30
7.30
7.29
7.29
7.28
7.28
7.10
7.08
7.08
7.08
7.07
7.07
7.06
7.05
7.04
7.04
7.04
7.02
7.01
7.01
7.01
7.00
7.00
6.99
6.99
6.98
6.49
6.48
6.48
6.47
6.47
6.46
6.46
6.32
6.32
6.32
6.30
6.30
6.30
6.30
6.11
4.25
4.18
3.81
3.77
PhO
N
H
OMe
MeO
OMe
1.96
2.01
1.88
1.96
1.00
2.00
2.01
1.03
3.06
6.09
f1 (ppm)

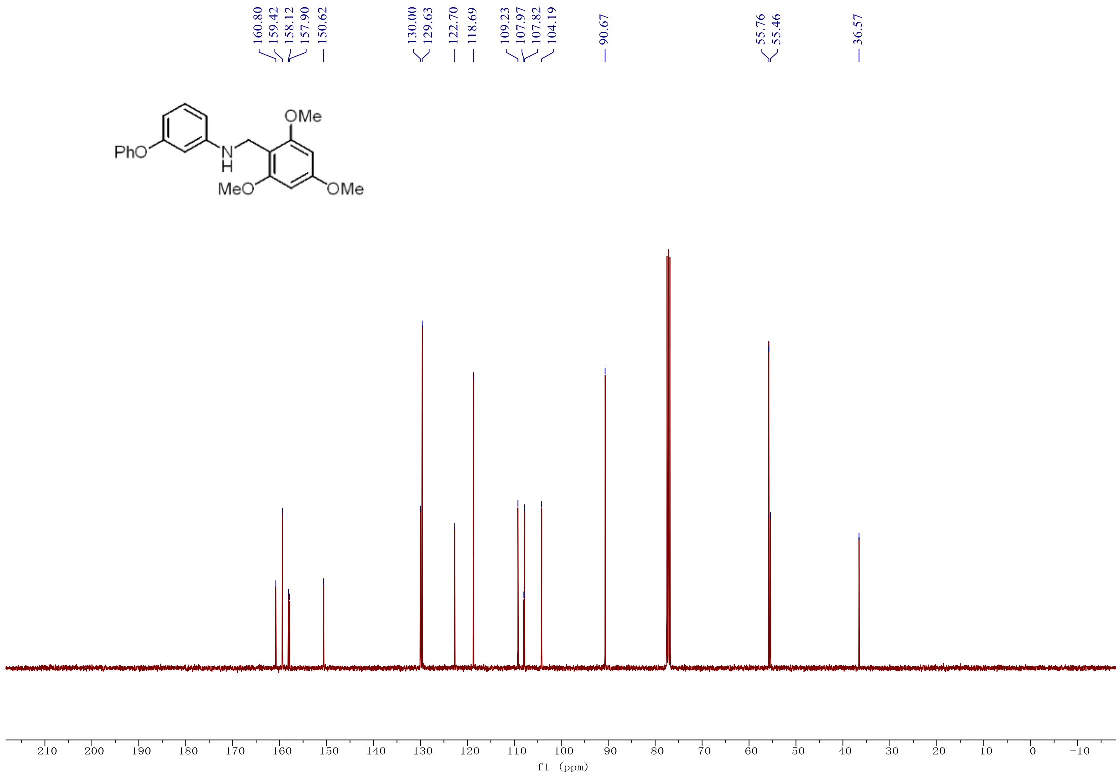
160.80
159.42
158.12
157.90
150.62
130.00
129.63
122.70
118.69
109.23
107.97
107.82
104.19
90.67
55.76
55.46
36.57
PhO
N
H
OMe
MeO
OMe
f1 (ppm)

2aj

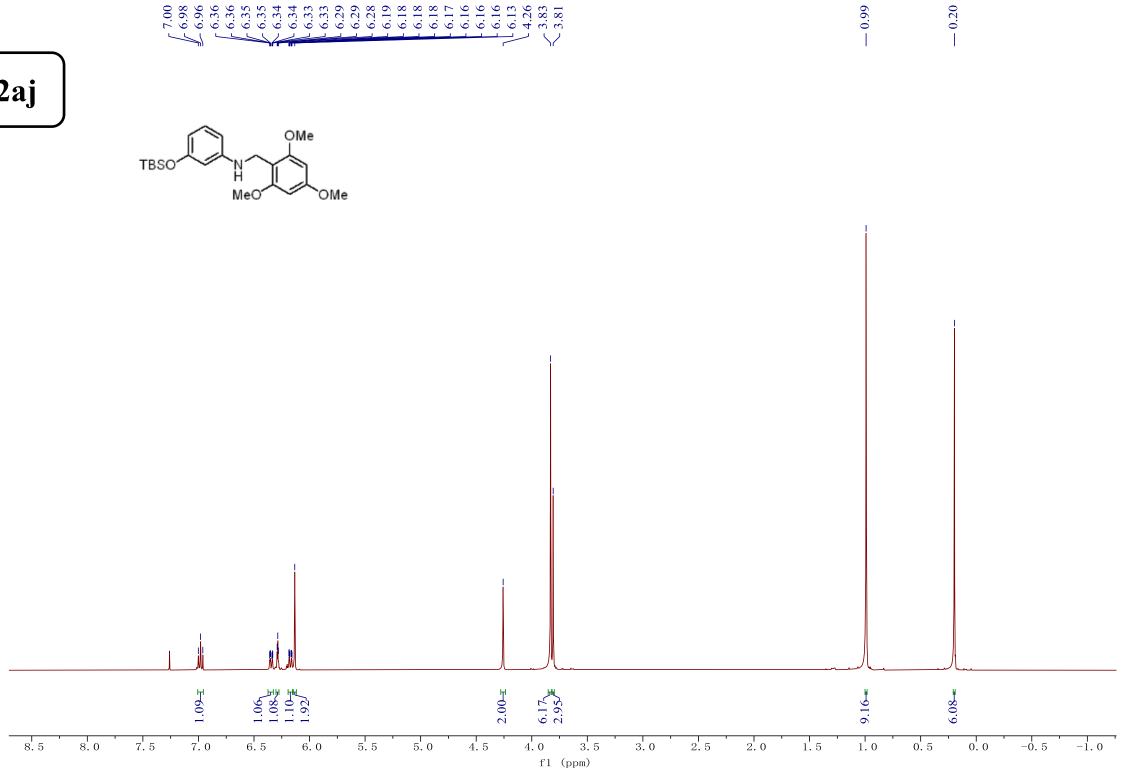
7.00 6.98 6.96 6.36 6.36 6.35 6.35 6.34 6.34 6.33 6.33 6.29 6.29 6.28 6.19 6.18 6.18 6.18 6.17 6.16 6.16 6.16 6.13 4.26 3.83 3.81
0.99
0.20
TBSO
N
H
OMe
MeO
OMe
1.09
1.06 1.08 1.10 1.92
2.00
6.17 2.95
9.16
6.08
f1 (ppm)

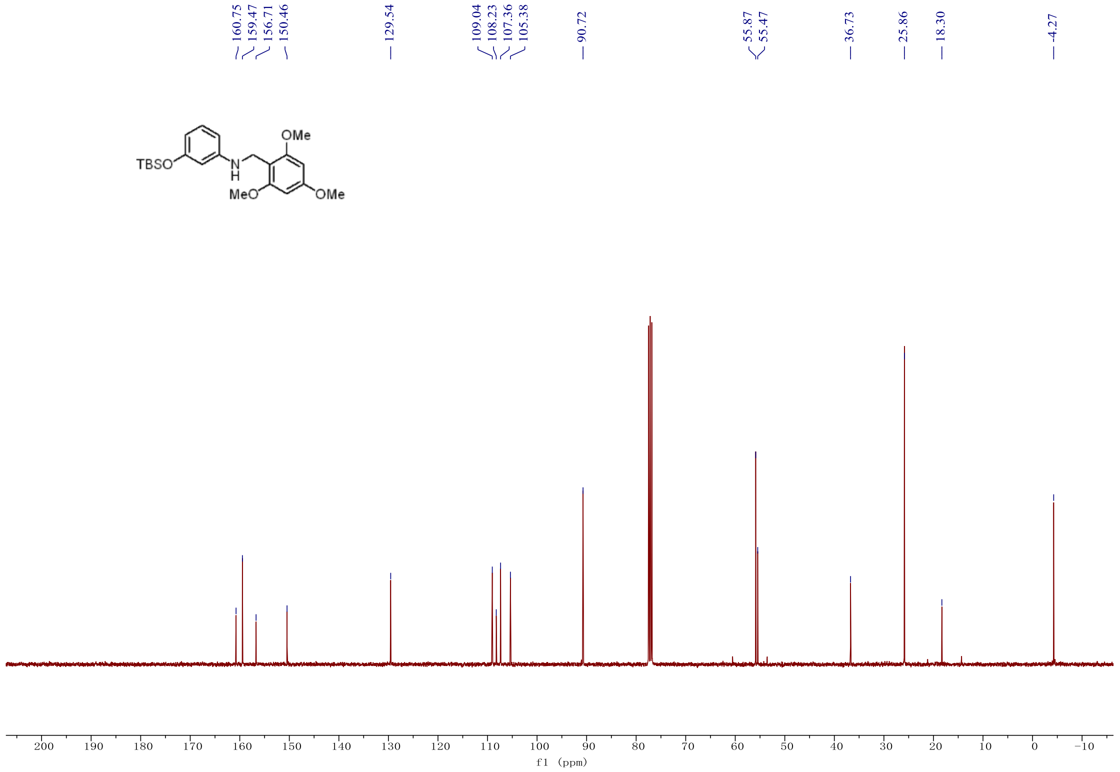
160.75 159.47 156.71 150.46
129.54
109.04 108.23 107.36 105.38
90.72
55.87 55.47
36.73
25.86
18.30
-4.27
TBSO
N
H
OMe
MeO
OMe
f1 (ppm)

2ak

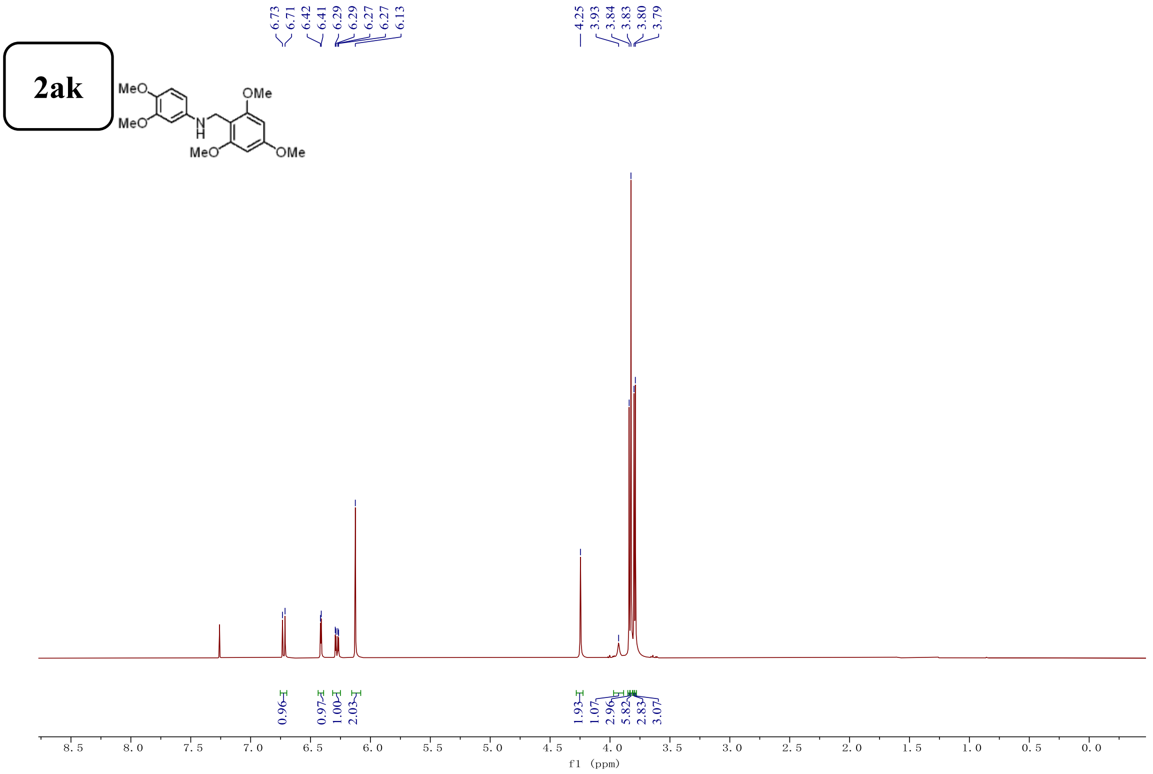
MeO
MeO
N
H
OMe
MeO
OMe
6.73
6.71
6.42
6.41
6.29
6.29
6.27
6.27
6.13
4.25
3.93
3.84
3.83
3.80
3.79
0.96
0.97
1.00
2.03
1.93
1.07
2.96
5.82
2.83
3.07
8.5 8.0 7.5 7.0 6.5 6.0 5.5 5.0 4.5 4.0 3.5 3.0 2.5 2.0 1.5 1.0 0.5 0.0
f1 (ppm)

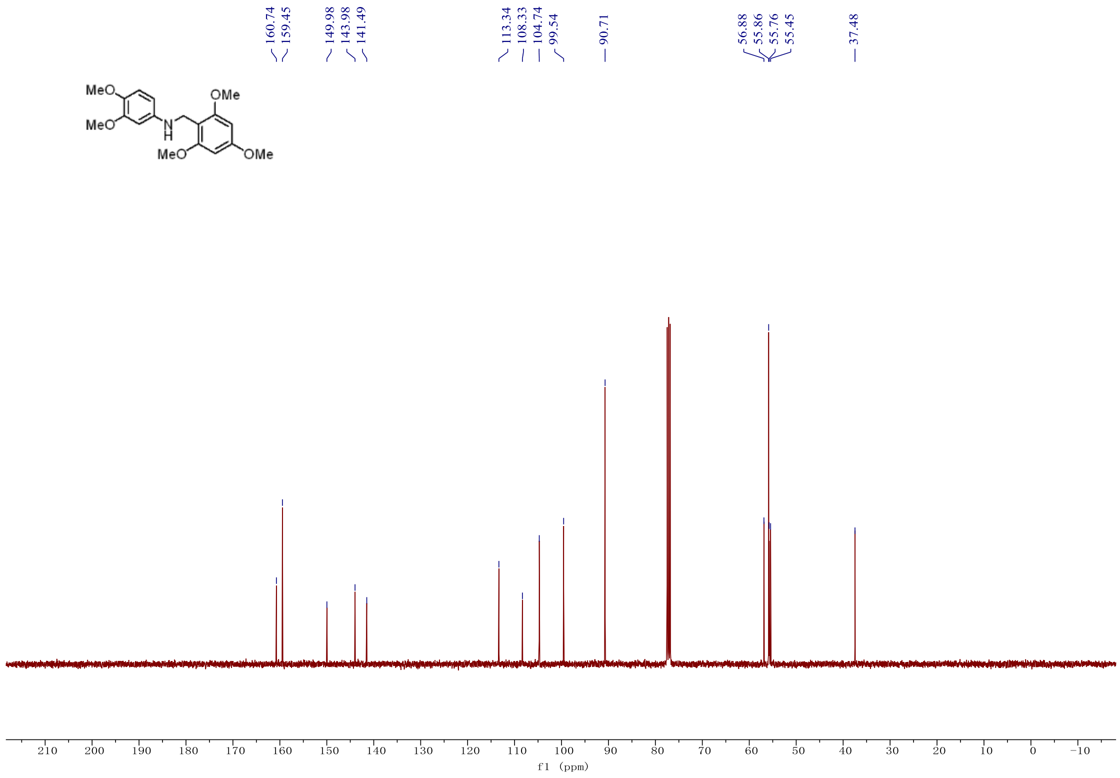
MeO
MeO
N
H
OMe
MeO
OMe
160.74
159.45
149.98
143.98
141.49
113.34
108.33
104.74
99.54
90.71
56.88
55.86
55.76
55.45
37.48
210 200 190 180 170 160 150 140 130 120 110 100 90 80 70 60 50 40 30 20 10 0 -10
f1 (ppm)

2al

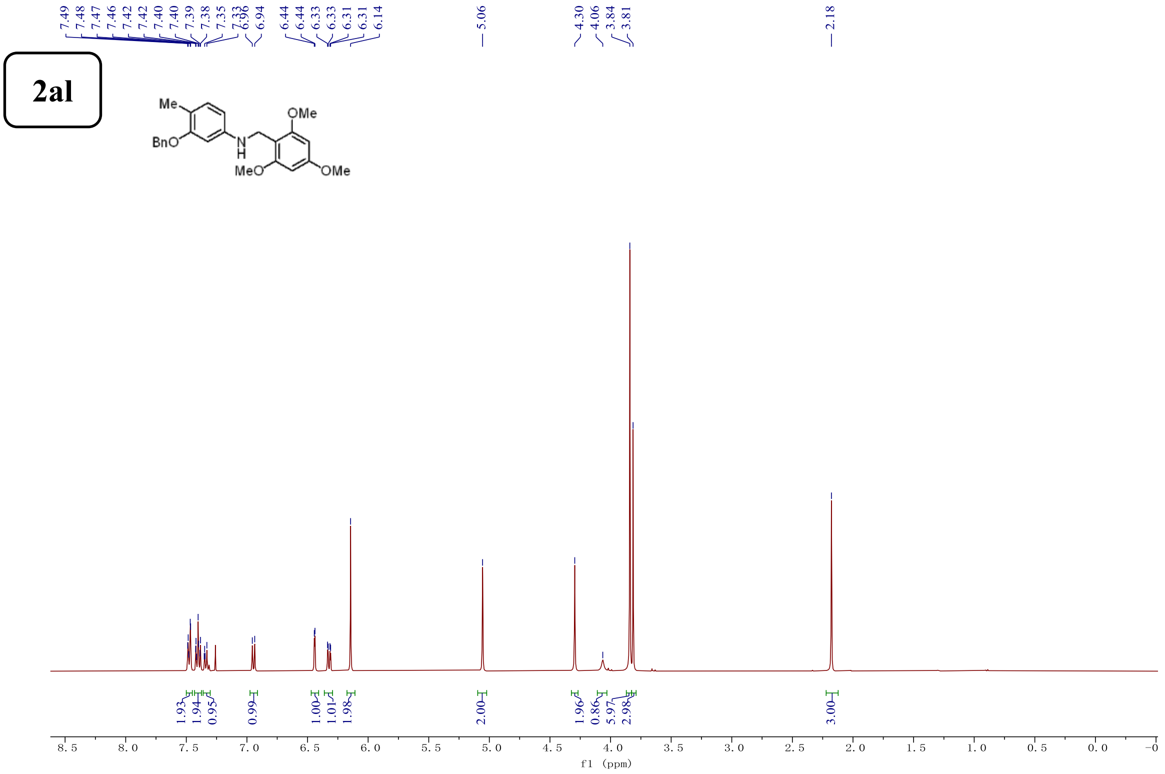
7.49
7.48
7.47
7.46
7.42
7.42
7.40
7.40
7.39
7.38
7.35
7.33
6.96
6.94
6.44
6.44
6.33
6.33
6.31
6.31
6.14
5.06
4.30
4.06
3.84
3.81
2.18
Me
BnO
N
H
OMe
MeO
OMe
1.93
1.94
0.95
0.99
1.00
1.01
1.98
2.00
1.96
0.86
5.97
2.98
3.00
f1 (ppm)

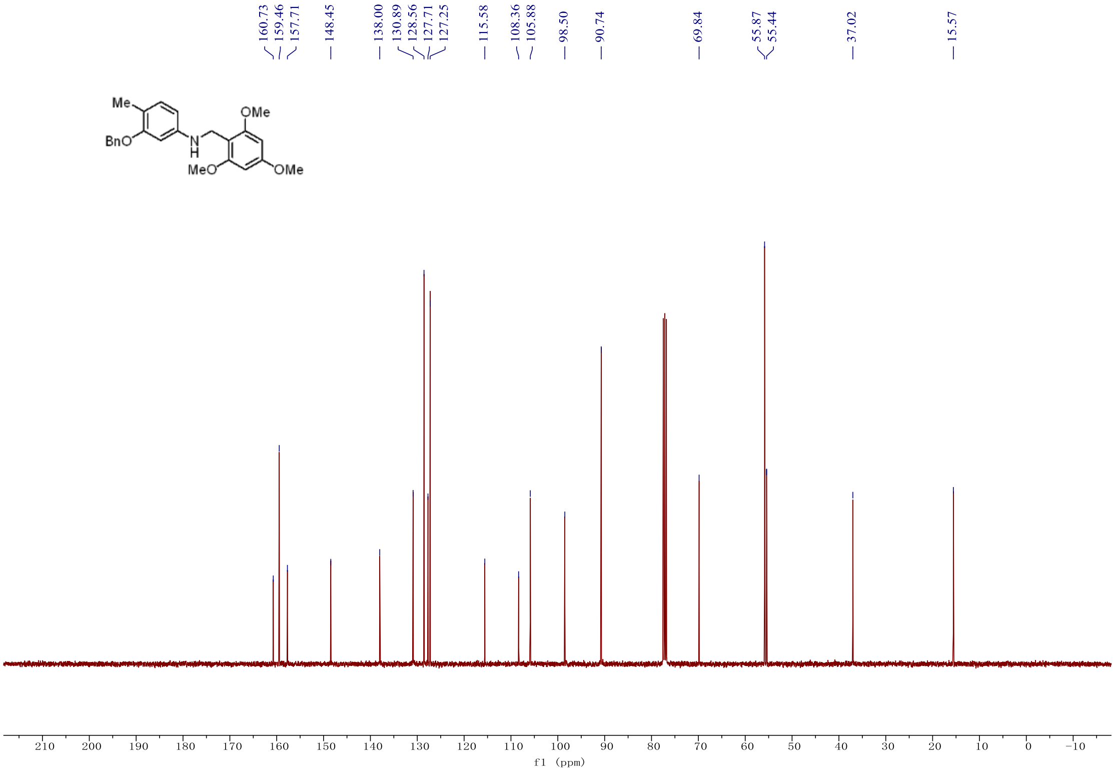
160.73
159.46
157.71
148.45
138.00
130.89
128.56
127.71
127.25
115.58
108.36
105.88
98.50
90.74
69.84
55.87
55.44
37.02
15.57
Me
BnO
N
H
OMe
MeO
OMe
f1 (ppm)

2am

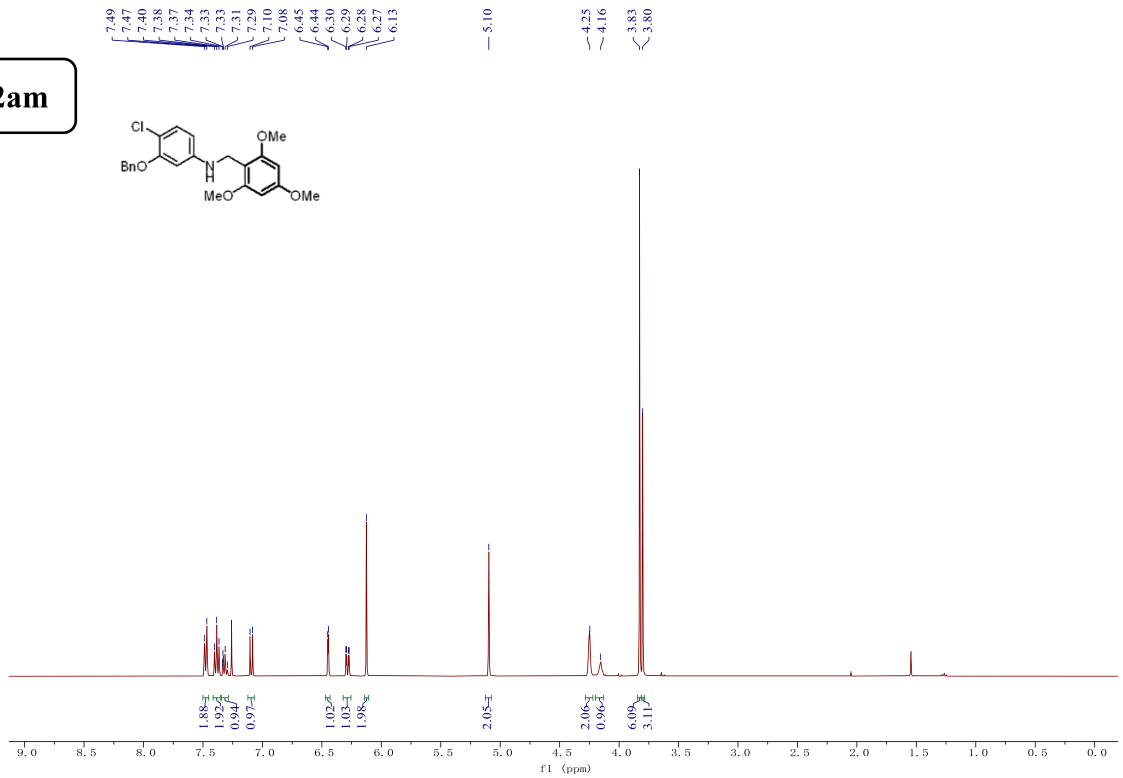
7.49
7.47
7.40
7.38
7.37
7.34
7.33
7.33
7.31
7.29
7.10
7.08
6.45
6.44
6.30
6.29
6.28
6.27
6.13
5.10
4.25
4.16
3.83
3.80
Cl
BnO
H
N
OMe
MeO
OMe
1.88
1.92
0.94
0.97
1.02
1.03
1.98
2.05
2.06
0.96
6.09
3.11
9.0 8.5 8.0 7.5 7.0 6.5 6.0 5.5 5.0 4.5 4.0 3.5 3.0 2.5 2.0 1.5 1.0 0.5 0.0
f1 (ppm)

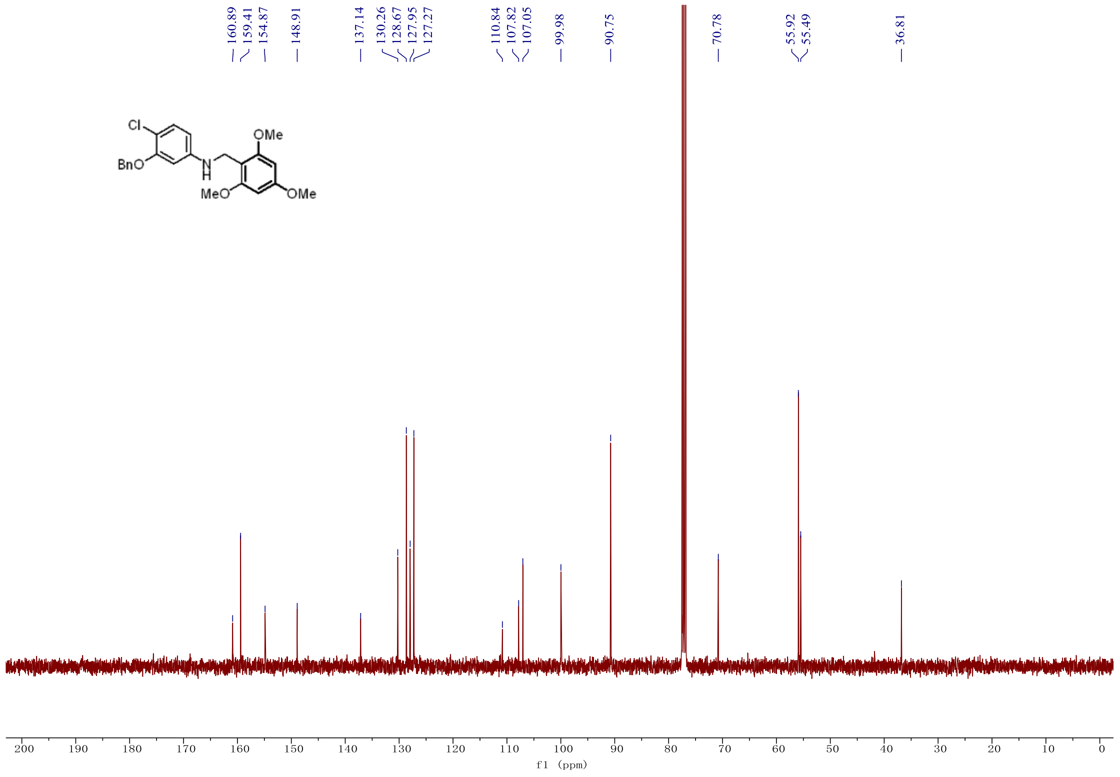
160.89
159.41
154.87
148.91
137.14
130.26
128.67
127.95
127.27
110.84
107.82
107.05
99.98
90.75
70.78
55.92
55.49
36.81
Cl
BnO
H
N
OMe
MeO
OMe
200 190 180 170 160 150 140 130 120 110 100 90 80 70 60 50 40 30 20 10 0
f1 (ppm)

2an

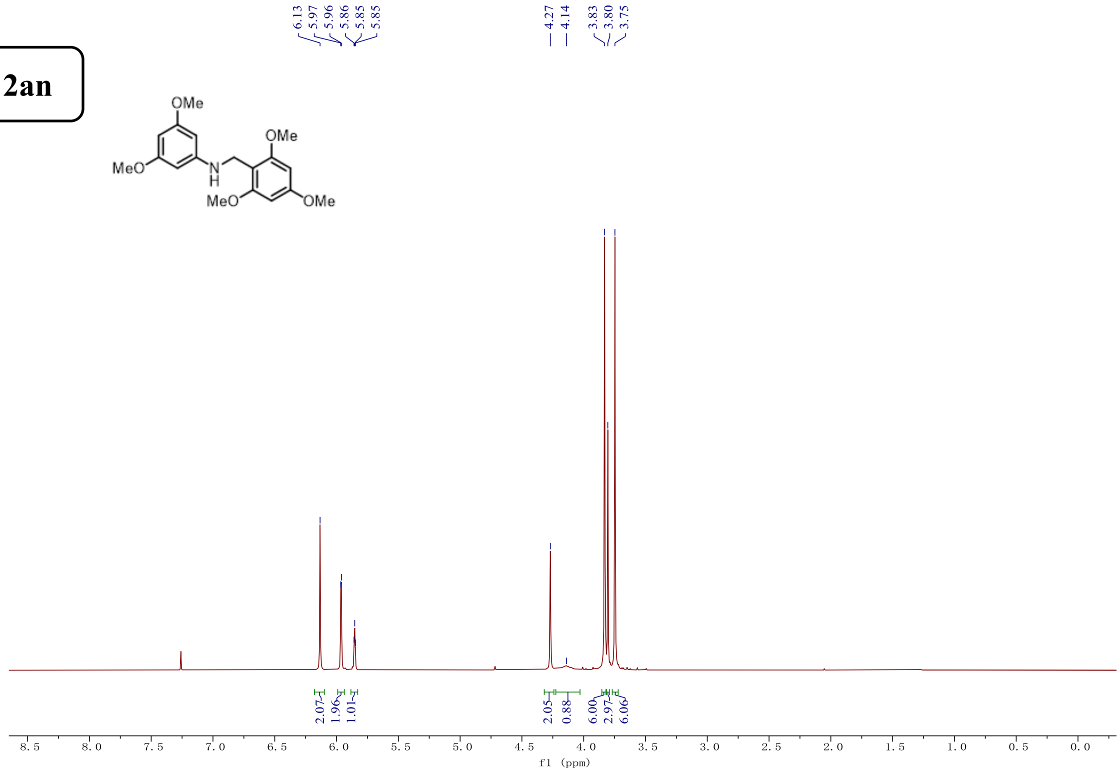
6.13
5.97
5.96
5.86
5.85
5.85
4.27
4.14
3.83
3.80
3.75
OMe
OMe
MeO
N
H
MeO
OMe
2.07
1.96
1.01
2.05
0.88
6.00
2.97
6.06
8.5 8.0 7.5 7.0 6.5 6.0 5.5 5.0 4.5 4.0 3.5 3.0 2.5 2.0 1.5 1.0 0.5 0.0
f1 (ppm)

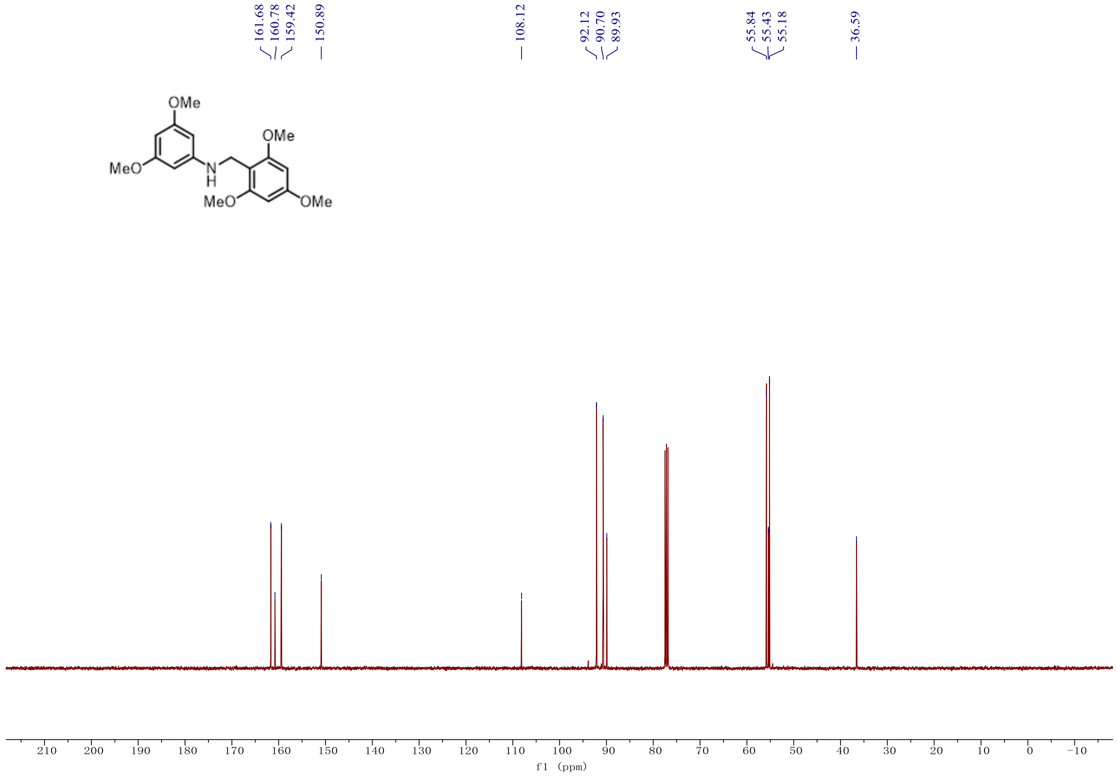
161.68
160.78
159.42
150.89
108.12
92.12
90.70
89.93
55.84
55.43
55.18
36.59
OMe
OMe
MeO
N
H
MeO
OMe
210 200 190 180 170 160 150 140 130 120 110 100 90 80 70 60 50 40 30 20 10 0 -10
f1 (ppm)

2ao

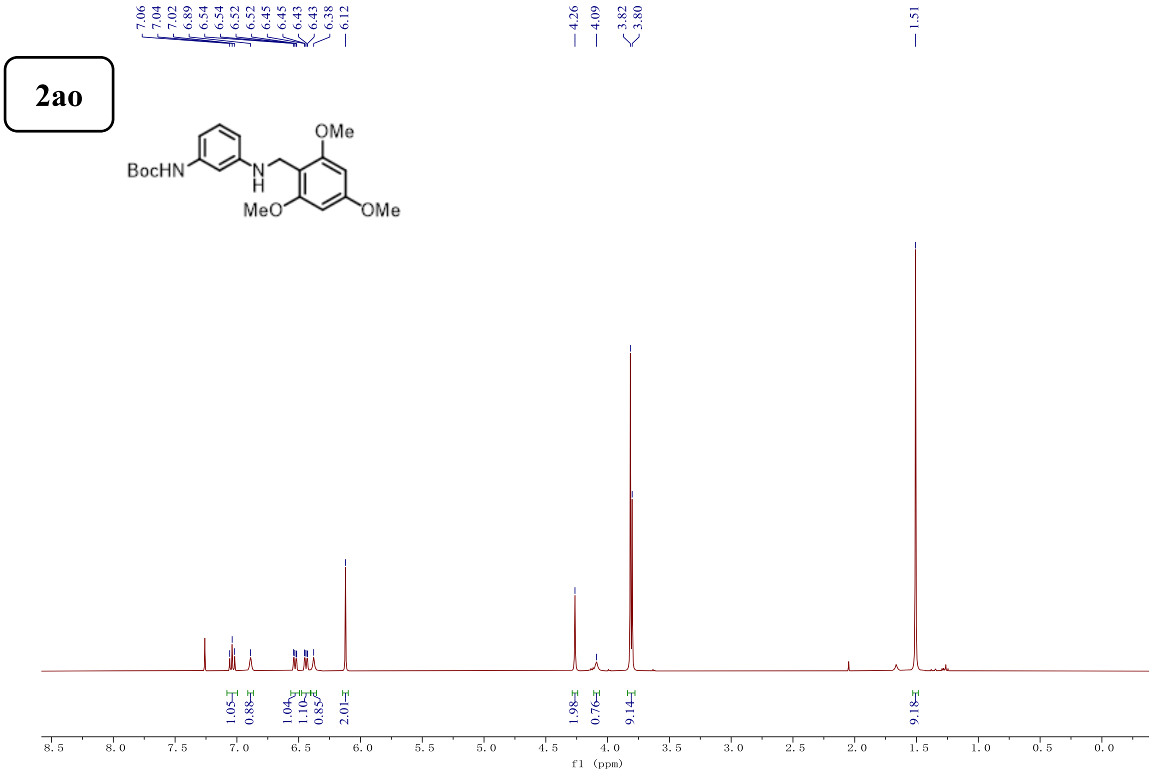

7.06
7.04
7.02
6.89
6.54
6.54
6.52
6.52
6.45
6.45
6.43
6.43
6.38
6.12
4.26
4.09
3.82
3.80
1.51
BocHN
N
H
OMe
MeO
OMe
1.05
0.88
1.04
1.10
0.85
2.01
1.98
0.76
9.14
9.18
8.5 8.0 7.5 7.0 6.5 6.0 5.5 5.0 4.5 4.0 3.5 3.0 2.5 2.0 1.5 1.0 0.5 0.0
f1 (ppm)

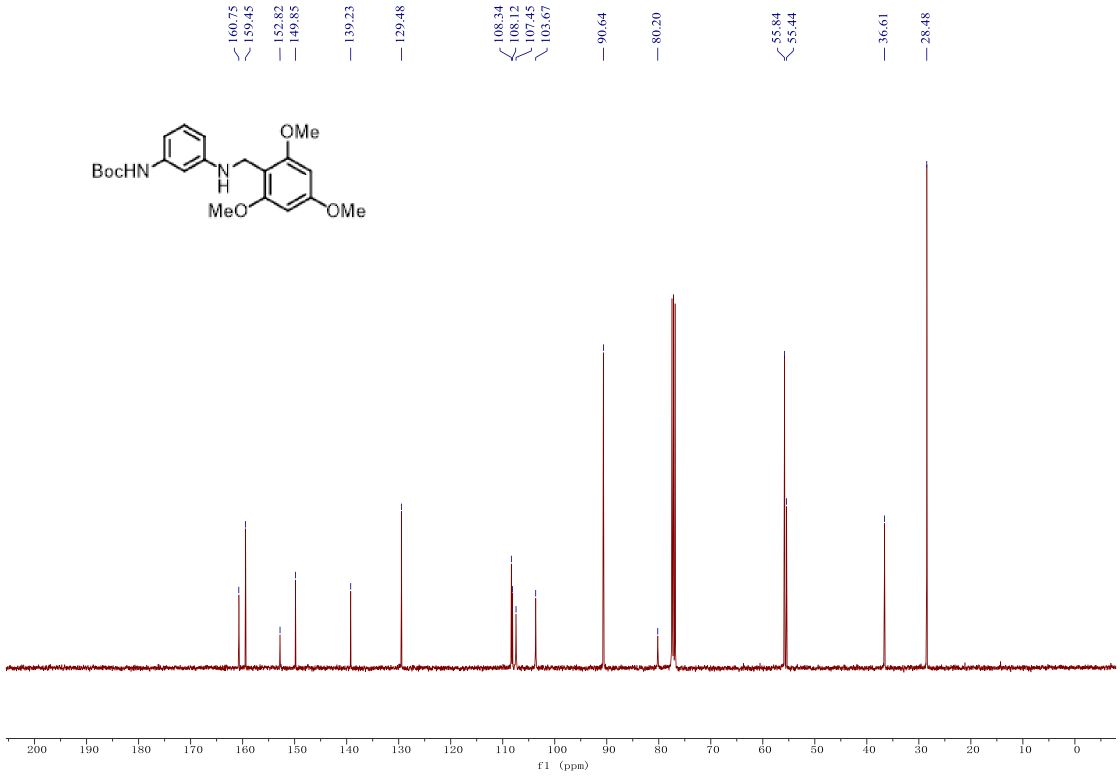

160.75
159.45
152.82
149.85
139.23
129.48
108.34
108.12
107.45
103.67
90.64
80.20
55.84
55.44
36.61
28.48
BocHN
N
H
OMe
MeO
OMe
200 190 180 170 160 150 140 130 120 110 100 90 80 70 60 50 40 30 20 10 0
f1 (ppm)

2ap

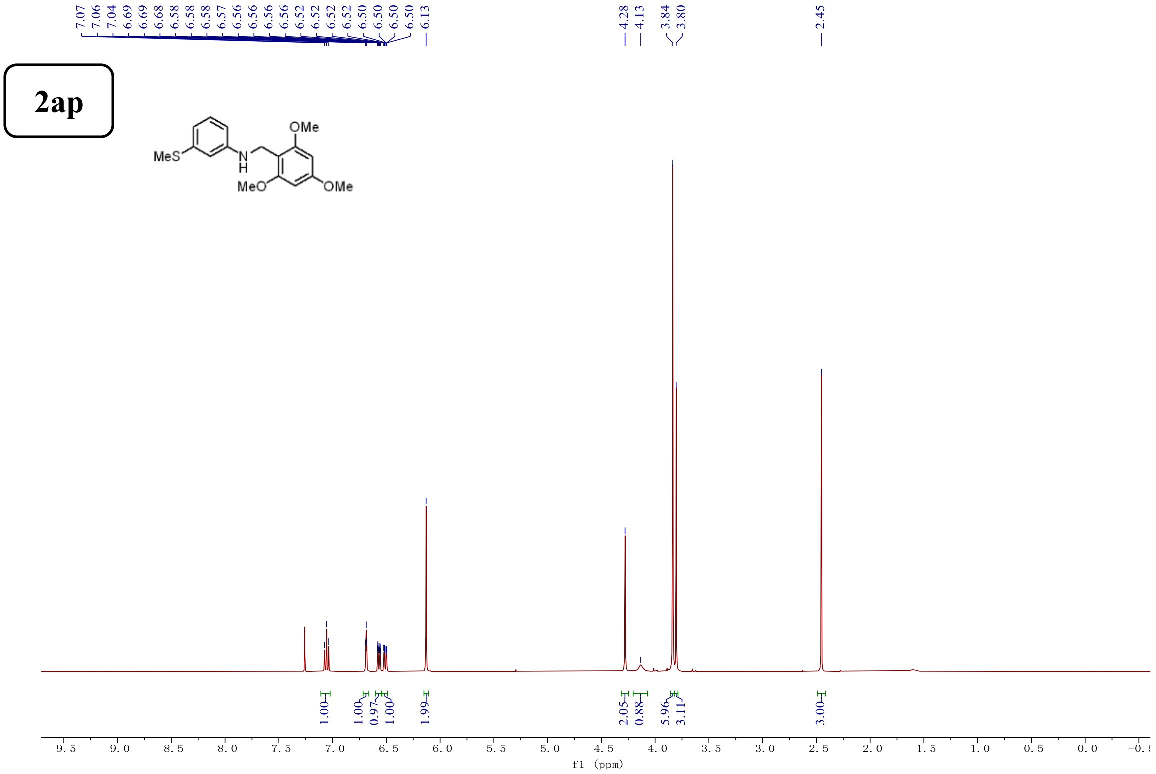

7.07
7.06
7.04
6.69
6.69
6.68
6.58
6.58
6.57
6.56
6.56
6.56
6.52
6.52
6.52
6.50
6.50
6.50
6.50
6.13
4.28
4.13
3.84
3.80
2.45
MeS
N
H
OMe
MeO
OMe
1.00
1.00
0.97
1.00
1.99
2.05
0.88
5.96
3.11
3.00
f1 (ppm)

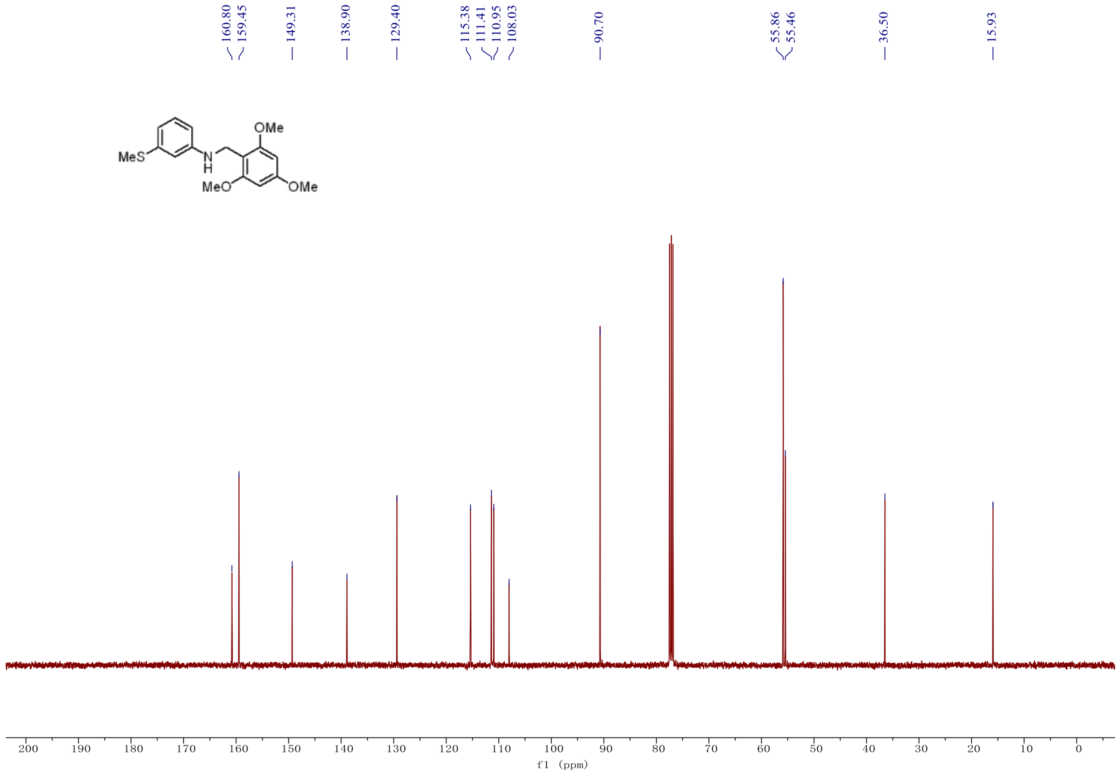

160.80
159.45
149.31
138.90
129.40
115.38
111.41
110.95
108.03
90.70
55.86
55.46
36.50
15.93
MeS
N
H
OMe
MeO
OMe
f1 (ppm)

3a

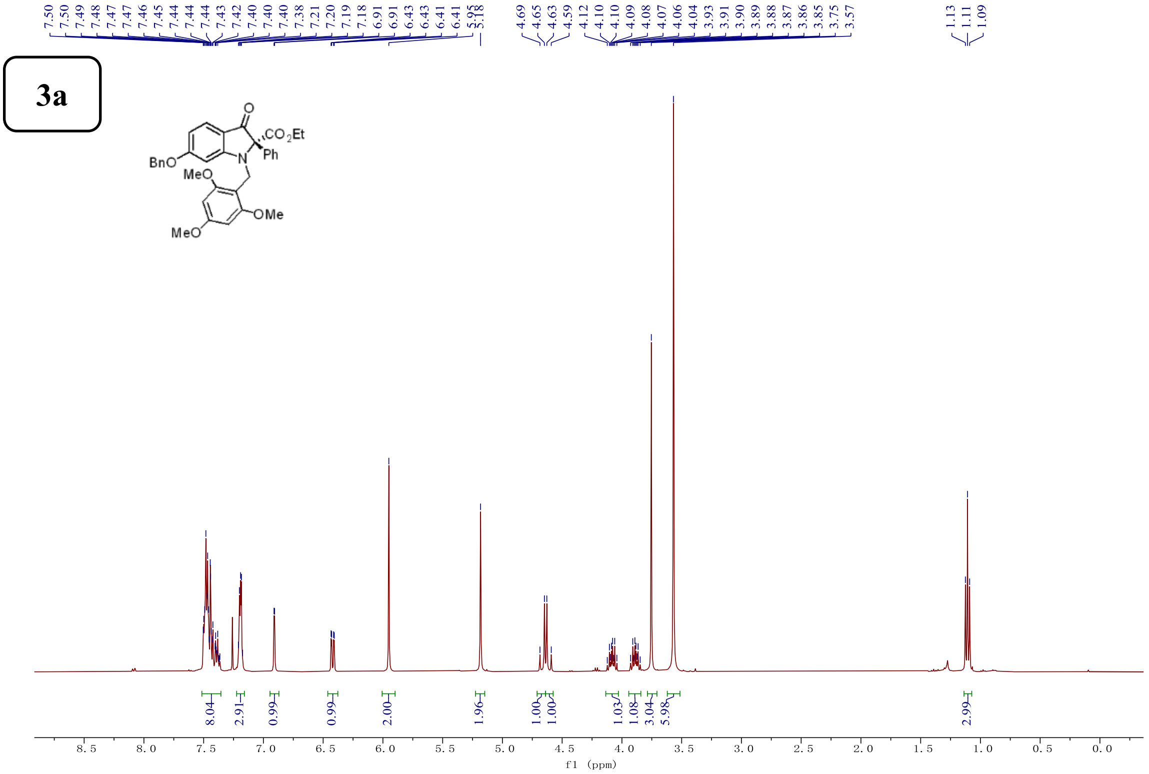
7.50
7.50
7.49
7.48
7.47
7.47
7.46
7.45
7.44
7.44
7.44
7.43
7.42
7.40
7.40
7.38
7.21
7.20
7.19
7.18
6.91
6.91
6.43
6.43
6.41
6.41
5.95
5.18
4.69
4.65
4.63
4.59
4.12
4.10
4.10
4.09
4.08
4.07
4.06
4.04
3.93
3.91
3.90
3.89
3.88
3.87
3.86
3.85
3.75
3.57
1.13
1.11
1.09
O
CO2Et
Ph
N
BnO
MeO
OMe
MeO
8.04
2.91
0.99
0.99
2.00
1.96
1.00
1.00
1.03
1.08
3.04
5.98
2.99
f1 (ppm)

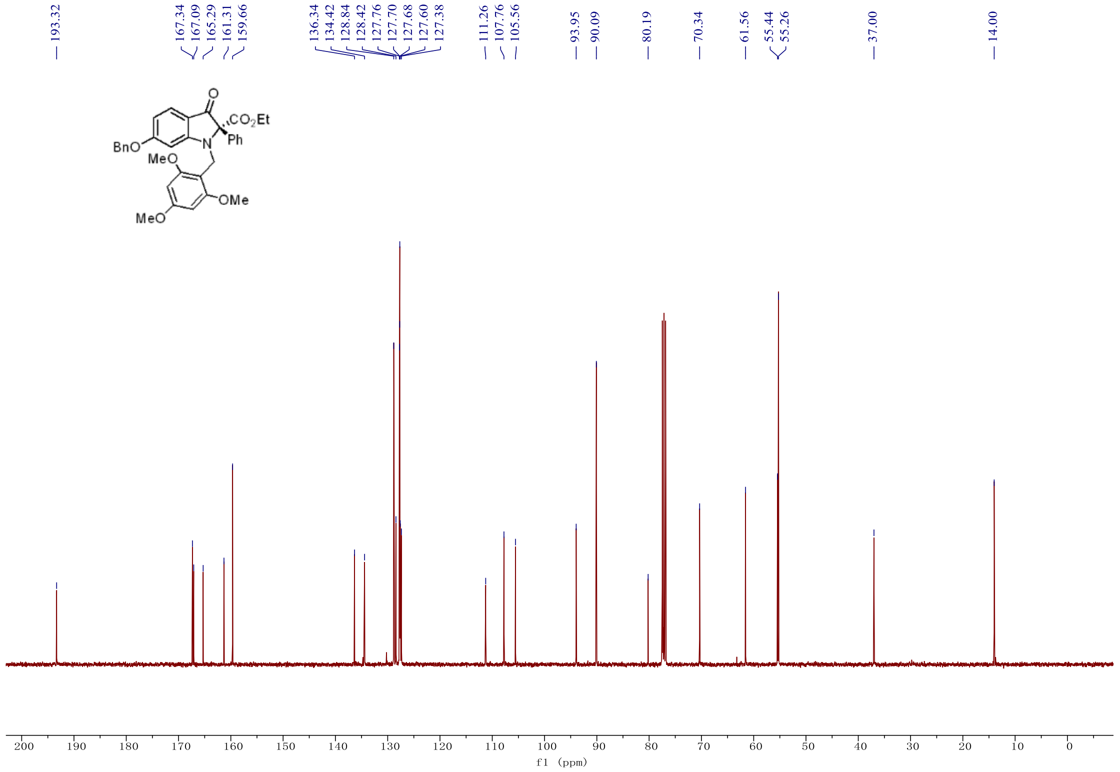
193.32
167.34
167.09
165.29
161.31
159.66
136.34
134.42
128.84
128.42
127.76
127.70
127.68
127.60
127.38
111.26
107.76
105.56
93.95
90.09
80.19
70.34
61.56
55.44
55.26
37.00
14.00
O
CO2Et
Ph
N
BnO
MeO
OMe
MeO
f1 (ppm)

3b

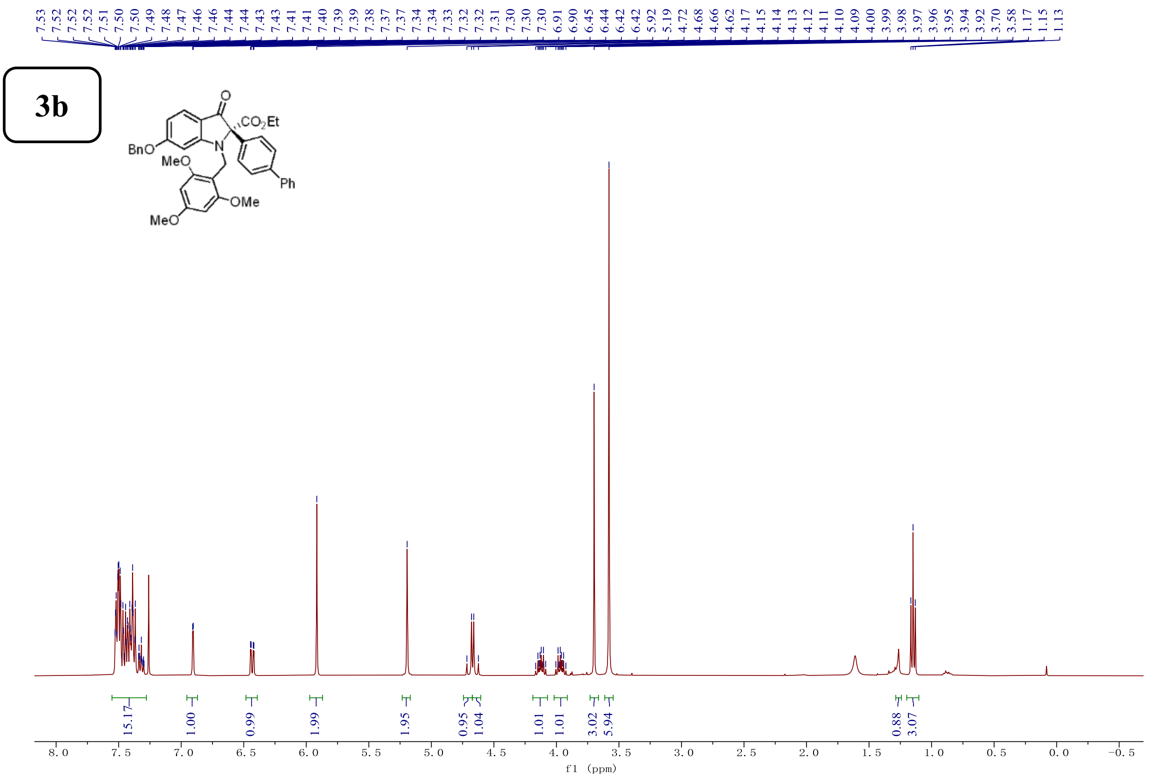
7.53 7.52 7.52 7.51 7.50 7.50 7.49 7.48 7.47 7.46 7.46 7.44 7.44 7.43 7.43 7.41 7.41 7.40 7.39 7.39 7.38 7.37 7.37 7.34 7.34 7.33 7.32 7.32 7.31 7.30 7.30 7.30 6.91 6.90 6.45 6.44 6.42 6.42 5.92 5.19 4.72 4.68 4.66 4.62 4.17 4.15 4.14 4.13 4.12 4.11 4.10 4.09 4.00 3.99 3.98 3.97 3.96 3.95 3.94 3.92 3.70 3.58 1.17 1.15 1.13
BnO
CO2Et
N
Ph
MeO
OMe
MeO
15.17 1.00 0.99 1.99 1.95 0.95 1.04 1.01 1.01 3.02 5.94 0.88 3.07
f1 (ppm)

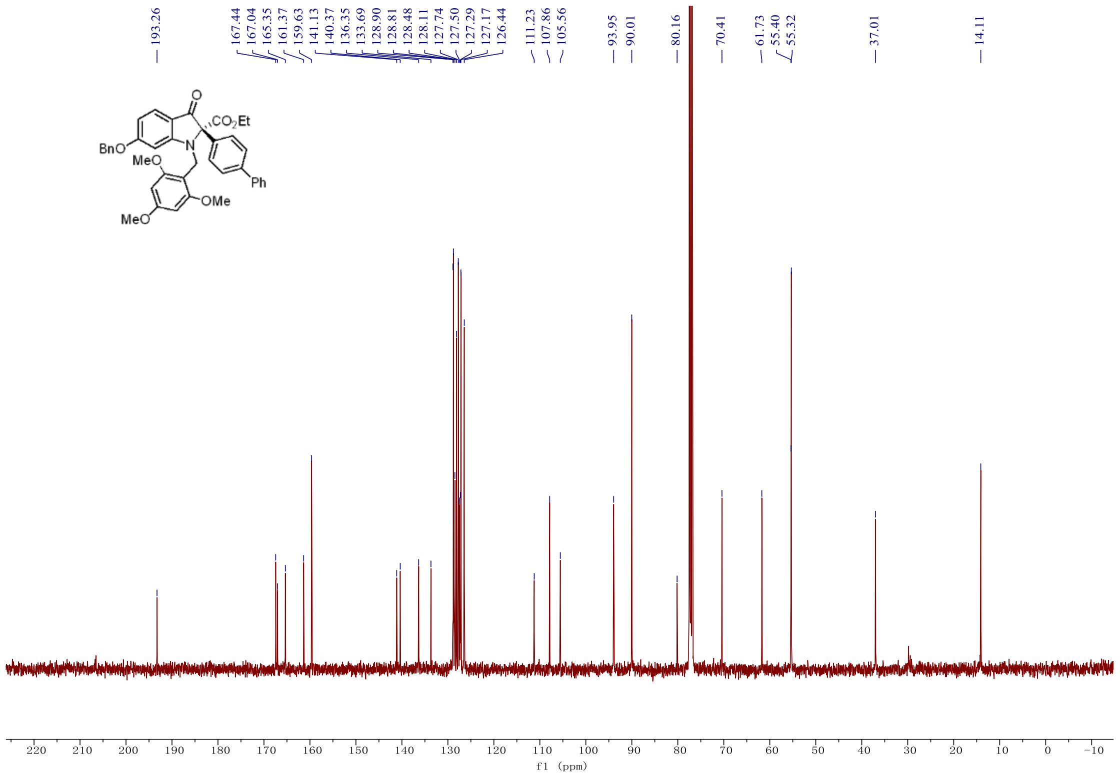
193.26 167.44 167.04 165.35 161.37 159.63 141.13 140.37 136.35 133.69 128.90 128.81 128.48 128.11 127.74 127.50 127.29 127.17 126.44 111.23 107.86 105.56 93.95 90.01 80.16 70.41 61.73 55.40 55.32 37.01 14.11
BnO
CO2Et
N
Ph
MeO
OMe
MeO
f1 (ppm)

3c

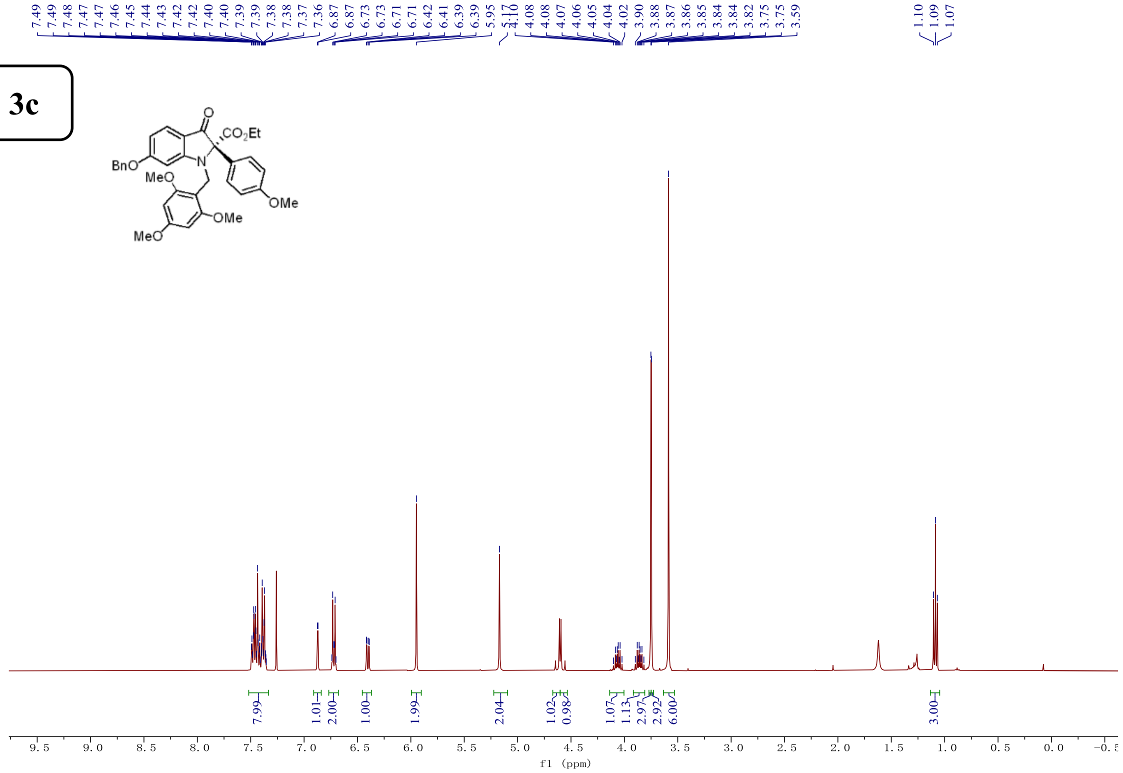
7.49 7.49 7.48 7.47 7.47 7.46 7.45 7.44 7.43 7.42 7.42 7.40 7.40 7.39 7.39 7.38 7.38 7.37 7.36 6.87 6.87 6.73 6.73 6.71 6.71 6.42 6.41 6.39 6.39 5.95 5.17 4.10 4.08 4.08 4.07 4.06 4.05 4.04 4.02 3.90 3.88 3.87 3.86 3.85 3.84 3.84 3.82 3.75 3.75 3.59
1.10 1.09 1.07
CO2Et
BnO
MeO
N
OMe
MeO
OMe
7.99 1.01 2.00 1.00 1.99 2.04 1.02 0.98 1.07 1.13 2.97 2.92 6.00 3.00
f1 (ppm)

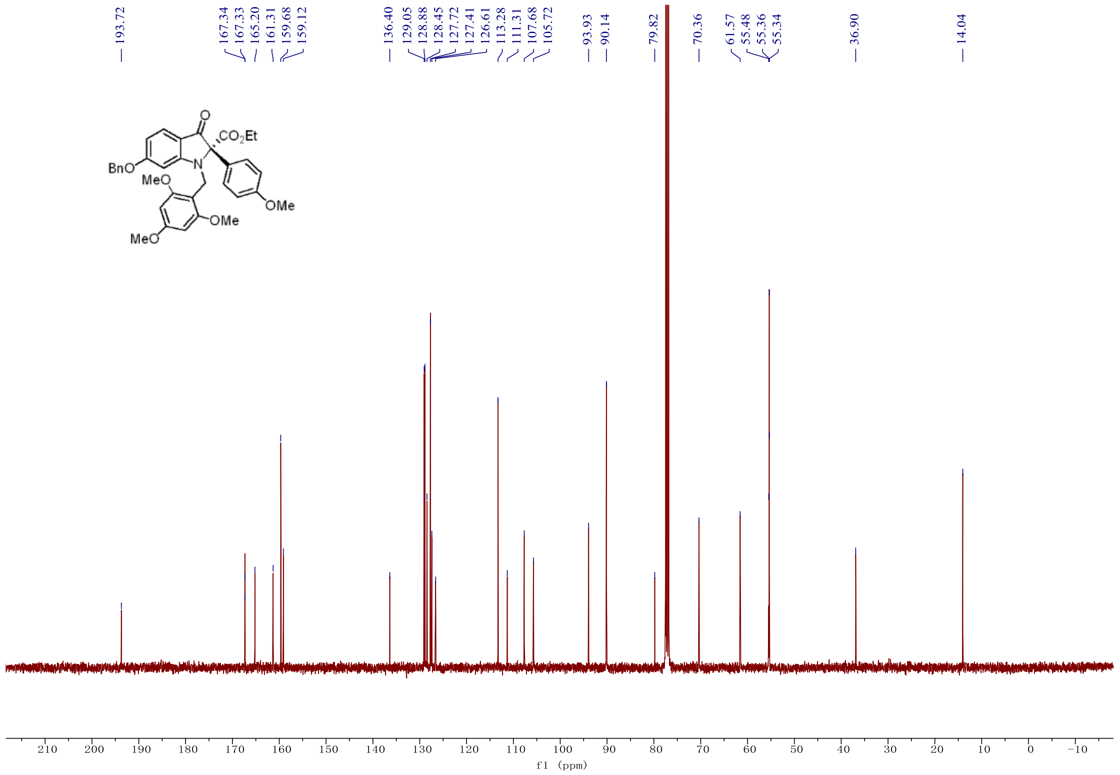
193.72
167.34 167.33 165.20 161.31 159.68 159.12
136.40 129.05 128.88 128.45 127.72 127.41 126.61 113.28 111.31 107.68 105.72
93.93 90.14
79.82
70.36
61.57 55.48 55.36 55.34
36.90
14.04
CO2Et
BnO
MeO
N
OMe
MeO
OMe
f1 (ppm)

3d

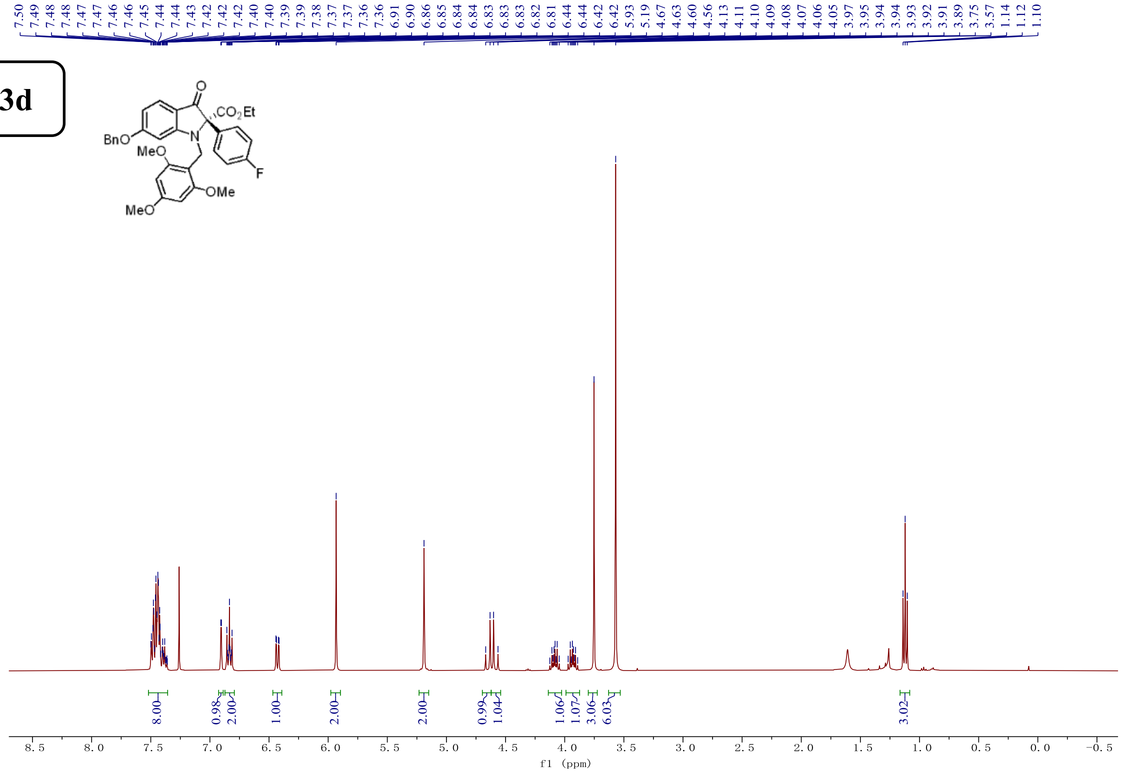
7.50 7.49 7.48 7.47 7.47 7.46 7.45 7.44 7.44 7.43 7.42 7.42 7.42 7.40 7.40 7.39 7.39 7.38 7.37 7.37 7.36 7.36 6.91 6.90 6.86 6.85 6.84 6.84 6.83 6.83 6.83 6.82 6.81 6.44 6.44 6.42 6.42 5.93 5.19 4.67 4.63 4.60 4.56 4.13 4.11 4.10 4.09 4.08 4.07 4.06 4.05 3.97 3.95 3.94 3.94 3.93 3.92 3.91 3.89 3.75 3.57 1.14 1.12 1.10
O
CO2Et
BnO
N
MeO
F
OMe
MeO
8.00
0.98
2.00
1.00
2.00
2.00
0.99
1.04
1.06
1.07
3.06
6.03
3.02
8.5 8.0 7.5 7.0 6.5 6.0 5.5 5.0 4.5 4.0 3.5 3.0 2.5 2.0 1.5 1.0 0.5 0.0 -0.5
f1 (ppm)

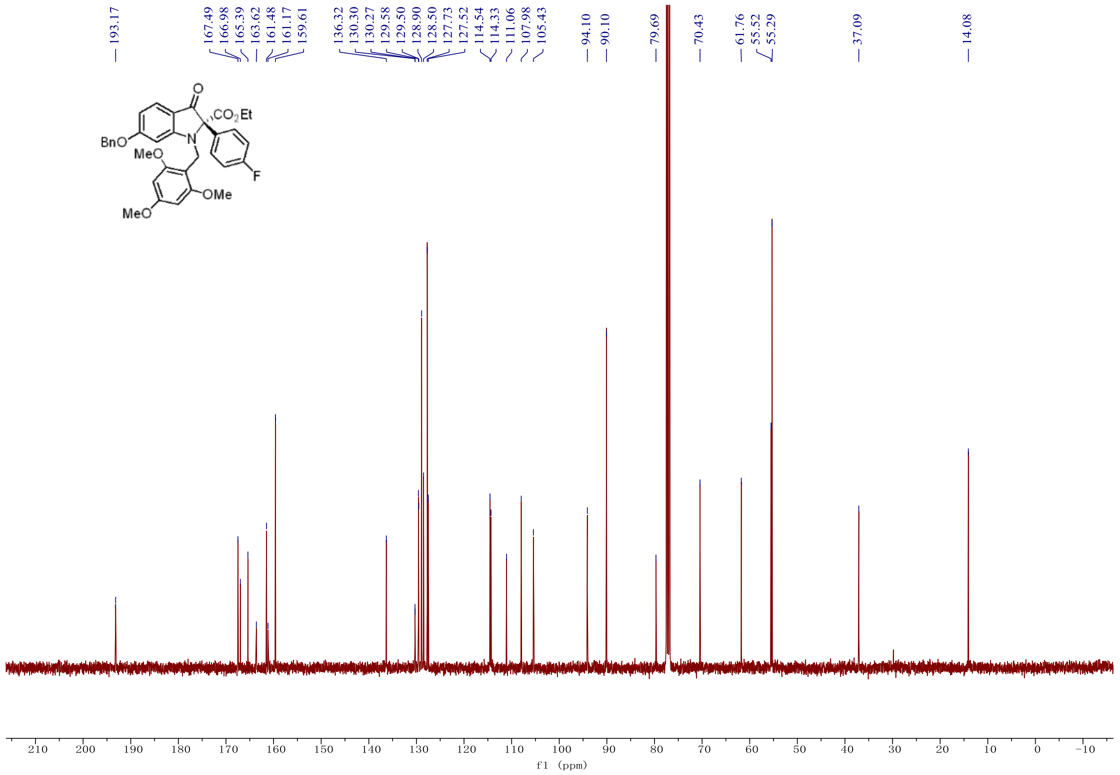
193.17
167.49 166.98 165.39 163.62 161.48 161.17 159.61
136.32 130.30 130.27 129.58 129.50 128.90 128.50 127.73 127.52 114.54 114.33 111.06 107.98 105.43
94.10 90.10 79.69 70.43 61.76 55.52 55.29 37.09 14.08
O
CO2Et
BnO
N
MeO
F
OMe
MeO
210 200 190 180 170 160 150 140 130 120 110 100 90 80 70 60 50 40 30 20 10 0 -10
f1 (ppm)

3e

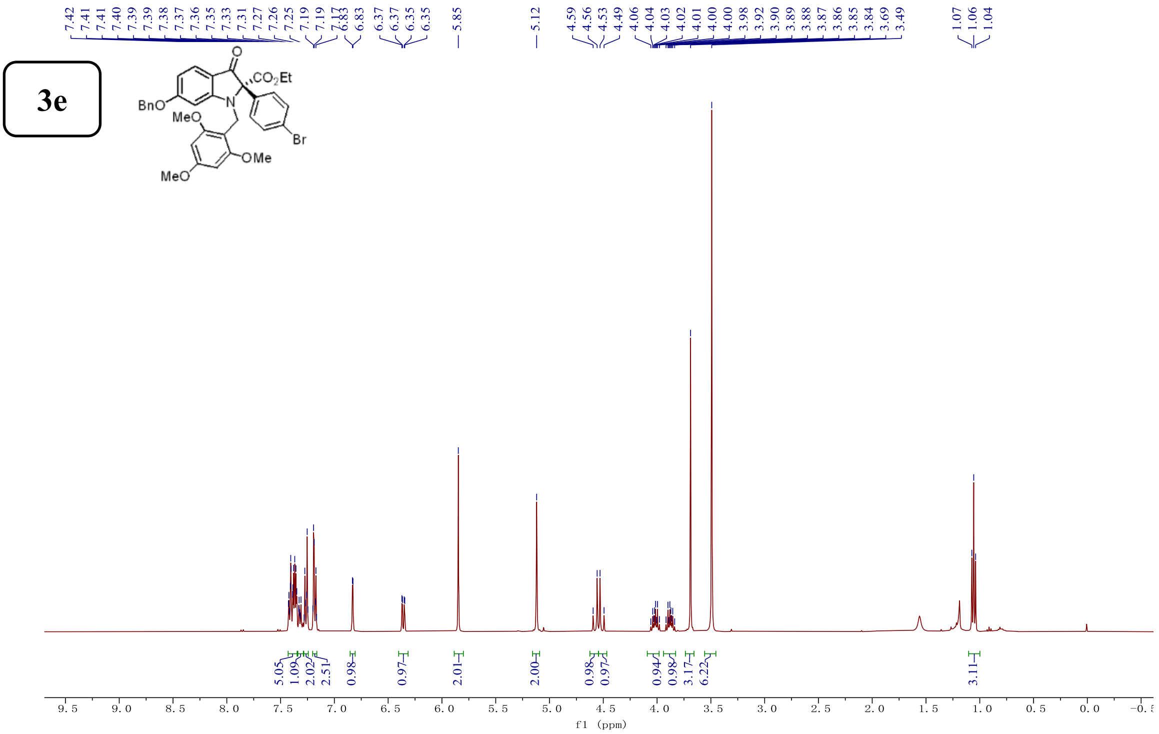
7.42 7.41 7.41 7.40 7.39 7.39 7.38 7.37 7.36 7.35 7.33 7.31 7.27 7.26 7.25 7.19 7.19 7.17 6.83 6.37 6.37 6.35 6.35
5.85
5.12
4.59 4.56 4.53 4.49 4.06 4.04 4.03 4.02 4.01 4.00 4.00 3.98 3.92 3.90 3.89 3.88 3.87 3.86 3.85 3.84 3.69 3.49
1.07 1.06 1.04
BnO
CO2Et
N
MeO
OMe
MeO
Br
5.05 1.09 2.02 2.51 0.98 0.97 2.01 2.00 0.98 0.97 0.94 0.98 3.17 6.22 3.11
f1 (ppm)

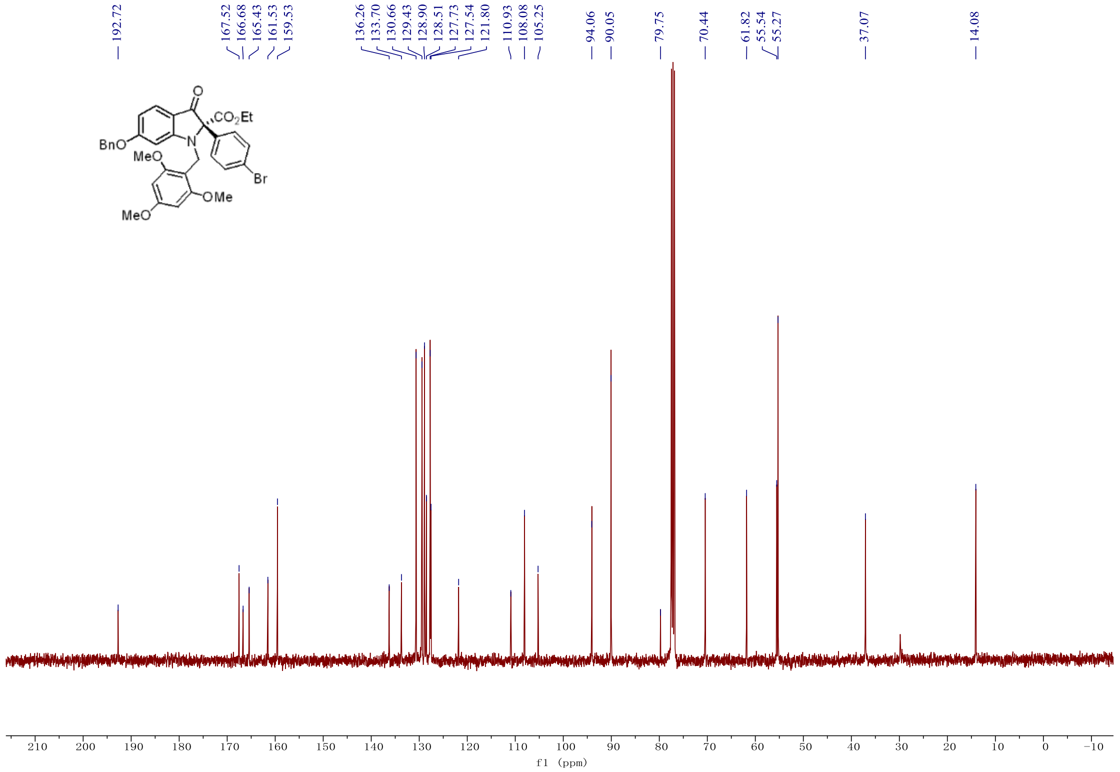
192.72
167.52 166.68 165.43 161.53 159.53
136.26 133.70 130.66 129.43 128.90 128.51 127.73 127.54 121.80
110.93 108.08 105.25
94.06 90.05
79.75
70.44
61.82 55.54 55.27
37.07
14.08
BnO
CO2Et
N
MeO
OMe
MeO
Br
f1 (ppm)

3f

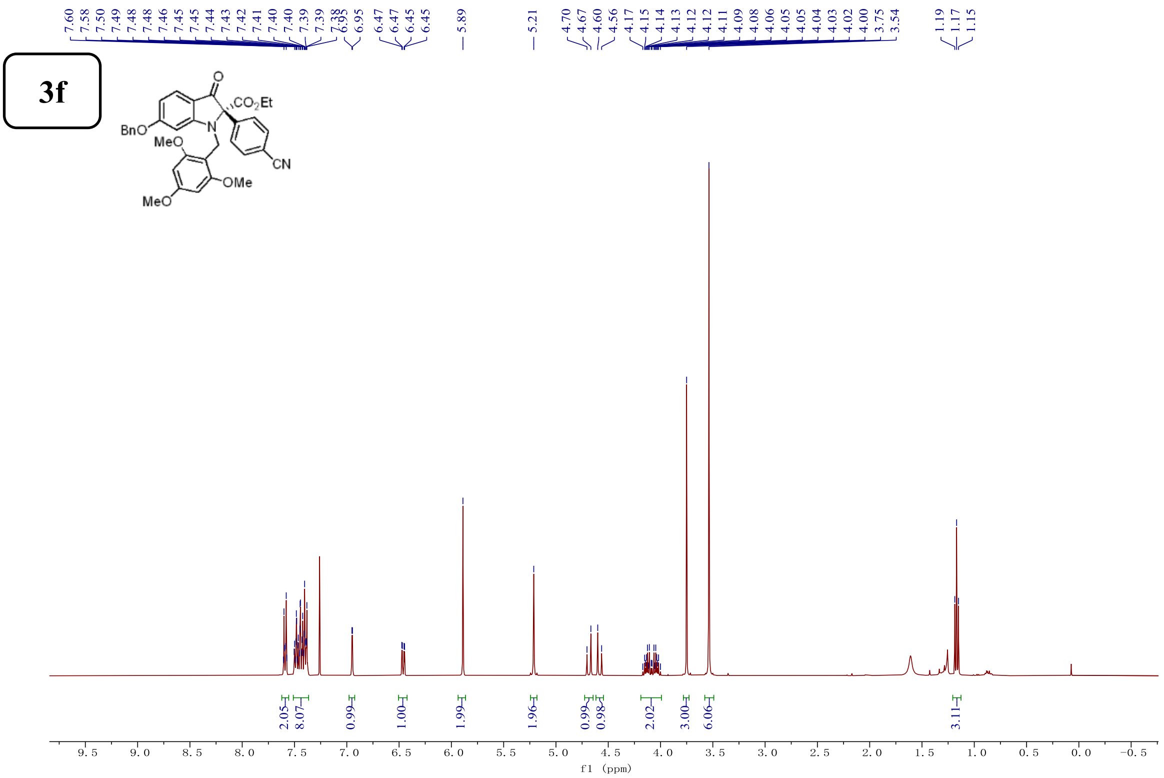

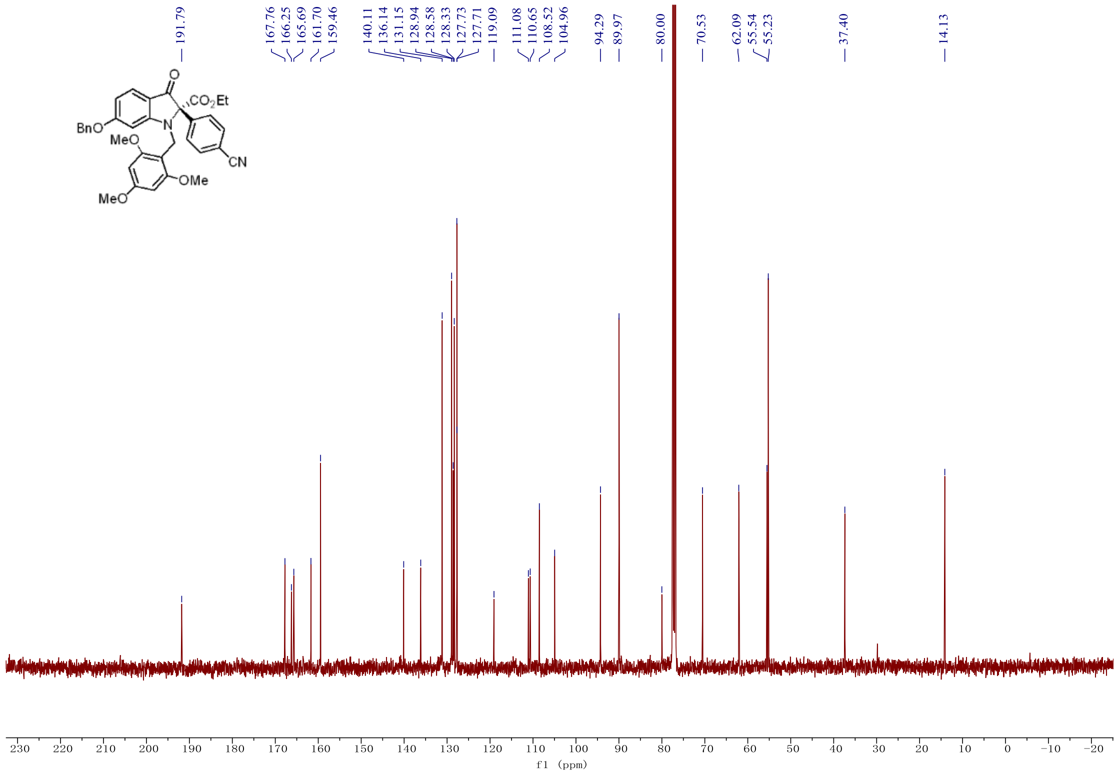

3g

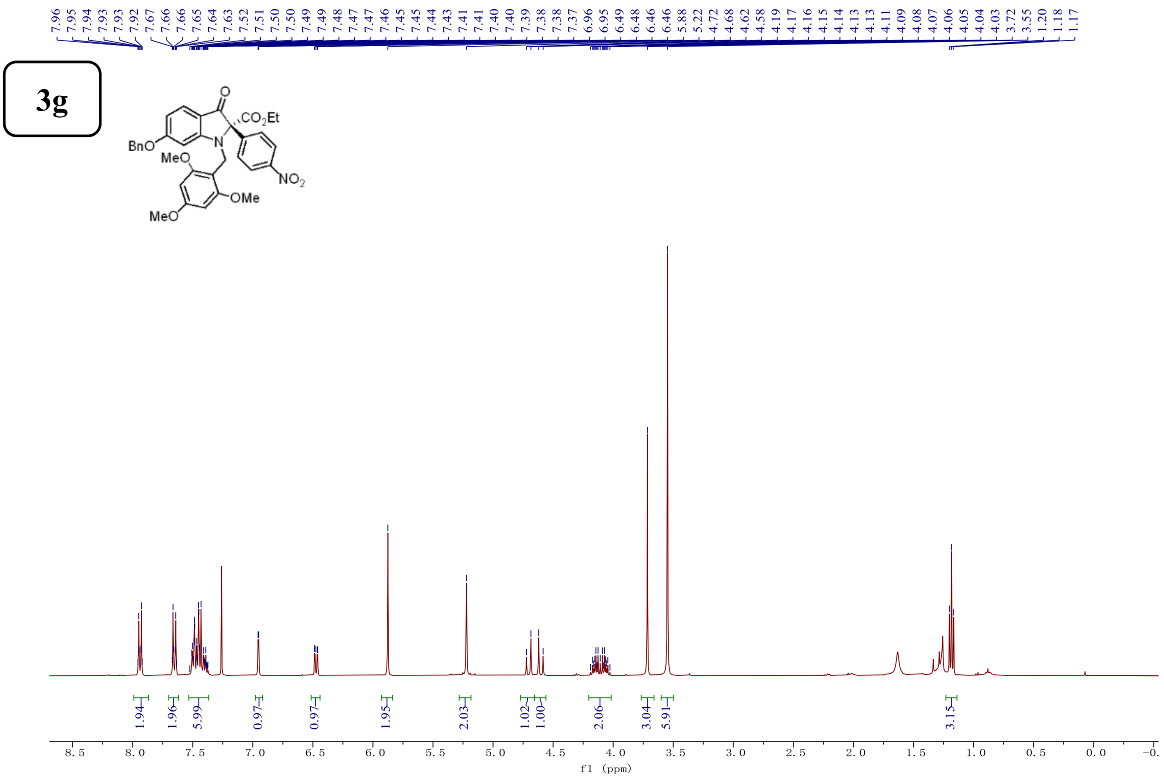

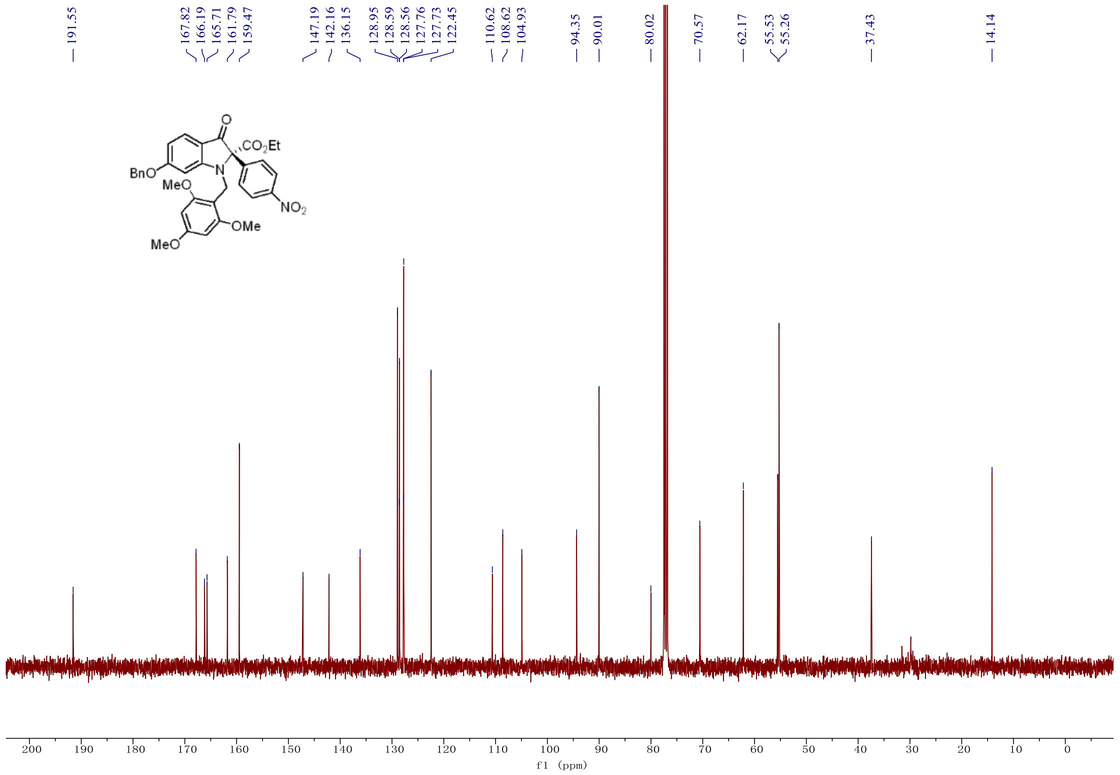

3h

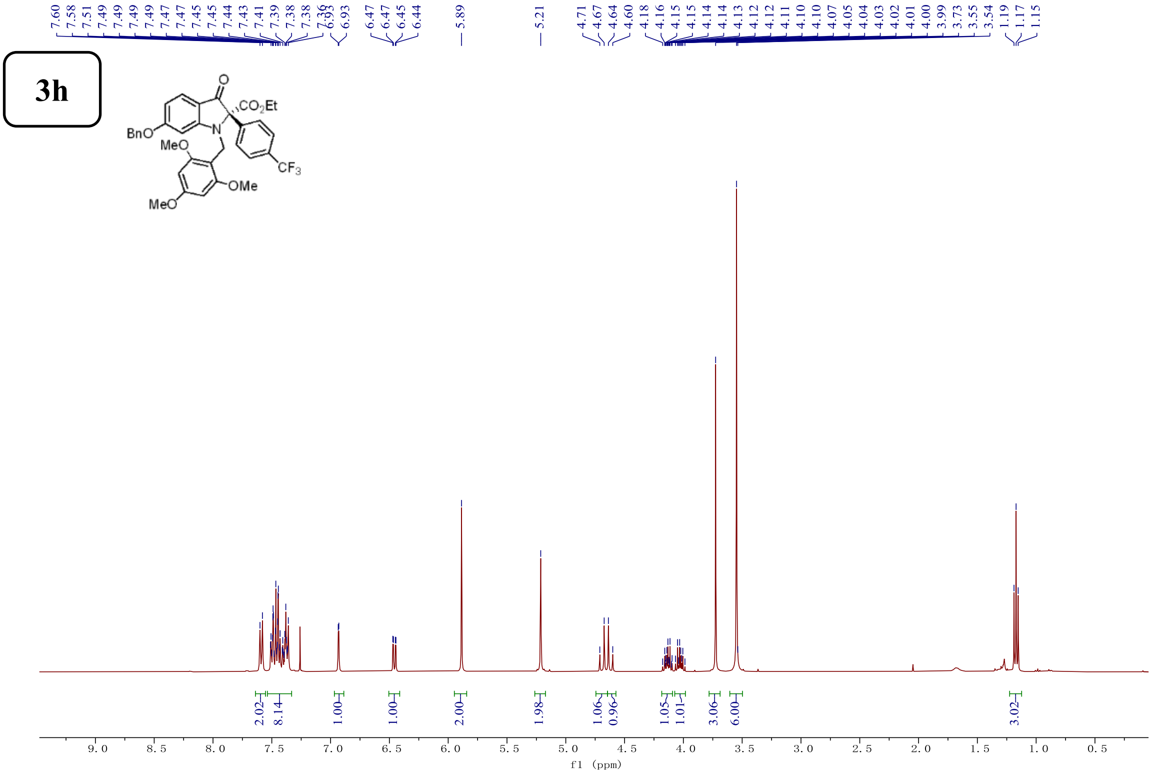

7.60
7.58
7.51
7.49
7.49
7.49
7.47
7.47
7.45
7.45
7.44
7.43
7.41
7.39
7.38
7.38
7.36
6.93
6.93
6.47
6.47
6.45
6.44
5.89
5.21
4.71
4.67
4.64
4.60
4.18
4.16
4.15
4.15
4.14
4.14
4.13
4.12
4.11
4.10
4.10
4.07
4.05
4.04
4.03
4.02
4.01
4.00
3.99
3.73
3.55
3.54
1.19
1.17
1.15
BnO
MeO
N
O
CO_2Et
CF_3
OMe
MeO
2.02
8.14
1.00
1.00
2.00
1.98
1.06
0.96
1.05
1.01
3.06
6.00
3.02
9.0
8.5
8.0
7.5
7.0
6.5
6.0
5.5
5.0
4.5
4.0
3.5
3.0
2.5
2.0
1.5
1.0
0.5
f1 (ppm)

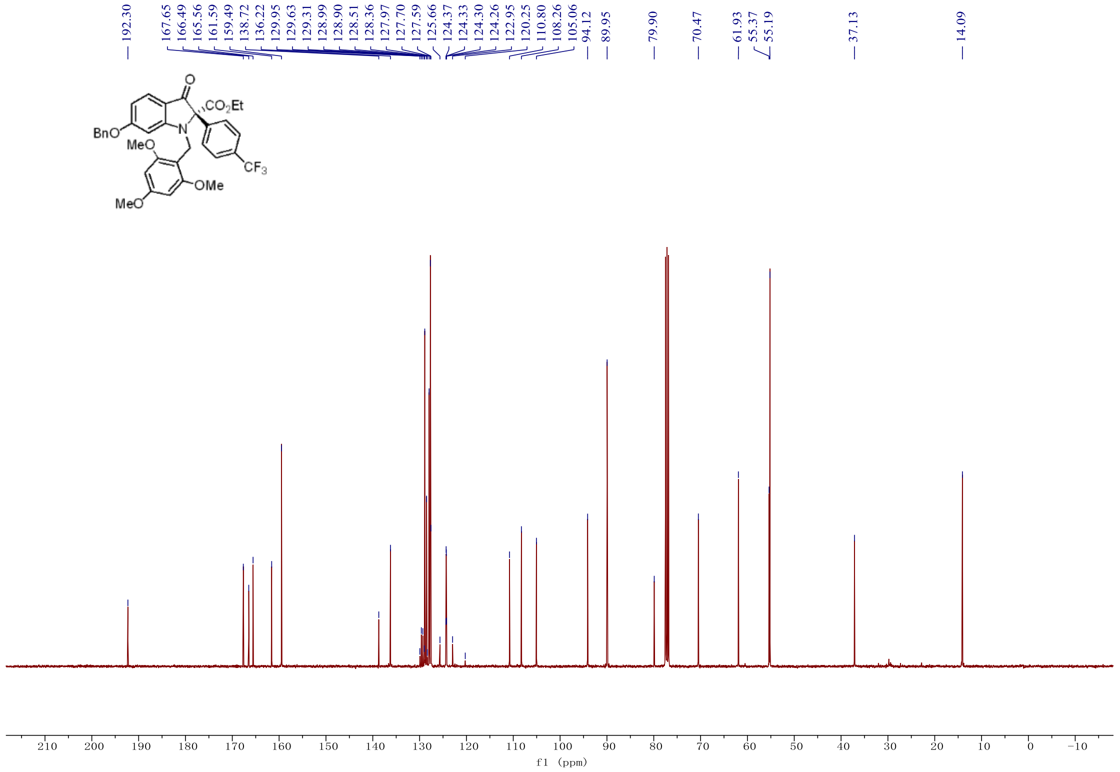

192.30
167.65
166.49
165.56
161.59
159.49
138.72
136.22
129.95
129.63
129.31
128.99
128.90
128.51
128.36
127.97
127.70
127.59
125.66
124.37
124.33
124.30
124.26
122.95
120.25
110.80
108.26
105.06
94.12
89.95
79.90
70.47
61.93
55.37
55.19
37.13
14.09
BnO
MeO
N
O
CO_2Et
CF_3
OMe
MeO
210
200
190
180
170
160
150
140
130
120
110
100
90
80
70
60
50
40
30
20
10
0
-10
f1 (ppm)

3i

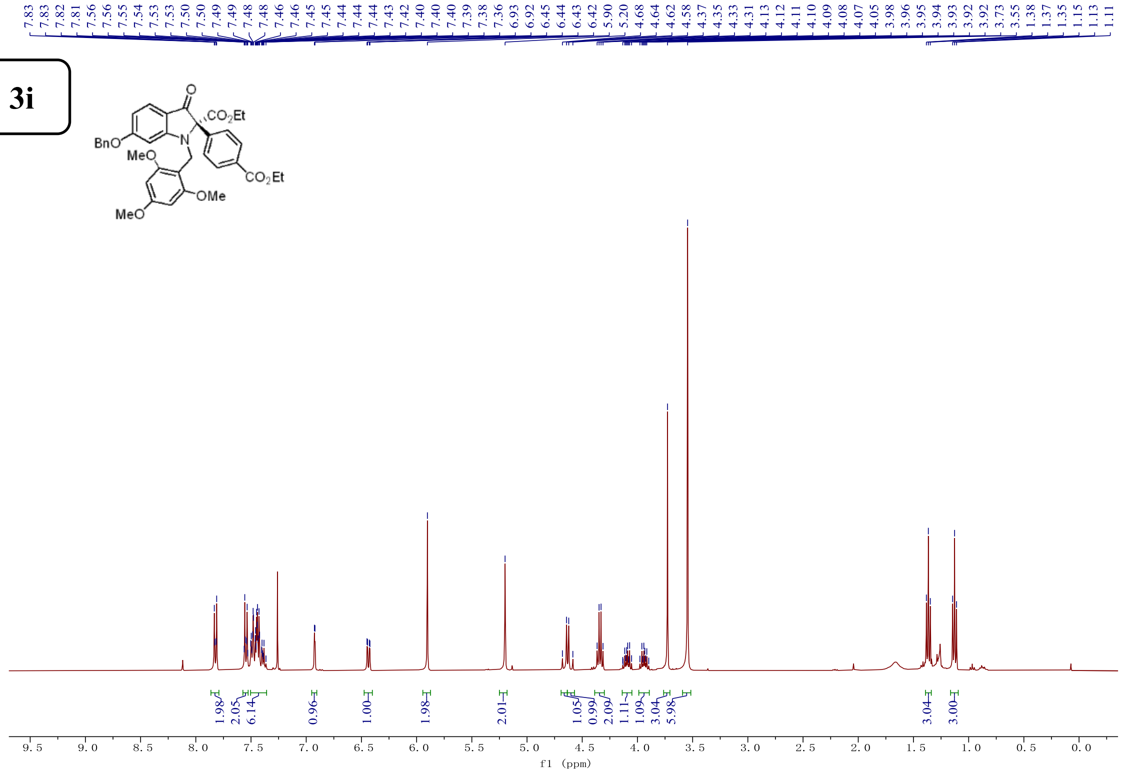
7.83 7.83 7.82 7.81 7.56 7.56 7.55 7.54 7.53 7.53 7.50 7.49 7.49 7.48 7.48 7.46 7.45 7.45 7.44 7.44 7.44 7.43 7.42 7.40 7.40 7.39 7.38 7.36 6.93 6.92 6.45 6.44 6.43 6.42 5.90 5.20 4.68 4.64 4.62 4.58 4.37 4.35 4.33 4.31 4.13 4.12 4.11 4.10 4.09 4.08 4.07 4.05 3.98 3.96 3.95 3.94 3.93 3.92 3.92 3.73 3.55 1.38 1.37 1.35 1.15 1.13 1.11
O
CO2Et
BnO
N
MeO
CO2Et
OMe
MeO
1.98 2.05 6.14 0.96 1.00 1.98 2.01 1.05 0.99 2.09 1.11 1.09 3.04 5.98 3.04 3.00
9.5 9.0 8.5 8.0 7.5 7.0 6.5 6.0 5.5 5.0 4.5 4.0 3.5 3.0 2.5 2.0 1.5 1.0 0.5 0.0
f1 (ppm)

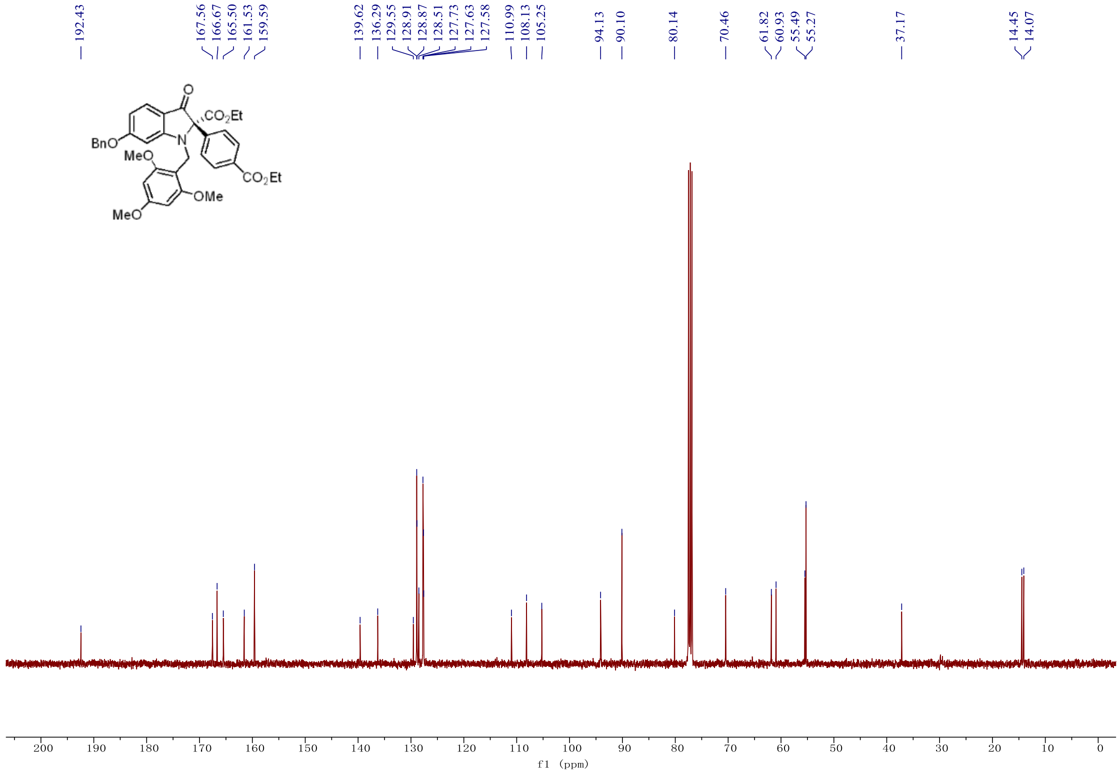
192.43
167.56 166.67 165.50 161.53 159.59
139.62 136.29 129.55 128.91 128.87 128.51 127.73 127.63 127.58
110.99 108.13 105.25
94.13 90.10 80.14 70.46 61.82 60.93 55.49 55.27 37.17 14.45 14.07
O
CO2Et
BnO
N
MeO
CO2Et
OMe
MeO
200 190 180 170 160 150 140 130 120 110 100 90 80 70 60 50 40 30 20 10 0
f1 (ppm)

3j

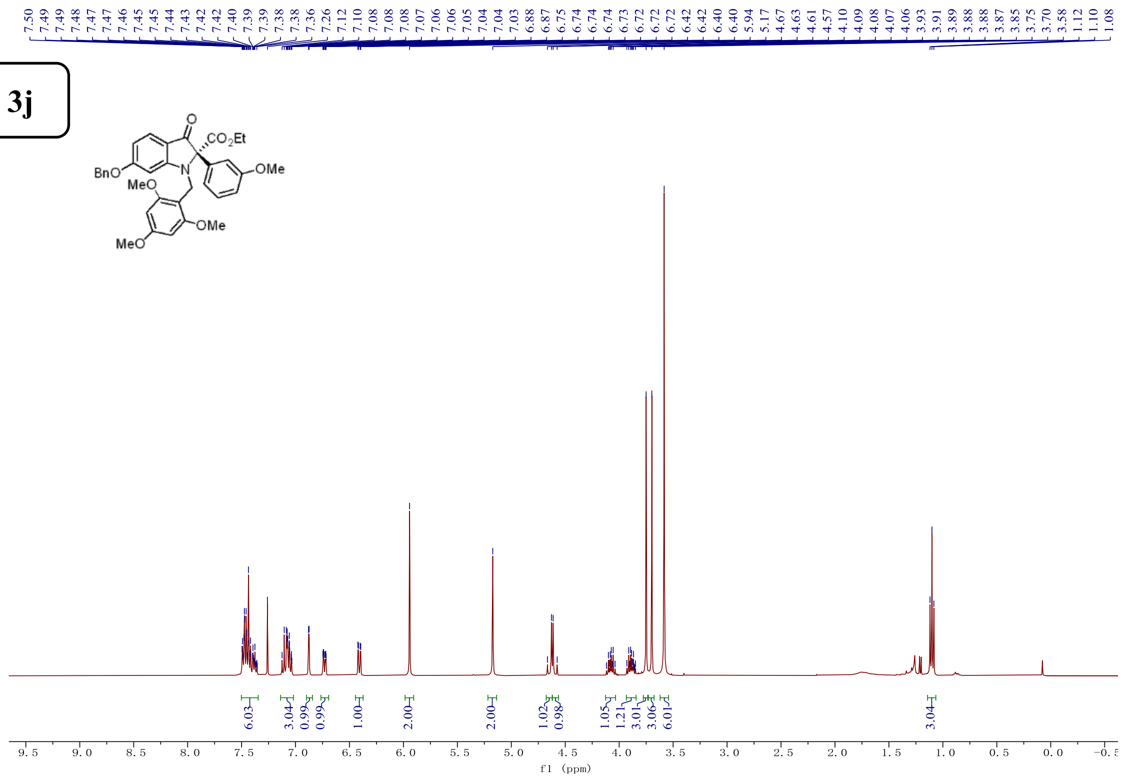
7.50 7.49 7.49 7.48 7.47 7.47 7.46 7.45 7.45 7.44 7.43 7.42 7.42 7.40 7.39 7.39 7.38 7.38 7.36 7.26 7.12 7.10 7.08 7.08 7.07 7.06 7.05 7.04 7.04 7.03 6.88 6.87 6.75 6.74 6.74 6.73 6.72 6.72 6.72 6.42 6.42 6.40 6.40 5.94 5.17 4.67 4.63 4.61 4.57 4.10 4.09 4.08 4.07 4.06 3.93 3.91 3.89 3.88 3.88 3.87 3.85 3.75 3.70 3.58 1.12 1.10 1.08
O
CO_2Et
BnO
N
OMe
MeO
OMe
MeO
6.03 3.04 0.99 0.99 1.00 2.00 2.00 1.02 0.98 1.05 1.21 3.01 3.06 6.01 3.04
f1 (ppm)

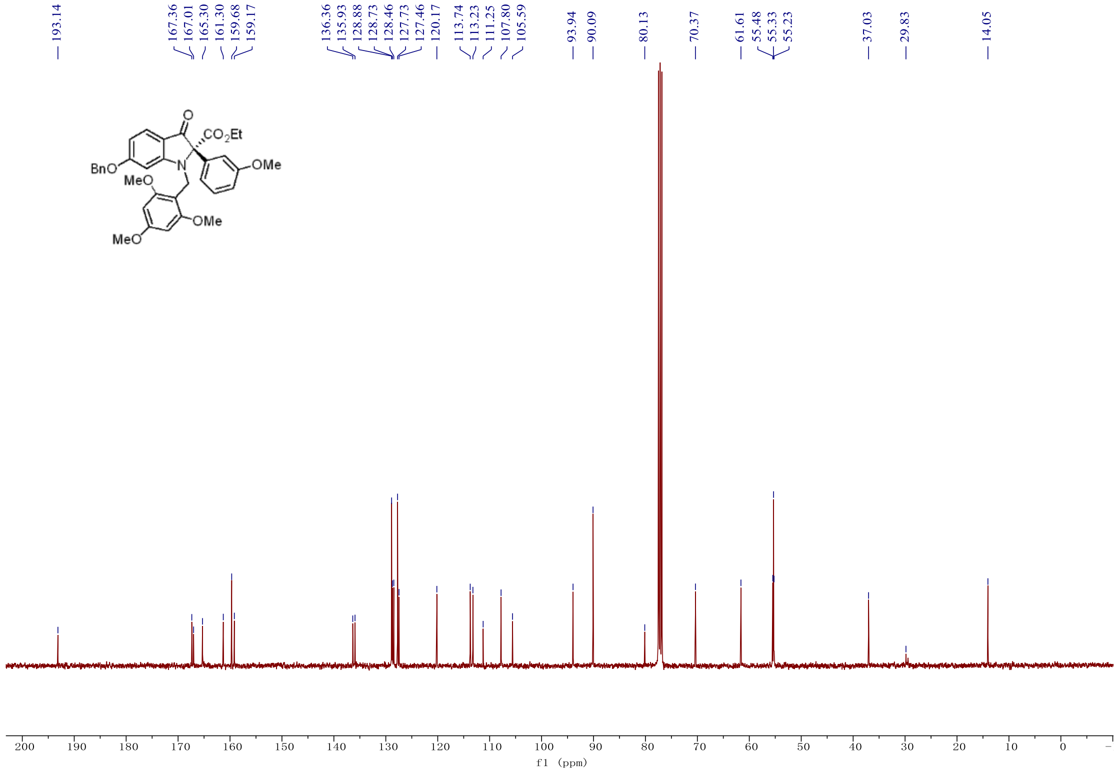
193.14 167.36 167.01 165.30 161.30 159.68 159.17 136.36 135.93 128.88 128.73 128.46 127.73 127.46 120.17 113.74 113.23 111.25 107.80 105.59 93.94 90.09 80.13 70.37 61.61 55.48 55.33 55.23 37.03 29.83 14.05
O
CO_2Et
BnO
N
OMe
MeO
OMe
MeO
f1 (ppm)

3k

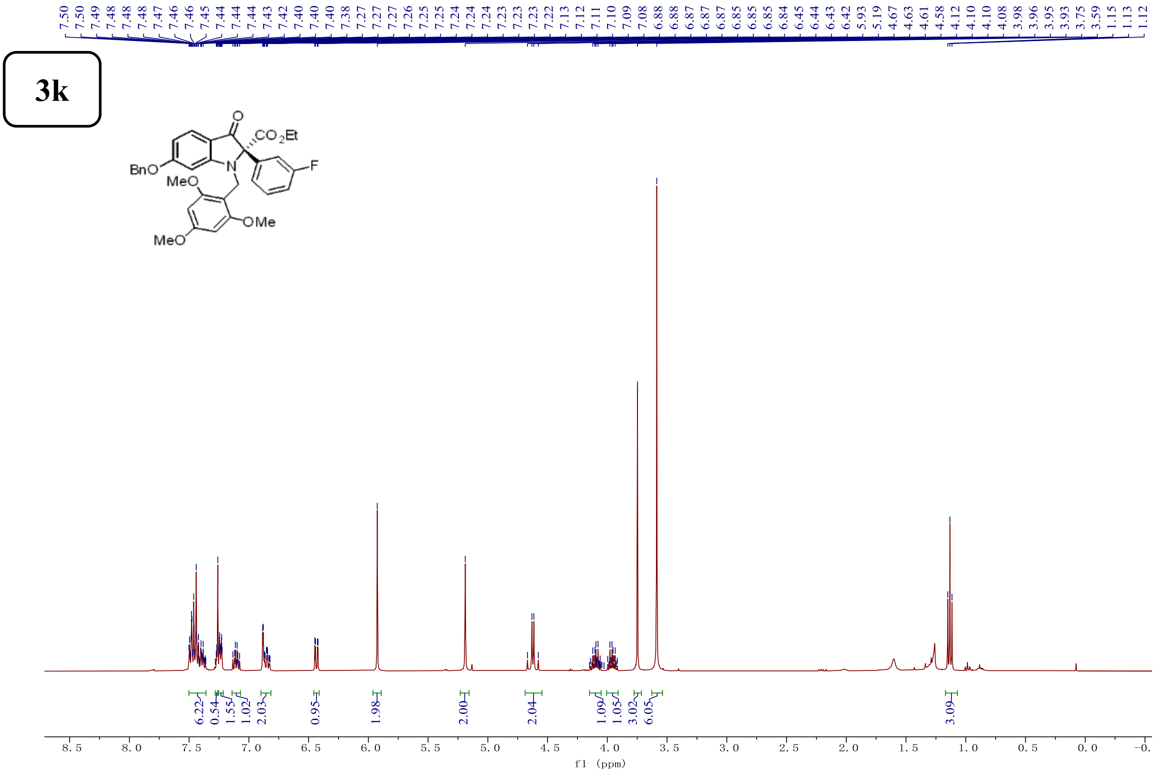
7.50 7.50 7.49 7.48 7.48 7.47 7.46 7.46 7.45 7.44 7.44 7.44 7.43 7.42 7.40 7.40 7.40 7.38 7.27 7.27 7.26 7.25 7.25 7.24 7.24 7.24 7.23 7.23 7.23 7.22 7.13 7.12 7.11 7.10 7.09 7.08 6.88 6.88 6.87 6.87 6.87 6.85 6.85 6.85 6.84 6.45 6.44 6.43 6.42 5.93 5.19 4.67 4.63 4.61 4.58 4.12 4.10 4.10 4.08 3.98 3.96 3.95 3.93 3.75 3.59 1.15 1.13 1.12
BnO
MeO
MeO
OMe
CO2Et
F
N
O
6.22 0.54 1.55 1.02 2.03 0.95 1.98 2.00 2.04 1.09 1.05 3.02 6.05 3.09
8.5 8.0 7.5 7.0 6.5 6.0 5.5 5.0 4.5 4.0 3.5 3.0 2.5 2.0 1.5 1.0 0.5 0.0 -0.
f1 (ppm)

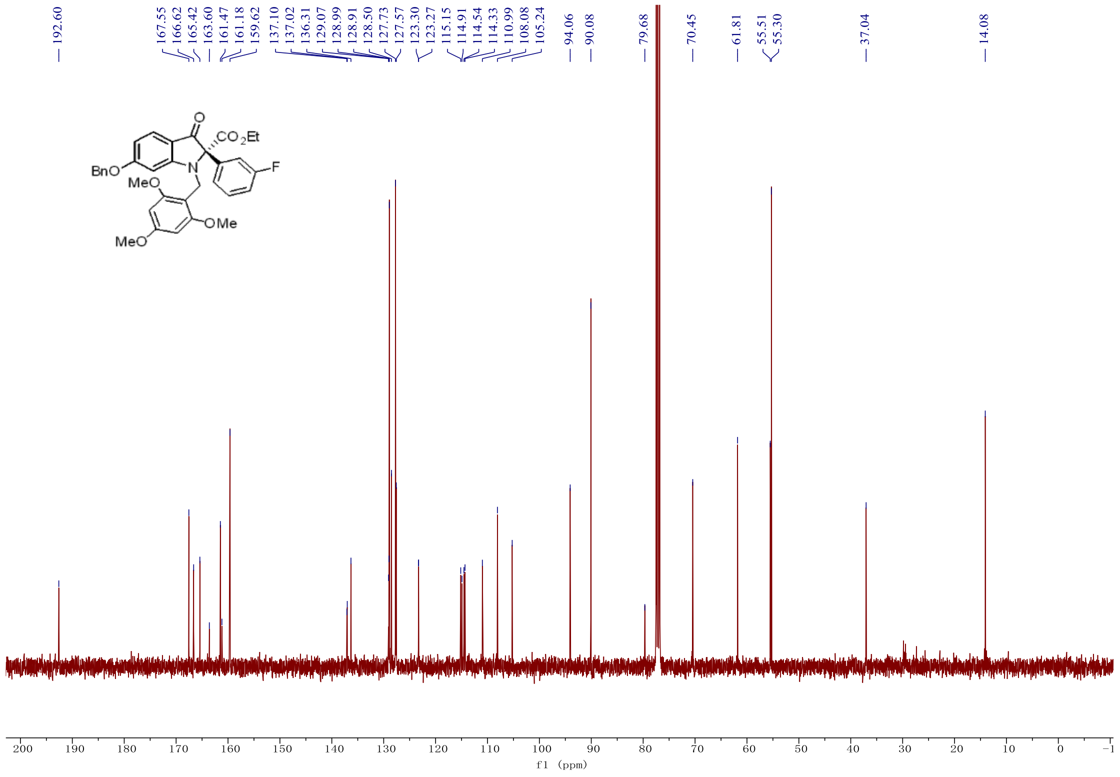
192.60
167.55 166.62 165.42 163.60 161.47 161.18 159.62 137.10 137.02 136.31 129.07 128.99 128.91 128.50 127.73 127.57 123.30 123.27 115.15 114.91 114.54 114.33 110.99 108.08 105.24
94.06 90.08 79.68 70.45 61.81 55.51 55.30 37.04 14.08
BnO
MeO
MeO
OMe
CO2Et
F
N
O
200 190 180 170 160 150 140 130 120 110 100 90 80 70 60 50 40 30 20 10 0 -1
f1 (ppm)

3l

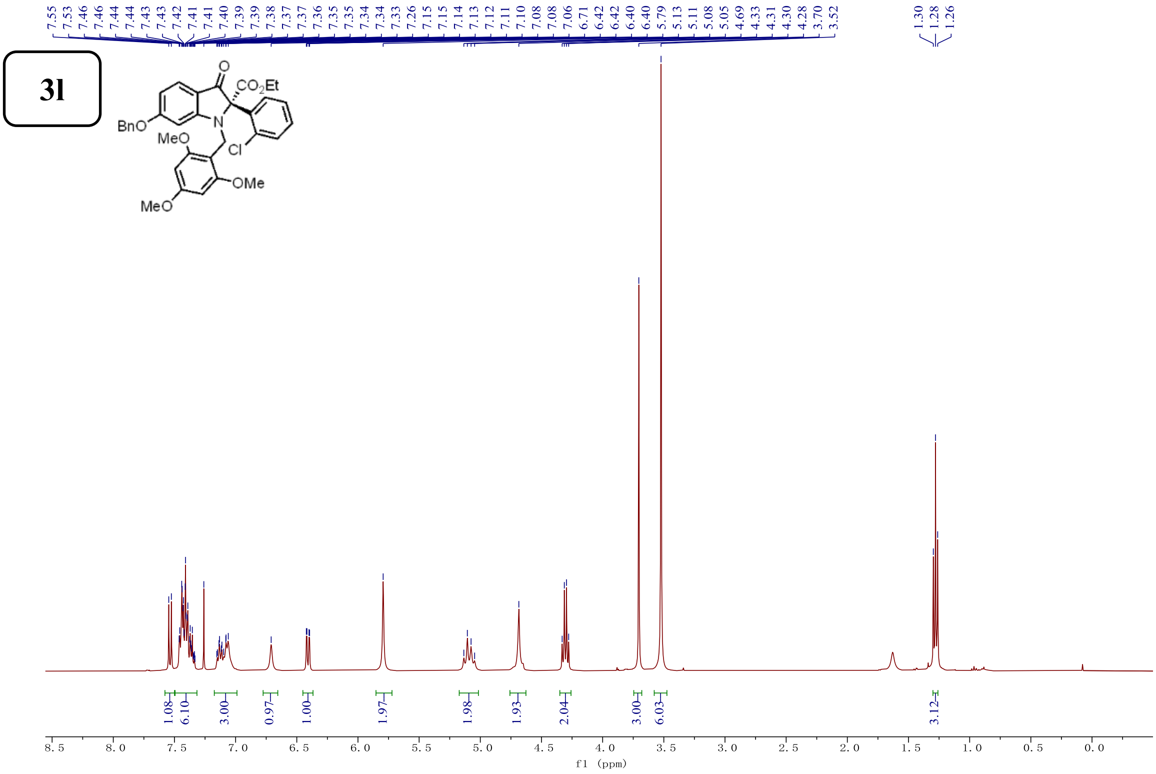

7.55 7.53 7.46 7.46 7.44 7.44 7.43 7.43 7.42 7.42 7.41 7.41 7.40 7.39 7.39 7.38 7.37 7.37 7.36 7.35 7.35 7.34 7.34 7.33 7.26 7.15 7.15 7.14 7.13 7.13 7.12 7.11 7.10 7.08 7.08 7.06 6.71 6.42 6.42 6.40 6.40 5.79 5.13 5.11 5.08 5.05 4.69 4.33 4.31 4.30 4.28 3.70 3.52
1.30 1.28 1.26
CO2Et
BnO
N
Cl
MeO
OMe
MeO
1.08 6.10 3.00 0.97 1.00 1.97 1.98 1.93 2.04 3.00 6.03 3.12
f1 (ppm)

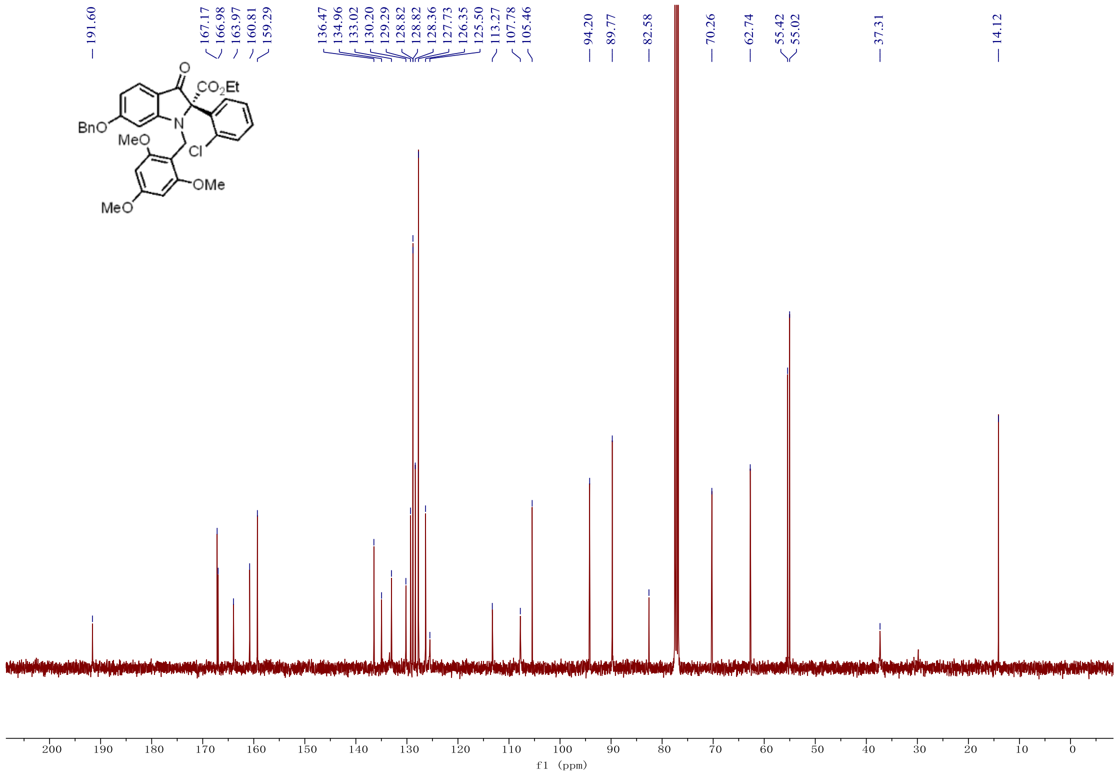

191.60
167.17 166.98 163.97 160.81 159.29
136.47 134.96 133.02 130.20 129.29 128.82 128.82 128.36 127.73 126.35 125.50 113.27 107.78 105.46
94.20 89.77 82.58
70.26 62.74 55.42 55.02
37.31
14.12
CO2Et
BnO
N
Cl
MeO
OMe
MeO
f1 (ppm)

3m

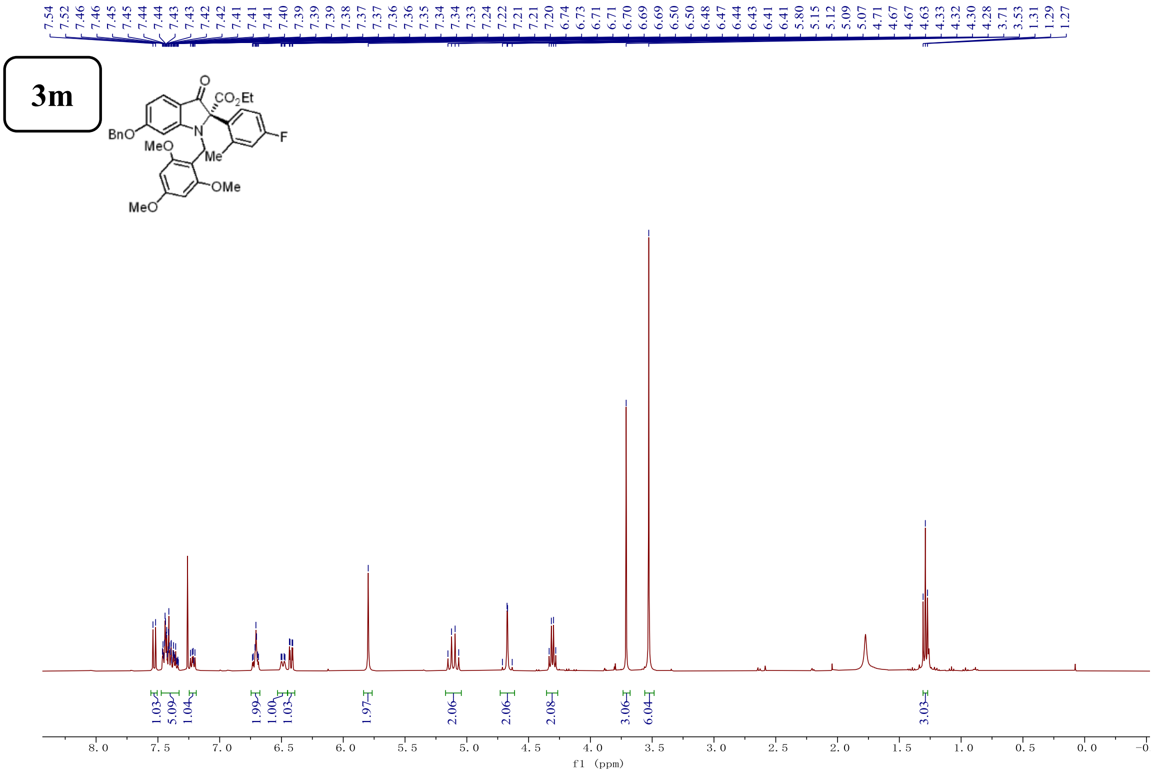
7.54 7.52 7.46 7.45 7.45 7.44 7.44 7.43 7.43 7.42 7.42 7.41 7.41 7.41 7.40 7.39 7.39 7.38 7.37 7.37 7.36 7.35 7.34 7.34 7.33 7.24 7.22 7.21 7.21 7.20 6.74 6.73 6.71 6.71 6.70 6.69 6.69 6.50 6.50 6.48 6.47 6.44 6.43 6.41 6.41 5.80 5.15 5.12 5.09 5.07 4.71 4.67 4.67 4.63 4.33 4.32 4.30 4.28 3.71 3.53 1.31 1.29 1.27
O
CO_2Et
BnO
N
F
MeO
Me
OMe
MeO
1.03 5.09 1.04 1.99 1.00 1.03 1.97 2.06 2.06 2.08 3.06 6.04 3.03
8.0 7.5 7.0 6.5 6.0 5.5 5.0 4.5 4.0 3.5 3.0 2.5 2.0 1.5 1.0 0.5 0.0
f1 (ppm)

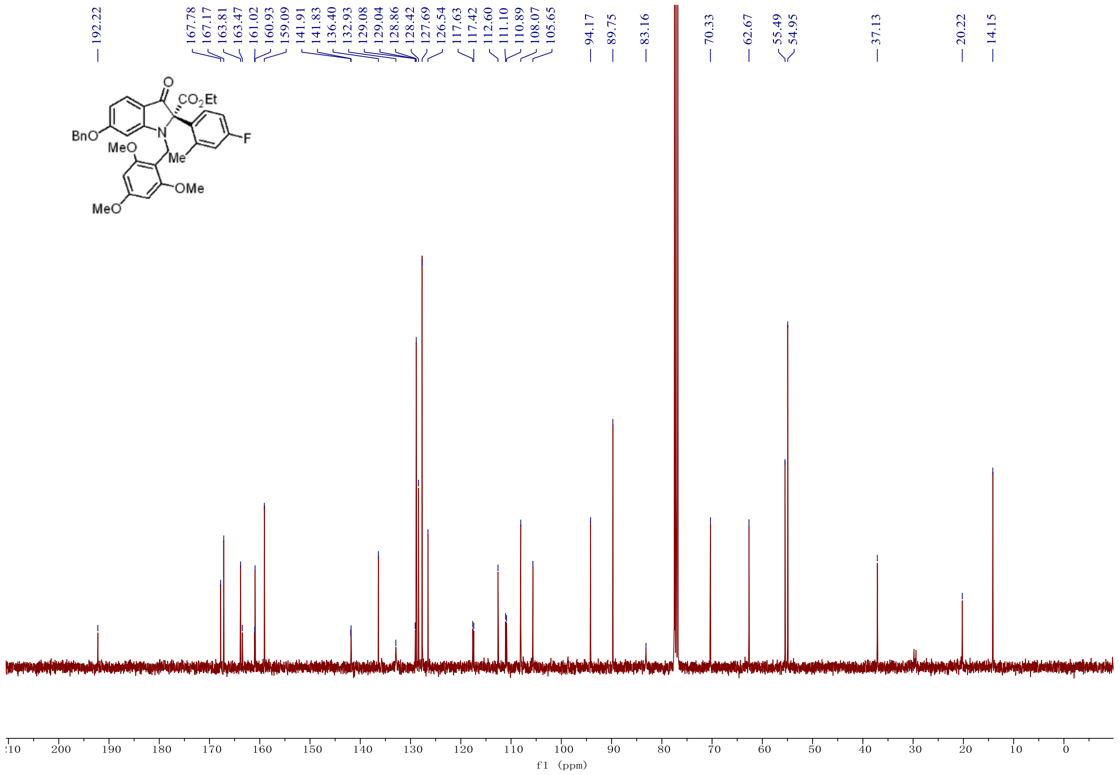
192.22
167.78 167.17 163.81 163.47 161.02 160.93 159.09 141.91 141.83 136.40 132.93 129.08 129.04 128.86 128.42 127.69 126.54 117.63 117.42 112.60 111.10 110.89 108.07 105.65
94.17 89.75 83.16 70.33 62.67 55.49 54.95 37.13 20.22 14.15
O
CO_2Et
BnO
N
F
MeO
Me
OMe
MeO
200 190 180 170 160 150 140 130 120 110 100 90 80 70 60 50 40 30 20 10 0
f1 (ppm)

3n

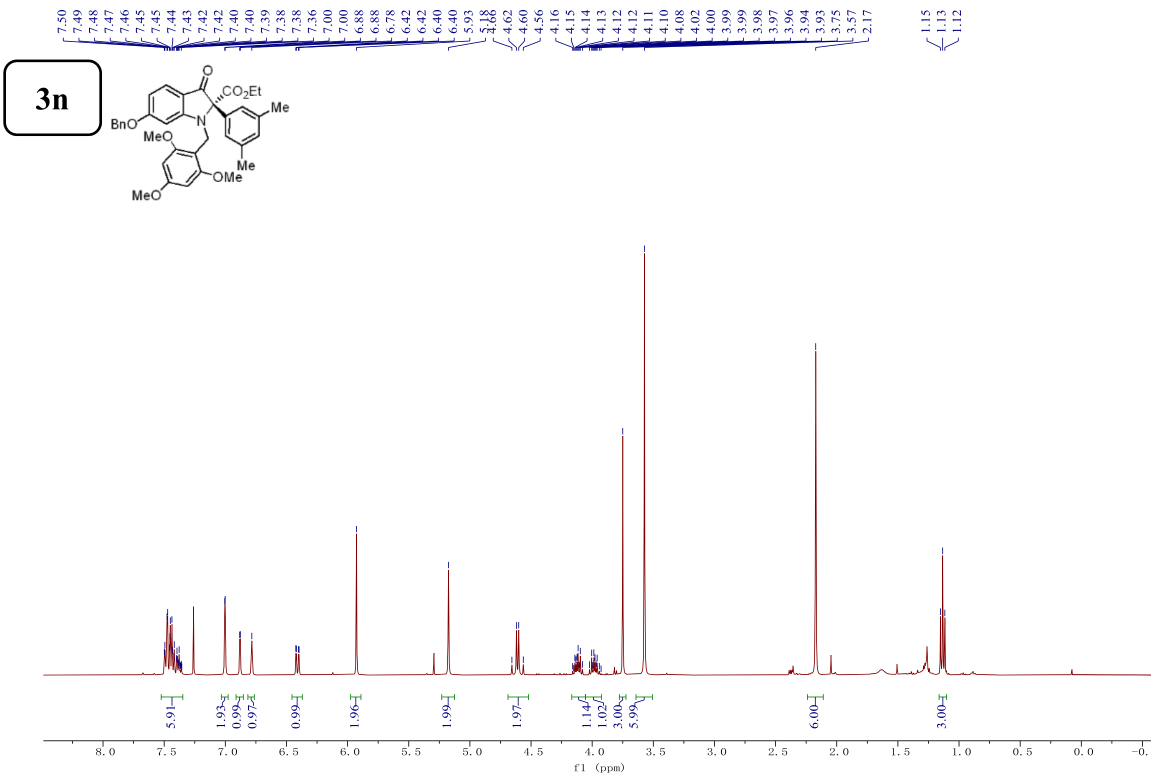

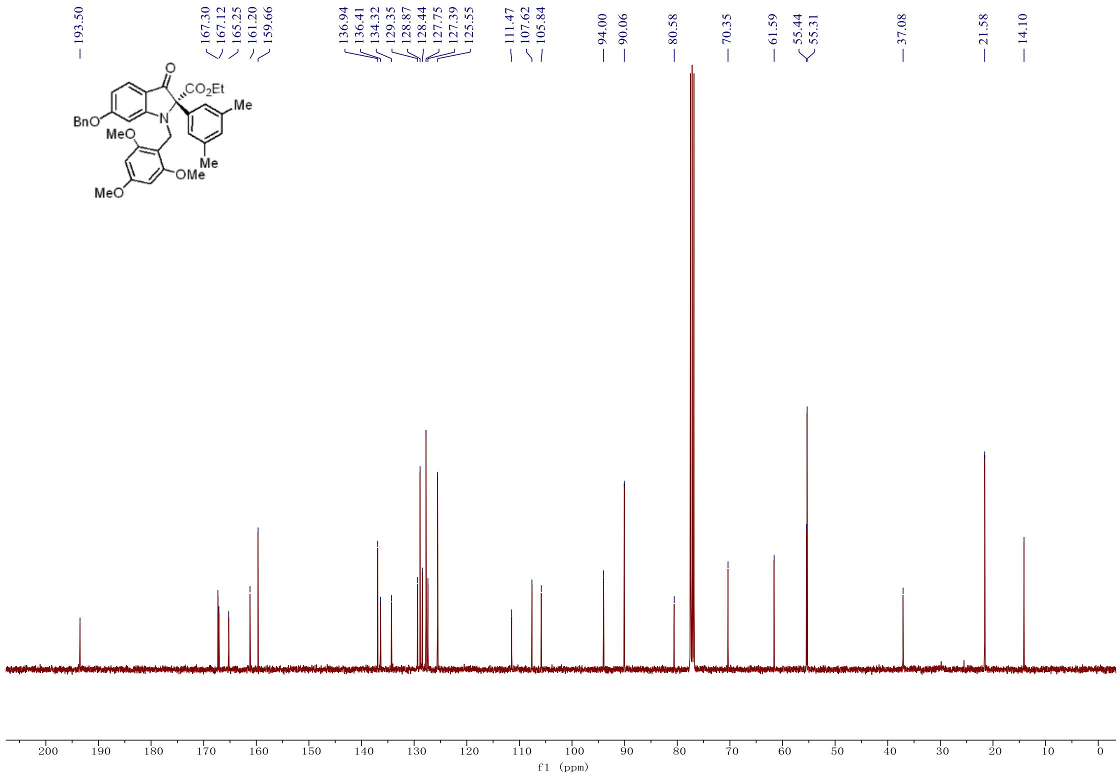

3o

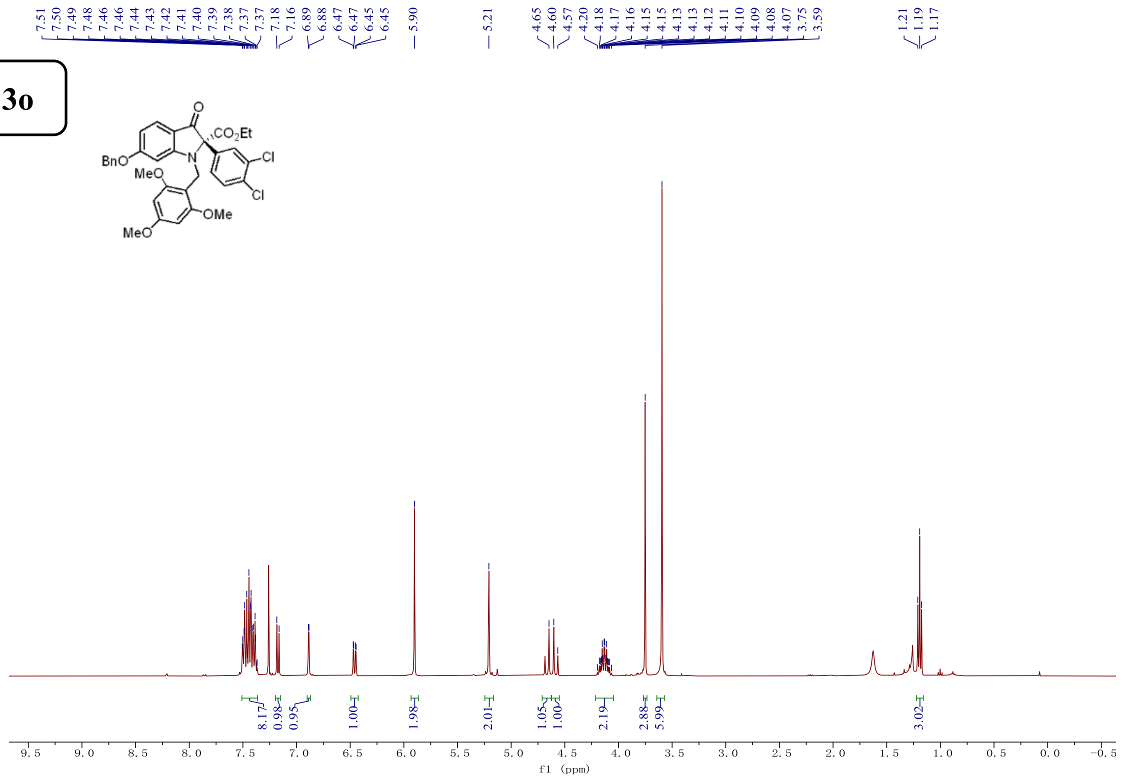

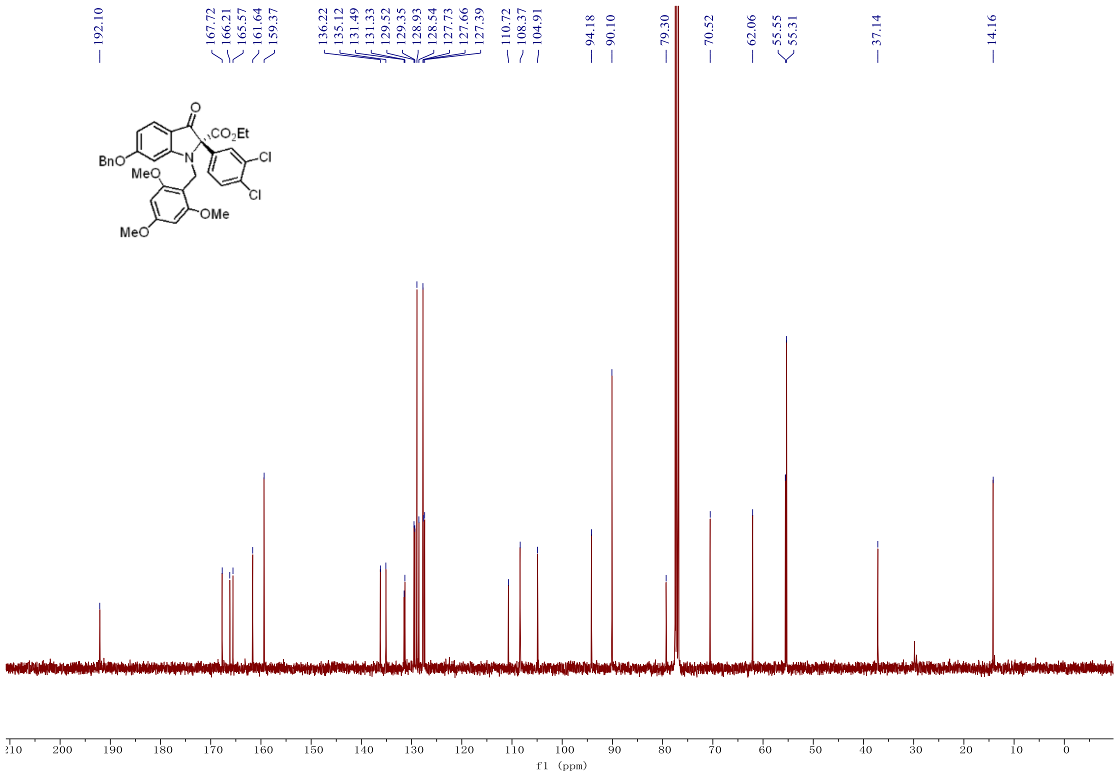

3p

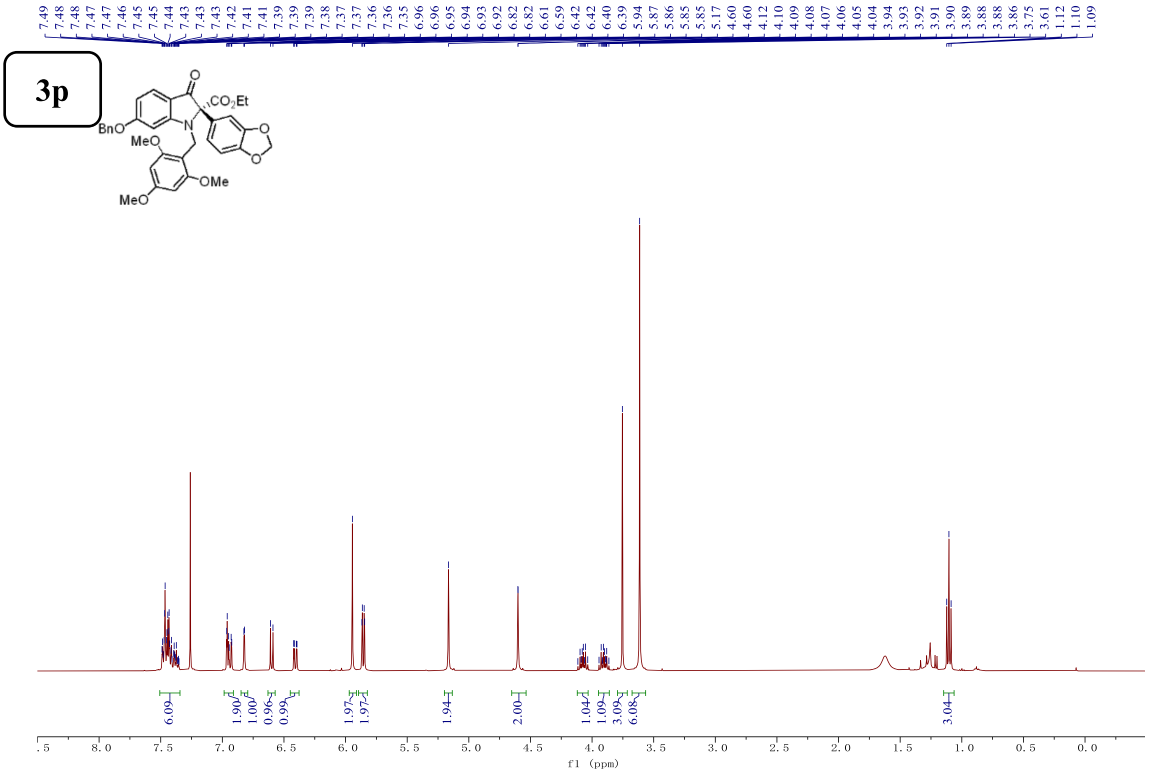
7.49 7.48 7.48 7.47 7.47 7.46 7.45 7.45 7.44 7.43 7.43 7.43 7.42 7.41 7.41 7.39 7.39 7.39 7.38 7.37 7.37 7.36 7.35 6.96 6.96 6.95 6.94 6.93 6.92 6.82 6.82 6.61 6.59 6.42 6.42 6.40 6.39 5.94 5.87 5.86 5.85 5.85 5.17 4.60 4.60 4.12 4.10 4.09 4.08 4.07 4.06 4.05 4.04 3.94 3.93 3.92 3.91 3.90 3.89 3.88 3.88 3.86 3.75 3.61 1.12 1.10 1.09
BnO
MeO
OMe
MeO
N
O
CO2Et
6.09
1.90
1.00
0.96
0.99
1.97
1.97
1.94
2.00
1.04
1.09
3.09
6.08
3.04
f1 (ppm)

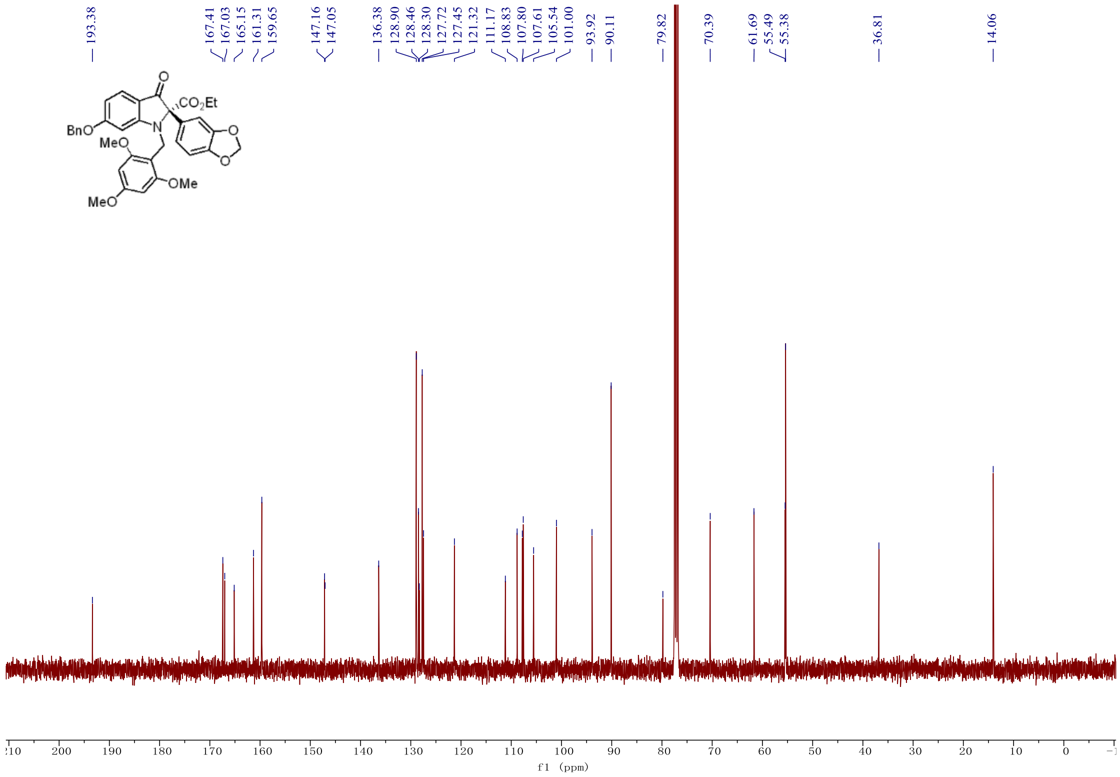
193.38
167.41 167.03 165.15 161.31 159.65
147.16 147.05
136.38 128.90 128.46 128.30 127.72 127.45 121.32 111.17 108.83 107.80 107.61 105.54 101.00
93.92 90.11
79.82
70.39
61.69 55.49 55.38
36.81
14.06
BnO
MeO
OMe
MeO
N
O
CO2Et
f1 (ppm)

3q

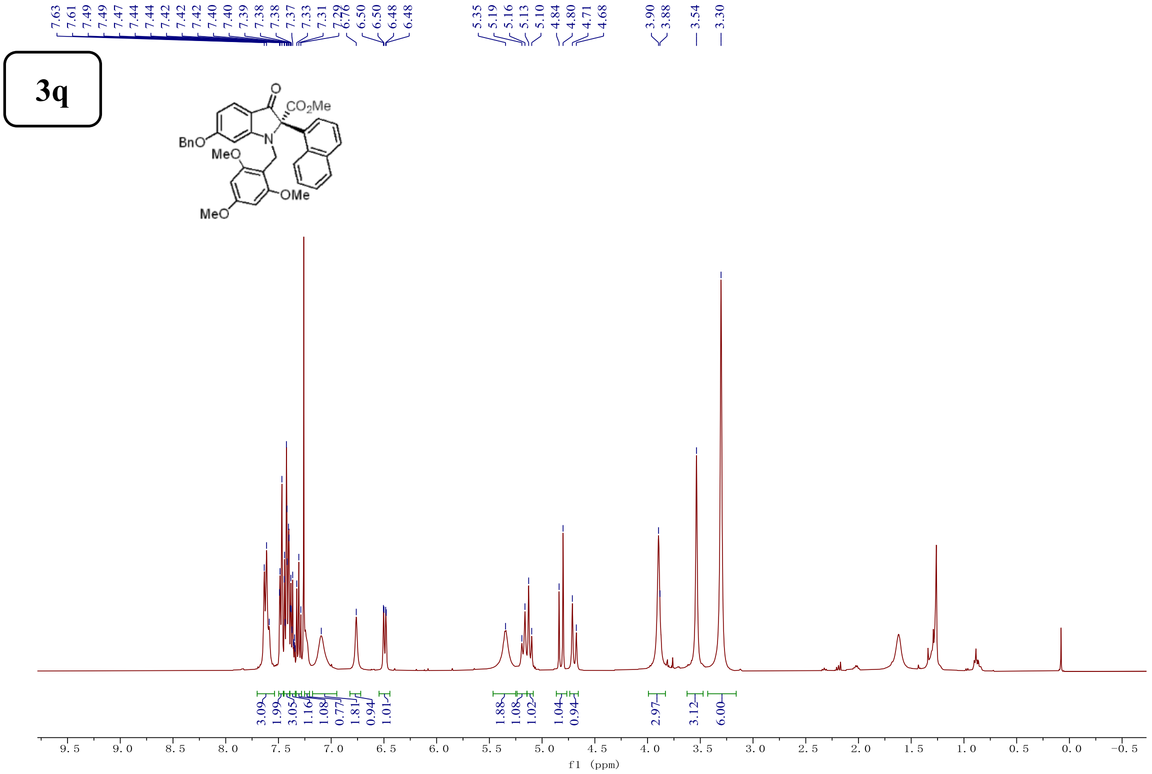
7.63
7.61
7.49
7.49
7.47
7.44
7.44
7.42
7.42
7.42
7.40
7.40
7.39
7.38
7.38
7.37
7.33
7.31
7.29
6.76
6.50
6.50
6.48
6.48
5.35
5.19
5.16
5.13
5.10
4.84
4.80
4.71
4.68
3.90
3.88
3.54
3.30
BnO
MeO
N
O
CO2Me
OMe
MeO
3.09
1.99
3.05
1.16
1.08
0.77
1.81
0.94
1.01
1.88
1.08
1.02
1.04
0.94
2.97
3.12
6.00
f1 (ppm)

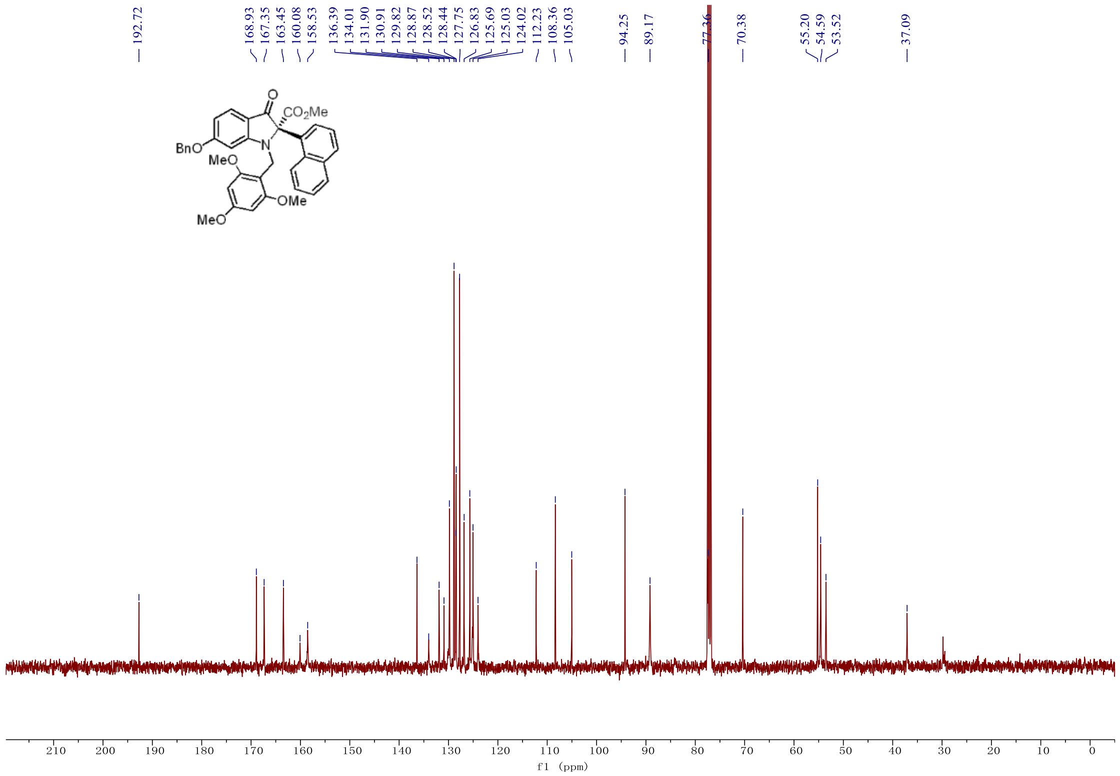
192.72
168.93
167.35
163.45
160.08
158.53
136.39
134.01
131.90
130.91
129.82
128.87
128.52
128.44
127.75
126.83
125.69
125.03
124.02
112.23
108.36
105.03
94.25
89.17
70.38
55.20
54.59
53.52
37.09
BnO
MeO
N
O
CO2Me
OMe
MeO
f1 (ppm)

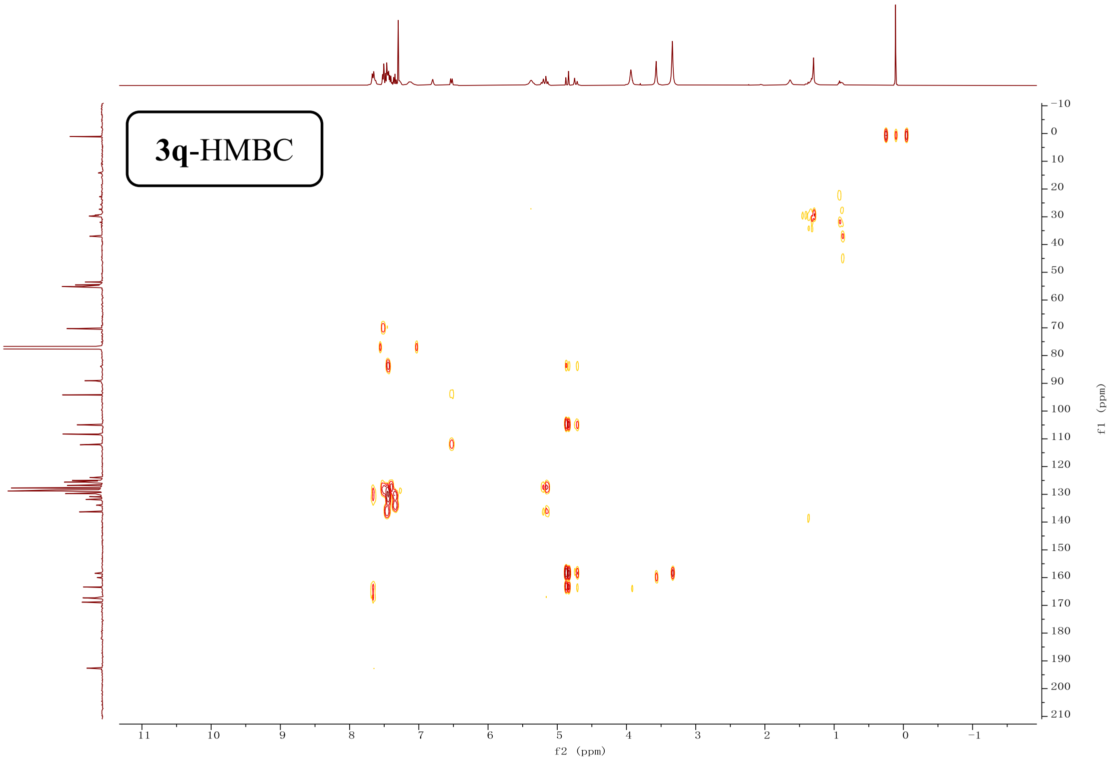
3q-HMBC
f2 (ppm)
f1 (ppm)

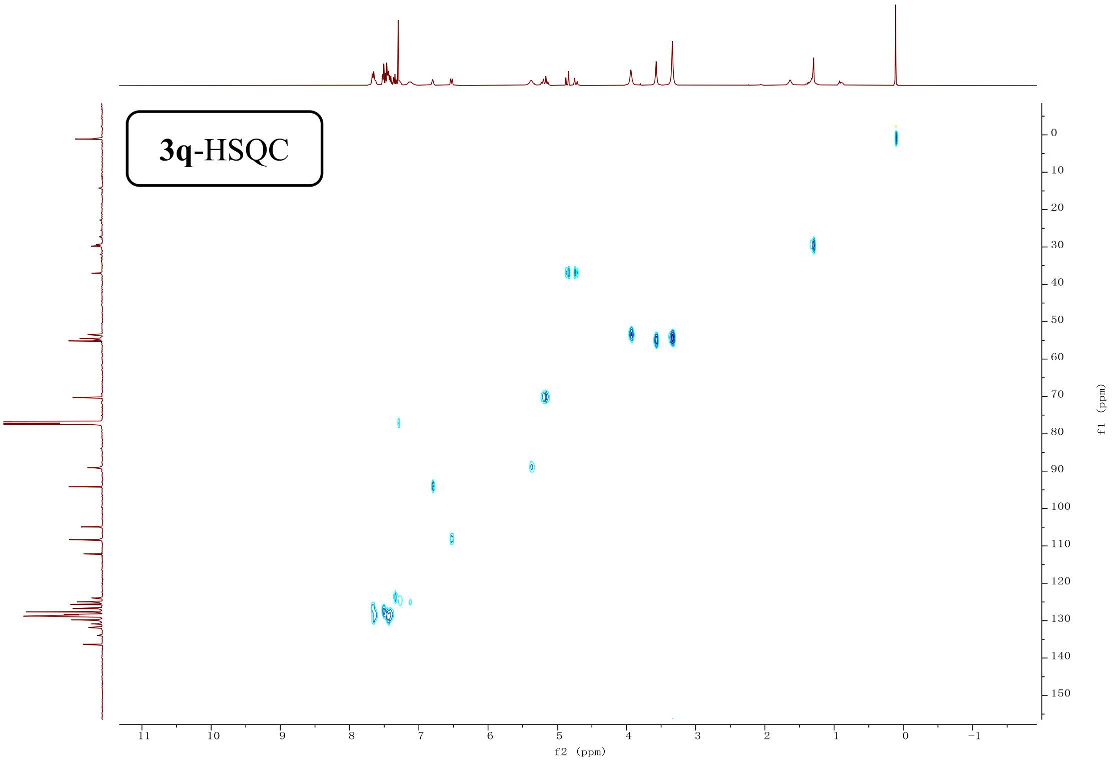
3q-HSQC
f2 (ppm)
f1 (ppm)

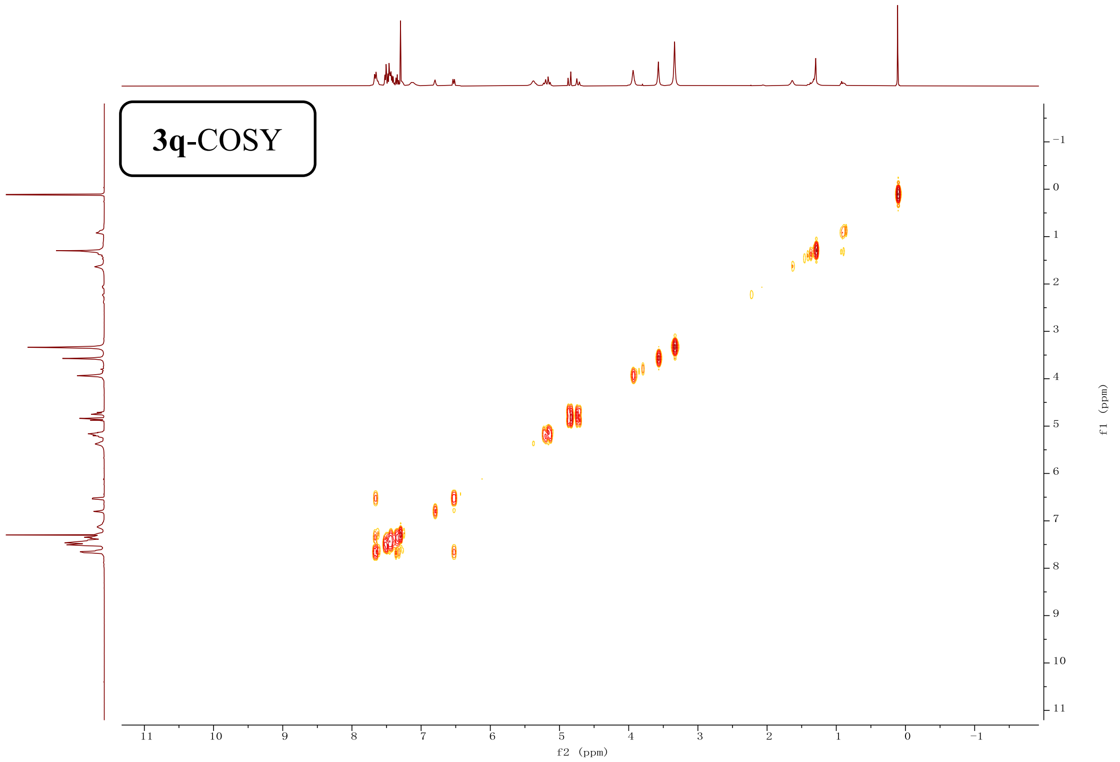
3q-COSY
f2 (ppm)
f1 (ppm)

3r

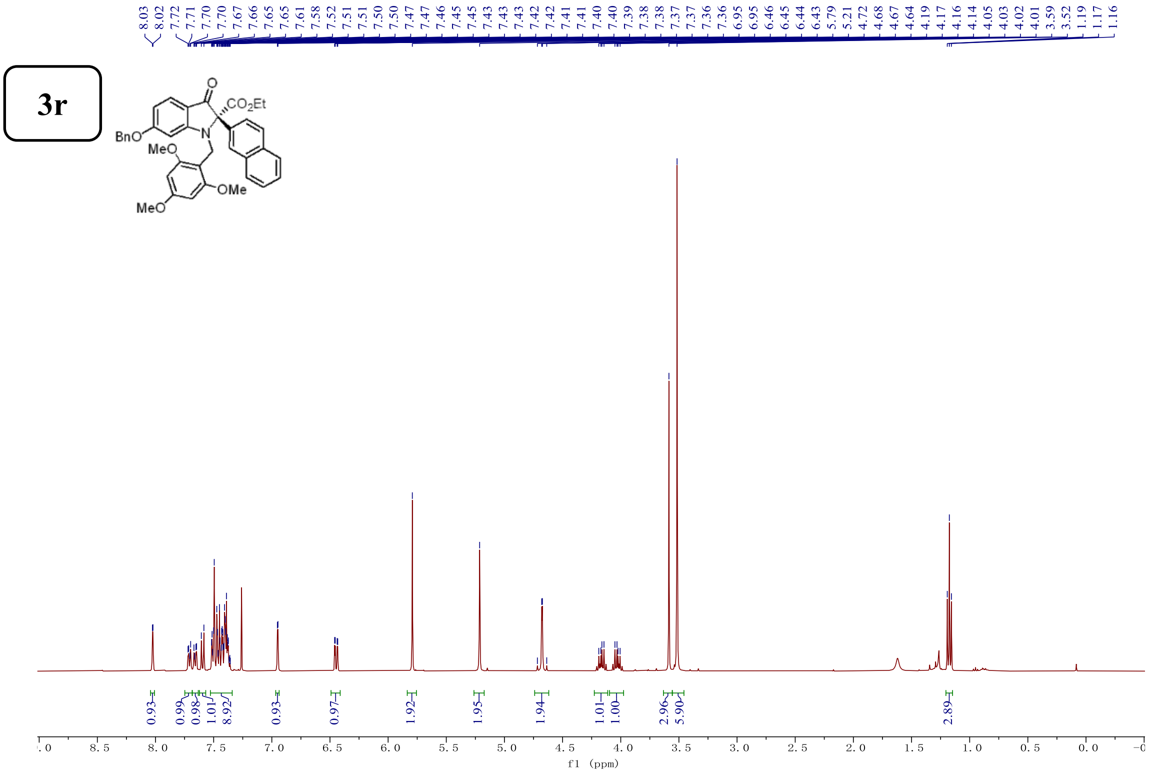
8.03 8.02 7.72 7.71 7.70 7.70 7.67 7.66 7.65 7.61 7.58 7.52 7.51 7.51 7.50 7.47 7.46 7.45 7.45 7.43 7.43 7.43 7.42 7.42 7.41 7.41 7.40 7.40 7.39 7.38 7.38 7.37 7.37 7.36 6.95 6.95 6.46 6.45 6.44 6.43 5.79 5.21 4.72 4.68 4.67 4.64 4.19 4.17 4.16 4.14 4.05 4.03 4.02 4.01 3.59 3.52 1.19 1.17 1.16
CO2Et
BnO
N
MeO
OMe
MeO
0.93 0.99 0.98 1.01 8.92 0.93 0.97 1.92 1.95 1.94 1.01 1.00 2.96 5.90 2.89
8.5 8.0 7.5 7.0 6.5 6.0 5.5 5.0 4.5 4.0 3.5 3.0 2.5 2.0 1.5 1.0 0.5 0.0
f1 (ppm)

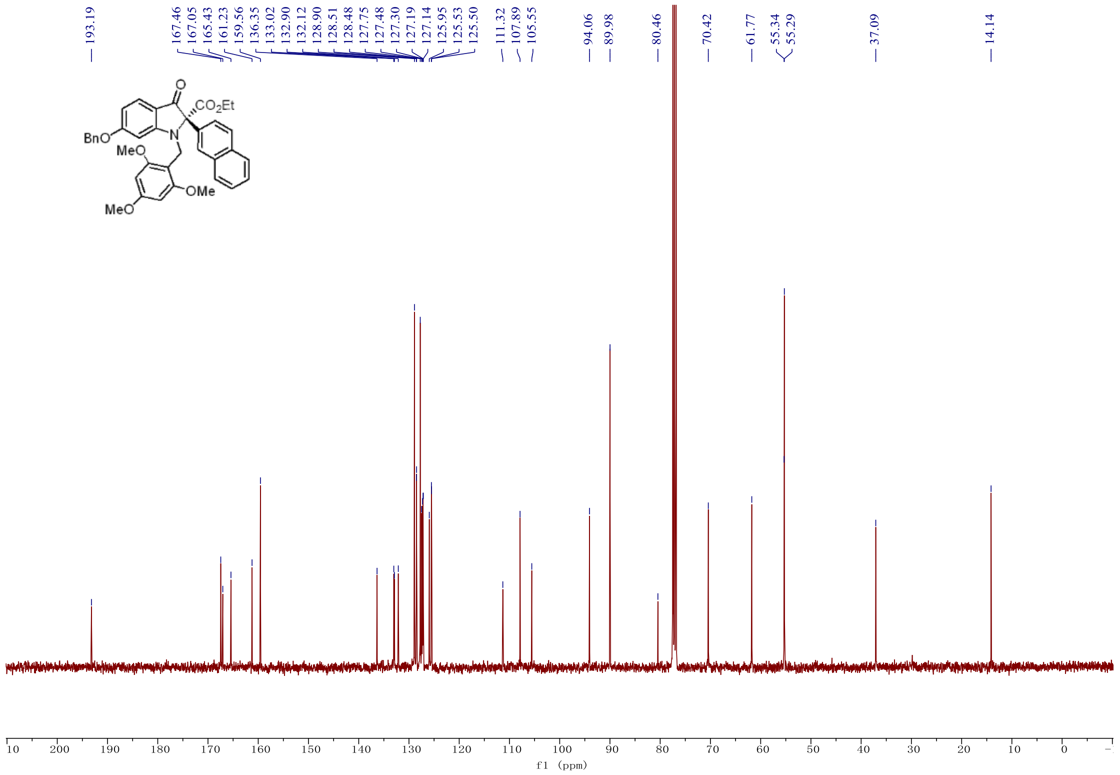
193.19
167.46 167.05 165.43 161.23 159.56 136.35 133.02 132.90 132.12 128.90 128.51 128.48 127.75 127.48 127.30 127.19 127.14 125.95 125.53 125.50
111.32 107.89 105.55
94.06 89.98 80.46 70.42 61.77 55.34 55.29 37.09 14.14
CO2Et
BnO
N
MeO
OMe
MeO
200 190 180 170 160 150 140 130 120 110 100 90 80 70 60 50 40 30 20 10 0
f1 (ppm)

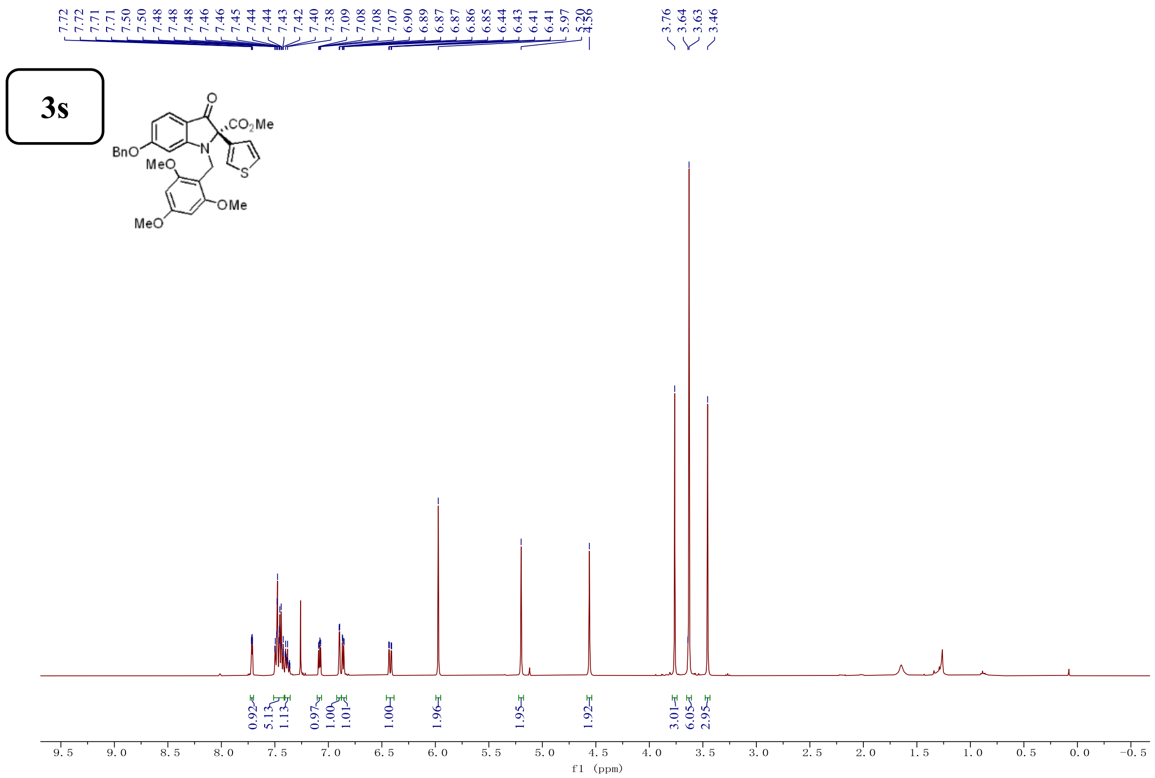
3s
BnO
CO2Me
N
S
MeO
OMe
MeO
7.72
7.72
7.71
7.71
7.50
7.50
7.48
7.48
7.46
7.46
7.45
7.44
7.44
7.43
7.42
7.40
7.38
7.09
7.08
7.08
7.07
6.90
6.89
6.87
6.87
6.86
6.85
6.44
6.43
6.41
6.41
5.97
5.20
4.56
3.76
3.64
3.63
3.46
0.92
5.13
1.13
0.97
1.00
1.01
1.00
1.96
1.95
1.92
3.01
6.05
2.95
f1 (ppm)

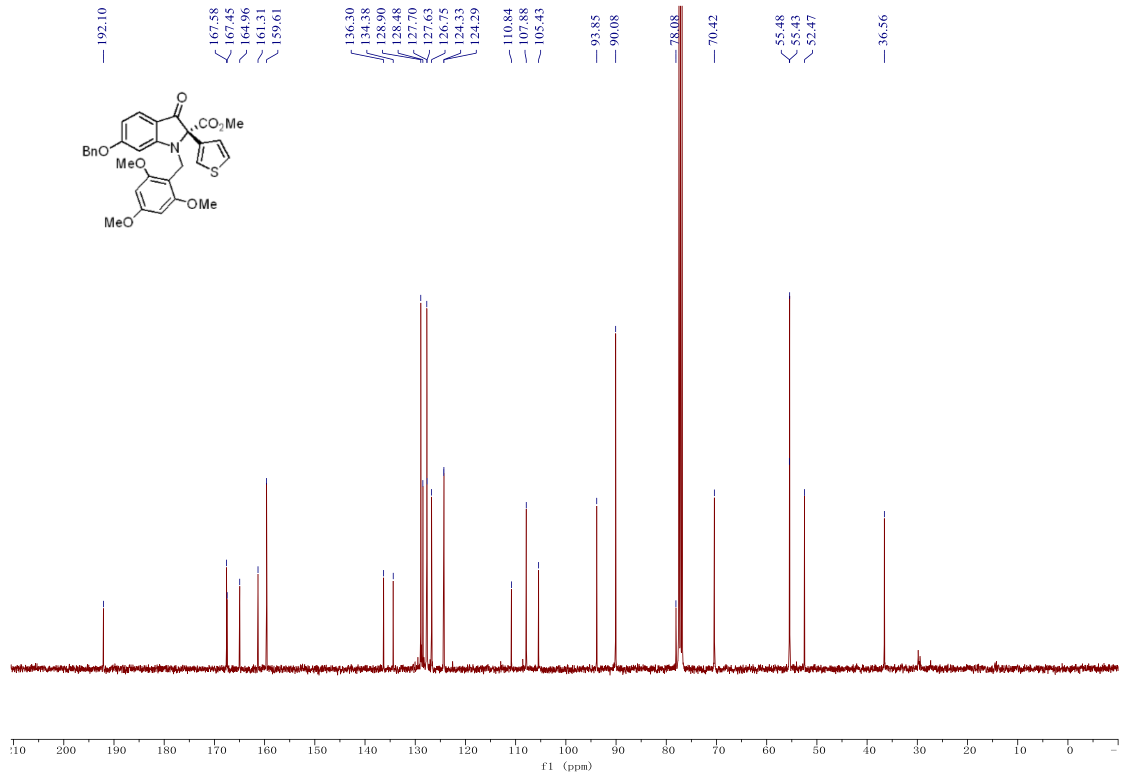
BnO
CO2Me
N
S
MeO
OMe
MeO
192.10
167.58
167.45
164.96
161.31
159.61
136.30
134.38
128.90
128.48
127.70
127.63
126.75
124.33
124.29
110.84
107.88
105.43
93.85
90.08
78.08
70.42
55.48
55.43
52.47
36.56
f1 (ppm)

3t

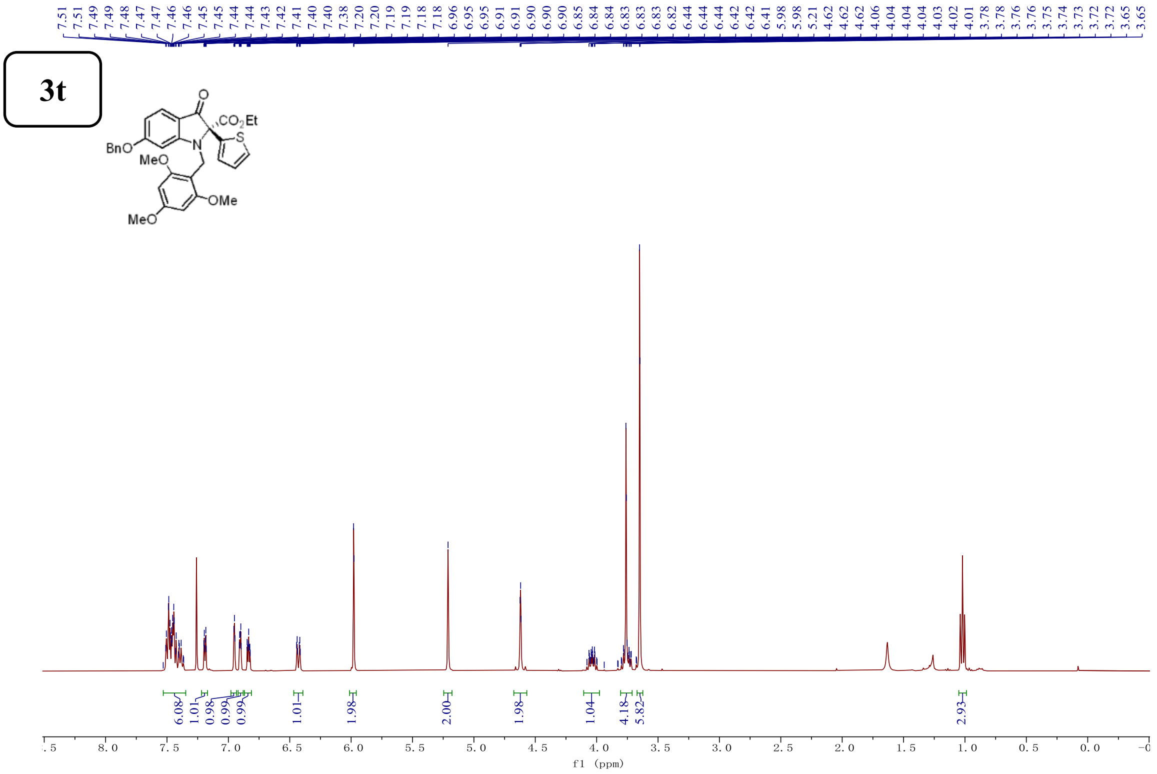

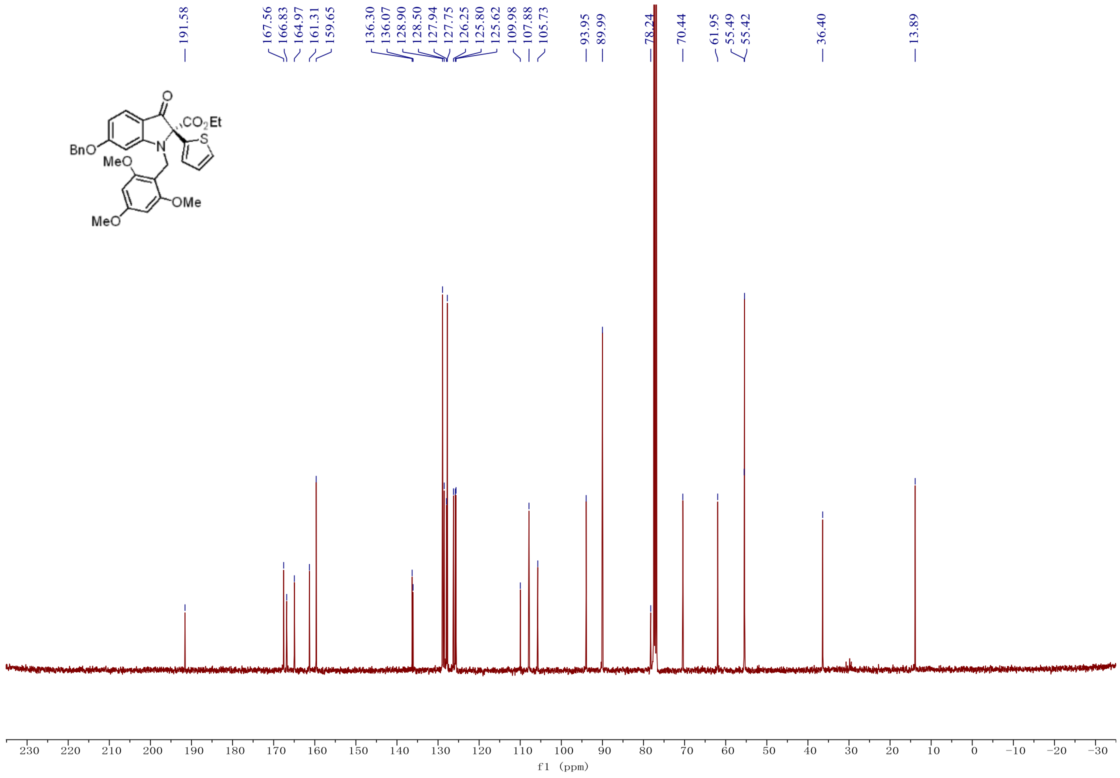

3u

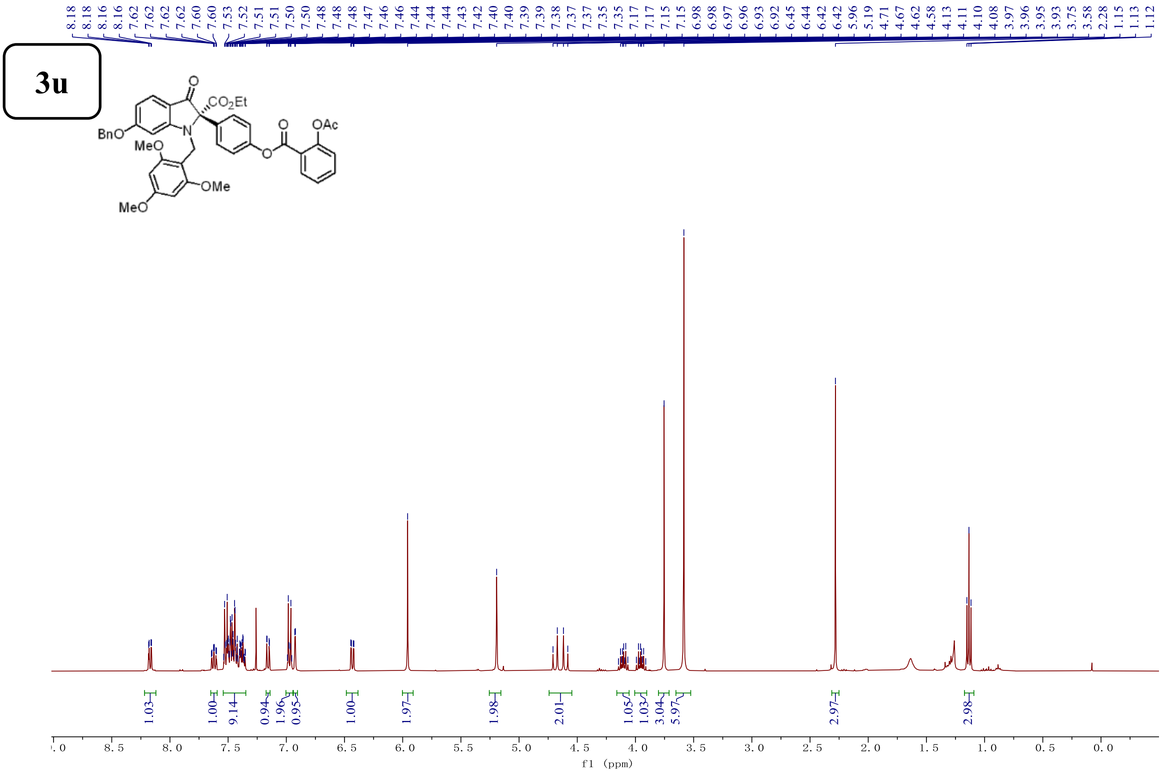
8.18 8.18 8.16 8.16 7.62 7.62 7.62 7.60 7.60 7.53 7.52 7.51 7.51 7.50 7.50 7.48 7.48 7.47 7.46 7.46 7.44 7.44 7.44 7.43 7.42 7.40 7.40 7.39 7.39 7.38 7.37 7.35 7.35 7.17 7.17 7.15 7.15 6.98 6.98 6.97 6.96 6.93 6.92 6.45 6.44 6.42 6.42 5.96 5.19 4.71 4.67 4.62 4.58 4.13 4.11 4.10 4.08 3.97 3.96 3.95 3.93 3.75 3.58 2.28 1.15 1.13 1.12
CO2Et
BnO
MeO
OAc
OMe
MeO
1.03 1.00 9.14 0.94 1.96 0.95 1.00 1.97 1.98 2.01 1.05 1.03 3.04 5.97 2.97 2.98
f1 (ppm)

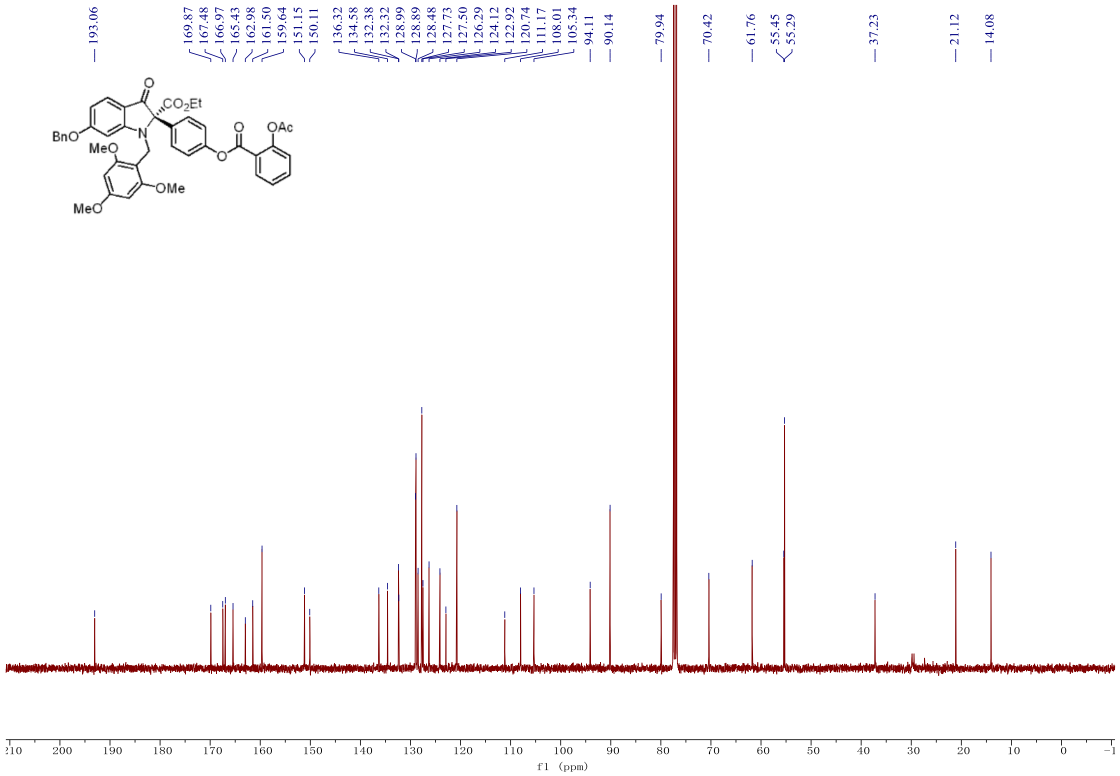
193.06 169.87 167.48 166.97 165.43 162.98 161.50 159.64 151.15 150.11 136.32 134.58 132.38 132.32 128.99 128.89 128.48 127.73 127.50 126.29 124.12 122.92 120.74 111.17 108.01 105.34 94.11 90.14 79.94 70.42 61.76 55.45 55.29 37.23 21.12 14.08
CO2Et
BnO
MeO
OAc
OMe
MeO
f1 (ppm)

3v

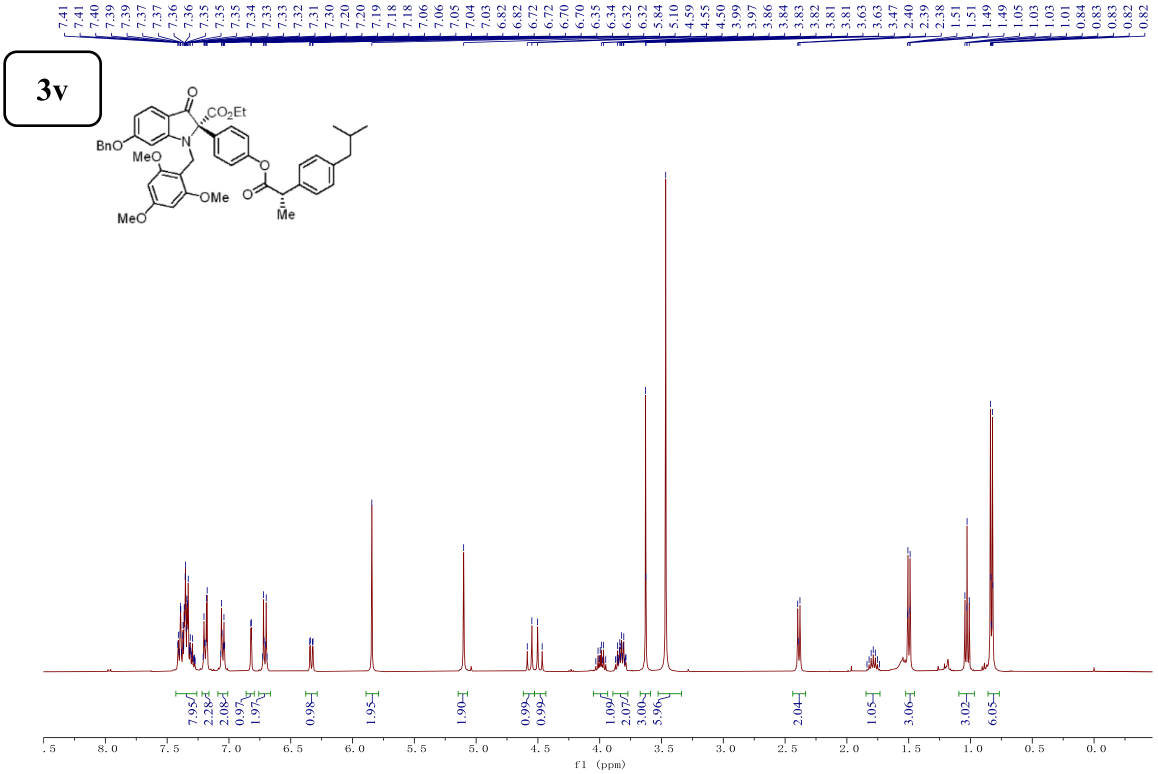
7.41 7.41 7.40 7.39 7.39 7.37 7.36 7.36 7.35 7.35 7.35 7.34 7.33 7.33 7.32 7.31 7.30 7.20 7.20 7.19 7.18 7.18 7.06 7.05 7.04 7.03 6.82 6.82 6.72 6.72 6.70 6.70 6.35 6.34 6.32 6.32 5.84 5.10 4.59 4.55 4.50 3.99 3.97 3.86 3.84 3.83 3.82 3.81 3.81 3.63 3.63 3.47 2.40 2.39 2.38 1.51 1.51 1.49 1.05 1.03 1.03 1.01 0.84 0.83 0.83 0.82 0.82
CO2Et
BnO
N
MeO
OMe
MeO
O
Me
7.95 2.28 2.08 0.97 1.97 0.98 1.95 1.90 0.99 0.99 1.09 2.07 3.00 5.96 2.04 1.05 3.06 3.02 6.05
f1 (ppm)

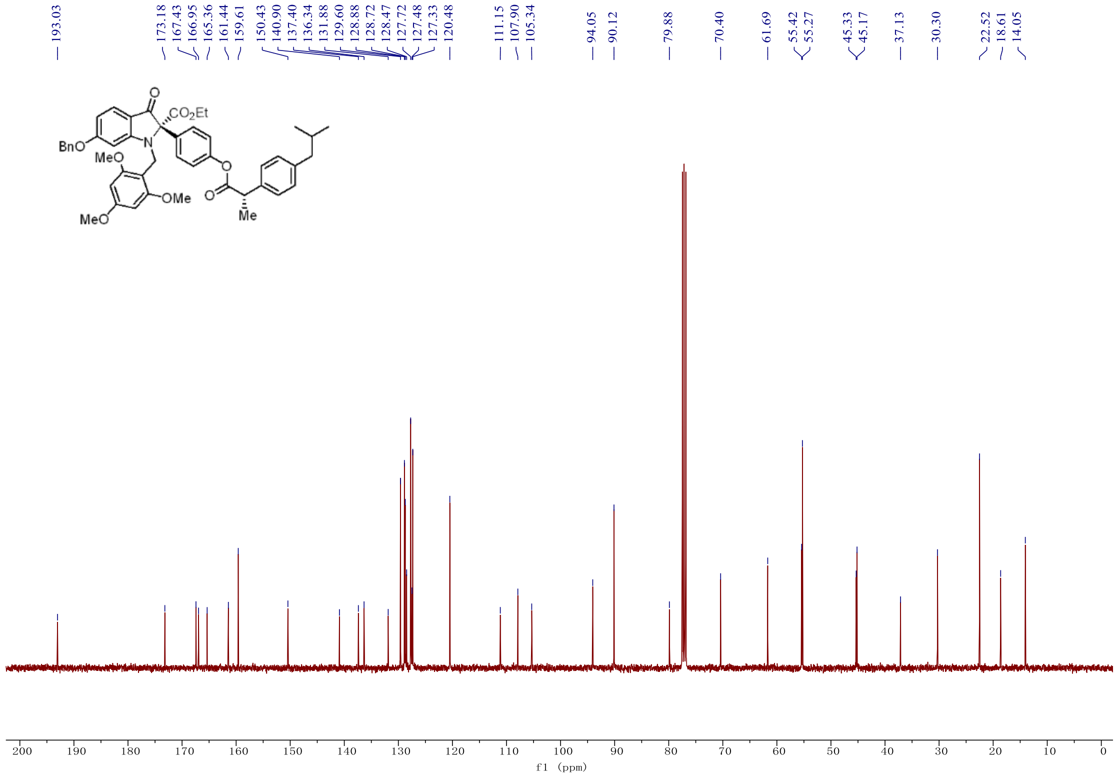
193.03 173.18 167.43 166.95 165.36 161.44 159.61 150.43 140.90 137.40 136.34 131.88 129.60 128.88 128.72 128.47 127.72 127.48 127.33 120.48 111.15 107.90 105.34 94.05 90.12 79.88 70.40 61.69 55.42 55.27 45.33 45.17 37.13 30.30 22.52 18.61 14.05
CO2Et
BnO
N
MeO
OMe
MeO
O
Me
f1 (ppm)

3w

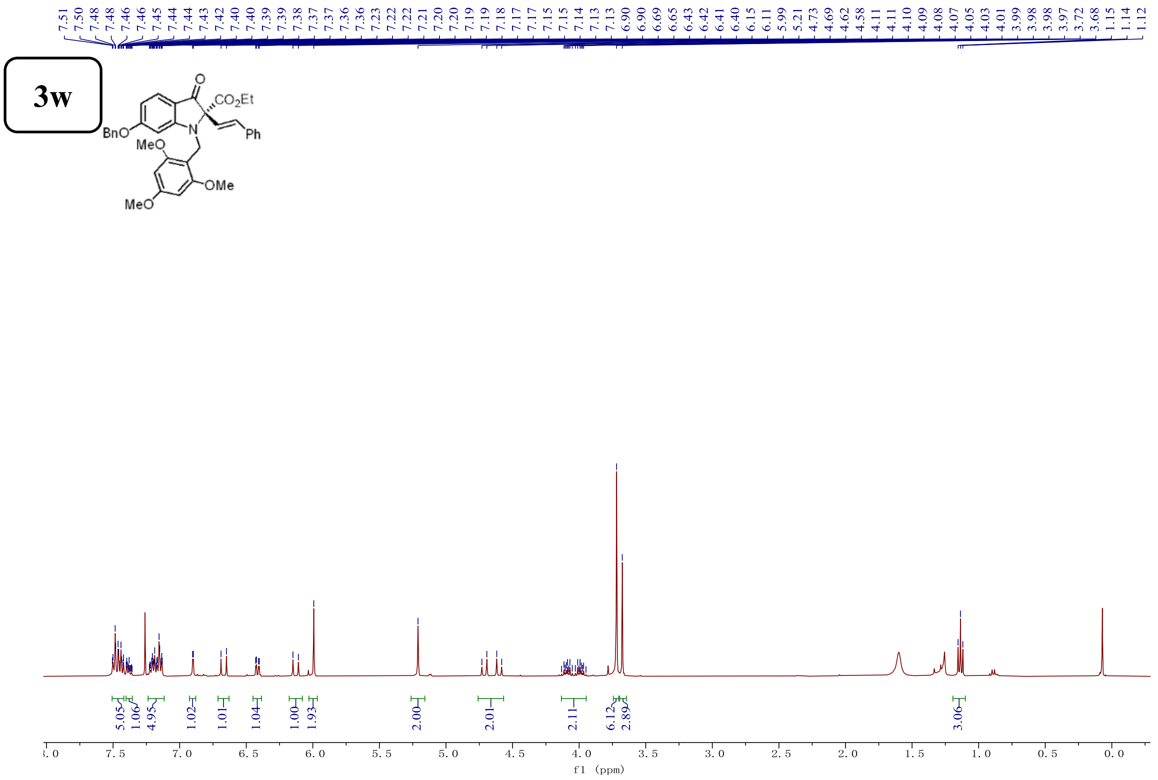
7.51 7.50 7.48 7.48 7.46 7.46 7.45 7.44 7.44 7.43 7.42 7.40 7.39 7.39 7.38 7.37 7.37 7.36 7.36 7.23 7.22 7.22 7.21 7.20 7.19 7.19 7.18 7.17 7.17 7.15 7.15 7.14 7.13 7.13 6.90 6.90 6.69 6.65 6.43 6.42 6.41 6.40 6.15 6.11 5.99 5.21 4.73 4.69 4.62 4.58 4.11 4.11 4.10 4.09 4.08 4.07 4.05 4.03 4.01 3.99 3.98 3.97 3.72 3.68 1.15 1.14 1.12
O
CO_2Et
BnO
N
Ph
MeO
OMe
MeO
5.05 1.06 4.95 1.02 1.01 1.04 1.00 1.93 2.00 2.01 2.11 6.12 2.89 3.06
f1 (ppm)

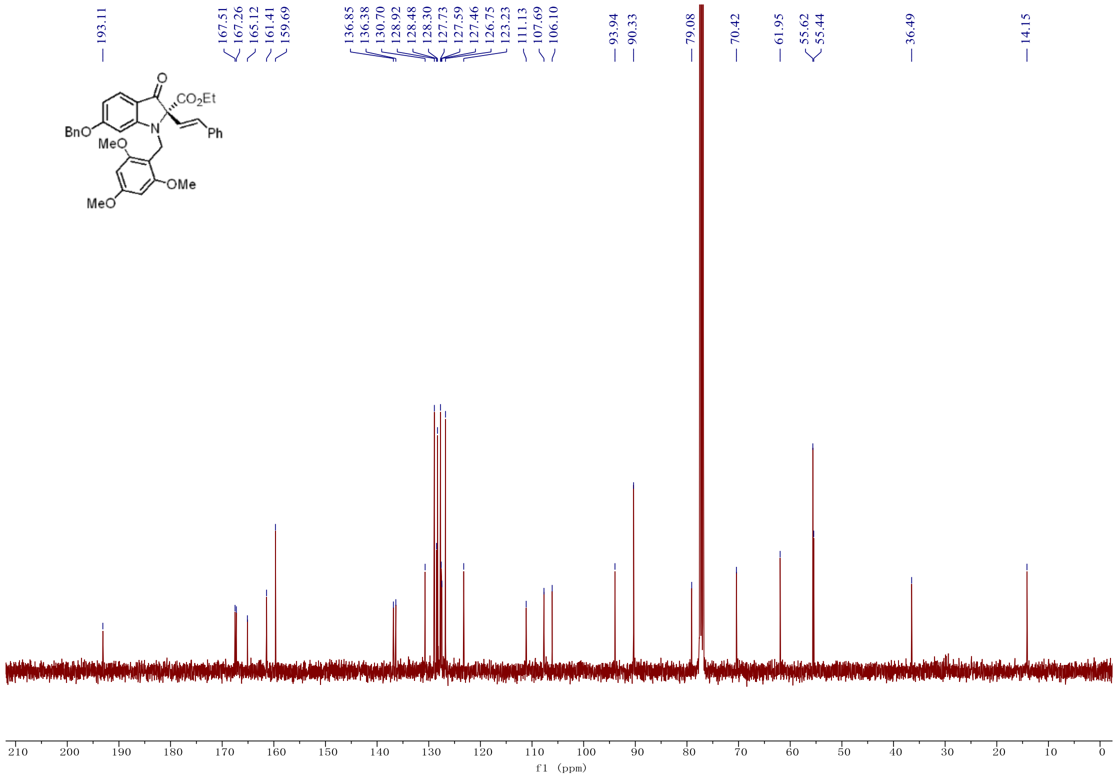
193.11 167.51 167.26 165.12 161.41 159.69 136.85 136.38 130.70 128.92 128.48 128.30 127.73 127.59 127.46 126.75 123.23 111.13 107.69 106.10 93.94 90.33 79.08 70.42 61.95 55.62 55.44 36.49 14.15
O
CO_2Et
BnO
N
Ph
MeO
OMe
MeO
f1 (ppm)

3x

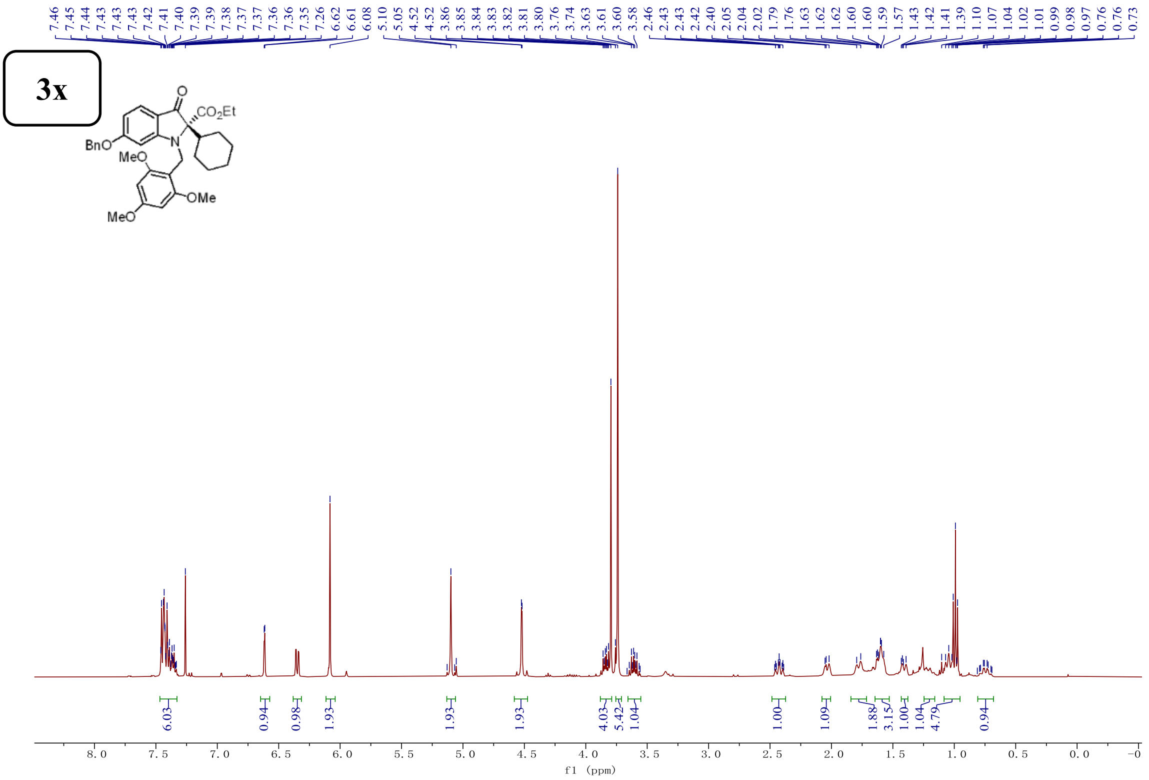
7.46
7.45
7.44
7.43
7.43
7.43
7.42
7.41
7.40
7.39
7.39
7.38
7.37
7.36
7.36
7.35
7.26
6.62
6.61
6.08
5.10
5.05
4.52
4.52
3.86
3.85
3.84
3.83
3.82
3.81
3.80
3.76
3.74
3.63
3.61
3.60
3.58
2.46
2.43
2.42
2.40
2.05
2.04
2.02
1.79
1.76
1.63
1.62
1.62
1.60
1.60
1.59
1.57
1.43
1.42
1.41
1.39
1.10
1.07
1.04
1.02
1.01
0.99
0.98
0.97
0.76
0.76
0.73
BnO
O
CO2Et
N
MeO
OMe
MeO
6.03
0.94
0.98
1.93
1.93
1.93
4.03
5.42
1.04
1.00
1.09
1.88
3.15
1.00
1.04
4.79
0.94
f1 (ppm)

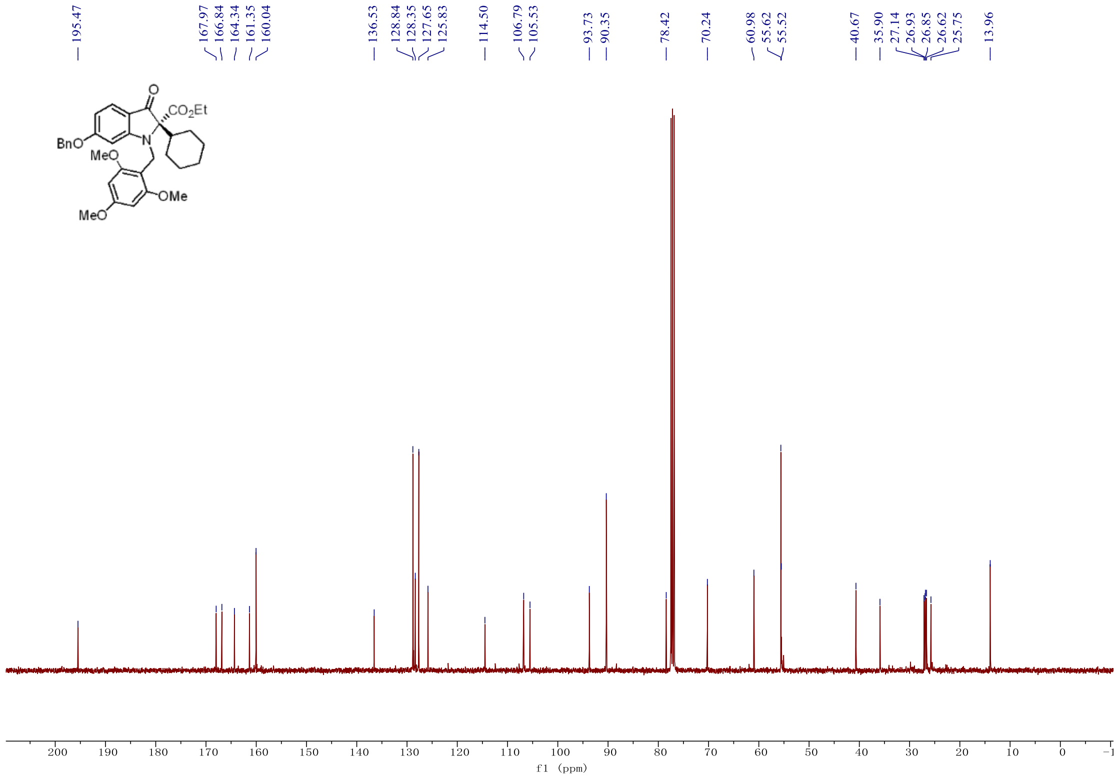
195.47
167.97
166.84
164.34
161.35
160.04
136.53
128.84
128.35
127.65
125.83
114.50
106.79
105.53
93.73
90.35
78.42
70.24
60.98
55.62
55.52
40.67
35.90
27.14
26.93
26.85
26.62
25.75
13.96
BnO
O
CO2Et
N
MeO
OMe
MeO
f1 (ppm)

3y

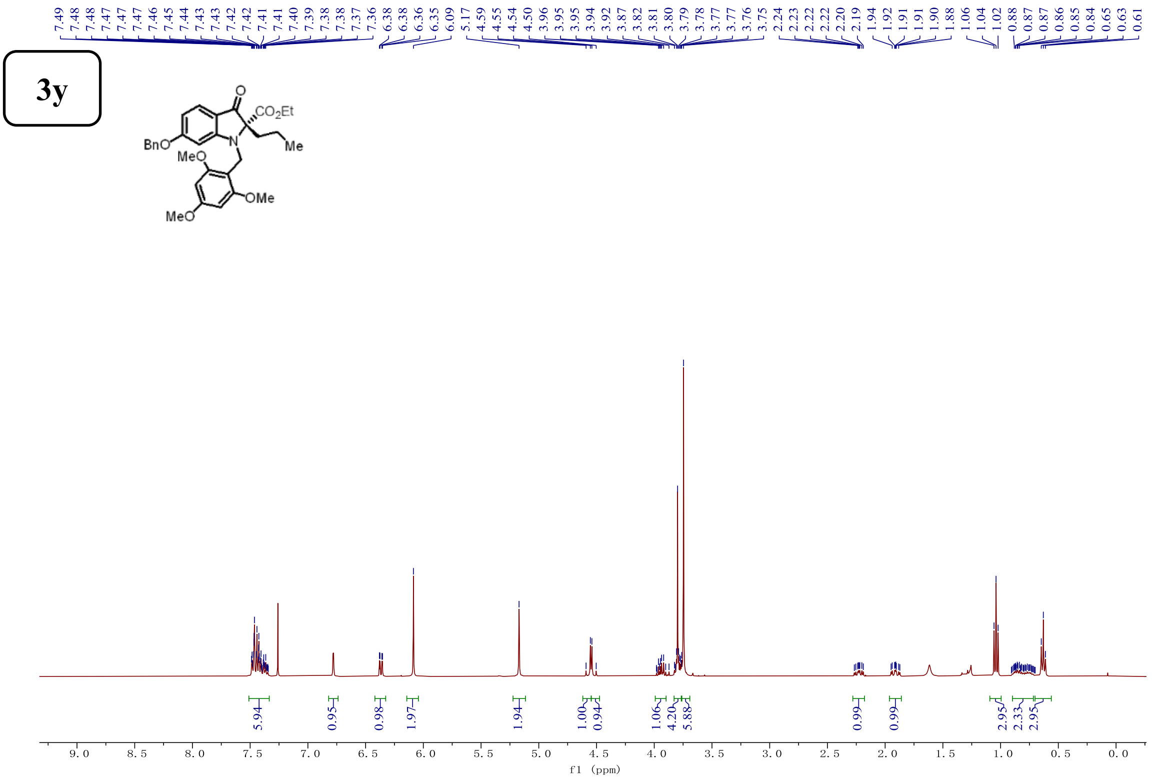
7.49 7.48 7.48 7.47 7.47 7.47 7.46 7.45 7.44 7.43 7.43 7.42 7.42 7.41 7.41 7.40 7.39 7.38 7.38 7.37 7.36 6.38 6.38 6.36 6.35 6.09 5.17 4.59 4.55 4.54 4.50 3.96 3.95 3.95 3.94 3.92 3.87 3.82 3.81 3.80 3.79 3.78 3.77 3.77 3.76 3.75 2.24 2.23 2.22 2.22 2.20 2.19 1.94 1.92 1.91 1.91 1.90 1.88 1.06 1.04 1.02 0.88 0.87 0.87 0.86 0.85 0.84 0.65 0.63 0.61
CO2Et
Me
BnO
MeO
OMe
MeO
O
N
5.94 0.95 0.98 1.97 1.94 1.00 0.94 1.06 4.20 5.88 0.99 0.99 2.95 2.33 2.95
f1 (ppm)

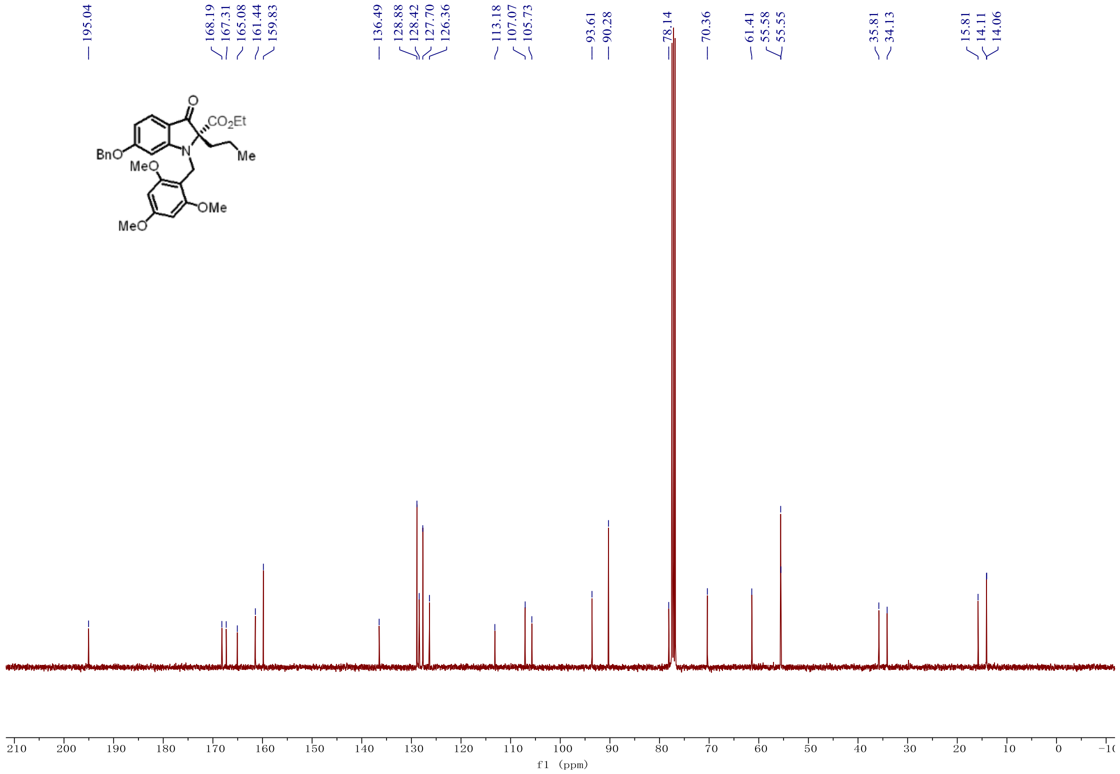
195.04 168.19 167.31 165.08 161.44 159.83 136.49 128.88 128.42 127.70 126.36 113.18 107.07 105.73 93.61 90.28 78.14 70.36 61.41 55.58 55.55 35.81 34.13 15.81 14.11 14.06
CO2Et
Me
BnO
MeO
OMe
MeO
O
N
f1 (ppm)

3z

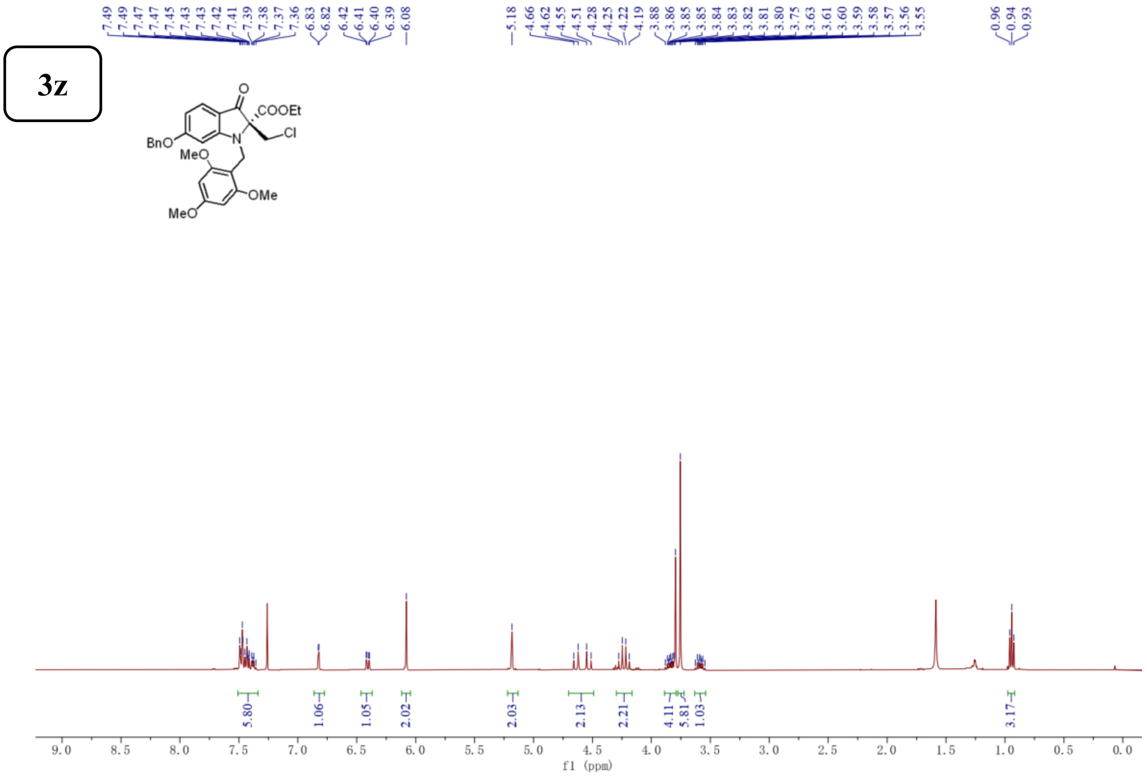

7.49 7.49 7.47 7.47 7.45 7.43 7.43 7.42 7.41 7.39 7.38 7.37 7.36 6.83 6.82 6.42 6.41 6.40 6.39 6.08
5.18 4.66 4.62 4.55 4.51 4.28 4.25 4.22 4.19 3.88 3.86 3.85 3.85 3.84 3.83 3.82 3.81 3.80 3.75 3.63 3.61 3.60 3.59 3.58 3.57 3.56 3.55
0.96 0.94 0.93
O
COOEt
Cl
BnO
N
MeO
OMe
MeO
5.80 1.06 1.05 2.02 2.03 2.13 2.21 4.11 5.81 1.03 3.17
9.0 8.5 8.0 7.5 7.0 6.5 6.0 5.5 5.0 4.5 4.0 3.5 3.0 2.5 2.0 1.5 1.0 0.5 0.0
f1 (ppm)

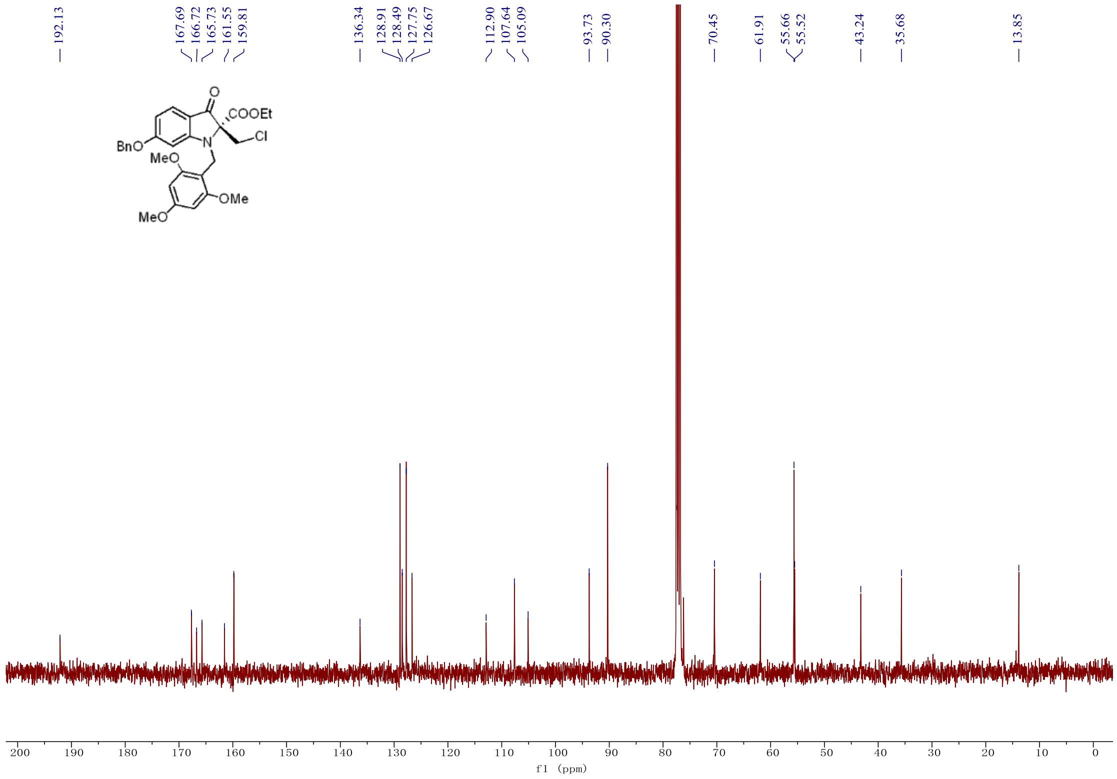

192.13
167.69 166.72 165.73 161.55 159.81
136.34 128.91 128.49 127.75 126.67
112.90 107.64 105.09
93.73 90.30
70.45
61.91 55.66 55.52
43.24 35.68
13.85
O
COOEt
Cl
BnO
N
MeO
OMe
MeO
200 190 180 170 160 150 140 130 120 110 100 90 80 70 60 50 40 30 20 10 0
f1 (ppm)

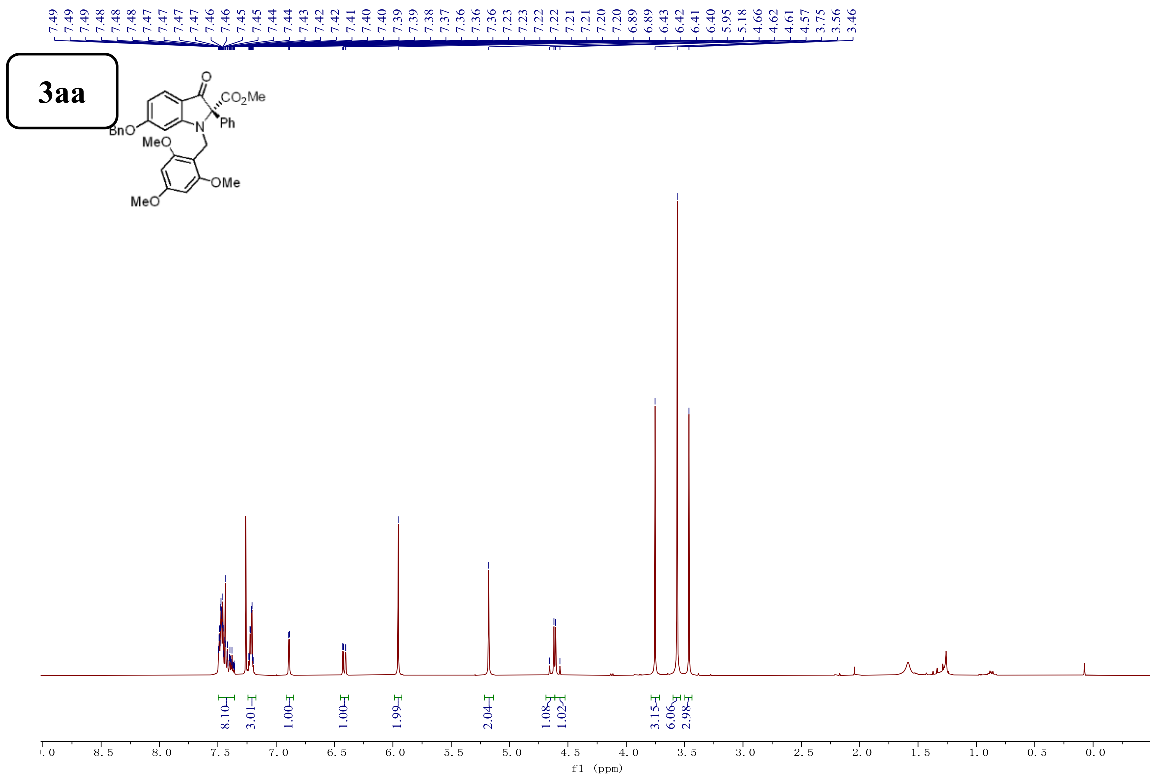

3aa
CO2Me
Ph
BnO
N
MeO
OMe
MeO
7.49 7.49 7.49 7.48 7.48 7.48 7.47 7.47 7.47 7.47 7.46 7.46 7.45 7.45 7.44 7.44 7.43 7.42 7.42 7.41 7.41 7.40 7.40 7.39 7.39 7.38 7.37 7.36 7.36 7.36 7.23 7.23 7.22 7.22 7.21 7.21 7.20 7.20 6.89 6.89 6.43 6.42 6.41 6.40 5.95 5.18 4.66 4.62 4.61 4.57 3.75 3.56 3.46
8.10 3.01 1.00 1.00 1.99 2.04 1.08 1.02 3.15 6.06 2.98
f1 (ppm)

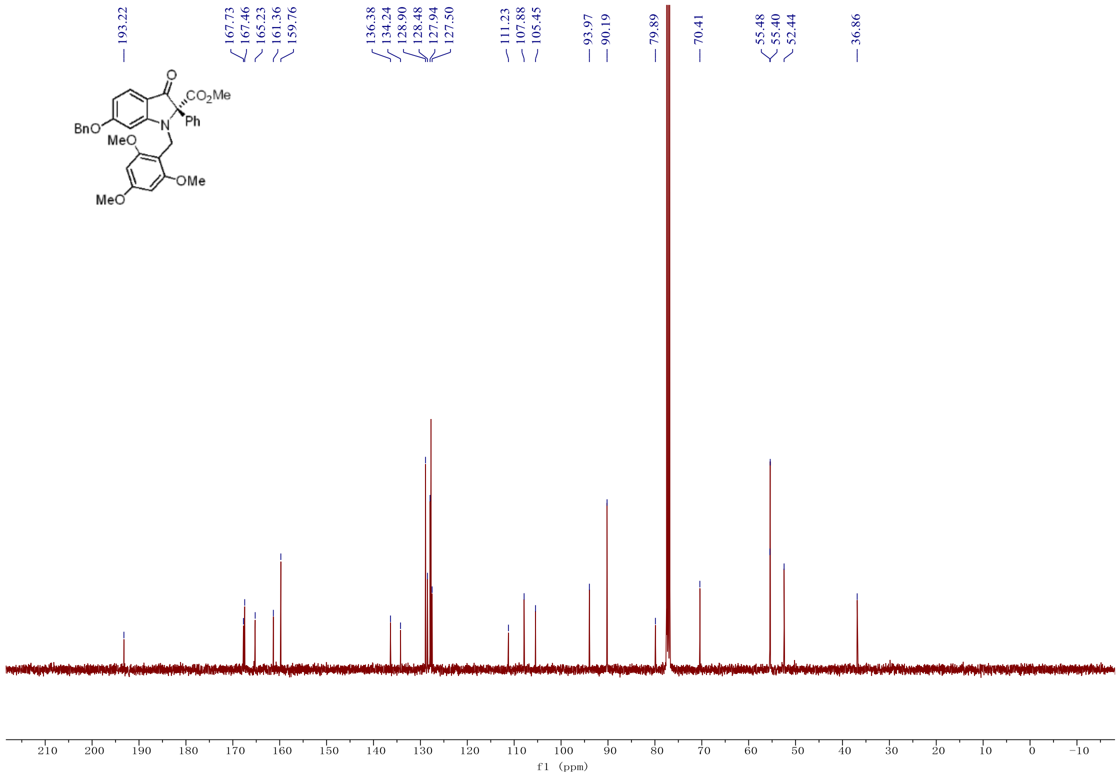

193.22
167.73 167.46 165.23 161.36 159.76
136.38 134.24 128.90 128.48 127.94 127.50
111.23 107.88 105.45
93.97 90.19
79.89
70.41
55.48 55.40 52.44
36.86
CO2Me
Ph
BnO
N
MeO
OMe
MeO
f1 (ppm)

3ab

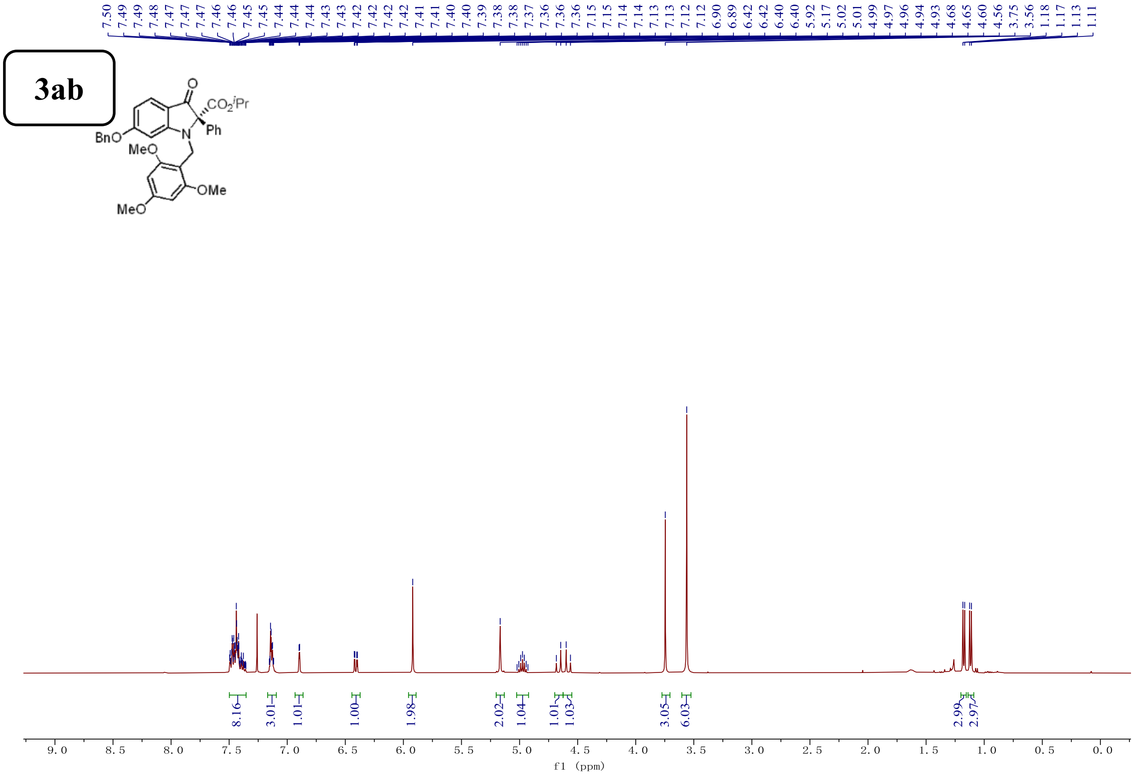
7.50 7.49 7.48 7.47 7.47 7.47 7.46 7.45 7.45 7.44 7.44 7.44 7.43 7.43 7.42 7.42 7.42 7.42 7.41 7.41 7.40 7.39 7.38 7.38 7.37 7.36 7.36 7.36 7.15 7.15 7.14 7.14 7.13 7.13 7.12 7.12 6.90 6.89 6.42 6.42 6.40 6.40 5.92 5.17 5.02 5.01 4.99 4.97 4.96 4.94 4.93 4.68 4.65 4.60 4.56 3.75 3.56 1.18 1.17 1.13 1.11
O
$CO_2{}^iPr$
Ph
BnO
N
MeO
OMe
MeO
8.16 3.01 1.01 1.00 1.98 2.02 1.04 1.01 1.03 3.05 6.03 2.99 2.97
9.0 8.5 8.0 7.5 7.0 6.5 6.0 5.5 5.0 4.5 4.0 3.5 3.0 2.5 2.0 1.5 1.0 0.5 0.0
f1 (ppm)

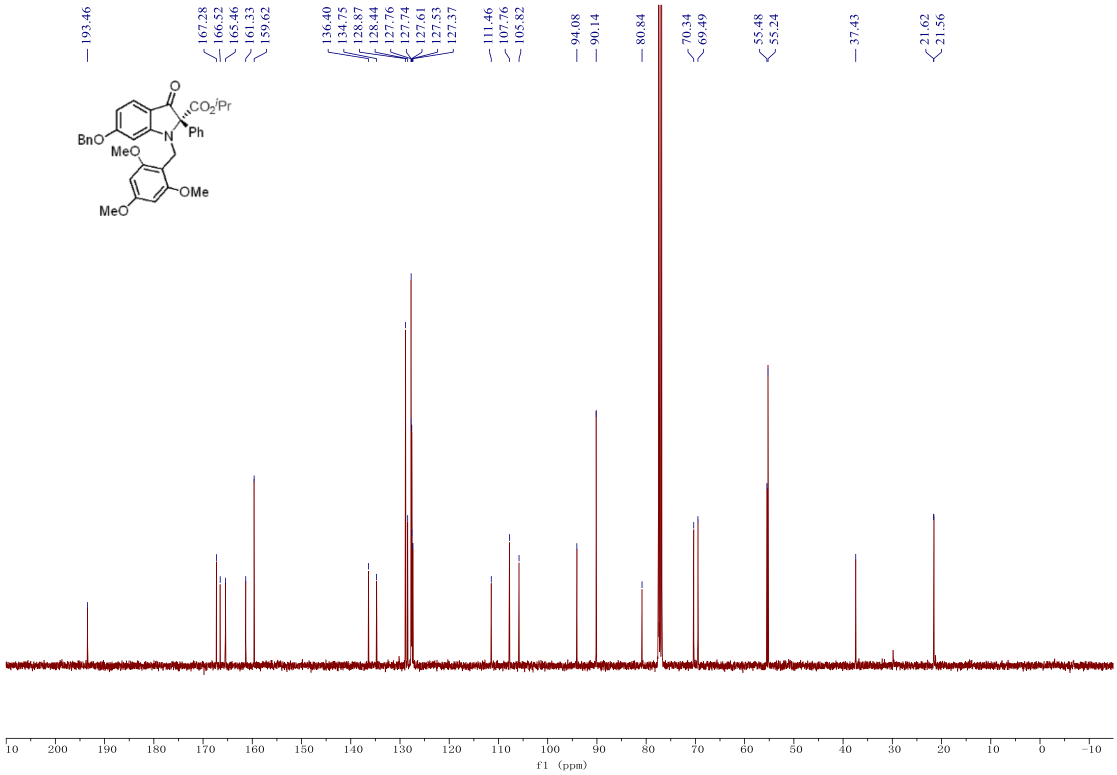
193.46
167.28 166.52 165.46 161.33 159.62
136.40 134.75 128.87 128.44 127.76 127.74 127.61 127.53 127.37
111.46 107.76 105.82
94.08 90.14
80.84
70.34 69.49
55.48 55.24
37.43
21.62 21.56
O
$CO_2{}^iPr$
Ph
BnO
N
MeO
OMe
MeO
10 200 190 180 170 160 150 140 130 120 110 100 90 80 70 60 50 40 30 20 10 0 -10
f1 (ppm)

3ac

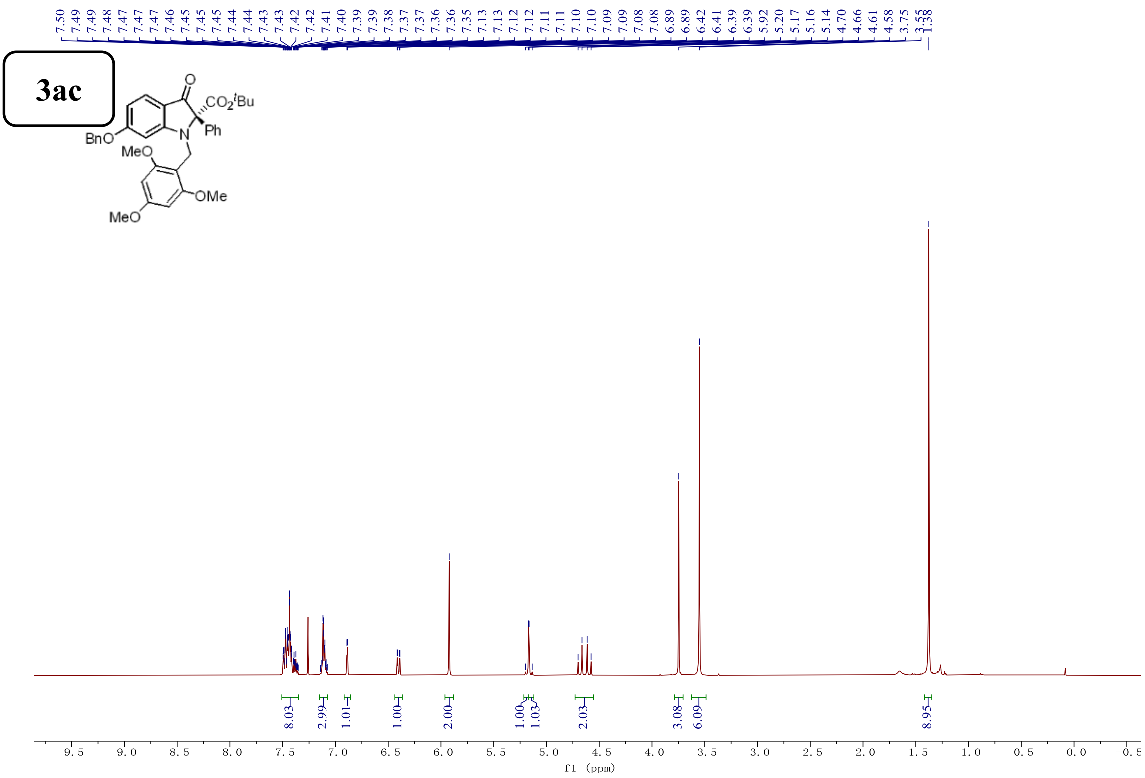
7.50 7.49 7.48 7.47 7.47 7.46 7.45 7.45 7.44 7.44 7.43 7.43 7.42 7.42 7.41 7.40 7.39 7.39 7.38 7.37 7.37 7.36 7.36 7.35 7.13 7.13 7.12 7.12 7.11 7.11 7.10 7.10 7.09 7.09 7.08 7.08 6.89 6.89 6.42 6.41 6.39 6.39 5.92 5.20 5.17 5.16 5.14 4.70 4.66 4.61 4.58 3.75 3.55 1.38
O
CO2tBu
Ph
N
BnO
MeO
OMe
MeO
8.03 2.99 1.01 1.00 2.00 1.00 1.03 2.03 3.08 6.09 8.95
9.5 9.0 8.5 8.0 7.5 7.0 6.5 6.0 5.5 5.0 4.5 4.0 3.5 3.0 2.5 2.0 1.5 1.0 0.5 0.0 -0.5
f1 (ppm)

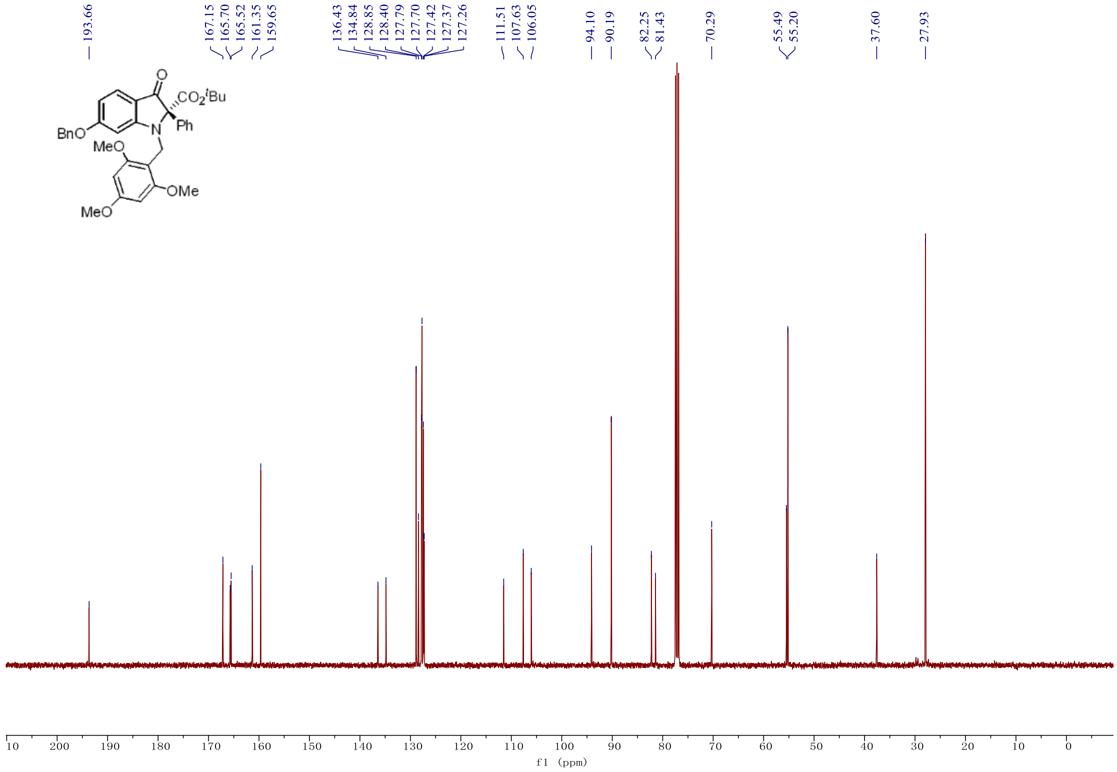
193.66
167.15 165.70 165.52 161.35 159.65
136.43 134.84 128.85 128.40 127.79 127.70 127.42 127.37 127.26
111.51 107.63 106.05
94.10 90.19
82.25 81.43
70.29
55.49 55.20
37.60
27.93
O
CO2tBu
Ph
N
BnO
MeO
OMe
MeO
10 200 190 180 170 160 150 140 130 120 110 100 90 80 70 60 50 40 30 20 10 0
f1 (ppm)

3ad

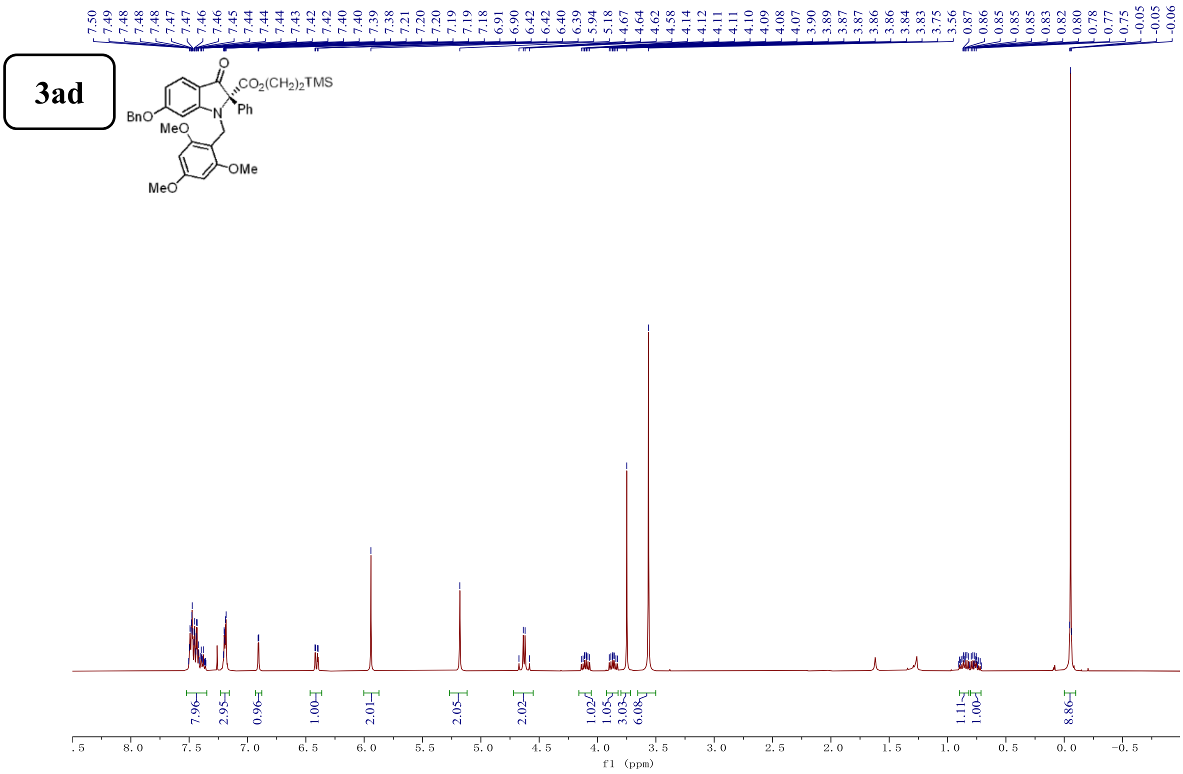
7.50 7.49 7.48 7.48 7.48 7.47 7.46 7.46 7.45 7.44 7.44 7.44 7.43 7.42 7.42 7.40 7.40 7.39 7.38 7.21 7.20 7.20 7.19 7.19 7.18 6.91 6.90 6.42 6.42 6.40 6.39 5.94 5.18 4.67 4.64 4.62 4.58 4.14 4.12 4.11 4.11 4.10 4.09 4.08 4.07 3.90 3.89 3.87 3.87 3.86 3.86 3.84 3.83 3.75 3.56 0.87 0.86 0.85 0.85 0.85 0.83 0.82 0.80 0.78 0.77 0.75 -0.05 -0.05 -0.06
CO2(CH2)2TMS
Ph
BnO
N
MeO
OMe
MeO
7.96
2.95
0.96
1.00
2.01
2.05
2.02
1.02
1.05
3.03
6.08
1.11
1.00
8.86
f1 (ppm)

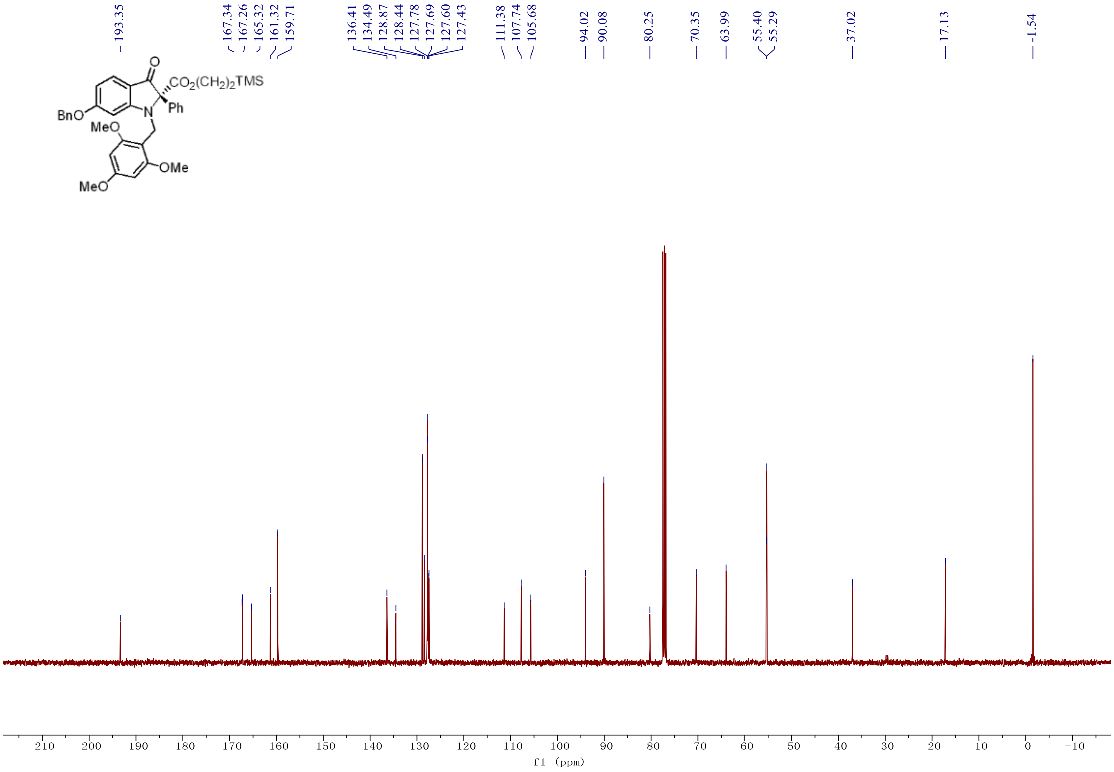
193.35
167.34 167.26 165.32 161.32 159.71
136.41 134.49 128.87 128.44 127.78 127.69 127.60 127.43
111.38 107.74 105.68
94.02 90.08
80.25
70.35
63.99
55.40 55.29
37.02
17.13
-1.54
CO2(CH2)2TMS
Ph
BnO
N
MeO
OMe
MeO
f1 (ppm)

3ae

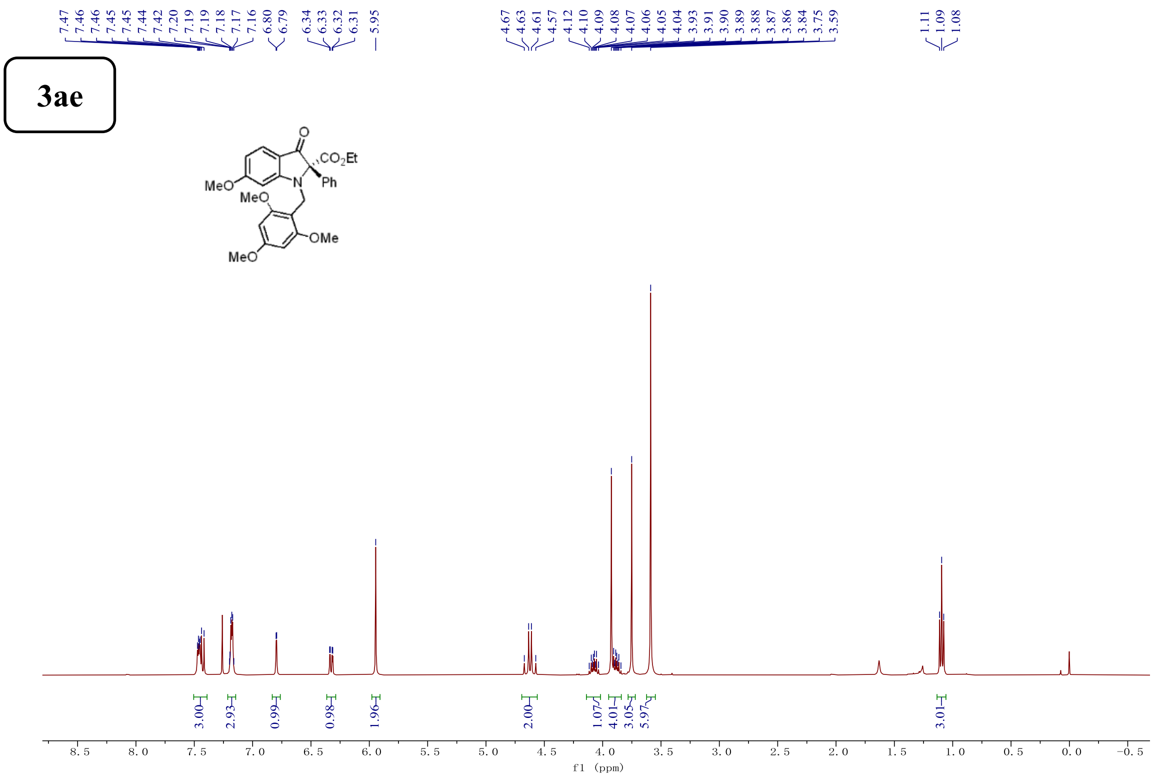

CO$_2$Et
Ph
N
O
MeO
MeO
OMe
MeO
f1 (ppm)

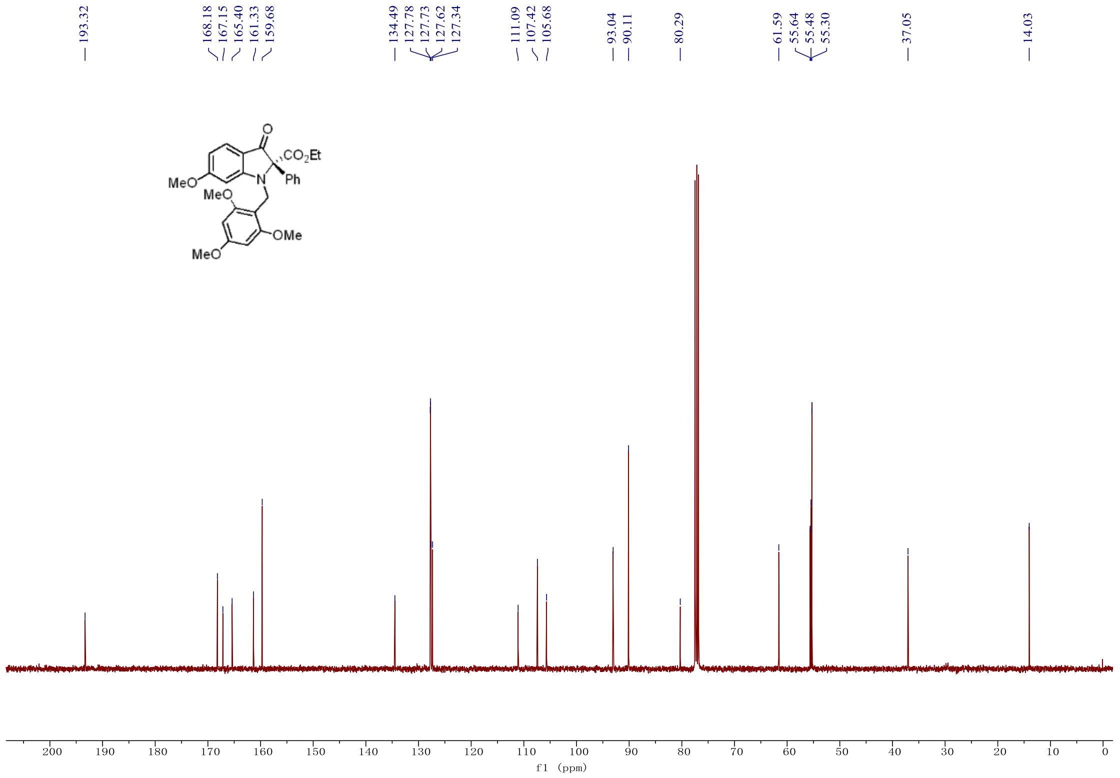

CO$_2$Et
Ph
N
O
MeO
MeO
OMe
MeO
f1 (ppm)

3af

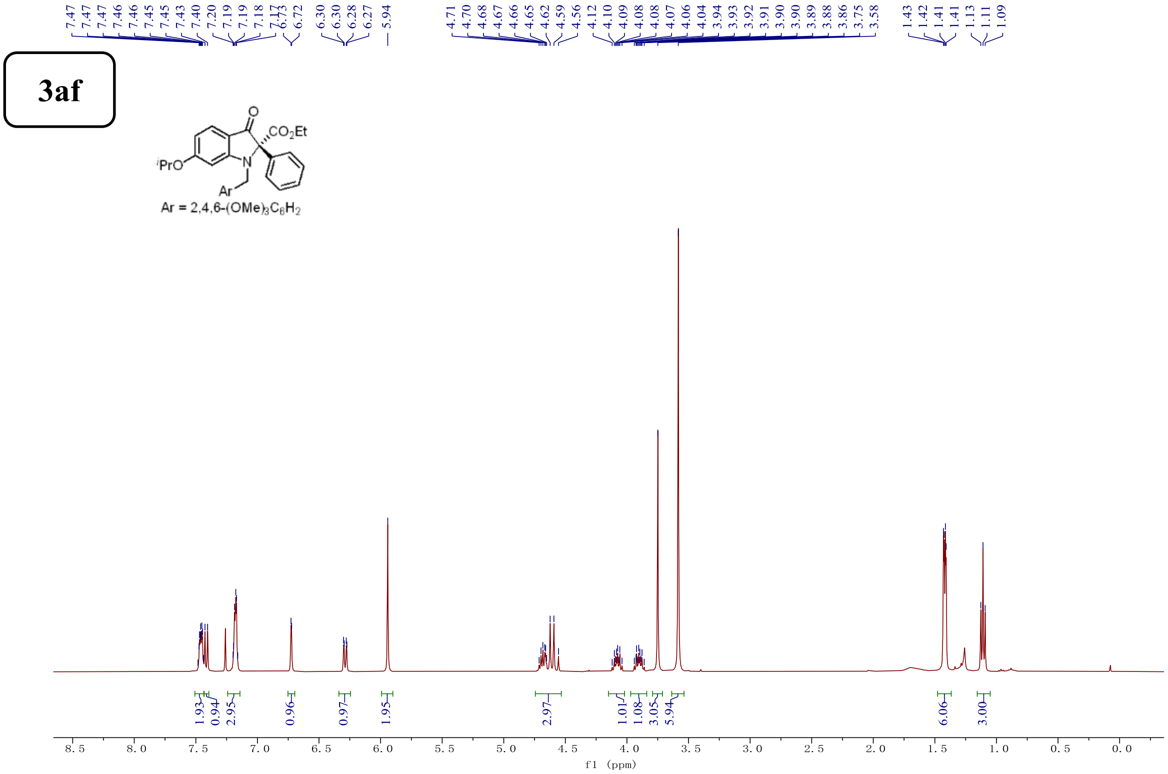
7.47 7.47 7.47 7.46 7.46 7.45 7.45 7.43 7.40 7.20 7.19 7.19 7.18 7.17 6.73 6.72 6.30 6.30 6.28 6.27 5.94
4.71 4.70 4.68 4.67 4.66 4.65 4.62 4.59 4.56 4.12 4.10 4.09 4.08 4.08 4.07 4.06 4.04 3.94 3.93 3.92 3.91 3.90 3.90 3.89 3.88 3.86 3.75 3.58
1.43 1.42 1.41 1.41 1.13 1.11 1.09
CO2Et
iPrO
Ar = 2,4,6-(OMe)3C6H2
1.93 0.94 2.95 0.96 0.97 1.95 2.97 1.01 1.08 3.05 5.94 6.06 3.00
f1 (ppm)

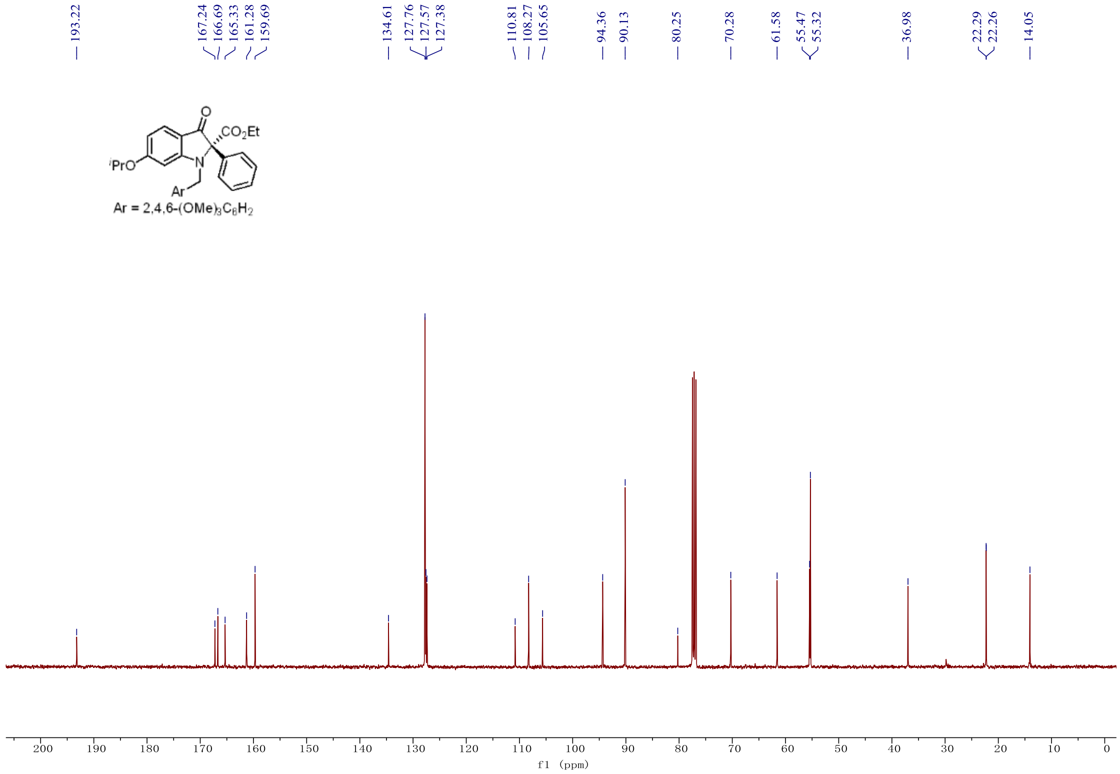
193.22
167.24 166.69 165.33 161.28 159.69
134.61 127.76 127.57 127.38
110.81 108.27 105.65
94.36 90.13 80.25 70.28 61.58 55.47 55.32 36.98 22.29 22.26 14.05
CO2Et
iPrO
Ar = 2,4,6-(OMe)3C6H2
f1 (ppm)

3ag

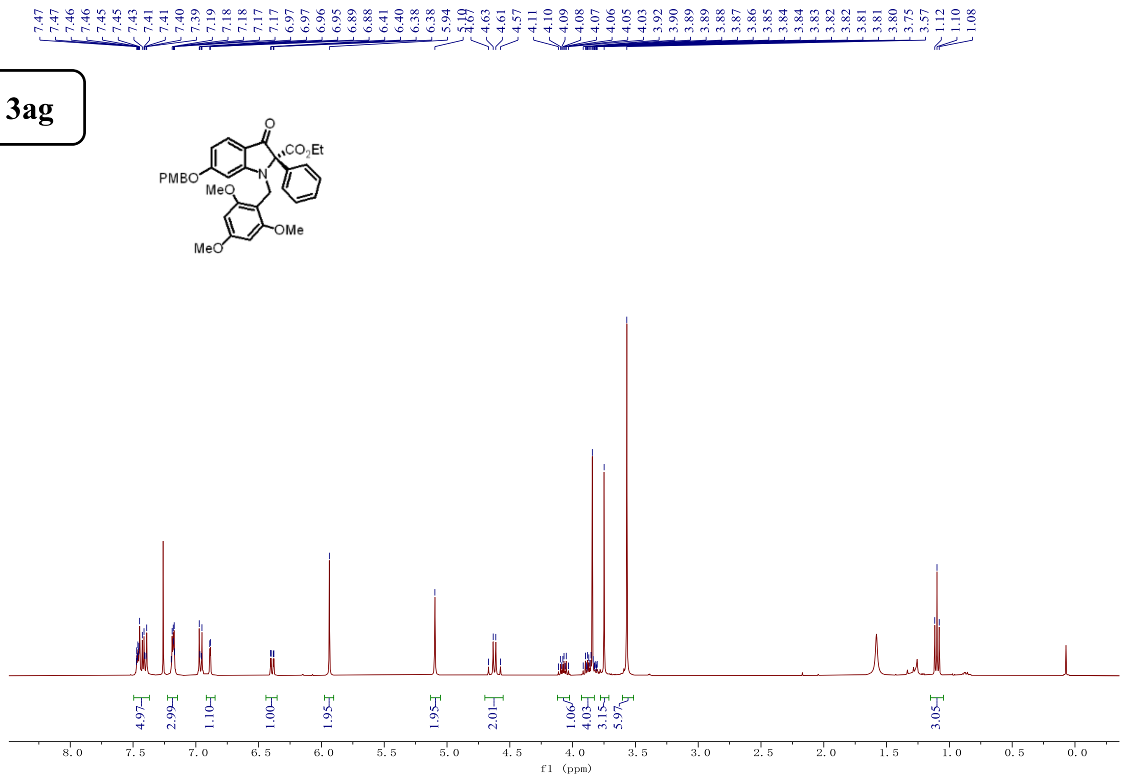

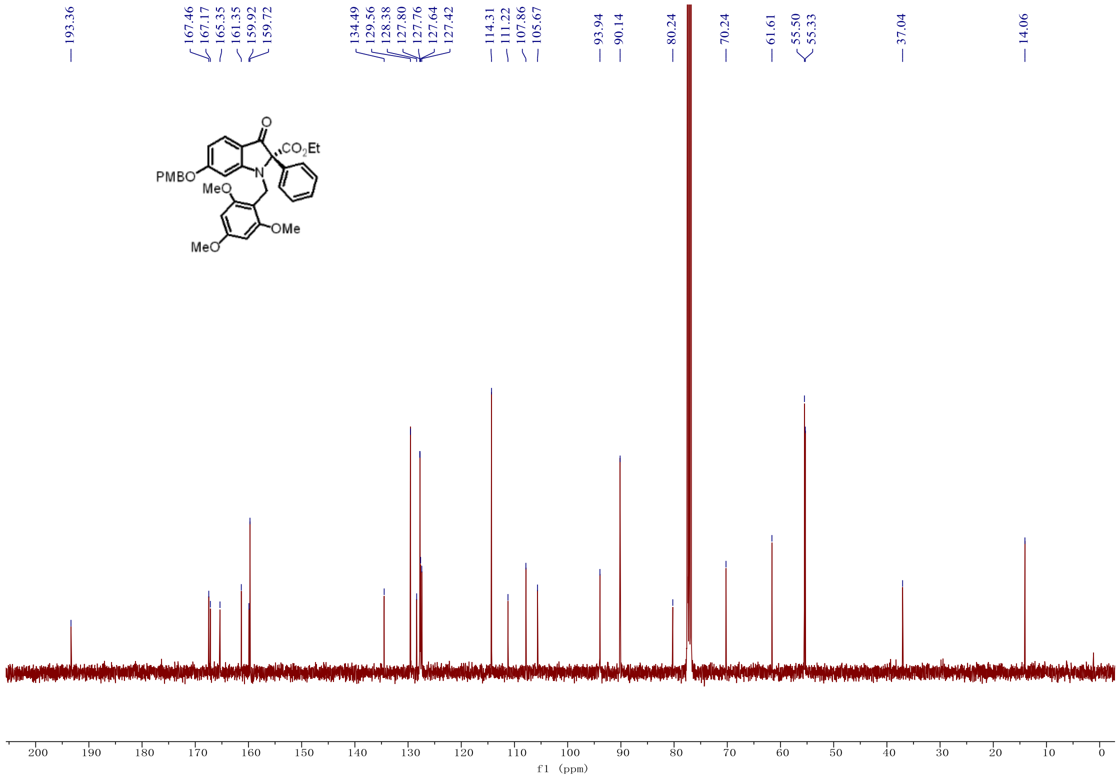

3ah

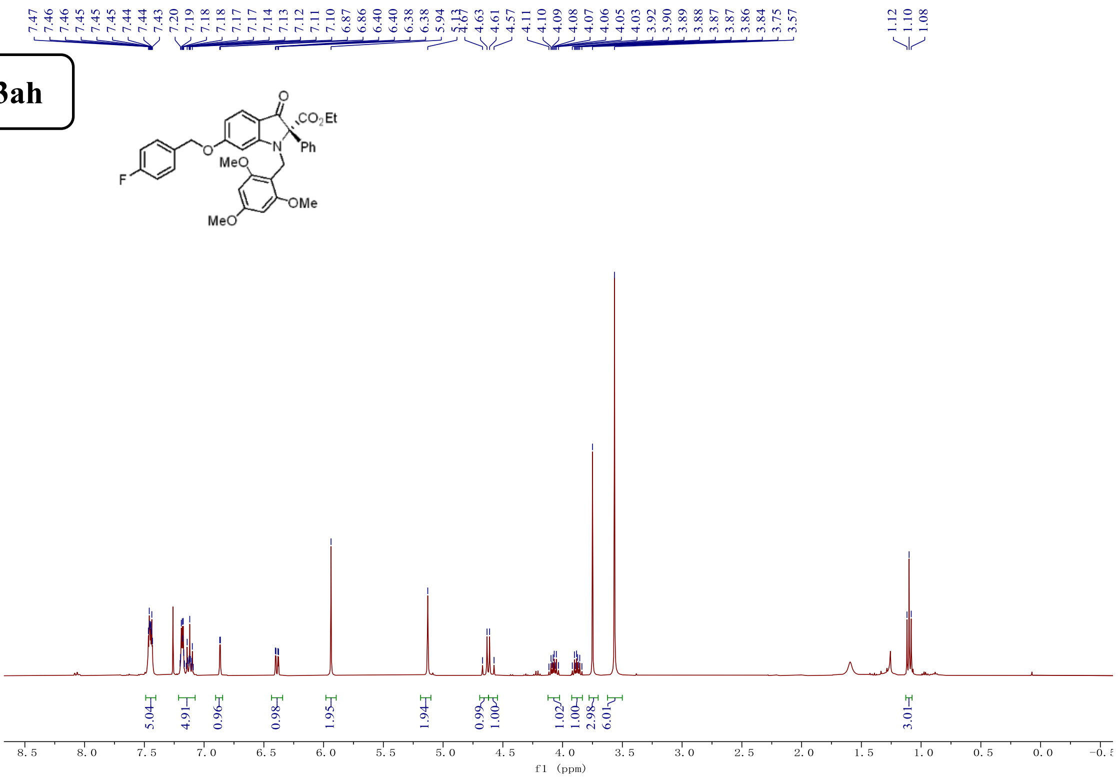

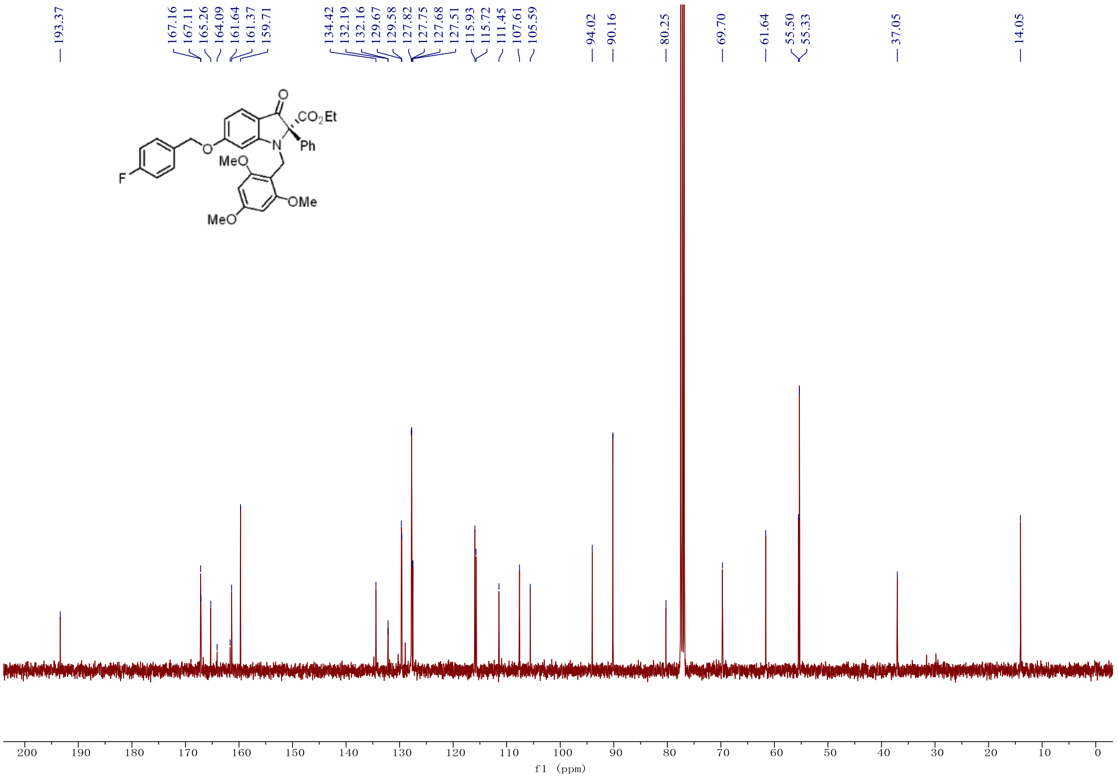

3ai

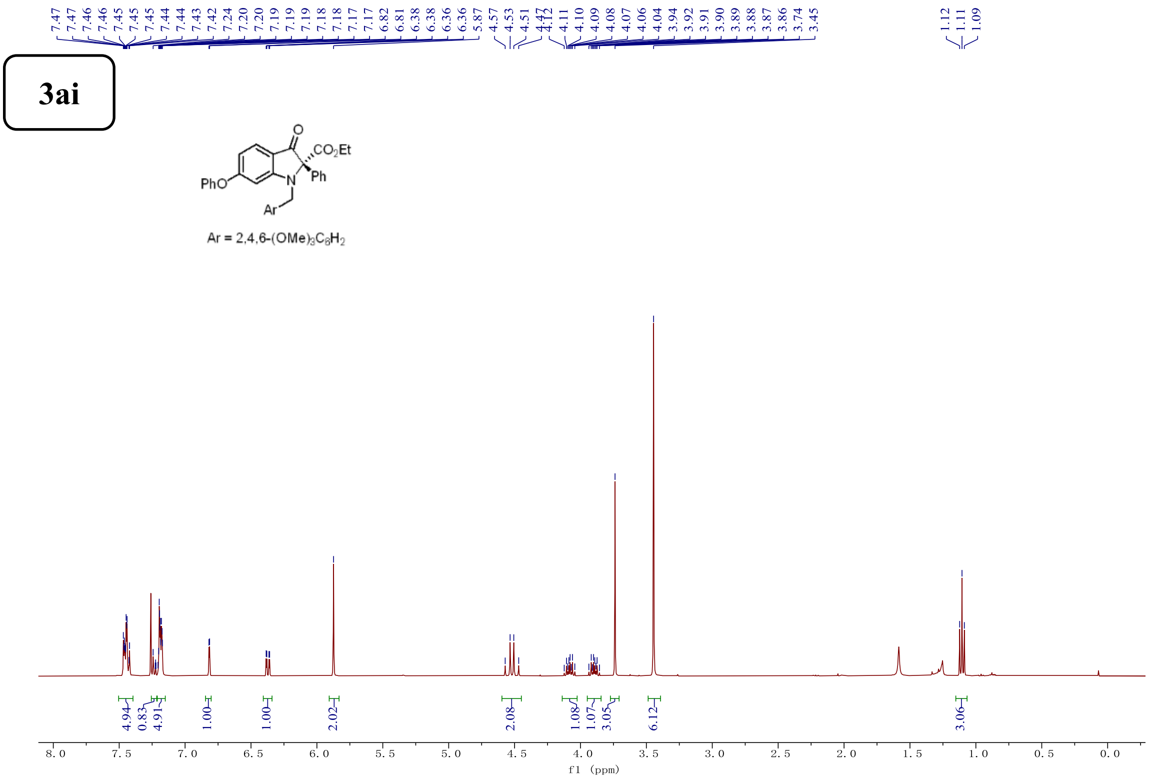

7.47 7.47 7.46 7.45 7.45 7.45 7.44 7.44 7.43 7.42 7.24 7.20 7.20 7.19 7.19 7.19 7.18 7.18 7.17 7.17 6.82 6.81 6.38 6.38 6.36 6.36 5.87 4.57 4.53 4.51 4.47 4.12 4.11 4.10 4.09 4.08 4.07 4.06 4.04 3.94 3.92 3.91 3.90 3.89 3.88 3.87 3.86 3.74 3.45 1.12 1.11 1.09
PhO
CO2Et
Ph
N
Ar
O
Ar = 2,4,6-(OMe)3C6H2
4.94 0.83 4.91 1.00 1.00 2.02 2.08 1.08 1.07 3.05 6.12 3.06
f1 (ppm)

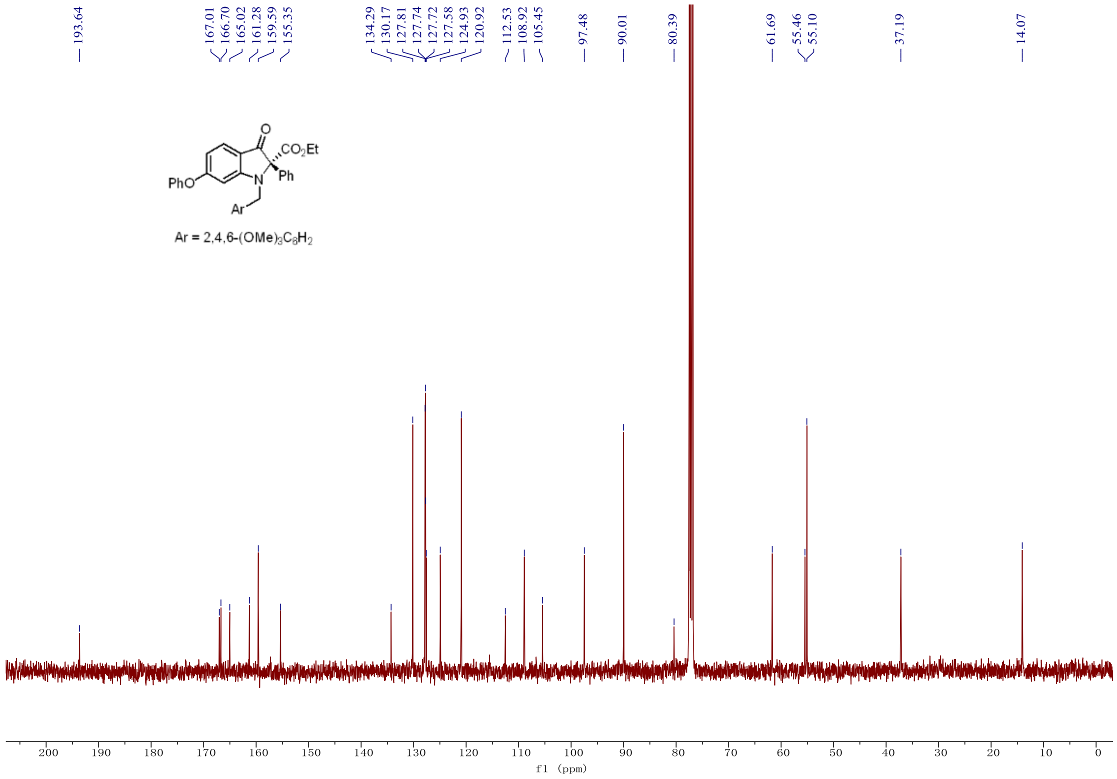

193.64 167.01 166.70 165.02 161.28 159.59 155.35 134.29 130.17 127.81 127.74 127.72 127.58 124.93 120.92 112.53 108.92 105.45 97.48 90.01 80.39 61.69 55.46 55.10 37.19 14.07
PhO
CO2Et
Ph
N
Ar
O
Ar = 2,4,6-(OMe)3C6H2
f1 (ppm)

3aj

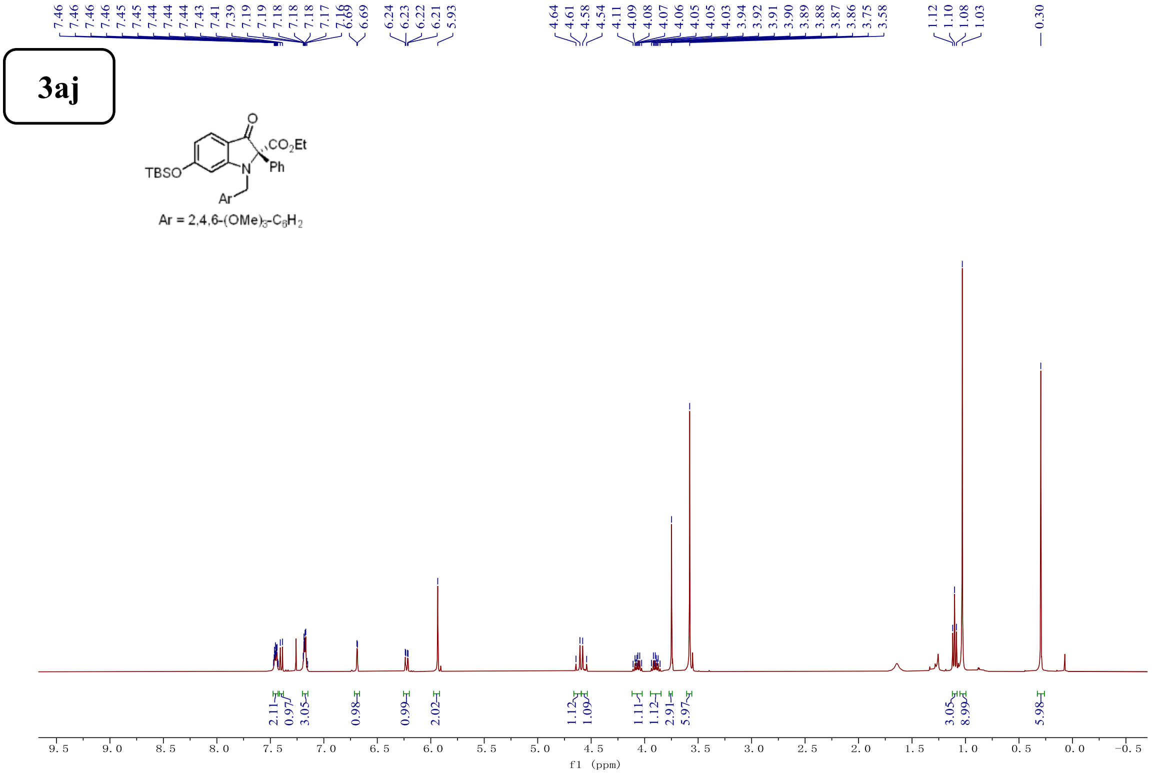
7.46 7.46 7.46 7.45 7.45 7.44 7.44 7.44 7.43 7.41 7.39 7.19 7.19 7.18 7.18 7.18 7.17 7.16 6.69 6.69 6.24 6.23 6.22 6.21 5.93
4.64 4.61 4.58 4.54 4.11 4.09 4.08 4.07 4.06 4.05 4.03 3.94 3.92 3.91 3.90 3.89 3.88 3.87 3.86 3.75 3.58
1.12 1.10 1.08 1.03
0.30
TBSO
CO2Et
Ph
N
Ar
Ar = 2,4,6-(OMe)3-C6H2
2.11 0.97 3.05 0.98 0.99 2.02 1.12 1.09 1.11 1.12 2.91 5.97 3.05 8.99 5.98
9.5 9.0 8.5 8.0 7.5 7.0 6.5 6.0 5.5 5.0 4.5 4.0 3.5 3.0 2.5 2.0 1.5 1.0 0.5 0.0 -0.5
f1 (ppm)

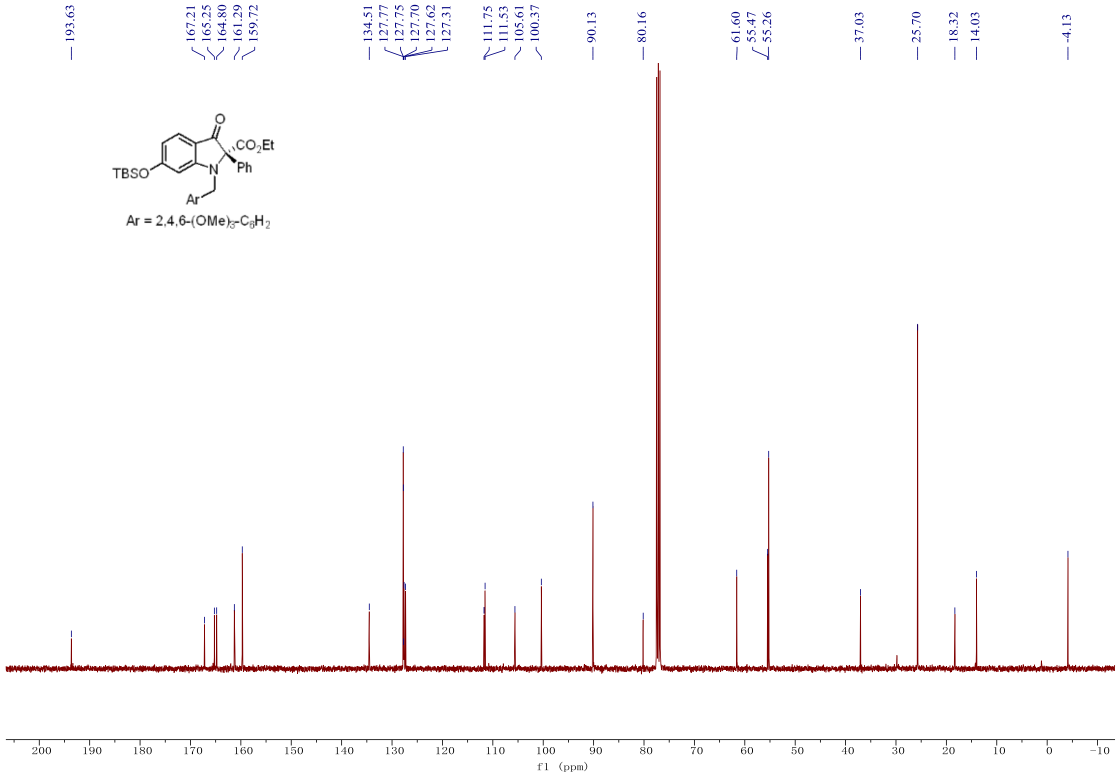
193.63
167.21 165.25 164.80 161.29 159.72
134.51 127.77 127.75 127.70 127.62 127.31
111.75 111.53 105.61 100.37
90.13
80.16
61.60 55.47 55.26
37.03
25.70
18.32
14.03
-4.13
TBSO
CO2Et
Ph
N
Ar
Ar = 2,4,6-(OMe)3-C6H2
200 190 180 170 160 150 140 130 120 110 100 90 80 70 60 50 40 30 20 10 0 -10
f1 (ppm)

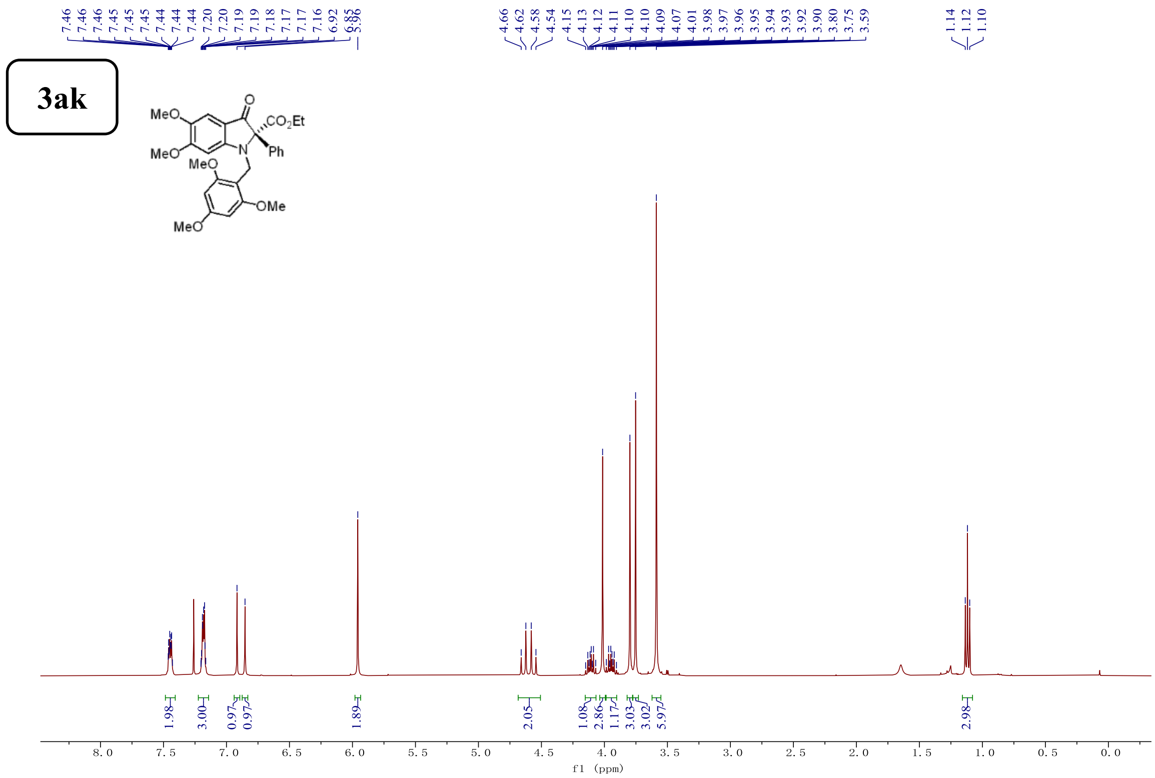
3ak
MeO
MeO
CO2Et
Ph
N
O
OMe
f1 (ppm)

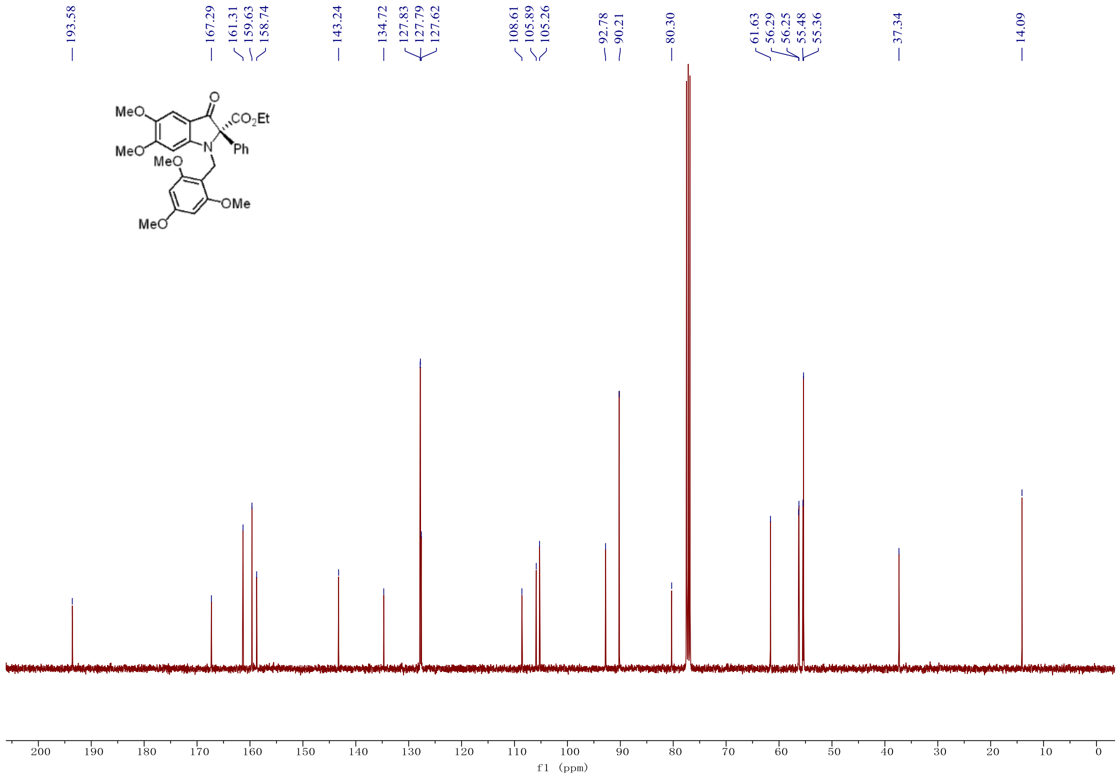
193.58
167.29
161.31
159.63
158.74
143.24
134.72
127.83
127.79
127.62
108.61
105.89
105.26
92.78
90.21
80.30
61.63
56.29
56.25
55.48
55.36
37.34
14.09
f1 (ppm)

3al

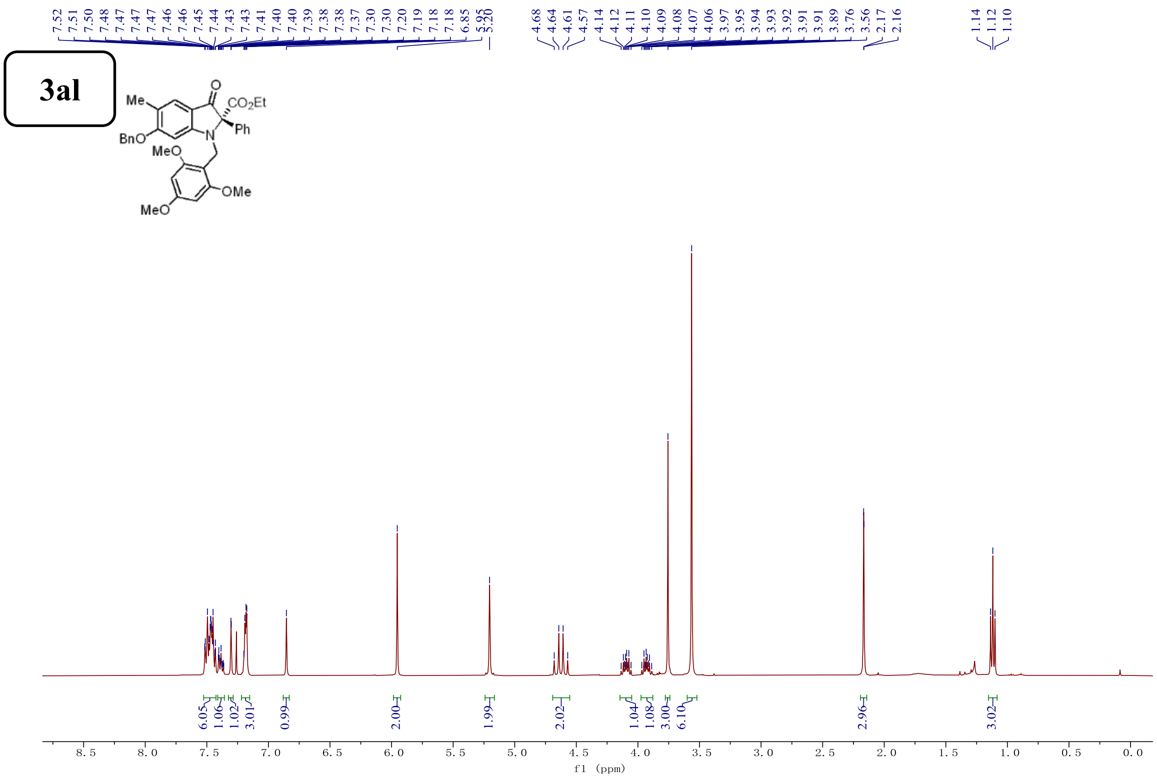
7.52 7.51 7.50 7.48 7.47 7.47 7.46 7.45 7.44 7.43 7.43 7.41 7.40 7.39 7.38 7.38 7.37 7.30 7.20 7.19 7.18 7.18 6.85 5.95 5.20
4.68 4.64 4.61 4.57 4.14 4.12 4.11 4.10 4.09 4.08 4.07 4.06 3.97 3.95 3.94 3.93 3.92 3.91 3.91 3.89 3.76 3.56 2.17 2.16
1.14 1.12 1.10
Me
O
CO_2Et
Ph
BnO
N
MeO
OMe
MeO
6.05 1.06 1.02 3.01 0.99 2.00 1.99 2.02 1.04 1.08 3.00 6.10 2.96 3.02
8.5 8.0 7.5 7.0 6.5 6.0 5.5 5.0 4.5 4.0 3.5 3.0 2.5 2.0 1.5 1.0 0.5 0.0
f1 (ppm)

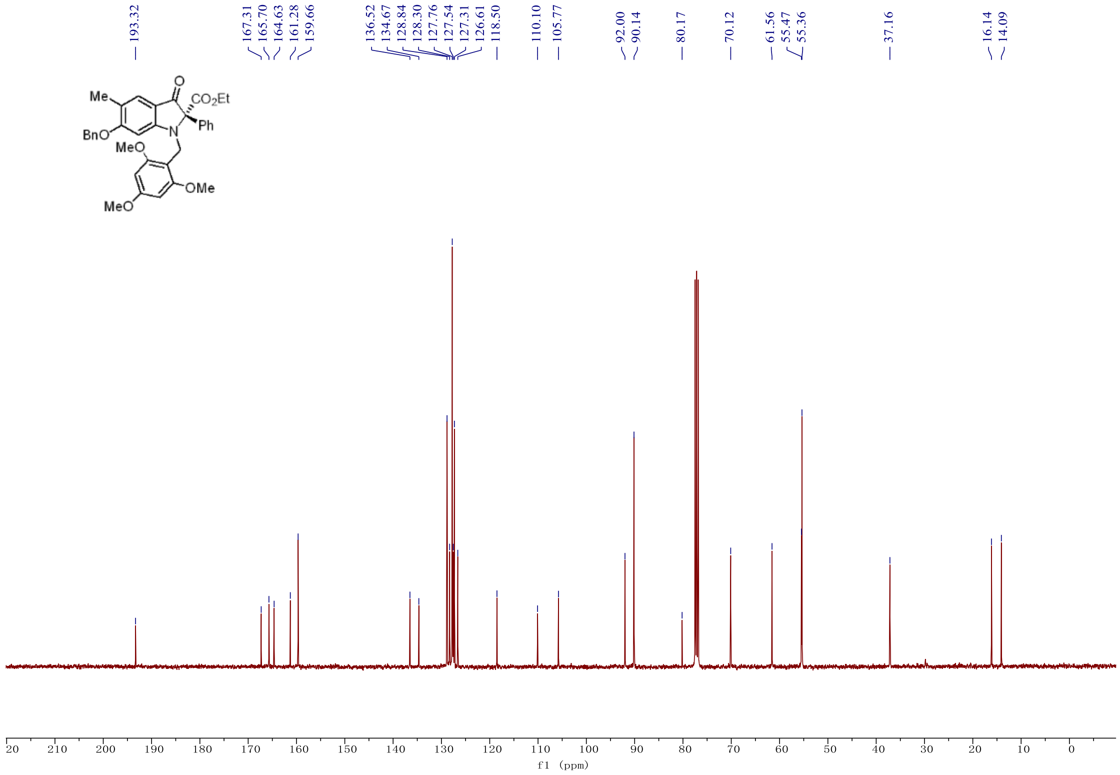
193.32
167.31 165.70 164.63 161.28 159.66
136.52 134.67 128.84 128.30 127.76 127.54 127.31 126.61 118.50
110.10 105.77
92.00 90.14
80.17
70.12
61.56 55.47 55.36
37.16
16.14 14.09
Me
O
CO_2Et
Ph
BnO
N
MeO
OMe
MeO
20 210 200 190 180 170 160 150 140 130 120 110 100 90 80 70 60 50 40 30 20 10 0
f1 (ppm)

3am

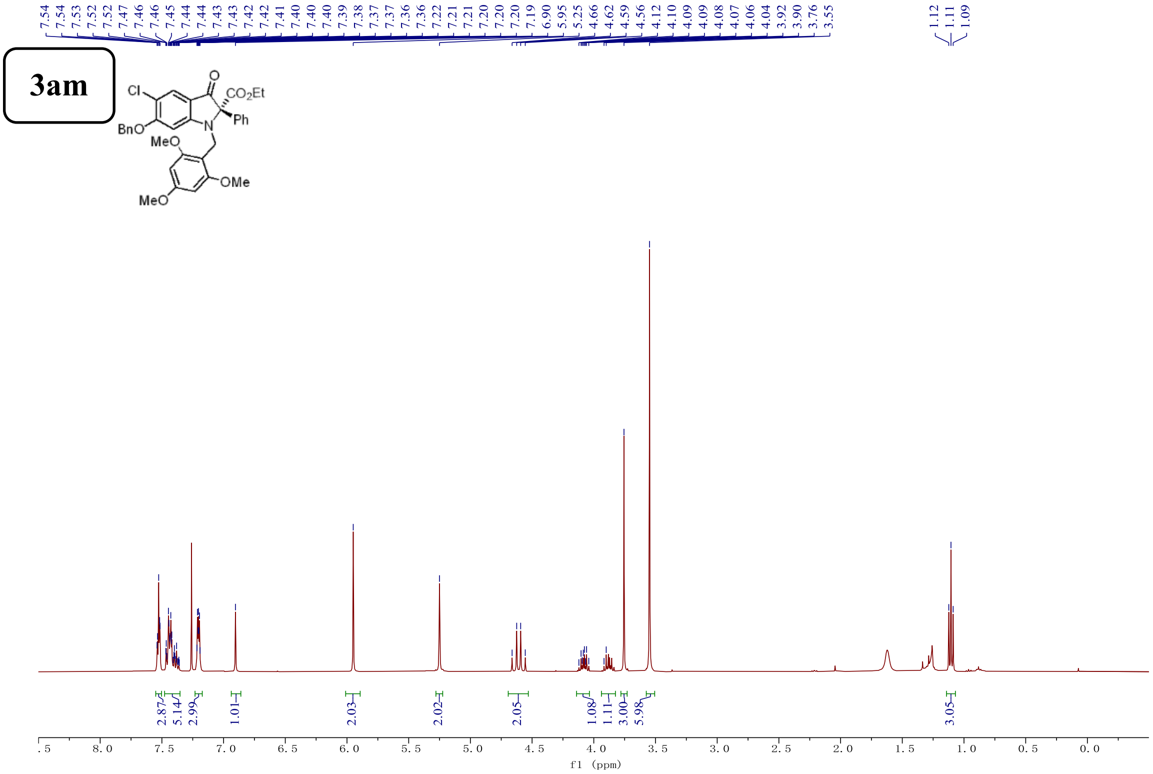
7.54 7.54 7.53 7.52 7.52 7.47 7.46 7.46 7.45 7.44 7.44 7.43 7.42 7.42 7.41 7.40 7.40 7.40 7.39 7.38 7.37 7.36 7.36 7.22 7.21 7.21 7.20 7.20 7.19 6.90 5.95 5.25 4.66 4.62 4.59 4.56 4.12 4.10 4.09 4.09 4.08 4.07 4.06 4.04 3.92 3.90 3.76 3.55
1.12 1.11 1.09
Cl
O
CO_2Et
Ph
N
BnO
MeO
OMe
MeO
2.87 5.14 2.99 1.01 2.03 2.02 2.05 1.08 1.11 3.00 5.98 3.05
f1 (ppm)

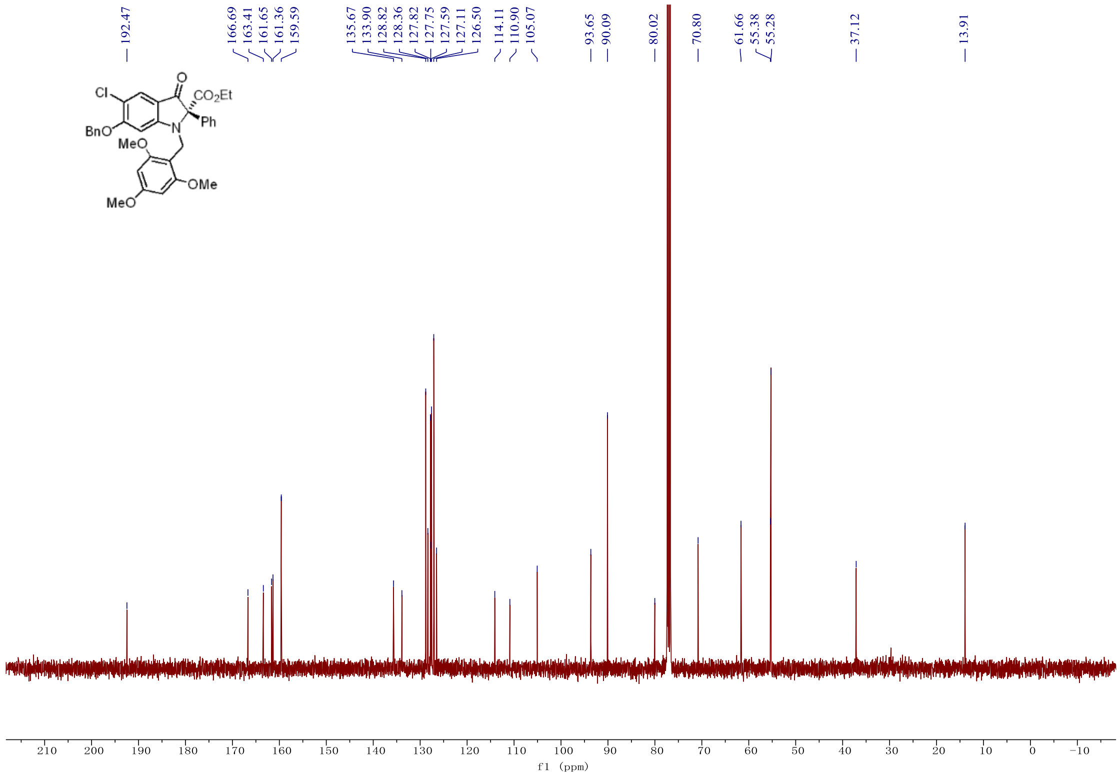
192.47
166.69 163.41 161.65 161.36 159.59
135.67 133.90 128.82 128.36 127.82 127.75 127.59 127.11 126.50
114.11 110.90 105.07
93.65 90.09
80.02
70.80
61.66 55.38 55.28
37.12
13.91
Cl
O
CO_2Et
Ph
N
BnO
MeO
OMe
MeO
f1 (ppm)

3an

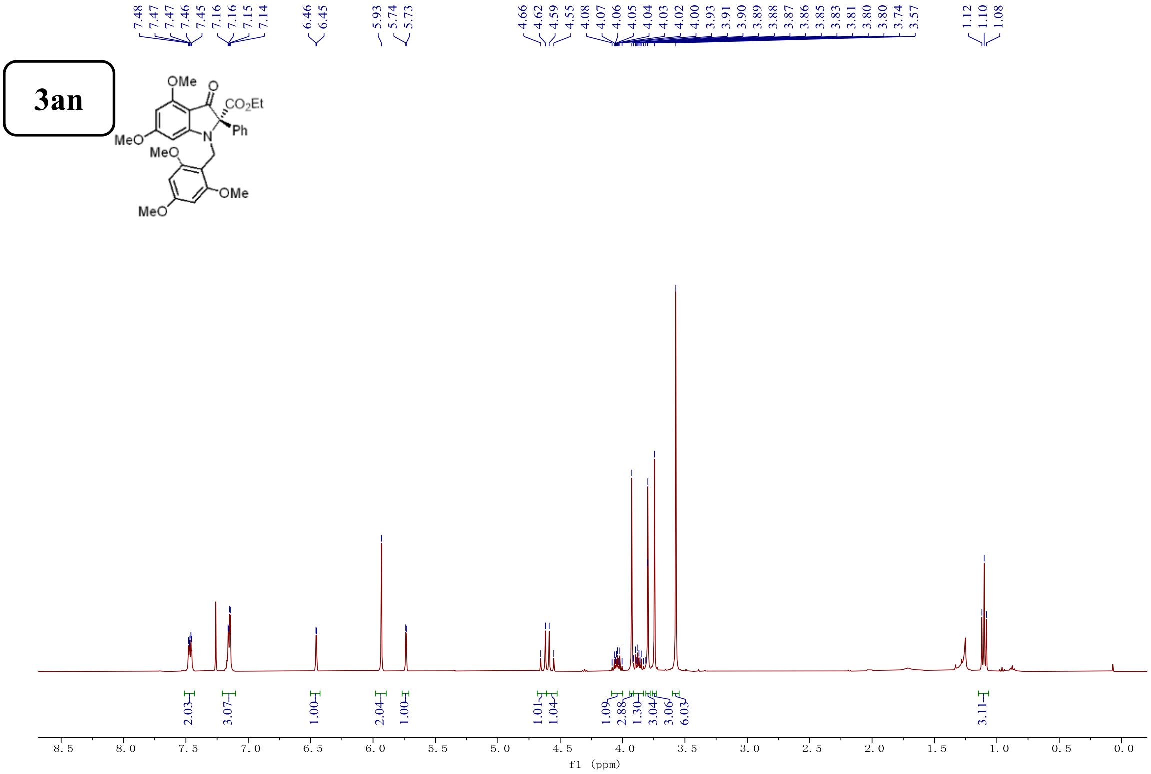

7.48
7.47
7.47
7.46
7.45
7.16
7.16
7.15
7.14
6.46
6.45
5.93
5.74
5.73
4.66
4.62
4.59
4.55
4.08
4.07
4.06
4.05
4.04
4.03
4.02
4.00
3.93
3.91
3.90
3.89
3.88
3.87
3.86
3.85
3.83
3.81
3.80
3.80
3.74
3.57
1.12
1.10
1.08
OMe
O
CO2Et
Ph
MeO
N
MeO
OMe
MeO
2.03
3.07
1.00
2.04
1.00
1.01
1.04
1.09
2.88
1.30
3.04
3.06
6.03
3.11
f1 (ppm)

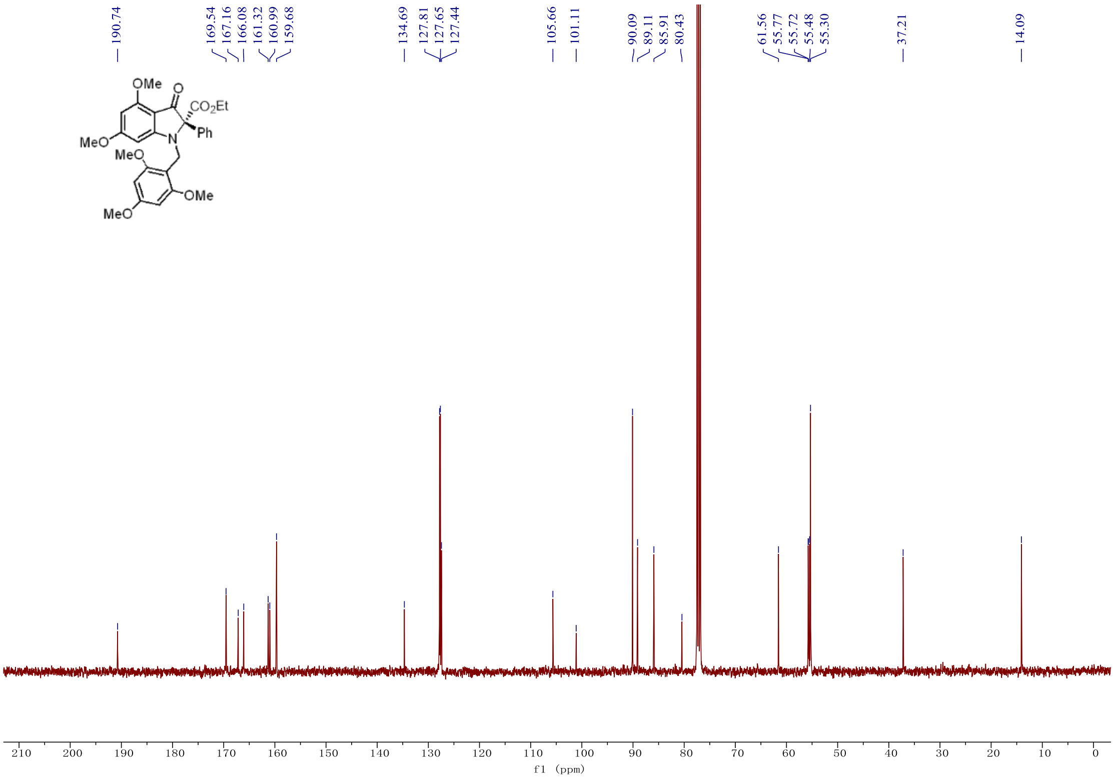

190.74
169.54
167.16
166.08
161.32
160.99
159.68
134.69
127.81
127.65
127.44
105.66
101.11
90.09
89.11
85.91
80.43
61.56
55.77
55.72
55.48
55.30
37.21
14.09
OMe
O
CO2Et
Ph
MeO
N
MeO
OMe
MeO
f1 (ppm)

3ao

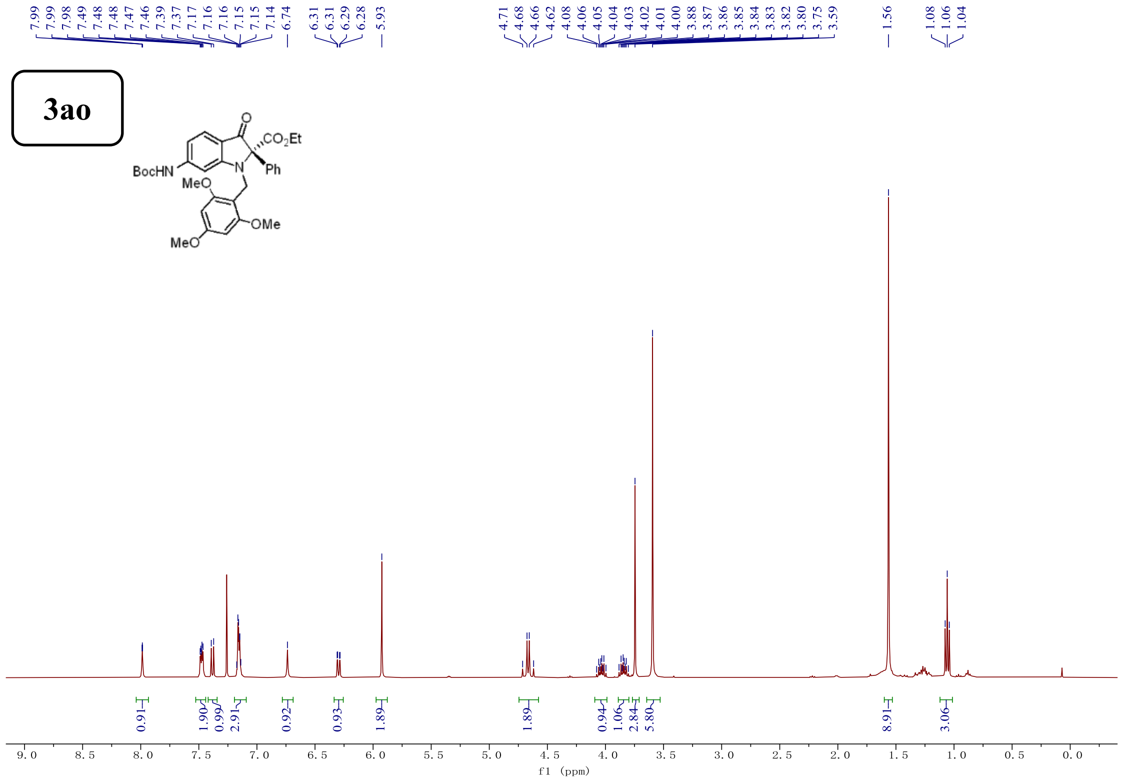

7.99 7.99 7.98 7.49 7.48 7.48 7.47 7.46 7.39 7.37 7.17 7.16 7.16 7.15 7.15 7.14 6.74 6.31 6.31 6.29 6.28 5.93
4.71 4.68 4.66 4.62 4.08 4.06 4.05 4.04 4.03 4.02 4.01 4.00 3.88 3.87 3.86 3.85 3.84 3.83 3.82 3.80 3.75 3.59
1.56 1.08 1.06 1.04
BocHN
MeO
OMe
MeO
CO2Et
Ph
N
O
0.91 1.90 0.99 2.91 0.92 0.93 1.89 1.89 0.94 1.06 2.84 5.80 8.91 3.06
f1 (ppm)

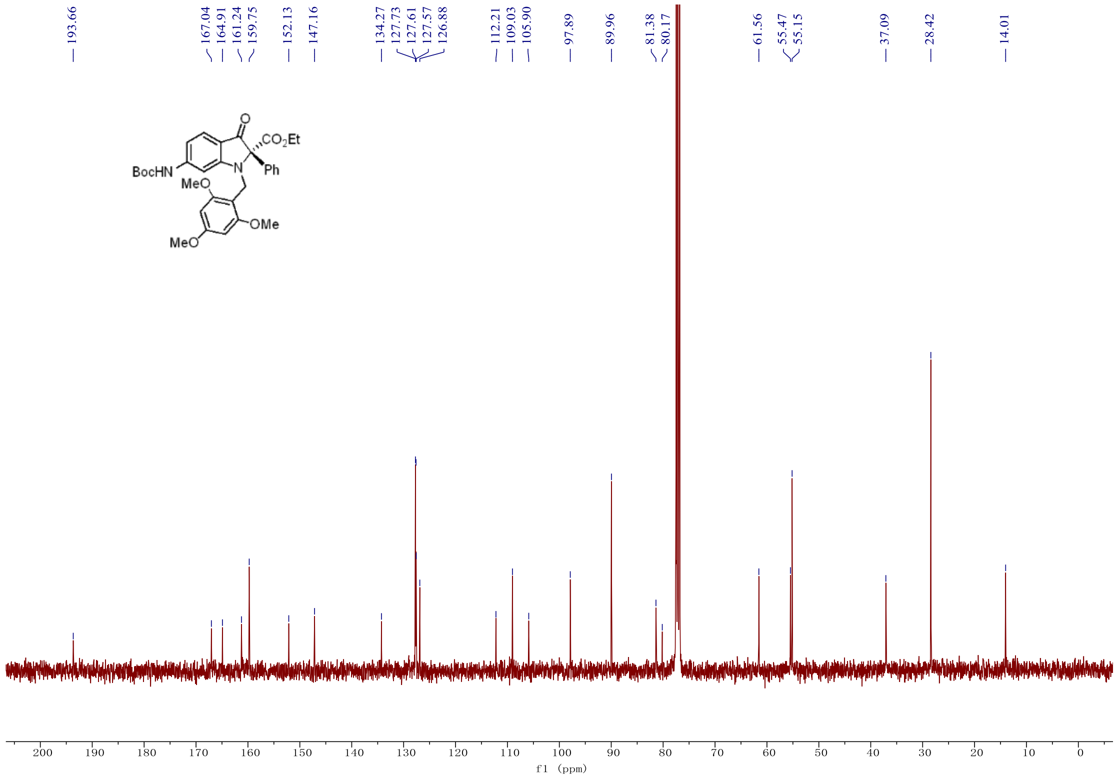

193.66 167.04 164.91 161.24 159.75 152.13 147.16 134.27 127.73 127.61 127.57 126.88 112.21 109.03 105.90 97.89 89.96 81.38 80.17 61.56 55.47 55.15 37.09 28.42 14.01
BocHN
MeO
OMe
MeO
CO2Et
Ph
N
O
f1 (ppm)

3ap

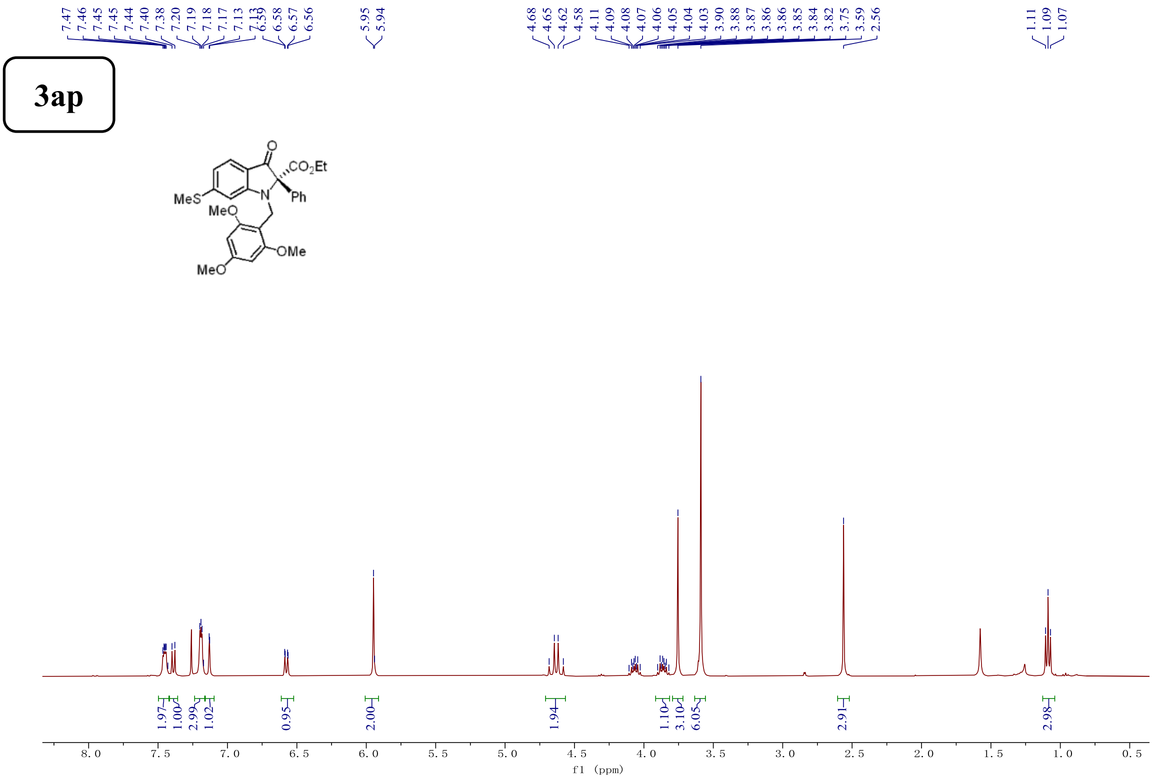
7.47
7.46
7.45
7.45
7.44
7.40
7.38
7.20
7.19
7.18
7.17
7.13
6.59
6.58
6.57
6.56
5.95
5.94
4.68
4.65
4.62
4.58
4.11
4.09
4.08
4.07
4.06
4.05
4.04
4.03
3.90
3.88
3.87
3.86
3.86
3.85
3.84
3.82
3.75
3.59
2.56
1.11
1.09
1.07
CO2Et
Ph
MeS
N
O
MeO
OMe
MeO
1.97
1.00
2.99
1.02
0.95
2.00
1.94
1.10
3.10
6.05
2.91
2.98
f1 (ppm)

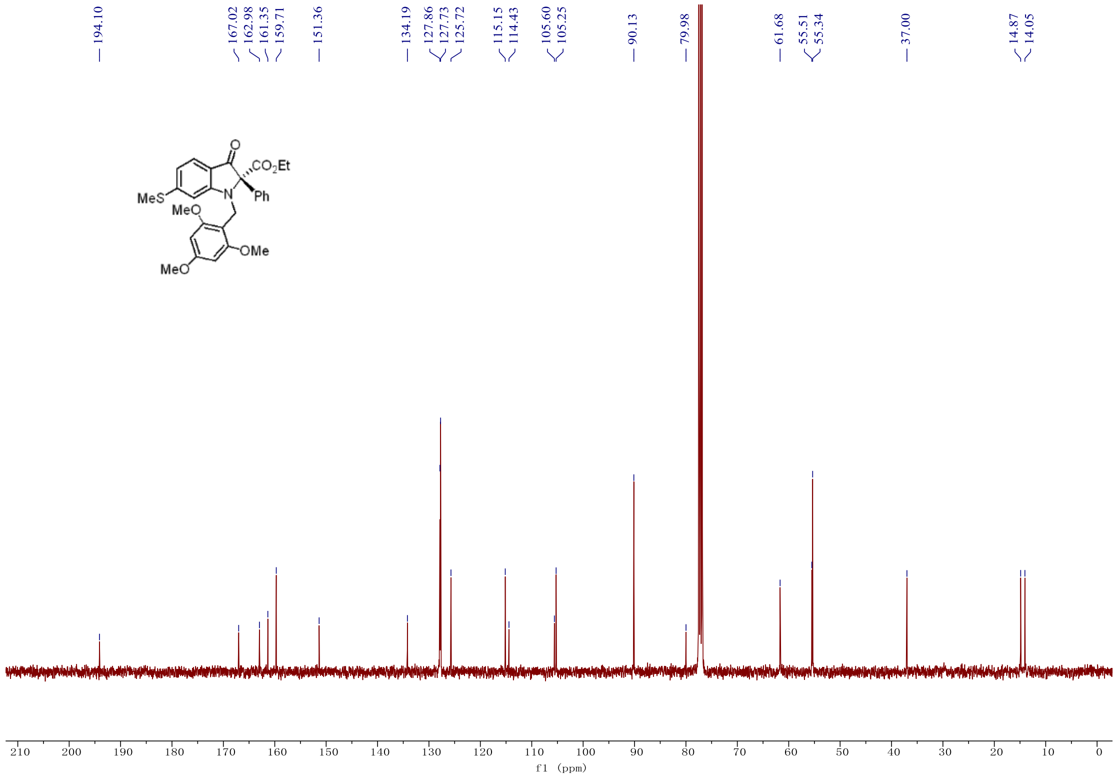
194.10
167.02
162.98
161.35
159.71
151.36
134.19
127.86
127.73
125.72
115.15
114.43
105.60
105.25
90.13
79.98
61.68
55.51
55.34
37.00
14.87
14.05
CO2Et
Ph
MeS
N
O
MeO
OMe
MeO
f1 (ppm)

3aq

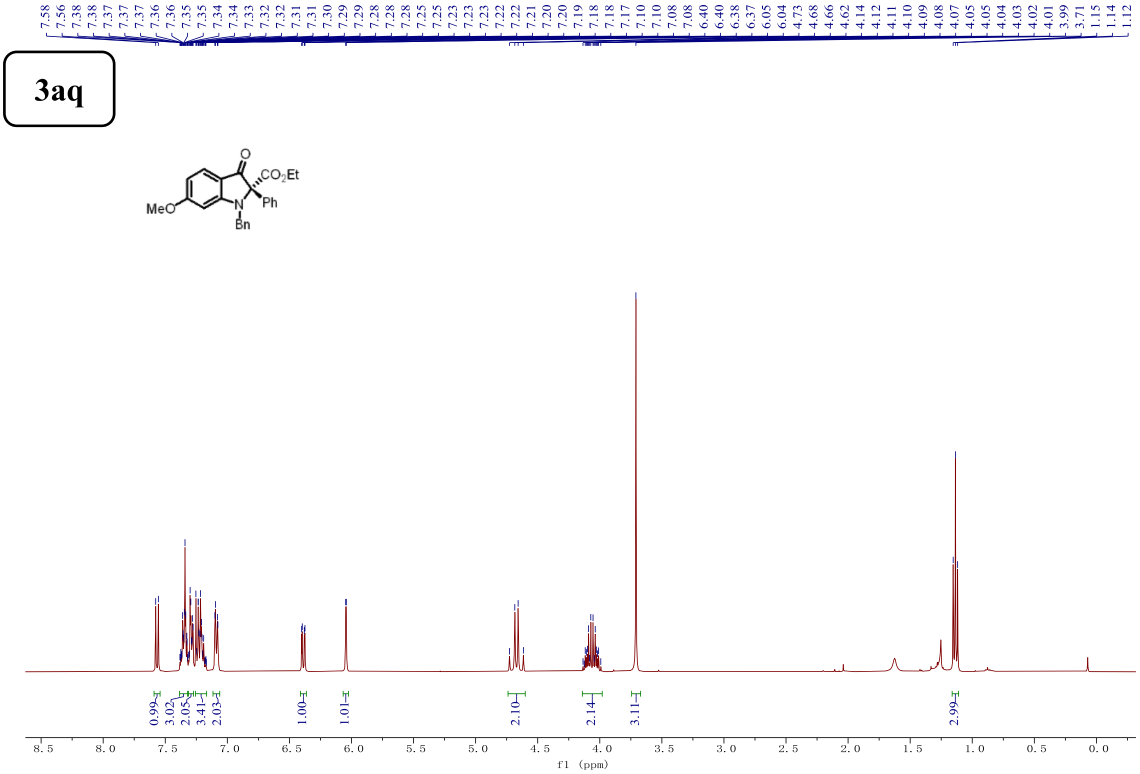
7.58 7.56 7.38 7.38 7.37 7.37 7.36 7.36 7.35 7.35 7.34 7.34 7.33 7.32 7.32 7.31 7.31 7.30 7.29 7.29 7.28 7.28 7.28 7.25 7.25 7.23 7.23 7.23 7.22 7.22 7.21 7.20 7.20 7.19 7.18 7.18 7.17 7.10 7.10 7.08 7.08 6.40 6.40 6.38 6.37 6.05 6.04 4.73 4.68 4.66 4.62 4.14 4.12 4.11 4.10 4.09 4.08 4.07 4.05 4.05 4.04 4.03 4.02 4.01 3.99 3.71 1.15 1.14 1.12
O
CO_2Et
Ph
MeO
N
Bn
0.99 3.02 2.05 3.41 2.03 1.00 1.01 2.10 2.14 3.11 2.99
8.5 8.0 7.5 7.0 6.5 6.0 5.5 5.0 4.5 4.0 3.5 3.0 2.5 2.0 1.5 1.0 0.5 0.0
f1 (ppm)

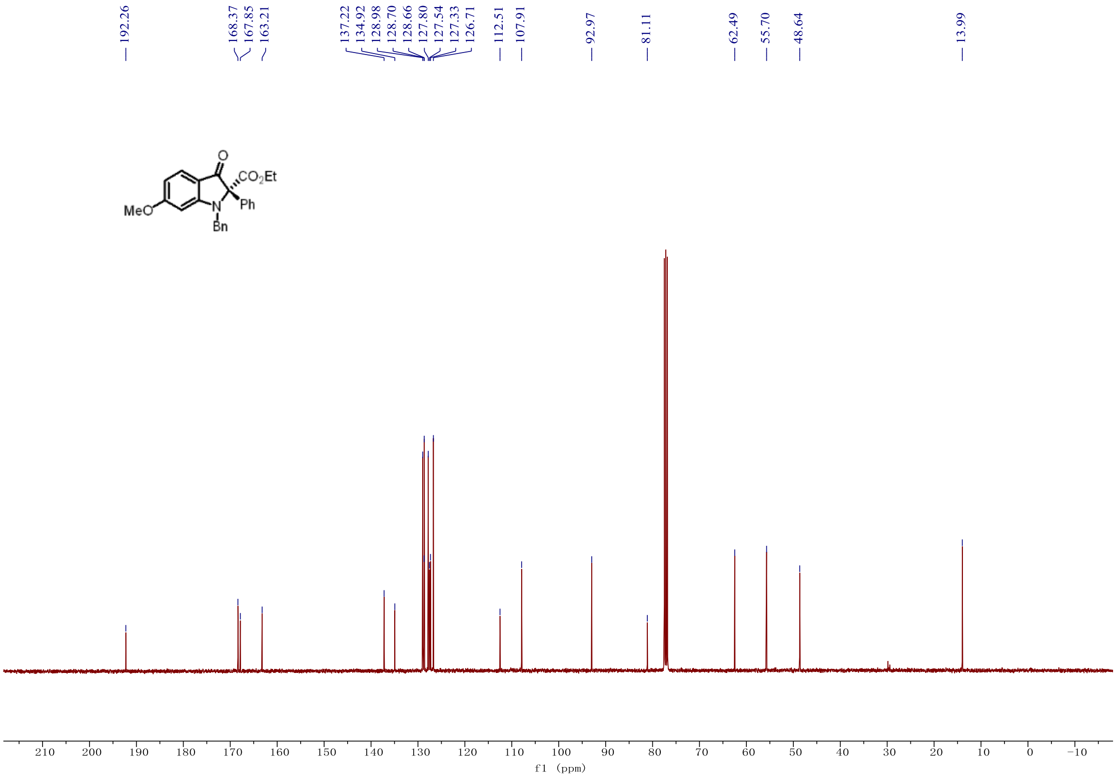
192.26 168.37 167.85 163.21 137.22 134.92 128.98 128.70 128.66 127.80 127.54 127.33 126.71 112.51 107.91 92.97 81.11 62.49 55.70 48.64 13.99
O
CO_2Et
Ph
MeO
N
Bn
210 200 190 180 170 160 150 140 130 120 110 100 90 80 70 60 50 40 30 20 10 0 -10
f1 (ppm)

3ar

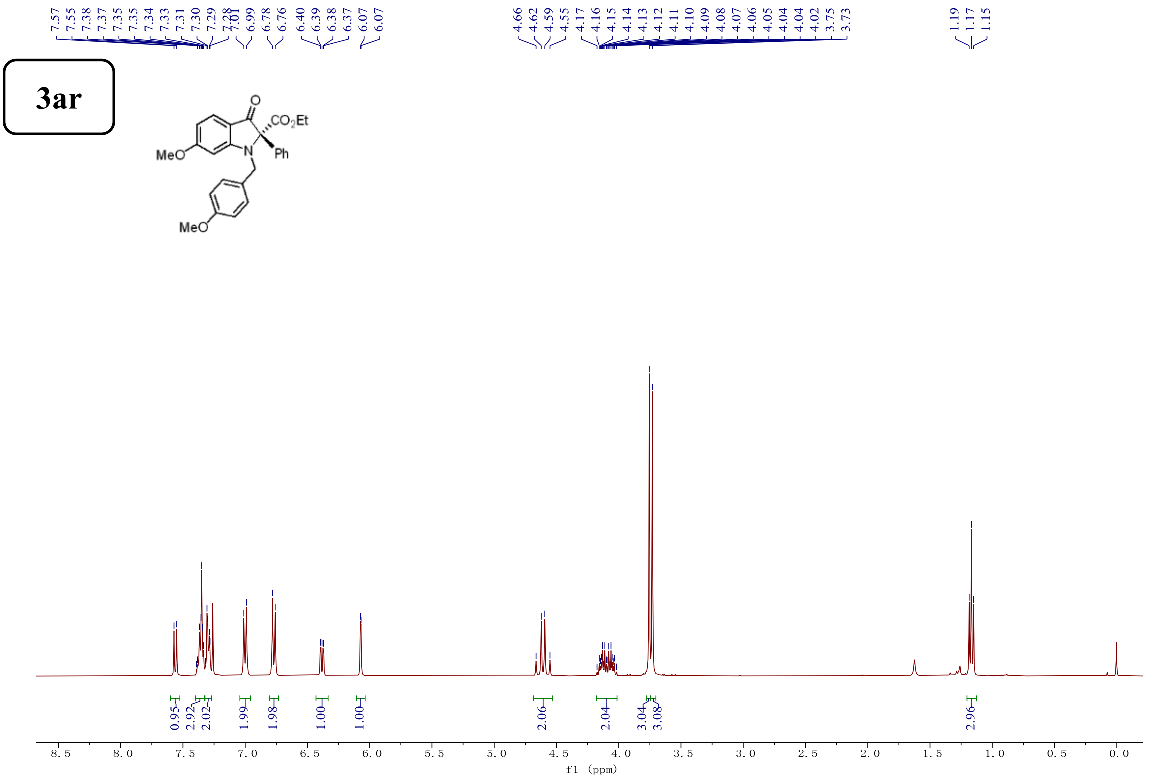
7.57 7.55 7.38 7.37 7.35 7.35 7.34 7.33 7.31 7.30 7.29 7.28 7.01 6.99 6.78 6.76 6.40 6.39 6.38 6.37 6.07 6.07
4.66 4.62 4.59 4.55 4.17 4.16 4.15 4.14 4.13 4.12 4.11 4.10 4.09 4.08 4.07 4.06 4.05 4.04 4.04 4.02 3.75 3.73
1.19 1.17 1.15
O
CO_2Et
MeO
N
Ph
MeO
0.95 2.92 2.02 1.99 1.98 1.00 1.00 2.06 2.04 3.04 3.08 2.96
8.5 8.0 7.5 7.0 6.5 6.0 5.5 5.0 4.5 4.0 3.5 3.0 2.5 2.0 1.5 1.0 0.5 0.0
f1 (ppm)

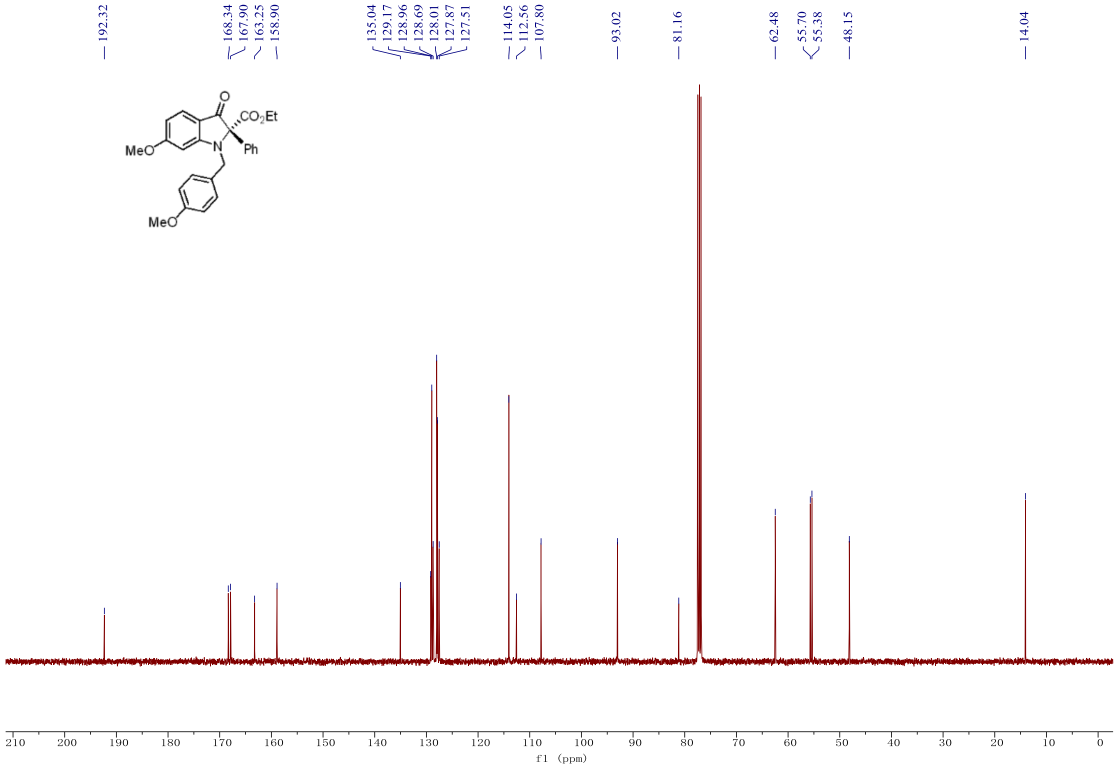
192.32
168.34 167.90 163.25 158.90
135.04 129.17 128.96 128.69 128.01 127.87 127.51
114.05 112.56 107.80
93.02
81.16
62.48
55.70 55.38
48.15
14.04
O
CO_2Et
MeO
N
Ph
MeO
210 200 190 180 170 160 150 140 130 120 110 100 90 80 70 60 50 40 30 20 10 0
f1 (ppm)

3as

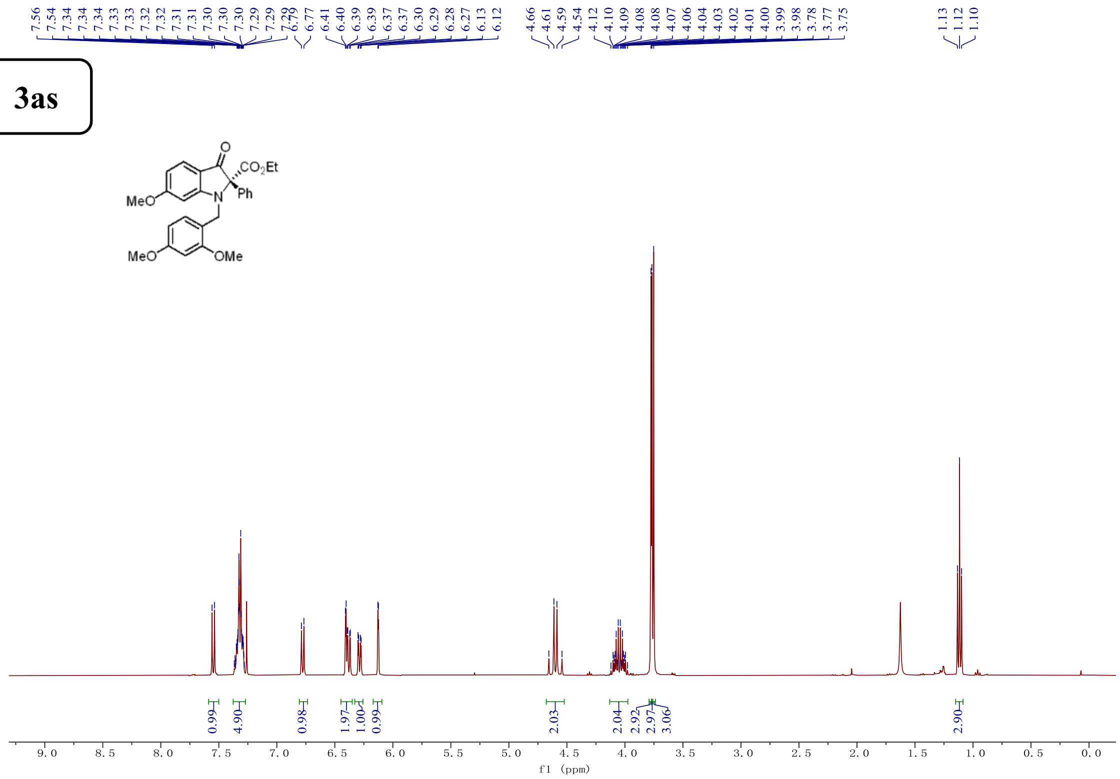

7.56 7.54 7.34 7.34 7.34 7.33 7.33 7.32 7.32 7.31 7.31 7.30 7.30 7.30 7.29 7.29 7.29 6.79 6.77 6.41 6.40 6.39 6.39 6.37 6.37 6.30 6.29 6.28 6.27 6.13 6.12
4.66 4.61 4.59 4.54 4.12 4.10 4.09 4.08 4.08 4.07 4.06 4.04 4.03 4.02 4.01 4.00 3.99 3.98 3.78 3.77 3.75
1.13 1.12 1.10
O
CO_2Et
MeO
N
Ph
MeO
OMe
0.99 4.90 0.98 1.97 1.00 0.99 2.03 2.04 2.92 2.97 3.06 2.90
9.0 8.5 8.0 7.5 7.0 6.5 6.0 5.5 5.0 4.5 4.0 3.5 3.0 2.5 2.0 1.5 1.0 0.5 0.0
f1 (ppm)

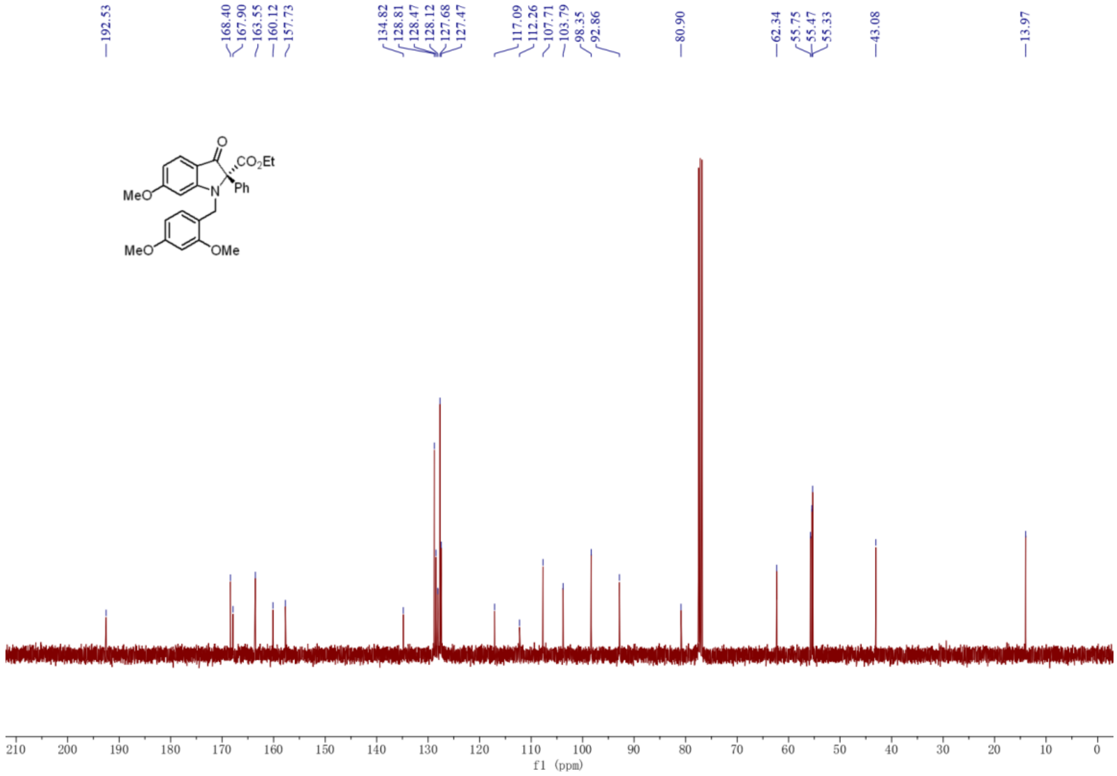

192.53
168.40 167.90 163.55 160.12 157.73
134.82 128.81 128.47 128.12 127.68 127.47
117.09 112.26 107.71 103.79 98.35 92.86
80.90
62.34 55.75 55.47 55.33
43.08
13.97
O
CO_2Et
MeO
N
Ph
MeO
OMe
210 200 190 180 170 160 150 140 130 120 110 100 90 80 70 60 50 40 30 20 10 0
f1 (ppm)

3at

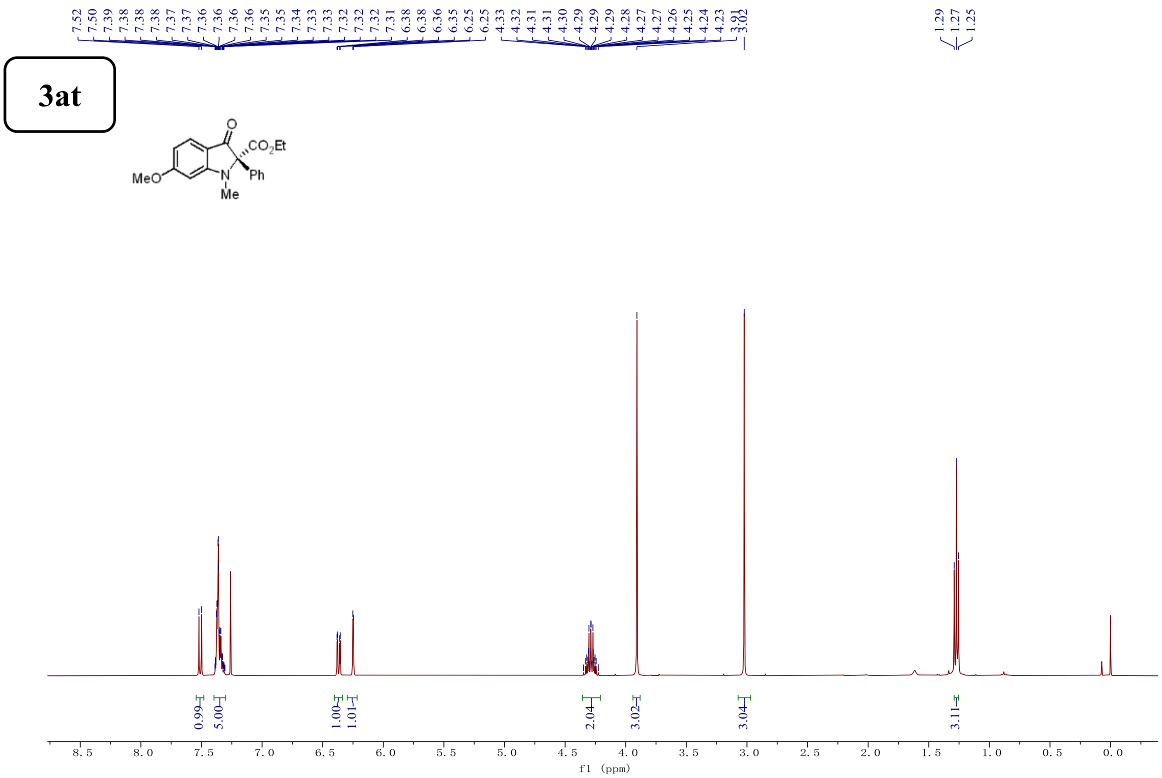
7.52 7.50 7.39 7.38 7.38 7.38 7.37 7.37 7.36 7.36 7.36 7.36 7.35 7.35 7.34 7.33 7.33 7.32 7.32 7.32 7.31 6.38 6.38 6.36 6.35 6.25 6.25 4.33 4.32 4.31 4.31 4.30 4.29 4.29 4.29 4.28 4.27 4.27 4.26 4.25 4.24 4.23 3.91 3.02
1.29 1.27 1.25
O
CO2Et
MeO
N
Ph
Me
0.99 5.00 1.00 1.01 2.04 3.02 3.04 3.11
8.5 8.0 7.5 7.0 6.5 6.0 5.5 5.0 4.5 4.0 3.5 3.0 2.5 2.0 1.5 1.0 0.5 0.0
f1 (ppm)

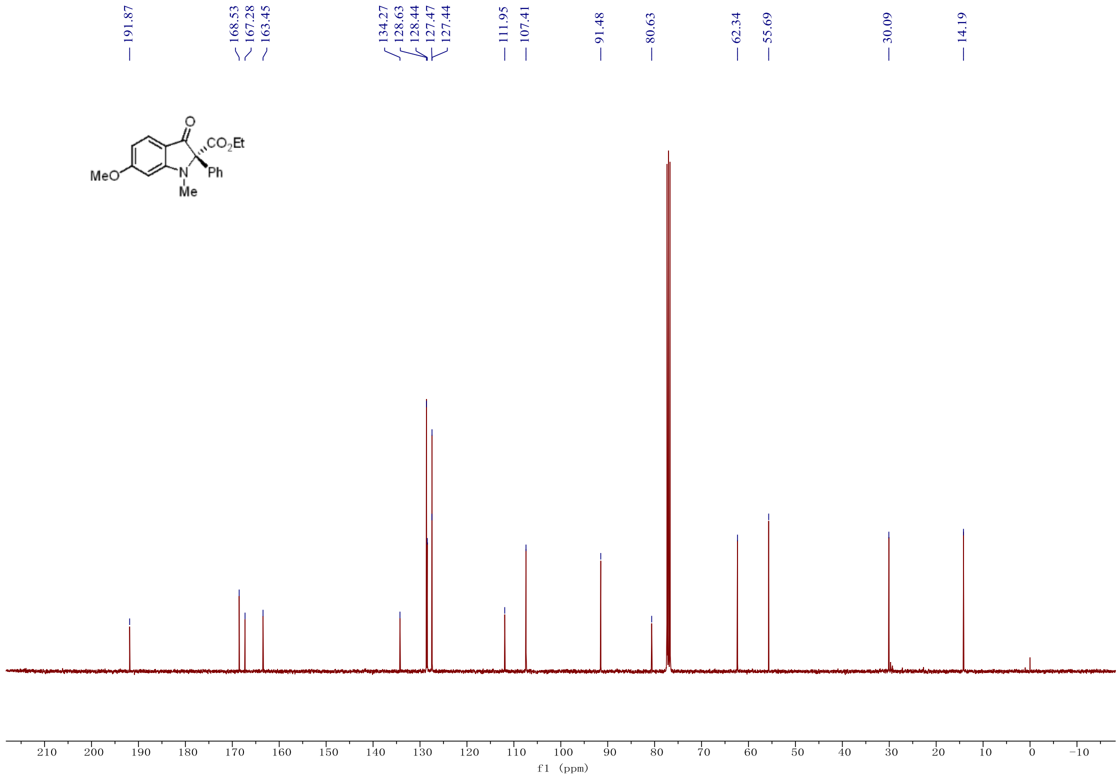
191.87
168.53 167.28 163.45
134.27 128.63 128.44 127.47 127.44
111.95 107.41
91.48
80.63
62.34 55.69
30.09
14.19
O
CO2Et
MeO
N
Ph
Me
210 200 190 180 170 160 150 140 130 120 110 100 90 80 70 60 50 40 30 20 10 0 -10
f1 (ppm)

3au

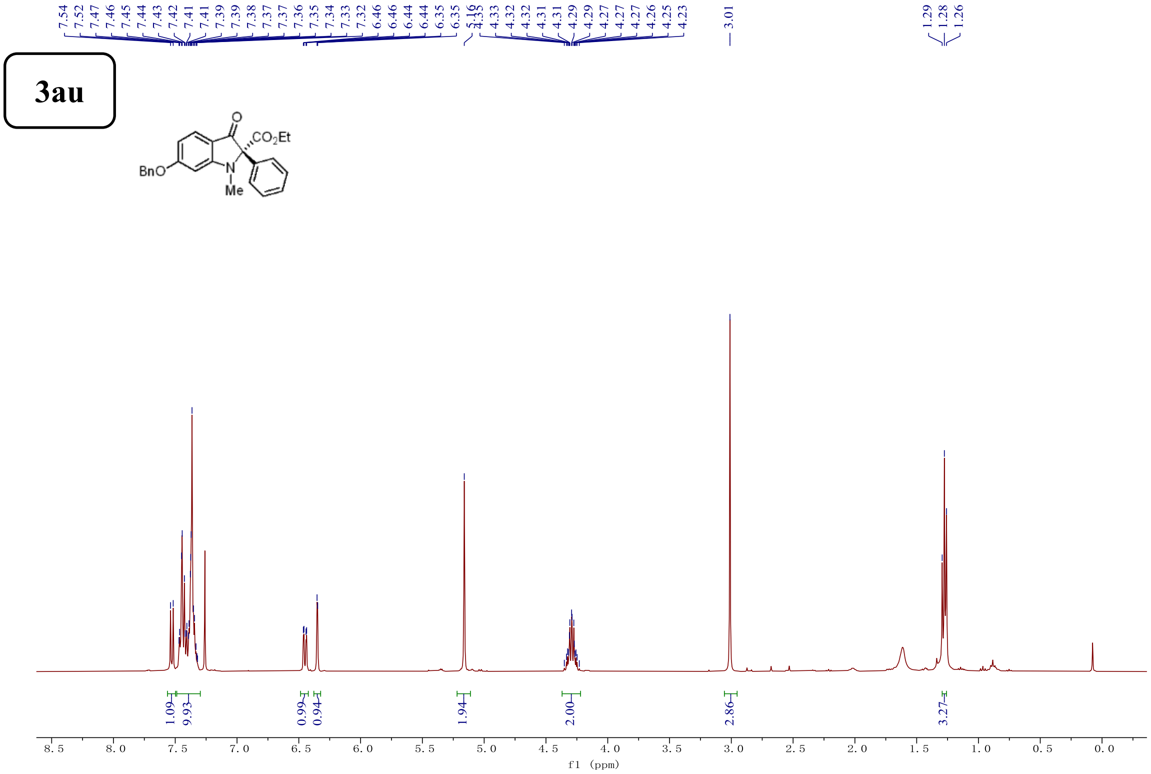
7.54
7.52
7.47
7.46
7.45
7.44
7.43
7.42
7.41
7.39
7.39
7.38
7.37
7.37
7.36
7.35
7.34
7.33
7.32
6.46
6.46
6.44
6.44
6.35
6.35
5.16
4.35
4.33
4.32
4.32
4.31
4.31
4.29
4.29
4.27
4.27
4.26
4.25
4.23
3.01
1.29
1.28
1.26
CO2Et
BnO
N
Me
O
1.09
9.93
0.99
0.94
1.94
2.00
2.86
3.27
f1 (ppm)

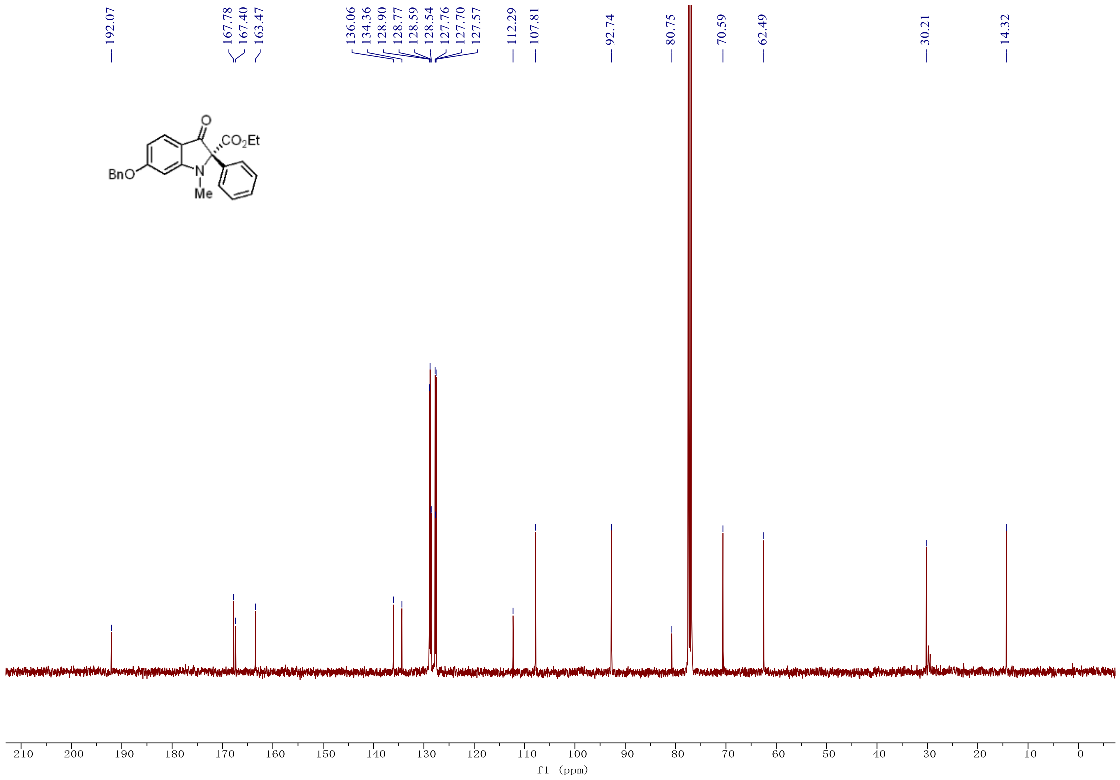
192.07
167.78
167.40
163.47
136.06
134.36
128.90
128.77
128.59
128.54
127.76
127.70
127.57
112.29
107.81
92.74
80.75
70.59
62.49
30.21
14.32
CO2Et
BnO
N
Me
O
f1 (ppm)

3av

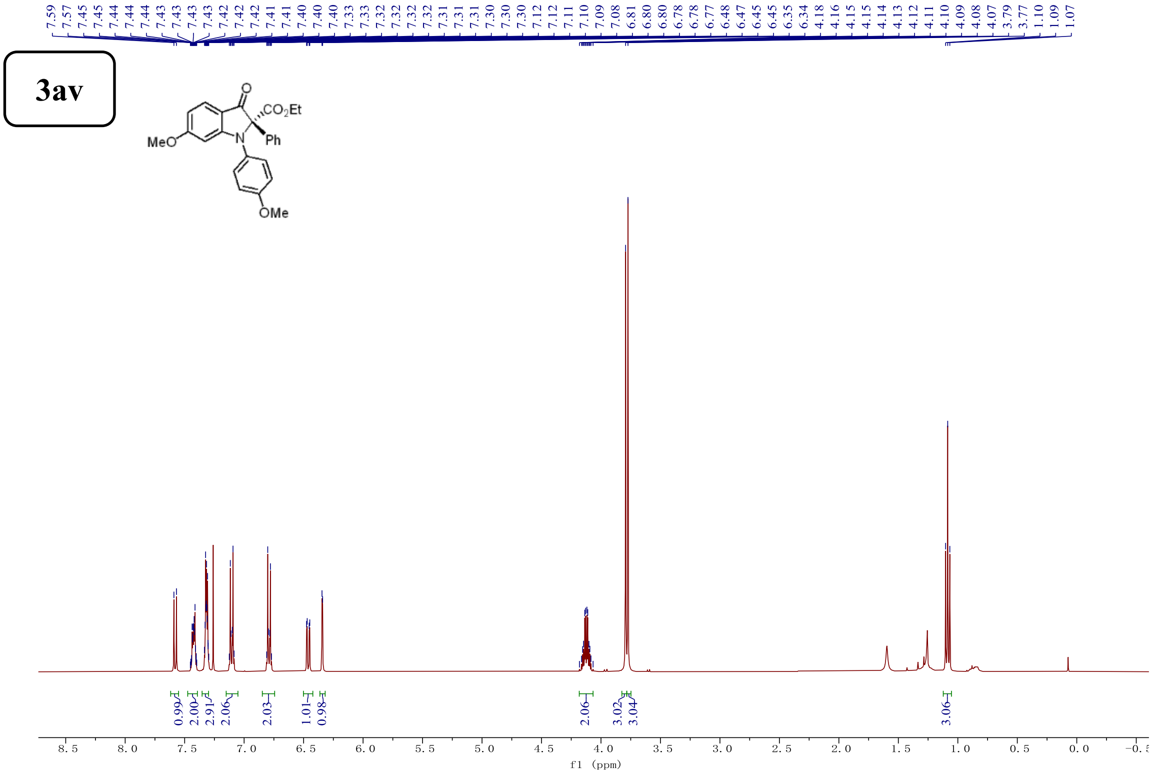
7.59 7.57 7.45 7.45 7.44 7.44 7.43 7.43 7.43 7.43 7.42 7.42 7.41 7.41 7.40 7.40 7.40 7.33 7.33 7.32 7.32 7.32 7.32 7.31 7.31 7.30 7.30 7.30 7.12 7.12 7.11 7.10 7.09 7.08 6.81 6.80 6.80 6.78 6.78 6.77 6.48 6.47 6.45 6.45 6.35 6.34 4.18 4.16 4.15 4.15 4.14 4.13 4.12 4.11 4.10 4.09 4.08 4.07 3.79 3.77 1.10 1.09 1.07
CO2Et
MeO
N
Ph
OMe
0.99 2.00 2.91 2.06 2.03 1.01 0.98 2.06 3.02 3.04 3.06
f1 (ppm)

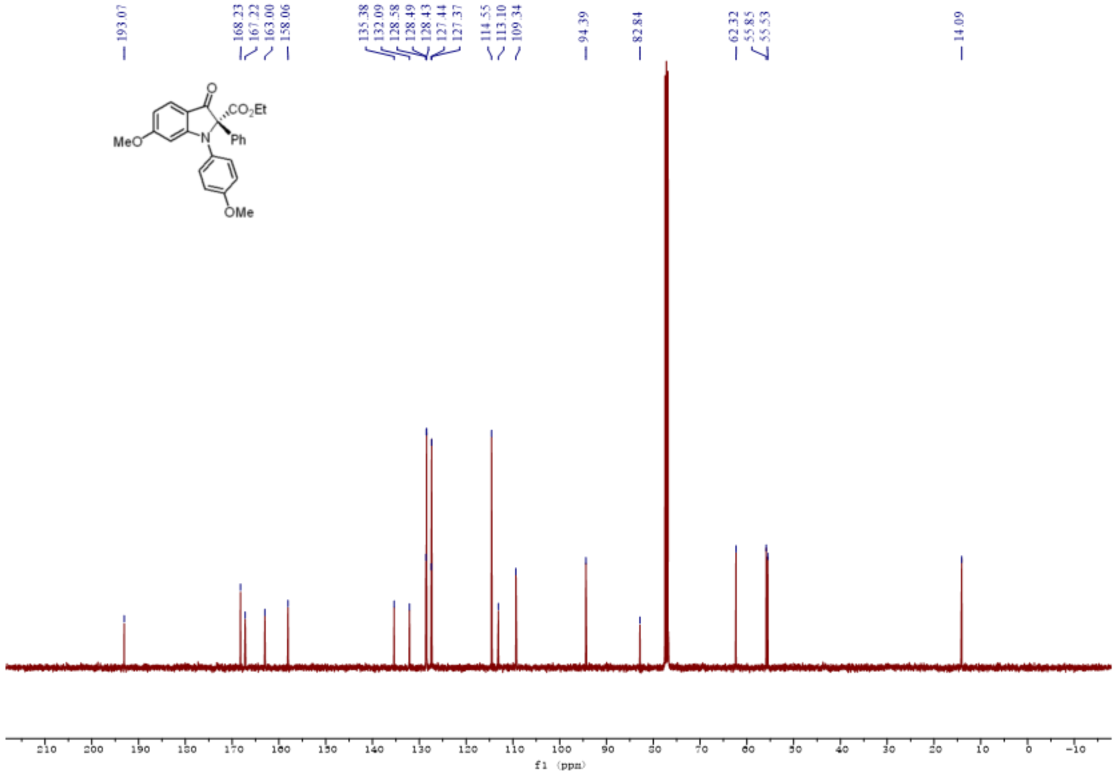
193.07 168.23 167.22 163.00 158.06 135.38 132.09 128.58 128.49 128.43 127.44 127.37 114.55 113.10 109.34 94.39 82.84 62.32 55.85 55.53 14.09
CO2Et
MeO
N
Ph
OMe
f1 (ppm)

3aw

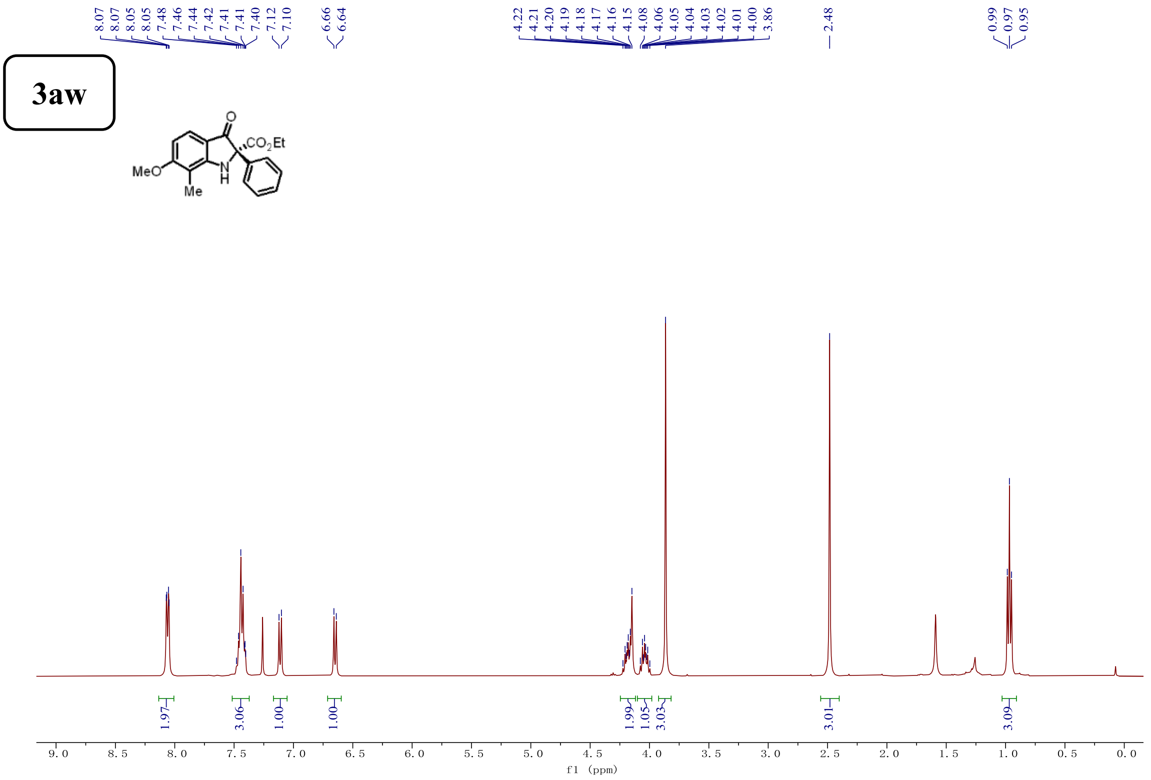
8.07 8.07 8.05 8.05 7.48 7.46 7.44 7.42 7.41 7.41 7.40 7.12 7.10 6.66 6.64
4.22 4.21 4.20 4.19 4.18 4.17 4.16 4.15 4.08 4.06 4.05 4.04 4.03 4.02 4.01 4.00 3.86
2.48
0.99 0.97 0.95
MeO
Me
N
H
O
CO2Et
1.97 3.06 1.00 1.00 1.99 1.05 3.03 3.01 3.09
f1 (ppm)

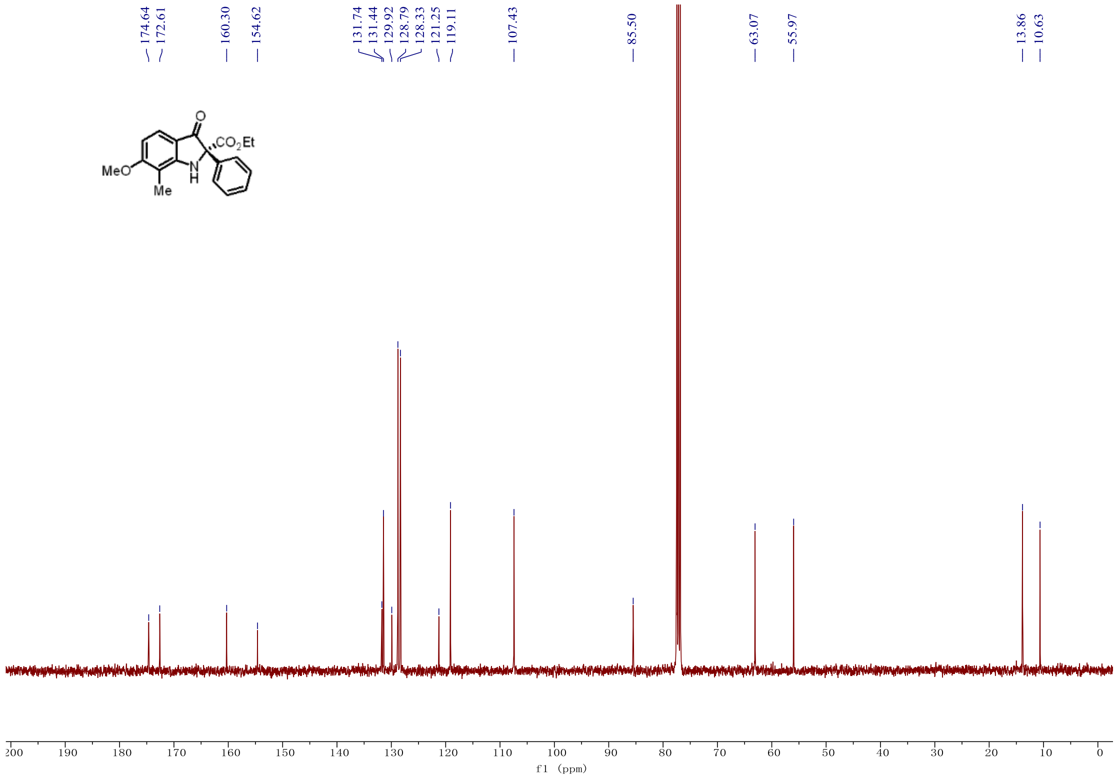
174.64 172.61 160.30 154.62 131.74 131.44 129.92 128.79 128.33 121.25 119.11 107.43 85.50 63.07 55.97 13.86 10.63
MeO
Me
N
H
O
CO2Et
f1 (ppm)

3ax

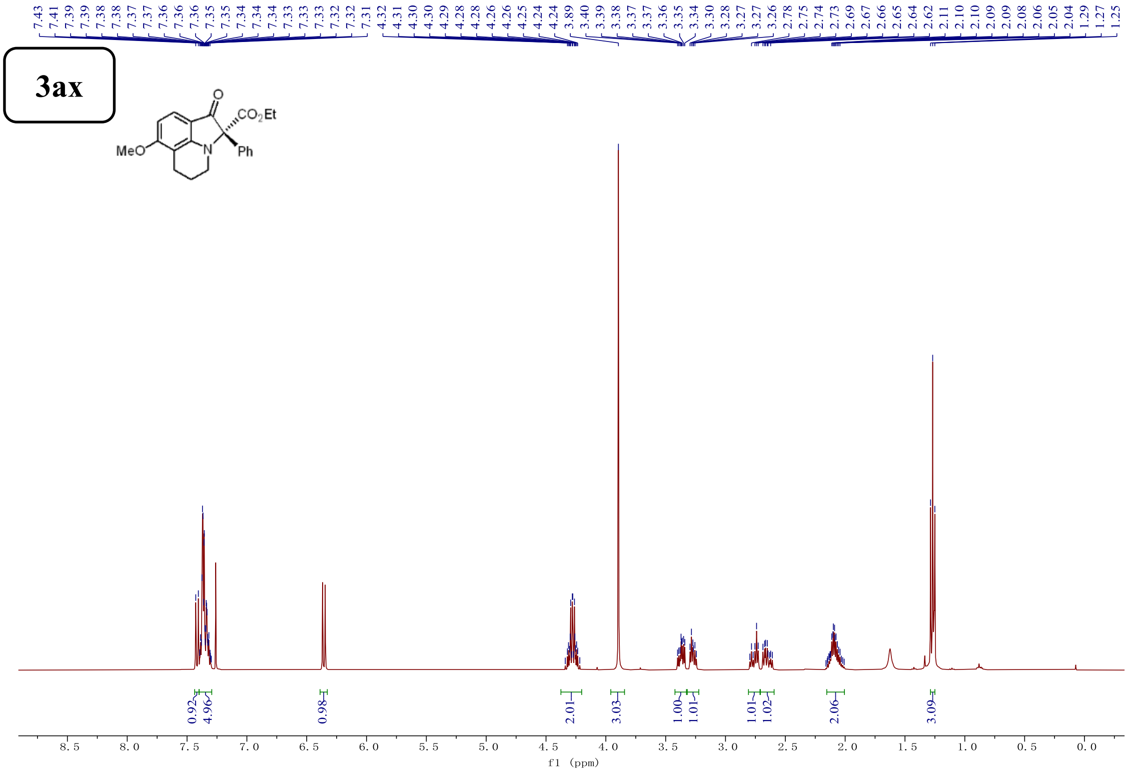

7.43
7.41
7.39
7.39
7.38
7.38
7.37
7.37
7.36
7.36
7.35
7.35
7.34
7.34
7.34
7.33
7.33
7.32
7.31
4.32
4.31
4.30
4.30
4.29
4.28
4.28
4.26
4.26
4.25
4.24
4.24
3.89
3.40
3.39
3.38
3.37
3.37
3.36
3.35
3.34
3.30
3.28
3.27
3.27
3.26
2.78
2.75
2.74
2.73
2.69
2.67
2.66
2.65
2.64
2.62
2.11
2.10
2.10
2.09
2.09
2.08
2.06
2.05
2.04
1.29
1.27
1.25
CO2Et
MeO
N
Ph
O
0.92
4.96
0.98
2.01
3.03
1.00
1.01
1.01
1.02
2.06
3.09
f1 (ppm)

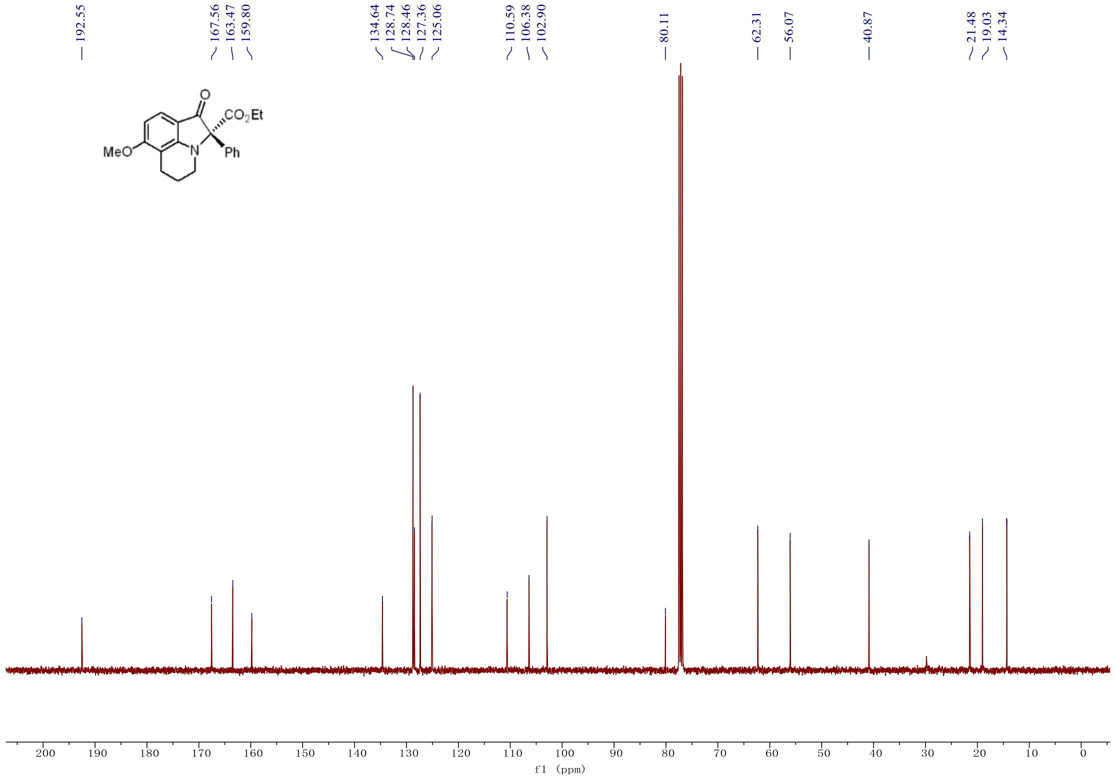

192.55
167.56
163.47
159.80
134.64
128.74
128.46
127.36
125.06
110.59
106.38
102.90
80.11
62.31
56.07
40.87
21.48
19.03
14.34
CO2Et
MeO
N
Ph
O
f1 (ppm)

3a’

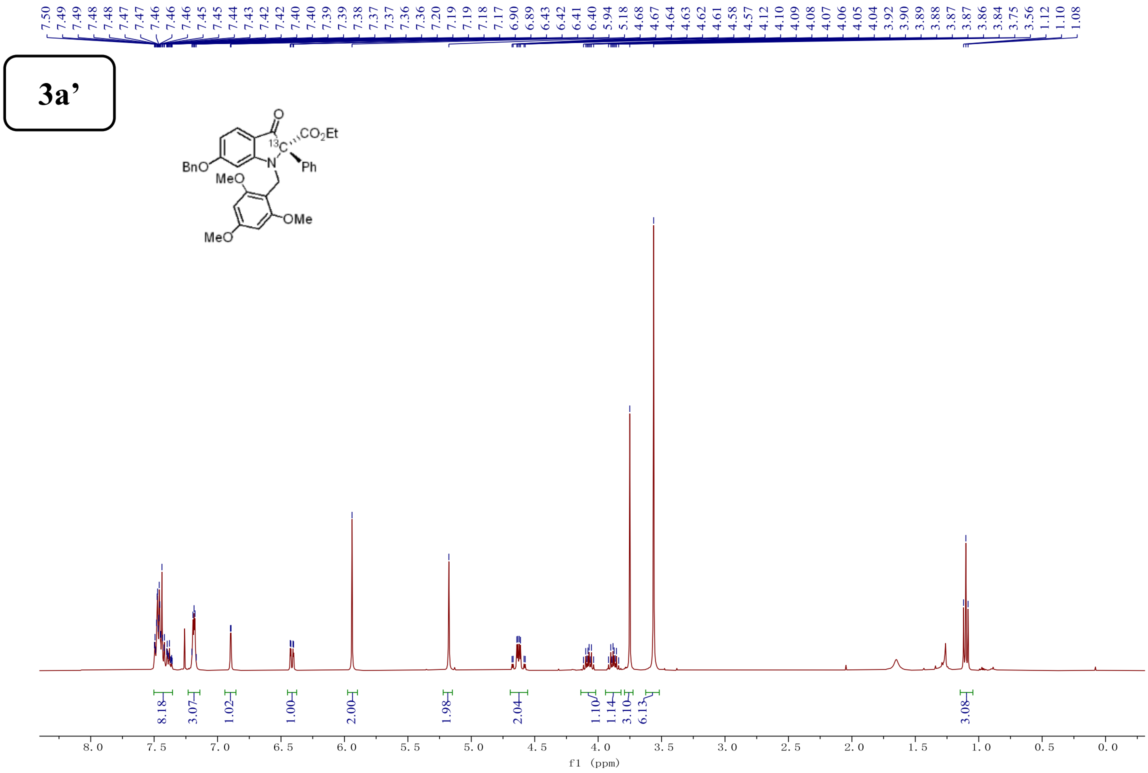
7.50 7.49 7.48 7.48 7.47 7.46 7.46 7.46 7.45 7.45 7.44 7.43 7.42 7.40 7.40 7.39 7.39 7.38 7.37 7.36 7.36 7.20 7.19 7.19 7.18 7.17 6.90 6.89 6.43 6.42 6.41 6.40 5.94 5.18 4.68 4.67 4.64 4.63 4.62 4.61 4.58 4.57 4.12 4.10 4.09 4.08 4.07 4.06 4.05 4.04 3.92 3.90 3.89 3.88 3.87 3.87 3.86 3.84 3.75 3.56 1.12 1.10 1.08
CO2Et
13C
Ph
BnO
N
MeO
OMe
MeO
8.18 3.07 1.02 1.00 2.00 1.98 2.04 1.10 1.14 3.10 6.13 3.08
8.0 7.5 7.0 6.5 6.0 5.5 5.0 4.5 4.0 3.5 3.0 2.5 2.0 1.5 1.0 0.5 0.0
f1 (ppm)

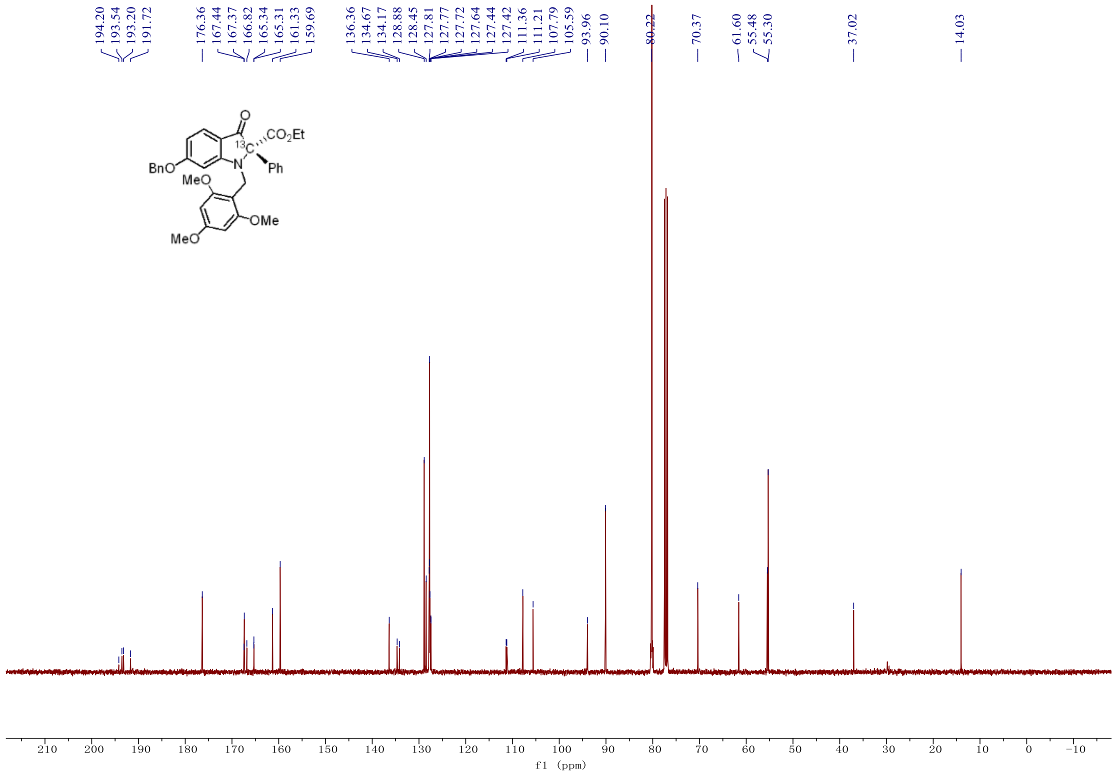
194.20 193.54 193.20 191.72 176.36 167.44 167.37 166.82 165.34 165.31 161.33 159.69 136.36 134.67 134.17 128.88 128.45 127.81 127.77 127.72 127.64 127.44 127.42 111.36 111.21 107.79 105.59 93.96 90.10 80.22 70.37 61.60 55.48 55.30 37.02 14.03
CO2Et
13C
Ph
BnO
N
MeO
OMe
MeO
210 200 190 180 170 160 150 140 130 120 110 100 90 80 70 60 50 40 30 20 10 0 -10
f1 (ppm)

6

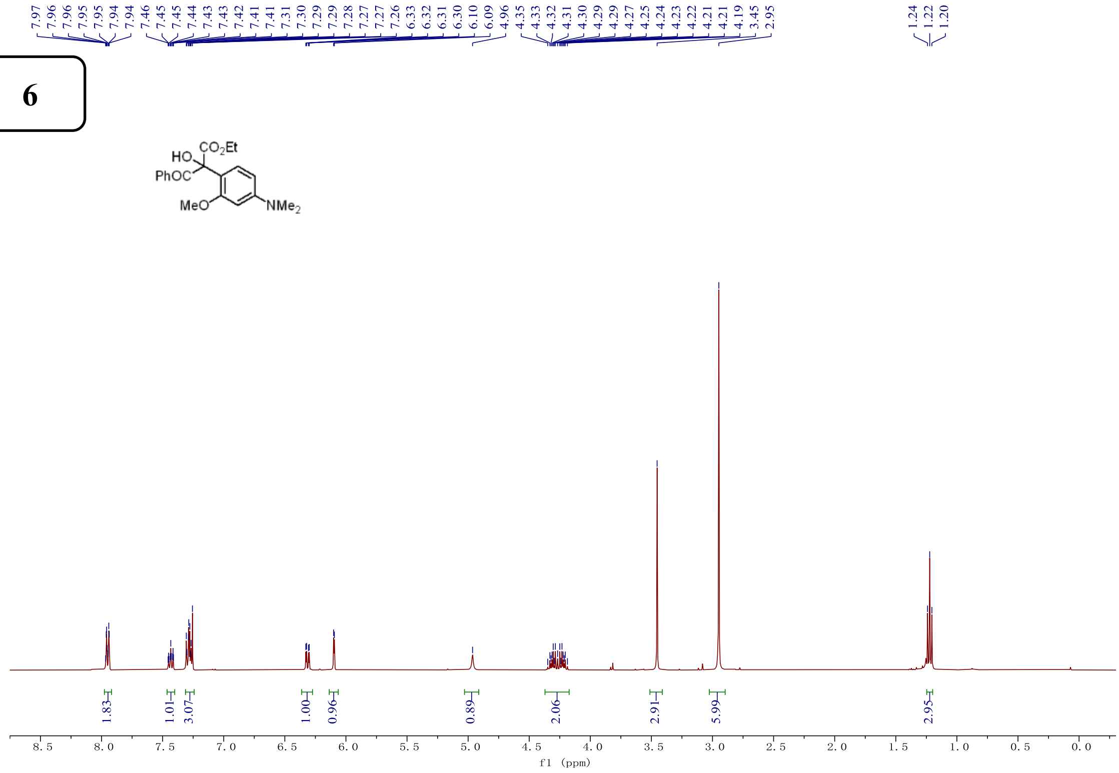
7.97 7.96 7.96 7.95 7.95 7.94 7.94 7.46 7.45 7.45 7.44 7.43 7.43 7.42 7.41 7.41 7.31 7.30 7.29 7.29 7.28 7.27 7.27 7.26 6.33 6.32 6.31 6.30 6.10 6.09 4.96 4.35 4.33 4.32 4.31 4.30 4.29 4.29 4.27 4.25 4.24 4.23 4.22 4.21 4.21 4.19 3.45 2.95
1.24 1.22 1.20
HO
CO2Et
PhOC
MeO
NMe2
1.83 1.01 3.07 1.00 0.96 0.89 2.06 2.91 5.99 2.95
8.5 8.0 7.5 7.0 6.5 6.0 5.5 5.0 4.5 4.0 3.5 3.0 2.5 2.0 1.5 1.0 0.5 0.0
f1 (ppm)

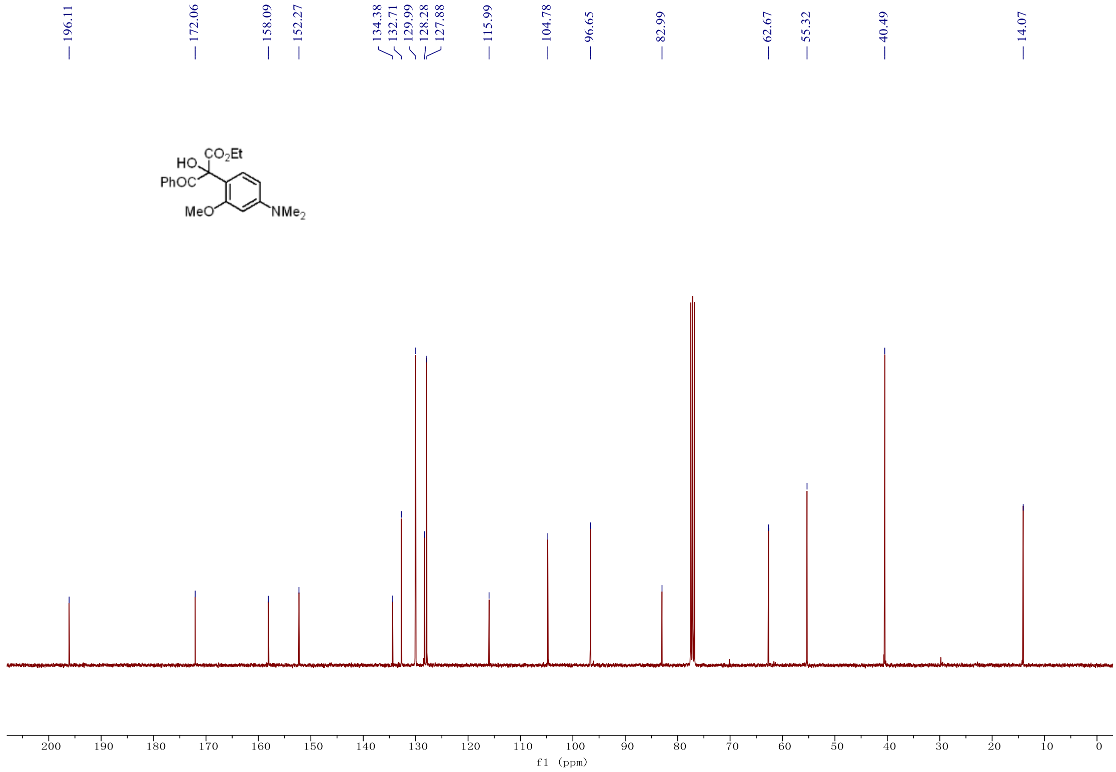
196.11
172.06
158.09
152.27
134.38 132.71 129.99 128.28 127.88
115.99
104.78
96.65
82.99
62.67
55.32
40.49
14.07
HO
CO2Et
PhOC
MeO
NMe2
200 190 180 170 160 150 140 130 120 110 100 90 80 70 60 50 40 30 20 10 0
f1 (ppm)

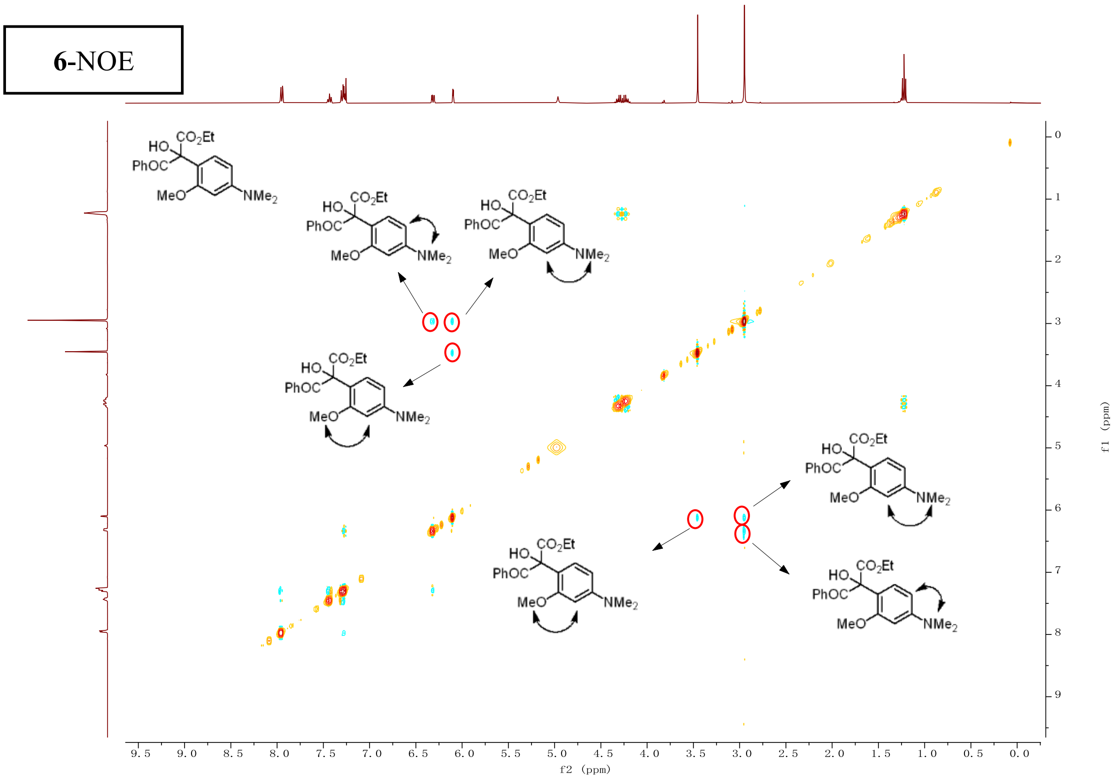
6-NOE
f2 (ppm)
f1 (ppm)

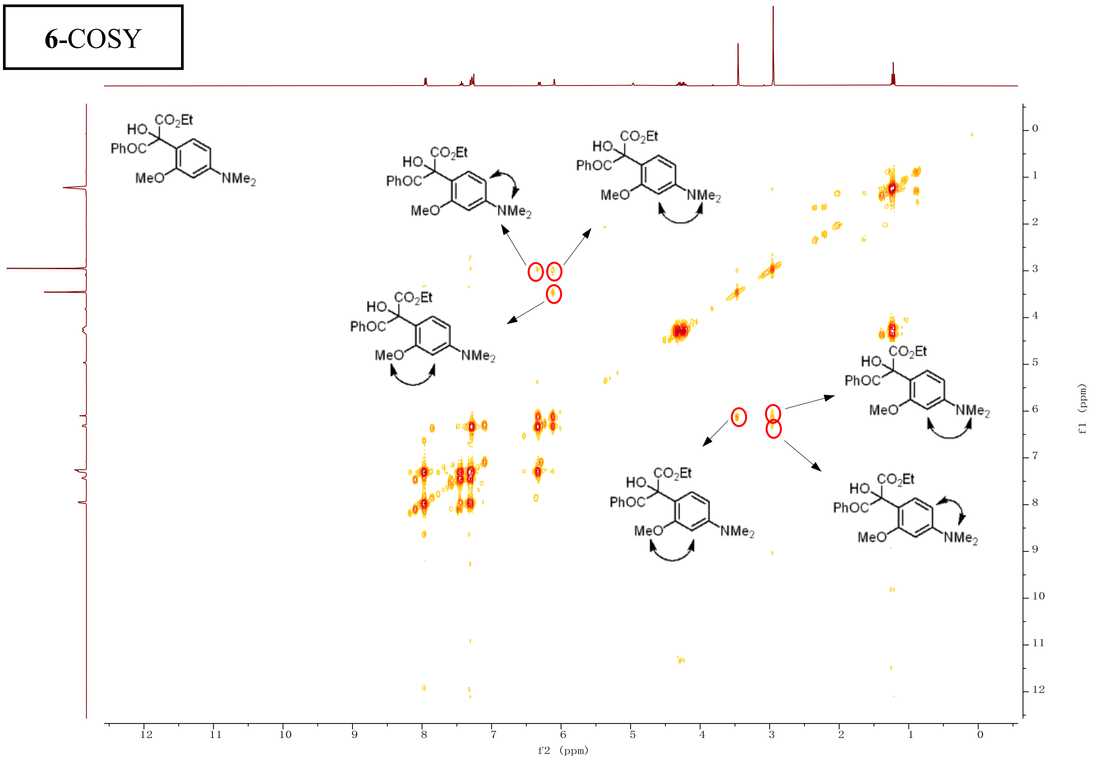
6-COSY
f2 (ppm)
f1 (ppm)

6-HMBC

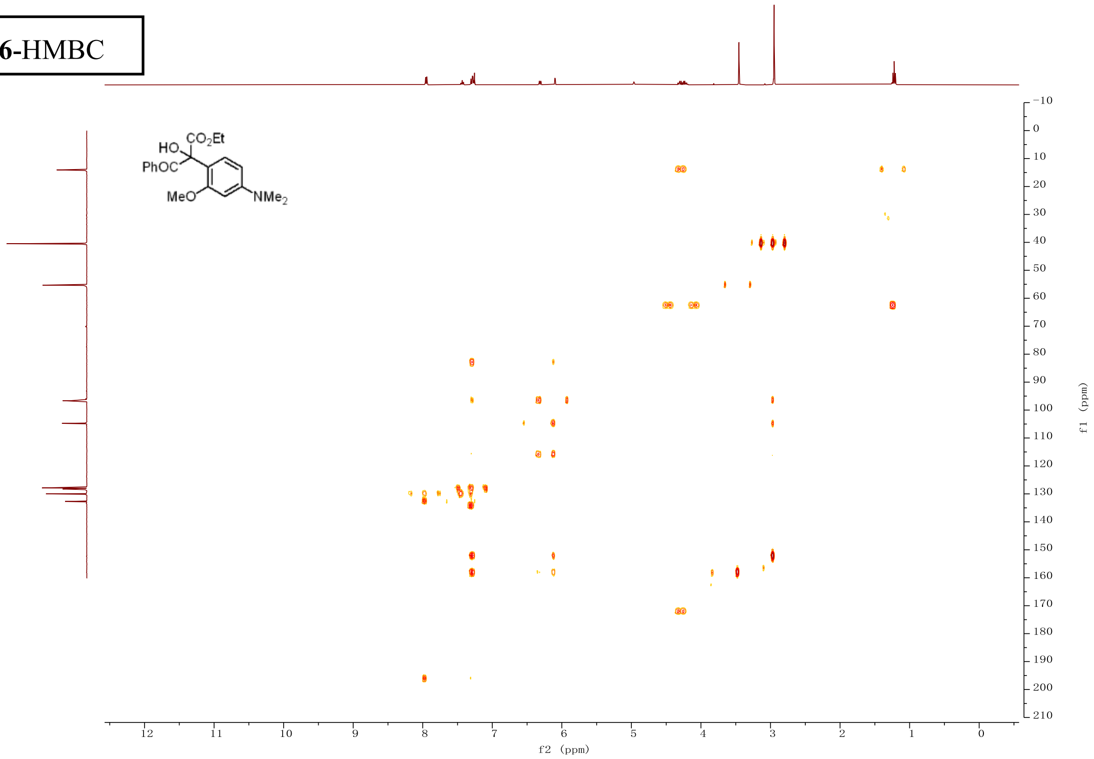

HO
CO2Et
PhOC
MeO
NMe2
f2 (ppm)
f1 (ppm)

6-HSQC

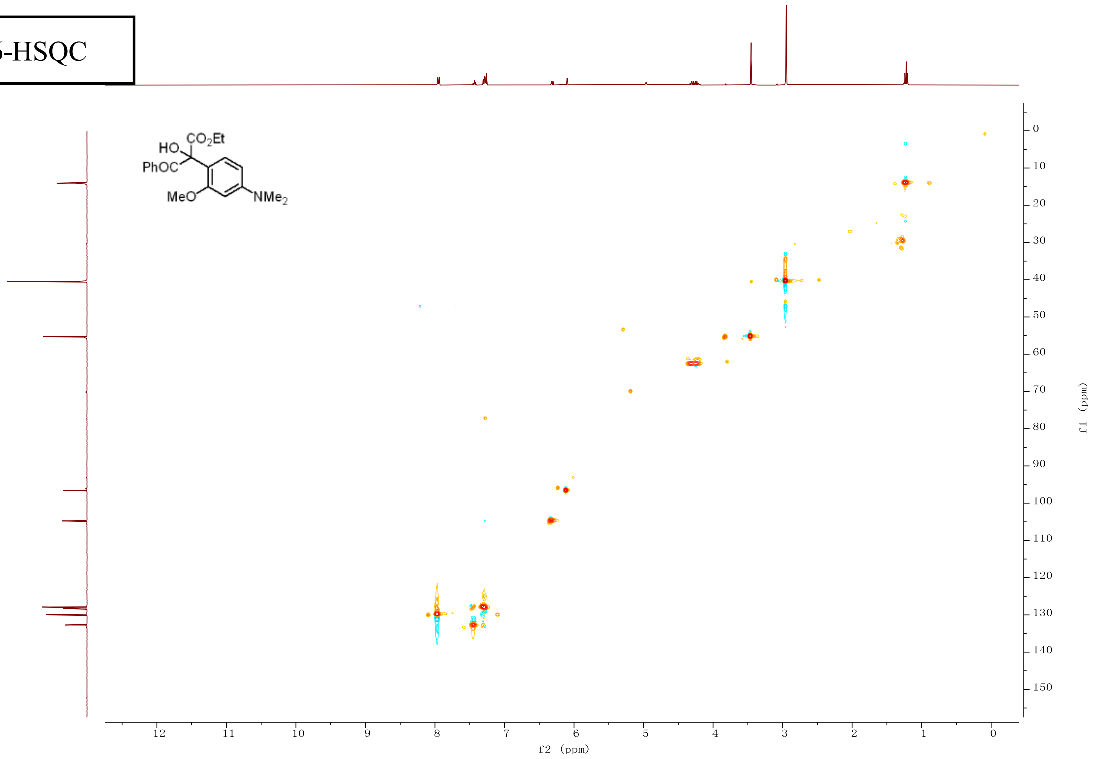

HO
CO2Et
PhOC
MeO
NMe2
f2 (ppm)
f1 (ppm)

6-DEPT135

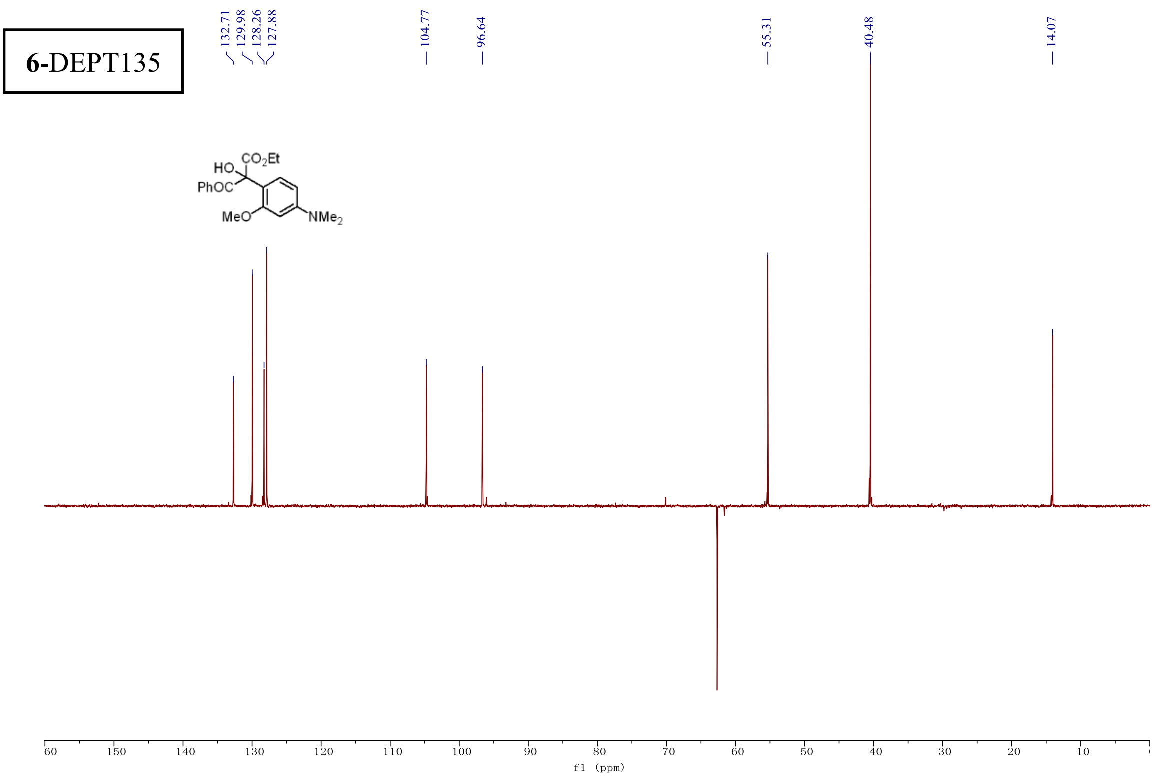

132.71
129.98
128.26
127.88
104.77
96.64
55.31
40.48
14.07
HO
CO2Et
PhOC
MeO
NMe2
160
150
140
130
120
110
100
90
80
70
60
50
40
30
20
10
f1 (ppm)

3ay

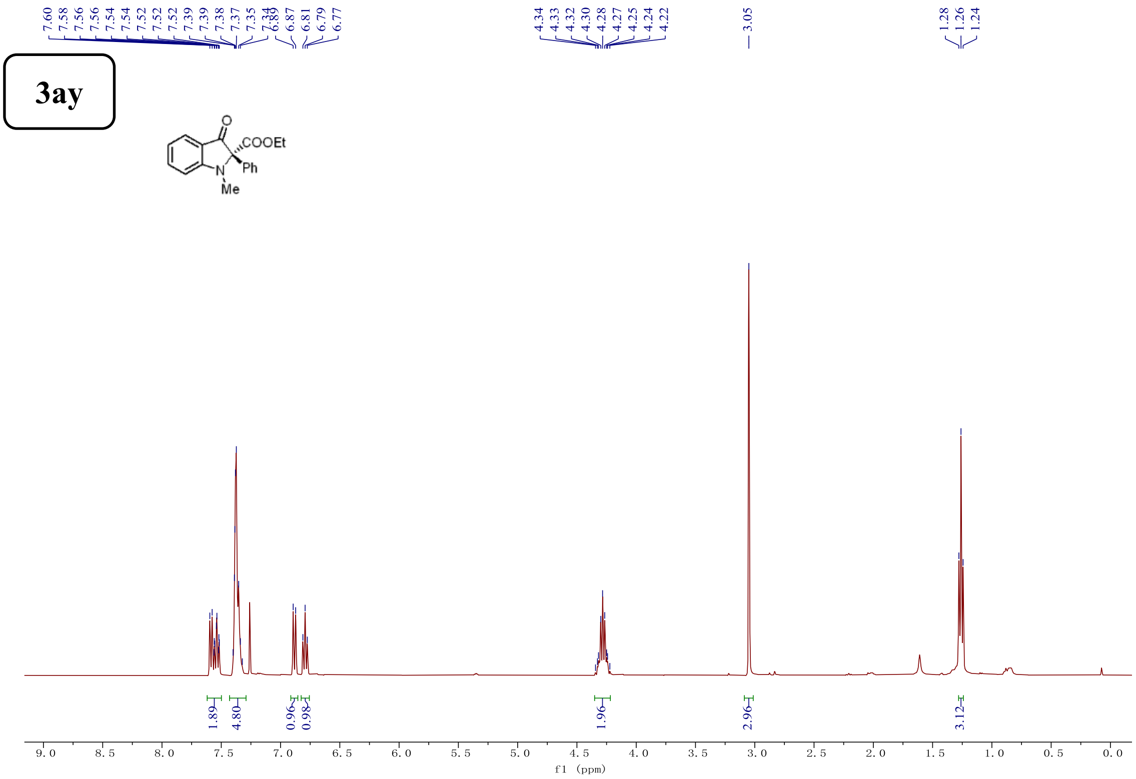
7.60 7.58 7.56 7.56 7.54 7.54 7.52 7.52 7.52 7.39 7.39 7.38 7.37 7.35 7.34 6.89 6.87 6.81 6.79 6.77
4.34 4.33 4.32 4.30 4.28 4.27 4.25 4.24 4.22
3.05
1.28 1.26 1.24
COOEt
Ph
N
Me
O
1.89 4.80 0.96 0.98 1.96 2.96 3.12
f1 (ppm)

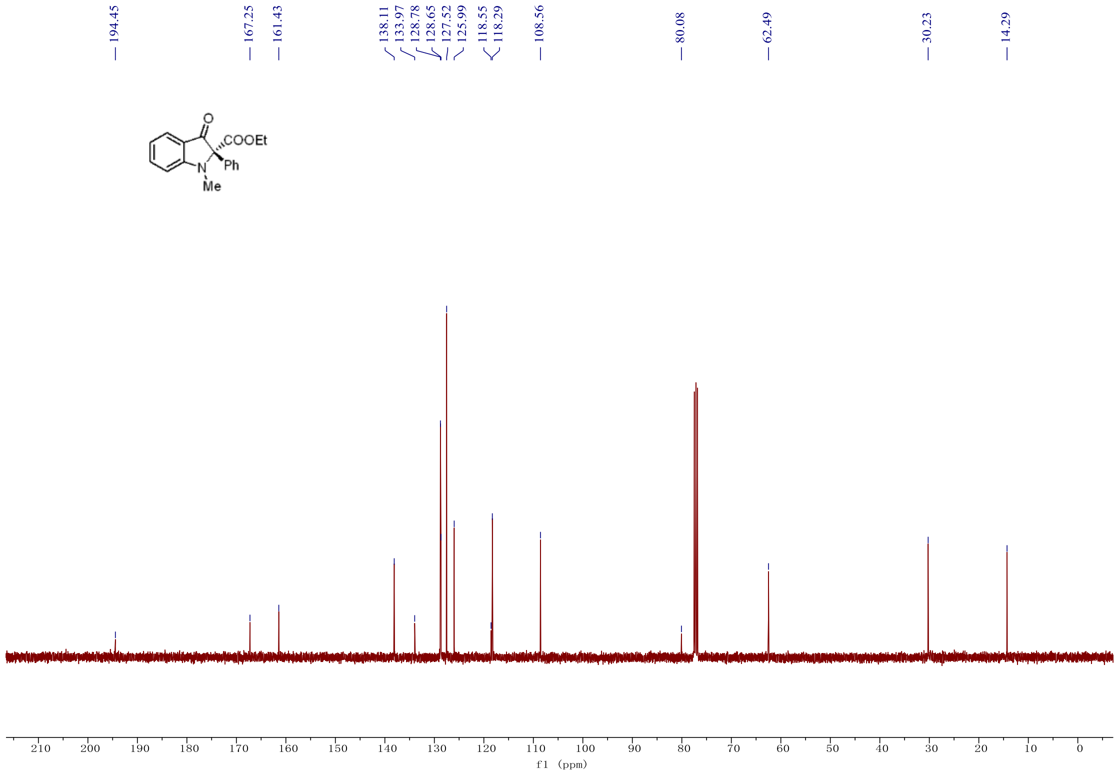
194.45
167.25
161.43
138.11 133.97 128.78 128.65 127.52 125.99 118.55 118.29
108.56
80.08
62.49
30.23
14.29
COOEt
Ph
N
Me
O
f1 (ppm)

8

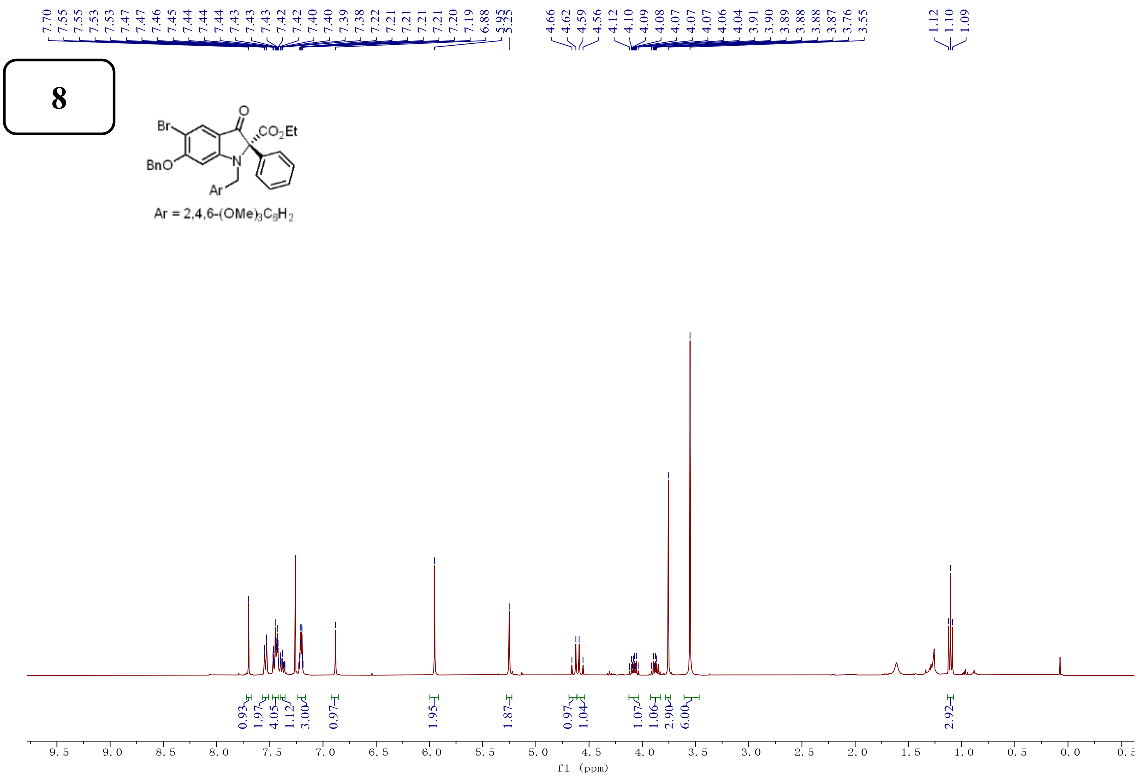
7.70 7.55 7.55 7.53 7.53 7.47 7.46 7.45 7.44 7.44 7.44 7.43 7.43 7.43 7.42 7.42 7.40 7.39 7.38 7.22 7.21 7.21 7.21 7.21 7.20 7.19 6.88 5.95 5.25
4.66 4.62 4.59 4.56 4.12 4.10 4.09 4.08 4.07 4.07 4.07 4.06 4.04 3.91 3.90 3.89 3.88 3.88 3.87 3.76 3.55
1.12 1.10 1.09
Br
O
CO2Et
BnO
N
Ar
Ar = 2,4,6-(OMe)3C6H2
0.93 1.97 4.05 1.12 3.00 0.97 1.95 1.87 0.97 1.04 1.07 1.06 2.90 6.00 2.92
9.5 9.0 8.5 8.0 7.5 7.0 6.5 6.0 5.5 5.0 4.5 4.0 3.5 3.0 2.5 2.0 1.5 1.0 0.5 0.0 -0.5
f1 (ppm)

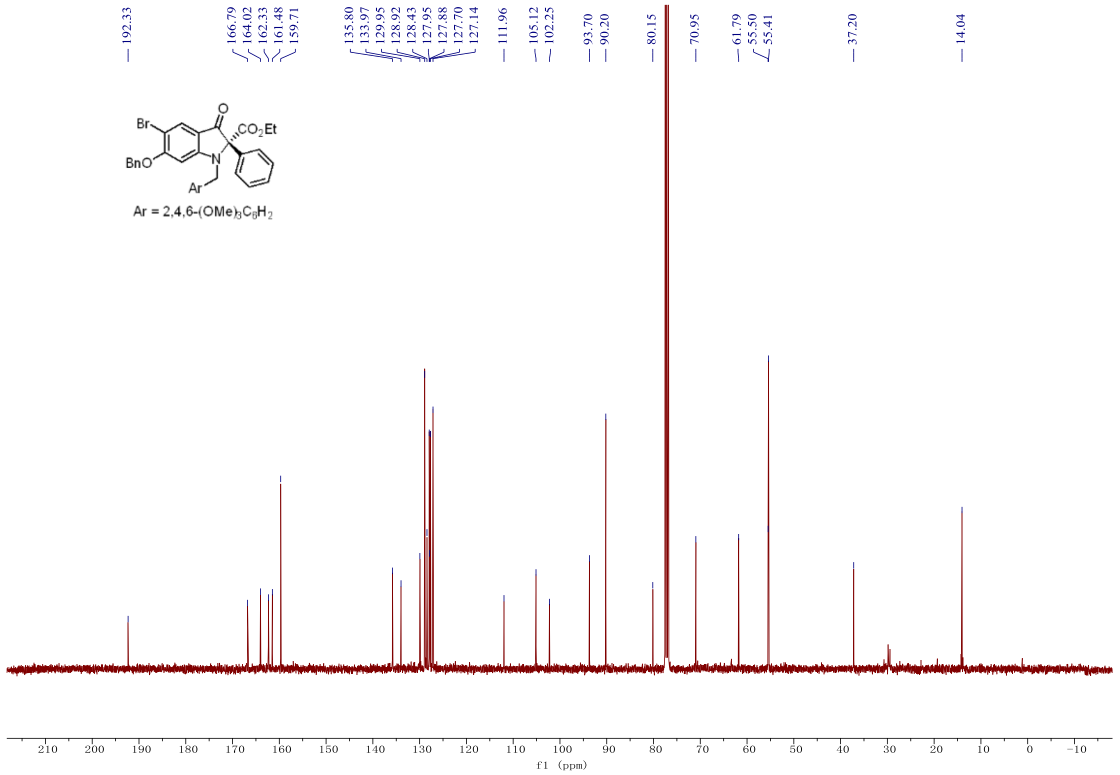
192.33
166.79 164.02 162.33 161.48 159.71
135.80 133.97 129.95 128.92 128.43 127.95 127.88 127.70 127.14
111.96
105.12 102.25
93.70 90.20
80.15
70.95
61.79 55.50 55.41
37.20
14.04
Br
O
CO2Et
BnO
N
Ar
Ar = 2,4,6-(OMe)3C6H2
210 200 190 180 170 160 150 140 130 120 110 100 90 80 70 60 50 40 30 20 10 0 -10
f1 (ppm)

9

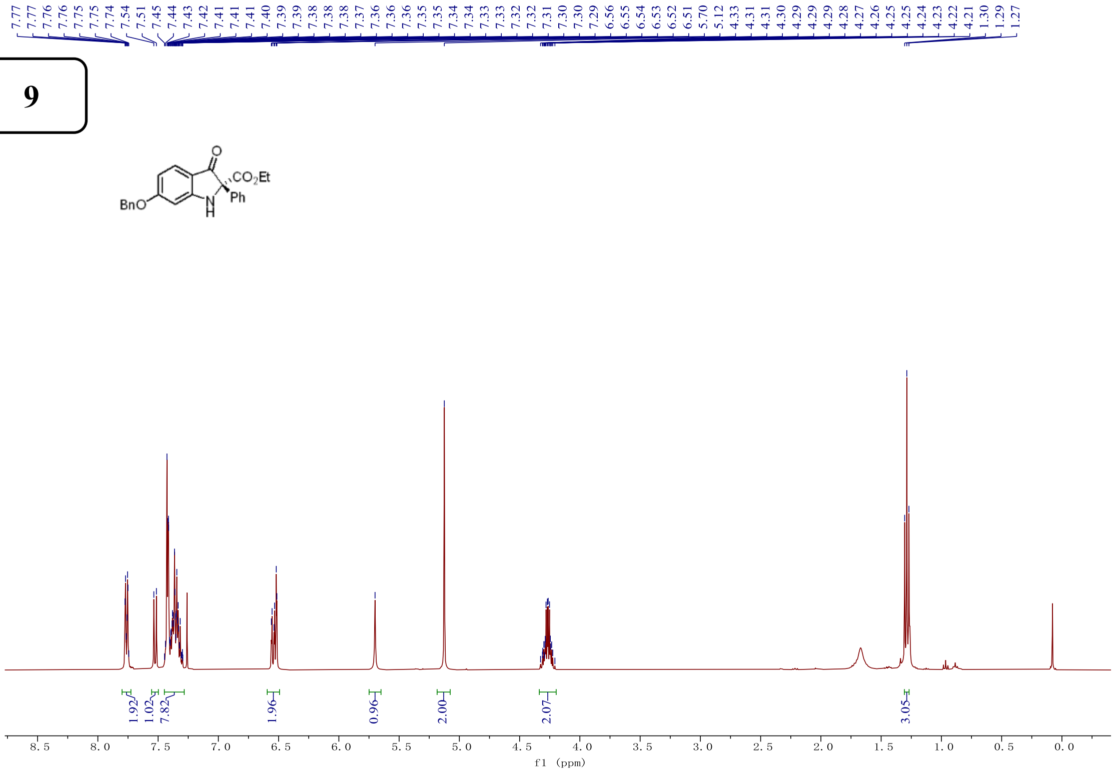
7.77 7.77 7.76 7.75 7.75 7.74 7.54 7.51 7.45 7.44 7.43 7.42 7.41 7.41 7.41 7.40 7.39 7.39 7.38 7.38 7.38 7.37 7.36 7.36 7.35 7.35 7.34 7.34 7.33 7.33 7.32 7.32 7.31 7.30 7.30 7.29 6.56 6.55 6.54 6.53 6.52 6.51 5.70 5.12 4.33 4.31 4.31 4.30 4.29 4.29 4.29 4.28 4.27 4.26 4.25 4.25 4.24 4.23 4.22 4.21 1.30 1.29 1.27
O
CO2Et
Ph
BnO
N
H
1.92
1.02
7.82
1.96
0.96
2.00
2.07
3.05
8.5 8.0 7.5 7.0 6.5 6.0 5.5 5.0 4.5 4.0 3.5 3.0 2.5 2.0 1.5 1.0 0.5 0.0
f1 (ppm)

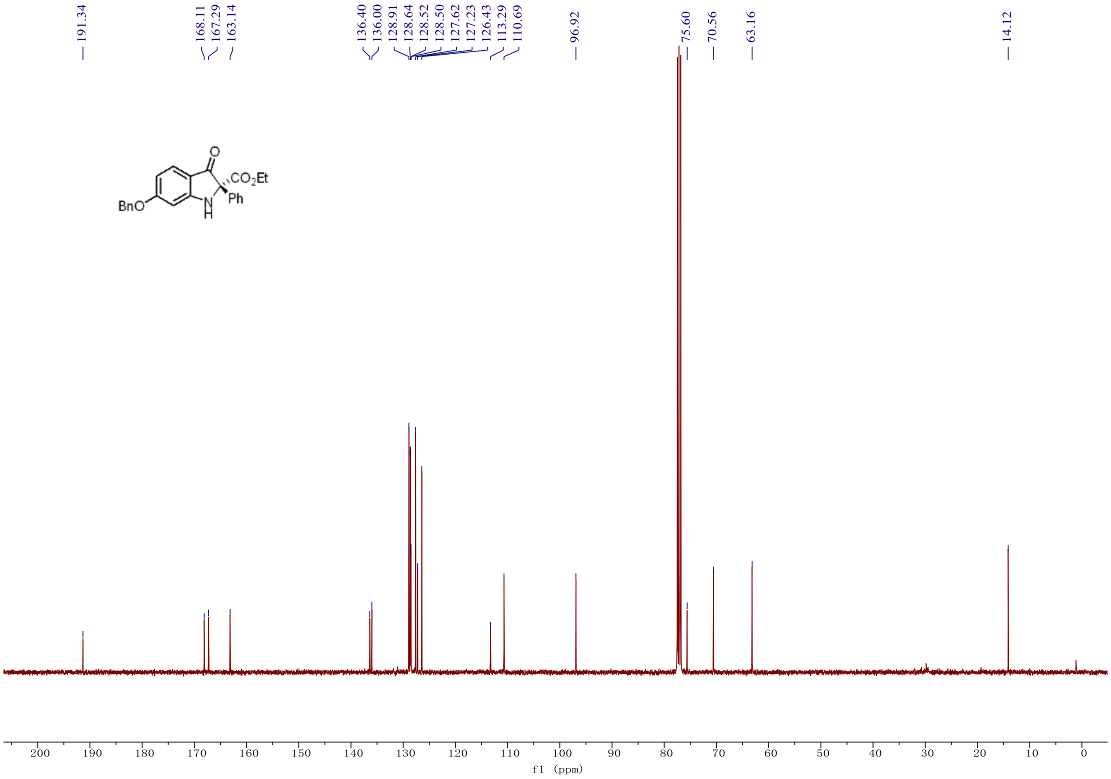
191.34
168.11 167.29 163.14
136.40 136.00 128.91 128.64 128.52 128.50 127.62 127.23 126.43 113.29 110.69
96.92
75.60
70.56
63.16
14.12
O
CO2Et
Ph
BnO
N
H
200 190 180 170 160 150 140 130 120 110 100 90 80 70 60 50 40 30 20 10 0
f1 (ppm)

10

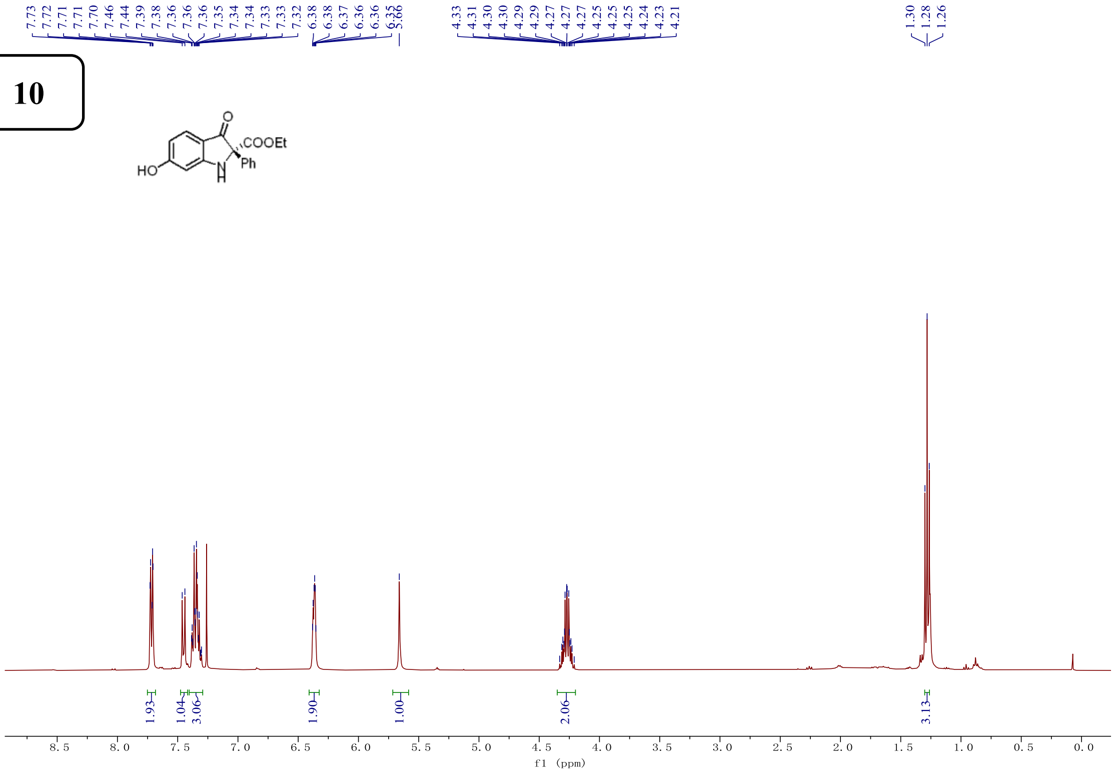
7.73
7.72
7.71
7.71
7.70
7.46
7.44
7.39
7.38
7.36
7.36
7.36
7.35
7.34
7.34
7.33
7.33
7.32
6.38
6.38
6.37
6.36
6.36
6.35
5.66
4.33
4.31
4.30
4.30
4.29
4.29
4.27
4.27
4.27
4.25
4.25
4.25
4.24
4.23
4.21
1.30
1.28
1.26
COOEt
Ph
HO
N
H
O
1.93
1.04
3.06
1.90
1.00
2.06
3.13
f1 (ppm)

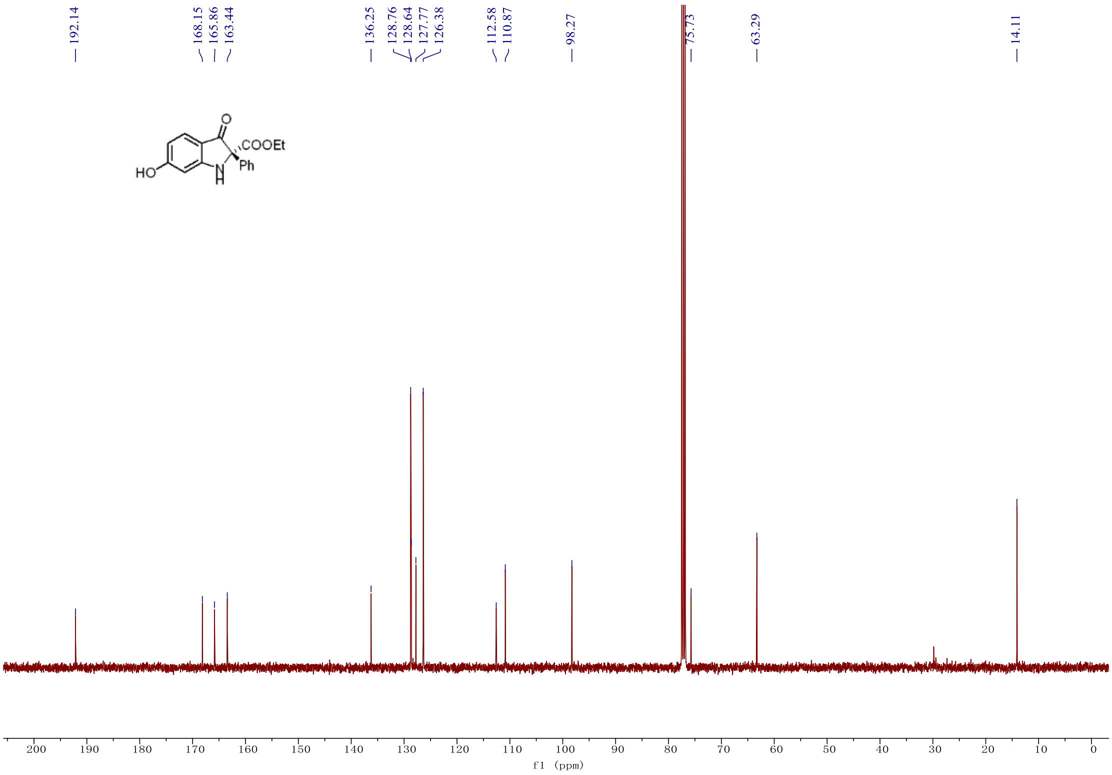
192.14
168.15
165.86
163.44
136.25
128.76
128.64
127.77
126.38
112.58
110.87
98.27
75.73
63.29
14.11
COOEt
Ph
HO
N
H
O
f1 (ppm)

11

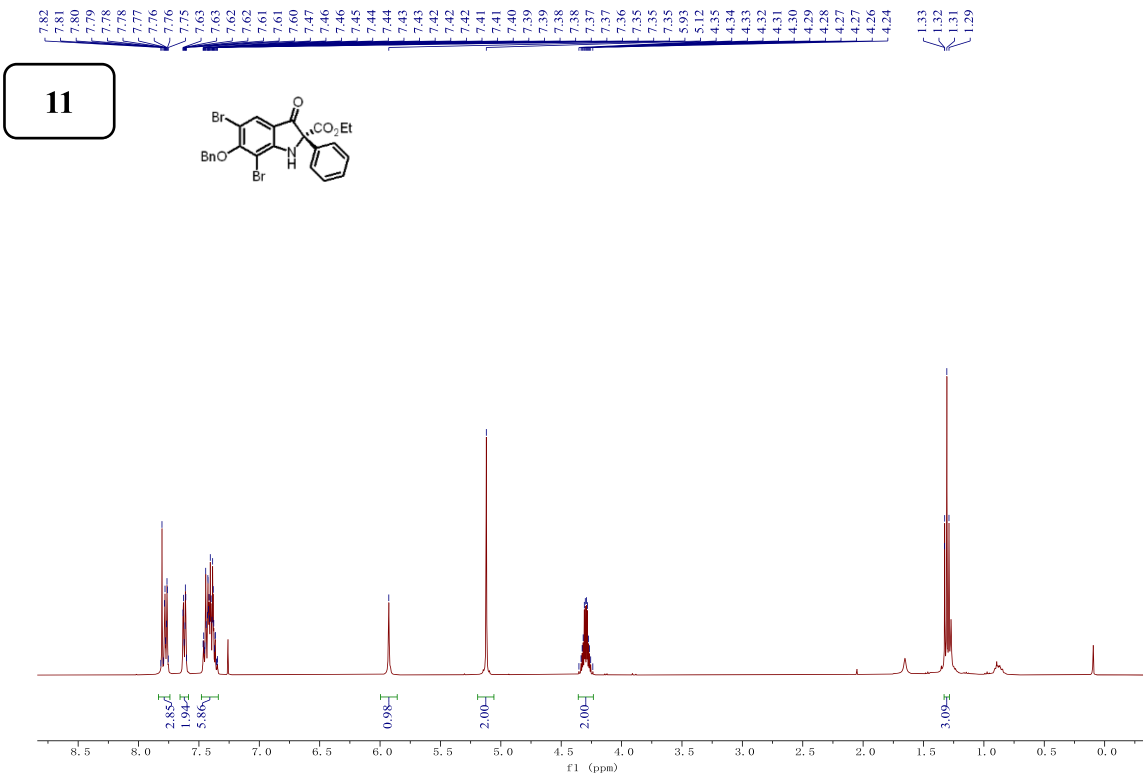

7.82 7.81 7.80 7.79 7.78 7.77 7.76 7.76 7.75 7.63 7.63 7.62 7.62 7.61 7.61 7.60 7.47 7.46 7.46 7.45 7.44 7.44 7.43 7.43 7.42 7.42 7.42 7.41 7.41 7.40 7.39 7.39 7.38 7.38 7.37 7.37 7.36 7.35 7.35 5.93 5.12 4.35 4.34 4.33 4.32 4.31 4.30 4.29 4.28 4.27 4.27 4.26 4.24
1.33 1.32 1.31 1.29
Br
BnO
Br
O
CO2Et
N
H
2.85
1.94
5.86
0.98
2.00
2.00
3.09
8.5 8.0 7.5 7.0 6.5 6.0 5.5 5.0 4.5 4.0 3.5 3.0 2.5 2.0 1.5 1.0 0.5 0.0
f1 (ppm)

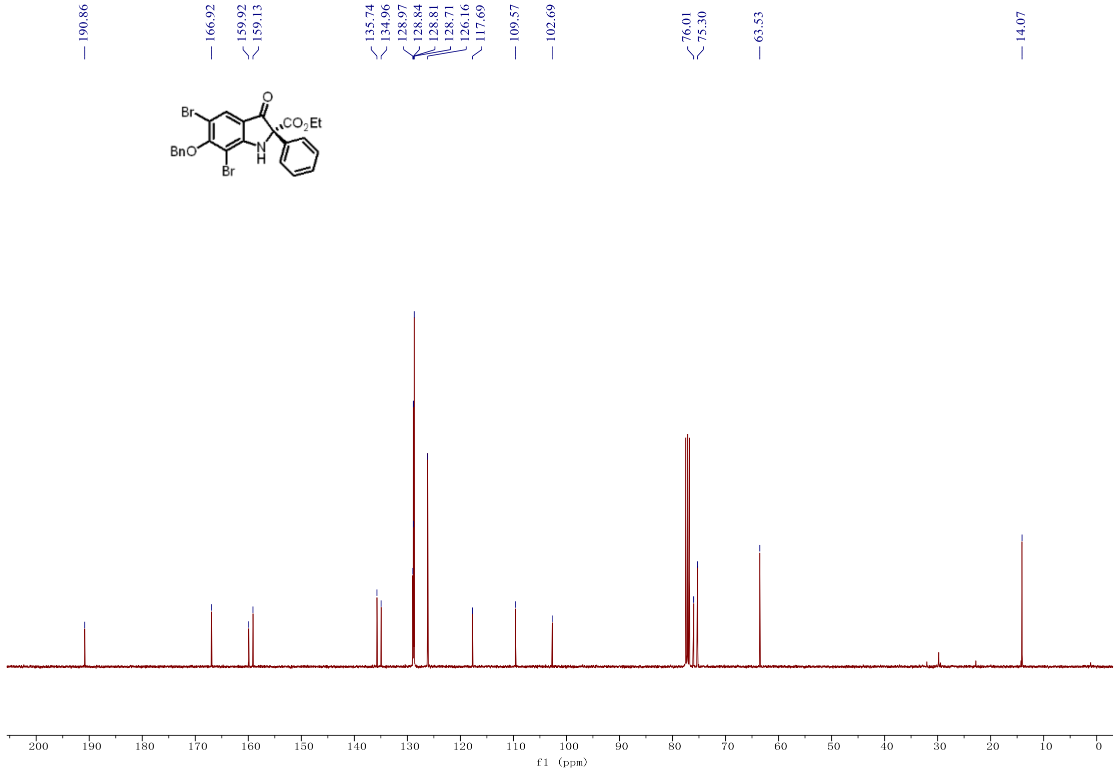

190.86
166.92
159.92
159.13
135.74
134.96
128.97
128.84
128.81
128.71
126.16
117.69
109.57
102.69
76.01
75.30
63.53
14.07
Br
BnO
Br
O
CO2Et
N
H
200 190 180 170 160 150 140 130 120 110 100 90 80 70 60 50 40 30 20 10 0
f1 (ppm)

12

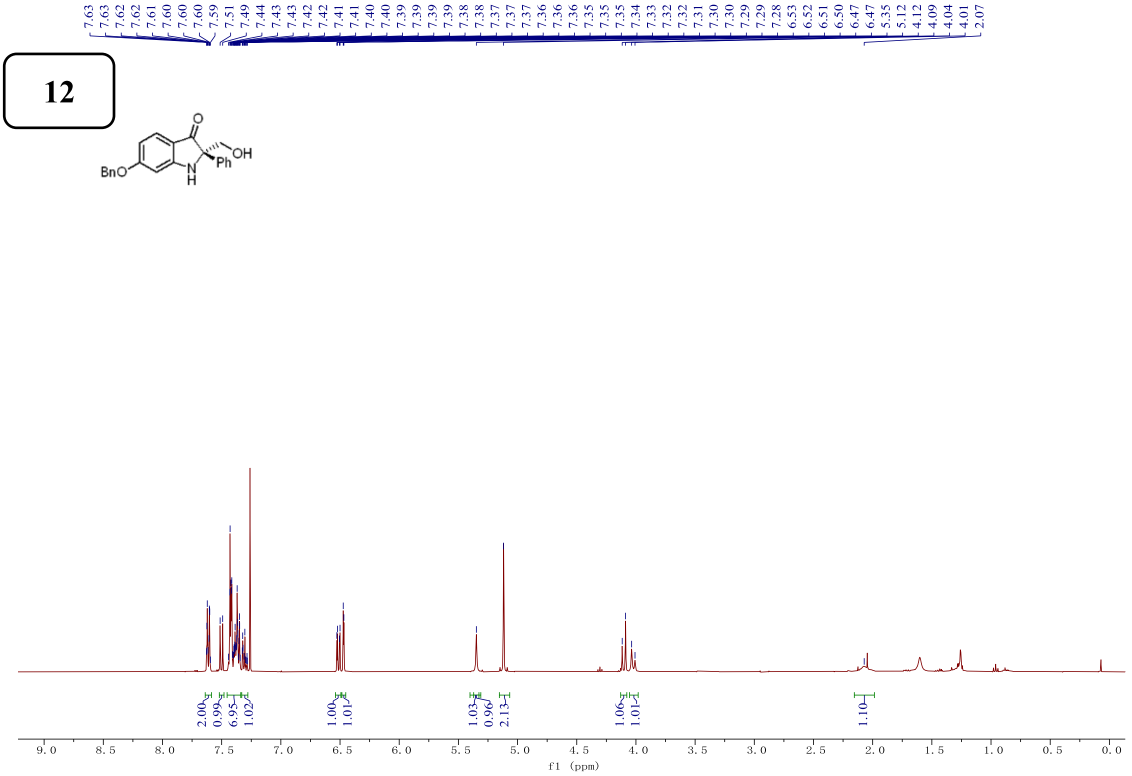

BnO
N
H
O
OH
Ph
7.63 7.63 7.62 7.62 7.61 7.60 7.60 7.60 7.59 7.51 7.49 7.44 7.43 7.43 7.42 7.41 7.41 7.41 7.40 7.40 7.39 7.39 7.39 7.39 7.38 7.38 7.37 7.37 7.37 7.36 7.36 7.36 7.35 7.35 7.35 7.34 7.33 7.32 7.32 7.31 7.30 7.30 7.29 7.29 7.28 6.53 6.52 6.51 6.50 6.47 6.47 5.35 5.12 4.12 4.09 4.04 4.01 2.07
2.00 0.99 6.95 1.02 1.00 1.01 1.03 0.96 2.13 1.06 1.01 1.10
9.0 8.5 8.0 7.5 7.0 6.5 6.0 5.5 5.0 4.5 4.0 3.5 3.0 2.5 2.0 1.5 1.0 0.5 0.0
f1 (ppm)

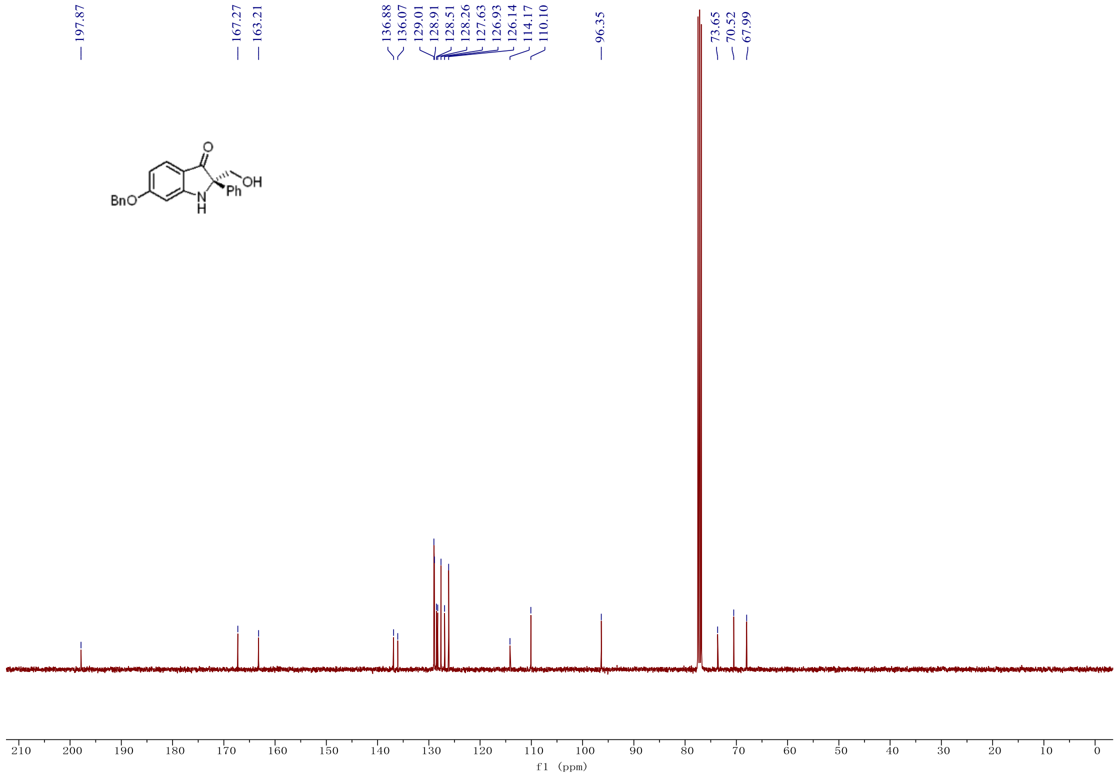

197.87
167.27
163.21
136.88
136.07
129.01
128.91
128.51
128.26
127.63
126.93
126.14
114.17
110.10
96.35
73.65
70.52
67.99
210 200 190 180 170 160 150 140 130 120 110 100 90 80 70 60 50 40 30 20 10 0
f1 (ppm)
BnO
N
H
O
OH
Ph

13

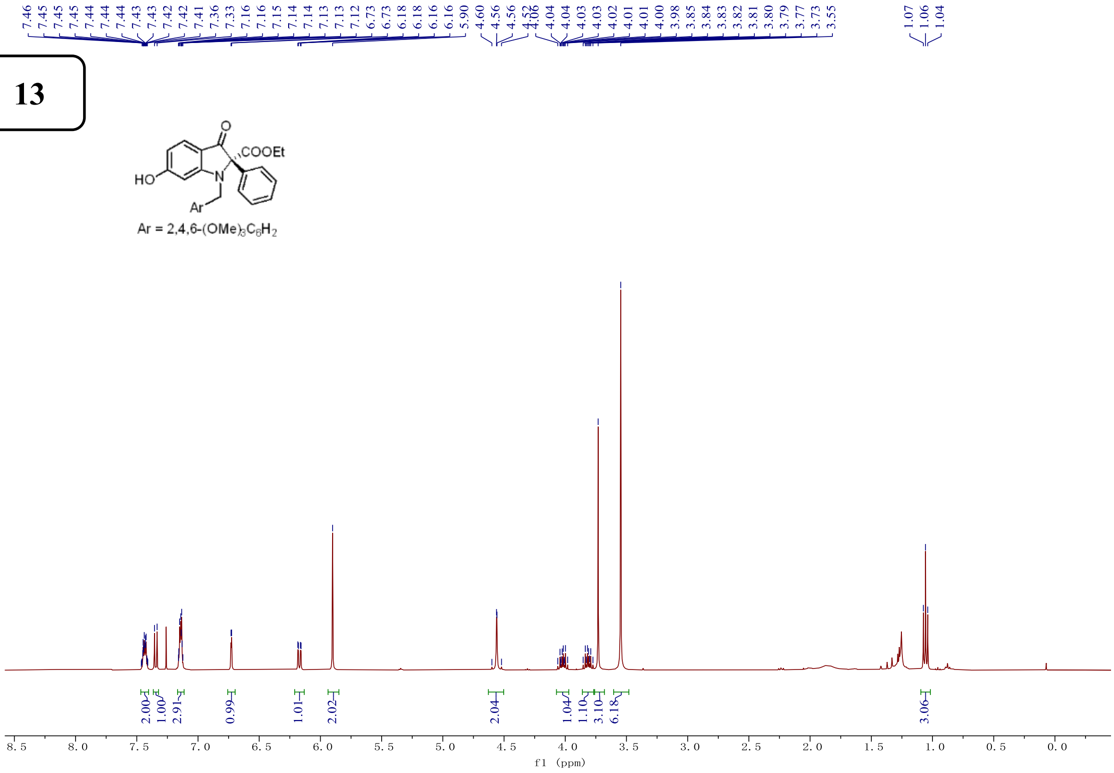
7.46 7.45 7.45 7.44 7.44 7.43 7.43 7.42 7.42 7.41 7.36 7.33 7.16 7.16 7.15 7.14 7.14 7.13 7.13 7.12 6.73 6.73 6.18 6.18 6.16 6.16 5.90 4.60 4.56 4.56 4.52 4.06 4.04 4.04 4.03 4.03 4.02 4.01 4.01 4.00 3.98 3.85 3.84 3.83 3.82 3.81 3.80 3.79 3.77 3.73 3.55 1.07 1.06 1.04
O
COOEt
HO
N
Ar
Ar = 2,4,6-(OMe)3C6H2
2.00 1.00 2.91 0.99 1.01 2.02 2.04 1.04 1.10 3.10 6.18 3.06
8.5 8.0 7.5 7.0 6.5 6.0 5.5 5.0 4.5 4.0 3.5 3.0 2.5 2.0 1.5 1.0 0.5 0.0
f1 (ppm)

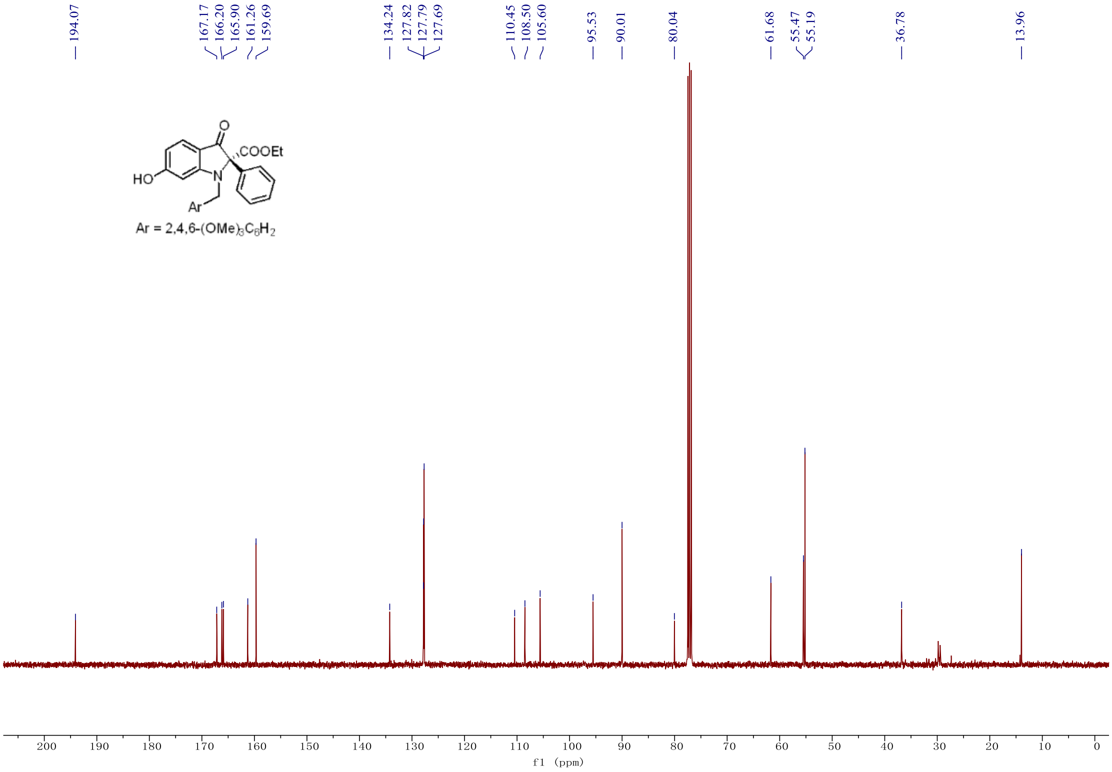
194.07
167.17 166.20 165.90 161.26 159.69
134.24 127.82 127.79 127.69
110.45 108.50 105.60
95.53
90.01
80.04
61.68 55.47 55.19
36.78
13.96
O
COOEt
HO
N
Ar
Ar = 2,4,6-(OMe)3C6H2
200 190 180 170 160 150 140 130 120 110 100 90 80 70 60 50 40 30 20 10 0
f1 (ppm)

14

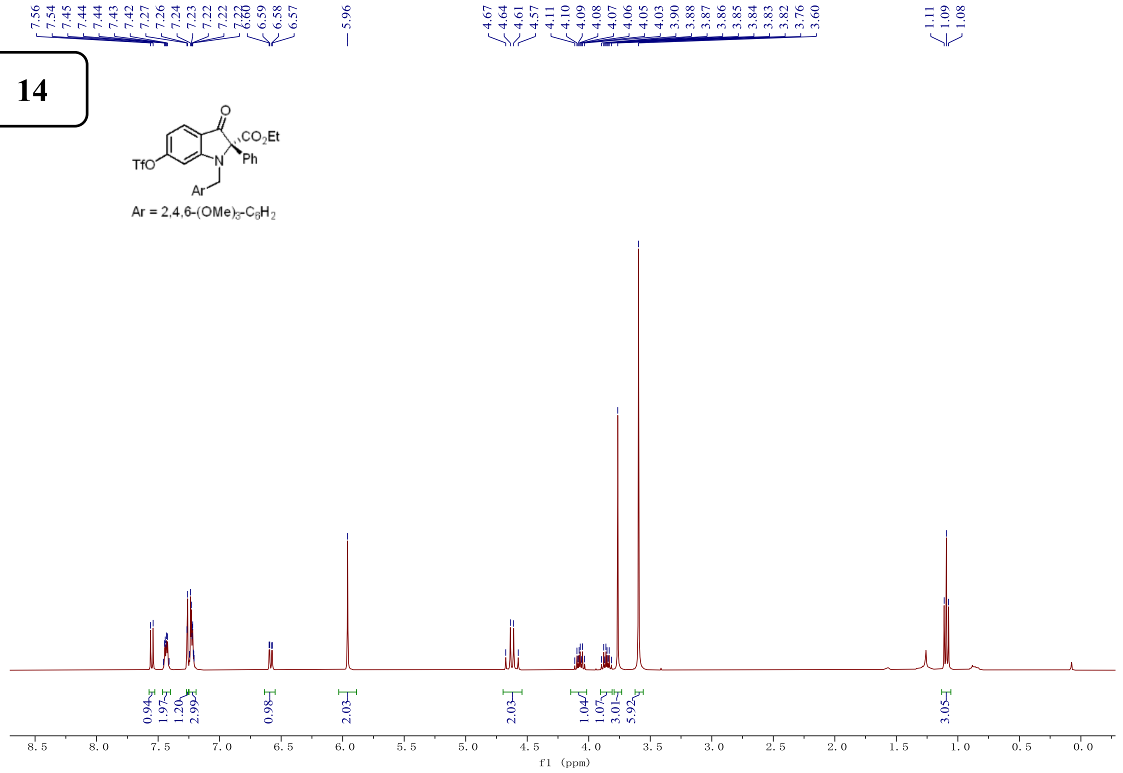

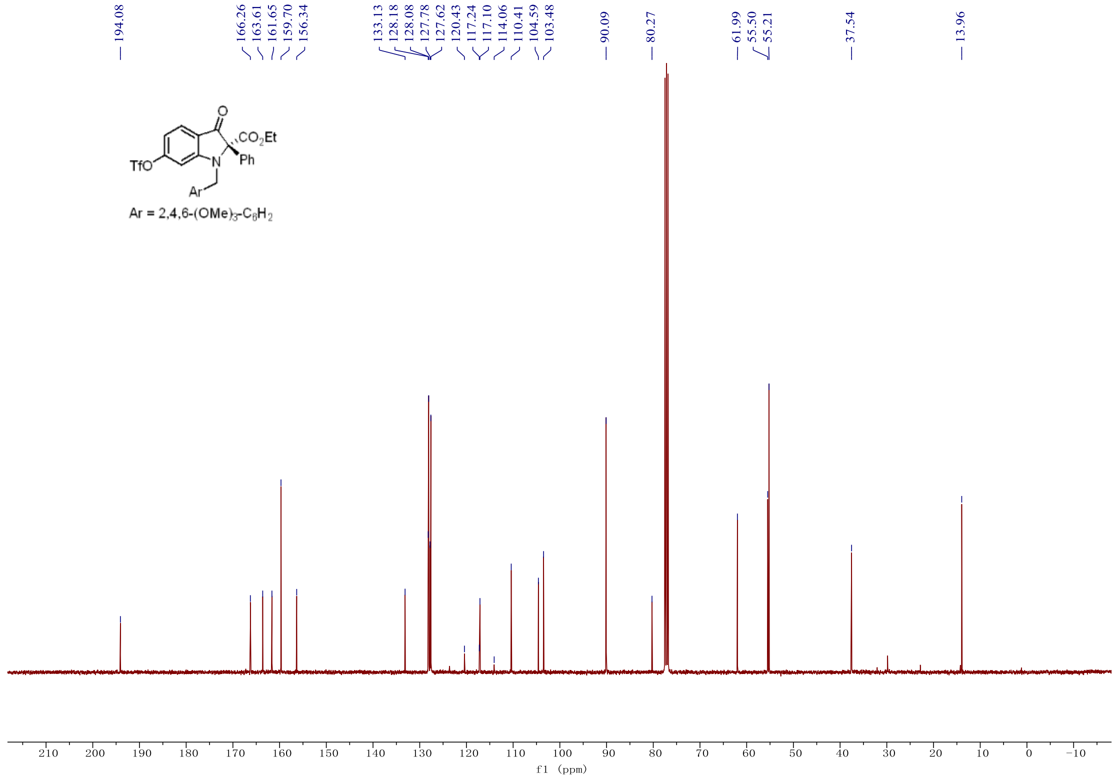

15

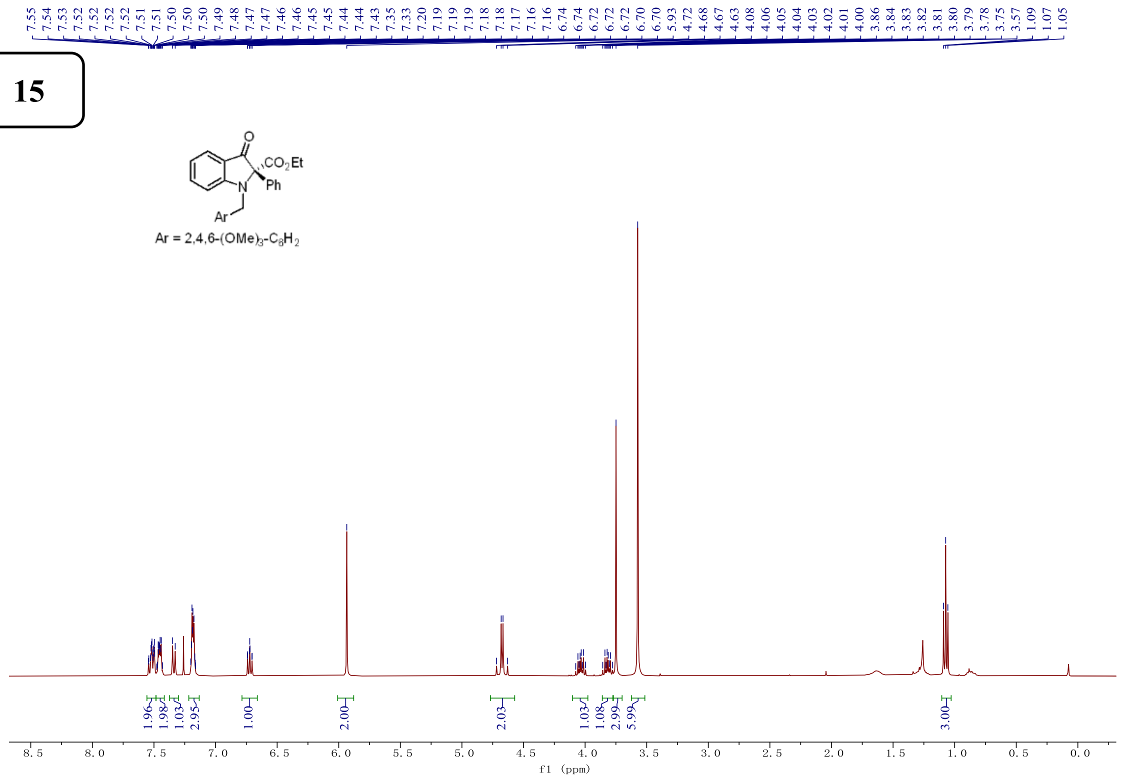

7.55 7.54 7.53 7.52 7.52 7.52 7.51 7.51 7.50 7.50 7.49 7.48 7.47 7.47 7.46 7.45 7.45 7.44 7.44 7.43 7.35 7.33 7.20 7.19 7.19 7.19 7.18 7.17 7.16 6.74 6.74 6.72 6.72 6.72 6.70 6.70 5.93 4.72 4.68 4.67 4.63 4.08 4.06 4.05 4.04 4.03 4.02 4.01 4.00 3.86 3.84 3.83 3.82 3.81 3.80 3.79 3.78 3.75 3.57 1.09 1.07 1.05
CO2Et
Ph
N
O
Ar
Ar = 2,4,6-(OMe)3-C6H2
1.96 1.98 1.03 2.95 1.00 2.00 2.03 1.03 1.08 2.99 5.99 3.00
f1 (ppm)

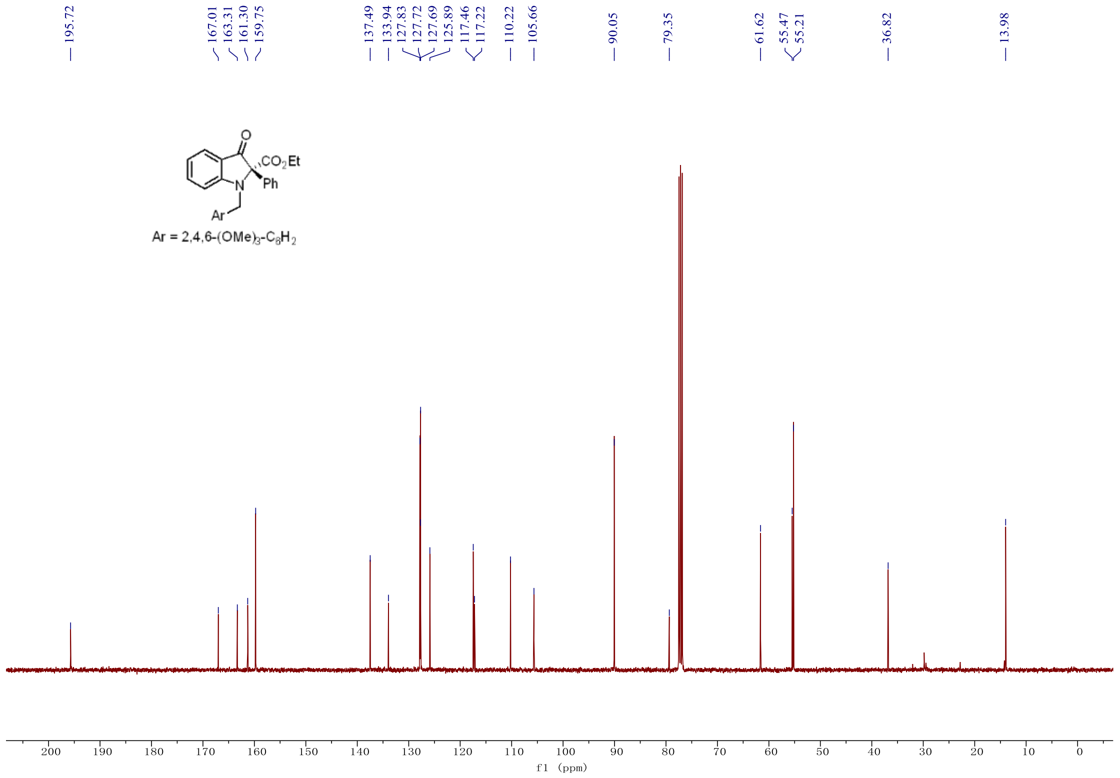

195.72 167.01 163.31 161.30 159.75 137.49 133.94 127.83 127.72 127.69 125.89 117.46 117.22 110.22 105.66 90.05 79.35 61.62 55.47 55.21 36.82 13.98
CO2Et
Ph
N
O
Ar
Ar = 2,4,6-(OMe)3-C6H2
f1 (ppm)

16

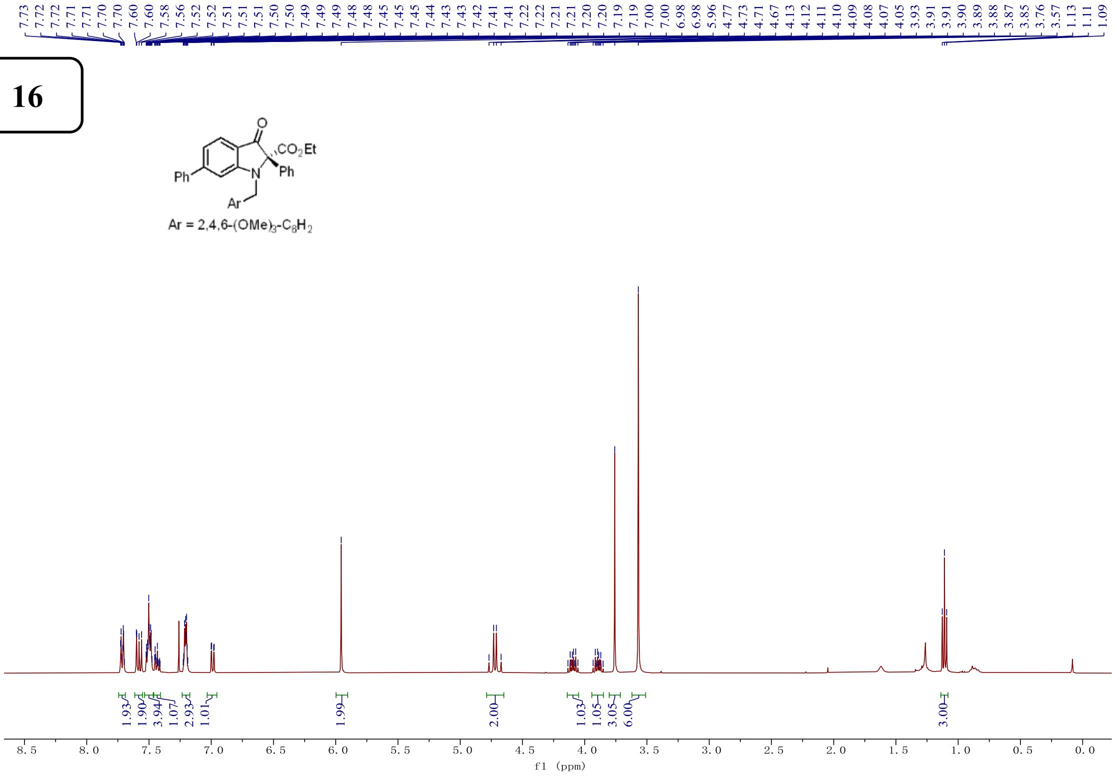
7.73 7.72 7.72 7.71 7.71 7.70 7.60 7.60 7.58 7.56 7.52 7.52 7.51 7.51 7.51 7.50 7.50 7.49 7.49 7.48 7.48 7.45 7.45 7.45 7.44 7.43 7.43 7.42 7.41 7.41 7.22 7.22 7.21 7.21 7.20 7.20 7.19 7.19 7.00 7.00 6.98 6.98 5.96 4.77 4.73 4.71 4.67 4.13 4.12 4.11 4.10 4.09 4.08 4.07 4.05 3.93 3.91 3.91 3.90 3.89 3.88 3.87 3.85 3.76 3.57 1.13 1.11 1.09
O
CO2Et
Ph
N
Ph
Ar
Ar = 2,4,6-(OMe)3-C6H2
1.93 1.90 3.94 1.07 2.93 1.01 1.99 2.00 1.03 1.05 3.05 6.00 3.00
8.5 8.0 7.5 7.0 6.5 6.0 5.5 5.0 4.5 4.0 3.5 3.0 2.5 2.0 1.5 1.0 0.5 0.0
f1 (ppm)

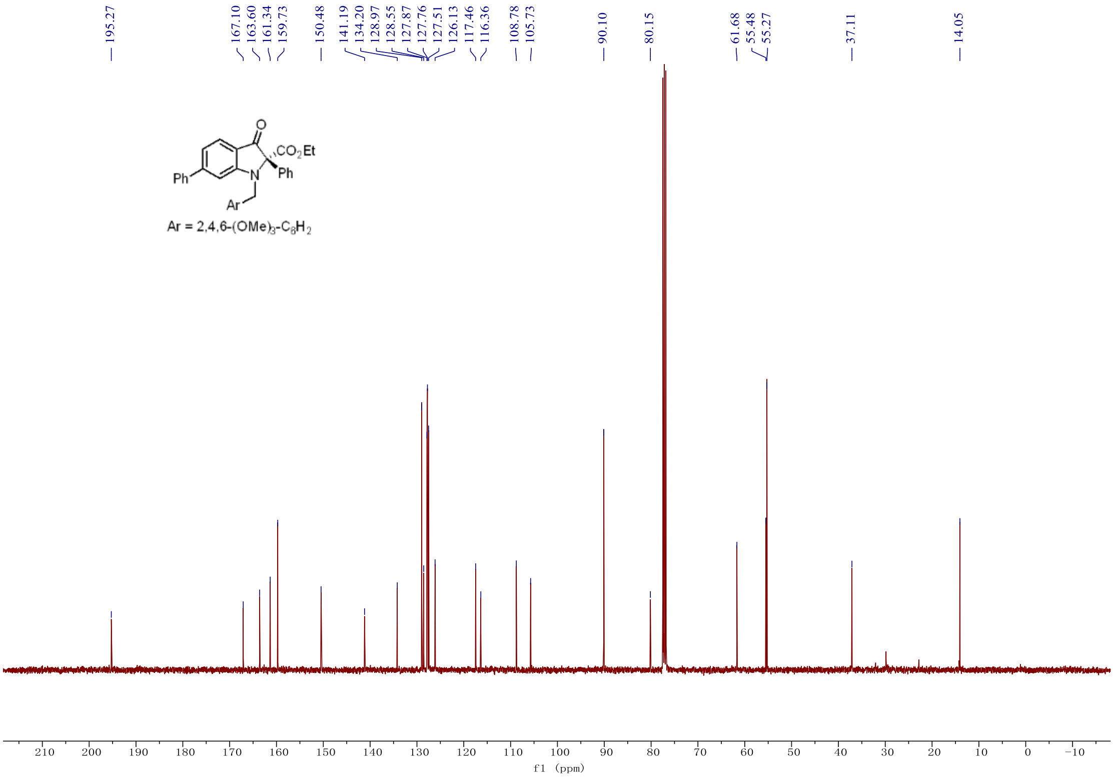
195.27 167.10 163.60 161.34 159.73 150.48 141.19 134.20 128.97 128.55 127.87 127.76 127.51 126.13 117.46 116.36 108.78 105.73 90.10 80.15 61.68 55.48 55.27 37.11 14.05
O
CO2Et
Ph
N
Ph
Ar
Ar = 2,4,6-(OMe)3-C6H2
210 200 190 180 170 160 150 140 130 120 110 100 90 80 70 60 50 40 30 20 10 0 -10
f1 (ppm)

11. HPLC chromatograms

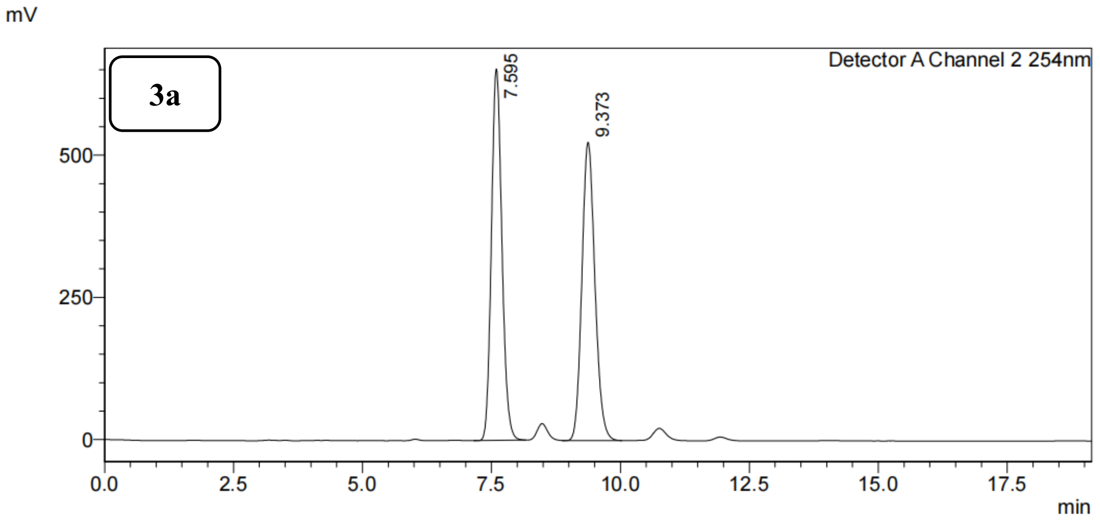

Detector A Channel 2 254nm

Peak#	Ret. Time	Area	Height	Conc.
1	7.595	9242303	652850	50.085
2	9.373	9210897	524227	49.915
Total		18453200	1177077	

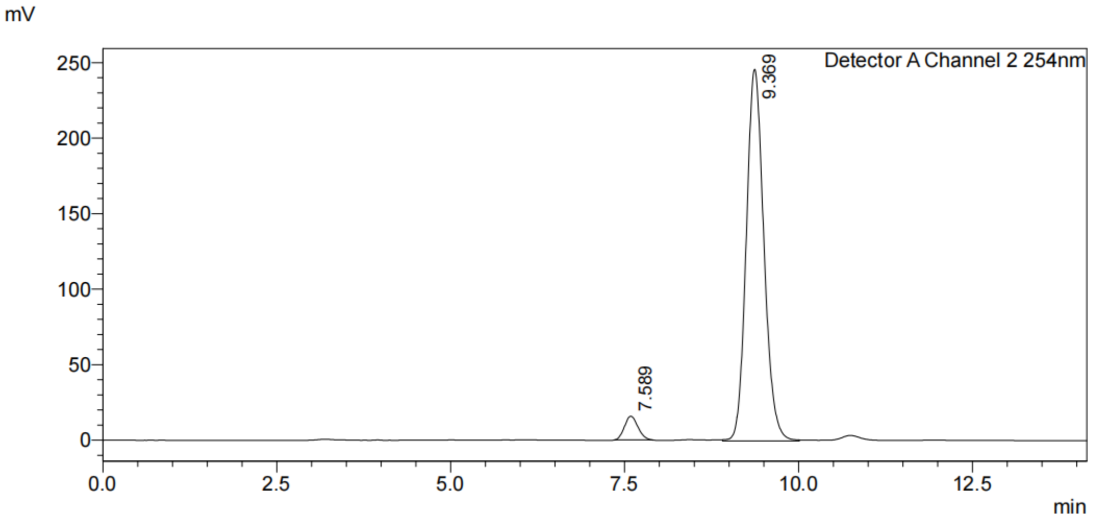

Detector A Channel 2 254nm

Peak#	Ret. Time	Area	Height	Conc.
1	7.589	210467	15680	4.620
2	9.369	4344953	246144	95.380
Total		4555420	261824	

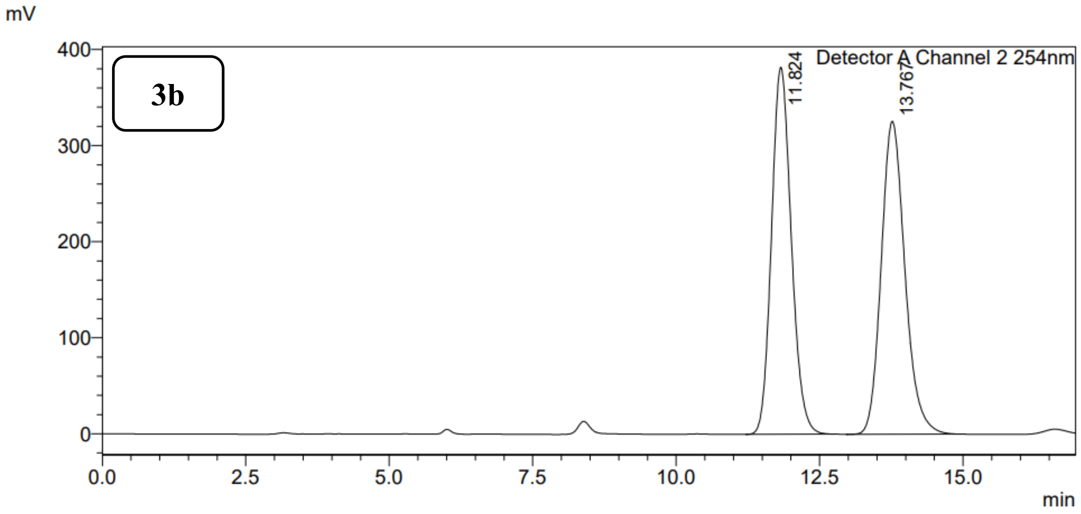

Detector A Channel 2 254nm

Peak#	Ret. Time	Area	Height	Conc.
1	11.824	9162685	382016	49.439
2	13.767	9370607	325682	50.561
Total		18533292	707698	

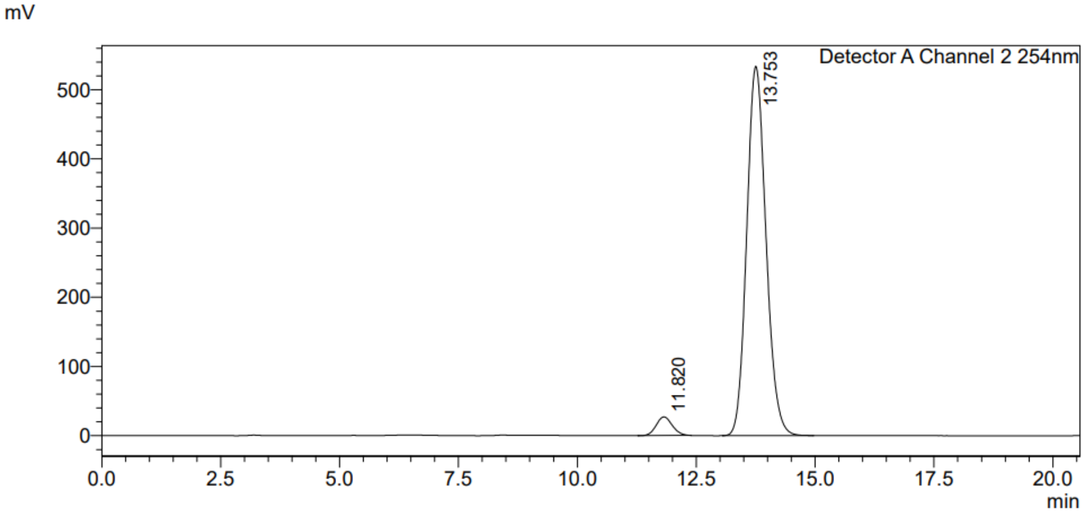

Detector A Channel 2 254nm

Peak#	Ret. Time	Area	Height	Conc.
1	11.820	633323	26950	4.038
2	13.753	15051553	533726	95.962
Total		15684876	560677	

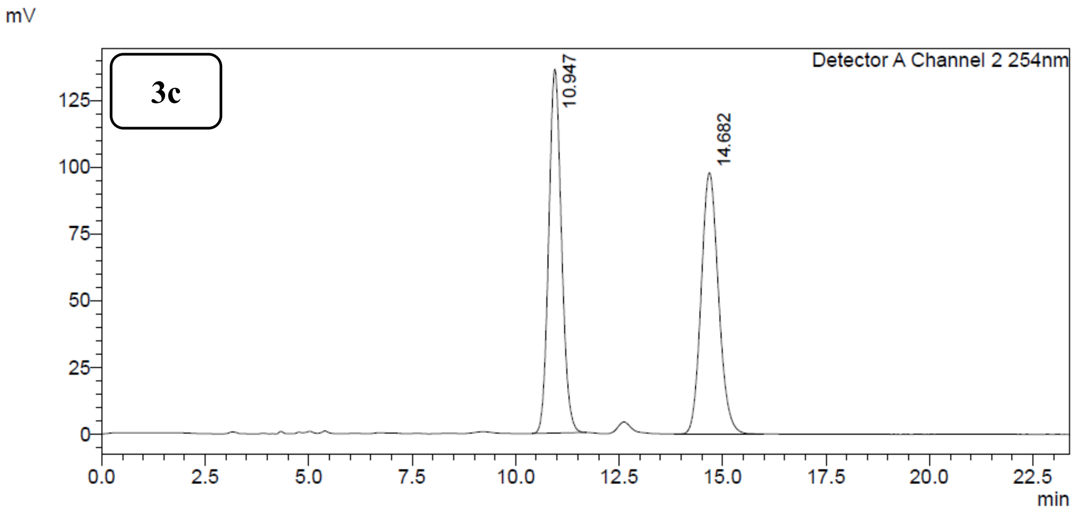

Detector A Channel 2 254nm

Peak#	Ret. Time	Area	Height	Conc.
1	10.947	2927754	136360	50.590
2	14.682	2859498	98029	49.410
Total		5787253	234389	

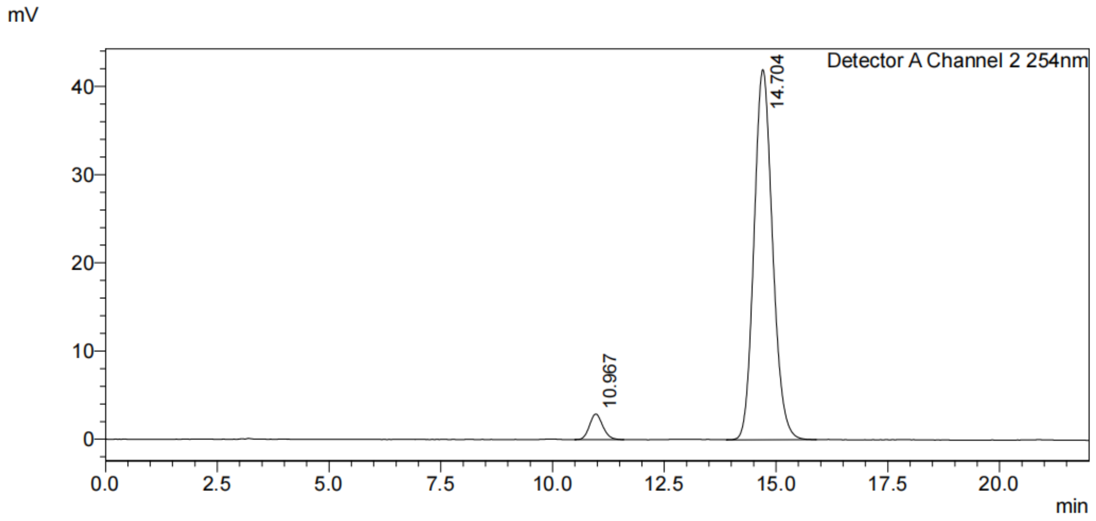

Detector A Channel 2 254nm

Peak#	Ret. Time	Area	Height	Conc.
1	10.967	59818	2893	4.707
2	14.704	1210926	41966	95.293
Total		1270744	44859	

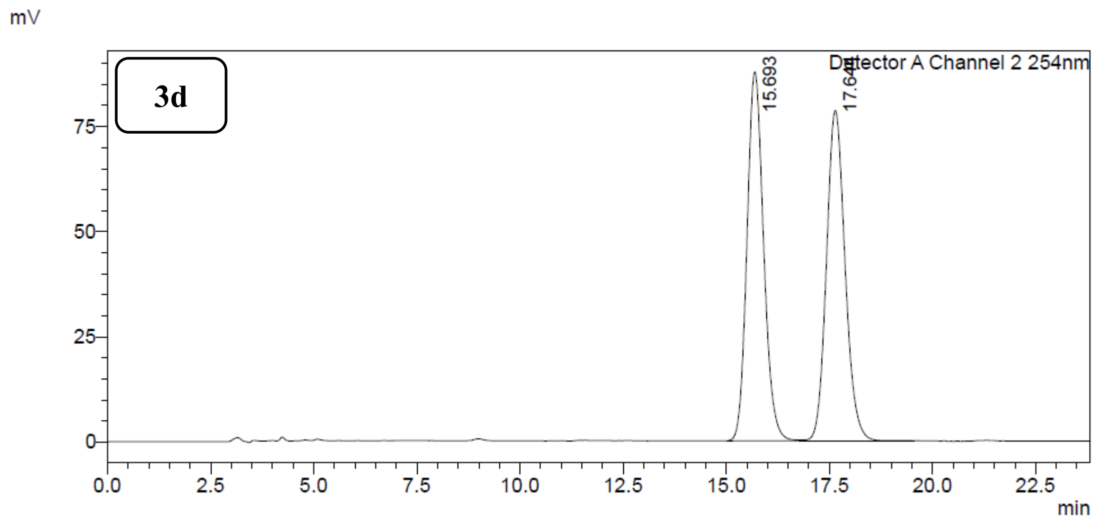

Detector A Channel 2 254nm

Peak#	Ret. Time	Area	Height	Conc.
1	15.693	2459767	87772	49.925
2	17.644	2467139	78554	50.075
Total		4926905	166326	

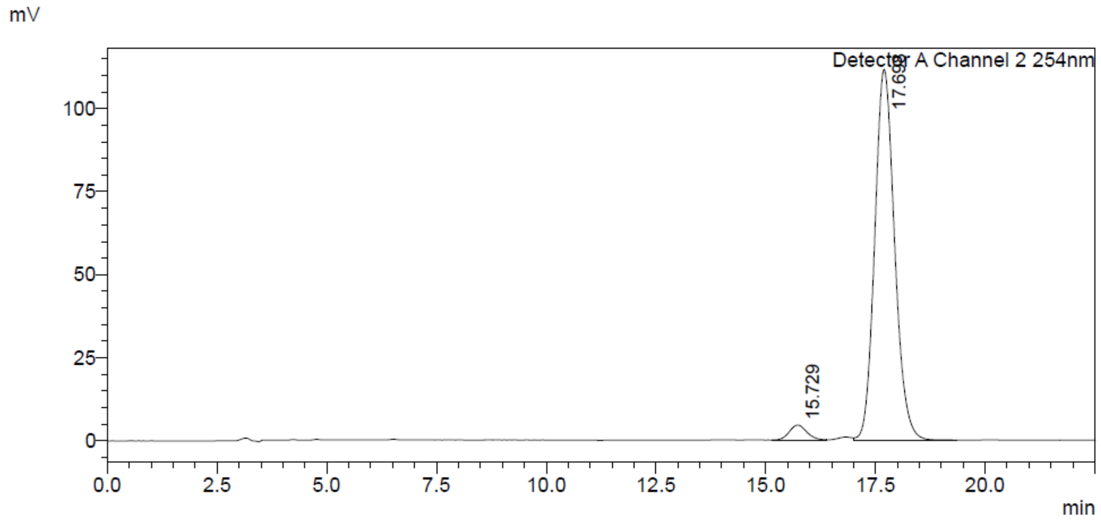

Detector A Channel 2 254nm

Peak#	Ret. Time	Area	Height	Conc.
1	15.729	123898	4515	3.408
2	17.698	3511841	111585	96.592
Total		3635739	116100	

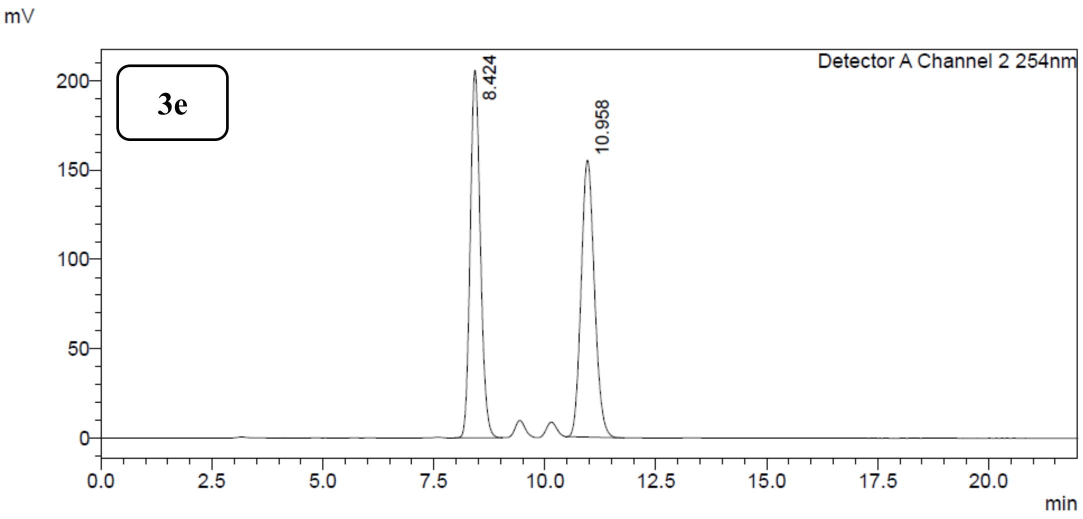

Detector A Channel 2 254nm

Peak#	Ret. Time	Area	Height	Conc.
1	8.424	3263456	205655	50.151
2	10.958	3243855	155111	49.849
Total		6507311	360766	

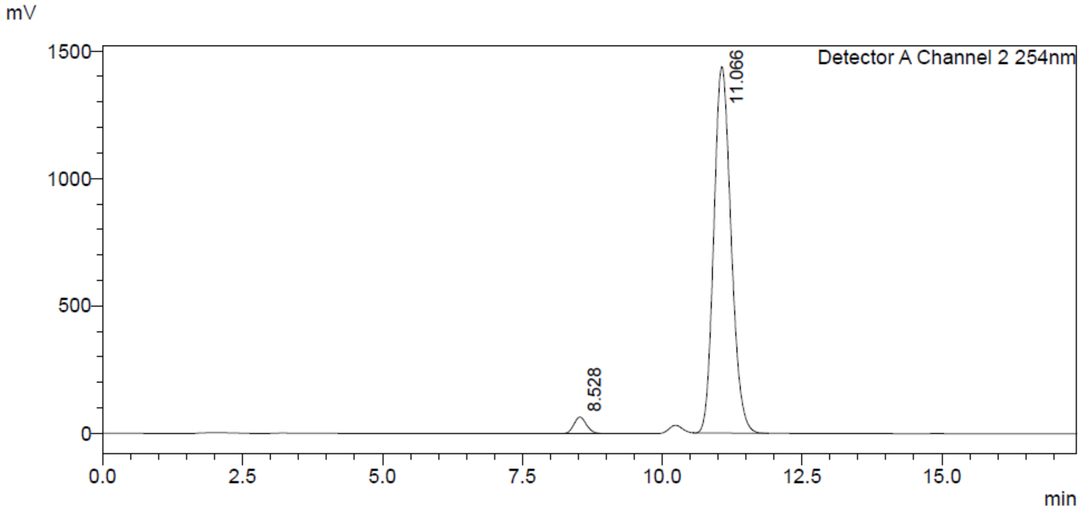

Detector A Channel 2 254nm

Peak#	Ret. Time	Area	Height	Conc.
1	8.528	1023695	65060	3.205
2	11.066	30914050	1438250	96.795
Total		31937745	1503310	

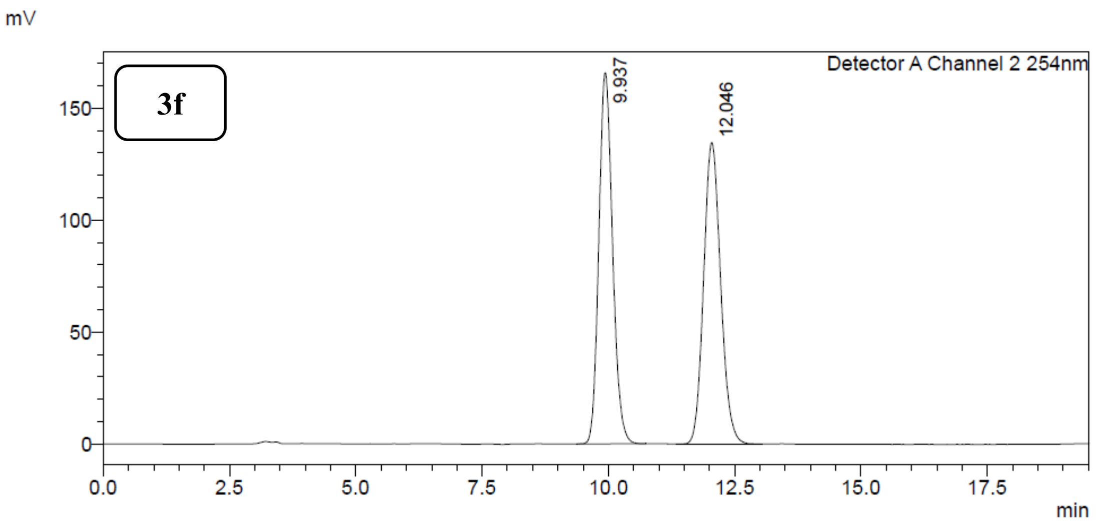

Detector A Channel 2 254nm

Peak#	Ret. Time	Area	Height	Conc.
1	9.937	3138578	165674	49.834
2	12.046	3159482	134701	50.166
Total		6298060	300375	

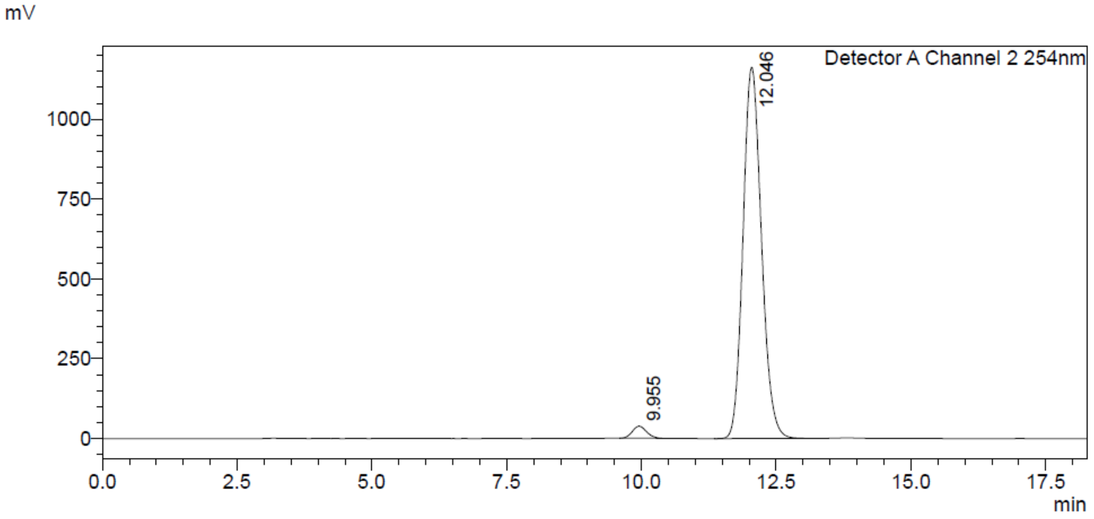

Detector A Channel 2 254nm

Peak#	Ret. Time	Area	Height	Conc.
1	9.955	700287	37725	2.471
2	12.046	27643050	1162552	97.529
Total		28343337	1200276	

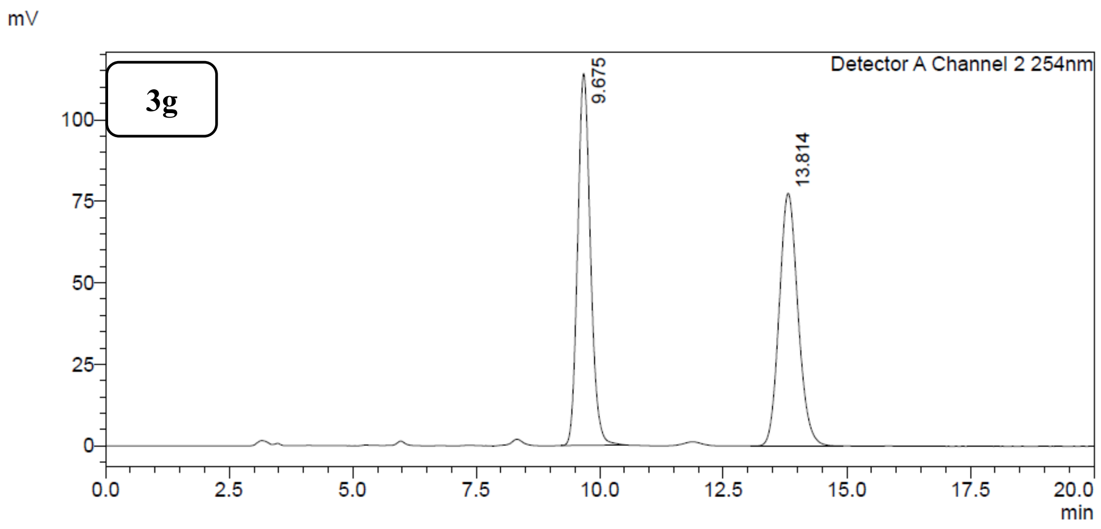

Detector A Channel 2 254nm

Peak#	Ret. Time	Area	Height	Conc.
1	9.675	2055617	114043	50.093
2	13.814	2047980	77491	49.907
Total		4103597	191534	

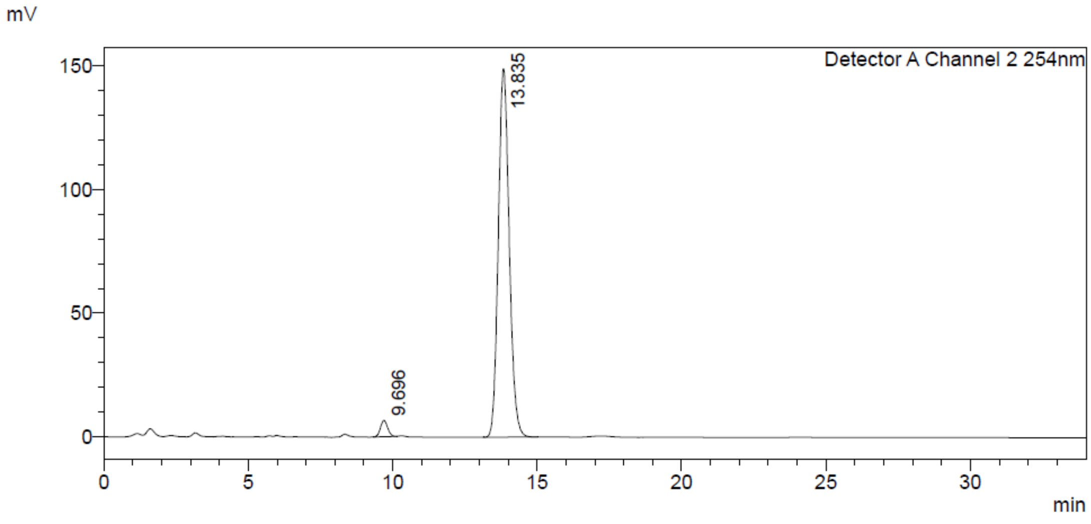

Detector A Channel 2 254nm

Peak#	Ret. Time	Area	Height	Conc.
1	9.696	114391	6628	2.832
2	13.835	3924432	149196	97.168
Total		4038823	155823	

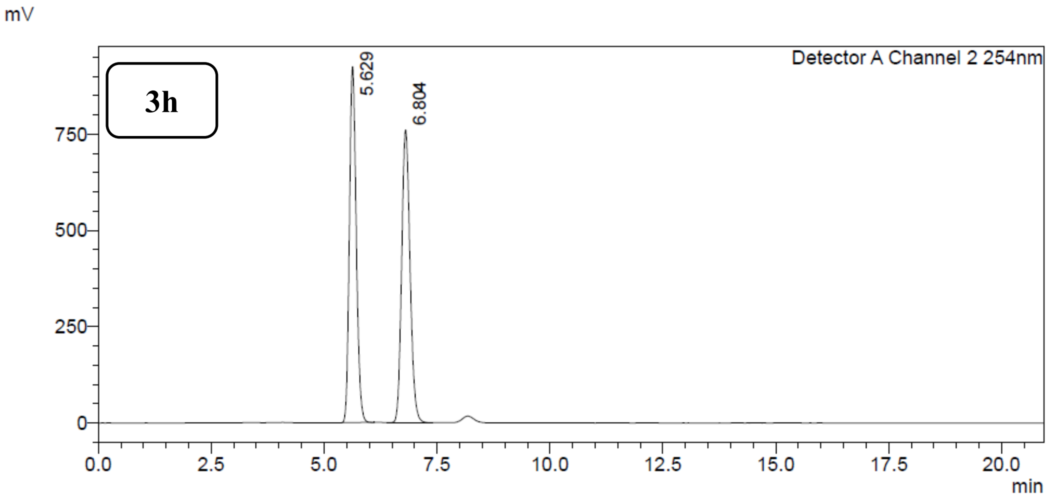

Detector A Channel 2 254nm

Peak#	Ret. Time	Area	Height	Conc.
1	5.629	9758031	924359	49.910
2	6.804	9793317	760414	50.090
Total		19551347	1684773	

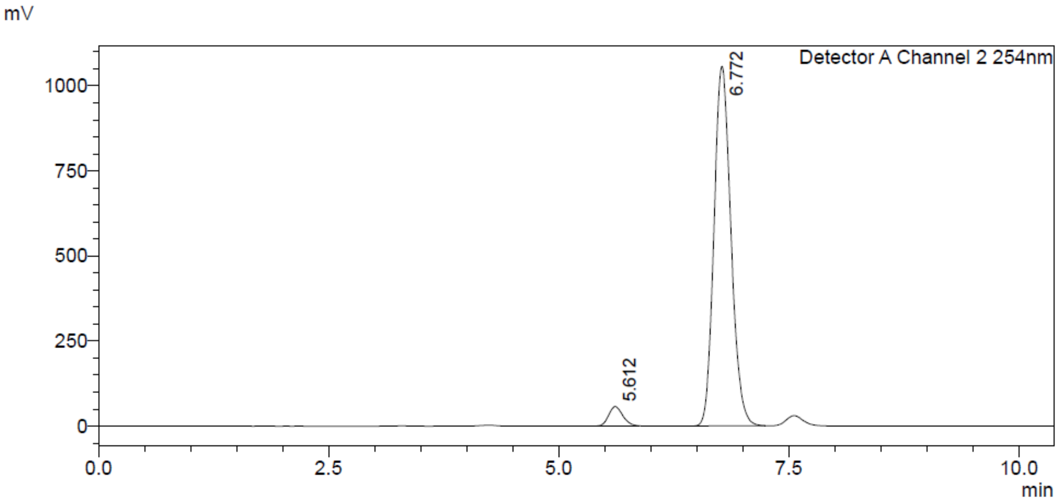

Detector A Channel 2 254nm

Peak#	Ret. Time	Area	Height	Conc.
1	5.612	593446	56524	4.195
2	6.772	13553120	1056439	95.805
Total		14146567	1112963	

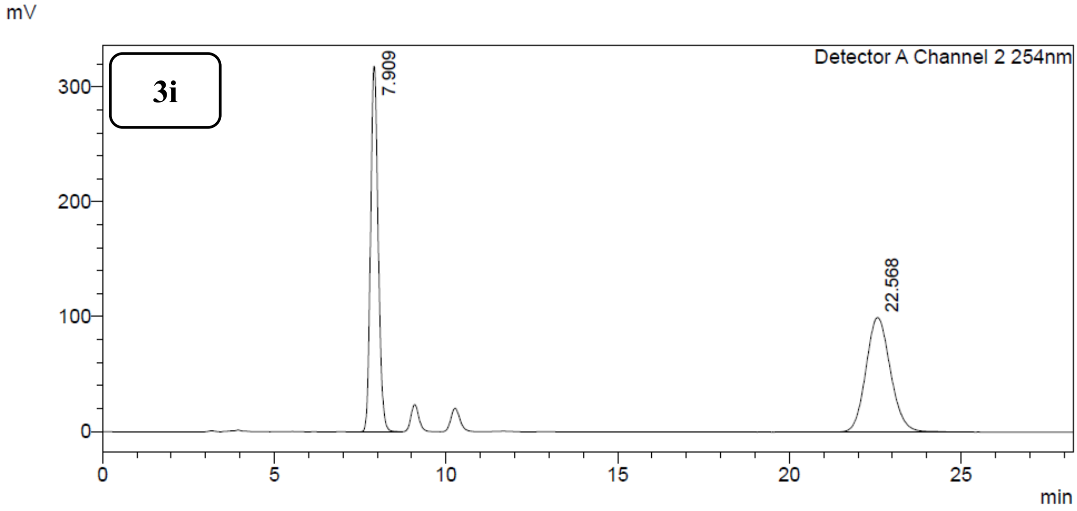

Detector A Channel 2 254nm

Peak#	Ret. Time	Area	Height	Conc.
1	7.909	4920554	318464	49.919
2	22.568	4936548	99673	50.081
Total		9857102	418137	

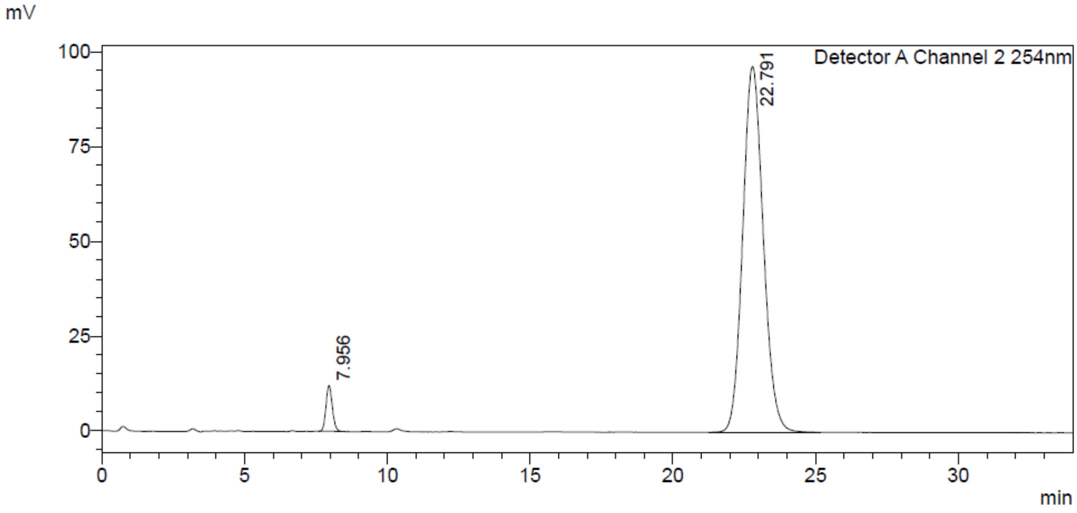

Detector A Channel 2 254nm

Peak#	Ret. Time	Area	Height	Conc.
1	7.956	187175	12127	3.735
2	22.791	4823626	96511	96.265
Total		5010801	108639	

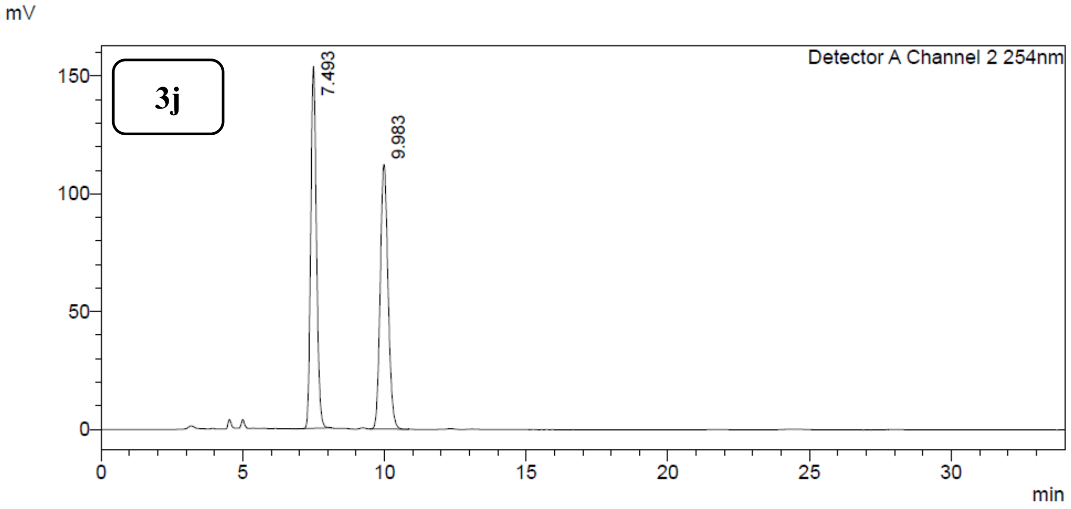

Detector A Channel 2 254nm

Peak#	Ret. Time	Area	Height	Conc.
1	7.493	2157816	153608	50.043
2	9.983	2154067	112386	49.957
Total		4311884	265994	

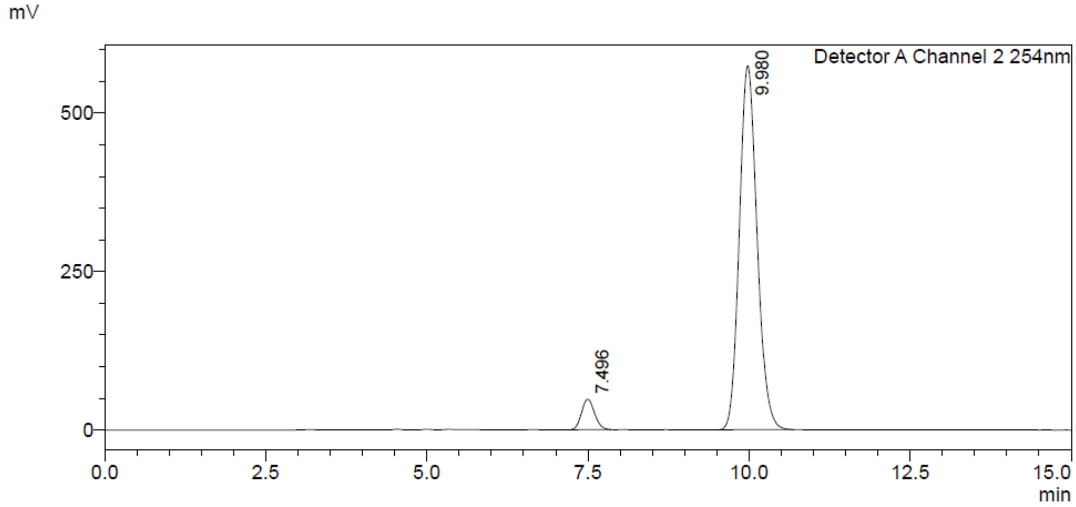

Detector A Channel 2 254nm

Peak#	Ret. Time	Area	Height	Conc.
1	7.496	664096	47989	5.672
2	9.980	11045140	574234	94.328
Total		11709236	622224	

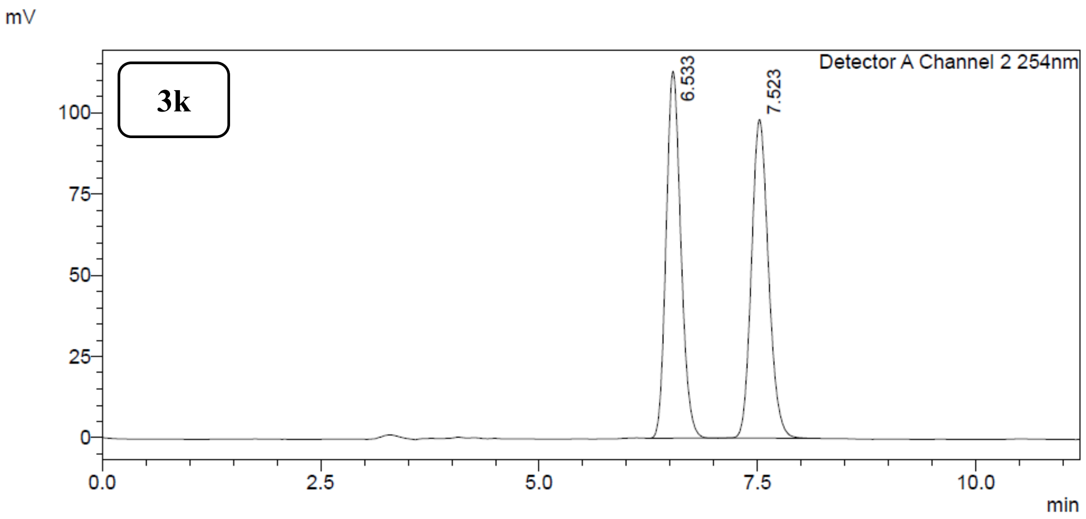

Detector A Channel 2 254nm

Peak#	Ret. Time	Area	Height	Conc.
1	6.533	1321866	112849	49.848
2	7.523	1329920	98022	50.152
Total		2651786	210871	

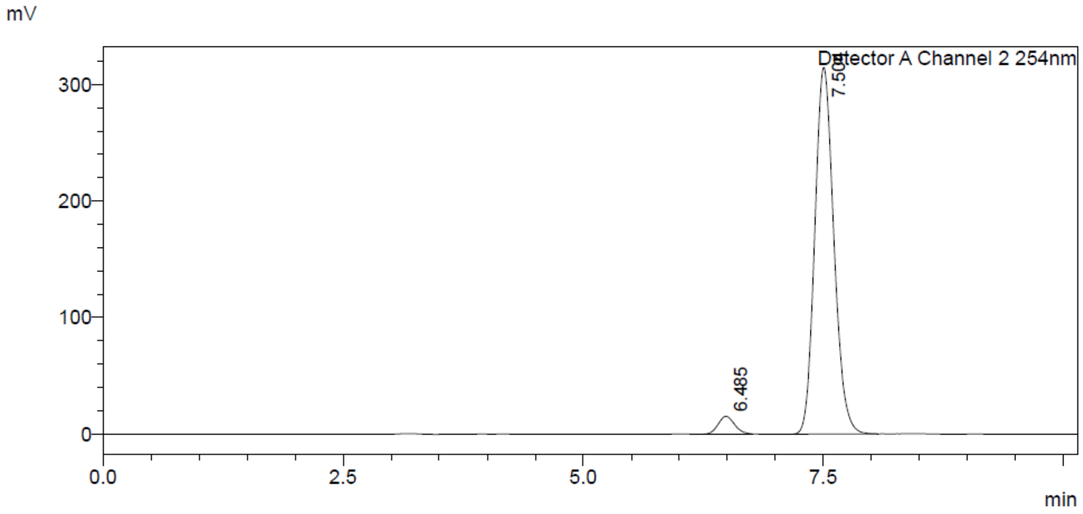

Detector A Channel 2 254nm

Peak#	Ret. Time	Area	Height	Conc.
1	6.485	175703	15088	3.903
2	7.504	4325549	314374	96.097
Total		4501252	329462	

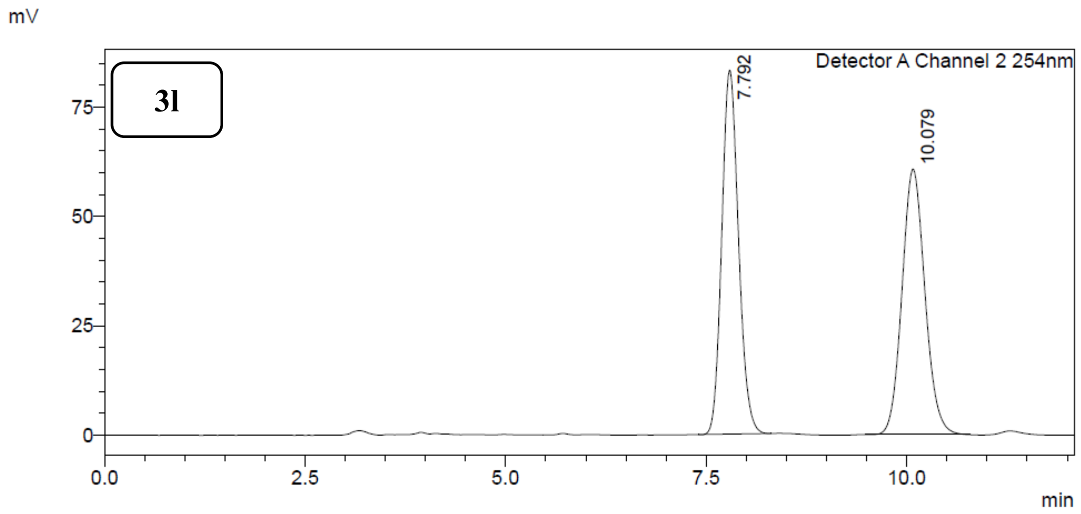

Detector A Channel 2 254nm

Peak#	Ret. Time	Area	Height	Conc.
1	7.792	1208314	83214	49.922
2	10.079	1212071	60693	50.078
Total		2420385	143907	

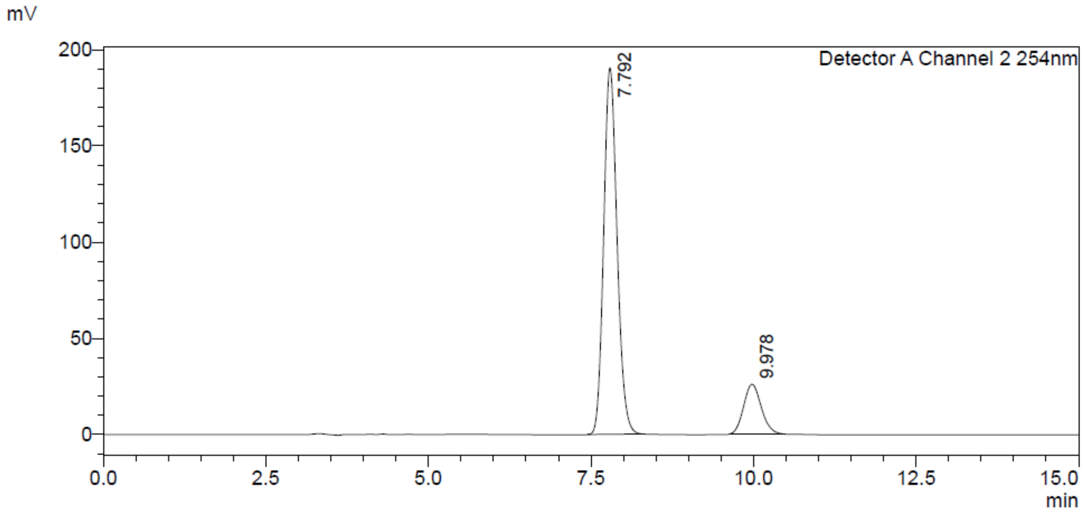

Detector A Channel 2 254nm

Peak#	Ret. Time	Area	Height	Conc.
1	7.792	2740935	190493	84.908
2	9.978	487171	25810	15.092
Total		3228106	216303	

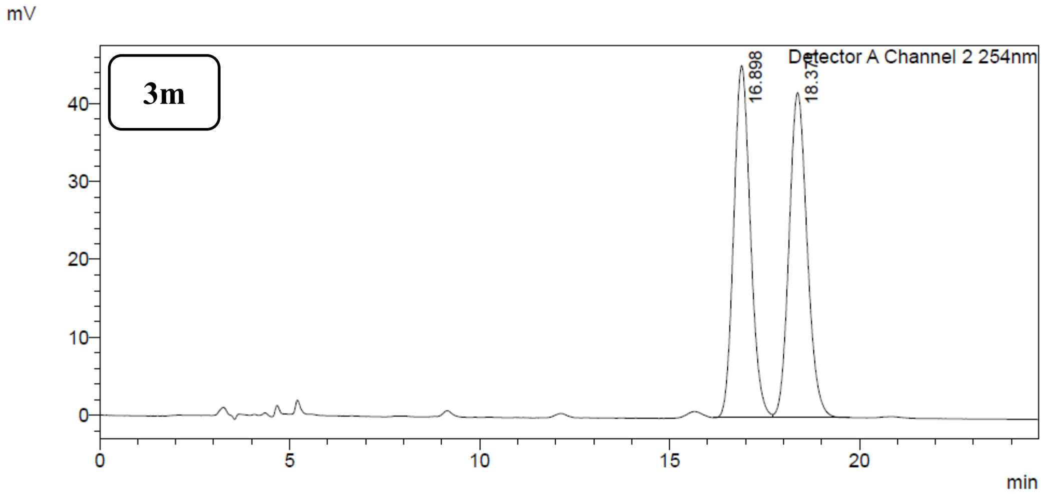

Detector A Channel 2 254nm

Peak#	Ret. Time	Area	Height	Conc.
1	16.898	1378151	45208	49.926
2	18.371	1382219	41736	50.074
Total		2760370	86943	

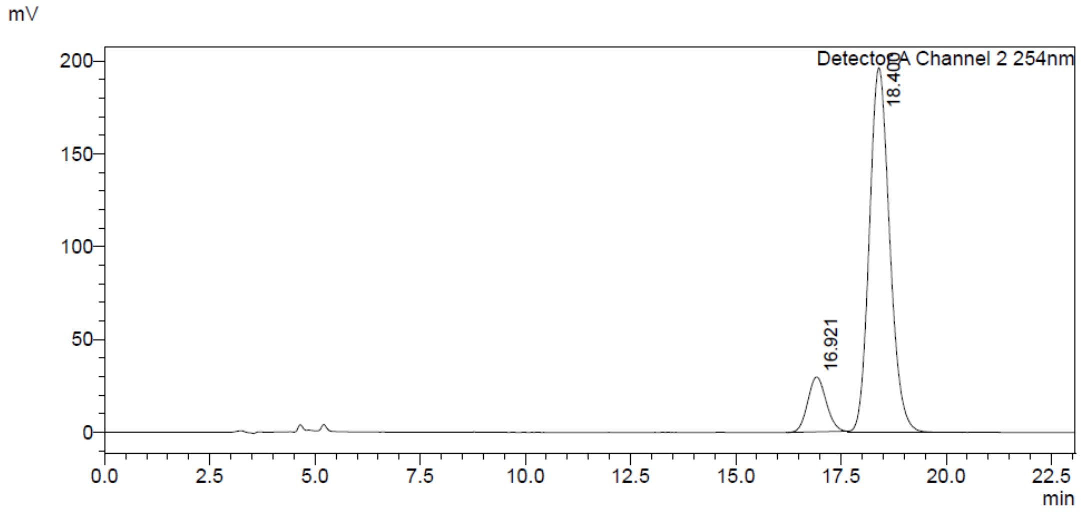

Detector A Channel 2 254nm

Peak#	Ret. Time	Area	Height	Conc.
1	16.921	900571	29602	12.012
2	18.400	6596633	196443	87.988
Total		7497204	226045	

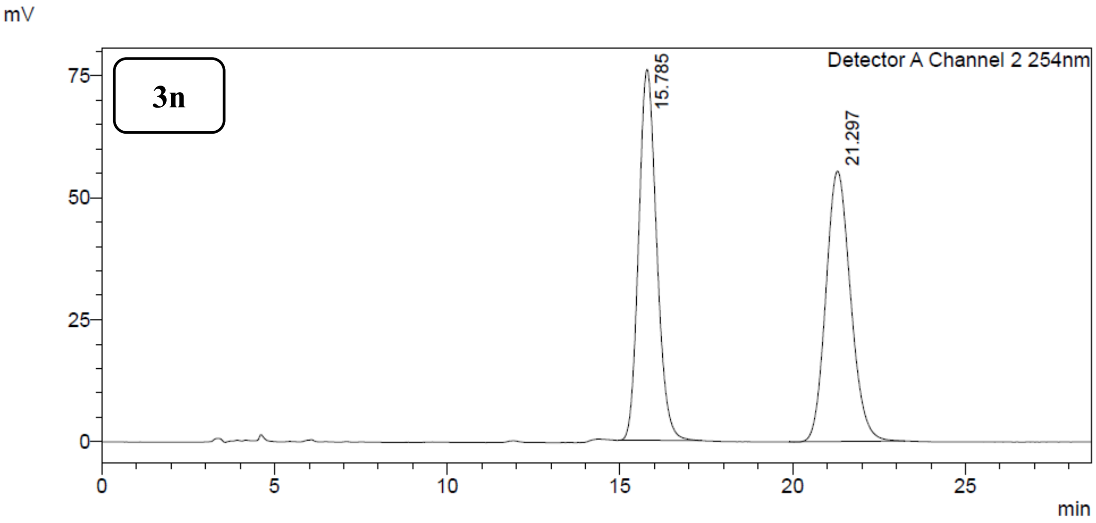

Detector A Channel 2 254nm

Peak#	Ret. Time	Area	Height	Conc.
1	15.785	2752921	76024	49.987
2	21.297	2754351	55413	50.013
Total		5507273	131437	

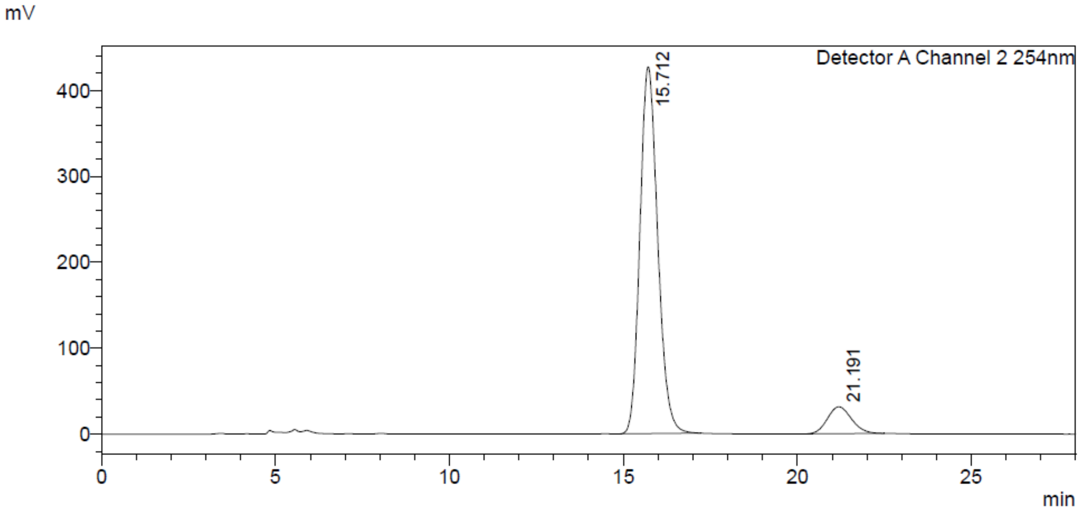

Detector A Channel 2 254nm

Peak#	Ret. Time	Area	Height	Conc.
1	15.712	15191442	426936	90.973
2	21.191	1507434	31210	9.027
Total		16698876	458146	

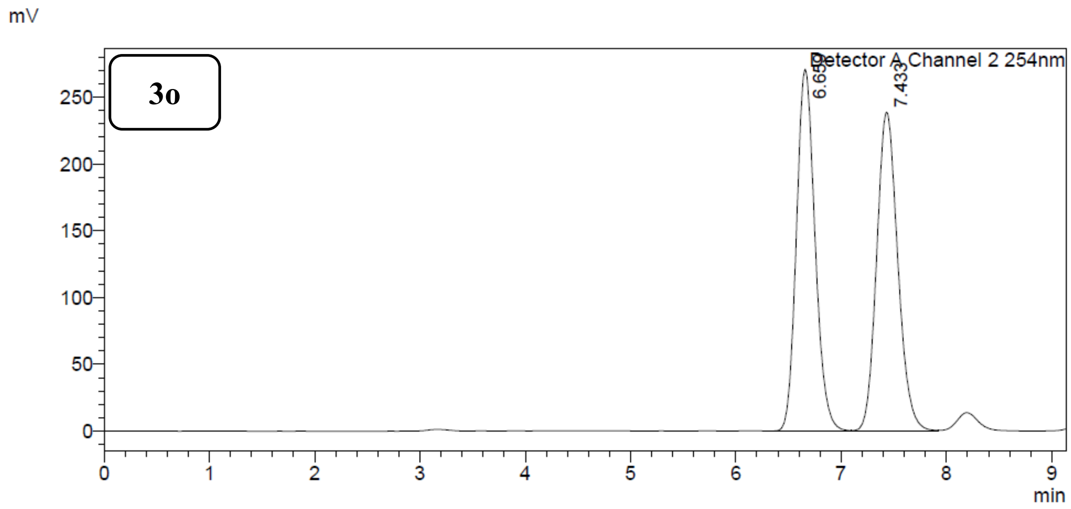

Detector A Channel 2 254nm

Peak#	Ret. Time	Area	Height	Conc.
1	6.659	3327586	271048	49.897
2	7.433	3341365	238912	50.103
Total		6668951	509960	

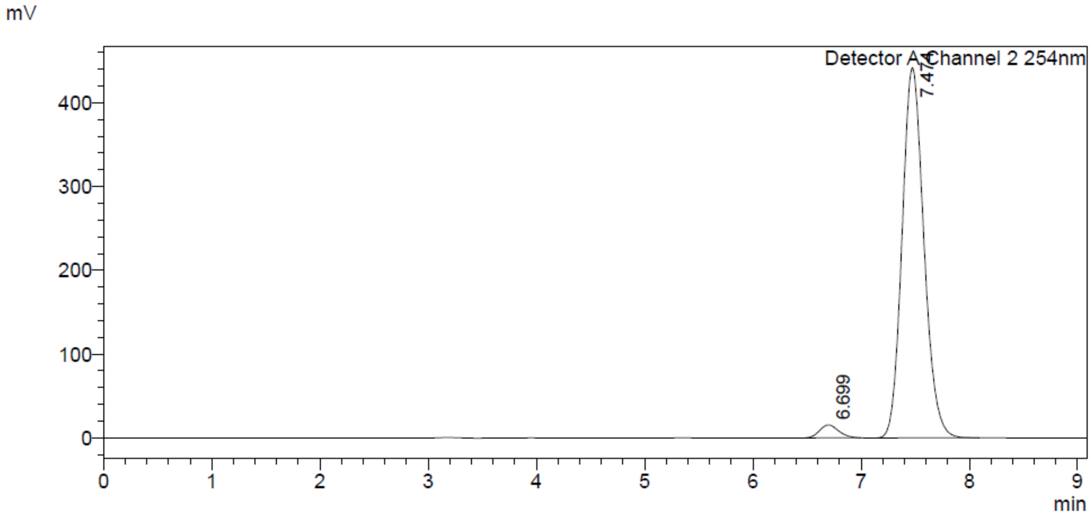

Detector A Channel 2 254nm

Peak#	Ret. Time	Area	Height	Conc.
1	6.699	188837	15438	2.962
2	7.474	6185840	441704	97.038
Total		6374677	457142	

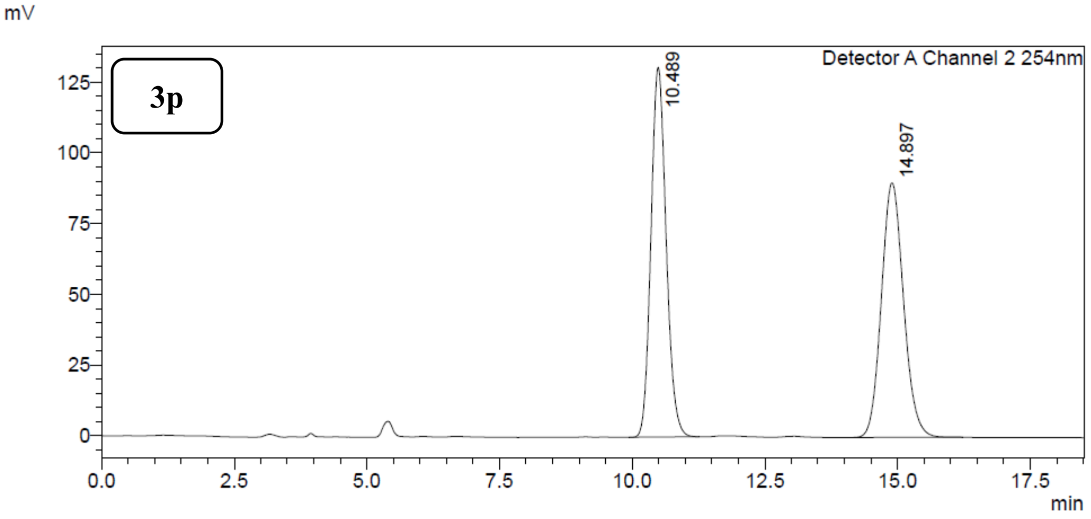

Detector A Channel 2 254nm

Peak#	Ret. Time	Area	Height	Conc.
1	10.489	2604049	130706	49.874
2	14.897	2617195	90001	50.126
Total		5221244	220707	

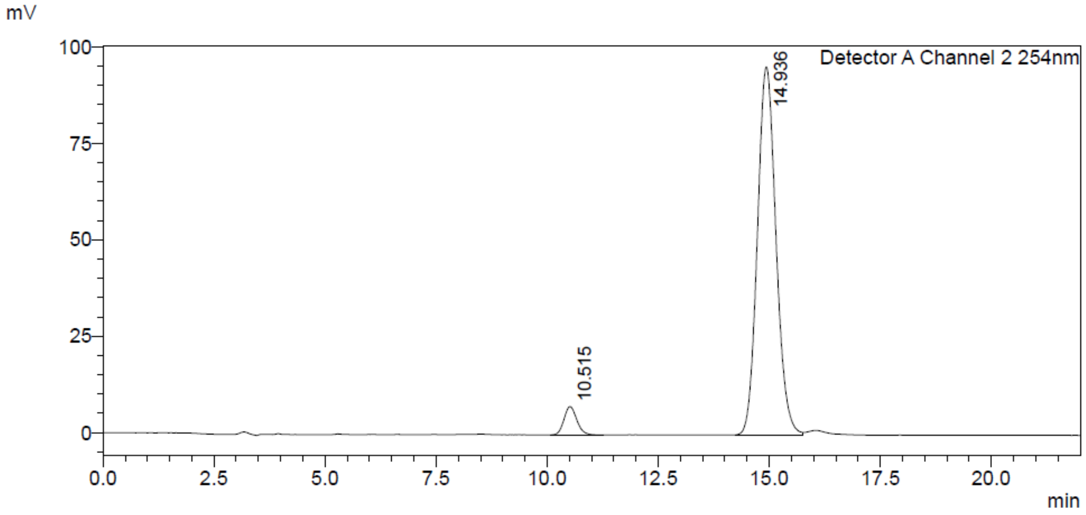

Detector A Channel 2 254nm

Peak#	Ret. Time	Area	Height	Conc.
1	10.515	147031	7337	5.029
2	14.936	2776469	95399	94.971
Total		2923500	102736	

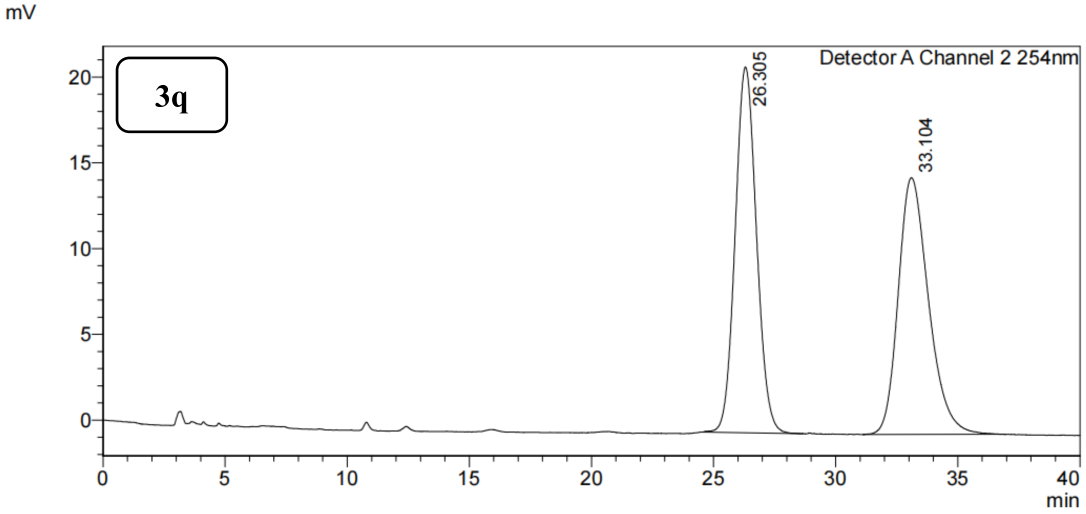

Detector A Channel 2 254nm

Peak#	Ret. Time	Area	Height	Conc.
1	26.305	1293436	21319	50.024
2	33.104	1292205	14968	49.976
Total		2585641	36287	

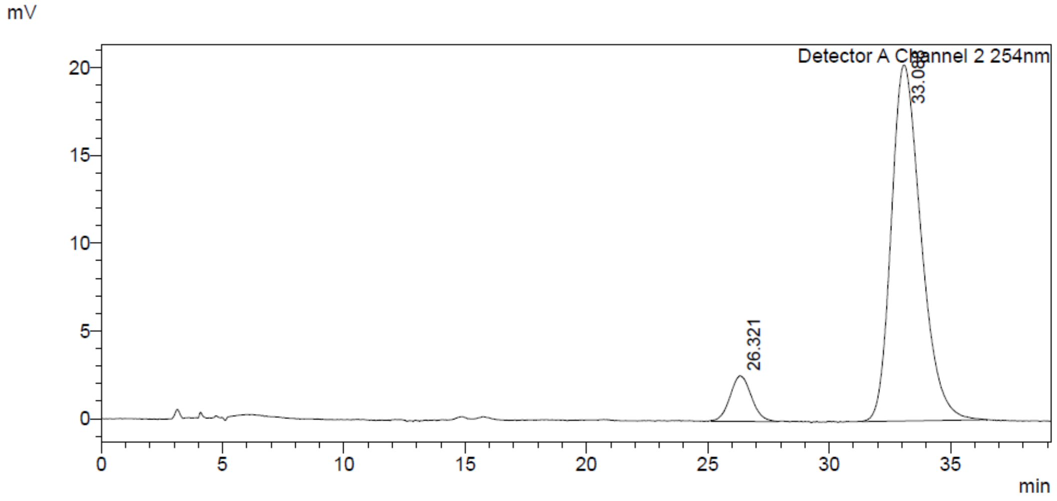

Detector A Channel 2 254nm

Peak#	Ret. Time	Area	Height	Conc.
1	26.321	159017	2597	8.408
2	33.088	1732258	20270	91.592
Total		1891275	22867	

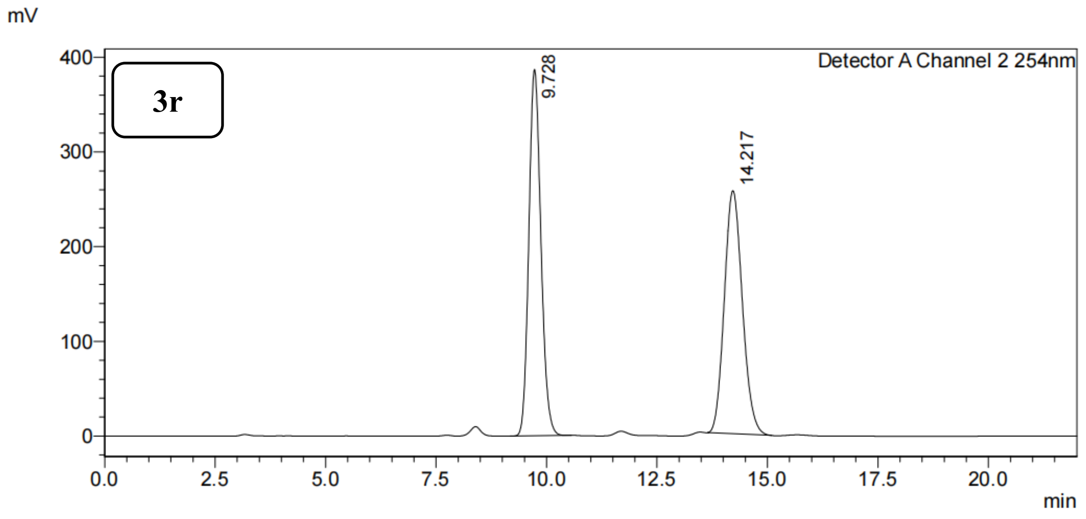

Detector A Channel 2 254nm

Peak#	Ret. Time	Area	Height	Conc.
1	9.728	7336863	386818	50.130
2	14.217	7298866	256802	49.870
Total		14635729	643620	

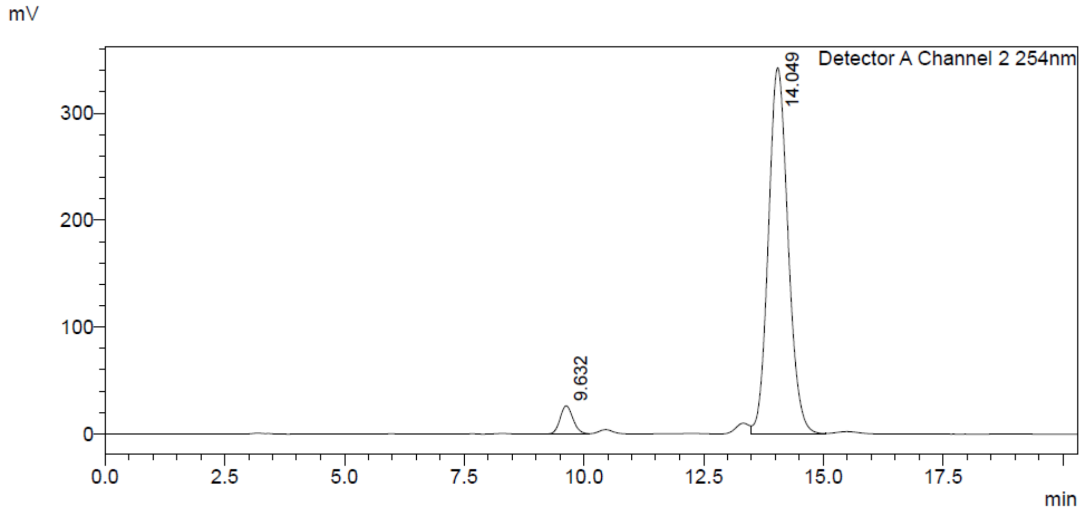

Detector A Channel 2 254nm

Peak#	Ret. Time	Area	Height	Conc.
1	9.632	498969	26425	4.820
2	14.049	9853423	343122	95.180
Total		10352391	369547	

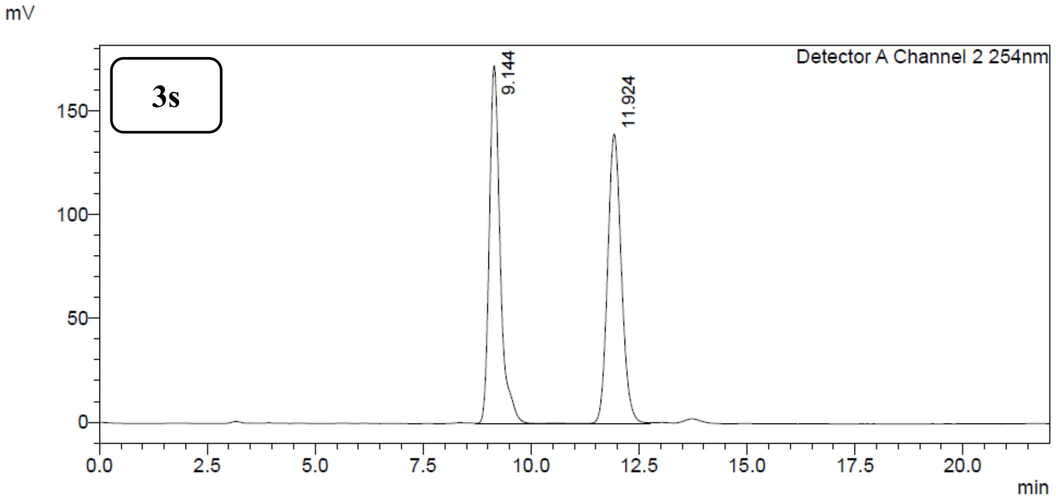

Detector A Channel 2 254nm

Peak#	Ret. Time	Area	Height	Conc.
1	9.144	3039165	172195	49.605
2	11.924	3087580	139229	50.395
Total		6126745	311424	

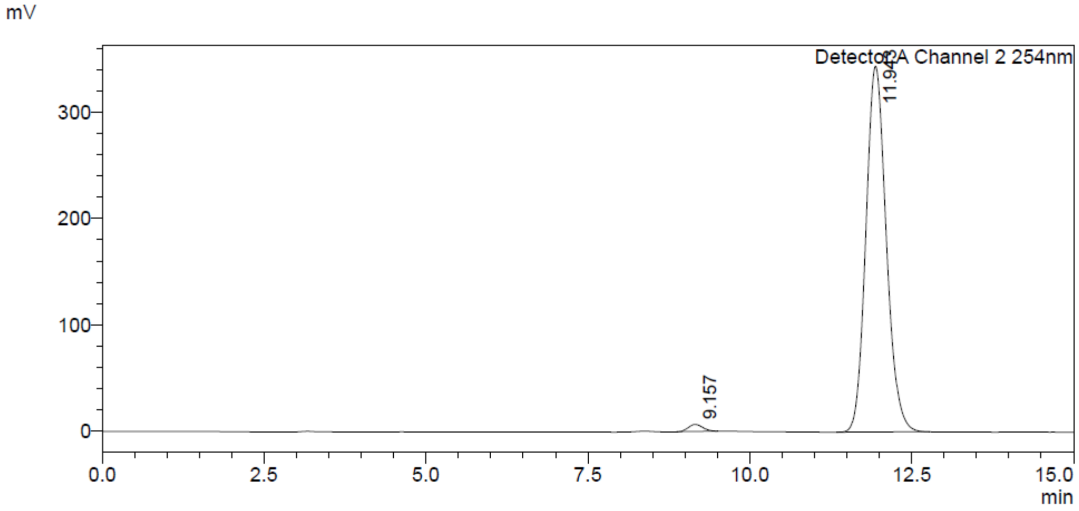

Detector A Channel 2 254nm

Peak#	Ret. Time	Area	Height	Conc.
1	9.157	104112	6685	1.359
2	11.943	7556791	343548	98.641
Total		7660902	350232	

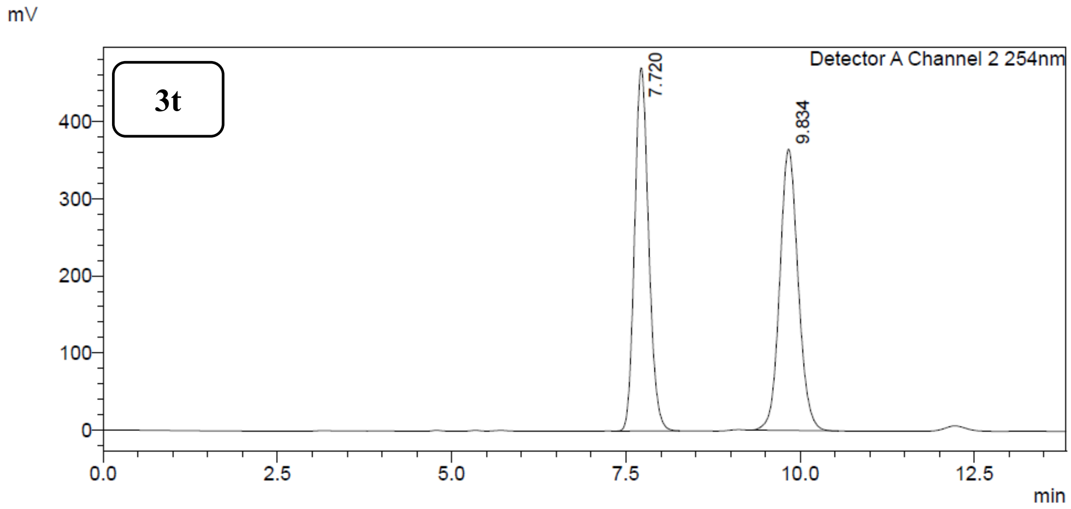

Detector A Channel 2 254nm

Peak#	Ret. Time	Area	Height	Conc.
1	7.720	6591752	470874	49.552
2	9.834	6711011	365040	50.448
Total		13302763	835915	

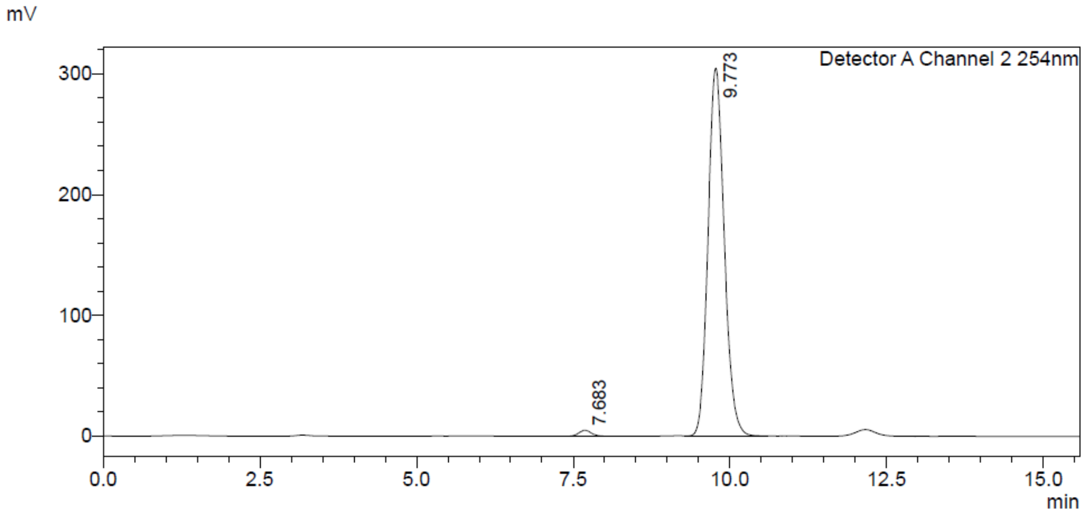

Detector A Channel 2 254nm

Peak#	Ret. Time	Area	Height	Conc.
1	7.683	62645	4725	1.129
2	9.773	5485902	304545	98.871
Total		5548547	309270	

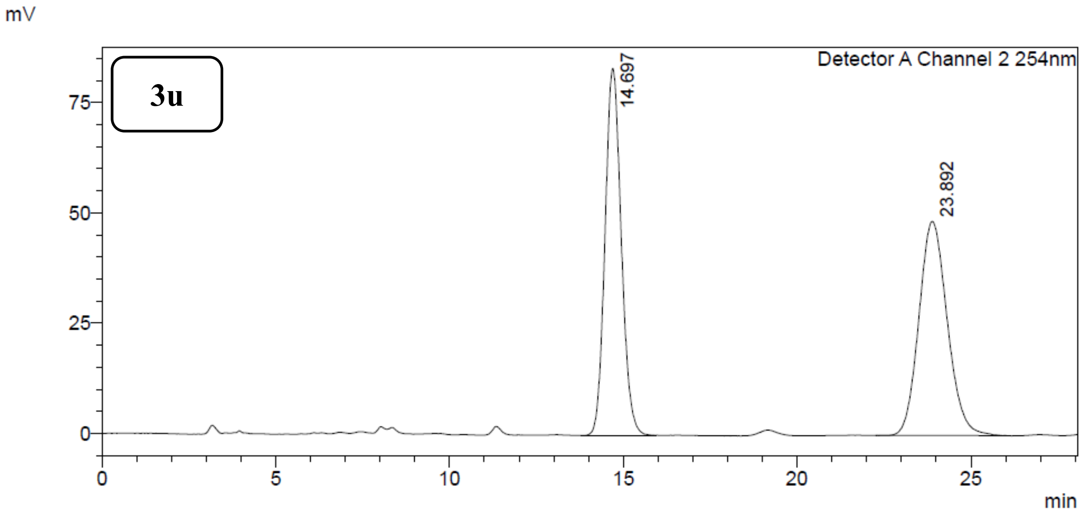

Detector A Channel 2 254nm

Peak#	Ret. Time	Area	Height	Conc.
1	14.697	2704358	83226	49.530
2	23.892	2755701	48522	50.470
Total		5460059	131748	

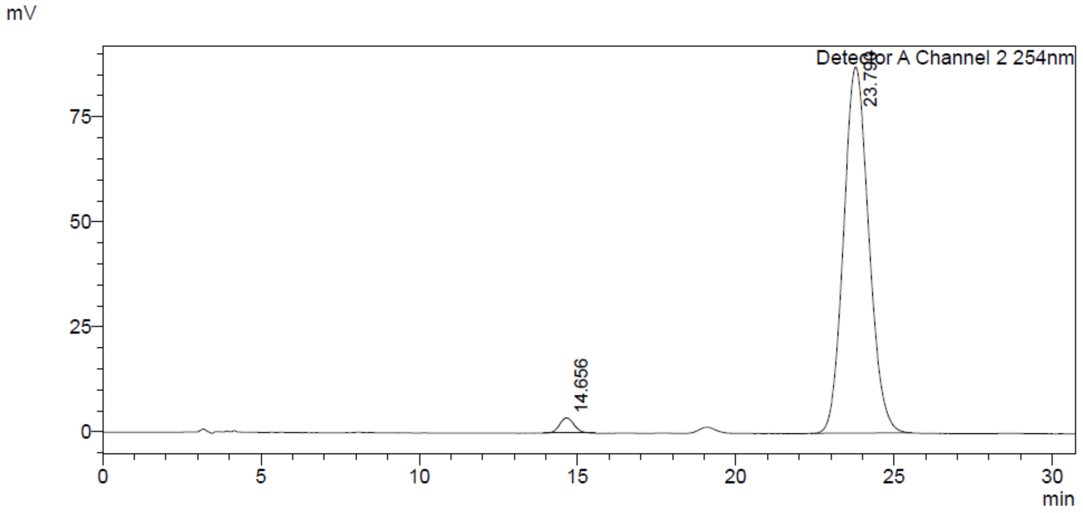

Detector A Channel 2 254nm

Peak#	Ret. Time	Area	Height	Conc.
1	14.656	116661	3632	2.361
2	23.790	4823487	87090	97.639
Total		4940147	90722	

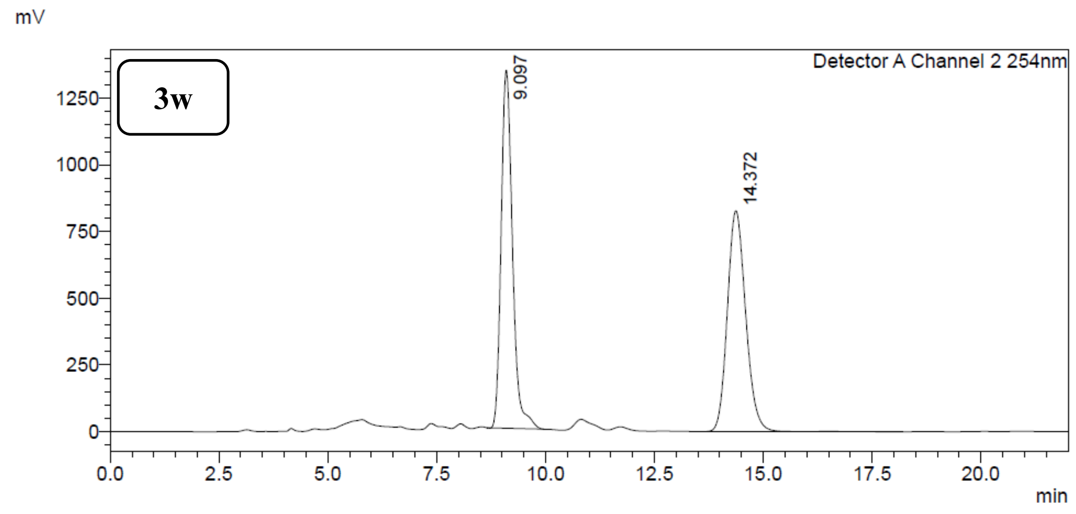

Detector A Channel 2 254nm

Peak#	Ret. Time	Area	Height	Conc.
1	9.097	24347708	1342998	49.960
2	14.372	24387104	828877	50.040
Total		48734812	2171875	

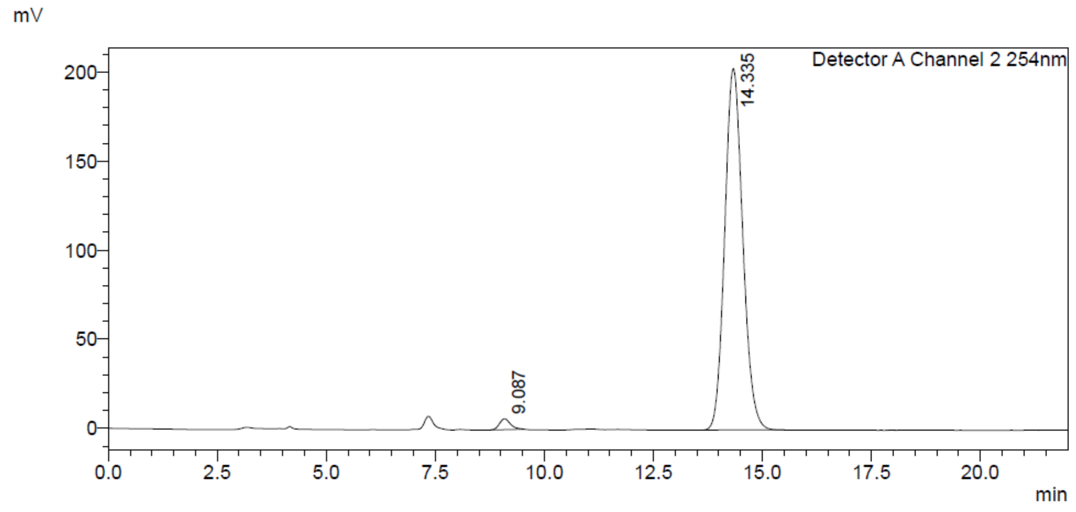

Detector A Channel 2 254nm

Peak#	Ret. Time	Area	Height	Conc.
1	9.087	105703	6051	1.775
2	14.335	5847746	202969	98.225
Total		5953449	209020	

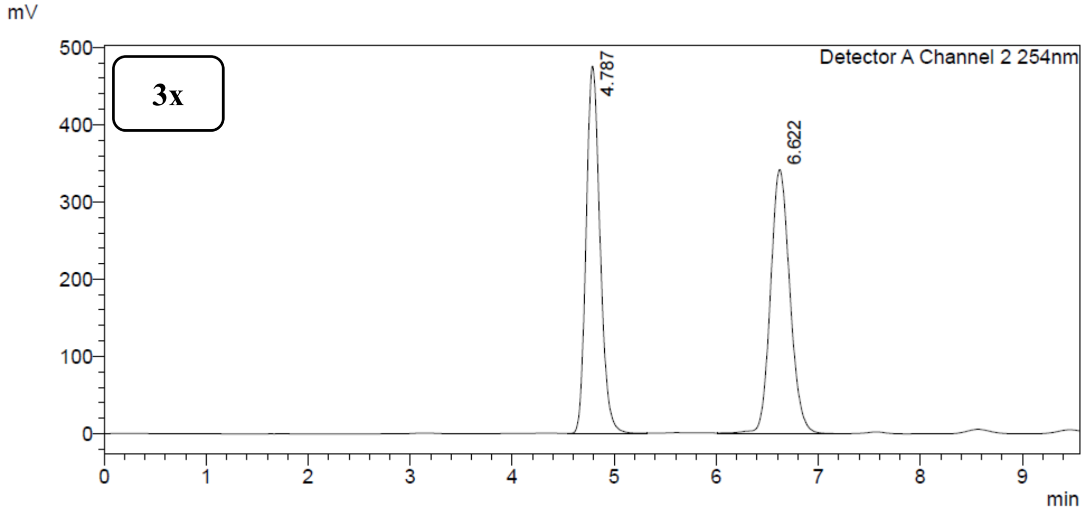

Detector A Channel 2 254nm

Peak#	Ret. Time	Area	Height	Conc.
1	4.787	4537025	476025	49.903
2	6.622	4554611	342289	50.097
Total		9091635	818314	

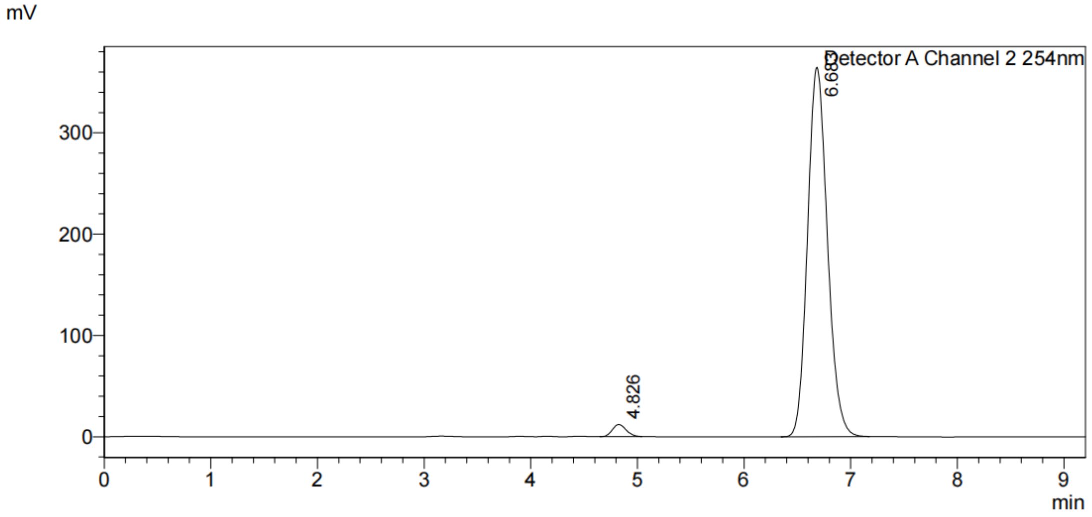

Detector A Channel 2 254nm

Peak#	Ret. Time	Area	Height	Conc.
1	4.826	107454	12002	2.227
2	6.683	4717862	364627	97.773
Total		4825316	376629	

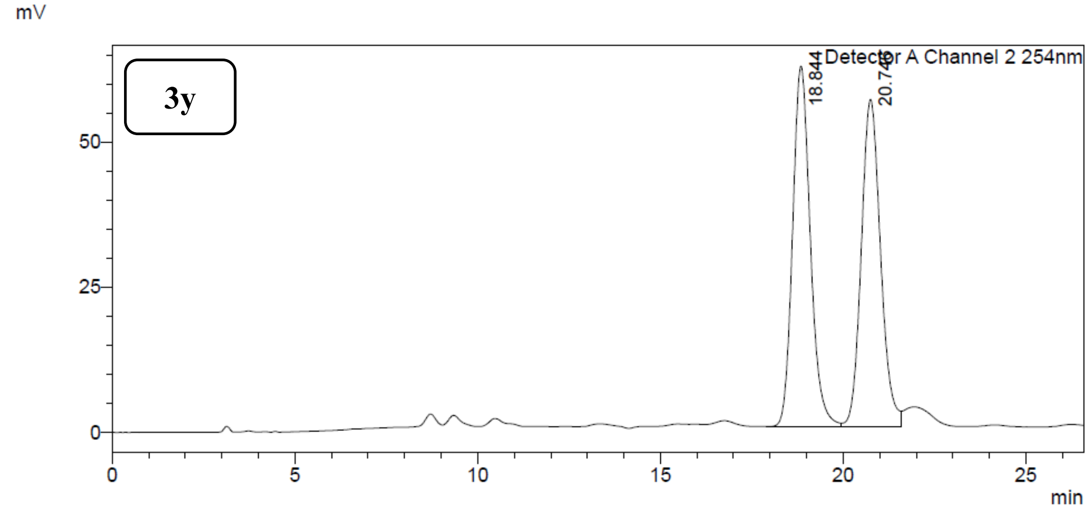

Detector A Channel 2 254nm

Peak#	Ret. Time	Area	Height	Conc.
1	18.844	2091727	62047	50.278
2	20.746	2068617	56352	49.722
Total		4160343	118399	

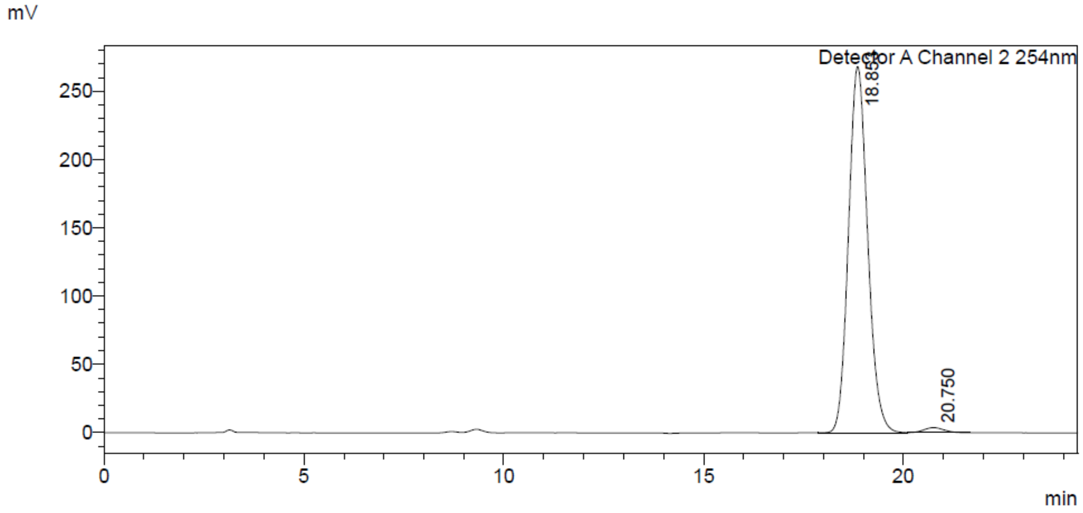

Detector A Channel 2 254nm

Peak#	Ret. Time	Area	Height	Conc.
1	18.853	8957620	268102	98.635
2	20.750	123970	3596	1.365
Total		9081590	271697	

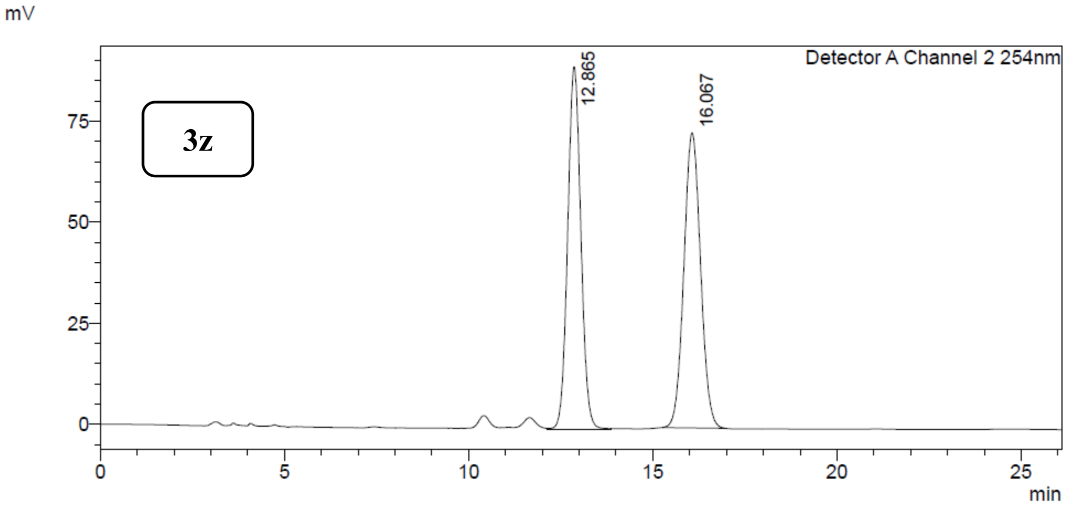

Detector A Channel 2 254nm

Peak#	Ret. Time	Area	Height	Conc.
1	12.865	2250183	89819	49.425
2	16.067	2302507	73051	50.575
Total		4552690	162870	

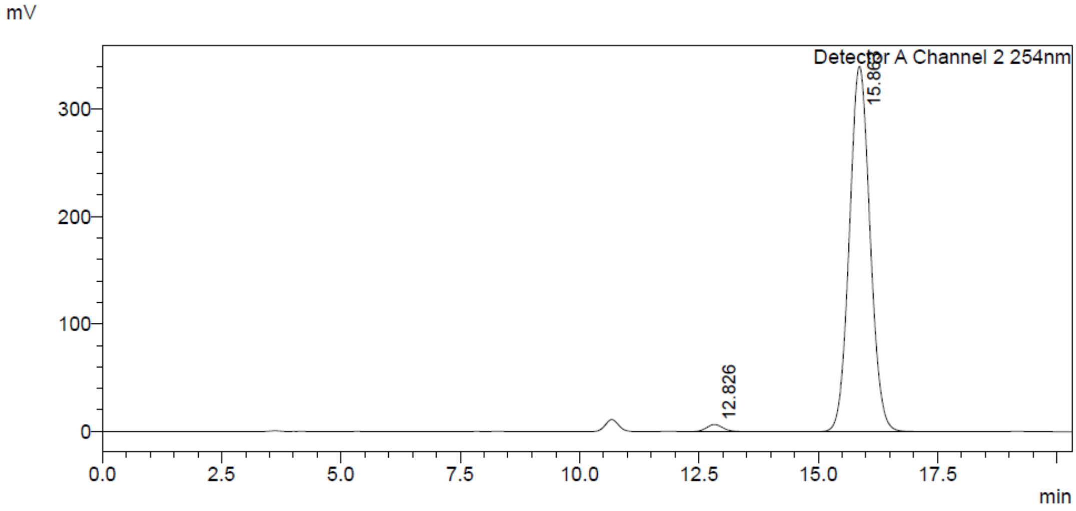

Detector A Channel 2 254nm

Peak#	Ret. Time	Area	Height	Conc.
1	12.826	149597	6454	1.441
2	15.863	10229950	340101	98.559
Total		10379547	346555	

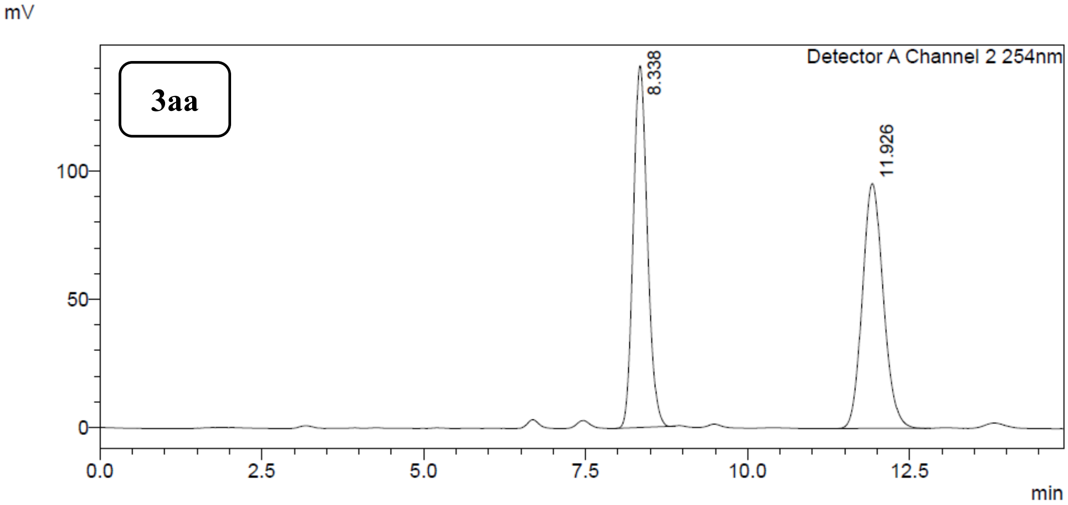

Detector A Channel 2 254nm

Peak#	Ret. Time	Area	Height	Conc.
1	8.338	2131506	141038	49.767
2	11.926	2151443	95452	50.233
Total		4282949	236490	

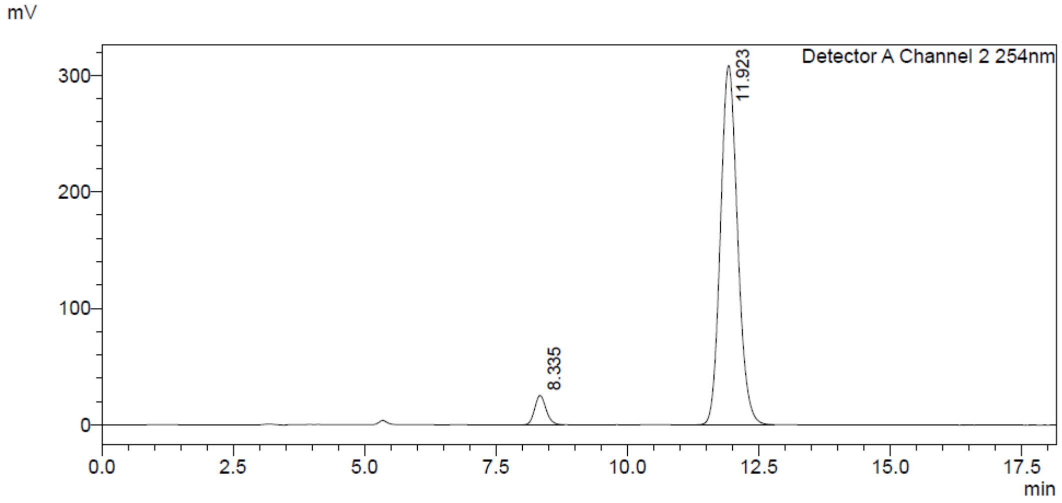

Detector A Channel 2 254nm

Peak#	Ret. Time	Area	Height	Conc.
1	8.335	375911	25052	5.120
2	11.923	6966563	308400	94.880
Total		7342474	333452	

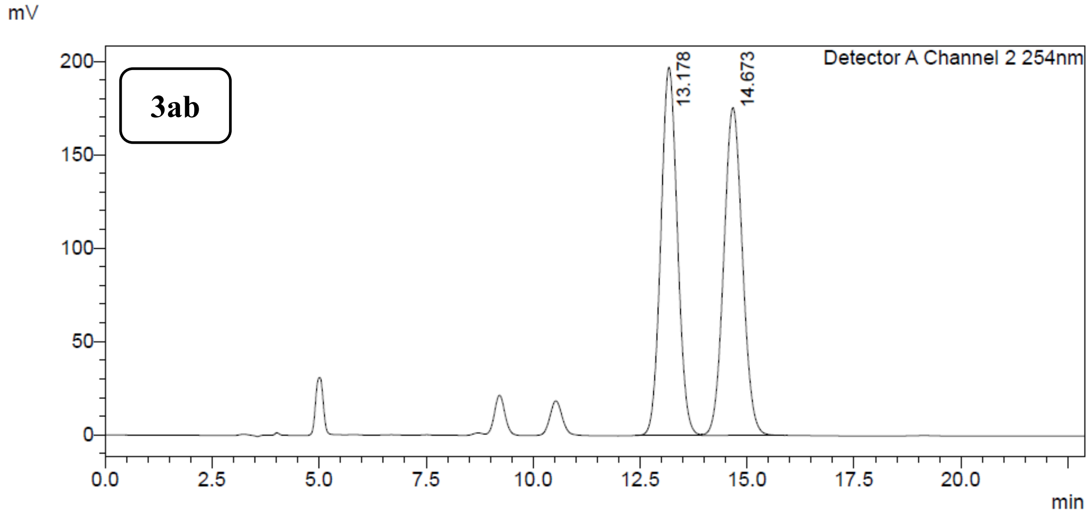

Detector A Channel 2 254nm

Peak#	Ret. Time	Area	Height	Conc.
1	13.178	5315970	197235	49.957
2	14.673	5325173	175517	50.043
Total		10641143	372752	

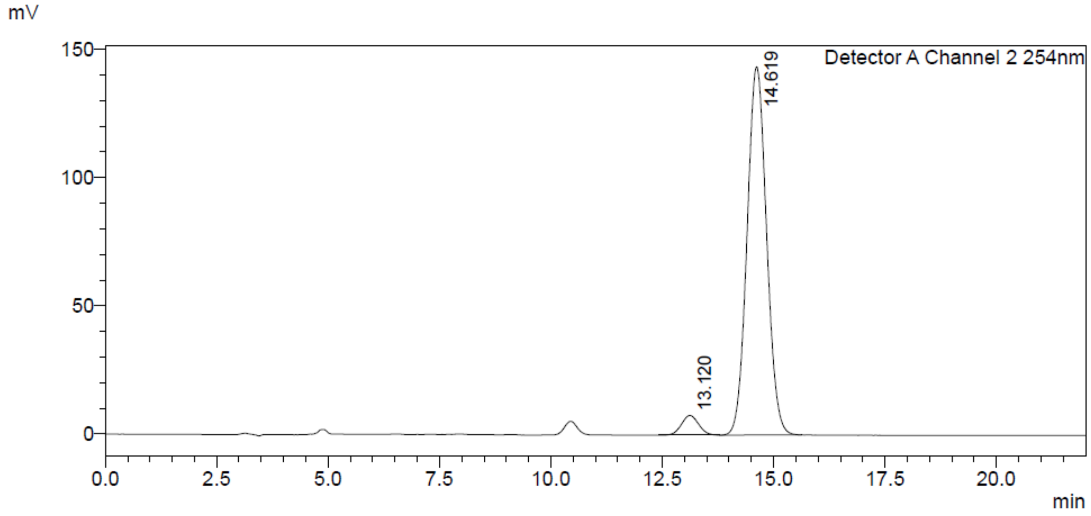

Detector A Channel 2 254nm

Peak#	Ret. Time	Area	Height	Conc.
1	13.120	209662	7705	4.580
2	14.619	4367967	143494	95.420
Total		4577629	151199	

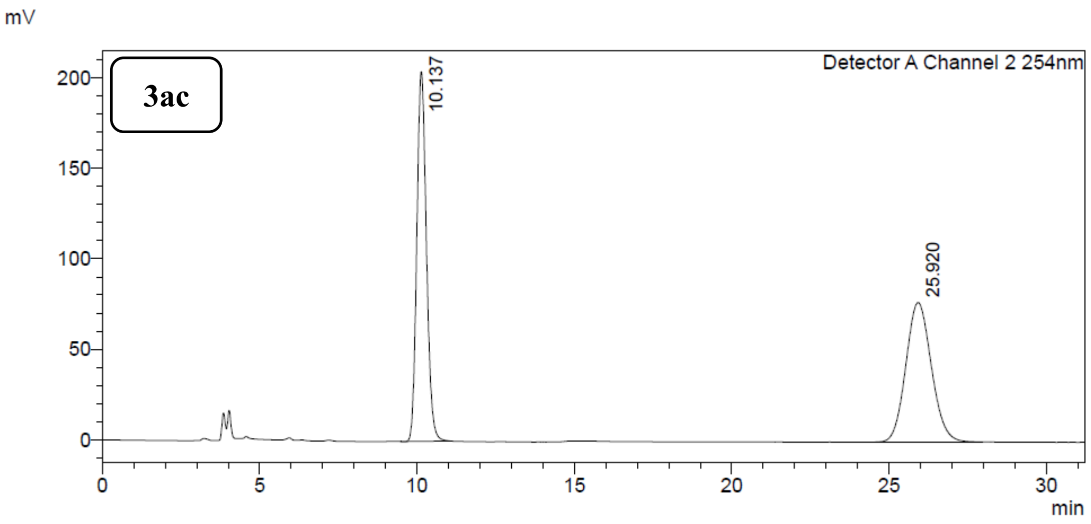

Detector A Channel 2 254nm

Peak#	Ret. Time	Area	Height	Conc.
1	10.137	4325178	204051	49.952
2	25.920	4333420	76984	50.048
Total		8658598	281035	

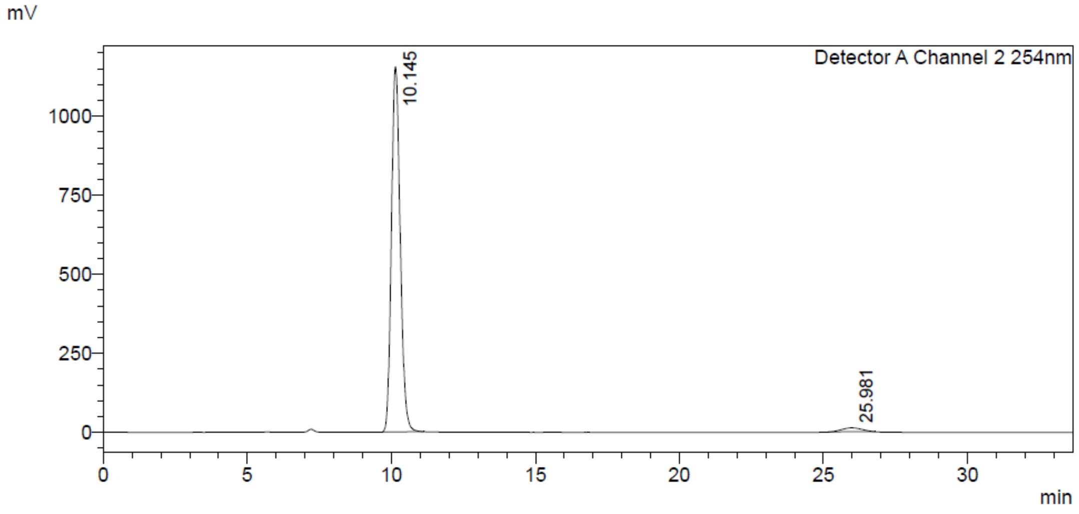

Detector A Channel 2 254nm

Peak#	Ret. Time	Area	Height	Conc.
1	10.145	24597560	1156164	97.583
2	25.981	609222	12348	2.417
Total		25206782	1168513	

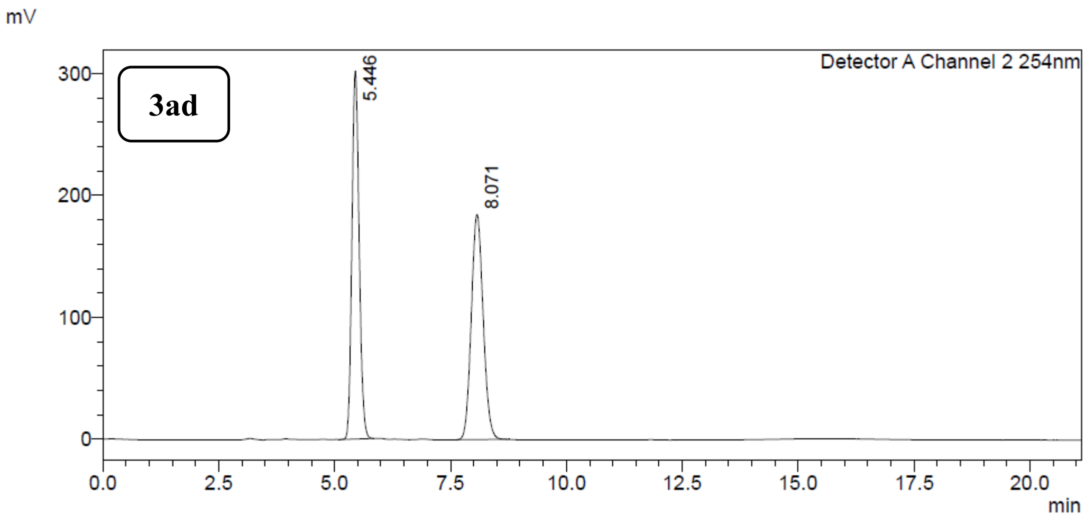

Detector A Channel 2 254nm

Peak#	Ret. Time	Area	Height	Conc.
1	5.446	3282028	302020	49.899
2	8.071	3295357	184721	50.101
Total		6577385	486741	

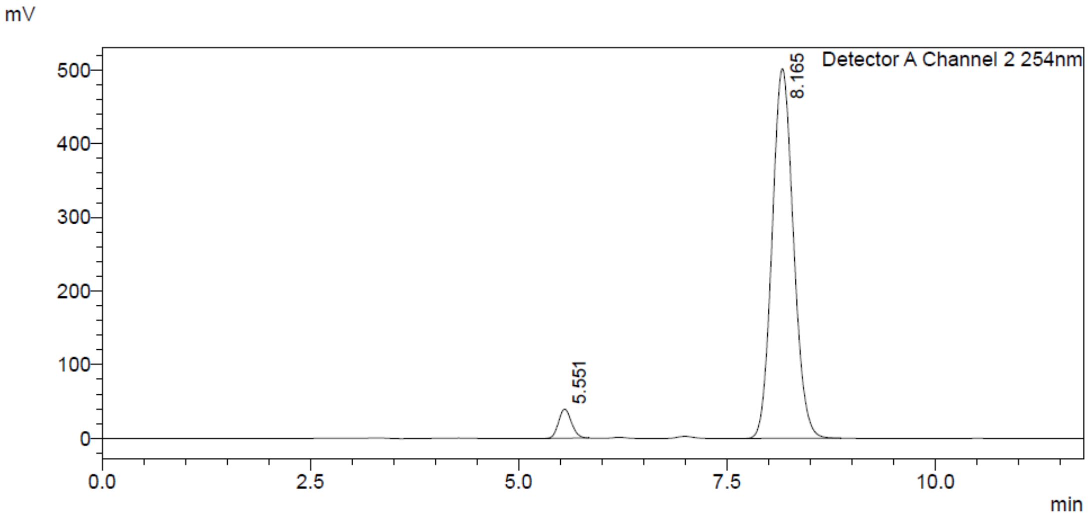

Detector A Channel 2 254nm

Peak#	Ret. Time	Area	Height	Conc.
1	5.551	425161	39501	4.547
2	8.165	8925508	501320	95.453
Total		9350669	540821	

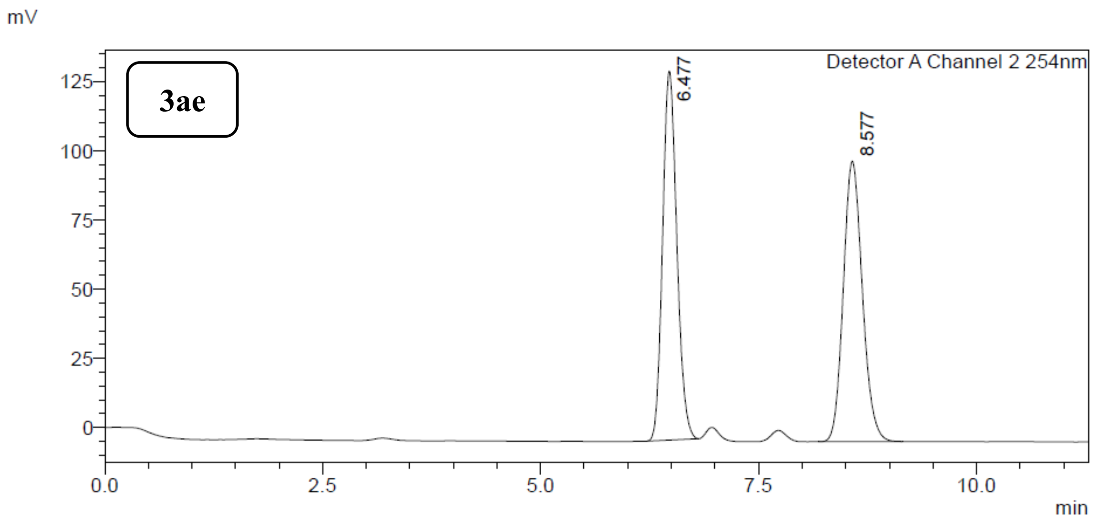

Detector A Channel 2 254nm

Peak#	Ret. Time	Area	Height	Conc.
1	6.477	1518957	133401	49.641
2	8.577	1540920	101359	50.359
Total		3059877	234760	

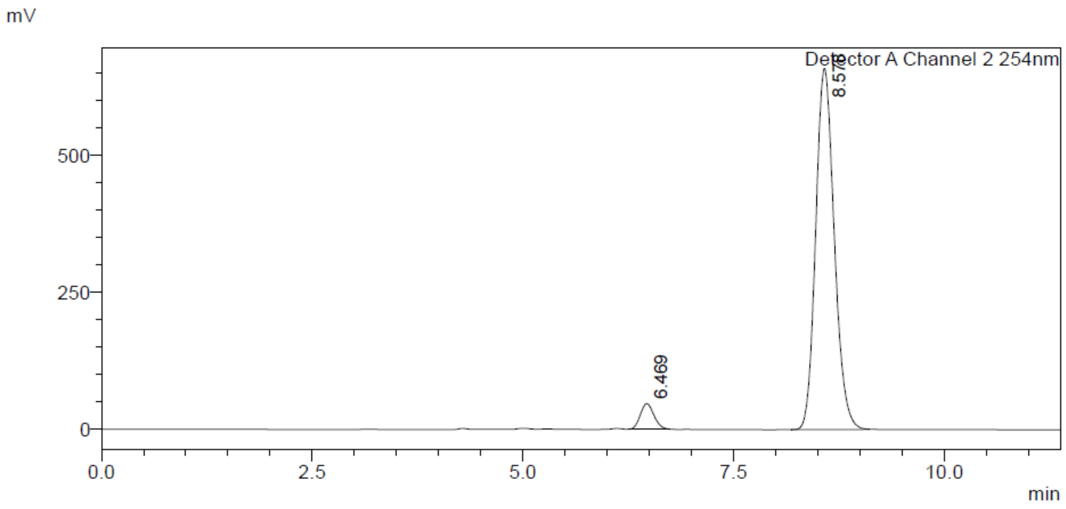

Detector A Channel 2 254nm

Peak#	Ret. Time	Area	Height	Conc.
1	6.469	522482	46398	4.925
2	8.578	10086793	658595	95.075
Total		10609275	704992	

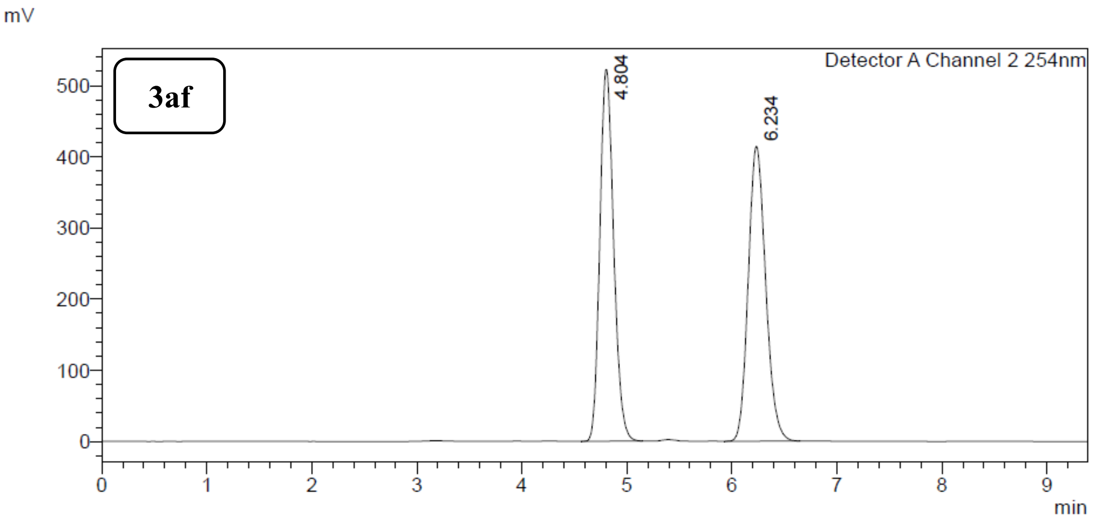

Detector A Channel 2 254nm

Peak#	Ret. Time	Area	Height	Conc.
1	4.804	4787078	521968	49.999
2	6.234	4787323	414262	50.001
Total		9574401	936229	

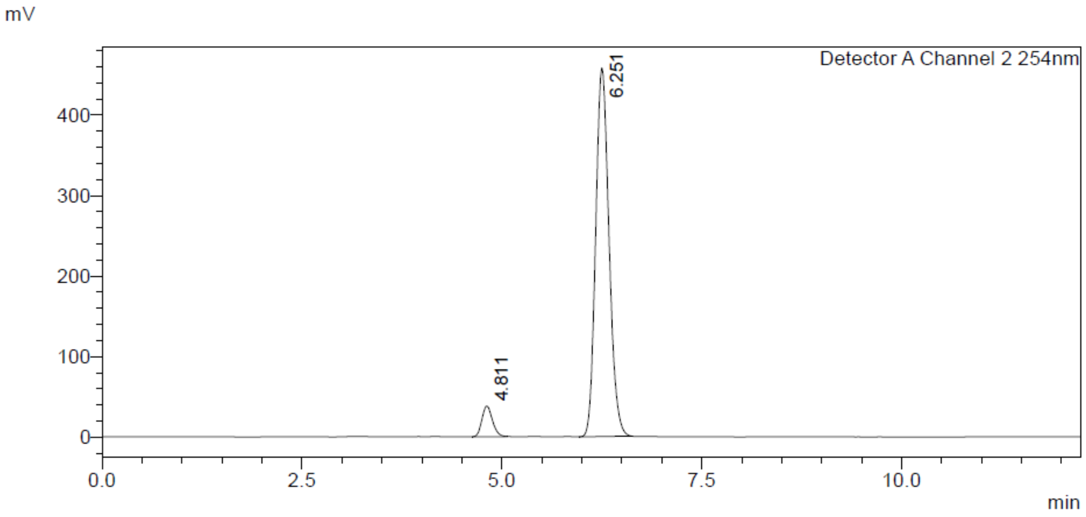

Detector A Channel 2 254nm

Peak#	Ret. Time	Area	Height	Conc.
1	4.811	347231	37965	6.134
2	6.251	5313962	458345	93.866
Total		5661193	496310	

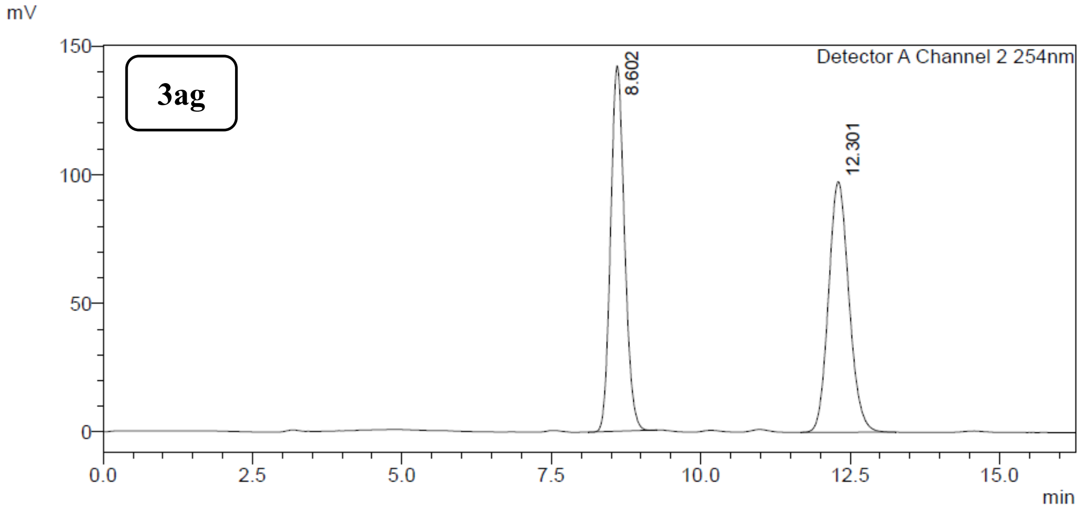

Detector A Channel 2 254nm

Peak#	Ret. Time	Area	Height	Conc.
1	8.602	2304143	142121	49.814
2	12.301	2321396	97456	50.186
Total		4625539	239577	

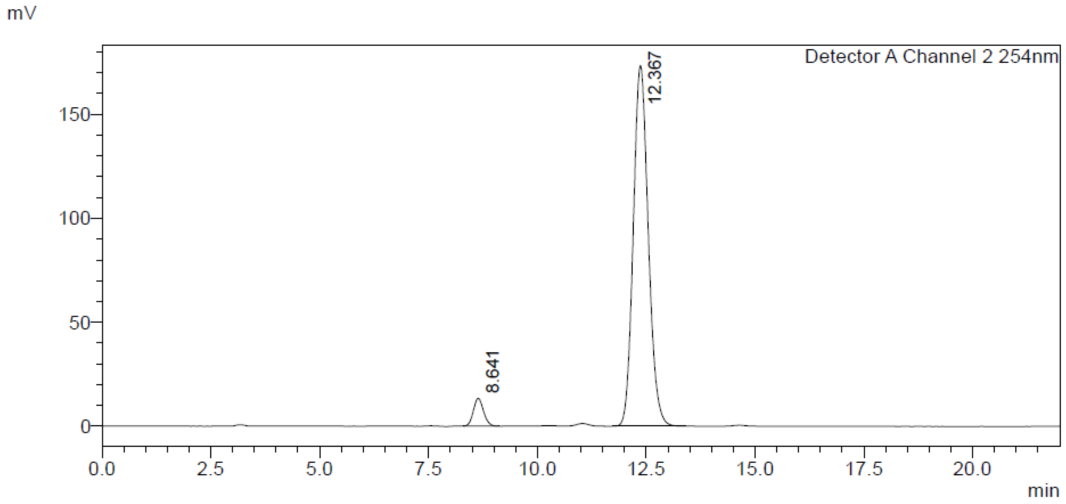

Detector A Channel 2 254nm

Peak#	Ret. Time	Area	Height	Conc.
1	8.641	216337	13362	4.943
2	12.367	4160044	173427	95.057
Total		4376381	186789	

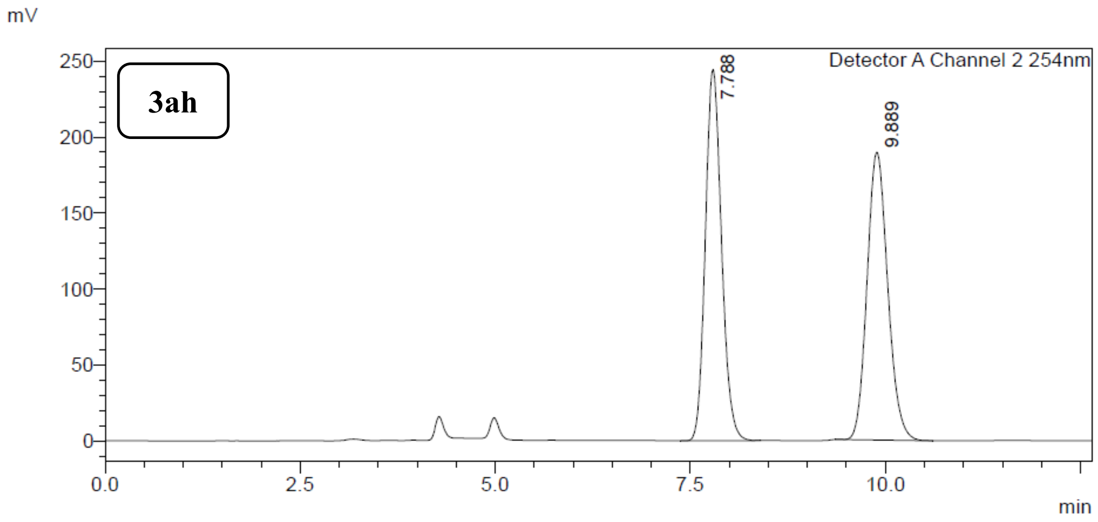

Detector A Channel 2 254nm

Peak#	Ret. Time	Area	Height	Conc.
1	7.788	3526435	244429	50.208
2	9.889	3497158	189474	49.792
Total		7023593	433903	

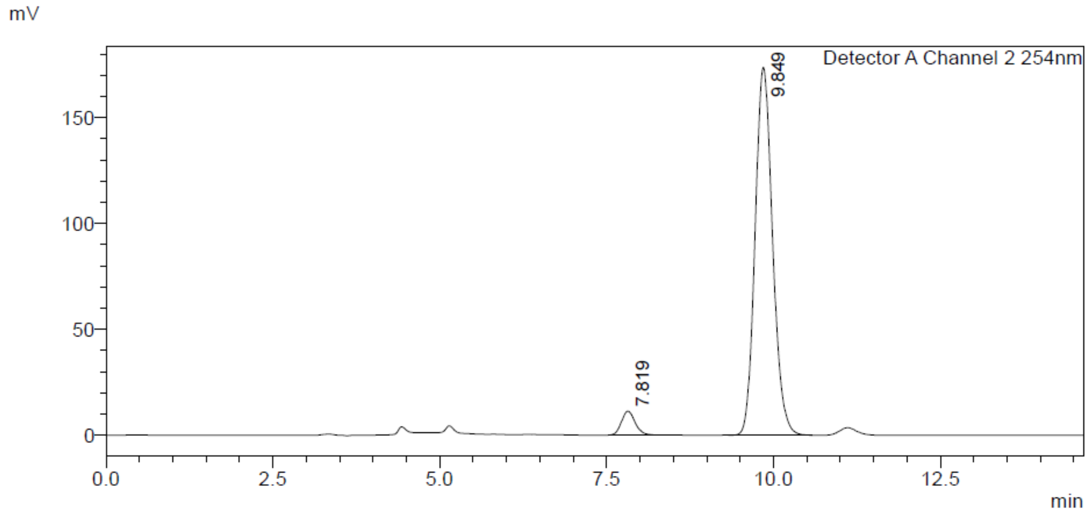

Detector A Channel 2 254nm

Peak#	Ret. Time	Area	Height	Conc.
1	7.819	156497	11175	4.714
2	9.849	3163633	173706	95.286
Total		3320130	184881	

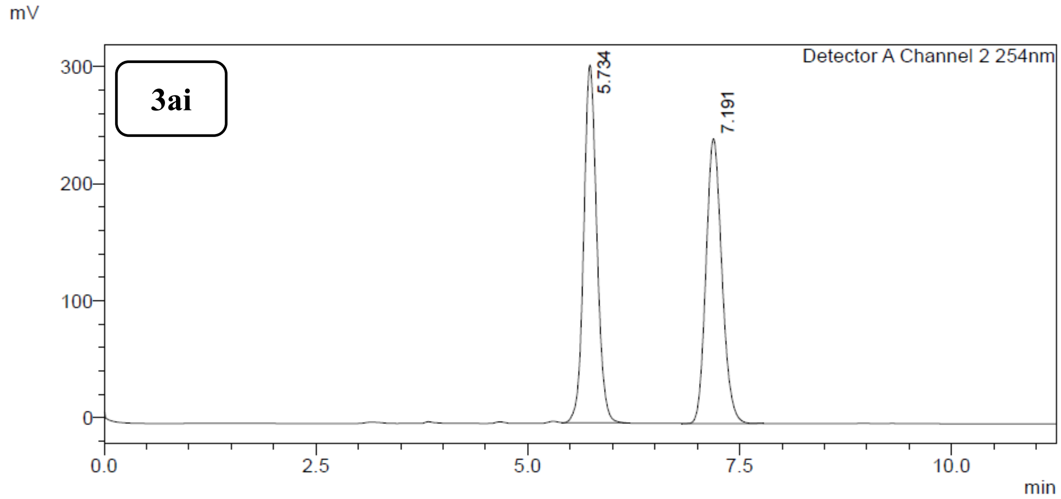

Detector A Channel 2 254nm

Peak#	Ret. Time	Area	Height	Conc.
1	5.734	3274741	305655	50.708
2	7.191	3183279	243478	49.292
Total		6458020	549132	

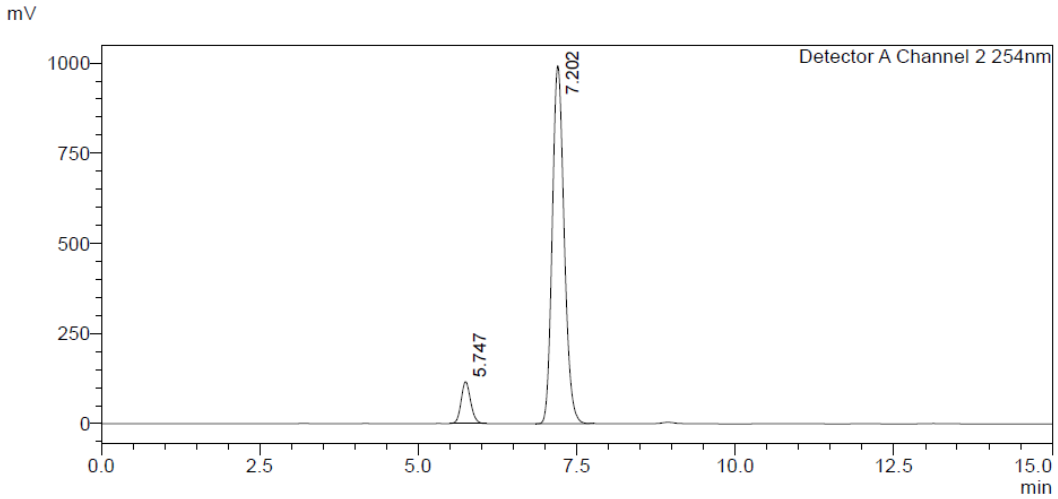

Detector A Channel 2 254nm

Peak#	Ret. Time	Area	Height	Conc.
1	5.747	1218680	115929	8.536
2	7.202	13058897	992803	91.464
Total		14277578	1108732	

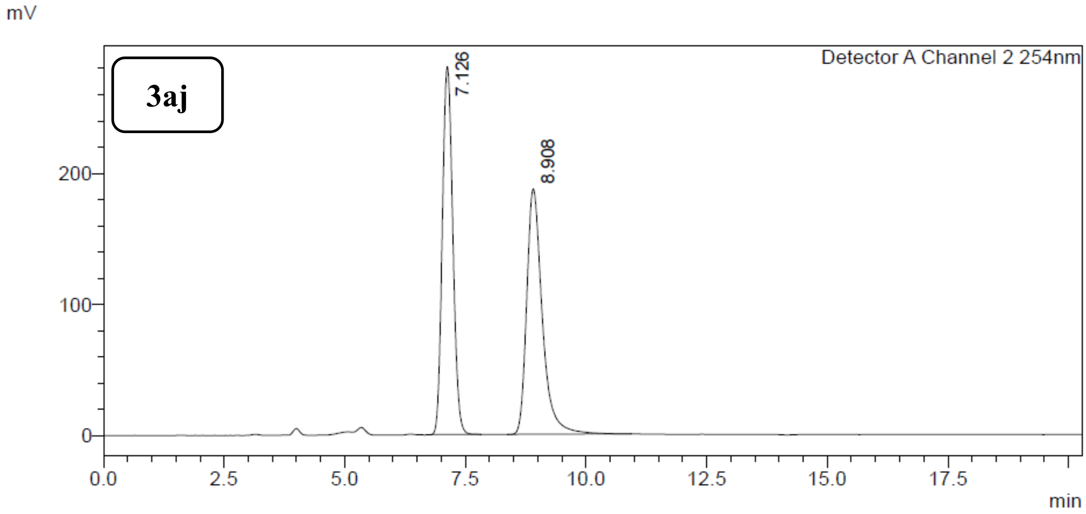

Detector A Channel 2 254nm

Peak#	Ret. Time	Area	Height	Conc.
1	7.126	4236827	280569	50.182
2	8.908	4206075	187105	49.818
Total		8442901	467674	

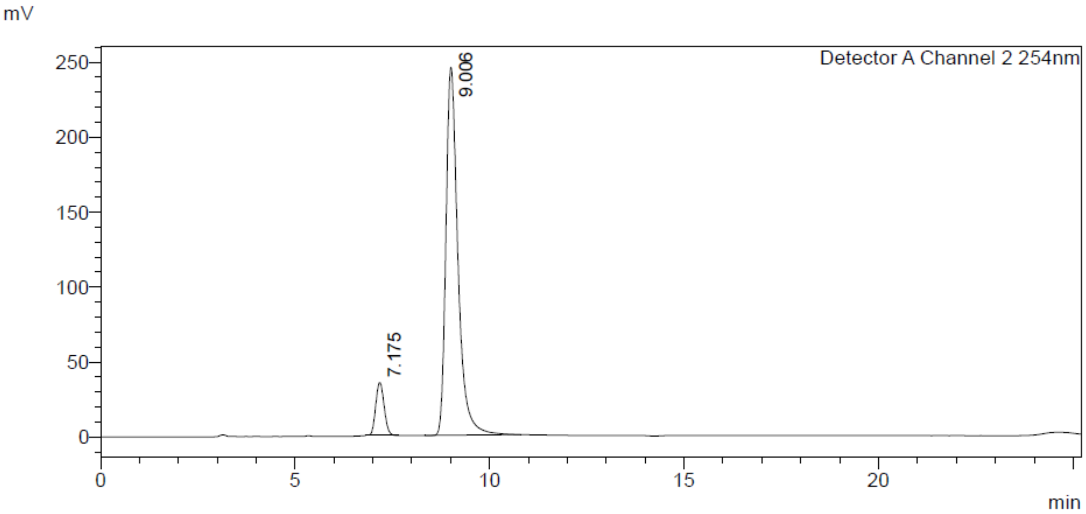

Detector A Channel 2 254nm

Peak#	Ret. Time	Area	Height	Conc.
1	7.175	531754	35330	9.155
2	9.006	5276705	245530	90.845
Total		5808459	280860	

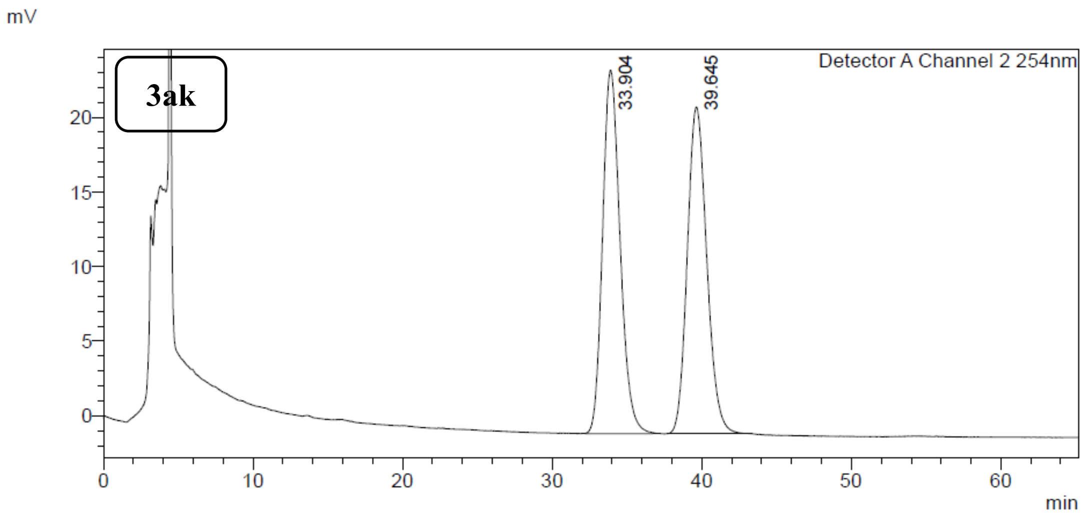

Detector A Channel 2 254nm

Peak#	Ret. Time	Area	Height	Conc.
1	33.904	1985485	24371	49.997
2	39.645	1985762	21925	50.003
Total		3971247	46296	

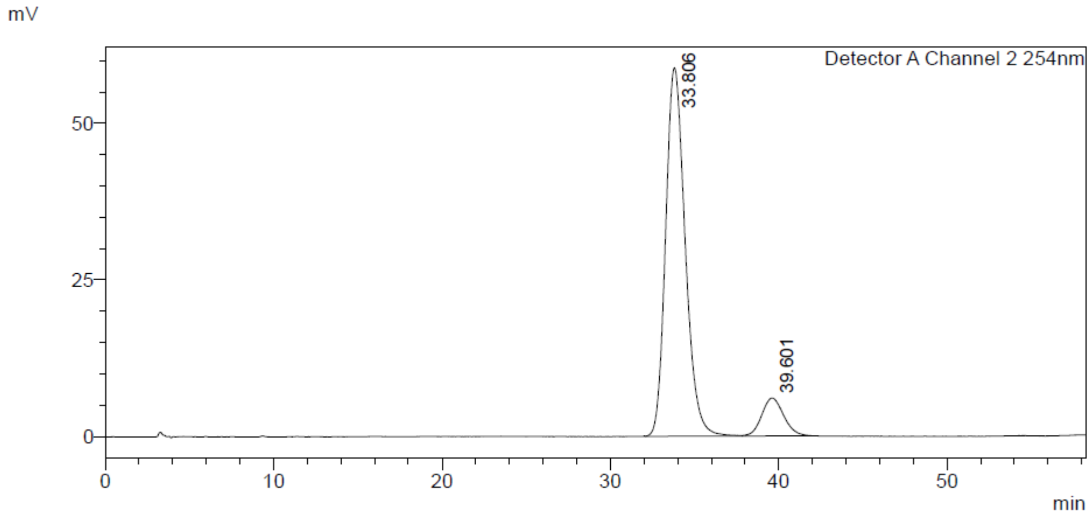

Detector A Channel 2 254nm

Peak#	Ret. Time	Area	Height	Conc.
1	33.806	4721669	58711	89.951
2	39.601	527465	5956	10.049
Total		5249134	64667	

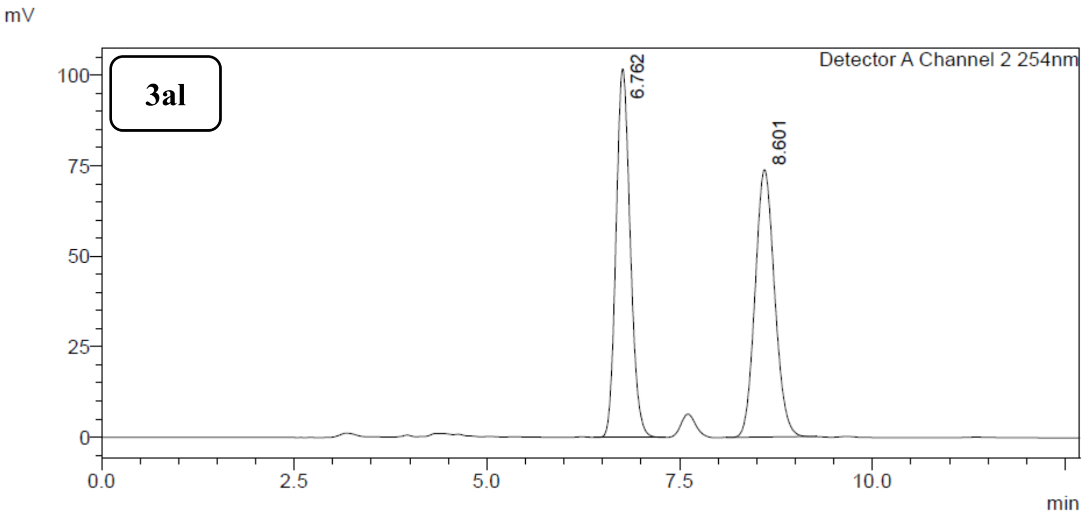

Detector A Channel 2 254nm

Peak#	Ret. Time	Area	Height	Conc.
1	6.762	1305601	101731	50.053
2	8.601	1302850	73815	49.947
Total		2608450	175546	

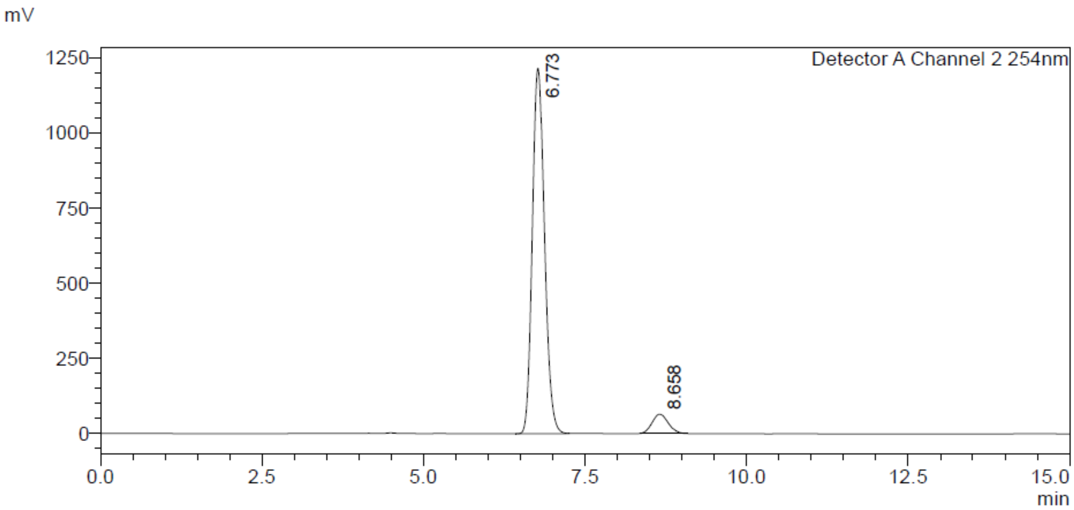

Detector A Channel 2 254nm

Peak#	Ret. Time	Area	Height	Conc.
1	6.773	15823613	1216293	93.360
2	8.658	1125403	64492	6.640
Total		16949016	1280785	

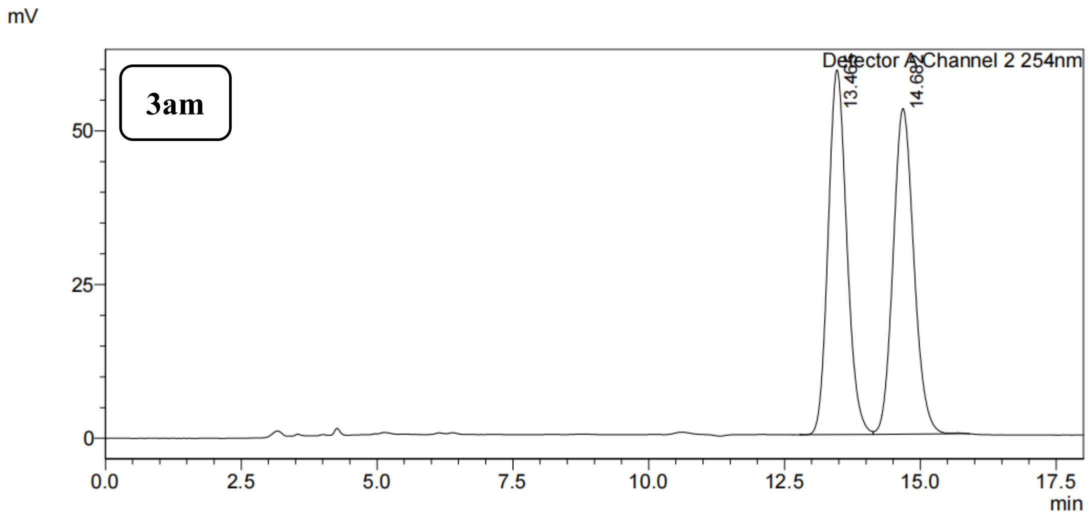

Detector A Channel 2 254nm

Peak#	Ret. Time	Area	Height	Conc.
1	13.465	1415424	59285	50.088
2	14.682	1410461	52949	49.912
Total		2825886	112234	

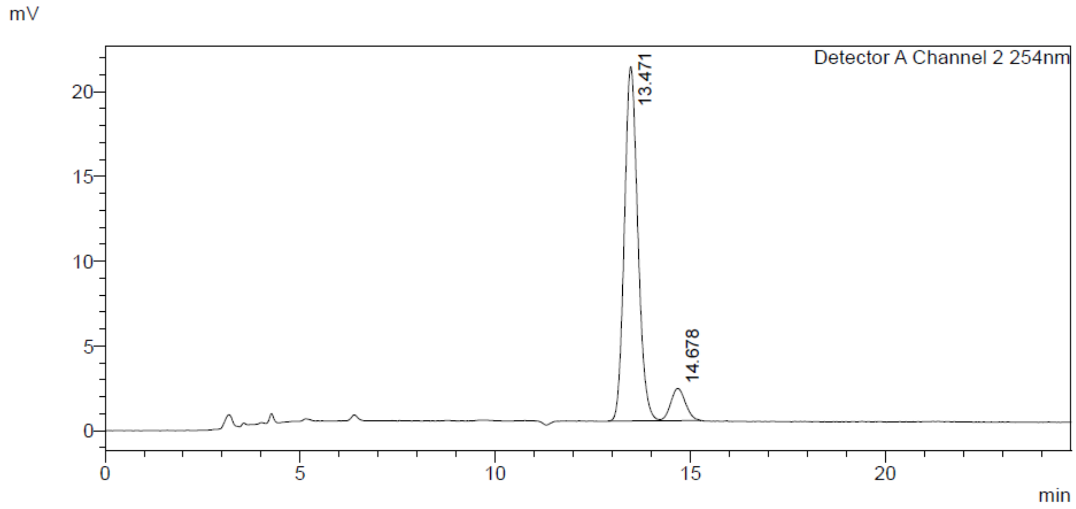

Detector A Channel 2 254nm

Peak#	Ret. Time	Area	Height	Conc.
1	13.471	501454	20889	91.022
2	14.678	49461	1894	8.978
Total		550915	22783	

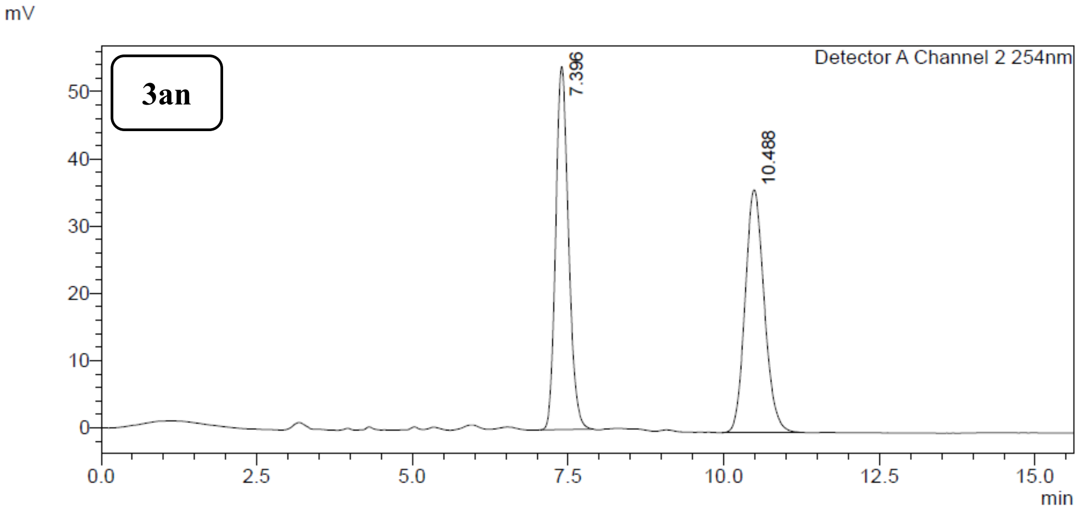

Detector A Channel 2 254nm

Peak#	Ret. Time	Area	Height	Conc.
1	7.396	775033	53995	50.071
2	10.488	772839	36103	49.929
Total		1547872	90098	

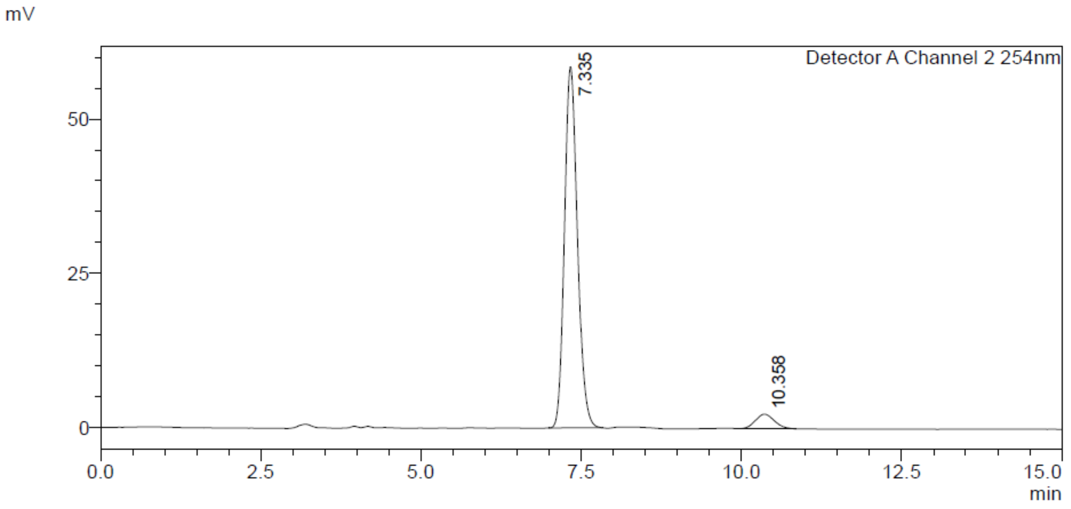

Detector A Channel 2 254nm

Peak#	Ret. Time	Area	Height	Conc.
1	7.335	825285	58554	94.567
2	10.358	47411	2332	5.433
Total		872695	60886	

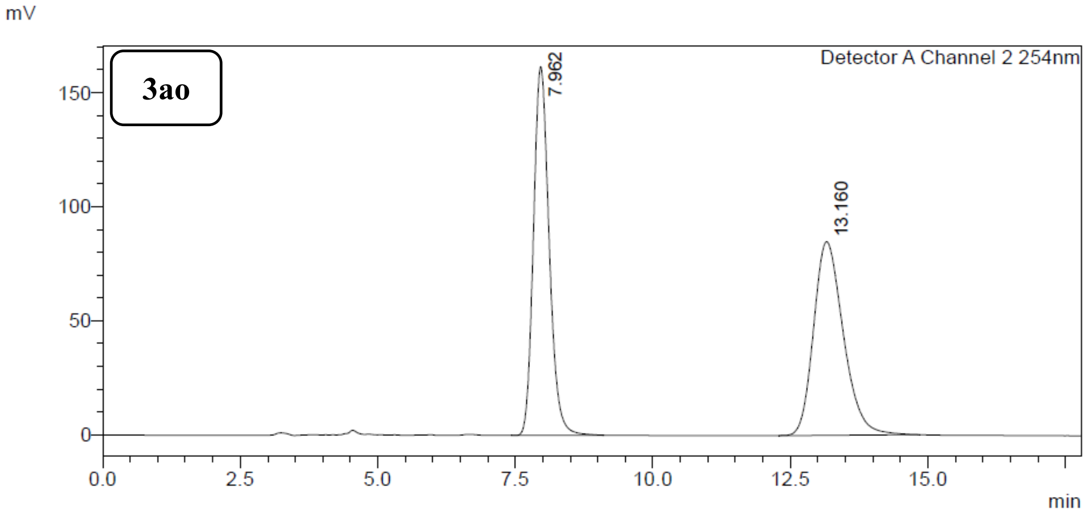

Detector A Channel 2 254nm

Peak#	Ret. Time	Area	Height	Conc.
1	7.962	3210110	161533	50.126
2	13.160	3193925	84863	49.874
Total		6404035	246397	

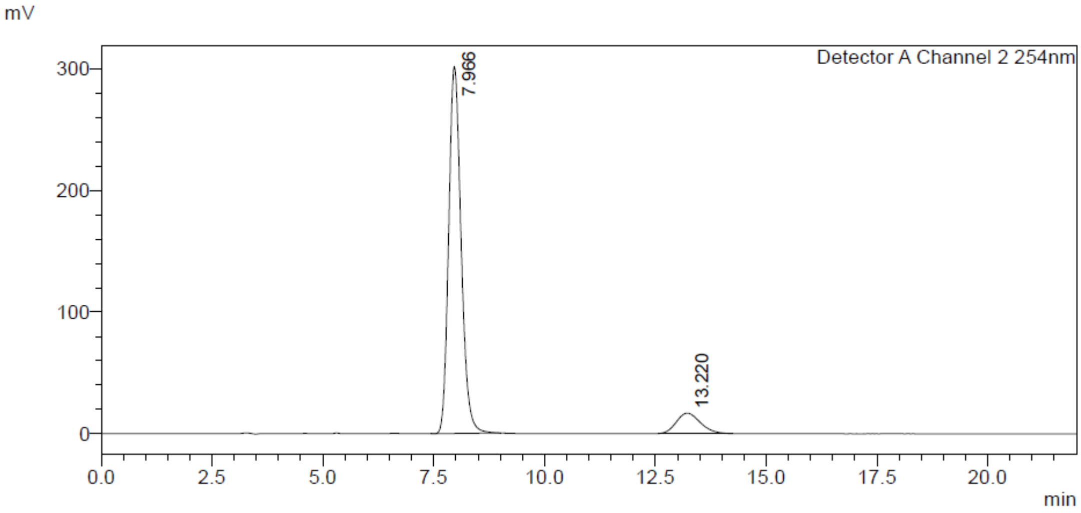

Detector A Channel 2 254nm

Peak#	Ret. Time	Area	Height	Conc.
1	7.966	5873365	301525	90.310
2	13.220	630216	16738	9.690
Total		6503581	318263	

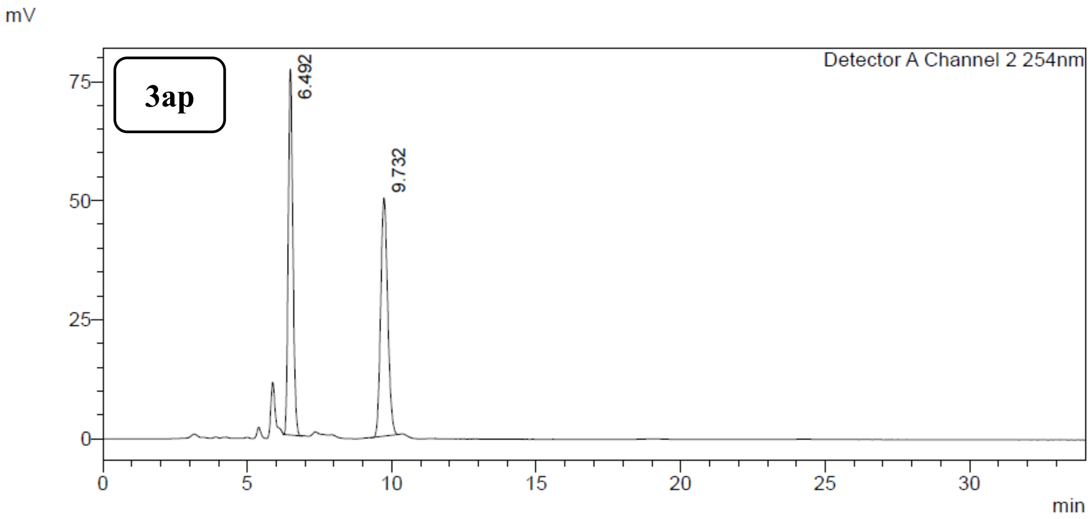

Detector A Channel 2 254nm

Peak#	Ret. Time	Area	Height	Conc.
1	6.492	858261	76814	50.328
2	9.732	847062	50018	49.672
Total		1705323	126832	

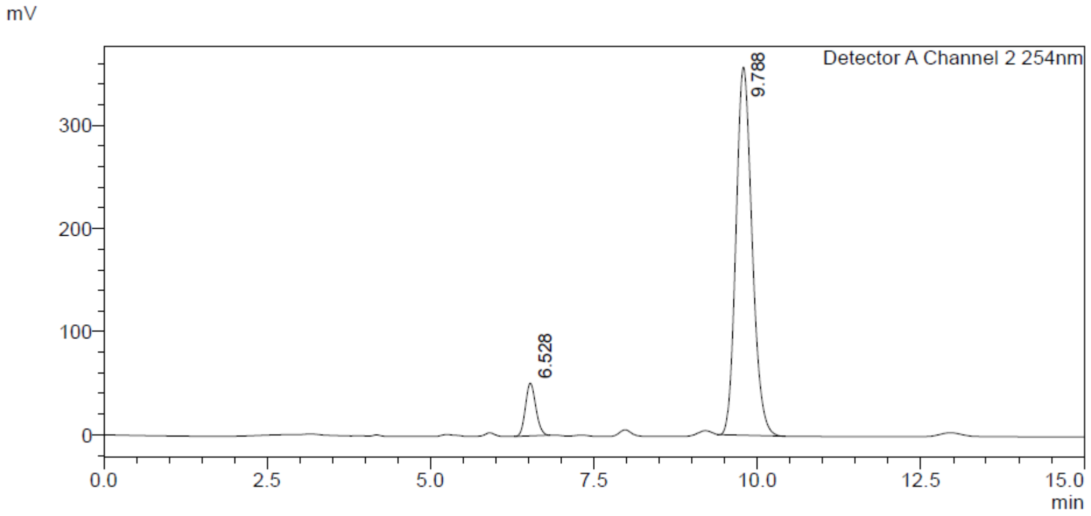

Detector A Channel 2 254nm

Peak#	Ret. Time	Area	Height	Conc.
1	6.528	571279	51067	8.488
2	9.788	6158876	356743	91.512
Total		6730155	407810	

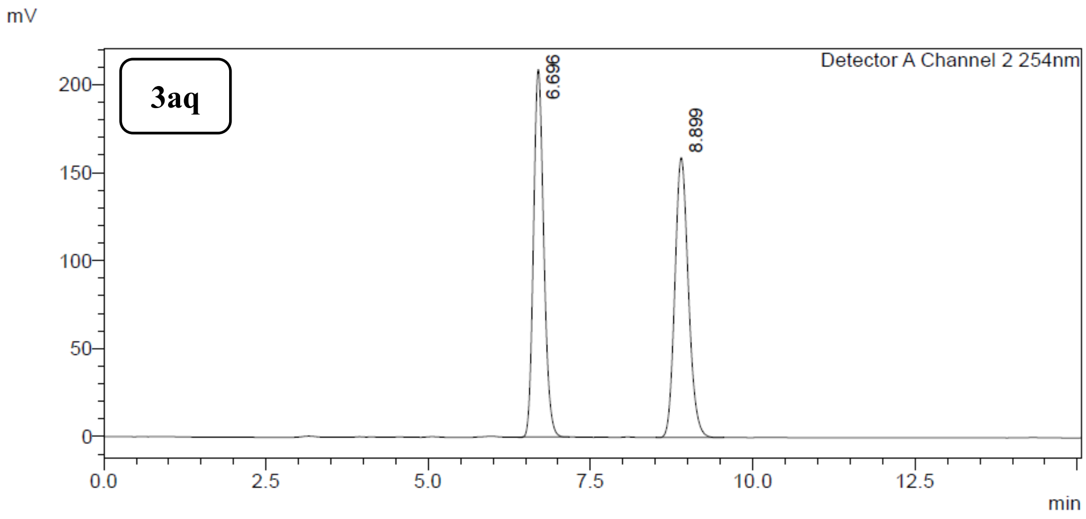

Detector A Channel 2 254nm

Peak#	Ret. Time	Area	Height	Conc.
1	6.696	2303088	208863	49.905
2	8.899	2311873	159158	50.095
Total		4614961	368021	

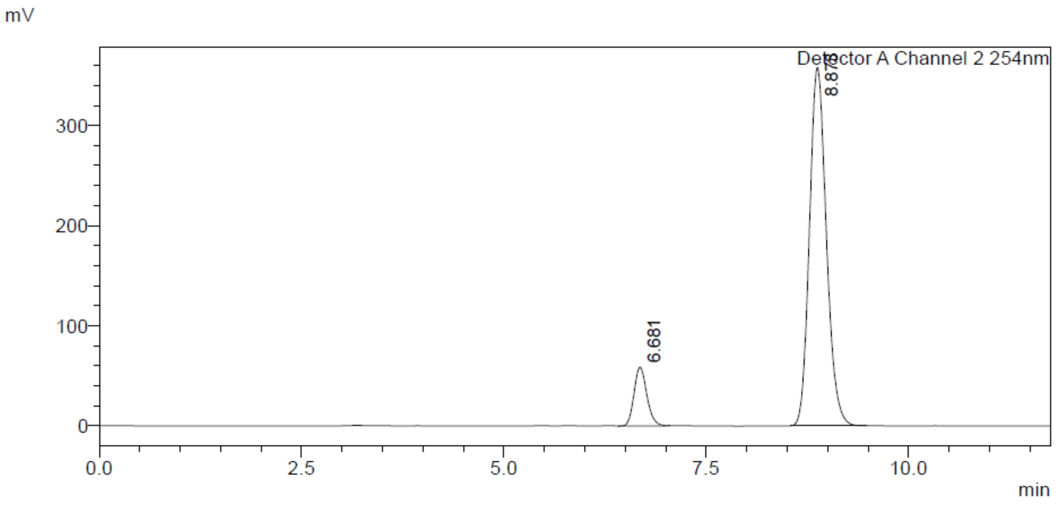

Detector A Channel 2 254nm

Peak#	Ret. Time	Area	Height	Conc.
1	6.681	644790	58692	11.030
2	8.873	5201186	357785	88.970
Total		5845976	416477	

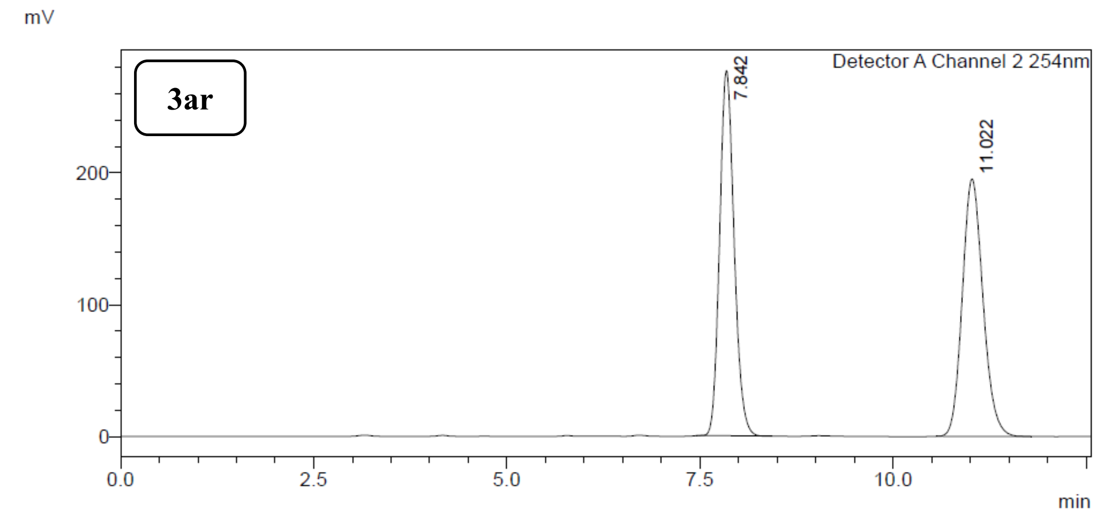

Detector A Channel 2 254nm

Peak#	Ret. Time	Area	Height	Conc.
1	7.842	3616325	276757	49.824
2	11.022	3641928	195118	50.176
Total		7258253	471875	

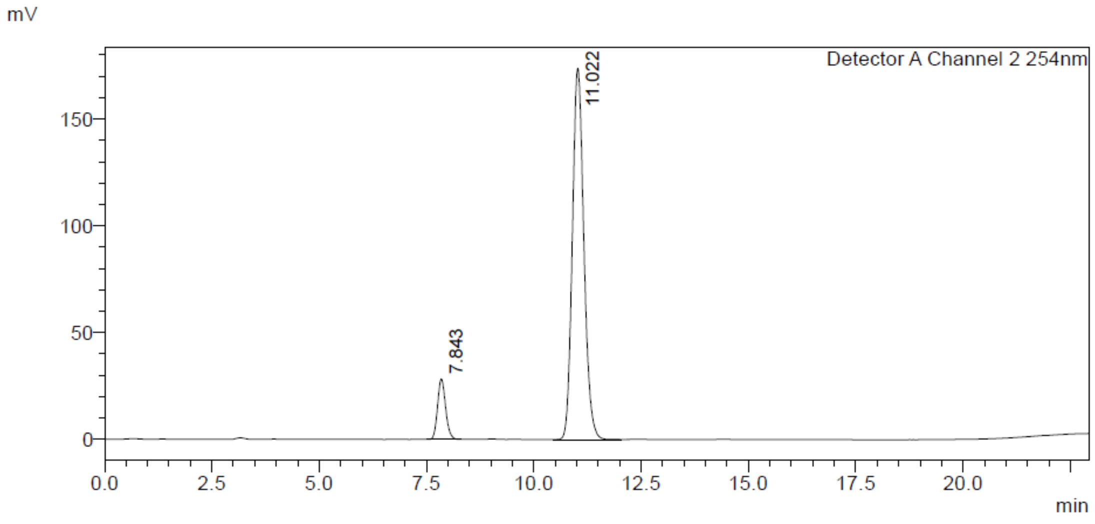

Detector A Channel 2 254nm

Peak#	Ret. Time	Area	Height	Conc.
1	7.843	369086	28281	10.208
2	11.022	3246567	173714	89.792
Total		3615652	201994	

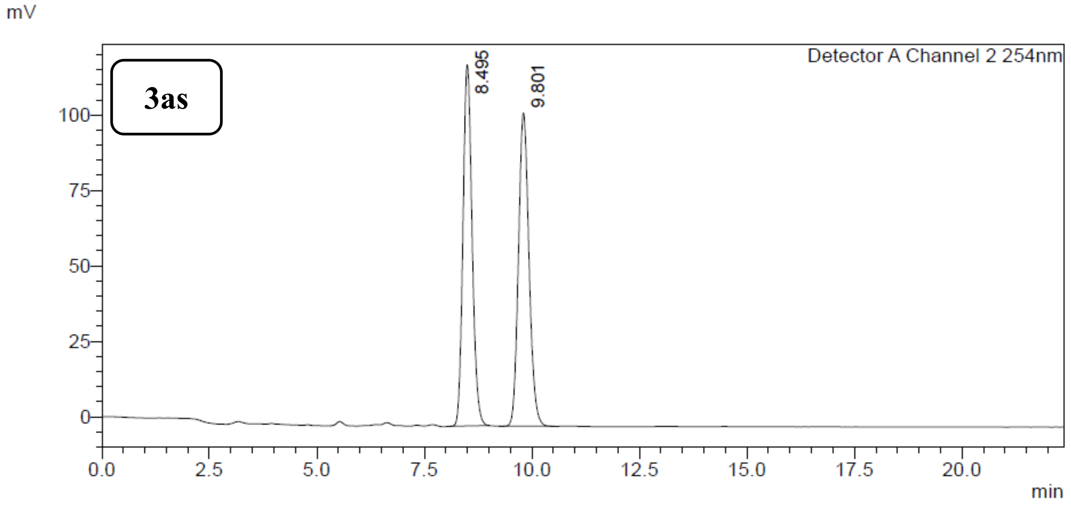

Detector A Channel 2 254nm

Peak#	Ret. Time	Area	Height	Conc.
1	8.495	1765149	119673	49.942
2	9.801	1769246	103846	50.058
Total		3534395	223518	

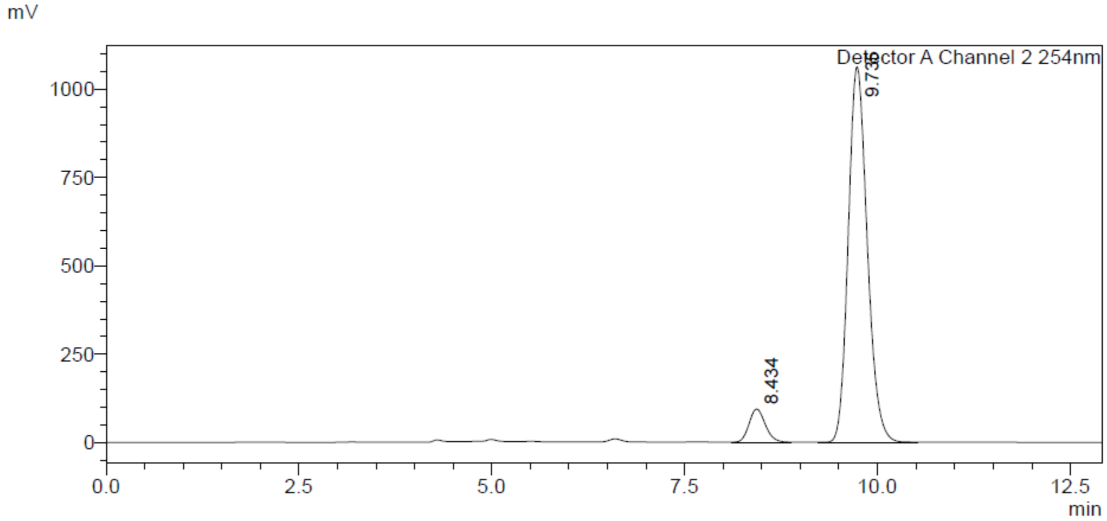

Detector A Channel 2 254nm

Peak#	Ret. Time	Area	Height	Conc.
1	8.434	1377306	94019	7.010
2	9.735	18271286	1063314	92.990
Total		19648592	1157333	

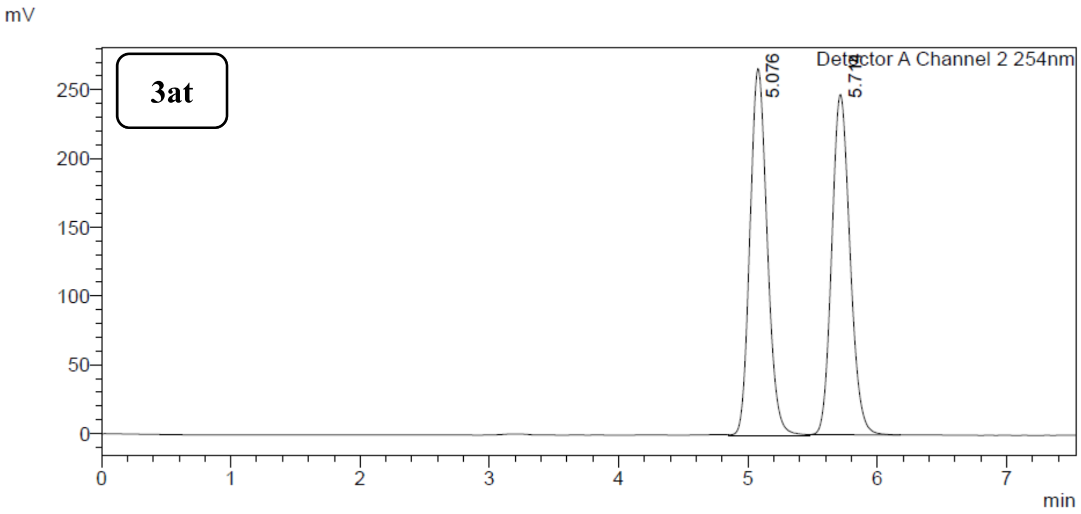

Detector A Channel 2 254nm

Peak#	Ret. Time	Area	Height	Conc.
1	5.076	2454812	266672	50.226
2	5.714	2432748	247286	49.774
Total		4887561	513959	

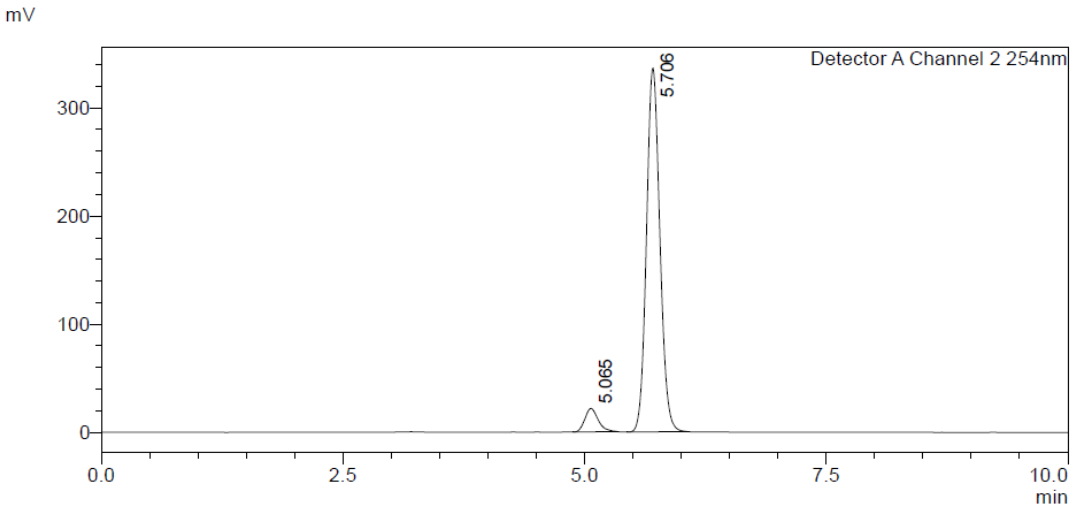

Detector A Channel 2 254nm

Peak#	Ret. Time	Area	Height	Conc.
1	5.065	206217	21662	5.946
2	5.706	3261986	336556	94.054
Total		3468203	358219	

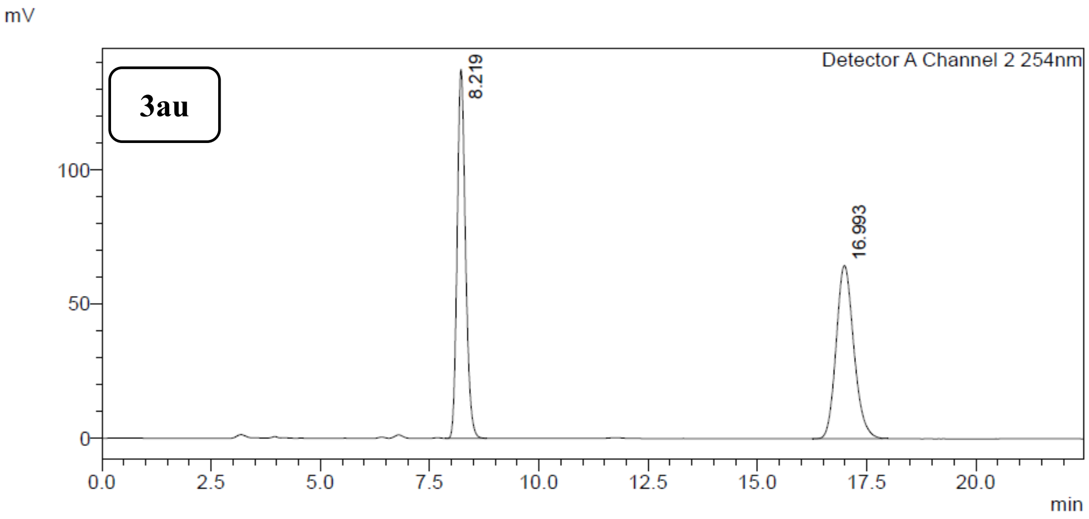

Detector A Channel 2 254nm

Peak#	Ret. Time	Area	Height	Conc.
1	8.219	1826789	137558	50.075
2	16.993	1821281	64581	49.925
Total		3648070	202138	

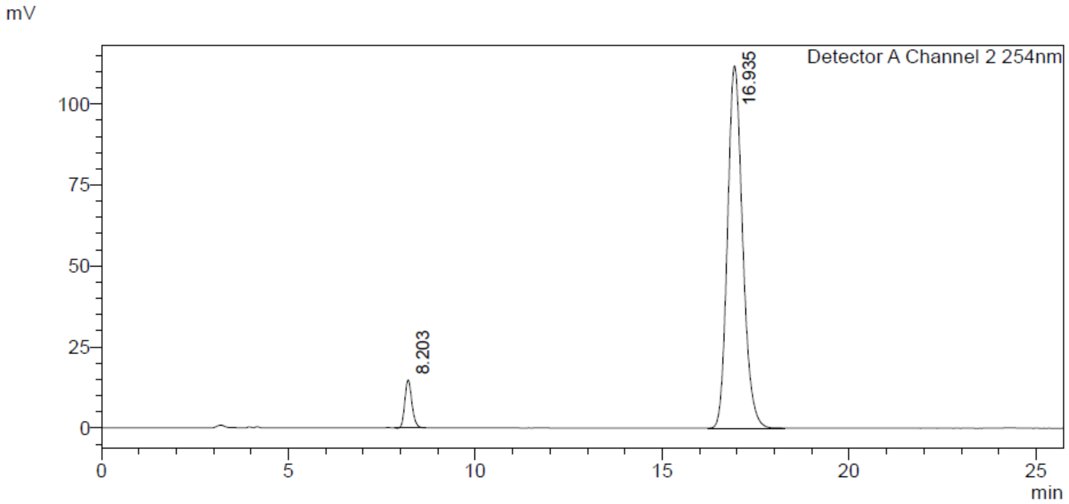

Detector A Channel 2 254nm

Peak#	Ret. Time	Area	Height	Conc.
1	8.203	195656	14850	5.840
2	16.935	3154390	111840	94.160
Total		3350046	126690	

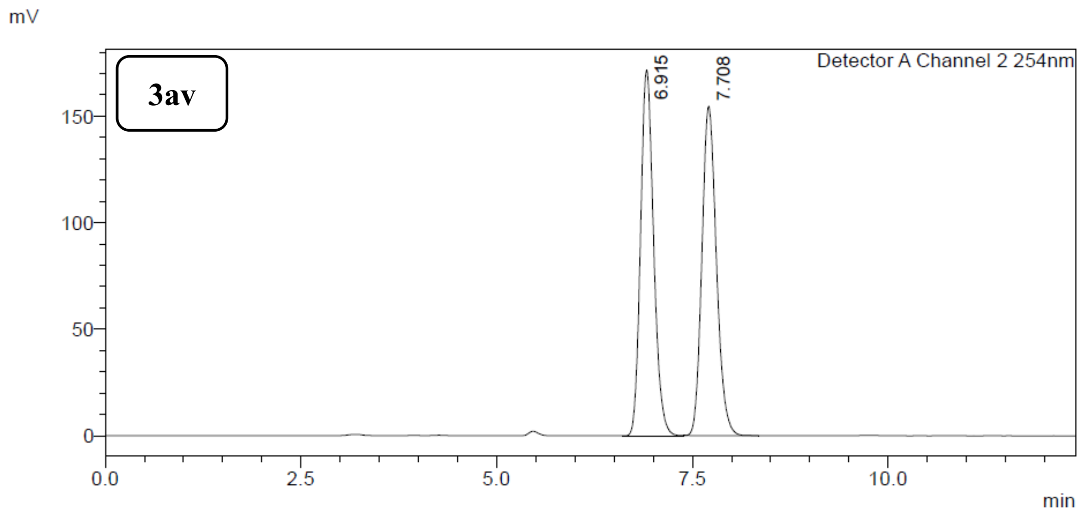

Detector A Channel 2 254nm

Peak#	Ret. Time	Area	Height	Conc.
1	6.915	1999716	171775	50.061
2	7.708	1994854	154694	49.939
Total		3994571	326469	

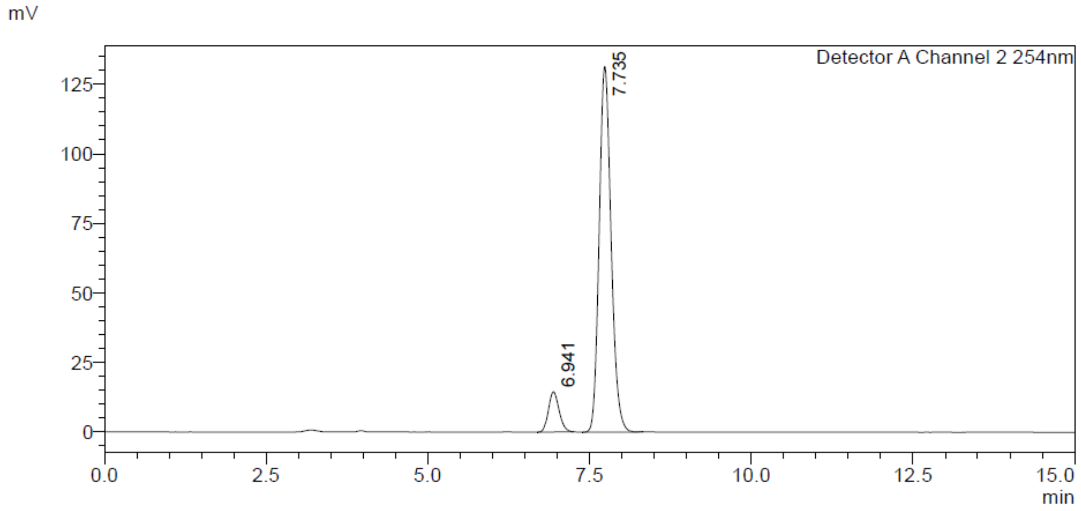

Detector A Channel 2 254nm

Peak#	Ret. Time	Area	Height	Conc.
1	6.941	165966	14462	8.906
2	7.735	1697571	131416	91.094
Total		1863536	145877	

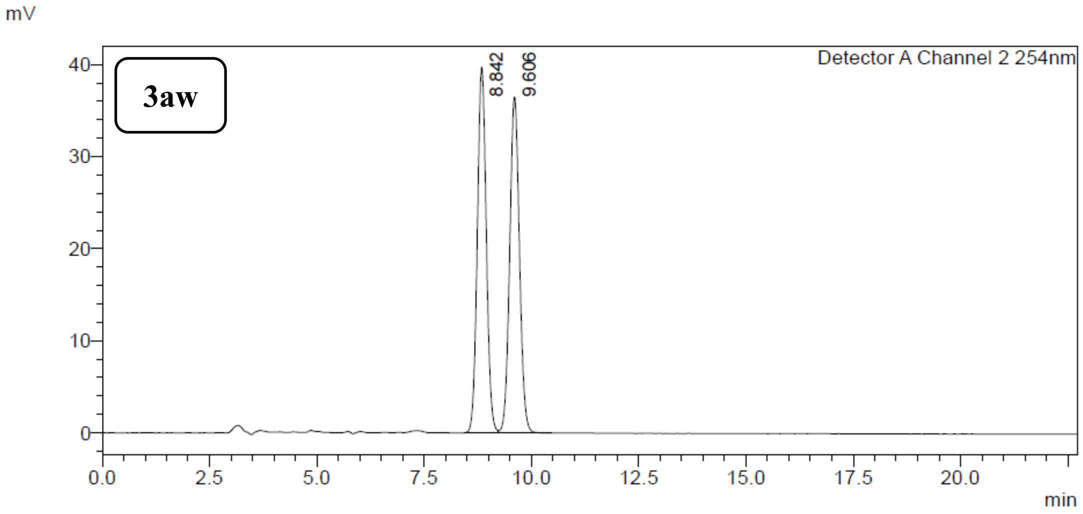

Detector A Channel 2 254nm

Peak#	Ret. Time	Area	Height	Conc.
1	8.842	579644	39751	49.956
2	9.606	580672	36512	50.044
Total		1160317	76263	

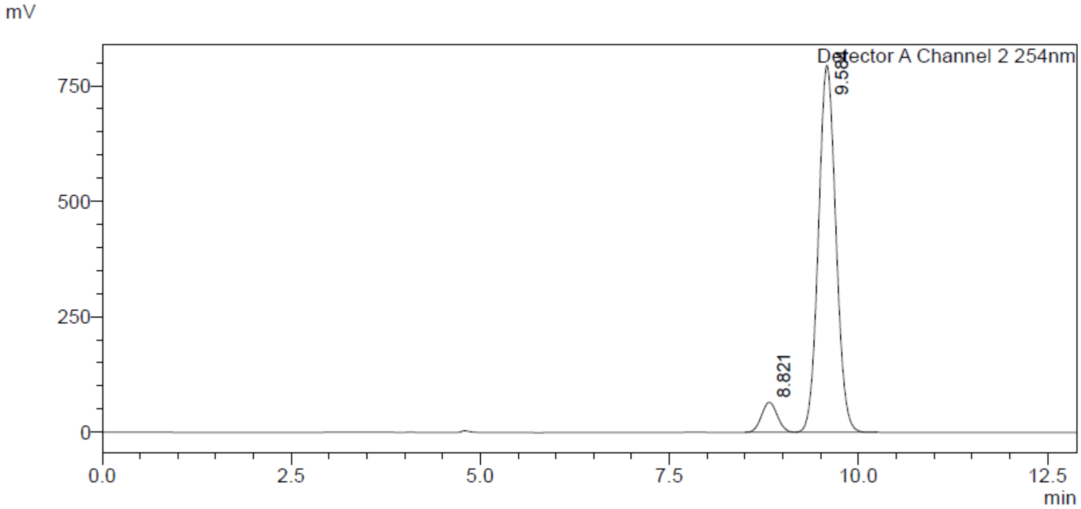

Detector A Channel 2 254nm

Peak#	Ret. Time	Area	Height	Conc.
1	8.821	942275	64848	6.868
2	9.584	12777700	794157	93.132
Total		13719975	859006	

3ax

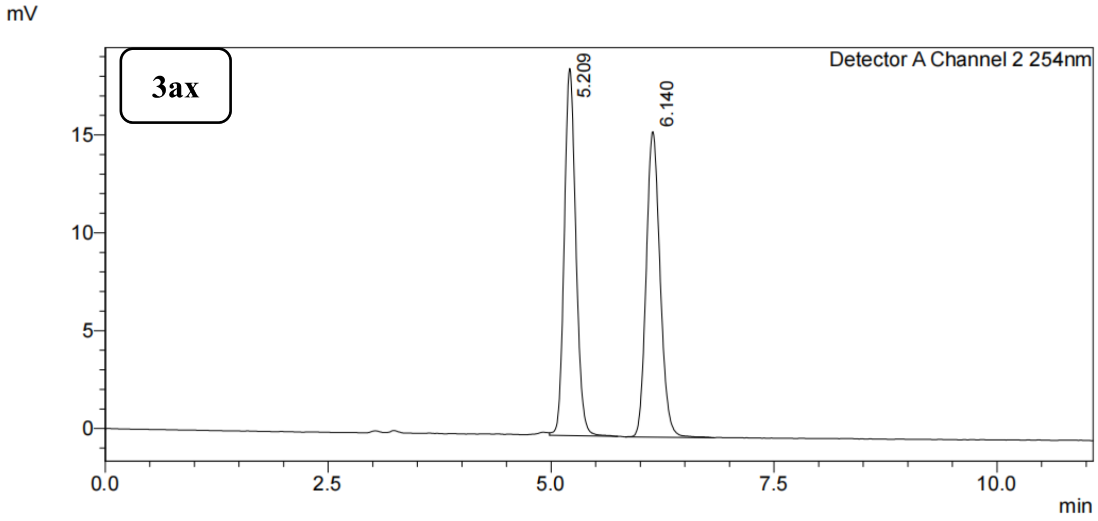

Detector A Channel 2 254nm

Peak#	Ret. Time	Area	Height	Conc.
1	5.209	169543	18753	50.017
2	6.140	169427	15609	49.983
Total		338969	34362	

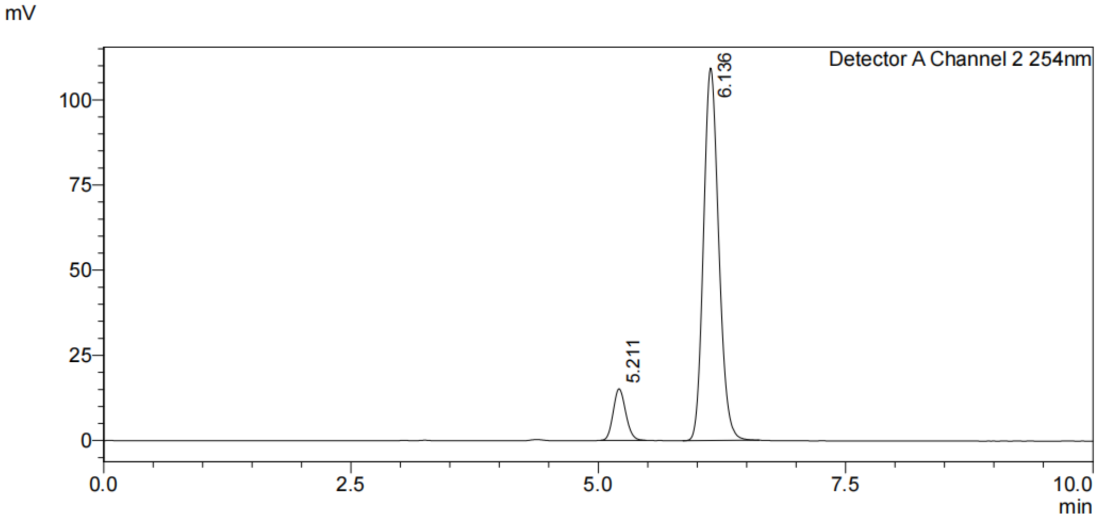

Detector A Channel 2 254nm

Peak#	Ret. Time	Area	Height	Conc.
1	5.211	133925	15125	10.203
2	6.136	1178613	109338	89.797
Total		1312538	124463	

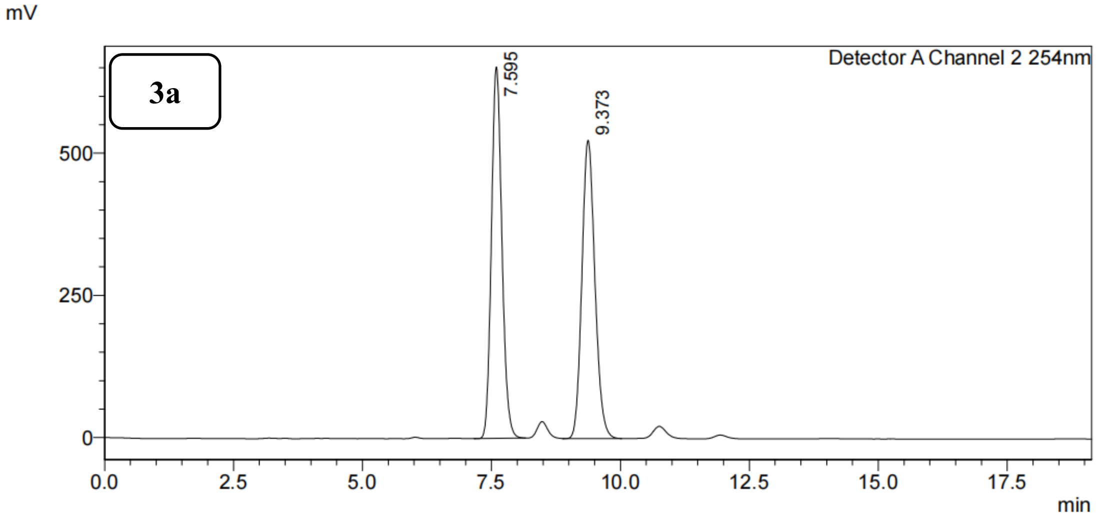

Detector A Channel 2 254nm

Peak#	Ret. Time	Area	Height	Conc.
1	7.595	9242303	652850	50.085
2	9.373	9210897	524227	49.915
Total		18453200	1177077	

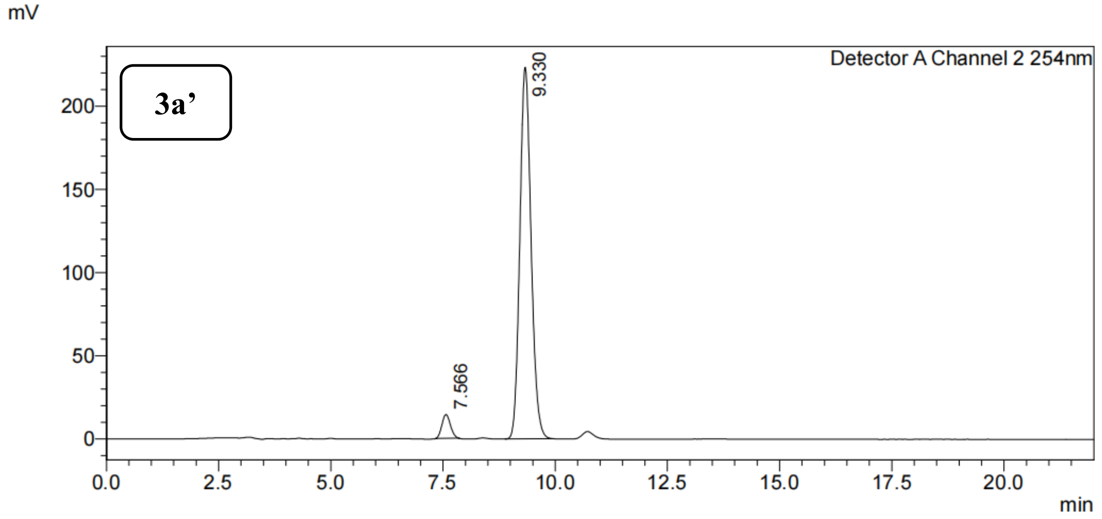

Detector A Channel 2 254nm

Peak#	Ret. Time	Area	Height	Conc.
1	7.566	188014	14315	4.610
2	9.330	3890174	223367	95.390
Total		4078187	237683	

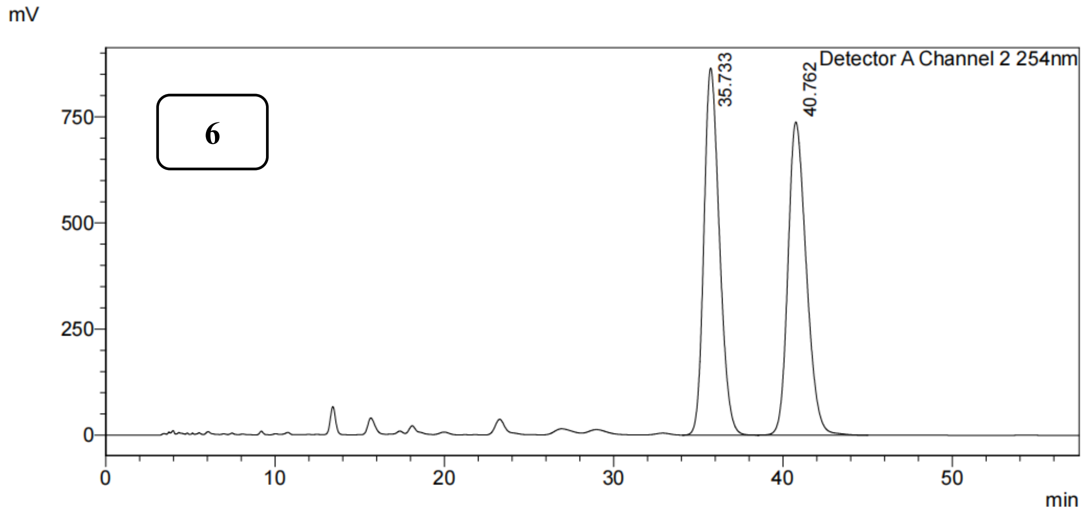

Detector A Channel 2 254nm

Peak#	Ret. Time	Area	Height	Conc.
1	35.733	55166610	864299	49.791
2	40.762	55628910	737990	50.209
Total		110795520	1602289	

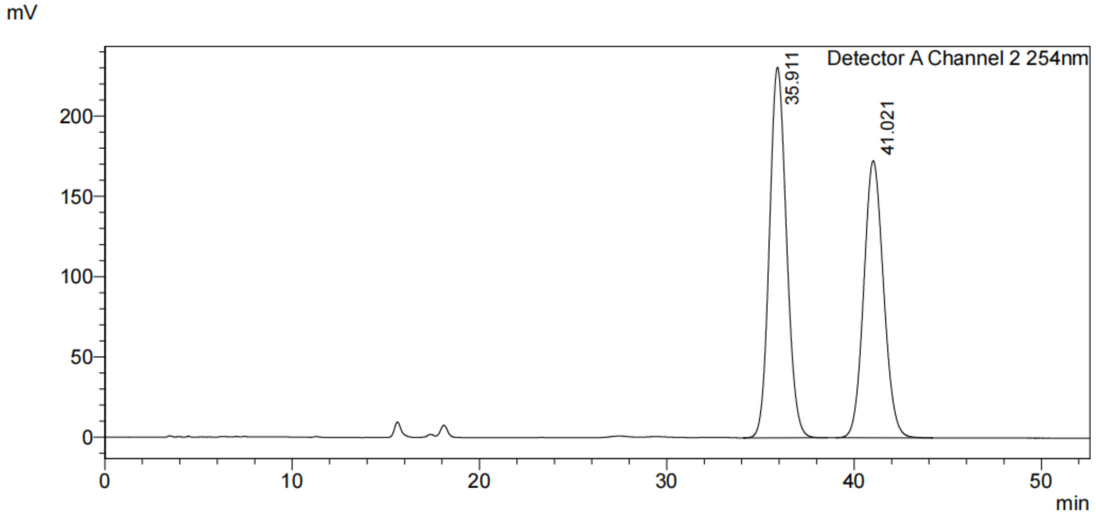

Detector A Channel 2 254nm

Peak#	Ret. Time	Area	Height	Conc.
1	35.911	14635244	230860	53.564
2	41.021	12687448	172632	46.436
Total		27322692	403492	

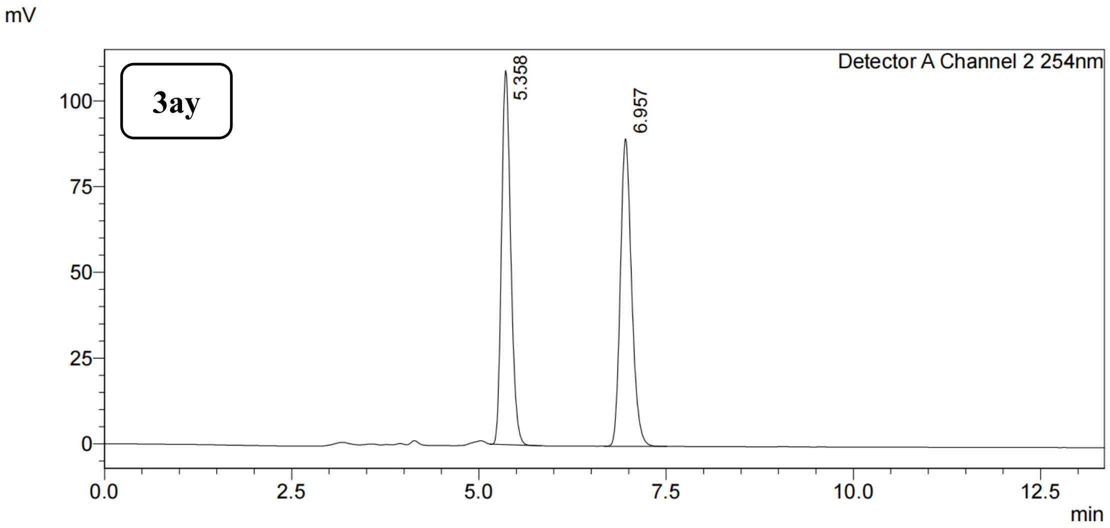

Detector A Channel 2 254nm

Peak#	Ret. Time	Area	Height	Conc.
1	5.358	931992	109049	49.808
2	6.957	939167	89603	50.192
Total		1871160	198652	

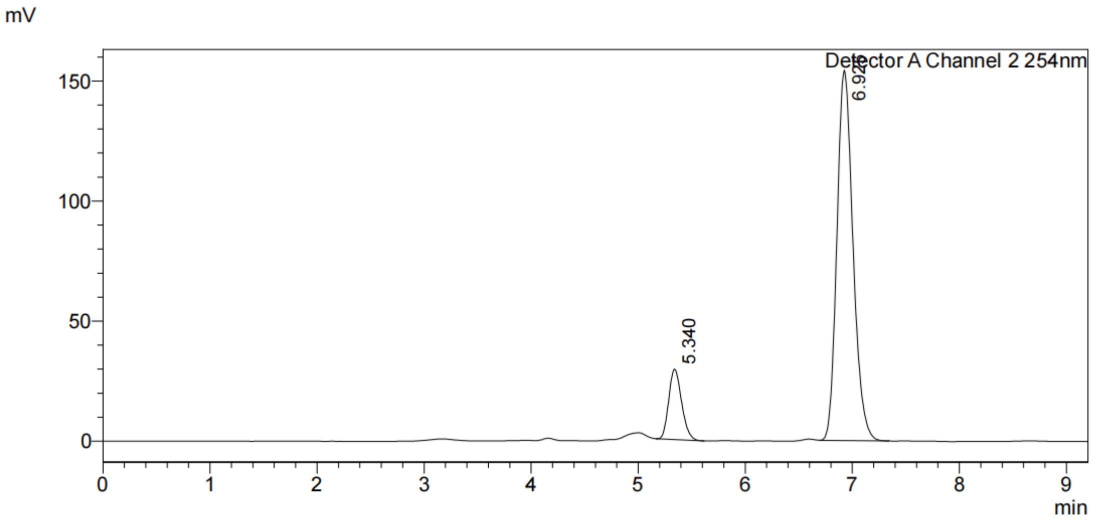

Detector A Channel 2 254nm

Peak#	Ret. Time	Area	Height	Conc.
1	5.340	245288	29326	13.229
2	6.925	1608819	154195	86.771
Total		1854107	183521	
